# H-NS Mediates the Silencing of Laterally Acquired Genes in Bacteria

**DOI:** 10.1371/journal.ppat.0020081

**Published:** 2006-08-18

**Authors:** Sacha Lucchini, Gary Rowley, Martin D Goldberg, Douglas Hurd, Marcus Harrison, Jay C. D Hinton

**Affiliations:** 1 Molecular Microbiology Group, Institute of Food Research, Colney, Norwich, United Kingdom; 2 Oxford Gene Technology, Yarnton, Oxford, United Kingdom; Tufts University School of Medicine, United States of America

## Abstract

Histone-like nucleoid structuring protein (H-NS) is a modular protein that is associated with the bacterial nucleoid. We used chromatin immunoprecipitation to determine the binding sites of H-NS and RNA polymerase on the Salmonella enterica serovar Typhimurium chromosome. We found that H-NS does not bind to actively transcribed genes and does not co-localize with RNA polymerase. This shows that H-NS principally silences gene expression by restricting the access of RNA polymerase to the DNA. H-NS had previously been shown to preferentially bind to curved DNA in vitro. In fact, at the genomic level we discovered that the level of H-NS binding correlates better with the AT-content of DNA. This is likely to have evolutionary consequences because we show that H-NS binds to many *Salmonella* genes acquired by lateral gene transfer, and functions as a gene silencer. The removal of H-NS from the cell causes un-controlled expression of several *Salmonella* pathogenicity islands, and we demonstrate that this has deleterious consequences for bacterial fitness. Our discovery of this novel role for H-NS may have implications for the acquisition of foreign genes by enteric bacteria**.**

## Synopsis

In recent decades, gene silencing has been well-characterised in plants and animals, and involves the prevention of transcription by DNA-methylation and histone-modification, or interference with translation by small RNA molecules. This issue of PLoS Pathogens reports the discovery that global gene silencing also occurs in bacteria. The novel mechanism is mediated by the highly abundant histone-like nucleoid structuring protein (H-NS), which blocks the expression of 254 genes in wild-type *Salmonella*. Many of these genes were acquired through horizontal gene transfer, including pathogenicity islands, and these are silenced by the binding of H-NS to AT-rich chromosomal regions. The study reveals that H-NS prevents the uncontrolled transcription of genes within pathogenicity islands to ensure that bacterial fitness is maintained. It is suggested that H-NS plays a role in bacterial evolution by influencing both the acquisition and maintenance of foreign DNA.

## Introduction

Bacteria have an extraordinary capacity to adapt to changes in their external environment. The histone-like nucleoid-structuring protein (H-NS) plays a key role for many Gram-negative bacteria by integrating a complex range of environmental signals such as temperature and osmolarity [1,2]. Consequently, H-NS acts as a pleiotropic regulator of gene expression [3]. Deletion of *hns* resulted in altered gene expression of almost 5% of Escherichia coli genes, of which more than 80% were up-regulated [4]. H-NS also influences the major DNA transactions, such as replication, transposition and recombination [5]. The modular nature of the H-NS protein is reflected by the three distinct domains, involved in DNA binding, dimerisation, and the formation of high order oligomers [3,6].

Unlike classical regulatory repressor and activator proteins, H-NS does not recognise particular sequences of DNA; however, a number of in vitro studies have shown that the protein has a small preference for curved DNA [7–9]. It has been proposed that after binding to a preferential site, H-NS oligomerizes cooperatively along the DNA to cover a larger region [10–12]. This idea is supported by the observation that mutant H-NS proteins that are unable to oligomerize no longer repress the expression of some promoters [10,13]. Following the binding of H-NS, changes in transcription are likely to be caused by modulations of DNA topology (i.e. compacting and looping DNA, and the constraint of DNA supercoils [14]).

As DNA curvature is most pronounced around the transcriptional start of genes [15] a simple model involving the preferential binding of H-NS to regulatory regions has been considered [16], with resultant effects upon global gene expression. However, since curved DNA is found near most promoters [17], and the majority of bacterial promoters are not regulated by H-NS (i.e. H-NS-independent), it has never been clear how specificity was achieved. The interaction between H-NS and DNA has been studied in vitro by traditional gel retardation and DNA-footprinting experiments, but knowledge of how and where H-NS attaches to DNA in vivo has remained indirect. Using chromatin immunoprecipitation on chip (ChIP-on-chip) [18,19], we have now addressed the question of where H-NS binds to DNA in the macro-molecular context of a living bacterial cell and show how this correlates with the distribution of RNA polymerase on the chromosome.

## Results/Discussion

**Figure 1:**
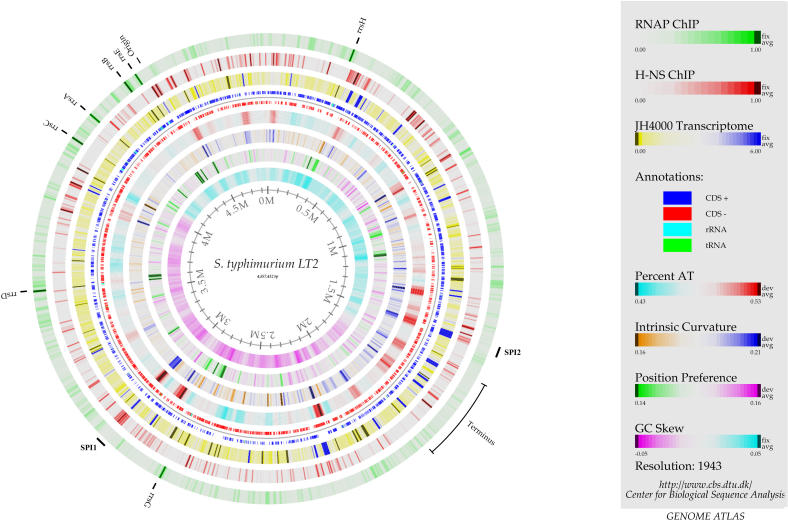
H-NS Chip and Transcriptomic Data in Context of the Genome Atlas for the S. typhimurium Chromosome The data from the RNAP and H-NS ChIP-on-chip experiments are shown using coloured concentric circles which are mapped onto the *Salmonella* chromosome. In addition, we show our previously published transcriptome data for the JH4000 *hns*-deletion mutant [2] and four DNA structural measures (http://www.cbs.dtu.dk/services/GenomeAtlas [15]). The order of the concentric circles is indicated in the legend. Numbers on the inside of the innermost circle are the location relative to position zero measured in millions of base-pairs (Mbp). The origin of replication, predicted terminus region, location of rRNAs and the SPI1 and SPI2 pathogenicity islands are indicated at the edge of the outermost circle.

**Figure 2:**
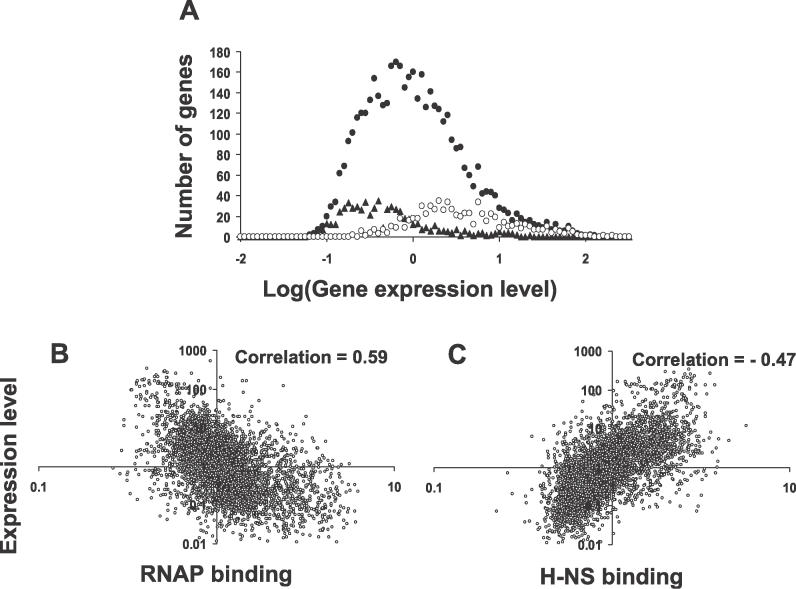
Correlation between Binding of H-NS, RNAP, and Level of Gene Expression (A) Frequency distribution of relative gene expression levels for *S. typhimurium.* The results are shown for all S. typhimurium genes (filled circle) and for genes found to co-localize with H-NS (filled triangle) or RNAP (open circle) in the ChIP-on-chip experiment. Relative gene expression levels for S. typhimurium strain LT2 were taken from Ono et al [2]. (B and C) Scatter plot correlating binding of RNAP or H-NS to gene expression levels. The relative expression level of a gene (*y-*axis) is plotted against the corresponding level of binding to RNAP or H-NS (*x-*axis) as measured in the ChIP-on-chip experiments (see Materials and Methods).

H-NS and RNA polymerase binding sites on the chromosome of live Salmonella enterica serovar Typhimurium (S. typhimurium) LT2 cells were identified by ChIP-on-chip, using a tiled oligonucleotide microarray (Figure 1, outer circles**)**. We first identified 495 chromosomal regions that bound RNA polymerase (RNAP) during exponential growth in Luria Broth (LB, Materials and Methods), corresponding to 965 genes (Table S1). As expected, the strongest RNAP binding was observed for genes involved in ribosome, flagella and ATP synthesis. Consultation of transcriptomic data for LT2 grown to exponential phase [2] revealed that RNAP-bound genes were expressed at high levels, and RNAP binding was only associated with genes that were transcribed (Figure 2).

**Figure 3:**
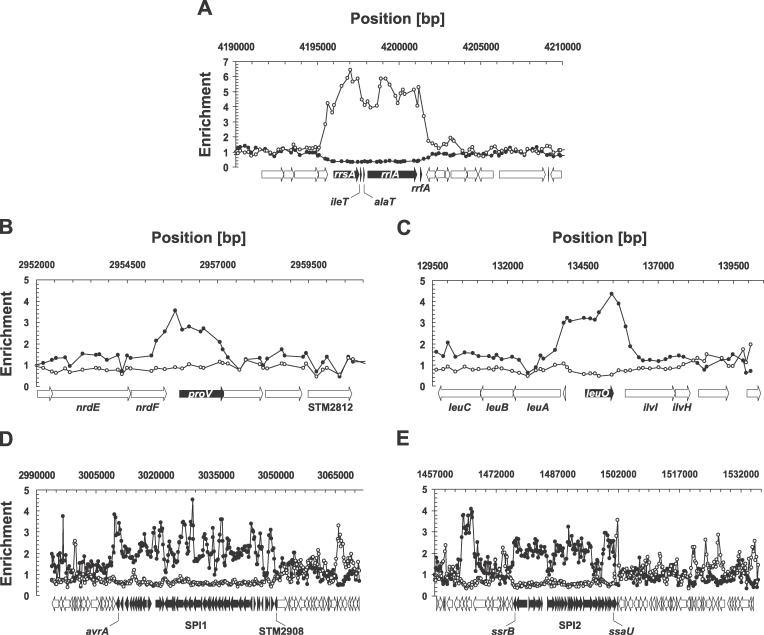
Distribution of H-NS and RNAP over Segments of the S. typhimurium Genome (A–C) Graphs showing H-NS (filled circle) and RNAP binding (open circle) over the *rrnA* locus, *proU* operon, and *leuO* gene. (D and E) H-NS and RNA polymerase binding over the *Salmonella* pathogenicity islands 1 and 2 (SPI1, SPI2). Genes within SPI1 or SPI2 are represented by black arrows. The boundaries of SPI1 and SPI2 were defined from the Horizontal Gene Transfer Database http://www.tinet.org/~debb/HGT [25].

The ChIP-on-chip experiments using anti-H-NS monoclonal antibodies repeatedly displayed high levels of signal across most of the chromosome (unpublished data). We confirmed this observation by repeating the experiment with H200 anti-H-NS polyclonal antibodies, and obtained very similar results (unpublished data). In contrast, the RNAP ChIP-on-chip showed a much lower background signal*.* These findings suggest that the low sequence specificity of H-NS and its high abundance [20] allows the protein to bind at differing levels throughout most of the chromosome. Significantly, the background signal was not homogeneously distributed over the genome; rRNA genes exhibited a low H-NS signal suggesting that H-NS is specifically excluded from highly transcribed genes (Figure 3).

We identified 422 regions that bound H-NS, corresponding to 746 genes, which we suggest contain high-affinity binding sites (Table S1), also known as H-NS nucleation sites [5]. To validate the data, we initially focused on the H-NS binding sites for published H-NS-dependent genes, such as *proV* and *leuO*. ProV is a well characterised H-NS-dependent osmoinducible transport protein in *Salmonella* [21]. H-NS has been shown to repress *proV* by binding to a DNA-region, the downstream regulatory element, which is located within the *proV* structural gene [7]. H-NS also transcriptionally represses *leuO,* a gene coding for a pleiotropic regulator of gene expression, by binding to a curved AT-rich DNA region up-stream of the *leuO* promoter [12]. Our data confirm the reported binding patterns (Figure 3). To add biological relevance to our ChIP-on-chip data we correlated our results with the transcriptomic analysis of strain LT2 and its congenic *hns*-deletion mutant JH4000 during late-exponential growth [2]. Genes bound by H-NS are generally expressed at lower than average levels in the wild-type LT2 strain (Figure 2A). A large proportion (254 or 47%) of the 535 genes de-repressed in JH4000 was bound by H-NS in the LT2 strain. In contrast, only 20 (6.1%) of the 330 genes down-regulated in JH4000 were bound by H-NS. These findings demonstrate a role for H-NS as a global gene silencer, and this was confirmed by the observed negative correlation between H-NS binding and gene expression levels (Figure 2C). Expression of about half of the genes that bound H-NS in LT2 were not affected by the deletion of *hns,* probably reflecting the absence of positive regulatory proteins in our culture conditions (exponential growth in LB). We hypothesise that these 422 binding sites are important for the nucleoid structuring role of H-NS.

Of the 1,699 genes that were bound by either H-NS or RNAP in wild-type *S. typhimurium,* only 12 were bound by both proteins. This suggests that repression by H-NS is generally achieved by preventing the RNA polymerase from binding to target promoter sequences, probably via alterations at the level of DNA topology. An alternate model of H-NS repression involves looping and trapping of RNA polymerase at the promoter [22,23]. This model appears to be contradicted by our data, as we do not observe co-localization of H-NS with the RNA polymerase. However, it remains possible that the trapping of RNA polymerase is too transient to be detected by our experimental approach.

**Figure 4:**
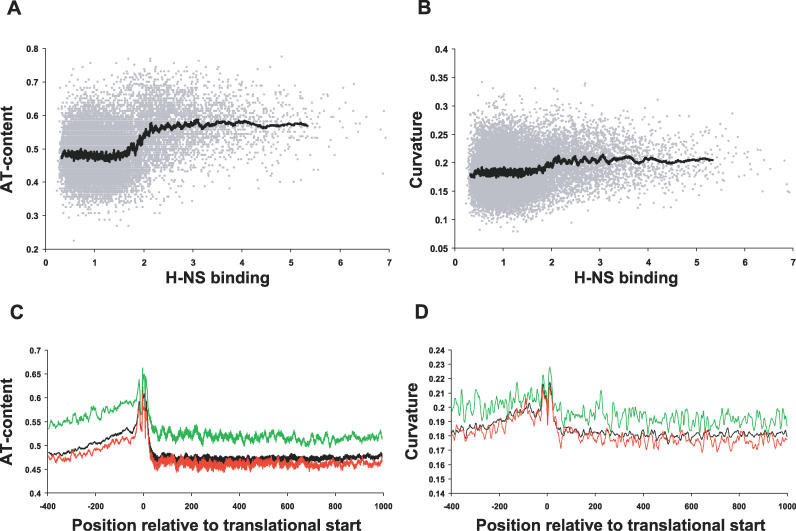
Correlation between AT-Content, Curvature and H-NS, and RNAP Binding to DNA For each oligonucleotide on the microarrays we calculated its average AT-content (A) and curvature (B). To minimize bias that might have been introduced by the algorithms used to design the oligonucleotides, flanking sequences (50 bp) of the oligonucleotides were included in the analysis. The results of each oligonucleotide were plotted against the level of H-NS binding (grey circles). Window-averaged results (window size: 101) are also shown (solid line). (C and D) AT-content and curvature profiles were calculated from putative promoter sequences aligned by over-laying every S. typhimurium gene at the translational start. The profiles have been calculated for all the S. typhimurium genes (black), and for those subsets of genes bound by H-NS (green) or RNAP (red).

No consensus DNA sequence has been identified for H-NS binding, but the protein was reported to have a slightly increased affinity for curved DNA around the regulatory region of genes in vitro [7–9]. We therefore correlated H-NS binding with the base-composition and structural features of DNA such as curvature, stacking energy, and protein-induced deformability. For each oligonucleotide on the microarray, we measured the characteristics of the DNA using the data provided by the Genome Atlas of the S. typhimurium chromosome (http://www.cbs.dtu.dk/services/GenomeAtlas/info/background.php). The best observed correlation was between H-NS binding and AT-content (Figure 4). As expected, analysis of H-NS binding in relation to stacking energy and protein deformability gave very similar profiles to the base composition (unpublished data), as these structural characteristics are largely dependent on AT-content. The correlation between H-NS binding and DNA-curvature was much smaller (Figure 4). Again, we correlated our results with the transcriptomic analysis of strain LT2 and its congenic *hns*-deletion mutant JH4000. Base composition was confirmed to play an important role for H-NS as a repressor of gene expression, because AT-rich genes were 20 times more likely to be repressed by H-NS than GC-rich genes. In contrast, genes with a strong curved DNA sequence in their regulatory regions were only two times more likely to be repressed by H-NS, compared to genes with low or no curvature.

Promoter regions are usually associated with both higher AT-content and curvature of DNA. However, not all bacterial promoters are regulated by H-NS invoking a requirement for binding specificity. We therefore investigated whether genes bound by H-NS displayed a particular topological or DNA sequence profile around their putative regulatory regions. We determined whether the average AT-content and curvature surrounding the translational start for the 746 H-NS-bound genes was different to the profile for all S. typhimurium genes. The genes bound by H-NS displayed higher AT-content and curvature throughout the entire length of their regulatory and structural regions (Figure 4C and 4D). No particular position in the DNA segment displayed a particular type of topology, relative to the translational start. A similar result was obtained for the 254 genes that are both bound by H-NS and de-repressed in JH4000 (unpublished data).

Our data show no requirement for a particularly AT-rich or curved DNA-segment that acts as an H-NS binding site to be positioned at any particular location relative to the promoter region. This corresponds to findings at the *proU* and *hdeA* promoters, showing that the H-NS binding site can be placed at various distances from the transcriptional start without affecting repression by H-NS [9,22]. The results also fit a model where H-NS must bind to regulatory regions at several locations to repress expression.

As genes acquired by *Salmonella* through lateral gene transfer are characterised by their high AT-content [24], we investigated whether H-NS showed increased affinity for islands of foreign DNA. We found a strong preference (*p* <1 × 10^−55^) for H-NS to bind DNA predicted to have recently entered the S. typhimurium genome; H-NS bound 42% of genes that have been acquired by lateral gene transfer [25]. Our data show that H-NS binds to DNA regions that extend throughout entire pathogenicity islands (Figure 3D and 3E). Previous studies have shown that H-NS represses the expression of virulence genes present on pathogenicity islands and plasmids in various Gram-negative bacteria, including *Salmonella, Shigella, E. coli,* and *Vibrio* [2,3,26,27]. Now we have determined that H-NS acts as a selective silencer based on AT-content. However, as H-NS does not bind to all AT-rich genes, cellular factors such as other nucleoid associated proteins might modulate H-NS-binding to particular locations on the genome. Interestingly, our data do not show preferential binding of H-NS to other mobile elements such as prophages. This can be explained by the fact that the four prophages present in the S. typhimurium LT2 genome do not display an AT-content higher than the genome average.

**Figure 5:**
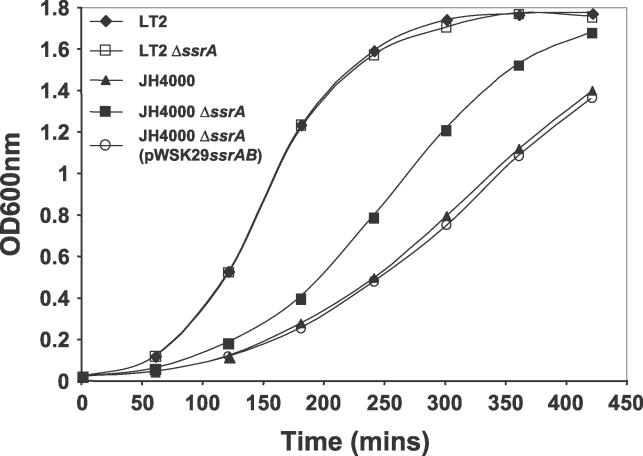
H-NS Confers a Growth Advantage for Wild-Type *Salmonella* by Silencing Genes Acquired through Lateral Gene Transfer The growth of the five S. typhimurium strains were followed in LB, as described in Materials and Methods. The data shown are representative of three experiments, and discussed in the text.

We hypothesised that the binding of H-NS to *Salmonella* pathogenicity islands would have biological significance if it caused silencing of large regions of horizontally-acquired DNA*.* Therefore*,* we compared the fitness of a strain unable to express the *Salmonella* pathogenicity island, SPI2 in both an *hns* ^−^ and *hns* ^+^ background. We discovered that the absence of expression of SPI2 in the strain lacking H-NS substantially improved the growth-rate of *Salmonella* (Figure 5). The S. typhimurium strain JH4000 displays a severely reduced growth-rate caused by the *hns* deletion; the growth rate (μmax) of the LT2 wild-type strain was 1.45, twice as fast as the JH4000 *hns*-deletion mutant (μmax = 0.72). The fitness defect was partially compensated in strain JH4000Δ*ssrA*, which is unable to express genes coded within the SPI2 island; deletion of *ssrA* from JH4000 caused the growth rate to significantly increase (*p* <0.05). In contrast, the absence of SPI2 expression in an *hns* ^+^ background did not affect growth-rate (strain LT2*ssrA*).

It has been reported that a *S. typhimurium hns* mutation was associated with compensatory mutations in the *phoP* and *rpoS* genes [28]. It should be noted that wild-type S. typhimurium strain LT2 already carries a well-characterised point mutation in *rpoS* which modifies the translation initiation codon, causing decreased translation of RpoS [29,30]. To prove that the effect on growth rate was indeed caused by deletion of the *ssrA* gene we ruled out the possibility of secondary mutations by complementing JH4000*ssrA* with plasmid pWSK29*ssrAB*. The plasmid caused a significant decrease in growth rate of JH4000 Δ*ssrA* (*p* <0.05).

Our data reveal that H-NS offers a fitness advantage to wild-type *Salmonella* by silencing the expression of foreign DNA under appropriate conditions. It appears that some pathogenicity islands have acquired the ability to resist H-NS-mediated silencing; the locus of enterocyte effacement (LEE) pathogenicity island of enteropathogenic E. coli codes for Ler, an H-NS homologue that can displace H-NS from the LEE regulatory regions [31].

In summary, we have discovered that global gene silencing occurs in bacteria, and is mediated by H-NS, which acts on at least 254 S. typhimurium genes. Different mechanisms have been proposed to explain the silencing of specific promoters by H-NS. For example, at the *ilvIH*-*leuO*-*leuABCD* gene cluster of *S. typhimurium,* H-NS is initially thought to bind an AT-rich curved DNA sequence which acts as a nucleation site and subsequently forms an extended nucleoprotein complex [12]. We suggest that this type of nucleoprotein complex is responsible for H-NS-mediated gene silencing, and are investigating the co-operative binding of H-NS at other chromosomal regions in *S. typhimurium.* Our observation that H-NS and RNA polymerase rarely co-localize during exponential growth indicates that H-NS generally prevents gene expression by excluding RNA polymerase from binding to DNA, rather than by trapping RNA polymerase at promoters. It is possible that such trapping by H-NS may occur in stationary phase bacteria, when a lower number of RNA polymerase molecules is required for active transcription.

After this manuscript was submitted, a related study was published by Navarre et al [28]. The authors used an analogous approach to detect H-NS binding sites in S. typhimurium strain 14028 and also concluded that H-NS preferentially binds to AT-rich genes. Taken together, these two studies give confidence about the phenomenon of global gene silencing in bacteria; the H-NS protein was found to bind to AT-rich genes in two different S. typhimurium strains (14028 and LT2) grown in two distinct media (minimal medium versus LB).

Taken together, our findings prove that H-NS has a strong preference for AT-rich DNA which facilitates binding to a large number of genes acquired through lateral gene transfer. We know that bacterial evolution relies upon the ability of newly acquired genes to offer a selective advantage to their host [32]. However, the expression of genes encoded by novel DNA must be controlled or the cell will suffer negative consequences. We have shown that H-NS is essential for the prevention of decreased fitness associated with uncontrolled expression of pathogenicity island genes. The evolutionary implications of this silencing function of a nucleoid–associated protein merit further study in many enterobacterial pathogens.

## Materials and Methods

### Bacterial strains and growth conditions.

Salmonella enterica serovar Typhimurium strain LT2 and its *hns* ^−^ derivative JH4000 were described in our recent transcriptomic study [2]. Where necessary, antibiotics were used as appropriate at the following concentrations: ampicillin (100 μg/ml), chloramphenicol (12.5 μg/ml). To measure growth rates, overnight cultures were subcultured 100-fold into 25 ml fresh LB-broth [33] and grown in 250-ml flasks in a 37 °C waterbath (Innova 3100, New Brunswick Scientific, Edison, New Jersey, United States) at 250 rpm.

### Strain construction and DNA manipulation.

The Δ*ssrA* deletion derivative of S. typhimurium LT2 (LT2*ssrA*) was generated using the λ Red method [34]. The oligonucleotides used for this construction were ssrARedF 5′GTGCCAAAGATTTTGCAACAGGCAACTGGAGGGAAGCATTGTGTAGGCTGGAGCTGCTTC-3′ and ssraRedR 5′-ATTCTTTCATTTTGCTGCCCTCGCGAAAATTA AGATAATACATATGAATATCCTCCTTAG-3′, in conjunction with template plasmid pKD4. The Δ*ssrA* deletion mutation was marked with the Kan antibiotic resistance cassette. After mutagenesis, the mutation was P22-transduced to a clean background using phage P22 HT105/1 *int-201*. EBU plates were used to select for non-lysogens [35]. The mutation was verified by PCR using primers external to the mutation. The Δ*hns*Δ*ssrA* double mutant of S. typhimurium LT2 (JH4000*ssrA*) was obtained by transducing the Δ*ssrA* mutation into JH4000.

### Design of a S. typhimurium tiled microarray.

The microarrays used in this study were designed and produced by Oxford Gene Technology (Kidlington, Oxfordshire, United Kingdom). The microarrays consisted of 21,340 60-mer oligonucleotides tiled throughout the S. typhimurium LT2 chromosome and does not cover the pSLT plasmid. The gap between oligonucleotides averaged 170 bp.

### ChIP-on-chip.

Cultures of S. typhimurium LT2 were grown in LB broth with aeration (37 °C, 250 rpm) in an Innova 3100 waterbath (New Brunswick Scientific) until OD_600_ = 0.6 (early-exponential phase), at which point formaldehyde was added to a final concentration of 1% (v/v). After 20 min incubation at 37 °C, the cross-linking reaction was quenched by adding glycine to a 0.5 M final concentration. Cells were harvested and washed twice in TBS. The cell pellet was then resuspended in 1 ml of lysis buffer (10 mM Tris [pH 8.0], 20% [w/v] sucrose, 50 mM NaCl, 10 mM EDTA [pH 8.0], 20 mg/ml lysozyme, 0.1 mg/ml RNAseA) and incubated for 1 h at 37 °C. Bead beating (3 × 20-s cycles, power 6 [FastPrep FP120, Savant, Farmingdale, New York, United States]) was used to achieve complete lysis. The cell lysate was then added to 4 ml of immunoprecipitation buffer (50 mM HEPES-KOH [pH 7.5], 150 mM NaCl, 1 mM EDTA, 1% [v/v] Triton X-100, 0.1% [w/v] sodium deoxycholate, 0.1% [w/v] SDS, 1 mM PMSF). Three aliquots (800 μl) were sonicated (3 × 20-s cycles at 10 μm amplitude, (MSE Soniprep 150, Sanyo Scientific, Bensenville, Illinois, United States) to solubilize the chromatin. Chromatin immuno-precipitation was then performed as described by Grainger et al [18] on two aliquots, using monoclonal H113 anti-H-NS antibodies [36] or anti-RNA polymerase-β subunit monoclonal antibodies (Neoclone, Madison, Wisconsin, United States). As a negative control, chromatin immuno-precipitation was done on the third aliquot without the addition of any antibodies. Additional control chromatin immuno-precipitations were performed using polyclonal H200 anti-H-NS antibodies [36].

The DNA obtained from chromatin immuno-precipitation was labelled with Cy5, mixed with a reference (Cy3-labelled S. typhimurium LT2 genomic DNA) and hybridized against the S. typhimurium LT2 tiled oligonucleotide microarray. The protocol used to label both types of DNA samples is described at http://www.ifr.bbsrc.ac.uk/safety/microarrays/protocols.html. The hybridization procedure is described by Grainger et al [37]. The experiment was performed with three independent biological replicates.

### Data acquisition and analysis.

The microarray slides were scanned using a GenePix 4000A scanner (Axon Instruments, Union City, California, United States). Spot intensities were quantified using BlueFuse software (BlueGnome, Cambridge, United Kingdom). Data analysis and visualization was performed using GeneSpring GX 7.3 (Agilent, Palo Alto, California, United States). To compensate for unequal dye incorporation and/or differences in background levels caused by the ChIP procedure, data centering was performed by bringing the median Ln(Red/Green) to zero. To determine the level of binding of H-NS or RNA polymerase-β subunit (the enrichment factor), results were normalized to the ratios obtained for the negative control (ChIP-on-chip performed without antibodies).

To identify regions of H-NS and RNAP binding, we only considered oligonucleotides that displayed enrichment factors ≥ 1.5 and 2 for RNAP and H-NS data, respectively, in all three biological replicates. The enrichment factor cut-off of 2 was chosen to reflect the higher variability observed in the H-NS ChIP data. We then retained those signals extending to two or more adjacent oligonucleotides on the genome; the rationale being that since the ChIP procedure generates DNA fragments 500–1,000 bp in length, we did not expect peaks of H-NS and RNAP binding to correspond to isolated oligonucleotides. The complete dataset is available as Supporting Information and at the ArrayExpress database (accession number E-MEXP-806).

## Supporting Information

Table S1 H-NS and RNAP ChIP Data on the S. typhimurium LT2 Chromosome (737 KB PDF)

### Accession Numbers

The NCBI (http://www.ncbi.nlm.nih.gov/entrez) accession number for the LT2 genome sequence is NC003197.

## Abbreviations

ChIP-on-chip: chromatin immunoprecipitation on microarray
H-NS: histone-like nucleoid structuring protein
LB: Luria broth
LEE: locus of enterocyte effacement
RNAP: RNA polymerase
SPI2: Salmonella pathogenicity island 2
