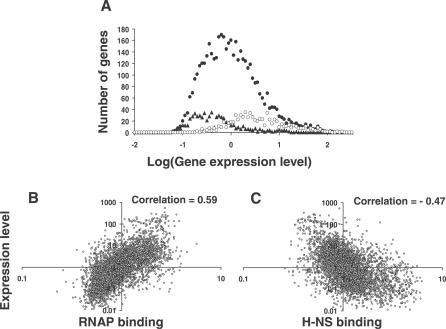# Correction: H-NS Mediates the Silencing of Laterally Acquired Genes in Bacteria 

**DOI:** 10.1371/journal.ppat.0030038

**Published:** 2007-03-30

**Authors:** Sacha Lucchini, Gary Rowley, Martin D Goldberg, Douglas Hurd, Marcus Harrison, Jay C. D Hinton

doi: 10.1371/journal.ppat.0020081

In *PLoS Pathogens*, volume 2, issue 8:

In Figure 2, the graphs for parts B and C were transposed. The correct Figure 2 is as follows:

**Figure:**