# Supplementary material for: H-NS Mediates the Silencing of Laterally Acquired Genes in Bacteria
Source: PLoS Pathog. 2006 Aug 18;2(8):e81. doi: 10.1371/journal.ppat.0020081 (PMC1550270; doi:10.1371/journal.ppat.0020081)
Supplement: Supplementary file 1 [file ppat.0020081.st001.pdf]

Oligonucleotide Systematic Name	Position on Chromosome	Neighbouring gene(s)	H-NS Enrichment Factor	RNAP Enrichment Factor	H-NS Binding site (1=yes, 0=no)	RNAP Binding site (1=yes, 0=no)
12410	3	thrL, thrA	1.530	0.811	0	0
17604	149	thrL, thrA	1.574	0.953	0	0
14029	606	thrA	1.402	0.820	0	0
15551	696	thrA	1.389	0.872	0	0
19756	1139	thrA	0.851	0.731	0	0
1510	1232	thrA	1.290	0.735	0	0
9178	1603	thrA	1.255	0.705	0	0
7787	1695	thrA	0.957	0.656	0	0
1587	1953	thrA	1.380	0.834	0	0
12327	2237	thrA	1.130	0.869	0	0
18772	2529	thrA, thrB	0.864	0.820	0	0
19194	2759	thrA, thrB	1.310	1.185	0	0
2468	2980	thrB	1.449	1.373	0	0
3751	3214	thrB	1.358	1.178	0	0
10893	3299	thrB, thrC	1.647	1.353	0	0
8892	3646	thrB, thrC	1.584	1.236	0	0
1030	3724	thrC	1.890	1.412	0	0
16057	3831	thrC	1.676	1.506	0	0
18580	4320	thrC	1.249	1.261	0	0
15712	4509	thrC	1.209	1.471	0	0
17016	4756	thrC	1.266	2.278	0	1
1268	4872	thrC	0.974	2.031	0	1
2999	5121	yaaA	0.946	1.430	0	0
6430	5255	yaaA	1.162	1.561	0	0
3499	5531	yaaA	0.650	0.830	0	0
2679	5841	yaaA	1.157	1.265	0	0
6191	6138	yaaA, yaaJ	1.116	1.007	0	0
6167	6225	yaaA, yaaJ	1.045	0.904	0	0
10579	6356	yaaJ	0.833	0.845	0	0
20860	6564	yaaJ	1.148	1.101	0	0
6037	6678	yaaJ	1.583	1.195	0	0
8213	7241	yaaJ, talB	1.136	1.319	0	0

17550	7433	yaaJ, talB	0.996	1.758	0	1
21946	7630	yaaJ, talB	0.979	2.684	0	1
18418	7989	talB	0.796	2.107	0	1
8648	8193	talB	0.908	2.419	0	1
6005	8272	talB, mog	0.916	2.042	0	1
8879	8650	mog	0.531	1.226	0	0
1138	8872	mog	0.903	1.221	0	0
17722	9108	mog	1.131	1.267	0	0
2729	9293		1.362	1.127	0	0
5665	9391	yaaH	1.261	1.082	0	0
13937	9721	yaaH	1.666	0.858	0	0
2058	9937	yaaH	1.933	0.996	0	0
4067	10011	yaaH	1.161	0.626	0	0
7881	10417	htgA	1.175	0.718	0	0
7562	10631	htgA	1.298	0.724	0	0
2952	10781	htgA	1.296	0.745	0	0
14202	11106	htgA, yaaI	1.391	0.999	0	0
12274	11217	htgA, yaaI	1.245	1.074	0	0
5539	11465	yaaI, dnaK	1.088	1.753	0	0
7961	11688	dnaK	0.651	1.640	0	1
15871	11816	dnaK	0.624	2.031	0	1
3856	12200	dnaK	0.574	2.268	0	1
8038	12470	dnaK	0.534	2.295	0	1
17062	12630	dnaK	0.565	2.501	0	1
16044	12780	dnaK	0.480	2.291	0	1
4717	13083	dnaK	0.871	2.200	0	1
14194	13443	dnaK, dnaJ	0.700	1.841	0	0
3498	13597	dnaJ	0.802	1.922	0	0
536	13721	dnaJ	0.762	2.216	0	0
6435	14090	dnaJ	0.443	0.917	0	0
13559	14344	dnaJ	1.008	1.360	0	0
6088	14594	dnaJ, STM0014	1.683	1.301	0	0
13872	14704	dnaJ, STM0014	1.614	0.991	0	0
1245	14799	STM0014	1.680	0.730	0	0
11013	15212	STM0014	2.443	0.605	1	0
11214	15283	STM0014	2.677	0.533	1	0

5127	15567	STM0014	2.139	0.564	0	0
248	15721	STM0014, STM0015	2.189	0.586	0	0
4870	16068	STM0015	2.720	0.512	1	0
12611	16177	STM0015	2.909	0.629	1	0
12066	16495	STM0016	1.997	0.491	0	0
17615	16806	STM0016	2.385	0.667	1	0
2014	17023	STM0016, STM0017	2.725	0.533	1	0
18038	17299	STM0016, STM0017	3.583	0.487	1	0
688	17373	STM0016, STM0017	2.778	0.526	1	0
2463	17486	STM0017, STM0018	2.381	0.436	1	0
10339	17833	STM0017, STM0018	2.926	0.533	1	0
10657	18137	STM0018	2.244	0.637	0	0
20741	18280	STM0018	1.459	0.640	0	0
12338	18520	STM0018	1.456	0.691	0	0
21161	18694	STM0018	1.245	0.809	0	0
2396	19054	STM0018	1.250	0.673	0	0
6008	19322	STM0018	2.188	0.781	0	0
242	19554	STM0018	1.617	0.761	0	0
14404	19812	STM0018, STM0019	1.283	0.732	0	0
9035	19940	STM0019	1.552	0.671	0	0
12498	20078	STM0019	1.504	0.666	0	0
10327	20237	STM0019	1.453	0.649	0	0
9567	20533	STM0019	1.632	0.749	0	0
1840	20619	STM0019	1.070	0.620	0	0
10025	21024	STM0019	1.658	0.815	0	0
15417	21324	STM0019	2.037	0.809	0	0
5371	21594	STM0019	1.905	0.777	0	0
18125	21797	STM0019	2.028	0.663	0	0
12830	22051	STM0019	1.483	0.636	0	0
5403	22299	STM0019	1.666	0.664	0	0
4662	22527	STM0019	1.852	0.696	0	0
13910	22661	STM0019	2.544	0.776	1	0
17667	22935	STM0019, STM0020	3.425	0.653	1	0
1922	23045	STM0020	3.624	0.609	1	0
325	23297	STM0020	3.073	0.509	1	0
18066	23487	STM0020	3.259	0.491	1	0

4190	23852	STM0020	2.772	0.571	1	0
13730	24009	STM0020, bcfA	3.875	0.554	1	0
13870	24301	bcfA	2.707	0.483	1	0
7275	24449	bcfA	3.252	0.548	1	0
10488	24685	bcfA, bcfB	2.663	0.602	1	0
14120	24866	bcfA, bcfB	2.290	0.607	0	0
2447	25209	bcfB	2.961	0.667	1	0
6946	25446	bcfB, bcfC	3.349	0.719	1	0
8674	25600	bcfB, bcfC	1.854	0.598	0	0
17335	25805	bcfC	2.031	0.717	0	0
18874	25996	bcfC	2.014	0.758	0	0
14427	26313	bcfC	3.985	0.690	0	0
12287	26535	bcfC	1.458	0.657	0	0
20948	26823	bcfC	1.604	0.881	0	0
1107	26946	bcfC	0.903	0.713	0	0
3058	27209	bcfC	1.540	0.777	0	0
20677	27440	bcfC	0.951	0.572	0	0
7365	27721	bcfC	1.416	0.604	0	0
18485	27826	bcfC	1.431	0.585	0	0
4560	28074	bcfC, bcfD	1.434	0.666	0	0
15923	28375	bcfC, bcfD	1.794	0.749	0	0
10365	28554	bcfD	1.731	0.738	0	0
18455	28835	bcfD	1.409	0.686	0	0
5561	28967	bcfD, bcfE	1.422	0.786	0	0
527	29116	bcfD, bcfE	1.228	0.768	0	0
18472	29414	bcfE	1.195	0.719	0	0
20611	29781	bcfE, bcfF	1.355	0.698	0	0
17860	29940	bcfE, bcfF	1.718	0.838	0	0
11146	30068	bcfF, bcfG	1.373	0.929	0	0
18678	30332	bcfF, bcfG	0.949	0.739	0	0
9488	30626	bcfG	1.787	0.826	0	0
17753	30862	bcfG, bcfH	1.553	0.811	0	0
1879	30994	bcfG, bcfH	1.435	0.641	0	0
5419	31266	bcfH	1.377	0.743	0	0
9047	31504	bcfH	1.177	0.580	0	0
22036	31662	bcfH	1.674	0.763	0	0

17112	32065	bcfH	2.525	0.588	1	0
7306	32161		3.509	0.512	1	0
17331	32450		2.634	0.514	1	0
19471	32758	STM0029	3.274	0.554	1	0
5380	32984	STM0029, STM0030	2.523	0.455	0	0
3840	33057	STM0029, STM0030	2.188	0.429	0	0
22274	33393	STM0029, STM0030	2.359	0.497	0	0
21487	33621	STM0030	2.337	0.513	0	0
20019	33798	STM0030	2.130	0.483	0	0
5461	33948	STM0030	2.132	0.455	0	0
13086	34187	STM0030	2.811	0.428	1	0
9878	34491	STM0031	2.870	0.598	1	0
8541	34634	STM0031	2.581	0.544	0	0
10631	34873	STM0031, STM0032	2.380	0.452	0	0
9436	35088	STM0031, STM0032	2.122	0.331	0	0
14301	35313	STM0032	2.507	0.464	0	0
3910	35438	STM0032	2.073	0.479	0	0
10004	35818	STM0032	2.442	0.510	1	0
5069	36021	STM0032	2.840	0.491	1	0
10476	36174	STM0032	2.246	0.527	0	0
15899	36411	STM0032	2.204	0.732	0	0
10568	36534	STM0032	1.856	0.609	0	0
15816	36748	STM0032	1.613	0.615	0	0
15610	36891	STM0032	2.000	0.660	0	0
17266	37470	STM0033	1.217	0.678	0	0
13705	37735	STM0033	1.089	0.653	0	0
4396	37813	STM0033	1.129	0.664	0	0
13802	38035	STM0033	1.768	0.619	0	0
17969	38251	STM0033	2.565	0.503	0	0
14926	38602	STM0033	2.737	0.484	0	0
14547	38828	STM0033, STM0034	2.174	0.553	0	0
5936	39139	STM0034	1.970	0.452	0	0
17733	39232	STM0034	2.172	0.561	0	0
1128	39580	STM0034	2.396	0.444	0	0
13478	39751	STM0034, STM0035	3.426	0.309	1	0
11109	39835	STM0034, STM0035	3.394	0.440	1	0

11326	40103	STM0035	4.649	0.515	1	0
20138	40291	STM0035	2.215	0.498	0	0
12114	40570	STM0035	1.328	0.481	0	0
2185	40729	STM0035	1.512	0.706	0	0
717	40833	STM0035	1.616	0.672	0	0
18445	41302	STM0035, STM0036	1.261	0.602	0	0
898	41534	STM0035, STM0036	1.386	0.599	0	0
13401	41690	STM0036	0.957	0.505	0	0
16281	41761	STM0036	1.236	0.541	0	0
17588	42228	STM0036	1.296	0.660	0	0
8749	42393	STM0036	1.364	0.676	0	0
14768	42572	STM0036, STM0037	1.653	0.741	0	0
15402	42851	STM0036, STM0037	1.735	0.577	0	0
9375	42933	STM0037	1.900	0.622	0	0
8585	43213	STM0037	1.792	0.638	0	0
15383	43679	STM0037	2.086	0.629	0	0
892	43762	STM0037	2.322	0.623	0	0
5184	44130	STM0037, STM0038	3.909	0.545	1	0
12795	44281	STM0038	2.920	0.593	1	0
16730	44353	STM0038	2.721	0.597	1	0
10034	44807	STM0038	2.610	0.645	1	0
14228	44928	STM0038	2.983	0.695	1	0
13749	45100	STM0038	2.021	0.647	0	0
11405	45360	STM0038	1.545	0.748	0	0
12921	45616	STM0038	1.259	0.733	0	0
4487	45903	STM0038, nhaA	1.365	1.210	0	0
20939	46146	nhaA	1.243	1.572	0	0
5191	46345	nhaA	1.227	1.505	0	0
12034	46514	nhaA	1.197	1.491	0	0
9570	46670	nhaA	1.237	0.960	0	0
7145	46868	nhaA	1.168	0.682	0	0
15608	47254	nhaA, nhaR	1.122	0.802	0	0
18530	47391	nhaR	1.297	0.741	0	0
19333	47656	nhaR	1.271	0.836	0	0
20159	47840	nhaR	1.403	1.115	0	0
9364	48035	nhaR	1.160	0.987	0	0

6921	48281	nhaR	1.406	1.184	0	0
19281	48573	STM0041	1.278	0.886	0	0
8761	48874	STM0041	1.381	0.802	0	0
19146	49118	STM0041	1.360	0.746	0	0
19302	49205	STM0041	1.532	0.712	0	0
15000	49532	STM0041	1.009	0.586	0	0
15257	49829	STM0041	1.519	0.933	0	0
17217	49924	STM0041	1.193	1.175	0	0
18465	50140	STM0041	0.750	0.743	0	0
9265	50419	STM0041	1.360	0.823	0	0
8272	50667	STM0041, STM0042	1.947	0.723	0	0
16073	50905	STM0042	2.800	0.701	1	0
7136	51138	STM0042	3.473	0.553	1	0
11672	51335	STM0042	4.669	0.583	1	0
4960	51538	STM0042	3.082	0.507	1	0
17656	51793	STM0042	2.518	0.549	1	0
1775	52001	STM0042	2.416	0.743	0	0
11474	52295	rpsT, yaaY	0.914	1.512	0	0
10809	52513	rpsT, yaaY	0.696	2.421	0	1
4996	52585	rpsT, yaaY	0.621	2.746	0	1
21257	52825	rpsT, yaaY	0.668	2.697	0	1
3201	53096	ribF	0.542	1.144	0	0
13468	53411	ribF, ileS	0.728	1.619	0	0
6782	53636	ribF, ileS	0.804	1.795	0	0
15013	53827	ileS	0.735	1.602	0	0
3042	54063	ileS	0.771	1.964	0	1
21044	54363	ileS	0.864	1.840	0	1
13814	54555	ileS	0.572	1.367	0	0
5706	54649	ileS	0.857	1.573	0	0
18377	54939	ileS	0.729	1.418	0	0
2446	55112	ileS	0.933	1.860	0	0
8866	55411	ileS	0.866	2.159	0	1
6614	55662	ileS	0.886	1.923	0	1
17569	55774	ileS	0.767	1.873	0	1
11686	56010	ileS	0.812	1.883	0	1
17937	56370	ileS, lspA	0.825	1.841	0	1

13262	56499	ileS, lspA	0.739	1.731	0	1
4597	56660	lspA	0.740	2.071	0	1
9103	56893	lspA, slpA	0.584	1.702	0	0
20521	57138	lspA, slpA	0.712	2.075	0	0
9953	57473	slpA, lytB	0.765	1.515	0	0
5409	57702	slpA, lytB	0.775	1.444	0	0
14848	57888	lytB	0.911	1.494	0	0
285	58214	lytB	1.551	1.669	0	0
18843	58422	lytB	0.458	0.732	0	0
3065	58679	lytB, STM0050	0.547	0.860	0	0
13139	58847	STM0050	0.889	1.136	0	0
13295	59091	STM0050	0.942	0.882	0	0
21460	59299	STM0050	0.949	1.134	0	0
7534	59412	STM0050	0.946	1.079	0	0
10562	59788	STM0050, rihC	0.881	0.970	0	0
6805	59909	STM0050, rihC	1.049	0.902	0	0
7899	60090	STM0050, rihC	0.983	0.798	0	0
17697	60253	rihC	1.250	0.887	0	0
10090	60541	rihC	0.851	0.773	0	0
468	60720	rihC	0.835	0.693	0	0
4679	61142	STM0052	1.308	1.011	0	0
15296	61328	STM0052	1.512	1.037	0	0
4411	61538	STM0052	1.421	0.773	0	0
1288	61634	STM0052	1.352	0.774	0	0
16291	61994	STM0052, STM0053	1.349	0.860	0	0
8943	62085	STM0052, STM0053	1.570	0.773	0	0
17744	62412	STM0053	1.258	0.670	0	0
13852	62699	STM0053	1.536	0.717	0	0
5984	62879	STM0053	1.380	0.769	0	0
6754	63191	STM0053	2.003	0.538	0	0
11586	63329	STM0053	1.660	0.463	0	0
12162	63435	STM0053	2.052	0.614	0	0
20111	63509	STM0053	1.931	0.848	0	0
20007	63715	STM0053, STM0054	1.770	0.721	0	0
12310	63892	STM0054	1.581	0.740	0	0
20120	64511	STM0054	0.509	0.484	0	0

15455	64653	STM0054	1.315	0.719	0	0
3908	64822	STM0054	1.603	0.681	0	0
19551	65153	STM0054, STM0055	1.825	0.729	0	0
1874	65284	STM0054, STM0055	2.730	0.914	0	0
10408	65512	STM0055	1.050	0.465	0	0
16899	65807	STM0055	2.138	0.764	0	0
15297	66109	STM0055	1.741	0.561	0	0
13718	66262	STM0055	1.977	0.695	0	0
3134	66607	STM0055	2.642	0.738	0	0
16767	66838	STM0055, STM0056	1.788	0.701	0	0
19137	67040	STM0055, STM0056	1.297	0.642	0	0
22114	67114	STM0056, STM0057	1.517	0.741	0	0
7447	67353	STM0057	0.457	0.643	0	0
10714	67729	STM0057	2.745	0.646	1	0
1463	67948	STM0057	3.920	0.591	1	0
14664	68204	STM0057, citC2	4.908	0.551	1	0
7722	68366	STM0057, citC2	3.106	0.493	0	0
11844	68548	STM0057, citC2	3.647	0.453	1	0
11608	68656	STM0057, citC2	5.545	0.657	1	0
13093	68889	citC2	3.623	0.641	1	0
5607	69171	citC2	1.542	0.635	0	0
6603	69314	citC2, citD2	0.967	0.481	0	0
17464	69653	citD2, citE2	0.880	0.508	0	0
12063	69754	citD2, citE2	1.378	0.607	0	0
13742	70167	citE2	1.190	0.610	0	0
7970	70289	citE2	1.539	0.616	0	0
11670	70643	citE2, citF2	1.434	0.630	0	0
12430	70849	citF2	1.591	0.644	0	0
15589	71148	citF2	1.179	0.634	0	0
6431	71312	citF2	1.474	0.674	0	0
4379	71452	citF2	0.845	0.441	0	0
5459	71640	citF2	1.351	0.773	0	0
16295	71880	citF2	1.064	0.766	0	0
14437	71980	citF2, citX2	1.728	0.907	0	0
17536	72362	citF2, citX2	1.381	0.599	0	0
20718	72597	citX2, citG2	1.530	0.688	0	0

3522	72876	citX2, citG2	1.334	0.605	0	0
11622	73058	citG2	1.656	0.668	0	0
15197	73396	citG2	1.648	0.840	0	0
16917	73558	citG2, dapB	0.845	0.719	0	0
10071	73820	dapB	1.042	0.882	0	0
5963	73978	dapB	1.495	0.980	0	0
17466	74192	dapB	1.208	0.981	0	0
22271	74427	dapB	0.935	0.794	0	0
730	74757	dapB, STM0065	1.335	0.891	0	0
3905	74928	STM0065	0.801	0.808	0	0
7669	75119	STM0065	0.745	0.666	0	0
20124	75266	STM0065	0.516	0.687	0	0
12268	75646	carA	0.489	0.646	0	0
16744	75854	carA	0.554	0.729	0	0
353	75996	carA	0.630	0.901	0	0
2103	76164	carA	0.783	0.809	0	0
18782	76311	carA	0.890	0.840	0	0
10920	76416	carA	0.796	0.946	0	0
10381	77018	carB	1.691	1.202	0	0
819	77229	carB	1.051	0.876	0	0
7314	77411	carB	1.448	1.094	0	0
844	77713	carB	1.006	1.103	0	0
1522	77888	carB	1.391	1.405	0	0
9802	78085	carB	1.452	1.117	0	0
15532	78432	carB	1.226	1.012	0	0
14111	78669	carB	1.724	1.222	0	0
1477	78824	carB	1.060	0.962	0	0
11730	79139	carB	1.792	1.424	0	0
11241	79305	carB	1.652	1.375	0	0
8303	79476	carB	1.426	1.165	0	0
12323	79686	carB	0.555	0.389	0	0
7240	79809	carB	1.896	1.316	0	0
3838	80042	carB	2.222	1.033	0	0
17928	80339	caiF	3.516	0.915	1	0
1657	80560	caiF	4.027	0.762	1	0
13215	80690	caiF	3.315	0.682	1	0

19943	81129	caiE	1.222	0.699	0	0
19866	81374	caiE	1.675	0.702	0	0
2170	81577	caiE	1.502	0.785	0	0
2256	81832	caiE, caiD	1.418	0.583	0	0
20116	82027	caiE, caiD	1.664	0.512	0	0
5728	82288	caiD	1.829	0.677	0	0
21271	82368	caiD	4.888	0.632	1	0
8465	82625	caiD, caiC	4.992	0.592	1	0
13200	82861	caiD, caiC	4.202	0.701	1	0
3317	83188	caiC	1.995	0.669	0	0
17249	83298	caiC	1.909	0.804	0	0
17618	83502	caiC	1.218	0.668	0	0
11460	83709	caiC	1.530	0.743	0	0
10059	83982	caiC	1.510	0.662	0	0
16951	84075	caiC	1.446	0.682	0	0
9404	84199	caiC, caiB	1.345	0.576	0	0
21088	84609	caiB	1.343	0.728	0	0
3831	84749	caiB	1.217	0.735	0	0
1305	85102	caiB	1.363	0.753	0	0
18289	85374	caiB	0.910	0.496	0	0
10089	85515	caiB, caiA	0.957	0.636	0	0
17289	85793	caiB, caiA	1.145	0.646	0	0
16968	86054	caiA	1.048	0.638	0	0
15398	86158	caiA	1.128	0.593	0	0
722	86609	caiA	1.314	0.552	0	0
12437	86687	caiA, caiT	1.575	0.662	0	0
4288	86913	caiA, caiT	1.221	0.520	0	0
9212	87041	caiA, caiT	1.576	0.707	0	0
13155	87170	caiT	1.399	0.524	0	0
19977	87612	caiT	1.963	0.746	0	0
13899	87853	caiT	2.599	0.651	1	0
6359	88025	caiT	5.496	0.557	1	0
19498	88306	caiT, fixA	4.755	0.504	1	0
10351	88550	caiT, fixA	4.377	0.439	1	0
9437	88744	fixA	4.933	0.466	1	0
423	89120	fixA, fixB	1.530	0.612	0	0

10883	89212	fixA, fixB	1.324	0.519	0	0
8855	89448	fixB	1.287	0.579	0	0
11906	89548	fixB	1.158	0.588	0	0
21824	89890	fixB	0.916	0.649	0	0
8551	90179	fixB, fixC	1.372	0.686	0	0
6600	90445	fixC	1.222	0.723	0	0
1414	90607	fixC	1.381	0.651	0	0
4893	90732	fixC	1.251	0.585	0	0
13689	91011	fixC	1.657	0.683	0	0
10594	91107	fixC	0.738	0.492	0	0
1918	91546	fixC, fixX	1.413	0.617	0	0
12392	91696	fixC, fixX	1.617	0.620	0	0
3223	91784	fixX, yaaU	1.236	0.631	0	0
5968	92187	yaaU	1.492	0.684	0	0
17277	92424	yaaU	1.807	1.018	0	0
4359	92516	yaaU	2.031	0.999	0	0
7671	92770	yaaU	1.340	0.873	0	0
16131	93036	yaaU	0.845	0.601	0	0
19587	93337	yaaU, STM0080	1.646	0.855	0	0
11600	93511	STM0080	1.966	0.860	0	0
1571	93596	STM0080	1.744	0.724	0	0
17780	94008	STM0081	2.333	0.681	1	0
7111	94112	STM0081	2.555	0.679	1	0
9780	94485	STM0081	1.971	0.615	0	0
20176	94759	STM0082	2.687	0.815	0	0
3559	94935	STM0082, STM0083	1.941	0.589	0	0
5688	95038	STM0082, STM0083	2.632	0.595	1	0
18396	95402	STM0082, STM0083	3.883	0.496	1	0
5297	95658	STM0084	3.058	0.546	1	0
11595	95853	STM0084	2.418	0.495	0	0
21095	95966	STM0084	2.967	0.591	1	0
13950	96229	STM0084	2.372	0.639	1	0
4670	96461	STM0084	2.090	0.558	0	0
3633	96698	STM0084	2.128	0.603	0	0
6556	96974	STM0084	2.093	0.587	0	0
6491	97059	STM0084	2.055	0.532	0	0

15467	97137	STM0084	2.600	0.640	1	0
18056	97539	STM0084	3.054	0.555	1	0
5474	97625	STM0084	2.611	0.536	1	0
13285	97794	STM0084, yabF	2.718	0.641	1	0
4232	98241	yabF	1.732	0.583	0	0
14996	98441	yabF, kefC	1.589	0.628	0	0
21657	98735	yabF, kefC	1.119	0.626	0	0
3539	98933	kefC	0.848	0.600	0	0
4062	99187	kefC	1.364	0.726	0	0
2808	99446	kefC	0.458	0.514	0	0
19154	99747	kefC	0.483	0.499	0	0
19607	99884	kefC	0.433	0.377	0	0
19661	100172	kefC	1.377	0.819	0	0
10934	100419	kefC, folA	1.121	0.850	0	0
8012	100607	kefC, folA	1.226	1.013	0	0
2666	100738	folA	1.304	1.204	0	0
11715	100928	folA	1.041	1.269	0	0
16423	101201	folA	0.506	0.897	0	0
916	101451	apaH	0.890	1.807	0	1
3904	101696	apaH	1.015	1.998	0	1
11273	101781	apaH	1.002	1.705	0	1
9605	102261	apaH, apaG	0.680	1.103	0	0
11407	102456	apaH, apaG	0.529	1.212	0	0
4831	102622	apaH, apaG	0.600	1.366	0	0
768	102760	apaG, ksgA	0.747	1.878	0	1
19810	103079	apaG, ksgA	0.704	1.909	0	1
13398	103367	ksgA	0.833	2.071	0	1
14654	103451	ksgA, pdxA	0.516	1.134	0	0
4220	103873	ksgA, pdxA	0.923	2.242	0	1
10040	104044	pdxA	0.807	2.124	0	1
19094	104166	pdxA	0.752	2.206	0	1
10402	104435	pdxA	0.772	2.727	0	1
4768	104591	pdxA, surA	0.836	2.452	0	1
20292	104873	pdxA, surA	0.846	2.101	0	1
8891	105213	surA	0.560	1.639	0	1
4812	105313	surA	0.603	1.826	0	1

2213	105478	surA	0.639	1.672	0	1
22321	105917	surA, imp	0.643	1.770	0	0
7984	106118	surA, imp	0.658	1.435	0	0
5240	106374	imp	0.783	1.693	0	0
19236	106535	imp	0.704	1.742	0	0
1740	106718	imp	0.632	1.817	0	0
20602	107019	imp	0.704	1.709	0	0
2619	107266	imp	0.707	2.306	0	1
20635	107434	imp	0.846	1.659	0	1
16703	107505	imp	0.858	1.801	0	1
16892	107982	imp, djlA	0.592	2.093	0	1
17423	108137	imp, djlA	0.721	2.243	0	1
617	108353	imp, djlA	0.704	1.641	0	0
4917	108631	djlA	0.983	1.301	0	0
9682	108876	djlA	0.850	0.976	0	0
14071	109068	djlA	0.767	0.977	0	0
4570	109181	djlA	1.246	1.429	0	0
22062	109316	rluA	1.206	1.630	0	0
21341	109447	rluA	1.142	1.597	0	0
18346	109889	rluA	0.857	0.811	0	0
14198	110212	rluA, hepA	1.287	1.006	0	0
13354	110409	rluA, hepA	1.276	0.909	0	0
10122	110535	hepA	1.355	0.922	0	0
13124	110781	hepA	1.004	0.720	0	0
3500	111149	hepA	1.270	0.934	0	0
3702	111379	hepA	1.206	0.830	0	0
14279	111467	hepA	1.399	1.018	0	0
1295	111762	hepA	1.060	0.956	0	0
9006	111856	hepA	1.301	0.955	0	0
7048	112248	hepA	0.703	0.520	0	0
9885	112529	hepA	1.235	1.178	0	0
2783	112650	hepA	0.707	0.850	0	0
1856	112880	hepA	0.927	1.593	0	0
2453	113081	hepA, polB	1.107	1.757	0	0
17859	113433	polB	1.210	1.053	0	0
17039	113536	polB	0.742	0.728	0	0

12739	113900	polB	1.235	0.664	0	0
14335	114130	polB	1.474	0.775	0	0
20747	114368	polB	1.368	0.761	0	0
10628	114478	polB	1.325	0.806	0	0
7701	114735	polB	1.170	0.716	0	0
8478	114925	polB	1.404	0.681	0	0
17923	115072	polB	1.547	0.787	0	0
17908	115425	polB	1.751	0.596	0	0
11587	115510	polB, STM0098	2.464	0.626	1	0
613	115746	polB, STM0098	2.735	0.617	1	0
4710	116070	STM0098, STM0099	3.484	0.571	1	0
9049	116379	STM0098, STM0099	3.208	0.518	1	0
240	116609	STM0100	2.944	0.637	1	0
410	116787	STM0100	3.138	0.668	1	0
10482	116970	STM0100	3.313	0.475	1	0
7354	117152	STM0100	3.411	0.543	1	0
13070	117474	araD	1.885	0.653	0	0
18608	117719	araD	0.643	0.458	0	0
9345	117895	araD	1.176	0.612	0	0
1639	118145	araD, araA	1.504	0.611	0	0
13365	118314	araD, araA	0.841	0.407	0	0
14402	118538	araA	1.584	0.749	0	0
6775	118707	araA	1.255	0.601	0	0
19466	118867	araA	1.625	0.682	0	0
1198	119253	araA	1.232	0.679	0	0
7207	119530	araA	1.193	0.628	0	0
17239	119646	araA, araB	1.316	0.855	0	0
11883	119851	araA, araB	1.213	0.699	0	0
9873	120162	araB	0.830	0.575	0	0
18604	120375	araB	1.751	0.817	0	0
15834	120580	araB	1.825	0.749	0	0
244	120816	araB	1.320	0.857	0	0
22233	120898	araB	1.638	0.891	0	0
21466	121288	araB, araC	1.218	0.803	0	0
3295	121583	araB, araC	0.995	0.877	0	0
1697	121736	araB, araC	1.530	1.235	0	0

6890	121807	araC	1.115	1.031	0	0
21818	122086	araC	1.437	1.680	0	0
10014	122458	yabI	1.181	1.339	0	0
6899	122597	yabI	1.041	1.635	0	0
7393	122845	yabI	0.727	1.026	0	0
21389	122995	yabI	1.290	1.800	0	0
20061	123322	yabI	0.967	1.376	0	0
17370	123409	yabJ	1.408	1.726	0	0
10890	123825	yabJ	1.419	1.161	0	0
20421	124057	yabJ	1.490	1.055	0	0
2309	124349	yabJ, yabK	0.857	0.643	0	0
18248	124503	yabJ, yabK	1.640	1.105	0	0
19259	124818	yabK	1.823	1.281	0	0
15286	124912	yabK	0.659	0.595	0	0
4867	125214	yabK	1.625	1.567	0	0
10125	125285	yabK	1.010	0.950	0	0
16286	125517	yabK	0.665	0.945	0	0
21264	125843	yabK, tbpA	1.418	1.177	0	0
19391	125977	yabK, tbpA	0.821	0.788	0	0
22220	126265	tbpA	1.527	1.256	0	0
15716	126374	tbpA	1.194	1.395	0	0
2625	126662	tbpA	1.918	1.661	0	0
11490	126919	tbpA, yabN	0.890	1.248	0	0
20866	127172	yabN	1.377	1.315	0	0
6625	127473	yabN	1.086	1.265	0	0
16949	127625	yabN	1.127	1.031	0	0
19831	127809	yabN	0.797	0.666	0	0
7575	128197	yabN	1.442	0.843	0	0
20314	128424	yabN	1.728	0.772	0	0
11947	128544	yabN	1.987	0.708	0	0
5235	128787	yabN	0.894	0.713	0	0
14353	129040	leuD	1.684	0.872	0	0
16549	129180	leuD	1.544	0.906	0	0
4134	129454	leuD	1.443	0.741	0	0
3117	129633	leuD	1.610	0.753	0	0
15386	129856	leuD, leuC	1.398	0.779	0	0

15919	130016	leuD, leuC	2.043	0.918	0	0
3091	130266	leuC	1.364	0.681	0	0
19401	130568	leuC	1.591	0.919	0	0
14130	130849	leuC	1.539	0.765	0	0
348	131040	leuC	1.394	0.794	0	0
12645	131376	leuC, leuB	1.408	0.806	0	0
12135	131543	leuB	1.323	0.757	0	0
11869	131641	leuB	1.459	0.866	0	0
6813	131806	leuB	1.269	0.820	0	0
11831	132154	leuB, leuA	1.374	0.690	0	0
6536	132385	leuB, leuA	1.235	0.612	0	0
9160	132668	leuA	0.617	0.482	0	0
20023	132937	leuA	0.883	0.719	0	0
2942	133102	leuA	1.440	0.787	0	0
18370	133184	leuA	1.280	0.737	0	0
1828	133568	leuA	1.698	0.993	0	0
10632	133866	leuA, leuL	2.977	1.043	1	0
12752	133982	leuA, leuL	3.216	0.885	1	0
15830	134084	leuA, leuL	3.064	0.706	1	0
4604	134533	leuO	3.218	0.558	1	0
20547	134741	leuO	3.201	0.591	1	0
393	134901	leuO	3.131	0.579	1	0
6779	135048	leuO	3.476	0.479	1	0
19162	135455	leuO, ilvI	4.362	0.523	1	0
12071	135689	ilvI	3.887	0.719	1	0
9111	135918	ilvI	2.804	0.710	1	0
3415	136073	ilvI	1.859	0.734	0	0
2840	136364	ilvI	1.351	0.825	0	0
2792	136508	ilvI	1.195	0.856	0	0
10393	136730	ilvI	1.226	0.807	0	0
17775	137023	ilvI	1.158	0.868	0	0
11880	137241	ilvI, ilvH	1.256	0.781	0	0
19307	137406	ilvI, ilvH	1.383	0.816	0	0
20271	137695	ilvH	1.425	0.865	0	0
7529	137849	ilvH, fruR	1.365	1.028	0	0
9502	138179	fruR	1.209	1.200	0	0

16113	138299	fruR	1.092	1.308	0	0
3809	138529	fruR	0.756	1.175	0	0
15827	138618	fruR	0.918	1.492	0	0
21481	139120	fruR	1.244	1.312	0	0
18316	139350		1.259	0.937	0	0
12027	139481	yabB	1.493	0.935	0	0
7280	139821	yabB	1.212	1.729	0	0
18760	139916	yabB, yabC	0.619	1.103	0	0
7398	140085	yabB, yabC	0.702	1.947	0	0
953	140390	yabC	0.732	1.955	0	0
18501	140585	yabC	0.653	1.929	0	1
8538	140682	yabC	0.667	1.816	0	1
14900	141111	yabC, ftsL	0.739	2.345	0	1
6405	141323	ftsL, ftsI	0.606	1.888	0	0
7751	141430	ftsL, ftsI	0.582	2.208	0	1
8196	141663	ftsL, ftsI	0.734	2.210	0	1
10496	141738	ftsI	0.564	1.763	0	1
2139	142148	ftsI	0.696	1.506	0	0
7423	142520	ftsI	0.718	2.090	0	0
4481	142759	ftsI	0.629	1.558	0	0
895	142843	ftsI	0.805	1.790	0	0
7271	142916	ftsI	0.718	1.669	0	0
13464	143435	ftsI, murE	0.594	1.323	0	0
6057	143655	murE	0.859	2.685	0	1
5891	143893	murE	0.716	1.967	0	1
1203	144136	murE	0.653	1.923	0	1
19443	144235	murE	0.723	2.234	0	1
19999	144364	murE	0.847	2.204	0	1
9475	144818	murE, murF	0.743	1.908	0	1
3637	144910	murE, murF	0.546	1.400	0	0
3281	145163	murF	0.857	2.148	0	1
6357	145400	murF	0.872	1.919	0	1
6429	145574	murF	0.833	1.944	0	1
295	145909	murF, mraY	1.065	2.440	0	1
14847	146097	murF, mraY	0.762	1.880	0	1
12809	146209	murF, mraY	0.526	1.175	0	0

14255	146556	mraY	0.667	2.322	0	1
5820	146808	mraY	0.726	1.916	0	1
8230	147064	mraY, murD	0.747	2.170	0	1
1760	147211	mraY, murD	0.661	2.115	0	1
11637	147393	murD	0.733	2.032	0	1
17419	147725	murD	0.724	1.836	0	0
15646	147924	murD	0.836	2.339	0	1
3885	148083	murD	0.655	2.111	0	1
6203	148167	murD	0.678	2.092	0	1
8175	148679	murD, ftsW	0.633	1.856	0	1
9368	148814	ftsW	0.707	2.450	0	1
14793	148939	ftsW	0.725	2.512	0	1
2930	149180	ftsW	0.639	1.476	0	0
21783	149410	ftsW	0.807	1.971	0	0
12573	149621	ftsW, murG	0.444	1.048	0	0
9518	149706	ftsW, murG	0.730	2.039	0	1
4050	150109	murG	0.748	2.471	0	1
1168	150316	murG	0.783	2.310	0	1
13690	150637	murG	0.815	2.494	0	1
1331	150775	murG, murC	0.336	0.755	0	0
4760	151121	murC	0.608	1.874	0	1
19627	151238	murC	0.811	2.424	0	1
14639	151339	murC	0.517	1.313	0	0
17621	151745	murC	0.823	2.173	0	1
14638	151875	murC	0.704	1.835	0	1
15728	152178	murC, ddlB	0.781	2.061	0	1
14203	152514	murC, ddlB	0.761	2.213	0	1
10262	152730	ddlB	0.740	2.146	0	1
18732	152808	ddlB	0.615	2.013	0	1
5867	153098	ddlB, ftsQ	0.664	1.719	0	1
7189	153190	ddlB, ftsQ	0.658	2.155	0	1
1624	153373	ddlB, ftsQ	0.648	2.431	0	1
18942	153859	ftsQ	0.681	2.259	0	1
16732	154129	ftsQ, ftsA	0.669	1.651	0	0
21662	154333	ftsQ, ftsA	0.405	1.045	0	0
10495	154487	ftsA	0.517	1.356	0	0

20105	154615	ftsA	0.759	1.681	0	0
18923	154852	ftsA	0.622	1.567	0	0
16326	155040	ftsA	0.673	2.423	0	0
7192	155274	ftsA, ftsZ	0.672	1.883	0	0
11877	155542	ftsA, ftsZ	0.702	1.934	0	0
18008	155649	ftsZ	0.525	1.133	0	0
12554	156040	ftsZ	0.484	1.233	0	0
7924	156288	ftsZ	0.604	2.117	0	1
21407	156609	ftsZ, lpxC	0.503	2.726	0	1
12661	156816	lpxC	0.484	2.987	0	1
12262	156918	lpxC	0.437	3.245	0	1
20608	157096	lpxC	0.363	2.457	0	1
2273	157366	lpxC	0.401	2.557	0	1
16012	157712	lpxC, yacA	0.453	2.214	0	1
926	157912	yacA	0.442	2.161	0	1
2010	158059	yacA	0.459	2.275	0	1
12128	158280	yacA, secA	0.532	2.235	0	1
22235	158665	secA	0.634	1.497	0	0
21528	158767	secA	0.734	1.562	0	0
5498	158927	secA	0.430	0.924	0	0
20363	159110	secA	0.670	1.674	0	0
10073	159403	secA	0.657	1.516	0	0
14673	159659	secA	0.867	1.893	0	1
3918	159869	secA	0.740	1.563	0	1
3040	160038	secA	0.830	1.596	0	0
12679	160439	secA	0.831	1.653	0	0
9068	160661	secA	0.673	1.142	0	0
7648	160809	secA	0.789	1.349	0	0
5896	161130	secA, mutT	0.813	1.464	0	0
6365	161349	secA, mutT	0.817	1.564	0	0
14561	161525	mutT	0.956	1.374	0	0
17756	161731	mutT	0.871	1.607	0	0
12583	161950		0.686	1.371	0	0
13196	162201		0.919	1.794	0	0
3092	162429	yacG	1.000	1.676	0	0
14418	162526	yacG, yacF	0.990	1.437	0	0

4171	162626	yacG, yacF	0.896	1.475	0	0
11083	163162	yacF	0.604	1.282	0	0
14820	163449	yacF, yacE	0.638	1.353	0	0
4373	163549	yacF, yacE	0.763	1.881	0	1
12418	163837	yacE, guaC	0.670	1.789	0	1
1963	164021	yacE, guaC	0.567	1.725	0	1
17585	164251	yacE, guaC	0.787	1.757	0	1
17643	164417	guaC	0.858	1.464	0	0
6726	164818	guaC	1.084	1.382	0	0
1779	164916	guaC	1.086	1.143	0	0
1826	165045	guaC	1.147	1.180	0	0
12035	165409	hofC	1.615	0.994	0	0
232	165505	hofC	1.136	0.780	0	0
6032	165701	hofC	1.667	0.767	0	0
19975	166046	hofC	1.186	0.746	0	0
8341	166312	hofC, hofB	1.194	0.668	0	0
7729	166490	hofC, hofB	1.400	0.643	0	0
12024	166717	hofC, hofB	1.189	0.687	0	0
19009	166916	hofB	1.451	0.751	0	0
2985	167131	hofB	1.143	0.652	0	0
7693	167509	hofB	1.049	0.658	0	0
4453	167697	hofB, ppdD	1.589	0.513	0	0
16022	167971	hofB, ppdD	1.084	0.702	0	0
20850	168095	hofB, ppdD	1.200	0.791	0	0
12969	168283	ppdD	1.363	0.861	0	0
12174	168674	nadC	1.446	1.234	0	0
5033	168812	nadC	1.010	1.011	0	0
9363	169112	nadC, ampD	1.237	1.592	0	0
8027	169293	nadC, ampD	1.289	1.527	0	0
18863	169502	nadC, ampD	0.975	1.205	0	0
1115	169669	nadC, ampD	1.040	1.245	0	0
10306	169879	ampD, ampE	0.899	1.036	0	0
2518	170056	ampE	1.030	1.085	0	0
10302	170273	ampE	1.086	1.225	0	0
20435	170484	ampE	1.160	1.263	0	0
22089	170722	ampE	0.559	0.886	0	0

3266	170950	STM0148	0.846	1.398	0	0
4998	171287	STM0148	1.252	0.842	0	0
20480	171389	STM0148	0.772	0.582	0	0
2096	171836	STM0148, STM0149	1.114	0.571	0	0
22299	171907	STM0148, STM0149	1.000	0.761	0	0
426	172299	STM0149	1.164	0.819	0	0
17027	172516	STM0149	1.037	0.789	0	0
18468	172751	STM0149	0.905	0.622	0	0
4350	172848	STM0149	1.219	0.648	0	0
5046	173182	STM0149	0.985	0.622	0	0
18430	173289	STM0149	0.877	0.656	0	0
12933	173364	STM0149, aroP	1.247	0.887	0	0
15614	173883	aroP	0.778	0.706	0	0
20406	174006	aroP	0.926	0.843	0	0
14139	174242	aroP	1.148	0.826	0	0
20298	174562	aroP	0.911	0.832	0	0
7594	174788	aroP	0.658	0.667	0	0
12169	174935	aroP, pdhR	0.800	0.703	0	0
15406	175220	pdhR	0.798	0.753	0	0
14643	175339	pdhR	0.731	0.735	0	0
10142	175554	pdhR	0.763	0.964	0	0
20976	175905	pdhR, aceE	1.082	1.186	0	0
8980	176065	aceE	1.092	0.948	0	0
20003	176157	aceE	0.900	0.680	0	0
11494	176317	aceE	1.267	1.066	0	0
13370	176815	aceE	0.603	0.500	0	0
13804	176976	aceE	1.111	1.057	0	0
9480	177058	aceE	0.893	0.746	0	0
19501	177529	aceE	1.084	0.950	0	0
6540	177741	aceE	0.965	1.091	0	0
21014	177895	aceE	1.070	1.110	0	0
1884	177987	aceE	0.806	0.954	0	0
20165	178366	aceE	1.104	1.196	0	0
18129	178600	aceE, aceF	0.959	0.868	0	0
20809	178894	aceF	1.184	1.034	0	0
4829	179078	aceF	0.953	0.824	0	0

18324	179238	aceF	0.945	0.903	0	0
17064	179394	aceF	0.920	1.008	0	0
15244	179565	aceF	1.169	0.982	0	0
14792	179713	aceF	1.161	0.942	0	0
12157	180092	aceF	0.884	0.860	0	0
5020	180442	aceF	0.775	0.843	0	0
6277	180672	aceF, lpdA	0.729	1.222	0	0
16907	180845	lpdA	0.633	1.337	0	0
10622	180989	lpdA	0.602	1.784	0	0
16522	181240	lpdA	0.720	1.984	0	1
3060	181555	lpdA	0.689	1.745	0	1
18777	181644	lpdA	0.732	1.517	0	0
5198	182016	lpdA	0.745	1.886	0	1
10706	182164	lpdA	0.799	1.696	0	1
22195	182494	STM0155	1.113	1.029	0	0
18947	182592	STM0155	0.889	0.800	0	0
14617	182709	STM0155	1.096	0.851	0	0
9048	183075	STM0156	1.179	0.796	0	0
13670	183409	STM0156	1.203	0.852	0	0
21112	183548	STM0156	1.091	0.988	0	0
13585	183780	STM0156	1.367	0.817	0	0
20384	183968	STM0156, yacH	1.160	0.779	0	0
15322	184174	STM0156, yacH	1.036	0.766	0	0
19840	184511	yacH	1.311	0.884	0	0
17813	184594	yacH	0.428	0.373	0	0
12003	184665	yacH	0.658	0.694	0	0
19229	185015	yacH	1.110	0.866	0	0
15681	185439	yacH, acnB	1.069	1.310	0	0
9220	185564	yacH, acnB	0.960	1.699	0	0
6667	185759	yacH, acnB	1.363	2.000	0	1
19300	185988	acnB	0.788	2.098	0	1
16336	186208	acnB	0.485	1.023	0	0
1620	186346	acnB	0.839	1.707	0	0
2422	186736	acnB	0.881	1.875	0	1
17941	186807	acnB	0.802	2.147	0	1
17832	187195	acnB	0.425	0.988	0	0

18747	187521	acnB	0.681	1.283	0	0
6411	187688	acnB	0.830	1.440	0	0
14088	187938	acnB	1.172	1.836	0	0
5973	188124	acnB	1.382	1.291	0	0
4555	188369	acnB	2.910	0.840	1	0
7874	188587	STM0159	2.915	0.514	1	0
3614	188693	STM0159	3.260	0.599	1	0
14080	189008	STM0159	2.689	0.586	0	0
7014	189168	STM0159, yacL	2.642	0.539	1	0
21114	189424	STM0159, yacL	2.611	0.603	1	0
3271	189678	STM0159, yacL	2.251	0.721	1	0
11491	189835	yacL, kdgT	1.407	0.708	0	0
11769	190123	kdgT	1.054	0.786	0	0
19808	190409	kdgT	1.203	0.867	0	0
4325	190599	kdgT	1.213	0.921	0	0
14849	190715	kdgT, STM0162	1.550	0.849	0	0
19251	191120	kdgT, STM0162	1.879	0.827	0	0
7624	191374	STM0162	1.245	0.714	0	0
1322	191586	STM0162	1.118	0.634	0	0
17292	191676	STM0162	1.002	0.660	0	0
14224	192069	STM0162, STM0163	1.605	0.864	0	0
1992	192287	STM0162, STM0163	1.610	0.965	0	0
18683	192371	STM0162, STM0163	1.120	0.794	0	0
8617	192443	STM0163	0.933	0.601	0	0
655	192859	STM0163	1.142	1.051	0	0
402	193208	STM0163, STM0164	1.001	1.423	0	0
13776	193388	STM0164	0.929	1.156	0	0
17803	193679	STM0164	0.927	1.283	0	0
14235	193763	STM0164	1.049	1.645	0	0
4625	193890	STM0164	1.045	1.393	0	0
11213	194360	speD	0.807	1.173	0	0
16868	194500	speD	0.773	0.946	0	0
19320	194825	speD	0.923	0.782	0	0
10769	194908	speD	1.054	0.865	0	0
11127	195039	speD, speE	1.143	0.751	0	0
18052	195387	speD, speE	1.015	1.016	0	0

8491	195547	speE	0.885	1.005	0	0
3646	195779	speE	0.933	1.379	0	0
12311	195958	speE, yacC	0.900	1.500	0	0
20997	196339	yacC, cueO	0.972	1.091	0	0
838	196417	yacC, cueO	0.848	0.939	0	0
18397	196630	yacC, cueO	1.427	0.906	0	0
19228	196763	yacC, cueO	1.526	0.911	0	0
1584	197080	cueO	1.679	0.835	0	0
10176	197334	cueO	1.024	0.635	0	0
7883	197759	cueO	1.343	0.912	0	0
16306	197906	cueO	1.065	0.853	0	0
13538	198083	cueO	1.203	0.949	0	0
9948	198211	gcd	1.052	1.047	0	0
11789	198543	gcd	1.394	0.927	0	0
12749	198663	gcd	1.419	0.915	0	0
4293	198981	gcd	1.963	0.777	0	0
15350	199120	gcd	1.834	0.863	0	0
18518	199536	gcd	1.421	0.777	0	0
7878	199819	gcd	1.334	0.813	0	0
10172	199916	gcd	1.313	0.777	0	0
4329	200020	gcd	1.013	0.755	0	0
17989	200274	gcd	1.041	0.787	0	0
15107	200585	gcd, hpt	1.091	0.946	0	0
845	200812	gcd, hpt	1.047	1.194	0	0
2042	201051	hpt	0.874	1.339	0	0
11140	201235	hpt	0.592	2.068	0	1
7853	201577	yadF	0.615	2.118	0	1
17113	201783	yadF, yadG	0.651	1.956	0	1
14162	202044	yadF, yadG	0.793	1.407	0	0
10268	202297	yadF, yadG	0.934	1.091	0	0
3094	202516	yadG	1.141	0.988	0	0
10614	202708	yadG, yadH	0.876	0.725	0	0
20831	202832	yadG, yadH	1.203	0.782	0	0
22298	203011	yadG, yadH	1.235	0.835	0	0
12122	203261	yadH	1.156	0.814	0	0
11400	203578	yadH	1.277	0.880	0	0

5635	203712	yadH	1.136	0.869	0	0
7583	204138	stiH	1.557	1.285	0	0
15556	204231	stiH	1.155	0.974	0	0
18908	204527	stiH	1.719	0.959	0	0
1555	204664	stiH	1.932	0.870	0	0
6512	204908	stiH	1.586	0.796	0	0
1643	205025	stiH	1.381	0.723	0	0
10722	205400	stiH, stiC	1.594	0.643	0	0
1368	205631	stiC	1.167	0.474	0	0
1153	205715	stiC	1.071	0.535	0	0
3249	205935	stiC	0.990	0.619	0	0
21444	206060	stiC	1.654	0.684	0	0
13021	206160	stiC	1.336	0.630	0	0
3689	206506	stiC	1.244	0.788	0	0
8646	207022	stiC	1.666	0.775	0	0
11694	207290	stiC	1.308	0.599	0	0
7225	207408	stiC	1.378	0.647	0	0
19821	207614	stiC	1.230	0.760	0	0
15570	207923	stiC, stiB	0.685	0.533	0	0
4267	208172	stiB	2.347	0.672	0	0
3771	208299	stiB	2.102	0.651	0	0
1002	208518	stiB, stiA	1.405	0.541	0	0
10790	208736	stiB, stiA	3.305	0.860	0	0
21679	208881	stiA, yadI	1.343	0.506	0	0
3159	209239	stiA, yadI	4.397	0.637	0	0
14947	209518	yadI, yadE	2.331	0.719	0	0
13064	209710	yadE	2.075	0.951	0	0
5601	209790	yadE	1.706	0.934	0	0
360	210170	yadE	1.641	0.957	0	0
10738	210298	yadE	1.558	0.872	0	0
19972	210506	yadE	2.266	0.740	0	0
13313	210634	yadE	2.999	1.054	0	0
15906	210797	yadE	2.295	1.133	0	0
19108	211230	panD	1.137	1.251	0	0
3575	211486	panD, panC	0.915	0.844	0	0
5786	211694	panD, panC	1.190	0.982	0	0

17311	211808	panD, panC	1.301	1.007	0	0
4164	212086	panC	0.995	0.985	0	0
14509	212339	panC	1.118	1.018	0	0
14167	212578	panC, panB	1.285	1.013	0	0
1176	212981	panB	1.370	1.096	0	0
8264	213177	panB	1.124	1.237	0	0
17950	213314	panB	0.940	1.190	0	0
7945	213449	panB, folK	1.229	1.895	0	1
948	213535	panB, folK	1.172	2.194	0	1
13184	214121	folK, pcnB	0.980	2.019	0	1
3528	214342	pcnB	0.683	1.493	0	0
8317	214413	pcnB	0.793	1.296	0	0
8499	214728	pcnB	0.683	1.562	0	0
14898	214886	pcnB	0.818	2.386	0	1
18883	215099	pcnB	0.735	2.407	0	1
20702	215274	pcnB	0.675	2.698	0	1
13751	215422	pcnB, yadB	0.806	3.100	0	1
16379	215699	pcnB, yadB	0.674	1.501	0	0
11091	215772	yadB	0.699	1.357	0	0
5598	216368	yadB, dksA	0.875	2.528	0	1
4551	216517	yadB, dksA	0.619	2.593	0	1
17343	216824	dksA	0.667	2.291	0	1
11136	217017	dksA, sfsA	0.735	1.458	0	0
12214	217286	dksA, sfsA	1.040	1.424	0	0
4571	217515	sfsA	1.086	1.312	0	0
13618	217640	sfsA	1.248	1.448	0	0
2427	217796	sfsA, ligT	1.328	1.675	0	1
16254	218046	sfsA, ligT	1.347	2.097	0	1
6452	218247	ligT, hrpB	2.649	1.846	0	1
11105	218636	ligT, hrpB	1.131	1.068	0	0
3984	218782	hrpB	0.873	0.807	0	0
19007	218994	hrpB	1.200	1.236	0	0
18476	219167	hrpB	1.100	1.069	0	0
705	219403	hrpB	0.811	0.643	0	0
17230	219783	hrpB	1.544	1.479	0	0
7166	219880	hrpB	1.671	1.425	0	0

6472	220087	hrpB	1.058	0.929	0	0
7144	220372	hrpB	1.093	1.331	0	0
13345	220462	hrpB, mrcB	1.020	1.204	0	0
18858	220832	mrcB	1.164	1.473	0	0
8821	221006	mrcB	1.216	1.555	0	0
14973	221098	mrcB	0.434	0.722	0	0
21433	221591	mrcB	1.287	1.619	0	0
16646	221794	mrcB	1.113	1.172	0	0
15835	222025	mrcB	0.611	0.816	0	0
20792	222256	mrcB	1.334	1.293	0	0
13989	222467	mrcB	0.889	1.262	0	0
8363	222605	mrcB	0.973	1.284	0	0
3241	222814	mrcB	0.644	0.754	0	0
9369	223187	mrcB	0.520	0.691	0	0
615	223258	mrcB, fhuA	0.978	1.493	0	0
18766	223546	fhuA	0.784	1.124	0	0
19861	223682	fhuA	0.989	0.954	0	0
12351	223991	fhuA	1.053	0.782	0	0
21807	224314	fhuA	0.975	0.779	0	0
11464	224500	fhuA	0.999	0.713	0	0
17009	224657	fhuA	0.956	0.763	0	0
512	224812	fhuA	1.308	0.813	0	0
11814	225149	fhuA	1.163	0.801	0	0
2046	225225	fhuA	1.300	0.831	0	0
8998	225540	fhuA, fhuC	0.952	0.525	0	0
17124	225626	fhuA, fhuC	1.165	0.752	0	0
13946	225957	fhuC	1.015	0.729	0	0
16438	226221	fhuC	1.467	0.769	0	0
909	226579	fhuC, fhuD	1.719	0.804	0	0
16591	226673	fhuC, fhuD	1.656	0.891	0	0
14199	226997	fhuD	1.654	0.818	0	0
8754	227296	fhuD, fhuB	1.293	0.763	0	0
12544	227454	fhuD, fhuB	1.842	0.929	0	0
10221	227585	fhuD, fhuB	0.922	0.598	0	0
1929	227867	fhuB	1.654	0.961	0	0
19440	228211	fhuB	1.544	0.933	0	0

22347	228447	fhuB	1.560	1.051	0	0
2242	228590	fhuB	1.583	0.789	0	0
502	228727	fhuB	1.509	0.751	0	0
268	229030	fhuB	0.629	0.516	0	0
20840	229253	fhuB	1.650	0.877	0	0
9757	229587	fhuB	2.597	0.747	1	0
5412	229798		3.512	0.557	1	0
14893	229883		3.854	0.653	1	0
9694	230040		3.325	0.490	1	0
21540	230345	stfA	3.501	0.475	1	0
20730	230485	stfA	3.719	0.551	1	0
11217	230861	stfA, stfC	3.488	0.556	1	0
16439	231110	stfA, stfC	3.327	0.543	1	0
13349	231183	stfA, stfC	4.383	0.568	1	0
620	231551	stfC	5.550	0.611	1	0
11154	231662	stfC	5.308	0.550	1	0
8127	231952	stfC	3.409	0.749	1	0
5948	232288	stfC	1.957	0.832	0	0
5521	232486	stfC	0.851	0.651	0	0
11391	232727	stfC	1.353	0.903	0	0
17598	232878	stfC	1.012	0.811	0	0
11325	233206	stfC	1.351	0.635	0	0
17816	233439	stfC	1.001	0.708	0	0
17279	233659	stfC, stfD	0.951	0.610	0	0
16361	233909	stfC, stfD	1.525	0.775	0	0
8820	234076	stfD	1.638	0.704	0	0
18121	234294	stfD, stfE	1.686	0.674	0	0
19195	234404	stfD, stfE	1.045	0.612	0	0
9613	234699	stfD, stfE	1.186	0.545	0	0
19613	234895	stfE, stfF	0.598	0.496	0	0
21103	235000	stfE, stfF	1.439	0.630	0	0
14732	235378	stfF, stfG	1.195	0.550	0	0
14593	235612	stfF, stfG	1.403	0.620	0	0
17251	235704	stfF, stfG	1.156	0.642	0	0
19536	236120	stfG, STM0201	1.396	0.731	0	0
11775	236338	STM0201	1.230	0.719	0	0

961	236523	STM0201	1.006	0.854	0	0
19418	236751	STM0201	0.949	0.804	0	0
798	237069	STM0201	0.563	0.790	0	0
15338	237240	hemL	0.678	0.841	0	0
20477	237312	hemL	1.171	1.309	0	0
17779	237730	hemL	0.646	0.897	0	0
14153	237930	hemL	0.902	0.936	0	0
16638	238035	hemL	0.593	0.754	0	0
9286	238306	hemL, yadQ	0.971	0.981	0	0
4115	238651	hemL, yadQ	0.924	0.916	0	0
6288	238900	hemL, yadQ	1.055	0.798	0	0
5988	239079	yadQ	1.157	0.814	0	0
5682	239297	yadQ	1.535	0.776	0	0
8435	239385	yadQ	1.441	0.838	0	0
17834	239651	yadQ, yadR	0.845	0.873	0	0
20762	239892	yadQ, yadR	0.846	1.420	0	0
12940	240063	yadR	0.951	1.953	0	1
12221	240297	yadR	0.821	2.274	0	1
4246	240413	yadR	0.816	2.394	0	1
4683	240592	yadS	0.928	2.170	0	1
7351	240748	yadS	0.792	2.227	0	1
3874	241356	yadS, btuF	1.116	1.449	0	0
8916	241524	yadS, btuF	1.257	1.159	0	0
18233	241729	btuF	1.051	1.104	0	0
3672	241844	btuF	0.987	1.254	0	0
12096	242153	btuF, pfs	0.835	0.896	0	0
7441	242414	btuF, pfs	0.879	0.864	0	0
1466	242748	pfs, dgt	0.988	0.944	0	0
4090	242820	pfs, dgt	1.299	1.167	0	0
4899	243042	pfs, dgt	0.982	0.577	0	0
19612	243388	dgt	2.070	0.880	0	0
13059	243622	dgt	2.531	0.792	1	0
6668	243894	dgt	2.868	0.761	1	0
13640	244131	dgt, htrA	2.837	0.734	1	0
16495	244321	dgt, htrA	3.103	0.780	1	0
6597	244485	htrA	2.417	0.566	0	0

19015	244627	htrA	1.890	0.754	0	0
6377	244906	htrA	0.805	0.682	0	0
561	245141	htrA	1.086	0.849	0	0
21990	245282	htrA	0.748	0.559	0	0
21479	245574	htrA, cdaR	1.042	0.912	0	0
16267	245878	htrA, cdaR	0.857	0.947	0	0
17982	246013	cdaR	0.920	1.313	0	0
13877	246359	cdaR	0.596	1.031	0	0
2002	246449	cdaR	1.048	1.523	0	0
5490	246661	cdaR	0.917	1.484	0	0
8604	246743	cdaR	1.155	1.612	0	0
6218	247100	cdaR	0.884	2.087	0	0
15726	247457	yaeH, STM0212	0.696	1.670	0	0
18327	247650	yaeH, STM0212	0.949	1.619	0	0
1357	247872	yaeH, STM0212	1.000	0.904	0	0
13746	248158	STM0212	1.149	0.986	0	0
15517	248290	STM0212	0.762	0.813	0	0
14172	248678	STM0212	1.027	1.170	0	0
14506	248785	STM0212	0.482	0.728	0	0
12678	248942	STM0212	0.895	1.582	0	0
1339	249276	dapD	0.939	1.584	0	0
22318	249439	dapD	0.923	1.759	0	0
856	249811	dapD	1.171	1.333	0	0
14463	249885	dapD	0.478	0.585	0	0
1698	250107	dapD	0.914	1.037	0	0
7334	250357	dapD, glnD	0.745	0.741	0	0
18358	250541	dapD, glnD	1.117	0.967	0	0
1073	250775	glnD	1.454	1.075	0	0
3296	251057	glnD	1.093	0.862	0	0
17793	251354	glnD	0.481	0.671	0	0
15255	251428	glnD	0.779	0.828	0	0
2600	251822	glnD	0.949	0.985	0	0
13428	251910	glnD	0.915	0.829	0	0
5239	252233	glnD	1.157	0.962	0	0
14227	252461	glnD	1.200	1.204	0	0
991	252594	glnD	1.175	1.581	0	0

1373	252784	glnD	1.200	1.687	0	0
9632	253100	glnD, map	0.751	1.562	0	0
2247	253192	glnD, map	0.745	1.749	0	0
15916	253541	map	0.792	1.368	0	0
4002	253802	map, rpsB	0.638	1.486	0	0
20583	253877	map, rpsB	0.586	1.502	0	0
12655	254045	map, rpsB	0.518	2.157	0	0
4737	254452	rpsB	0.391	1.581	0	0
4572	254635	rpsB	0.534	2.429	0	1
15875	254806	rpsB, tsf	0.480	2.194	0	1
11046	255214	tsf	0.496	2.256	0	1
7817	255342	tsf	0.610	2.451	0	1
15343	255576	tsf	0.639	1.629	0	0
18303	255813	tsf, pyrH	0.578	1.647	0	1
13456	256076	tsf, pyrH	0.942	2.044	0	1
3234	256238	pyrH	0.607	1.695	0	1
7885	256347	pyrH	0.715	1.967	0	1
14119	256732	pyrH, frr	0.672	1.540	0	0
9896	257054	frr	0.646	1.452	0	0
8488	257127	frr	0.590	1.421	0	0
12821	257335	frr	0.578	1.689	0	0
9435	257688	dxr	0.557	1.762	0	1
6998	257814	dxr	0.573	1.805	0	1
11409	257998	dxr	0.801	1.976	0	1
12652	258291	dxr	0.777	1.155	0	0
13634	258576	dxr	0.676	0.928	0	0
15473	258892	dxr, uppS	0.864	1.415	0	0
4702	259019	uppS	0.680	1.729	0	1
19814	259291	uppS	0.718	1.793	0	1
10183	259519	uppS	0.838	2.077	0	1
4097	259683	uppS, cdsA	0.648	1.636	0	1
3502	259914	uppS, cdsA	0.535	1.070	0	0
22073	260105	cdsA	0.614	1.599	0	0
18961	260328	cdsA	0.675	1.928	0	1
16701	260466	cdsA	0.607	1.982	0	1
16937	260551	cdsA, yaeL	0.550	1.814	0	0

16787	260977	yaeL	0.596	2.265	0	1
1015	261301	yaeL	0.625	2.077	0	1
18980	261570	yaeL	0.583	2.015	0	1
21608	261727	yaeL	0.584	2.380	0	1
15725	262026	yaeL, yaeT	0.534	1.995	0	1
20327	262286	yaeL, yaeT	0.451	1.785	0	0
4458	262518	yaeT	0.427	1.148	0	0
12599	262747	yaeT	0.555	1.721	0	0
2449	262927	yaeT	0.559	1.798	0	1
526	263087	yaeT	0.720	2.210	0	1
11506	263413	yaeT	0.527	1.581	0	0
6175	263623	yaeT	0.504	1.401	0	0
2177	263760	yaeT	0.381	1.265	0	0
5914	264001	yaeT	0.640	1.737	0	1
1193	264141	yaeT	0.620	1.816	0	1
3077	264421	yaeT, hlpA	0.555	1.415	0	0
1120	264781	hlpA	0.613	1.462	0	0
598	264892	hlpA	0.569	1.556	0	0
10049	265008	hlpA, lpxD	0.588	1.701	0	0
8406	265363	hlpA, lpxD	0.399	0.970	0	0
8350	265518	lpxD	0.475	1.895	0	0
20076	265871	lpxD	0.590	2.219	0	1
12790	266083	lpxD, fabZ	0.557	2.207	0	1
8559	266325	lpxD, fabZ	0.504	2.081	0	1
3147	266521	fabZ, lpxA	0.616	2.089	0	1
2296	266753	fabZ, lpxA	0.683	1.809	0	1
14037	266984	lpxA	0.727	1.954	0	1
14099	267174	lpxA	0.755	1.731	0	1
2322	267328	lpxA, lpxB	0.675	1.605	0	0
3857	267696	lpxA, lpxB	0.789	1.666	0	0
20966	267926	lpxB	0.669	1.418	0	0
977	268054	lpxB	0.833	1.694	0	0
15704	268192	lpxB	0.798	1.171	0	0
5441	268622	lpxB, rnhB	0.754	1.515	0	0
16415	268902	rnhB	0.613	1.050	0	0
13953	269043	rnhB	0.826	1.408	0	0

8917	269143	rnhB, dnaE	0.754	1.393	0	0
18460	269514	dnaE	0.800	1.262	0	0
14580	269813	dnaE	1.025	1.359	0	0
14365	269885	dnaE	0.834	1.108	0	0
21832	270000	dnaE	0.893	1.456	0	0
19619	270138	dnaE	1.238	1.506	0	0
7868	270549	dnaE	0.834	0.815	0	0
10471	270666	dnaE	0.947	1.126	0	0
10685	271131	dnaE	0.826	1.113	0	0
12866	271290	dnaE	0.980	1.371	0	0
6216	271593	dnaE	1.125	1.402	0	0
17418	271769	dnaE	1.194	1.737	0	0
19207	271990	dnaE	1.053	1.534	0	0
9603	272081	dnaE	0.540	0.893	0	0
13851	272420	dnaE	0.674	1.177	0	0
2763	272684	dnaE, accA	0.541	0.918	0	0
11007	272989	dnaE, accA	0.712	2.089	0	0
4700	273060	accA	0.834	2.310	0	1
7548	273373	accA	0.903	2.188	0	1
7640	273677	accA	0.727	1.354	0	0
18941	273909	accA, STM0233	0.940	1.721	0	0
2020	274043	STM0233	1.190	1.132	0	0
14364	274325	STM0233	1.266	0.934	0	0
9247	274427	STM0233	1.838	0.977	0	0
18536	274824	STM0233	1.088	0.838	0	0
6502	274940	STM0233	0.948	0.741	0	0
14682	275022	STM0233	1.062	0.852	0	0
8837	275366	STM0233	1.231	0.827	0	0
15715	275660	STM0233, ldcC	0.794	0.789	0	0
11476	275823	STM0233, ldcC	0.958	0.964	0	0
3509	275960	ldcC	1.011	0.863	0	0
4654	276202	ldcC	1.213	0.933	0	0
13150	276411	ldcC	1.390	0.907	0	0
16179	276651	ldcC	1.212	1.089	0	0
19951	276932	ldcC	1.121	0.973	0	0
13880	277131	ldcC	1.221	0.949	0	0

14992	277322	ldcC	0.993	0.896	0	0
14058	277592	ldcC	1.035	0.981	0	0
9347	277720	ldcC, yaeR	1.064	1.004	0	0
4874	278168	yaeR	1.054	1.124	0	0
19045	278259	yaeR, mesJ	1.013	1.257	0	0
21414	278652	mesJ	0.821	1.354	0	0
8457	278788	mesJ	0.945	1.323	0	0
5830	279138	mesJ	0.524	0.639	0	0
3696	279360	mesJ	0.762	1.177	0	0
8354	279568	mesJ	1.035	1.476	0	0
17401	279760	mesJ	1.136	1.559	0	0
13892	279898	mesJ	0.456	0.805	0	0
1732	280161	rof	0.519	1.443	0	0
14851	280241	rof, yaeQ	0.634	1.295	0	0
17483	280465	rof, yaeP	0.888	1.540	0	0
5063	280540	rof, yaeP	0.804	1.353	0	0
19084	280698	rof, yaeP	0.748	1.063	0	0
7413	281155	yaeQ, yaeJ	1.206	1.257	0	0
4056	281226	yaeJ	1.024	1.277	0	0
5118	281658		1.156	1.487	0	0
18028	281845		1.200	1.483	0	0
5735	282035		0.952	1.500	0	0
924	282486	proS	0.975	1.381	0	0
728	282576	proS	1.000	1.411	0	0
11050	282915	proS	0.800	1.295	0	0
21932	283033	proS	0.772	1.282	0	0
4886	283346	proS	0.861	1.246	0	0
4583	283660	proS	0.928	1.429	0	0
13423	283802	proS	0.932	2.019	0	1
795	284139	proS	0.667	1.828	0	1
11979	284242	proS	0.843	2.169	0	1
9307	284435	proS, yaeB	0.978	2.056	0	1
8167	284624	proS, yaeB	0.761	1.537	0	0
6791	284935	yaeB	1.057	1.799	0	0
4661	285195	yaeB, rcsF	1.071	1.513	0	0
14874	285362	yaeB, rcsF	0.920	0.830	0	0

16195	285478	rcsF	1.003	1.117	0	0
22273	285788	rcsF, yaeC	0.940	0.987	0	0
5842	285949	yaeC	0.941	0.901	0	0
8937	286103	yaeC	0.902	0.850	0	0
6454	286398	yaeC, yaeE	1.093	0.987	0	0
3470	286779	yaeE	1.096	1.167	0	0
3361	286995	yaeE, abc	0.777	0.843	0	0
1704	287128	yaeE, abc	0.800	1.061	0	0
10768	287451	yaeE, abc	0.942	1.061	0	0
12489	287749	abc, yaeD	0.802	0.898	0	0
19159	287867	abc, yaeD	0.872	1.094	0	0
6645	288147	abc, yaeD	0.699	0.901	0	0
13149	288221	abc, yaeD	0.707	1.042	0	0
19334	288507	yaeD	0.629	2.012	0	1
15137	288776	yaeD, rrsH	0.586	3.555	0	1
11707	289030	rrsH	0.347	3.878	0	1
408	289160	rrsH	0.406	4.690	0	1
4908	289587	rrsH	0.401	4.792	0	1
7202	289828	rrsH	0.367	5.307	0	1
7185	289921	rrsH	0.362	5.797	0	1
17996	290132	rrsH	0.344	6.377	0	1
506	290321	rrsH, ileV	0.457	5.540	0	1
1159	290604	rrsH, ileV	0.352	5.352	0	1
1206	290864	alaV, rrlH	0.375	5.164	0	1
20456	290938	alaV, rrlH	0.377	4.375	0	1
15102	291028	alaV, rrlH	0.369	4.413	0	1
6171	291370	rrlH	0.379	4.168	0	1
11734	291777	rrlH	0.408	4.080	0	1
9309	291873	rrlH	0.303	2.861	0	1
550	292030	rrlH	0.375	5.475	0	1
8851	292388	rrlH	0.365	5.300	0	1
8910	292646	rrlH	0.356	5.518	0	1
12939	292882	rrlH	0.339	4.087	0	1
9170	293191	rrlH	0.352	4.743	0	1
6882	293330	rrlH	0.372	5.404	0	1
11735	293475	rrlH	0.356	4.751	0	1

16136	293573	rrlH	0.393	4.585	0	1
20655	294139	rrlH, rrfH	0.367	4.805	0	1
10362	294352	rrfH, aspU	0.410	4.311	0	1
20972	294435	rrfH, aspU	0.388	5.666	0	1
1585	294631	aspU, yafB	0.449	4.474	0	1
12688	294927	yafB	0.495	3.418	0	1
11012	295054	yafB	0.547	2.818	0	1
13699	295252	yafB	0.729	1.881	0	1
21757	295416	yafB	0.743	1.293	0	0
12648	295917	yafC	0.936	0.884	0	0
18369	296081	yafC	1.048	0.896	0	0
14739	296379	yafC	0.968	0.780	0	0
17968	296450	yafC, STM0257	0.932	0.706	0	0
17918	296770	yafC, STM0257	1.232	0.695	0	0
19830	296876	yafC, STM0257	1.221	0.512	0	0
4685	297265	STM0257	1.116	0.584	0	0
6183	297477	STM0257	0.602	0.498	0	0
3953	297617	STM0257	1.134	0.745	0	0
12143	297826	STM0257, yafD	1.158	1.095	0	0
12008	298115	yafD	1.015	1.580	0	0
9717	298189	yafD	1.018	1.743	0	1
18021	298666	yafD, yafE	0.954	1.776	0	1
17036	298901	yafD, yafE	0.863	1.729	0	0
4141	299106	yafE	0.946	1.376	0	0
19408	299324	yafE	1.035	1.346	0	0
20391	299482	yafE	0.908	1.311	0	0
17450	299781	yafE	0.909	1.325	0	0
13553	299904	dniR	0.769	1.175	0	0
8411	300041	dniR	0.736	1.574	0	0
19905	300382	dniR	0.823	1.849	0	0
2799	300577	dniR	1.018	1.484	0	0
13192	300896	dniR	0.622	1.219	0	0
15579	300999	dniR	0.658	1.575	0	0
1977	301339	dniR, gloB	1.084	1.347	0	0
879	301563	dniR, gloB	0.958	0.863	0	0
12370	301660	dniR, gloB	1.079	0.780	0	0

21356	302053	gloB, yafS	1.081	0.880	0	0
14502	302237	gloB, yafS	0.717	0.724	0	0
7884	302524	gloB, yafS	0.831	0.876	0	0
15960	302636	yafS	0.936	1.031	0	0
7063	302987	rnhA, dnaQ	0.736	0.864	0	0
4609	303103	rnhA, dnaQ	0.736	0.783	0	0
20578	303381	rnhA, dnaQ	0.749	0.911	0	0
6309	303679	rnhA, dnaQ	0.548	1.031	0	0
11569	303819	dnaQ, aspV	0.485	1.202	0	0
7625	304122	aspV	0.498	3.262	0	1
17954	304352		0.631	4.679	0	1
12443	304597		0.641	3.898	0	1
16565	304703	STM0266	0.760	3.165	0	1
11488	304826	STM0266	0.666	1.734	0	1
16027	305021	STM0266	1.177	1.527	0	0
946	305393	STM0266	1.315	0.871	0	0
14497	305689	STM0266	1.817	0.839	0	0
16926	305855	STM0266, STM0267	1.576	0.746	0	0
20366	306100	STM0266, STM0267	1.588	0.689	0	0
1772	306379	STM0267	1.984	0.789	0	0
1765	306594	STM0267	0.998	0.593	0	0
7824	306830	STM0267, STM0268	1.266	0.670	0	0
5798	306980	STM0267, STM0268	2.230	0.837	0	0
15985	307253	STM0268	1.972	0.820	0	0
6967	307443	STM0268	1.803	0.757	0	0
14196	307517	STM0268	1.387	0.705	0	0
15313	307769	STM0268	1.820	0.882	0	0
1062	307965	STM0268	1.882	0.796	0	0
20585	308284	STM0268	1.571	0.863	0	0
14272	308566	STM0268	2.227	0.883	0	0
8389	308683	STM0268, STM0269	1.954	0.736	0	0
18383	309015	STM0268, STM0269	1.104	0.667	0	0
1485	309119	STM0269, STM0270	1.559	0.802	0	0
22342	309527	STM0269, STM0270	1.647	0.899	0	0
16462	309633	STM0270	1.616	0.757	0	0
10668	309939	STM0270, STM0271	1.426	0.555	0	0

427	310209	STM0270, STM0271	2.427	0.699	1	0
10601	310387	STM0271	2.590	0.570	1	0
19382	310458	STM0271	2.627	0.624	0	0
4313	310766	STM0271, STM0272	4.433	0.457	1	0
6936	311145	STM0271, STM0272	5.619	0.514	1	0
16227	311342	STM0272	4.427	0.586	1	0
4626	311441	STM0272	4.623	0.614	1	0
15053	311622	STM0272	2.709	0.718	1	0
225	312026	STM0272	2.550	0.658	0	0
18840	312282	STM0272	1.774	0.660	0	0
6902	312363	STM0272	1.684	0.608	0	0
9867	312555	STM0272	1.224	0.593	0	0
1083	312695	STM0272	2.383	0.891	0	0
2801	313116	STM0272	1.246	0.676	0	0
3245	313367	STM0272	1.075	0.755	0	0
2129	313595	STM0272, STM0273	1.132	0.803	0	0
22336	313769	STM0272, STM0273	1.553	0.945	0	0
4594	314097	STM0273, STM0274	1.817	0.684	0	0
21347	314196	STM0273, STM0274	1.644	0.741	0	0
17625	314433	STM0273, STM0274	1.649	0.716	0	0
6173	314810	STM0274	1.273	0.673	0	0
9018	314933	STM0274	1.265	0.633	0	0
2101	315122	STM0274	1.204	0.582	0	0
4542	315286	STM0274	1.751	0.637	0	0
7454	315527	STM0274	1.245	0.482	0	0
2266	315776	STM0274, STM0274A	2.640	0.702	1	0
16312	316108	STM0274A, STM0275	3.864	0.645	1	0
14452	316201	STM0275	3.678	0.524	1	0
10844	316443	STM0275, STM0276	3.397	0.472	1	0
14397	316543	STM0275, STM0276	4.018	0.509	1	0
18009	316757	STM0276	1.916	0.516	0	0
4322	316953	STM0276	2.307	0.505	0	0
958	317424	STM0277	1.327	0.514	0	0
5089	317743	STM0277, STM0278	2.267	0.517	0	0
2868	317986	STM0277, STM0278	2.741	0.430	1	0
9122	318104	STM0277, STM0278	2.860	0.434	1	0

15300	318222	STM0278, STM0279	3.484	0.464	1	0
21825	318438	STM0278, STM0279	3.615	0.405	1	0
1546	318569	STM0279	1.889	0.406	0	0
9235	318950	STM0279, STM0280	2.137	0.525	0	0
15098	319209	STM0280	1.323	0.611	0	0
10907	319380	STM0280, STM0281	1.485	0.568	0	0
579	319761	STM0281	1.775	0.637	0	0
1093	319926	STM0281	1.386	0.548	0	0
12267	320097	STM0281	1.966	0.633	0	0
8831	320337	STM0281	0.903	0.443	0	0
5124	320628	STM0281, STM0282	0.999	0.592	0	0
10401	320804	STM0281, STM0282	1.591	0.665	0	0
8131	321142	STM0282	1.523	0.761	0	0
2706	321369	STM0282	1.464	0.536	0	0
13367	321538	STM0282	2.039	0.633	0	0
16014	321620	STM0282	0.849	0.421	0	0
5861	321904	STM0282	1.297	0.573	0	0
16056	321998	STM0282, STM0283	1.168	0.648	0	0
19457	322505	STM0283	1.638	0.690	0	0
9262	322706	STM0283	2.212	0.536	0	0
11455	322944	STM0283, STM0284	4.185	0.650	1	0
9448	323024	STM0283, STM0284	4.073	0.579	1	0
19745	323378	STM0284, STM0285	2.243	0.536	0	0
9561	323451	STM0284, STM0285	4.638	0.526	1	0
863	323833	STM0285	4.590	0.652	1	0
16230	324043	STM0285	5.058	1.011	1	0
11688	324361	STM0285	1.477	0.590	0	0
17259	324556	STM0285	2.317	0.915	0	0
19669	324825	STM0285	2.136	0.983	0	0
11609	324897	STM0285	0.622	0.633	0	0
20056	325147	STM0285	1.781	0.758	0	0
668	325357	STM0285	0.952	0.536	0	0
13569	325492	STM0285	0.372	0.567	0	0
10693	325862	STM0285	2.358	0.761	1	0
5817	326011	STM0285	2.416	0.690	1	0
17751	326242	STM0285	1.966	0.699	0	0

17412	326451	STM0285	1.459	0.825	0	0
3022	326819	STM0285	1.162	0.715	0	0
14133	326962	STM0285	1.520	0.687	0	0
1380	327298	STM0285, STM0286	2.297	0.894	0	0
12913	327394	STM0285, STM0286	1.279	0.704	0	0
14807	327575	STM0285, STM0286	0.851	0.632	0	0
22133	327985	STM0286	0.774	0.795	0	0
21604	328119	STM0286, STM0287	0.367	0.498	0	0
18306	328447	STM0286, STM0287	1.073	0.808	0	0
1682	328649	STM0287, STM0288	2.051	1.142	0	0
8043	328826	STM0287, STM0288	2.165	0.932	0	0
21986	328923	STM0288	2.250	0.858	0	0
15494	329332	STM0288	3.092	0.602	1	0
6940	329497	STM0289	5.047	0.457	1	0
18805	329808	STM0289	6.271	0.528	1	0
17294	329879	STM0289	4.449	0.529	1	0
20684	330229	STM0289	2.516	0.681	1	0
17247	330459	STM0289	1.385	0.525	0	0
19039	330625	STM0289	1.076	0.580	0	0
3666	330771	STM0289	1.197	0.671	0	0
15706	331071	STM0289	1.313	0.689	0	0
18907	331335	STM0289	1.531	0.743	0	0
17209	331434	STM0289	1.508	0.629	0	0
9772	331746	STM0289, STM0290	1.409	0.600	0	0
12636	332046	STM0290	1.856	0.714	0	0
16983	332130	STM0290, STM0291	1.976	0.749	0	0
11772	332437	STM0290, STM0291	0.827	0.609	0	0
1776	332659	STM0291	1.463	0.718	0	0
7465	332855	STM0291	1.778	0.878	0	0
7713	333010	STM0291	0.616	0.659	0	0
17231	333119	STM0291	1.510	0.639	0	0
12533	333528	STM0291	1.837	0.712	0	0
10539	333753	STM0291	1.656	0.623	0	0
16479	334080	STM0291	1.429	0.826	0	0
19420	334156	STM0291	1.689	0.804	0	0
10636	334571	STM0291	0.461	0.567	0	0

4179	334823	STM0291	1.619	0.827	0	0
5356	334938	STM0291	1.982	0.862	0	0
1494	335214	STM0291	1.509	0.529	0	0
10366	335426	STM0291	1.824	0.826	0	0
2310	335551	STM0291	1.946	0.699	0	0
17435	335880	STM0291	0.850	0.432	0	0
22253	336141	STM0291	1.848	1.055	0	0
14841	336333	STM0291	4.006	1.112	1	0
323	336542	STM0291	4.564	0.886	1	0
15512	336755	STM0292	4.231	0.513	1	0
15688	336828	STM0292	4.408	0.582	1	0
22155	336909	STM0292	3.853	0.671	1	0
2550	337346	STM0292, STM0293	1.585	0.846	0	0
22203	337612	STM0292, STM0293	1.924	0.765	0	0
21181	337743	STM0292, STM0293	2.550	0.834	0	0
7438	337859	STM0293	2.513	0.592	0	0
8554	338353	STM0294	2.718	0.548	1	0
10695	338496	STM0294	2.632	0.580	1	0
5444	338775	STM0294	3.085	0.679	1	0
21323	339134	STM0294	3.006	0.503	1	0
10954	339328		1.880	0.558	0	0
19223	339599	STM0295	2.272	0.690	0	0
4824	339829	STM0295	3.154	0.549	1	0
7563	339934	STM0295	3.066	0.446	1	0
12015	340154	STM0295	2.751	0.473	0	0
11842	340303	STM0295, STM0297	3.082	0.490	1	0
627	340625	STM0296, STM0297	2.496	0.650	1	0
12868	340768	STM0296, STM0297	1.935	0.507	0	0
11821	341139	STM0296, STM0298	1.908	0.544	0	0
18861	341373	STM0298	2.770	0.536	1	0
16710	341520		3.094	0.582	1	0
5214	341684		3.136	0.554	1	0
19773	341945	safA	3.355	0.489	1	0
3085	342288	safA	3.225	0.502	1	0
20983	342438	safA, safB	3.017	0.635	1	0
18057	342558	safA, safB	3.206	0.657	1	0

7999	342980	safB	2.920	0.615	1	0
21611	343063	safB	2.368	0.592	1	0
17595	343432	safB, safC	1.148	0.579	0	0
9829	343509	safB, safC	1.294	0.653	0	0
20870	343734	safC	0.917	0.613	0	0
18575	343925	safC	1.344	0.638	0	0
13758	344230	safC	1.087	0.673	0	0
4991	344303	safC	0.921	0.528	0	0
8442	344608	safC	1.012	0.590	0	0
4592	344907	safC	1.508	1.116	0	0
10879	345040	safC	1.183	1.159	0	0
9730	345287	safC	1.423	1.337	0	0
21279	345651	safC, safD	1.381	0.967	0	0
14284	345918	safC, safD	1.527	0.912	0	0
4882	346067	safD	2.199	0.773	0	0
21591	346276	safD	1.860	0.833	0	0
6753	346568	ybeJ	4.756	0.634	1	0
6018	346768	ybeJ	3.978	0.604	1	0
15030	347001	ybeJ	4.284	0.582	1	0
15196	347119	ybeJ	4.450	0.600	1	0
16568	347475	ybeJ	1.539	0.723	0	0
21506	347565	ybeJ	1.457	0.645	0	0
7657	347783		1.429	0.660	0	0
13408	348124	sinR	1.994	0.620	0	0
1467	348245	sinR	2.420	0.568	0	0
18332	348382	sinR	2.556	0.528	0	0
10454	348514	sinR	2.048	0.418	0	0
6395	348955	sinR	2.451	0.485	0	0
223	349177	sinR, STM0305	2.044	0.618	0	0
20029	349558	STM0305	2.262	0.559	0	0
9978	349649	STM0305	2.202	0.652	0	0
4100	349862		1.429	0.608	0	0
941	350118	STM0306	2.299	0.689	0	0
16144	350350	STM0306	1.902	0.694	0	0
4724	350569	STM0306	2.086	0.602	0	0
3379	350846	STM0306	1.842	0.587	0	0

20194	350957	STM0306, STM0307	2.099	0.637	0	0
17344	351170	STM0307	1.845	0.827	0	0
10578	351305	STM0307	1.722	0.750	0	0
5864	351636	STM0307	0.959	0.737	0	0
17409	351754	STM0307, yafV	1.454	1.066	0	0
10633	352150	yafV	1.201	0.890	0	0
18976	352436	yafV	1.461	0.899	0	0
8834	352511	yafV	1.765	0.889	0	0
15965	352771	yafV, yafH	1.171	0.813	0	0
18965	353144	yafH	1.069	0.668	0	0
1984	353403	yafH	1.590	1.001	0	0
4434	353595	yafH	1.410	0.923	0	0
18039	353794	yafH	1.906	1.117	0	0
2421	353985	yafH	1.721	0.881	0	0
14217	354105	yafH	1.634	1.049	0	0
14605	354518	yafH	1.636	0.868	0	0
10785	354829	yafH, ghmA	0.968	0.719	0	0
19559	354939	yafH, ghmA	1.084	0.809	0	0
8962	355219	yafH, ghmA	1.095	0.791	0	0
9400	355445	yafH, ghmA	1.181	0.867	0	0
5383	355548	ghmA	1.302	0.787	0	0
19572	355792	ghmA, yafJ	1.540	0.938	0	0
9336	355978	yafJ	1.401	0.962	0	0
19805	356358	yafJ	1.391	1.060	0	0
18723	356559	yafJ	1.389	1.290	0	0
13793	356781	yafJ, yafK	1.115	1.227	0	0
21704	356853	yafK	1.070	1.288	0	0
15138	357197	yafK	0.918	1.268	0	0
19240	357388	yafK, dinP	0.880	1.127	0	0
13237	357538	yafK, dinP	0.827	1.146	0	0
18359	357693	yafK, dinP	0.989	1.127	0	0
8739	358140	dinP	1.869	0.854	0	0
6414	358368	dinP	3.407	0.747	1	0
2384	358586	dinP	3.440	0.702	1	0
16836	358833		4.016	0.787	1	0
7764	358919		4.230	0.695	1	0

2251	359090		3.817	0.729	1	0
16602	359161		4.701	0.987	1	0
21551	359521		2.305	0.792	1	0
5686	359597		1.929	0.680	0	0
11824	360229	prfH	1.276	0.892	0	0
15026	360381	prfH	1.398	0.886	0	0
6249	360492	prfH	1.256	1.094	0	0
3714	360873		1.397	1.472	0	0
8324	361066	pepD	1.247	1.508	0	0
7971	361294	pepD	1.053	1.146	0	0
5503	361493	pepD	0.892	1.148	0	0
20058	361825	pepD	1.082	1.178	0	0
4788	362028	pepD	0.916	1.280	0	0
3994	362128	pepD	0.757	1.065	0	0
21163	362472	pepD, gpt	0.721	1.757	0	1
12669	362597	pepD, gpt	0.704	1.832	0	1
5108	362822	pepD, gpt	0.752	2.012	0	1
15334	363038	gpt, yafA	0.770	1.378	0	0
13225	363252	yafA	0.837	1.367	0	0
726	363339	yafA	0.922	1.226	0	0
6460	363895	yafA	1.274	1.081	0	0
4306	363966	yafA	0.836	1.028	0	0
2657	364344	yafA, crl	0.931	1.061	0	0
6462	364519	crl	0.942	1.191	0	0
18591	364708	crl	0.859	1.386	0	0
12517	364812	crl	0.835	1.404	0	0
10470	365004	phoE	0.759	0.948	0	0
14331	365292	phoE	0.978	0.988	0	0
7820	365477	phoE	1.213	0.841	0	0
15637	365856	phoE, proB	1.546	0.934	0	0
4805	366054	phoE, proB	1.515	1.238	0	0
2189	366277	phoE, proB	1.002	1.627	0	0
18973	366559	proB	1.245	1.520	0	0
14039	366691	proB	1.269	1.313	0	0
850	366995	proB, proA	1.192	1.024	0	0
19280	367248	proB, proA	1.253	1.115	0	0

19148	367399	proA	1.288	1.086	0	0
4466	367495	proA	1.475	1.271	0	0
14239	367956	proA	1.138	1.304	0	0
10387	368101	proA	0.847	1.167	0	0
13770	368344	proA, thrW	0.607	1.243	0	0
8813	368419	proA, thrW	0.802	3.049	0	1
2758	368888	STM0324	0.544	2.860	0	1
2609	369003	STM0324	0.626	2.693	0	1
7875	369074	STM0324	0.611	2.313	0	1
16246	369208	STM0324, STM0325	1.152	1.331	0	0
1566	369808	STM0325	1.270	0.726	0	0
8061	369917	STM0325	1.116	0.587	0	0
10115	370139	STM0325	0.854	0.484	0	0
7123	370254	STM0325, STM0327	1.162	0.604	0	0
7301	370689	STM0327	1.414	0.952	0	0
21787	370816	STM0327	1.781	0.908	0	0
16811	371045	STM0328	2.489	0.746	0	0
16301	371352	STM0328	2.794	0.435	0	0
15213	371542	STM0328	3.114	0.623	0	0
20042	371836	STM0328	1.315	0.566	0	0
12313	371998	STM0328	1.778	0.567	0	0
12873	372147	STM0328	1.949	0.551	0	0
599	372316	STM0328, STM0329	2.458	0.532	1	0
18969	372603	STM0328, STM0329	3.590	0.443	1	0
15717	372735	STM0329	4.794	0.506	1	0
12833	372806	STM0329	4.427	0.514	1	0
239	373296	STM0329	1.699	0.661	0	0
6981	373482	STM0329	1.365	0.515	0	0
21026	373775	STM0329, STM0330	1.431	0.638	0	0
22048	373977	STM0329, STM0330	1.220	0.771	0	0
5047	374233	STM0330	1.025	0.524	0	0
18932	374463	STM0330, STM0331	1.080	0.727	0	0
2417	374814	STM0331	1.497	0.652	0	0
15099	374897	STM0331	1.409	0.738	0	0
1177	375175	STM0331	1.424	0.667	0	0
15034	375448	STM0331, STM0332	0.917	0.561	0	0

14135	375590	STM0331, STM0332	1.162	0.692	0	0
16699	375670	STM0331, STM0332	1.068	0.669	0	0
19635	375747	STM0332	1.131	0.685	0	0
13129	376181	STM0332, STM0333	1.284	0.701	0	0
19695	376592	STM0332, STM0333	0.999	0.637	0	0
986	376789	STM0333	1.203	0.665	0	0
3291	376932	STM0333	0.509	0.503	0	0
20517	377040	STM0333	1.395	0.737	0	0
439	377528	STM0333	1.271	0.742	0	0
2790	377756	STM0334	1.681	0.566	0	0
20054	377874	STM0334	2.547	0.543	0	0
6554	378106	STM0334, STM0335	2.656	0.342	0	0
16929	378345	STM0335	2.531	0.490	0	0
11388	378599	STM0335	3.029	0.524	0	0
10477	378903	STM0335	2.526	0.474	0	0
789	379133	stbE	3.265	0.618	1	0
16148	379213	stbE	2.821	0.617	1	0
10299	379592	stbE	1.822	0.660	0	0
8557	379799	stbE, stbD	1.693	0.687	0	0
8285	379882	stbE, stbD	1.460	0.697	0	0
9022	380211	stbD	2.304	0.832	1	0
3804	380401	stbD	2.847	0.747	1	0
14098	380562	stbD	2.369	0.734	1	0
279	380803	stbD	2.083	0.793	1	0
13007	380987	stbD	1.459	0.598	0	0
3817	381175	stbD, stbC	0.857	0.567	0	0
21258	381402	stbD, stbC	1.341	0.738	0	0
3410	381776	stbC	1.351	0.714	0	0
18719	382005	stbC	1.509	0.855	0	0
1788	382229	stbC	1.589	0.883	0	0
7159	382463	stbC	1.803	1.029	0	0
13861	382622	stbC	1.474	0.874	0	0
1536	382880	stbC	1.303	0.728	0	0
3939	383002	stbC	1.673	0.667	0	0
21566	383343	stbC	1.843	0.547	0	0
7424	383459	stbC	2.273	0.561	1	0

20263	383859	stbC, stbB	4.520	0.543	1	0
18983	383949	stbC, stbB	4.803	0.452	1	0
2680	384273	stbB	3.178	0.525	1	0
4509	384576	stbB, stbA	2.953	0.504	1	0
15779	384796	stbA	3.044	0.495	1	0
17720	384883	stbA	2.380	0.491	1	0
2710	385155	stbA, STM0341	2.311	0.502	0	0
13276	385293	stbA, STM0341	2.412	0.453	0	0
1543	385554	STM0341	1.261	0.448	0	0
2564	385817	STM0341, STM0342	2.072	0.513	0	0
12145	385959	STM0341, STM0342	2.557	0.504	0	0
1784	386361	STM0342	2.612	0.453	0	0
14528	386502	STM0342	2.317	0.496	0	0
18029	386784	STM0343	2.157	0.495	0	0
7631	386974	STM0343	1.888	0.432	0	0
297	387154	STM0343	2.010	0.565	0	0
2253	387346	STM0343	1.522	0.550	0	0
2800	387741	STM0343	1.637	0.556	0	0
17650	387881	STM0343	2.197	0.656	0	0
8960	388037	STM0343	2.236	0.612	1	0
13178	388208	STM0343	2.201	0.679	1	0
541	388348	STM0343, STM0344	1.922	0.654	0	0
1763	388856	STM0344	2.229	0.653	0	0
21898	388954	STM0344	2.154	0.573	0	0
7071	389298	STM0344, STM0345	2.218	0.577	0	0
11269	389431	STM0344, STM0345	2.111	0.480	0	0
21806	389616	STM0345	1.934	0.388	0	0
14206	389883	STM0345	2.112	0.544	0	0
18452	390030	STM0346	2.053	0.535	0	0
10458	390420	STM0346	2.021	0.510	0	0
15882	390612	STM0346, STM0347	2.291	0.528	0	0
9980	390734	STM0346, STM0347	2.328	0.560	0	0
20388	390811	STM0346, STM0347	2.123	0.544	0	0
6139	391359	STM0347, STM0348	2.517	0.492	0	0
12247	391528	STM0347, STM0348	2.058	0.490	0	0
2132	391684	STM0348, STM0349	2.125	0.582	0	0

22219	391844	STM0348, STM0349	1.721	0.505	0	0
9798	391995	STM0349	1.359	0.517	0	0
10210	392455	STM0350	1.517	0.695	0	0
10943	392697	STM0350	1.669	0.752	0	0
21435	392988	STM0350	0.565	0.429	0	0
9770	393201	STM0350	0.487	0.320	0	0
5785	393359	STM0350	1.843	0.775	0	0
2181	393632	STM0350	1.904	0.777	0	0
20484	393854	STM0350	1.894	0.734	0	0
8077	393931	STM0350, STM0351	1.066	0.647	0	0
3404	394058	STM0350, STM0351	1.625	1.477	0	0
5368	394429	STM0351	1.578	0.737	0	0
11479	394741	STM0351	1.886	0.675	0	0
22186	394920	STM0351	1.735	0.843	0	0
15072	395031	STM0351	1.042	0.426	0	0
17048	395314	STM0351	1.380	0.711	0	0
5417	395689	STM0351	1.624	0.713	0	0
3899	395829	STM0351	1.212	0.740	0	0
12152	396070	STM0351	1.602	0.892	0	0
11191	396268	STM0351	1.002	0.747	0	0
14539	396592	STM0351	1.687	0.794	0	0
21051	396778	STM0351	1.372	0.603	0	0
3819	396998	STM0351	0.880	0.390	0	0
2612	397232	STM0351, STM0352	1.151	0.675	0	0
9895	397514	STM0352	1.756	0.604	0	0
4563	397593	STM0352	1.831	0.591	0	0
14429	397969	STM0352	1.525	0.564	0	0
16328	398081	STM0352, STM0353	1.586	0.559	0	0
20707	398422	STM0352, STM0353	1.754	0.734	0	0
4053	398506	STM0352, STM0353	1.455	0.632	0	0
16751	398884	STM0353	1.355	0.558	0	0
763	398971	STM0353	1.025	0.537	0	0
21709	399113	STM0353	1.385	0.644	0	0
12412	399592	STM0353	0.984	0.715	0	0
6757	399822	STM0353	1.048	0.552	0	0
12082	399909	STM0353	1.299	0.674	0	0

19317	400110	STM0353	0.686	0.602	0	0
6410	400360	STM0353, STM0354	0.862	0.642	0	0
6066	400612	STM0353, STM0354	1.476	0.816	0	0
21188	400871	STM0354	1.879	1.123	0	0
7918	401021	STM0354, STM0355	1.436	1.008	0	0
659	401370	STM0355	0.827	0.922	0	0
16547	401441	STM0355, STM0356	1.034	0.987	0	0
1551	401647	STM0356	1.057	0.807	0	0
17637	401851	STM0356	0.777	0.758	0	0
15012	401935	STM0356	0.859	0.781	0	0
14575	402114	STM0356	1.105	0.961	0	0
13065	402589	STM0356	0.873	0.677	0	0
15784	402683	STM0356	1.040	0.826	0	0
18542	403212	mod	0.748	0.694	0	0
4082	403323	mod	0.816	0.728	0	0
16570	403648	mod	1.073	0.615	0	0
10200	403813	mod	1.162	0.737	0	0
15601	404058	mod	1.098	0.625	0	0
15370	404137	mod	1.217	0.699	0	0
1314	404430	mod	1.155	0.686	0	0
16673	404645	mod	1.065	0.801	0	0
1241	404845	mod, res	1.070	0.766	0	0
21880	405226	mod, res	1.146	0.708	0	0
8708	405428	res	1.231	0.694	0	0
3478	405605	res	1.199	0.760	0	0
13421	405741	res	1.520	0.759	0	0
6965	406078	res	1.602	0.729	0	0
20526	406373	res	1.548	0.657	0	0
283	406489	res	1.762	0.759	0	0
14307	406838	res	1.381	0.851	0	0
1549	406963	res	1.290	0.848	0	0
1276	407110	res	1.332	0.948	0	0
1865	407314	res	1.277	0.887	0	0
9824	407661	res	1.823	0.775	0	0
9240	407816	res	2.144	0.808	0	0
14726	407928	res	2.120	0.703	0	0

15946	408244	STM0359	2.433	0.775	1	0
11588	408515	STM0359, STM0360	3.105	0.618	1	0
6421	408734	STM0360	2.443	0.526	0	0
18498	408805	STM0360	3.283	0.646	0	0
4520	409075	STM0360	1.985	0.629	0	0
20645	409190	STM0360	1.956	0.700	0	0
18325	409684	STM0360, STM0361	1.210	0.761	0	0
2954	409935	STM0360, STM0361	1.703	0.869	0	0
18675	410012	STM0360, STM0361	1.158	0.668	0	0
3016	410185	STM0361	1.295	0.884	0	0
20161	410257	STM0361	1.729	0.989	0	0
17115	410464	STM0361	1.568	1.130	0	0
3779	411137	STM0361, STM0362	1.564	0.645	0	0
1964	411365	STM0362, STM0363	1.035	0.913	0	0
14828	411467	STM0363	1.342	0.980	0	0
6634	411696	STM0363	1.123	0.959	0	0
7876	411773	STM0363	0.577	0.584	0	0
1680	412082	STM0363	1.388	0.987	0	0
7793	412282	STM0363, foxA	1.110	0.851	0	0
21226	412589	foxA	1.540	0.912	0	0
3510	412928	foxA	1.130	0.724	0	0
21283	413042	foxA	1.090	0.766	0	0
17519	413445	foxA	1.293	0.852	0	0
17440	413516	foxA	1.313	0.733	0	0
15856	413606	foxA	1.371	0.735	0	0
3846	414002	foxA	1.125	0.665	0	0
1693	414241	foxA	1.240	0.765	0	0
21164	414581	foxA	0.718	0.522	0	0
1769	414687	foxA	1.176	0.806	0	0
15699	414805	yahN	1.365	0.915	0	0
8133	415004	yahN	1.505	0.908	0	0
20419	415426	yahN, yahO	1.739	1.033	0	0
7676	415645	yahN, yahO	2.203	0.826	0	0
17644	415717	yahN, yahO	2.513	0.872	0	0
9951	415881	yahN, yahO	1.187	0.492	0	0
16735	416235	prpR	1.105	0.709	0	0

2334	416387	prpR	1.892	1.154	0	0
12515	416665	prpR	1.342	1.057	0	0
18515	416869	prpR	0.538	0.522	0	0
14916	417289	prpR	1.454	1.252	0	0
11440	417421	prpR	1.161	1.132	0	0
20998	417753	prpR, prpB	1.886	1.288	0	0
11866	417912	prpR, prpB	1.644	1.189	0	0
17684	418015	prpR, prpB	1.533	1.229	0	0
9631	418341	prpB	1.061	0.779	0	0
21809	418503	prpB, prpC	0.769	0.697	0	0
12240	418755	prpB, prpC	0.923	0.706	0	0
13030	418945	prpC	0.988	0.725	0	0
6294	419082	prpC	1.389	0.713	0	0
2663	419385	prpC	1.311	0.662	0	0
20539	419757	prpC, prpD	0.814	0.510	0	0
1215	419910	prpC, prpD	1.075	0.576	0	0
2926	420204	prpD	1.050	0.569	0	0
11174	420456	prpD	1.478	0.700	0	0
3468	420609	prpD	1.613	0.745	0	0
5502	420683	prpD	0.832	0.574	0	0
21861	420997	prpD	1.921	0.916	0	0
14112	421140	prpD	0.933	0.696	0	0
8189	421494	prpD, prpE	1.708	0.839	0	0
14771	421680	prpE	1.668	0.865	0	0
5320	421911	prpE	1.343	0.692	0	0
15777	422005	prpE	1.893	0.872	0	0
1027	422509	prpE	1.901	0.736	0	0
18251	422620	prpE	1.775	0.616	0	0
7167	422799	prpE	0.898	0.363	0	0
17362	423169	prpE	1.685	0.818	0	0
5080	423259	prpE	0.704	0.713	0	0
17499	423664	hemB	1.654	1.169	0	0
13744	423762	hemB	1.333	0.903	0	0
386	424047	hemB	1.661	1.271	0	0
16297	424326	hemB	1.498	0.902	0	0
13541	424501	hemB	2.866	0.994	1	0

11387	424661	hemB	3.743	0.892	1	0
10856	424755	hemB, yaiU	3.696	0.581	1	0
9306	425152	yaiU	3.180	0.505	1	0
19929	425343	yaiU	2.878	0.501	1	0
17501	425618	yaiU	2.364	0.507	0	0
9142	425795	yaiU	2.078	0.520	0	0
19573	426097	yaiU	1.768	0.617	0	0
13534	426267	yaiU	1.138	0.593	0	0
18344	426582	yaiU	0.695	0.444	0	0
20317	426779	yaiU	0.712	0.543	0	0
349	426856	yaiU	0.755	0.600	0	0
7571	427054	yaiU	0.901	0.708	0	0
1662	427525	yaiU	0.959	0.821	0	0
1867	427759	yaiU, yaiV	1.276	0.831	0	0
9013	427955	yaiU, yaiV	1.879	0.757	0	0
4363	428200	yaiV	2.646	0.533	0	0
18241	428326	yaiV	2.010	0.620	0	0
6172	428522	yaiV	2.748	0.680	0	0
13290	428773	yaiV	2.190	0.773	0	0
5965	428853	ampH	2.651	0.803	1	0
13052	429097	ampH	2.265	0.900	1	0
610	429177	ampH	1.934	1.089	0	0
11892	429705	ampH	1.087	1.104	0	0
7667	429873	ampH, sbmA	1.023	1.255	0	0
6279	430192	ampH, sbmA	1.036	1.698	0	0
2386	430337	ampH, sbmA	0.755	1.257	0	0
9587	430538	sbmA	1.022	1.319	0	0
931	430739	sbmA	0.660	0.669	0	0
22307	430931	sbmA	1.222	0.952	0	0
17572	431328	sbmA, yaiW	1.166	0.894	0	0
2735	431496	sbmA, yaiW	1.224	0.869	0	0
1449	431713	yaiW	0.645	0.551	0	0
21146	432019	yaiW	1.123	0.803	0	0
6812	432120	yaiW	0.939	0.662	0	0
5434	432379	yaiW	1.262	0.816	0	0
320	432590	yaiW	1.015	0.837	0	0

19293	432928	yaiY, yaiZ	1.218	1.067	0	0
9011	433204	yaiY, yaiZ	1.017	1.257	0	0
12714	433296	yaiY, yaiZ	1.089	1.499	0	0
9976	433659	ddlA	0.562	1.042	0	0
14377	433856	ddlA	0.866	1.359	0	0
18662	434123	ddlA	0.991	1.128	0	0
13771	434322	ddlA, STM0381	1.022	1.062	0	0
15514	434535	ddlA, STM0381	1.100	1.371	0	0
18014	434624	ddlA, STM0381	1.253	1.503	0	0
21667	434798	ddlA, STM0381	1.158	1.559	0	0
16851	435001	ddlA, STM0381	1.036	1.164	0	0
13933	435459	STM0382	1.136	0.643	0	0
15667	435531	STM0382	1.087	0.612	0	0
4968	435709	STM0382	1.220	0.651	0	0
5000	436123	STM0382	1.136	0.622	0	0
12561	436243	STM0382	1.199	0.640	0	0
2578	436575	STM0382	1.905	0.728	0	0
17345	436838	yaiB	2.725	0.729	0	0
5413	437034	yaiB	2.727	0.653	0	0
9041	437122	yaiB	2.854	0.720	1	0
8358	437517	psiF	3.134	0.676	1	0
799	437661	psiF, yaiC	3.437	0.714	1	0
15147	437865	psiF, yaiC	1.354	0.524	0	0
9134	438048	yaiC	0.928	0.580	0	0
504	438132	yaiC	2.296	0.725	0	0
16503	438664	yaiC	1.093	0.831	0	0
21468	438845	yaiC	1.287	1.106	0	0
17202	439135	yaiC	1.323	1.382	0	0
17330	439251	proC	1.189	1.459	0	0
12845	439376	proC	1.053	1.071	0	0
5966	439794	proC, yaiI	0.883	1.067	0	0
19254	439916	proC, yaiI	0.803	1.137	0	0
18170	440037	proC, yaiI	0.623	1.226	0	0
5045	440164	proC, yaiI	1.071	1.686	0	0
10962	440679	aroL	1.350	1.024	0	0
8434	440761	aroL	1.285	0.919	0	0

18310	441087	aroL, yaiA	0.949	1.183	0	0
15631	441359	aroL, yaiA	1.092	1.386	0	0
17521	441593	aroM	1.129	1.218	0	0
16567	441700	aroM	1.081	0.946	0	0
16321	441906	aroM	0.993	1.101	0	0
19378	442083	aroM	1.265	1.095	0	0
1473	442520	yaiE	1.256	1.548	0	0
10688	442612	yaiE	1.330	1.665	0	0
20268	442761	yaiE	0.817	1.441	0	0
16850	442995	rdgC	1.074	2.276	0	0
14780	443418	rdgC	1.182	1.323	0	0
19895	443492	rdgC, yajF	0.832	1.121	0	0
14592	443909	rdgC, yajF	1.208	1.598	0	0
15224	444063	rdgC, yajF	1.135	1.481	0	0
21811	444183	rdgC, yajF	1.243	1.511	0	0
4075	444476	yajF	1.259	1.020	0	0
1825	444658	yajF	1.304	1.213	0	0
17602	444917	araJ	0.971	1.030	0	0
19984	445200	araJ	1.405	1.522	0	0
7183	445353	araJ	0.698	0.869	0	0
7895	445564	araJ	1.374	1.213	0	0
1835	445795	araJ	0.857	0.929	0	0
5645	446025	araJ	1.012	1.060	0	0
17682	446281	araJ, sbcC	1.157	1.206	0	0
14936	446429	araJ, sbcC	1.301	1.043	0	0
14755	446702	sbcC	1.268	1.043	0	0
8539	447002	sbcC	0.766	0.681	0	0
2127	447299	sbcC	0.792	0.718	0	0
13267	447506	sbcC	0.787	0.744	0	0
14309	447668	sbcC	1.057	1.027	0	0
7636	447949	sbcC	1.312	0.937	0	0
19539	448219	sbcC	0.622	0.827	0	0
10313	448326	sbcC	0.555	0.611	0	0
17515	448679	sbcC	1.195	1.043	0	0
22148	448767	sbcC	1.319	1.110	0	0
9573	448973	sbcC	1.364	1.063	0	0

16707	449342	sbcC	1.354	1.138	0	0
15441	449591	sbcC, sbcD	1.092	0.901	0	0
3733	449771	sbcC, sbcD	1.034	1.033	0	0
16520	449875	sbcD	0.873	0.886	0	0
4775	450067	sbcD	0.798	0.835	0	0
16742	450362	sbcD, phoB	1.243	1.196	0	0
10155	450524	sbcD, phoB	0.580	0.786	0	0
17663	450871	sbcD, phoB	0.908	1.764	0	0
22352	450983	sbcD, phoB	0.712	1.600	0	1
11899	451310	phoB, phoR	0.777	1.921	0	1
21619	451496	phoR	0.827	2.184	0	1
7500	451683	phoR	0.709	1.883	0	0
10868	451960	phoR	0.787	1.964	0	0
19659	452296	phoR	0.581	1.146	0	0
12271	452441	phoR	0.732	1.409	0	0
14975	452728	phoR	0.795	1.341	0	0
12086	452822	brnQ	0.784	1.057	0	0
9019	453195	brnQ	0.786	1.146	0	0
21415	453327	brnQ	0.784	1.061	0	0
2592	453634	brnQ	0.641	1.183	0	0
5096	453792	brnQ	1.016	1.498	0	0
11014	453977	brnQ	0.749	1.207	0	0
3119	454225	brnQ, proY	0.741	1.256	0	0
12775	454309	brnQ, proY	1.063	2.098	0	0
7431	454610	proY	0.926	0.700	0	0
6844	454815	proY	0.741	0.930	0	0
8588	455094	proY	0.949	0.951	0	0
21156	455379	proY	1.094	0.960	0	0
13348	455502	proY	1.056	1.106	0	0
14072	455849	proY, malZ	1.026	1.017	0	0
2692	456096	malZ	1.171	0.903	0	0
1790	456265	malZ	1.341	0.919	0	0
15751	456544	malZ	1.373	1.020	0	0
6329	456733	malZ	1.229	0.771	0	0
16772	457063	malZ	1.295	0.851	0	0
2138	457141	malZ	0.956	0.639	0	0

9691	457374	malZ	1.402	1.024	0	0
9696	457499	malZ	1.368	0.964	0	0
15813	457978		1.104	1.260	0	0
16563	458052	STM0402	0.906	1.380	0	0
2461	458396	STM0402	0.814	2.164	0	0
4786	458547	STM0402	0.685	1.760	0	0
9227	458740	STM0402	0.688	2.126	0	1
2173	459021	STM0402, yajB	0.675	1.664	0	1
13241	459313	yajB, queA	1.025	1.536	0	0
12843	459538	yajB, queA	1.134	1.914	0	0
15703	459680	yajB, queA	1.024	1.466	0	0
16533	459932	queA	1.020	1.264	0	0
544	460191	queA	0.946	1.196	0	0
8379	460409	queA, tgt	1.060	1.299	0	0
4025	460497	queA, tgt	1.061	1.271	0	0
10903	460774	tgt	1.008	1.586	0	0
708	461021	tgt	0.795	1.262	0	0
21896	461191	tgt	0.919	1.393	0	0
3396	461388	tgt	0.759	1.091	0	0
10946	461595	tgt, yajC	0.668	1.344	0	0
15538	461856	tgt, yajC	0.763	1.799	0	1
14043	462106	yajC, secD	0.803	1.773	0	1
10119	462471	secD	0.652	1.837	0	1
3669	462734	secD	0.869	1.436	0	0
19750	462819	secD	0.780	1.539	0	0
4281	462999	secD	0.783	1.266	0	0
8555	463389	secD	0.708	1.509	0	0
7272	463496	secD	0.464	1.092	0	0
20052	463710	secD, secF	0.601	1.424	0	0
21244	463978	secD, secF	0.667	1.823	0	1
6272	464192	secF	0.699	1.678	0	1
19678	464506	secF	0.618	1.422	0	0
5396	464811	secF, STM0409	0.686	1.497	0	0
7281	464920	secF, STM0409	0.691	1.585	0	0
4636	465215	STM0409, STM0410	0.805	1.035	0	0
15607	465298	STM0409, STM0410	0.796	1.110	0	0

20776	465621	STM0409, STM0410	0.878	0.841	0	0
18177	465904	STM0410	0.872	0.751	0	0
11056	466142	STM0410, yajD	0.829	0.784	0	0
22311	466342	STM0410, yajD	1.009	1.012	0	0
586	466414	yajD	1.044	1.018	0	0
1497	466747	yajD	0.843	1.149	0	0
9458	466947		0.854	1.196	0	0
1407	467070		0.762	1.211	0	0
7211	467506	STM0412	0.668	1.071	0	0
7913	467737	STM0412, tsx	0.827	1.188	0	0
9958	467976	tsx	0.917	0.820	0	0
14602	468191	tsx	0.924	0.732	0	0
7576	468367	tsx	0.852	0.683	0	0
13554	468596	tsx	1.477	0.723	0	0
18720	468815	tsx, yajI	0.817	0.781	0	0
7866	469097	yajI, ybaD	0.785	0.877	0	0
5442	469220	yajI, ybaD	0.785	1.165	0	0
21130	469389	yajI, ybaD	0.833	1.519	0	0
1129	469611	yajI, ybaD	1.101	2.002	0	1
14845	469742	ybaD, ribD	0.978	1.889	0	1
21550	470219	ribD	1.408	2.760	0	1
12200	470408	ribD	1.005	1.639	0	0
7283	470672	ribD, ribH	0.555	1.301	0	0
20828	470880	ribD, ribH	0.848	2.829	0	1
17258	471008	ribH	0.813	2.827	0	1
10848	471372	ribH, nusB	0.793	2.029	0	1
9521	471480	nusB	0.804	2.024	0	1
20198	471793	nusB, thiL	0.729	1.809	0	0
9735	472037	thiL	0.881	2.164	0	0
4865	472185	thiL	0.743	1.575	0	0
20348	472528	thiL, pgpA	0.804	1.055	0	0
20301	472718	thiL, pgpA	0.644	0.935	0	0
6123	472930	thiL, pgpA	1.061	1.314	0	0
18788	473215	pgpA	0.893	1.164	0	0
19641	473443	pgpA	1.018	1.306	0	0
15388	473648	yajO	1.171	1.564	0	0

12534	473828	yajO	1.433	1.685	0	0
12773	473979	yajO	1.005	1.313	0	0
15485	474156	yajO	1.138	1.515	0	0
14549	474509	yajO	0.819	1.714	0	1
17636	474716	yajO, dxs	1.030	1.690	0	1
22351	474940	yajO, dxs	0.939	1.595	0	0
18904	475081	dxs	0.866	1.510	0	0
14325	475398	dxs	0.863	1.800	0	0
17806	475559	dxs	0.979	1.815	0	0
6381	475778	dxs	0.748	1.485	0	0
13635	475928	dxs	0.870	1.885	0	1
5935	476222	dxs	1.003	2.113	0	1
10889	476458	dxs	0.811	2.968	0	1
11965	476703	dxs, ispA	0.656	2.373	0	1
10560	477059	ispA	0.796	1.698	0	0
9045	477279	ispA	0.821	1.816	0	0
7403	477421	ispA, xseB	0.825	1.610	0	0
12713	477654	ispA, xseB	0.799	1.935	0	0
568	477864	xseB, thiI	0.893	1.987	0	0
2738	478087	xseB, thiI	0.917	1.371	0	0
18114	478315	thiI	0.769	0.926	0	0
19235	478574	thiI	0.775	1.039	0	0
10462	478909	thiI	1.364	1.207	0	0
6686	479136	thiI	1.077	1.127	0	0
12872	479349	phnV	0.898	1.174	0	0
12743	479576	phnV	0.918	0.985	0	0
9947	479828	phnV	1.048	0.837	0	0
7228	479937	phnV	1.258	0.823	0	0
9044	480217	phnV, phnU	1.396	0.813	0	0
15624	480303	phnV, phnU	1.464	0.798	0	0
954	480521	phnV, phnU	1.558	0.821	0	0
4632	480684	phnU	1.452	0.818	0	0
15175	480991	phnU	1.655	0.852	0	0
14027	481363	phnU, phnT	1.742	1.074	0	0
10478	481518	phnT	1.243	0.808	0	0
6820	481793	phnT	1.287	0.746	0	0

2330	481991	phnT	1.259	0.793	0	0
9496	482118	phnT, phnS	1.245	0.837	0	0
8685	482328	phnT, phnS	1.247	0.918	0	0
11033	482677	phnS	1.464	0.810	0	0
1202	482763	phnS	1.319	0.688	0	0
6242	483200	phnS	1.187	0.665	0	0
20379	483348	phnS, phnR	1.095	0.881	0	0
7721	483507	phnS, phnR	0.530	0.614	0	0
12223	483886	phnR, phnW	0.841	0.805	0	0
6784	484094	phnR, phnW	0.914	0.710	0	0
15987	484184	phnR, phnW	0.874	0.784	0	0
9473	484260	phnR, phnW	0.969	0.732	0	0
21333	484731	phnW	1.348	0.638	0	0
14061	484903	phnW, phnX	0.937	0.624	0	0
15989	485102	phnW, phnX	1.254	0.696	0	0
17899	485290	phnX	1.669	0.827	0	0
16293	485581	phnX	1.330	0.787	0	0
13004	485659	phnX	0.897	0.642	0	0
8582	485935	phnX	1.518	1.266	0	0
17059	486308	thiJ	1.143	1.146	0	0
9708	486414	thiJ	1.561	1.294	0	0
6072	486811	thiJ, apbA	1.151	1.185	0	0
21091	486963	thiJ, apbA	1.111	1.229	0	0
10003	487036	thiJ, apbA	1.029	1.106	0	0
13973	487336	apbA, yajQ	1.105	1.143	0	0
14650	487536	apbA, yajQ	1.037	0.935	0	0
3735	487926	apbA, yajQ	0.960	1.381	0	0
3919	488053	apbA, yajQ	1.031	1.471	0	0
21280	488349	yajR	0.863	1.995	0	1
853	488450	yajR	0.882	2.056	0	1
10677	488869	yajR	1.108	1.219	0	0
9387	489018	yajR	1.089	1.089	0	0
21393	489126	yajR	1.243	1.117	0	0
5876	489599	yajR	1.587	1.049	0	0
17795	489697	yajR	0.987	0.797	0	0
18643	489859	yajR, STM0437	1.753	0.652	0	0

10332	490117	yajR, STM0436A	3.167	0.709	1	0
8590	490195	STM0436A, STM0437	3.275	0.678	1	0
12235	490271	STM0436A, STM0437	3.074	0.631	1	0
15433	490741	STM0437	3.324	0.619	1	0
11251	491136	STM0437	2.298	0.559	1	0
11823	491228	STM0437	2.168	0.581	0	0
1350	491486	STM0437	1.980	0.537	0	0
16852	491557	STM0437, STM0438	2.043	0.574	0	0
4779	492004	STM0438	2.799	0.529	0	0
2215	492163	STM0438	2.012	0.529	0	0
12127	492283	STM0438	2.350	0.615	0	0
18875	492596	STM0438	2.386	0.581	0	0
1528	492918	STM0438	2.122	0.660	0	0
1149	493095	STM0438	2.025	0.752	0	0
9726	493361	cyoE	1.249	1.266	0	0
8032	493595	cyoE	0.647	1.709	0	1
1703	493818	cyoE	0.645	1.801	0	1
18871	494019	cyoE	0.522	2.246	0	1
7387	494182	cyoE	0.323	1.523	0	0
17670	494568	cyoE, cyoD	0.537	2.523	0	1
4614	494815	cyoD, cyoC	0.516	2.437	0	1
12373	494940	cyoD, cyoC	0.590	2.034	0	1
13694	495173	cyoC, cyoB	0.514	2.124	0	1
15958	495265	cyoC, cyoB	0.561	2.705	0	1
16858	495409	cyoC, cyoB	0.509	2.592	0	1
21598	495752	cyoB	0.476	3.114	0	1
7728	496038	cyoB	0.544	2.684	0	1
18885	496350	cyoB	0.468	2.385	0	1
17221	496535	cyoB	0.523	3.462	0	1
1252	496799	cyoB	0.497	2.399	0	1
19684	497020	cyoB	0.478	2.499	0	1
17037	497148	cyoB	0.470	2.587	0	1
15048	497315	cyoB, cyoA	0.560	2.268	0	1
6148	497720	cyoA	0.655	2.034	0	1
10662	497879	cyoA	0.831	2.064	0	1
1556	498065	cyoA	1.438	1.946	0	1

2415	498295	cyoA	1.555	1.020	0	0
19802	498453	cyoA	2.134	1.215	0	0
16831	498575	cyoA, ampG	2.236	1.172	0	0
2340	499001	ampG	1.356	1.063	0	0
17241	499365	ampG	1.350	1.330	0	0
22123	499496	ampG	0.997	1.376	0	0
20842	499667	ampG	1.063	1.385	0	0
6853	499881	ampG	0.511	0.895	0	0
20753	500141	ampG, yajG	0.822	1.011	0	0
5486	500342	ampG, yajG	0.697	0.850	0	0
9988	500684	yajG, bolA	1.002	1.342	0	0
10121	500878	yajG, bolA	0.903	1.141	0	0
2568	500981	yajG, bolA	1.100	0.806	0	0
13385	501320	tig	0.584	1.894	0	1
1196	501404	tig	0.605	2.138	0	1
4826	501639	tig	0.517	1.938	0	1
1178	501765	tig	0.641	2.016	0	1
1863	501948	tig	0.598	2.084	0	1
5572	502335	tig	0.951	2.217	0	1
15918	502517	tig	0.558	2.043	0	1
8147	502790	tig, clpP	0.543	1.975	0	1
3465	503172	clpP	0.601	1.910	0	1
9054	503290	clpP	0.582	1.972	0	1
14774	503468	clpP	0.528	1.655	0	0
21337	503780	clpP, clpX	0.540	1.870	0	1
4735	504056	clpX	0.595	2.268	0	1
9650	504196	clpX	0.631	2.090	0	1
15271	504525	clpX	0.684	1.999	0	1
9386	504762	clpX	0.458	1.246	0	0
21966	504937	clpX	0.547	1.687	0	1
12557	505221	clpX, lon	0.592	2.008	0	1
573	505447	lon	0.590	2.328	0	1
13823	505518	lon	0.530	2.665	0	1
16216	505625	lon	0.585	2.811	0	1
12710	506074	lon	0.644	1.858	0	0
5174	506219	lon	1.006	1.849	0	1

667	506543	lon	0.615	2.228	0	1
2711	506773	lon	0.530	1.574	0	0
11805	506984	lon	0.619	1.798	0	1
16290	507197	lon	0.753	1.967	0	1
11231	507487	lon	0.745	2.416	0	1
9270	507739	lon, hupB	0.762	2.028	0	1
13208	507963	hupB	0.521	1.771	0	0
15371	508175	hupB, cypD	0.620	2.621	0	1
11466	508399	cypD	0.577	2.614	0	1
21625	508621	cypD	0.612	2.110	0	1
20280	508693	cypD	0.483	1.793	0	0
10961	508934	cypD	0.660	1.761	0	0
21650	509290	cypD	0.750	1.374	0	0
21749	509433	cypD	0.832	1.388	0	0
7557	509657	cypD	0.834	1.230	0	0
9874	509782	cypD	0.879	1.331	0	0
3110	510106	cypD	0.733	1.081	0	0
13374	510457	cypD, ybaV	1.022	1.233	0	0
7408	510654	ybaV	0.789	0.600	0	0
15047	510827	ybaV, ybaW	0.996	0.863	0	0
1451	510944	ybaV, ybaW	0.965	0.722	0	0
7319	511026	ybaV, ybaW	0.886	0.730	0	0
20320	511103	ybaW	0.930	0.722	0	0
6980	511201	ybaW	0.960	0.875	0	0
3621	511859	ybaX	1.019	1.248	0	0
3171	512056	ybaX	0.997	1.345	0	0
20553	512362	ybaX	1.231	2.281	0	0
2853	512533	ybaX, ybaE	0.651	0.908	0	0
20437	512976	ybaE	1.125	0.926	0	0
16552	513077	ybaE	1.125	1.135	0	0
3768	513227	ybaE	0.945	0.906	0	0
3763	513399	ybaE	0.967	0.908	0	0
21190	513742	ybaE, cof	1.475	1.192	0	0
11618	514093	ybaE, cof	1.314	0.875	0	0
10273	514176	ybaE, cof	1.319	0.844	0	0
10520	514360	ybaE, cof	1.212	0.806	0	0

7673	514618	cof	1.106	0.847	0	0
13619	514742	cof	0.488	0.520	0	0
4368	515019	cof	1.073	0.852	0	0
10433	515386	STM0458	1.406	1.003	0	0
3558	515479	STM0458	1.286	0.958	0	0
22121	515910	STM0458, ybaO	1.447	1.153	0	0
6474	516021	STM0458, ybaO	1.572	1.092	0	0
7690	516167	STM0458, ybaO	1.402	0.925	0	0
17616	516247	STM0458, ybaO	1.165	0.908	0	0
2860	516677	ybaO, mdlA	1.009	0.935	0	0
20503	517034	mdlA	1.550	1.199	0	0
6875	517199	mdlA	1.480	1.124	0	0
22088	517270	mdlA	1.476	1.125	0	0
16666	517578	mdlA	1.019	0.979	0	0
13027	517917	mdlA	1.206	0.962	0	0
20877	518217	mdlA, mdlB	1.420	1.140	0	0
1860	518375	mdlA, mdlB	1.384	1.100	0	0
19374	518647	mdlB	1.365	1.356	0	0
10537	518887	mdlB	1.465	1.401	0	0
19171	519130	mdlB	1.511	1.617	0	0
12139	519295	mdlB	1.268	1.343	0	0
13509	519582	mdlB	1.190	1.100	0	0
10754	519685	mdlB	0.819	0.727	0	0
19554	519777	mdlB	0.736	0.599	0	0
21369	520218	mdlB, glnK	1.364	1.035	0	0
22357	520422	glnK, amtB	1.226	1.107	0	0
12279	520500	glnK, amtB	1.203	0.700	0	0
11394	520875	amtB	1.398	0.804	0	0
13591	521074	amtB	1.066	0.705	0	0
17707	521228	amtB	1.559	1.043	0	0
10637	521529	amtB	1.625	0.924	0	0
4820	521838	amtB	0.990	0.829	0	0
5725	521993	amtB	0.559	0.597	0	0
1724	522263	tesB	0.904	0.959	0	0
20559	522514	tesB	1.100	0.848	0	0
18902	522643	tesB	1.379	1.065	0	0

3784	522909	tesB, ybaY	1.009	0.880	0	0
3737	523209	tesB, ybaY	1.103	1.109	0	0
3616	523447	tesB, ybaY	0.760	0.958	0	0
12803	523628	ybaY	0.971	1.139	0	0
7066	523718	ybaY	0.983	1.310	0	0
5058	523940	ybaZ, ffs	0.842	1.542	0	0
20780	524235	ybaZ, ffs	0.593	4.375	0	1
3507	524597	ybaZ, ylaB	0.568	3.335	0	1
7960	524779	ybaZ, ylaB	0.737	2.061	0	1
1550	524850	ylaB	0.826	2.378	0	1
6641	525028	ylaB	0.765	1.481	0	0
15806	525445	ylaB	0.874	1.137	0	0
13796	525624	ylaB	1.077	1.158	0	0
16098	525780	ylaB	0.930	0.949	0	0
22295	526102	ylaB, rpmE2	0.766	0.827	0	0
12331	526260	ylaB, rpmE2	0.745	0.814	0	0
16521	526404	ylaB, rpmE2	0.753	1.051	0	0
3684	526732		0.658	1.609	0	0
21308	526888	ylaC	0.663	1.632	0	0
13610	527043	ylaC	0.489	1.544	0	0
18024	527282	ylaC	0.607	1.838	0	0
8609	527694	ylaC, maa	0.943	1.364	0	0
17211	527957	maa	1.527	1.267	0	0
10803	528148	maa, hha	1.322	1.136	0	0
6308	528449	hha, ybaJ	1.556	0.954	0	0
4828	528648	hha, ybaJ	1.533	0.840	0	0
11189	528731	hha, ybaJ	1.912	0.887	0	0
10892	528961	ybaJ	1.847	0.694	0	0
495	529224	acrB	2.487	0.747	0	0
11417	529508	acrB	1.451	1.050	0	0
6217	529817	acrB	0.915	1.179	0	0
9330	529888	acrB	0.827	0.973	0	0
13036	530184	acrB	1.090	1.194	0	0
14799	530419	acrB	0.886	0.947	0	0
15202	530565	acrB	1.053	1.200	0	0
6729	530719	acrB	0.968	1.096	0	0

18405	530791	acrB	0.730	0.797	0	0
8076	531377	acrB	0.857	0.854	0	0
6581	531546	acrB	0.916	1.320	0	0
17896	531808	acrB	0.887	1.244	0	0
11378	531958	acrB	0.718	1.134	0	0
8108	532109	acrB	0.756	1.178	0	0
9456	532372	acrB	0.885	1.771	0	1
11583	532460	acrB, acrA	0.781	2.322	0	1
3012	532736	acrB, acrA	1.087	2.516	0	1
9941	533177	acrA	0.710	1.144	0	0
12820	533328	acrA, acrR	0.743	1.416	0	0
16392	533593	acrA, acrR	0.796	1.613	0	0
12237	533721	acrA, acrR	0.838	1.506	0	0
19123	533955	acrA, acrR	1.147	1.302	0	0
20246	534266	acrR, aefA	1.291	1.190	0	0
5449	534499	aefA	1.429	1.133	0	0
5305	534685	aefA	1.234	1.176	0	0
21046	534939	aefA	1.038	1.159	0	0
250	535027	aefA	0.726	1.037	0	0
11923	535434	aefA	1.177	0.900	0	0
2577	535565	aefA	0.723	0.738	0	0
13055	535800	aefA	1.140	0.873	0	0
21866	535871	aefA	1.134	0.875	0	0
1207	536139	aefA	1.270	1.281	0	0
2702	536595	aefA	0.904	0.863	0	0
12649	536694	aefA	1.021	1.004	0	0
18296	536949	aefA	1.223	1.244	0	0
18481	537296	aefA	0.902	1.063	0	0
20378	537429	aefA	1.022	1.220	0	0
11054	537755	aefA	0.972	1.750	0	1
20394	537829	aefA	0.957	1.725	0	1
17469	538047	STM0479	0.610	1.331	0	0
15479	538292	STM0479	1.080	1.638	0	0
18734	538651	STM0479	0.758	0.889	0	0
16263	538723	STM0479	1.040	1.293	0	0
7978	539025	STM0479, ybaM	1.024	1.067	0	0

17537	539249	STM0479, ybaM	0.934	1.184	0	0
1523	539486	ybaM, priC	0.713	1.066	0	0
11518	539670	priC, ybaN	0.890	1.750	0	1
12725	539940	priC, ybaN	0.595	2.295	0	1
1694	540013	priC, ybaN	0.731	3.116	0	1
9335	540263	apt	0.659	2.971	0	1
13320	540366	apt	0.758	2.701	0	1
8532	540856	dnaX	0.700	2.716	0	1
16259	541128	dnaX	0.909	2.030	0	1
12945	541258	dnaX	0.854	2.001	0	1
12151	541437	dnaX	1.148	1.887	0	1
14970	541626	dnaX	0.598	1.056	0	0
3823	541870	dnaX	1.305	1.995	0	0
4777	542149	dnaX	1.607	1.579	0	0
18763	542359	dnaX	1.118	1.553	0	0
9959	542623	dnaX, ybaB	1.157	1.581	0	0
20050	542852	ybaB, recR	0.673	1.006	0	0
12589	543186	recR	0.893	1.942	0	0
19006	543437	recR, htpG	0.457	0.956	0	0
13959	543572	recR, htpG	0.839	1.999	0	0
10716	543904	htpG	0.763	1.976	0	1
13903	543993	htpG	0.637	2.002	0	1
11339	544088	htpG	0.623	1.707	0	0
1667	544500	htpG	0.752	2.090	0	0
4112	544620	htpG	0.758	2.011	0	0
4677	544825	htpG	0.628	2.020	0	1
1848	545165	htpG	0.905	1.844	0	1
15493	545309	htpG	0.781	2.067	0	1
13710	545612	htpG, adk	0.677	2.176	0	1
11787	545894	adk	0.590	1.954	0	1
5298	545972	adk	0.649	1.920	0	1
16630	546073	adk	0.672	1.911	0	1
12201	546505	adk, hemH	0.797	1.540	0	0
10483	546820	hemH	0.753	1.670	0	0
2727	546959	hemH	1.017	1.753	0	0
10737	547250	hemH	0.592	0.673	0	0

19187	547475	hemH	1.094	1.177	0	0
15306	547648	hemH	1.137	1.065	0	0
20469	547725	hemH	0.894	0.997	0	0
16485	548133	aes	1.153	1.425	0	0
2234	548230	aes	0.504	0.656	0	0
11478	548366	aes	1.236	0.900	0	0
7912	548811	aes, gsk	1.338	1.202	0	0
11651	549105	aes, gsk	1.335	1.373	0	0
18673	549367	gsk	1.130	1.235	0	0
13194	549596	gsk	1.217	1.375	0	0
17986	549714	gsk	1.186	1.326	0	0
18272	549855	gsk	1.172	1.305	0	0
12973	550094	gsk	1.329	2.119	0	0
15659	550422	ybaL	0.713	1.348	0	0
11501	550532	ybaL	1.080	1.537	0	0
4617	550851	ybaL	1.357	1.452	0	0
2873	551122	ybaL	1.286	1.119	0	0
5156	551239	ybaL	1.244	1.310	0	0
350	551426	ybaL	0.579	1.079	0	0
4369	551639	ybaL	0.581	0.723	0	0
10802	552022	ybaL	1.004	1.072	0	0
3706	552133	ybaL	0.883	0.766	0	0
1541	552229	ybaL, fsr	0.983	0.914	0	0
17238	552486	fsr	1.307	0.941	0	0
13788	552717	fsr	1.354	0.741	0	0
10190	553194	fsr, ushA	1.168	0.914	0	0
16945	553311	fsr, ushA	1.262	0.899	0	0
17761	553598	fsr, ushA	1.215	0.842	0	0
1684	553716	fsr, ushA	0.920	0.762	0	0
13195	554016	ushA	1.712	0.875	0	0
2112	554170	ushA	1.843	0.923	0	0
1040	554545	ushA	1.662	1.153	0	0
22051	554646	ushA	1.389	1.086	0	0
12189	554879	ushA	0.928	1.052	0	0
3261	555176	ushA	1.307	1.534	0	0
7942	555375	ybaK	0.953	1.555	0	0

14674	555450	ybaK	0.568	1.132	0	0
2053	555815	ybaK	0.784	1.999	0	1
866	555943	ybaK	0.864	2.244	0	1
22193	556015	ybaK	0.919	2.189	0	1
15534	556515	ybaP	1.129	1.252	0	0
11997	556629	ybaP	1.349	1.102	0	0
7415	556849	ybaP	2.213	1.011	0	0
9703	557147	ybaP, STM0497	3.971	0.715	1	0
9425	557500	STM0497	4.994	0.740	1	0
19752	557576	STM0497	3.864	0.624	1	0
14929	557687	STM0497	5.326	0.683	1	0
16869	558075	STM0497, copA	3.559	0.654	1	0
16107	558369	copA	1.935	0.805	0	0
8833	558616	copA	0.912	0.536	0	0
19472	558789	copA	1.298	0.695	0	0
20299	559124	copA	1.974	0.934	0	0
21742	559288	copA	1.627	0.806	0	0
13835	559544	copA	1.419	0.755	0	0
22041	559813	copA	1.142	0.840	0	0
2975	559940	copA	1.025	0.911	0	0
17152	560198	copA, cueR	1.051	0.864	0	0
15955	560422	copA, cueR	1.245	1.190	0	0
21361	560530	copA, cueR	1.060	0.953	0	0
15915	560713	copA, cueR	1.031	1.418	0	0
6112	560917	cueR	0.949	1.769	0	0
18712	561375	ybbJ	1.013	1.460	0	0
18349	561541	ybbJ, ybbK	1.244	1.089	0	0
12555	561781	ybbJ, ybbK	1.286	0.882	0	0
13485	562066	ybbK, ybbL	1.144	0.769	0	0
20955	562148	ybbK, ybbL	1.482	0.904	0	0
2040	562345	ybbK, ybbL	1.588	0.682	0	0
2418	562625	ybbK, ybbL	1.852	0.683	0	0
2918	562988	ybbL, ybbM	1.653	0.677	0	0
747	563059	ybbL, ybbM	1.733	0.806	0	0
1653	563391	ybbM	1.155	0.977	0	0
8030	563544	ybbM	0.566	0.701	0	0

6773	563837	ybbM	0.748	1.470	0	0
4444	564127	ybbN	0.868	2.016	0	1
19169	564317	ybbN	1.029	1.895	0	1
12591	564473	ybbN	0.498	1.134	0	0
9167	564811	ybbN	0.949	1.747	0	1
20665	564921	ybbN, ybbO	0.945	1.557	0	1
20575	565105	ybbN, ybbO	1.195	1.364	0	0
14179	565381	ybbO	0.687	0.850	0	0
18565	565682	ybbO	0.971	1.222	0	0
9523	565841	ybbO, tesA	1.098	1.552	0	0
4256	565950	ybbO, ybbA	1.132	1.447	0	0
3141	566259	ybbA, tesA	1.166	1.402	0	0
17139	566405	ybbA, tesA	1.253	1.341	0	0
19041	566690	ybbA, tesA	0.997	0.864	0	0
16121	566960	ybbA, ybbP	1.151	0.944	0	0
3523	567214	ybbP	1.601	0.915	0	0
3054	567523	ybbP	1.696	0.927	0	0
5946	567732	ybbP	1.346	0.756	0	0
2996	567822	ybbP	1.634	0.895	0	0
13381	568036	ybbP	0.983	0.759	0	0
5957	568146	ybbP	0.917	0.665	0	0
13491	568590	ybbP	1.359	0.863	0	0
12717	568874	ybbP	1.520	0.890	0	0
12855	569052	ybbP	1.324	0.814	0	0
21929	569265	ybbP, STM0509	1.191	0.965	0	0
16603	569592	STM0509	1.266	0.507	0	0
16574	569681	STM0509	1.107	0.400	0	0
10571	569880	STM0509	1.369	0.807	0	0
3925	570135	STM0509	0.534	0.646	0	0
7698	570290	STM0509	0.846	0.976	0	0
16434	570540	STM0509	0.816	0.836	0	0
8758	570774	STM0509, sfbA	0.884	0.751	0	0
8209	570942	sfbA	0.909	0.628	0	0
621	571347	sfbA	0.962	0.719	0	0
14330	571458	sfbA	1.109	0.690	0	0
4144	571780	sfbA, sfbB	1.421	0.720	0	0

778	571949	sfbB	1.497	0.823	0	0
20297	572230	sfbB	1.271	0.930	0	0
2606	572527	sfbB, sfbC	0.968	0.795	0	0
8129	572680	sfbB, sfbC	0.930	0.676	0	0
9196	572986	sfbC	1.590	0.725	0	0
3555	573091	sfbC	1.586	0.838	0	0
5104	573162	sfbC	0.946	0.626	0	0
15010	573652		1.129	1.006	0	0
5986	573743	ybbB	1.153	1.079	0	0
2968	573911	ybbB	0.894	0.904	0	0
4150	574150	ybbB	1.290	0.914	0	0
16163	574412	ybbB	1.176	1.004	0	0
6718	574746	ybbB	1.129	0.983	0	0
20460	574828	ybbB, ybbS	1.174	0.877	0	0
1349	575228	ybbS	1.155	0.824	0	0
15862	575382	ybbS	1.244	0.757	0	0
17261	575642	ybbS, allA	0.784	0.657	0	0
11904	575908	ybbS, allA	0.954	0.587	0	0
21086	575982	ybbS, allA	1.055	0.603	0	0
13154	576065	ybbS, allA	1.030	0.700	0	0
19325	576485	allR	1.004	0.835	0	0
10219	576793	allR	1.040	0.837	0	0
14713	576929	allR	1.183	0.980	0	0
13236	577162	allR, gcl	0.602	0.619	0	0
14910	577434	gcl	1.366	0.781	0	0
7484	577548	gcl	1.230	0.691	0	0
13040	577943	gcl	1.159	0.536	0	0
9970	578129	gcl	0.948	0.634	0	0
12398	578315	gcl	1.412	0.568	0	0
21232	578599	gcl	1.392	0.671	0	0
2431	578847	gcl, gip	1.646	0.554	0	0
15096	579135	gcl, gip	2.400	0.545	1	0
19073	579240	gip	2.552	0.523	1	0
12198	579363	gip	3.140	0.565	1	0
17071	579527	gip	2.488	0.601	1	0
19092	579940	gip, glxR	0.941	0.352	0	0

6878	580112	glxR	3.305	0.560	0	0
19253	580383	glxR	2.236	0.552	0	0
4330	580551	glxR	1.886	0.626	0	0
20502	580811	glxR, STM0520	2.371	0.548	0	0
21453	581102	STM0520	1.579	0.624	0	0
7129	581268	STM0520	2.614	0.487	1	0
5976	581527	STM0520	2.562	0.587	1	0
20479	581731	STM0520	1.988	0.566	0	0
10972	581903	STM0520	2.418	0.580	0	0
1866	582093	STM0520, ybbV	2.316	0.496	0	0
15272	582482	ybbV, allP	3.656	0.495	0	0
22276	582572	ybbV, allP	3.316	0.430	0	0
11657	582818	allP	4.155	0.536	1	0
21314	582895	allP	3.949	0.598	1	0
16566	583437	allP	4.168	0.450	1	0
16747	583581	allP, allB	5.139	0.450	1	0
2350	583819	allP, allB	4.445	0.526	1	0
11801	583925	allB	3.992	0.564	1	0
20201	584250	allB	2.500	0.592	1	0
17301	584475	allB	1.291	0.544	0	0
11252	584762	allB	1.483	0.535	0	0
6768	584838	allB	1.922	0.576	0	0
12832	585133	allB, ybbY	2.182	0.417	0	0
8206	585431	ybbY	1.487	0.581	0	0
19881	585665	ybbY	0.978	0.647	0	0
1125	585905	ybbY	1.479	0.556	0	0
12884	586149	ybbY	2.640	0.791	0	0
15091	586349	ybbY, glxK	2.423	0.475	0	0
13300	586558	ybbY, glxK	3.773	0.619	1	0
20686	586714	glxK	4.081	0.610	1	0
12175	586870	glxK	2.808	0.635	1	0
1854	587235	glxK	1.162	0.484	0	0
1004	587371	glxK	1.368	0.583	0	0
14948	587746	glxK	1.832	0.766	0	0
6941	587925	ylbA	1.622	0.842	0	0
12891	588167	ylbA	1.890	1.062	0	0

10430	588394	ylbA	1.960	0.908	0	0
8620	588672	ylbA	1.539	0.682	0	0
20696	588743	ylbA, allC	1.677	0.689	0	0
6384	589086	ylbA, allC	1.485	0.586	0	0
7623	589167	allC	1.512	0.554	0	0
2651	589344	allC	1.228	0.502	0	0
14021	589695	allC	3.709	0.731	1	0
8899	589913	allC	4.835	0.591	1	0
16530	590151	allC, allD	5.015	0.706	1	0
7736	590226	allC, allD	5.342	0.793	1	0
18630	590308	allC, allD	3.059	0.648	1	0
20551	590715	allD	2.930	0.653	1	0
1324	590990	allD, fdrA	4.116	0.606	1	0
12942	591250	allD, fdrA	5.304	0.548	1	0
9889	591417	allD, fdrA	3.931	0.608	1	0
6023	591782	fdrA	3.329	0.656	0	0
1782	591971	fdrA	1.495	0.626	0	0
16024	592284	fdrA	1.936	0.679	0	0
12486	592475	fdrA	1.733	0.678	0	0
19005	592560	fdrA, ylbE	0.887	0.466	0	0
5556	592988	fdrA, ylbE	1.065	0.326	0	0
16532	593106	ylbE	1.898	0.735	0	0
3736	593366	ylbE	1.871	0.674	0	0
19310	593669	ylbE	1.825	0.665	0	0
1971	593864	ylbE, ylbF	1.089	0.717	0	0
18495	594035	ylbE, ylbF	1.657	0.582	0	0
15953	594283	ylbF	2.106	0.668	0	0
21262	594365	ylbF	0.820	0.742	0	0
8159	594510	ylbF	1.352	0.649	0	0
17679	594934	ylbF, arcC	1.791	0.740	0	0
15542	595085	arcC	1.683	0.665	0	0
16887	595453	arcC	2.116	0.762	0	0
13169	595599	arcC	1.135	0.723	0	0
11075	595750	arcC	1.086	0.738	0	0
22343	596146	purK	1.422	2.235	0	0
16320	596238	purK	1.059	1.475	0	0

1659	596554	purK	1.549	1.684	0	1
20905	596627	purK	1.565	1.863	0	1
7080	596846	purK	1.483	1.377	0	0
6437	597097	purK, purE	1.423	1.640	0	0
13314	597455	purK, purE	0.981	1.238	0	0
6618	597654	purE	0.663	0.933	0	0
17198	597737	purE, lpxH	0.951	1.541	0	0
1317	598219	lpxH	0.911	1.603	0	0
5196	598439	lpxH, ppiB	0.879	1.708	0	1
18655	598545	lpxH, ppiB	0.751	1.698	0	1
21561	598726	lpxH, ppiB	0.739	2.069	0	1
21565	599033	ppiB, cysS	0.746	1.346	0	0
6523	599288	ppiB, cysS	0.962	1.453	0	0
16466	599474	cysS	1.082	1.367	0	0
16385	599639	cysS	0.969	1.327	0	0
16575	599745	cysS	1.083	1.632	0	0
14938	600159	cysS	1.026	1.473	0	0
3915	600366	cysS	1.350	1.550	0	0
4412	600559	STM0538	0.365	0.619	0	0
20410	600745	STM0538	1.311	1.156	0	0
20703	601025	STM0538	1.128	0.793	0	0
3114	601300	STM0538	1.190	0.747	0	0
887	601545	STM0538, STM0539	1.552	0.808	0	0
15420	601839	STM0539, ybcI	1.286	0.794	0	0
17260	602027	STM0539, ybcI	1.452	0.917	0	0
5543	602131	STM0539, ybcI	1.424	0.884	0	0
7499	602368	ybcI	1.107	1.176	0	0
15207	602672	ybcI, ybcJ	0.988	1.282	0	0
11633	602848	ybcJ, folD	1.291	1.385	0	0
18728	603164	folD	0.555	0.689	0	0
7307	603266	folD	1.121	1.046	0	0
18828	603553	folD	2.537	0.744	0	0
7067	603735	folD, fimA	4.026	0.683	1	0
19273	604070	fimA	4.859	0.597	1	0
14601	604183	fimA	4.745	0.744	1	0
19870	604311	fimA, fimI	3.351	1.015	1	0

12356	604703	fimI	1.916	0.717	0	0
8933	604877	fimI, fimC	2.064	0.650	0	0
15594	605150	fimI, fimC	3.105	0.611	1	0
13067	605442	fimC	4.606	0.669	1	0
19165	605655	fimC, fimD	2.069	0.546	0	0
18478	605805	fimC, fimD	1.520	0.505	0	0
19568	605876	fimC, fimD	2.240	0.533	0	0
4339	606247	fimD	1.675	0.557	0	0
6506	606587	fimD	1.541	0.669	0	0
1270	606773	fimD	1.274	0.636	0	0
18337	607044	fimD	1.390	0.710	0	0
7700	607157	fimD	1.444	0.762	0	0
8083	607470	fimD	1.864	0.589	0	0
654	607567	fimD	1.482	0.733	0	0
20318	607642	fimD	1.794	0.635	0	0
16539	608118	fimD	1.788	0.780	0	0
3377	608288	fimD, fimH	0.924	0.498	0	0
19479	608650	fimH	1.438	0.741	0	0
3766	608884	fimH	1.866	0.736	0	0
12980	609135	fimH	1.938	0.678	0	0
11156	609346	fimH, fimF	1.654	0.718	0	0
9407	609518	fimH, fimF	1.573	0.627	0	0
16050	609671	fimF	1.426	0.781	0	0
5386	609753	fimF	1.386	0.667	0	0
5278	610086	fimF	2.019	0.547	0	0
19848	610184		2.009	0.493	0	0
21329	610594	fimZ	2.700	0.496	1	0
16660	610848	fimZ	2.607	0.492	1	0
13210	611106	fimZ	2.712	0.464	1	0
5246	611334		1.686	0.456	0	0
14989	611548	fimY	1.693	0.557	0	0
1605	611657	fimY	1.723	0.504	0	0
16229	611909	fimY	1.762	0.501	0	0
22184	612289	fimY, STM0551	2.636	0.566	1	0
8327	612425	fimY, STM0551	2.504	0.555	1	0
10486	612538	fimY, STM0551	2.808	0.518	1	0

13738	612872	STM0551, fimW	2.489	0.652	1	0
5700	612998	fimW	2.194	0.672	0	0
1579	613439	fimW, argU	1.309	1.573	0	0
19915	613677	fimW, STM0554	0.911	1.240	0	0
19396	613892	STM0554	0.936	0.997	0	0
15833	614134	STM0554	1.175	0.757	0	0
4140	614237	STM0556	1.252	0.646	0	0
7759	614492	STM0556	1.738	0.509	0	0
2747	614693	STM0556	2.667	0.588	0	0
15900	614853	STM0556, STM0557	2.276	0.548	0	0
4166	615229	STM0557	3.125	0.470	1	0
11953	615449	STM0557	2.561	0.459	1	0
16453	615559	STM0557	2.039	0.483	0	0
2670	615720	STM0557	1.990	0.525	0	0
2784	616026	STM0557	2.049	0.443	0	0
14292	616312	STM0557	2.087	0.474	0	0
10291	616545	STM0557, yfdH	2.524	0.512	0	0
16102	616625	STM0557, yfdH	2.611	0.512	0	0
11057	617032	yfdH	1.588	0.528	0	0
19057	617233	yfdH	1.396	0.533	0	0
9494	617321	yfdH	1.333	0.494	0	0
15746	617659	yfdH	1.416	0.590	0	0
21710	617840		1.108	0.535	0	0
14450	618134		1.899	0.716	0	0
11008	618229		2.546	0.598	1	0
21277	618455		3.111	0.807	1	0
792	618568	STM0561, STM0562	3.555	0.892	1	0
6700	618804	STM0561, STM0562	2.260	0.722	0	0
16610	619300	STM0561, STM0563	1.694	1.000	0	0
10293	619429	STM0563	0.979	0.559	0	0
7356	619694	STM0563	1.093	0.587	0	0
7845	619805	STM0563	1.025	0.546	0	0
10935	620159	STM0563, STM0564	1.081	0.450	0	0
17746	620252	STM0563, STM0564	1.250	0.544	0	0
10735	620595	STM0564	1.245	0.622	0	0
515	620835	STM0564	1.695	0.665	0	0

16847	620924	STM0564	1.590	0.629	0	0
18845	621204	STM0564	1.761	0.707	0	0
15262	621586	STM0564, STM0565	3.503	0.603	1	0
16988	621791	STM0565, STM0566	4.361	0.644	1	0
3032	622040	STM0566	3.083	0.622	0	0
2779	622139	STM0566	2.978	0.589	1	0
11755	622212	STM0566	3.826	0.750	1	0
21197	622673	STM0567	1.397	1.163	0	0
983	622916	STM0567	1.224	1.007	0	0
7479	623012	STM0567	1.373	1.029	0	0
15711	623410	STM0567	1.402	0.909	0	0
18179	623535	STM0567	1.303	1.160	0	0
18924	623891	STM0567, pheP	1.298	0.912	0	0
21967	624072	STM0567, pheP	1.423	1.026	0	0
7381	624147	STM0567, pheP	1.146	0.894	0	0
11375	624434	pheP	1.321	1.071	0	0
18756	624629	pheP	1.249	0.920	0	0
21098	624920	pheP	1.166	1.014	0	0
4405	625025	pheP	1.101	1.039	0	0
8473	625326	pheP	1.048	1.148	0	0
20214	625682	ybdG	1.311	1.045	0	0
19511	625757	ybdG	0.923	0.734	0	0
1632	625959	ybdG	1.414	0.790	0	0
5178	626171	ybdG	2.183	0.781	0	0
4655	626376	ybdG	2.154	0.592	0	0
15685	626654	ybdG	4.198	0.929	1	0
22222	626961	ybdG, apeE	3.032	0.742	1	0
282	627166	ybdG, apeE	3.129	0.807	1	0
17042	627334	apeE	2.598	0.803	1	0
15477	627489	apeE	1.037	0.694	0	0
15451	627937	apeE	1.166	0.758	0	0
4381	628170	apeE	1.731	0.876	0	0
2908	628411	apeE	1.892	0.811	0	0
19967	628482	apeE	1.475	0.789	0	0
2390	628787	apeE	1.472	0.930	0	0
1174	629007	apeE, STM0571	1.364	0.910	0	0

2876	629326	STM0571	1.306	0.909	0	0
22104	629426	STM0571	1.052	1.007	0	0
10960	629829	STM0571	1.852	0.893	0	0
4559	629905	STM0571	1.871	0.914	0	0
21452	630228	STM0571	2.177	0.909	0	0
2678	630307	STM0571	1.731	0.820	0	0
2439	630591	STM0571	2.096	0.724	0	0
10357	630704	STM0571	1.821	0.795	0	0
7089	630845	STM0571	1.602	0.759	0	0
9223	631379	STM0571	2.563	0.615	1	0
3172	631605	STM0571	4.500	0.580	1	0
21763	631703	STM0571	4.829	0.426	1	0
1370	631999	STM0571	5.192	0.635	1	0
19722	632180	STM0572	1.135	0.534	0	0
12176	632324	STM0572	2.582	0.795	0	0
10733	632491	STM0572	1.935	0.695	0	0
4701	632989	STM0572	5.814	0.732	1	0
22116	633087	STM0572	5.270	0.615	1	0
18474	633365	STM0572, STM0573	8.016	0.693	1	0
22259	633522	STM0572, STM0573	6.873	0.807	1	0
20622	633827	STM0573	2.122	0.781	0	0
2019	634058	STM0573	1.287	0.661	0	0
15177	634264	STM0573	0.992	0.586	0	0
2913	634348	STM0573, STM0574	1.516	0.645	0	0
6632	634822	STM0574	1.430	0.726	0	0
1418	634895	STM0574	1.317	0.658	0	0
8212	635155	STM0574, STM0575	1.641	0.644	0	0
1362	635274	STM0574, STM0575	1.075	0.558	0	0
19769	635431	STM0574, STM0575	1.975	0.734	0	0
14876	635898	STM0575	1.777	0.680	0	0
8016	636128	STM0575, STM0576	0.857	0.715	0	0
19828	636379	STM0576, STM0577	1.141	0.599	0	0
7245	636509	STM0576, STM0577	1.064	0.657	0	0
21039	636812	STM0576, STM0577	1.013	0.632	0	0
22237	636896	STM0577	1.631	0.868	0	0
16798	637114	STM0577, nfnB	0.801	0.818	0	0

18958	637245	STM0577, nfnB	1.034	0.875	0	0
11246	637653	nfnB	1.161	0.913	0	0
22331	637805	nfnB, ybdF	0.988	0.973	0	0
3000	637937	nfnB, ybdF	1.083	0.956	0	0
829	638428	ybdF, STM0580	0.877	0.888	0	0
14983	638500	ybdF, STM0580	0.791	0.736	0	0
22018	638695	STM0580, STM0581	0.698	0.949	0	0
2525	639043	STM0580, STM0581	0.865	1.139	0	0
18101	639209	STM0581	0.500	0.711	0	0
19102	639577	ybdJ	1.022	1.179	0	0
1670	639665	ybdJ, ybdK	0.802	0.867	0	0
4900	639933	ybdJ, ybdK	1.448	0.914	0	0
5458	640117	ybdK	1.359	0.761	0	0
4605	640379	ybdK	1.237	0.708	0	0
18035	640614	ybdK	1.247	0.737	0	0
21949	640762	ybdK	1.382	0.688	0	0
16309	640864	ybdK, entD	1.462	0.799	0	0
8200	641151	ybdK, entD	0.858	0.599	0	0
4251	641534	entD, fepA	1.295	0.887	0	0
21157	641787	entD, fepA	1.106	0.884	0	0
14775	641945	entD, fepA	1.182	0.889	0	0
6900	642285	fepA	1.136	0.715	0	0
18180	642484	fepA	1.077	0.665	0	0
14176	642654	fepA	0.931	0.584	0	0
10154	642891	fepA	1.137	0.759	0	0
1286	643068	fepA	0.540	0.291	0	0
20617	643254	fepA	0.773	0.625	0	0
11943	643441	fepA	1.088	0.652	0	0
10435	643786	fepA, fes	1.396	0.627	0	0
5022	643973	fepA, fes	1.192	0.590	0	0
1820	644045	fepA, fes	1.641	0.645	0	0
11472	644509	fes	1.296	0.625	0	0
3049	644588	fes	1.315	0.780	0	0
20267	644670	fes	0.667	0.501	0	0
21292	645211	ybdZ, entF	1.569	0.549	0	0
2847	645416	ybdZ, entF	1.557	0.546	0	0

10465	645595	entF	2.066	0.725	0	0
18318	645815	entF	2.182	0.795	0	0
1358	645961	entF	1.364	0.519	0	0
7744	646228	entF	0.863	0.457	0	0
21737	646510	entF	1.862	0.957	0	0
10611	646761	entF	1.475	0.717	0	0
19091	646858	entF	1.745	0.678	0	0
12052	647218	entF	1.069	0.589	0	0
2863	647366	entF	1.828	0.783	0	0
7050	647759	entF	1.579	0.809	0	0
21442	647861	entF	1.167	0.632	0	0
18539	648007	entF	1.051	0.472	0	0
15025	648409	entF	1.279	0.467	0	0
17619	648660	entF	2.119	0.904	0	0
1491	648776	entF	1.182	0.547	0	0
3009	648989	entF	3.316	1.054	1	0
5162	649289	entF, fepE	3.132	0.710	1	0
17319	649544	fepE	2.503	0.638	0	0
8372	649679	fepE	2.010	0.645	0	0
5032	649863	fepE	1.752	0.611	0	0
7027	650037	fepE	2.843	0.755	1	0
13327	650259	fepE	2.739	0.836	1	0
7029	650535	fepE	2.079	0.976	1	0
14476	650820	fepC	1.124	0.934	0	0
7262	651001	fepC	1.588	1.223	0	0
16278	651237	fepC	1.521	1.063	0	0
5722	651561	fepC, fepG	2.467	1.106	0	0
9636	651651	fepC, fepG	2.139	1.001	0	0
13278	652068	fepG	2.119	0.836	0	0
12064	652275	fepG	0.882	0.703	0	0
12124	652362	fepG	1.802	0.743	0	0
14003	652662	fepG, fepD	0.893	0.488	0	0
20509	652825	fepG, fepD	2.092	0.907	0	0
20604	653001	fepG, fepD	1.424	0.817	0	0
8826	653161	fepD	1.846	0.734	0	0
19262	653655	fepD, ybdA	1.407	0.708	0	0

18752	653804	fepD, ybdA	1.101	0.605	0	0
4673	654128	ybdA	0.718	0.458	0	0
2722	654300	ybdA	1.736	0.665	0	0
21537	654519	ybdA	1.251	0.772	0	0
12750	654657	ybdA	0.964	0.776	0	0
3062	654833	ybdA	0.351	0.372	0	0
18380	655011	fepB	1.493	0.830	0	0
4994	655269	fepB	1.163	0.760	0	0
5859	655457	fepB	1.337	0.768	0	0
13715	655919	fepB, entC	0.854	0.678	0	0
20991	656008	fepB, entC	0.932	0.674	0	0
9339	656233	fepB, entC	0.812	0.586	0	0
4229	656313	fepB, entC	0.874	0.603	0	0
5833	656740	entC	1.275	0.622	0	0
11561	656950	entC, entE	1.198	0.661	0	0
8923	657176	entC, entE	1.338	0.672	0	0
6891	657398	entC, entE	1.439	0.794	0	0
15132	657664	entE	1.279	0.862	0	0
1190	657847	entE	0.685	0.464	0	0
4966	658029	entE	1.371	0.679	0	0
2465	658403	entE	1.035	0.535	0	0
4974	658663	entE, entB	1.260	0.559	0	0
18513	658830	entE, entB	0.906	0.476	0	0
17276	659030	entB	1.463	0.727	0	0
690	659254	entB	1.515	0.751	0	0
9912	659468	entB, entA	1.570	0.747	0	0
6374	659756	entB, entA	1.257	0.729	0	0
13109	659900	entA	1.532	0.737	0	0
17895	660029	entA	1.624	0.829	0	0
9423	660213	entA, ybdB	0.312	0.500	0	0
1058	660585	entA, ybdB	0.924	0.648	0	0
12382	660677	ybdB	1.302	0.841	0	0
17938	661076	cstA	0.932	1.173	0	0
18612	661242	cstA	0.877	1.216	0	0
15259	661381	cstA	0.454	0.633	0	0
20522	661490	cstA	0.841	1.671	0	0

21940	661852	cstA	0.866	2.541	0	0
1827	661930	cstA	0.657	1.100	0	0
3014	662397	cstA	0.612	1.409	0	0
5126	662664	cstA	0.581	1.152	0	0
6441	662758	cstA	0.604	1.004	0	0
4331	663181	cstA, ybdD	0.382	0.973	0	0
6755	663404	ybdD	0.682	1.195	0	0
11153	663654	ybdH	0.615	1.468	0	0
17356	663903	ybdH	0.833	2.173	0	0
6496	664099	ybdH	1.209	1.448	0	0
17065	664313	ybdH	1.139	1.012	0	0
5724	664543	ybdH, ybdL	1.968	0.893	0	0
12735	664816	ybdH, ybdL	0.951	0.575	0	0
18352	664902	ybdH, ybdL	1.711	0.647	0	0
7860	665121	ybdH, ybdL	1.641	0.776	0	0
11576	665457	ybdL	1.134	0.746	0	0
14399	665577	ybdL	1.455	0.853	0	0
20339	665808	ybdL	0.665	0.499	0	0
903	665960	ybdL	1.162	0.952	0	0
20396	666375	ybdM	1.198	0.854	0	0
399	666488	ybdM	1.061	0.763	0	0
20277	666613	ybdM, ybdN	0.725	0.533	0	0
7256	666954	ybdM, ybdN	1.273	0.651	0	0
15378	667228	ybdN	1.128	0.620	0	0
11333	667447	ybdN	1.871	0.580	0	0
11081	667635	ybdN	2.755	0.559	1	0
8464	667839	ybdN	3.722	0.567	1	0
9491	668185	ybdN, ybdO	3.816	0.584	1	0
19190	668393	ybdO	3.186	0.585	1	0
10719	668529	ybdO	3.114	0.575	1	0
21227	668730	ybdO	3.535	0.511	1	0
766	668898	ybdO	2.760	0.502	1	0
20821	669281	ybdO, dsbG	2.178	0.776	0	0
10982	669567	dsbG	1.360	0.721	0	0
10730	669669	dsbG	1.081	0.821	0	0
5028	669760	dsbG	1.099	0.734	0	0

1227	670213	dsbG, ahpC	1.339	0.974	0	0
4631	670327	dsbG, ahpC	1.096	0.979	0	0
9527	670669	ahpC	0.565	0.675	0	0
21507	670765	ahpC, ahpF	1.121	1.113	0	0
18448	670957	ahpC, ahpF	1.050	1.090	0	0
14134	671221	ahpF	1.588	1.115	0	0
3595	671603	ahpF	1.137	0.834	0	0
21570	671773	ahpF	1.450	0.976	0	0
2167	672000	ahpF	1.658	1.276	0	0
14321	672078	ahpF	1.063	0.946	0	0
20000	672422	ahpF	1.223	0.823	0	0
2782	672636	ahpF	2.265	0.959	0	0
6883	672782	ahpF, STM0610	3.102	0.737	1	0
6493	673213	STM0610, STM0611	5.074	0.516	1	0
21306	673438	STM0610, STM0611	7.370	0.616	1	0
8736	673674	STM0610, STM0611	6.055	0.631	1	0
12108	673874	STM0611	2.988	0.560	1	0
6415	674111	STM0611	1.931	0.655	0	0
17724	674294	STM0611	1.623	0.665	0	0
13168	674556	STM0611	1.526	0.791	0	0
15117	674824	STM0611	1.433	0.724	0	0
3097	674937	STM0611	1.450	0.755	0	0
3932	675189	STM0611	1.260	0.668	0	0
14499	675340	STM0611	1.516	0.773	0	0
13633	675661	STM0611, STM0612	1.338	0.762	0	0
11519	675806	STM0611, STM0612	1.369	0.832	0	0
12639	675953	STM0611, STM0612	1.534	0.718	0	0
21512	676307	STM0612, STM0613	1.507	0.745	0	0
10333	676557	STM0613	1.152	0.680	0	0
3862	676722	STM0613	1.260	0.769	0	0
15682	676920	STM0613	0.974	1.092	0	0
11547	677247	STM0613	0.956	1.414	0	0
15275	677529	ybdQ, ybdR	1.212	1.732	0	0
1103	677756	ybdQ, ybdR	1.161	0.961	0	0
19002	677968	ybdQ, ybdR	1.900	0.953	0	0
3293	678138	ybdQ, ybdR	1.266	0.623	0	0

9651	678288	ybdR	1.327	0.644	0	0
15798	678570	ybdR	1.104	0.694	0	0
8537	678719	ybdR	1.420	0.791	0	0
7578	679048	ybdR	1.461	0.900	0	0
666	679300		1.451	1.298	0	0
5824	679557	rnk	0.949	1.496	0	0
16652	679748	rnk	0.842	2.150	0	1
22234	679866	rnk	0.873	2.215	0	1
20080	680101	rnk, rna	1.131	1.889	0	1
9517	680253	rna	0.986	1.512	0	0
8721	680646	rna	1.142	1.630	0	0
21217	680769	rna	1.297	1.597	0	0
15860	680865	rna	1.182	1.391	0	0
21510	681198	rna, citT	1.149	1.112	0	0
16314	681576	citT	1.337	0.868	0	0
3027	681734	citT	1.077	0.888	0	0
689	681917	citT	1.640	0.832	0	0
22275	682290	citT	1.187	0.602	0	0
2041	682401	citT, citG	1.756	0.725	0	0
19089	682473	citT, citG	1.995	0.766	0	0
18644	682615	citT, citG	0.939	0.659	0	0
19717	683219	citG	1.537	0.716	0	0
10958	683296	citG, citX	0.897	0.702	0	0
2260	683588	citG, citX	0.891	0.515	0	0
11296	683805	citX, citF	1.589	0.658	0	0
13756	683954	citX, citF	1.462	0.796	0	0
15389	684365	citF	1.747	0.699	0	0
13360	684529	citF	1.525	0.590	0	0
17705	684640	citF	1.367	0.784	0	0
19222	684855	citF	1.512	0.619	0	0
7344	685079	citF	1.527	0.654	0	0
2867	685340	citF, citE	1.591	0.657	0	0
5247	685578	citF, citE	1.369	0.676	0	0
5576	685892	citE	1.903	0.799	0	0
22320	686005	citE	1.742	0.917	0	0
2052	686216	citE	1.297	0.587	0	0

13733	686545	citE, citD	0.952	0.526	0	0
1133	686794	citD, citC	1.662	0.584	0	0
7036	687047	citC	2.013	0.511	0	0
20804	687298	citC	4.129	0.647	1	0
13048	687489	citC	4.203	0.582	1	0
20097	687694	citC, dpiB	4.320	0.438	1	0
397	687914	citC, dpiB	2.918	0.505	0	0
16101	688155	dpiB	4.321	0.518	0	0
380	688406	dpiB	2.261	0.620	0	0
1359	688509	dpiB	2.046	0.578	0	0
2152	688701	dpiB	1.834	0.881	0	0
12298	688824	dpiB	1.707	0.782	0	0
2328	689349	dpiB, dpiA	1.371	0.735	0	0
14036	689582	dpiB, dpiA	2.077	0.783	0	0
13995	689778	dpiA	2.085	0.713	0	0
5143	689938	dpiA	1.703	0.816	0	0
1264	690129	dpiA	1.200	0.660	0	0
7833	690243	dpiA	1.575	0.761	0	0
21216	690595	dcuC	1.336	0.763	0	0
9036	690723	dcuC	2.111	0.768	0	0
3105	691070	dcuC	1.478	0.703	0	0
773	691162	dcuC	1.608	0.704	0	0
9012	691398	dcuC	2.109	0.658	0	0
13762	691745	dcuC, pagP	3.578	0.603	1	0
16182	692025	dcuC, pagP	3.242	0.683	1	0
17352	692144	dcuC, pagP	2.820	0.676	1	0
10517	692431	pagP, cspE	1.270	1.094	0	0
5418	692707	cspE	0.657	1.678	0	0
5730	692819	cspE	0.578	1.998	0	0
4348	693093	cspE	0.673	2.149	0	1
21355	693344	crcB, ybeM	0.752	1.638	0	1
8977	693567	crcB, ybeM	0.886	1.089	0	0
5702	693854	crcB, ybeM	1.073	0.948	0	0
12278	693974	crcB, ybeM	1.181	0.959	0	0
17890	694264	ybeM, ybeC	0.748	0.686	0	0
2476	694553	ybeC	0.810	0.790	0	0

9408	694828		0.735	0.995	0	0
1212	694937	lipA	0.753	1.208	0	0
1589	695081	lipA	0.704	1.015	0	0
11692	695295	lipA	0.646	0.878	0	0
22141	695556	lipA	0.822	1.508	0	0
10129	695831	lipA	0.846	1.100	0	0
12519	695977	lipA	0.789	1.053	0	0
16550	696234	lipA, ybeF	1.090	1.264	0	0
17252	696453	ybeF	1.440	1.152	0	0
4905	696819	ybeF	3.631	0.822	0	0
12364	696893	ybeF	2.266	0.664	0	0
18378	697175	ybeF	1.380	0.620	0	0
3445	697429	lipB	2.871	0.885	0	0
18775	697572	lipB	1.253	0.656	0	0
9670	697804	lipB	1.248	1.241	0	0
18227	698003	lipB, ybeD	0.916	1.237	0	0
14465	698254	lipB, ybeD	0.752	1.471	0	0
1678	698337	ybeD	0.786	1.266	0	0
15360	698579	ybeD, dacA	0.948	1.520	0	0
9742	698954	dacA	0.907	1.186	0	0
6731	699331	dacA	0.805	1.753	0	1
8611	699599	dacA	0.911	2.322	0	1
18629	699704	dacA	0.935	2.735	0	1
22242	699775	dacA, rlpA	0.950	3.048	0	1
7467	700200	rlpA	1.279	2.266	0	1
8694	700376	rlpA	1.210	1.943	0	1
8911	700673	rlpA	1.038	2.160	0	1
21632	700905	rlpA, mrdB	0.895	1.472	0	0
18797	701017	rlpA, mrdB	0.710	1.268	0	0
364	701153	rlpA, mrdB	0.800	1.240	0	0
6515	701411	mrdB	1.484	1.548	0	0
17574	701482	mrdB	0.983	1.188	0	0
13252	701975	mrdB	1.058	1.581	0	0
19914	702066	mrdB, mrdA	0.636	0.880	0	0
618	702480	mrdA	1.024	1.516	0	0
17529	702700	mrdA	1.118	1.678	0	1

14625	702776	mrdA	1.017	1.603	0	1
2823	702997	mrdA	0.819	1.386	0	0
5075	703238	mrdA	0.859	1.241	0	0
13099	703616	mrdA	0.942	1.516	0	0
19803	703765	mrdA	0.869	1.503	0	0
12984	703931	mrdA, ybeA	0.449	0.979	0	0
15372	704307	mrdA, ybeA	0.906	1.650	0	0
17350	704398	ybeA, ybeB	0.941	1.548	0	0
16329	704632	ybeA, ybeB	1.042	1.861	0	0
10057	704907	ybeB	0.676	0.981	0	0
22327	705209	cobC	0.798	1.073	0	0
18642	705413	cobC, cobD	0.847	1.072	0	0
10245	705669	cobC, cobD	1.199	1.251	0	0
15450	705911	cobC, cobD	1.018	1.089	0	0
7322	706124	cobD	0.975	0.906	0	0
17507	706303	cobD	1.320	1.015	0	0
19701	706377	cobD	1.024	0.893	0	0
1573	706649	cobD	1.167	1.297	0	0
18953	707062	nadD	0.961	1.743	0	0
13862	707260	nadD	0.983	1.646	0	1
19461	707456	nadD	1.035	1.791	0	1
6985	707540	nadD, holA	0.865	1.347	0	0
770	707730	nadD, holA	0.919	1.519	0	0
13565	708151	holA	1.029	2.281	0	1
9296	708319	holA	0.910	1.922	0	1
14636	708492	holA, rlpB	0.674	1.238	0	0
13077	708896	holA, rlpB	0.734	1.536	0	0
7075	709138	rlpB, leuS	0.875	2.065	0	1
10380	709262	rlpB, leuS	0.672	1.661	0	1
406	709559	leuS	0.827	2.092	0	1
1489	709651	leuS	0.866	1.747	0	1
9355	709821	leuS	0.785	1.578	0	0
17514	710193	leuS	0.720	1.350	0	0
15342	710264	leuS	0.857	1.648	0	0
9593	710624	leuS	0.963	1.471	0	0
7284	710753	leuS	1.014	1.732	0	0

13061	711113	leuS	0.810	1.410	0	0
4947	711236	leuS	1.060	1.641	0	0
3820	711597	leuS, STM0649	0.751	2.379	0	1
10349	711726	leuS, STM0649	0.778	2.171	0	1
5768	711963	leuS, STM0649	0.925	1.261	0	0
16052	712270	STM0650	1.434	0.625	0	0
5919	712479	STM0650	2.039	0.700	0	0
3798	712589	STM0650	1.701	0.747	0	0
10331	712848	STM0650	2.235	0.746	0	0
19933	713021	STM0650	2.857	0.723	0	0
9634	713328	STM0650, STM0651	2.515	0.587	0	0
20587	713499	STM0651	2.850	0.679	1	0
869	713635	STM0651	2.180	0.769	1	0
18600	714109	STM0651, STM0652	1.551	0.717	0	0
7745	714181	STM0651, STM0652	1.411	0.718	0	0
20953	714548	STM0652	2.025	1.030	0	0
17453	714655	STM0652	3.037	0.909	1	0
15090	714808	STM0652	3.510	0.789	1	0
11742	715133	STM0652	2.898	0.763	1	0
9313	715238	STM0652	3.214	0.796	1	0
5740	715567	STM0652	1.867	0.972	0	0
14801	715783	STM0652	1.383	0.941	0	0
17715	715988	STM0652	1.457	1.340	0	0
19314	716358	STM0652, ybeL	1.641	1.164	0	0
16253	716537	ybeL	1.545	1.233	0	0
12568	716754	ybeL	1.128	1.091	0	0
12509	716895	ybeL	0.799	1.000	0	0
6843	717226	ybeQ	1.472	1.260	0	0
12361	717490	ybeQ	1.823	0.845	0	0
6577	717622	ybeQ	2.155	0.850	0	0
16733	717798	ybeQ, ybeR	3.706	0.745	1	0
4114	718116	ybeQ, ybeR	4.584	0.639	1	0
2091	718303	ybeQ, ybeR	4.742	0.580	1	0
8616	718677	ybeR, ybeS	2.212	0.607	1	0
5211	718855	ybeR, ybeS	1.516	0.548	0	0
703	719081	ybeS	1.838	0.674	0	0

20409	719178	ybeS	1.777	0.750	0	0
2615	719452	ybeS	0.598	0.705	0	0
10420	719540	ybeS	1.163	0.606	0	0
4312	719842	ybeS	1.551	0.656	0	0
15930	720074	ybeS, ybeU	2.100	0.763	0	0
9791	720457	ybeU	1.634	0.532	0	0
5460	720548	ybeU, ybeV	1.963	0.693	0	0
10232	720853	ybeU, ybeV	1.380	0.678	0	0
3237	721035	ybeV	1.370	0.733	0	0
16161	721306	ybeV	1.661	0.733	0	0
20270	721551	ybeV	1.591	0.777	0	0
3545	721656	ybeV	1.467	0.815	0	0
3278	722045	ybeV	1.515	0.865	0	0
1118	722280	ybeV	1.595	1.013	0	0
12535	722467	ybeV	0.802	0.592	0	0
8731	722687	hscC	1.337	0.754	0	0
5145	722845	hscC	1.497	0.808	0	0
18036	722951	hscC	1.400	0.823	0	0
5874	723396	hscC	1.221	0.580	0	0
1785	723543	hscC	2.059	0.702	0	0
17160	723848	hscC	4.324	0.700	1	0
1281	723944	hscC	4.419	0.790	1	0
13889	724175	hscC	4.450	0.858	1	0
15169	724441	hscC, STM0660	1.896	0.846	0	0
6970	724677	STM0660	1.424	0.961	0	0
20589	724889	STM0660	1.237	1.106	0	0
6467	725119	STM0660	1.245	1.114	0	0
21032	725430	STM0660	1.307	1.462	0	0
18246	725501	STM0660	1.127	1.203	0	0
480	725608	STM0660, ybeK	0.949	0.997	0	0
19278	725782	STM0660, ybeK	1.368	1.153	0	0
13782	725945	STM0660, ybeK	1.000	0.958	0	0
11858	726466	ybeK	0.741	1.352	0	0
11721	726666	ybeK, gltL	0.846	1.742	0	0
5758	726773	ybeK, gltL	0.689	1.475	0	0
414	727129	gltL	0.897	1.907	0	0

17203	727237	gltL	0.596	1.021	0	0
18334	727748	gltL, gltK	0.610	1.212	0	0
19200	727957	gltK	0.583	1.392	0	0
6019	728204	gltK, gltJ	0.688	2.109	0	1
16289	728394	gltK, gltJ	0.701	1.818	0	1
1457	728569	gltJ	0.831	2.692	0	1
11110	728908	gltJ, gltI	0.509	1.877	0	0
10179	728979	gltJ, gltI	0.573	2.253	0	1
14881	729273	gltI	0.660	2.058	0	1
7011	729458	gltI	0.654	1.985	0	1
2211	729780	gltI	0.528	1.952	0	1
22028	729890	gltI	0.349	1.097	0	0
296	730074	gltI	1.109	1.893	0	0
1273	730324	lnt	0.590	1.440	0	0
20475	730684	lnt	0.650	1.221	0	0
5363	730882	lnt	0.830	1.781	0	1
8318	730953	lnt	0.708	1.712	0	1
22021	731062	lnt	0.725	1.528	0	0
20942	731473	lnt	0.711	2.500	0	1
11427	731682	lnt	0.738	2.790	0	1
6092	732006	lnt, ybeX	0.639	1.826	0	1
19220	732228	ybeX	0.503	1.616	0	0
12288	732523	ybeX	0.503	1.603	0	0
276	732759	ybeX, ybeY	0.650	2.078	0	1
10953	732831	ybeX, ybeY	0.603	1.858	0	1
11908	733030	ybeX, ybeY	0.680	1.636	0	1
18717	733209	ybeY, phoL	0.659	1.707	0	1
7996	733514	ybeY, phoL	0.748	1.404	0	0
21503	733613	ybeY, phoL	0.786	1.676	0	1
17708	734138	phoL	0.817	2.432	0	1
10019	734244	phoL	0.761	2.760	0	1
17781	734466	phoL, miaB	0.507	1.437	0	0
7210	734604	phoL, miaB	0.905	2.029	0	0
6471	734908	miaB	0.449	0.572	0	0
21587	735154	miaB	0.552	0.947	0	0
20717	735409	miaB	1.094	1.248	0	0

460	735672	miaB, ubiF	0.986	1.858	0	1
387	735879	miaB, ubiF	1.248	2.604	0	1
4695	736029	miaB, ubiF	0.801	1.639	0	0
15979	736281	miaB, ubiF	0.744	1.143	0	0
5789	736587	ubiF	1.096	1.401	0	0
11304	736758	ubiF	1.050	1.399	0	0
6727	737066	ubiF	0.864	1.366	0	0
8957	737275	STM0672	0.554	0.867	0	0
21427	737467	STM0672	0.556	1.191	0	0
11535	737708	STM0672	0.464	2.162	0	1
376	737836	STM0672	0.437	3.091	0	1
7704	737926	STM0672	0.398	3.309	0	1
6404	738199	STM0672, glnX	0.342	4.266	0	1
19827	738433	glnX, glnV	0.355	4.046	0	1
20586	738779	glnW, glnU	0.392	3.183	0	1
17372	738854	glnU, leuW	0.582	4.005	0	1
16172	739199	asnB	0.691	2.075	0	1
12650	739472	asnB	0.741	1.219	0	0
21860	739630	asnB	0.752	0.883	0	0
520	739899	asnB	0.376	0.554	0	0
6888	740141	asnB	1.009	0.828	0	0
6061	740433	asnB	0.908	0.901	0	0
19274	740677	asnB	0.810	0.803	0	0
19893	740753	asnB	0.738	0.912	0	0
20244	740940	asnB, nagD	0.801	1.094	0	0
7844	741254	nagD	0.958	1.219	0	0
15673	741609	nagD	0.869	1.436	0	0
8840	741826	nagD, nagC	0.760	1.087	0	0
15781	742017	nagD, nagC	0.943	1.280	0	0
2760	742169	nagC	0.884	1.324	0	0
2555	742452	nagC	0.923	1.591	0	0
9087	742566	nagC	0.461	0.886	0	0
7456	742872	nagC	0.659	1.375	0	0
6963	743166	nagC, nagA	0.510	1.031	0	0
13683	743385	nagA	0.796	1.864	0	0
21927	743615	nagA	0.774	1.665	0	0

13235	743784	nagA	0.721	1.288	0	0
9881	744118	nagA, nagB	0.288	0.552	0	0
11227	744208	nagA, nagB	0.472	0.749	0	0
11196	744531	nagB	0.638	1.047	0	0
7540	744629	nagB	0.656	1.106	0	0
8632	744904	nagB, nagE	0.566	0.859	0	0
15447	745119	nagB, nagE	0.858	1.120	0	0
17946	745224	nagB, nagE	0.915	1.087	0	0
14295	745618	nagE	1.022	1.421	0	0
9661	745780	nagE	0.737	1.291	0	0
10703	746130	nagE	0.695	0.947	0	0
822	746366	nagE	1.074	1.125	0	0
4981	746466	nagE	1.015	1.496	0	0
10674	746745	nagE	0.685	0.944	0	0
21638	746889	nagE	0.657	1.096	0	0
1199	747299	glnS	0.907	2.003	0	1
6458	747374	glnS	1.157	1.997	0	1
18237	747639	glnS	0.792	1.809	0	1
487	747871	glnS	1.144	1.614	0	0
3306	748014	glnS	0.630	1.116	0	0
11022	748091	glnS	0.746	1.400	0	0
7568	748456	glnS	1.071	1.294	0	0
4909	748699	glnS	0.907	1.309	0	0
4714	749136	ybfM	1.973	1.006	0	0
19022	749255	ybfM	2.099	0.955	0	0
14857	749491	ybfM	2.149	1.347	0	0
13766	749720	ybfM	2.199	1.453	0	0
5749	749845	ybfM	2.359	1.300	0	0
5852	750134	ybfM	2.620	1.177	0	0
7492	750249	ybfM	1.852	1.031	0	0
11636	750537	ybfM, ybfN	1.222	0.960	0	0
21839	750799	ybfM, ybfN	1.132	1.003	0	0
4686	750893	ybfM, ybfN	1.196	1.027	0	0
12711	751140	ybfN	1.110	0.957	0	0
5854	751293		1.213	1.078	0	0
13825	751628	citA	0.631	0.660	0	0

7200	752037	citA	1.727	0.984	0	0
15516	752205	citA	1.271	0.818	0	0
12862	752472	citA	1.225	0.736	0	0
7081	752543	citA	1.846	0.853	0	0
4698	752684	citA	0.665	0.640	0	0
11066	753132	citB	1.371	0.661	0	0
2373	753370	citB	0.929	0.692	0	0
10542	753671	citB	1.597	0.737	0	0
10260	753810	citB	0.919	0.644	0	0
20603	753929	citB, STM0691	1.114	1.083	0	0
18068	754315	STM0691	1.195	1.257	0	0
22030	754387	STM0691	1.433	1.369	0	0
17573	754556	STM0691	1.539	1.161	0	0
20786	754933	STM0691	1.400	1.051	0	0
16562	755226	STM0691	0.530	0.665	0	0
21785	755450	STM0691, STM0692	0.672	0.914	0	0
3543	755689	STM0691, STM0692	0.518	0.921	0	0
13201	755919	STM0692	0.805	1.518	0	0
7773	756149	STM0692	0.784	1.866	0	1
7215	756272	STM0692	0.724	1.785	0	1
18453	756524	STM0692, fur	0.608	1.726	0	0
17315	756821	fur	0.500	1.790	0	0
14653	756916	fur	0.553	1.805	0	1
3892	757021	fur	0.626	1.719	0	1
319	757529	fldA	0.552	1.411	0	0
17101	757628	fldA	0.603	1.509	0	0
6562	757767	fldA	0.634	1.735	0	1
17889	758057	fldA, ybfE	0.720	2.774	0	1
14752	758200	ybfE	0.654	1.774	0	0
1041	758494	ybfE, ybfF	0.756	1.316	0	0
21705	758838	ybfF, seqA	0.771	1.545	0	0
11766	758985	ybfF, seqA	0.710	1.688	0	0
19345	759147	ybfF, seqA	0.747	1.691	0	0
10388	759447	seqA, pgm	0.587	1.216	0	0
9525	759703	seqA, pgm	0.890	1.648	0	0
17428	759831	pgm	0.909	1.709	0	0

20881	760051	pgm	0.723	1.408	0	0
20721	760360	pgm	0.787	1.275	0	0
10227	760498	pgm	0.916	1.620	0	0
5166	760740	pgm	0.873	1.721	0	1
12726	760940	pgm	1.043	1.757	0	1
11065	761335	pgm	1.123	1.284	0	0
8351	761559	STM0699	0.575	0.746	0	0
17534	761831	STM0699	0.951	0.697	0	0
20274	761923	STM0699	1.268	0.745	0	0
11806	762289	STM0699, potE	1.285	0.730	0	0
14188	762443	potE	1.697	0.811	0	0
14986	762619	potE	1.327	0.673	0	0
1459	762814	potE	1.168	0.598	0	0
12916	763094	potE	1.508	0.706	0	0
2748	763311	potE	0.793	0.502	0	0
10679	763678	potE, speF	1.155	0.521	0	0
13098	763831	speF	1.278	0.622	0	0
7302	764082	speF	1.250	0.659	0	0
8149	764223	speF	1.252	0.676	0	0
15761	764465	speF	0.938	0.649	0	0
7137	764551	speF	1.069	0.632	0	0
19502	764939	speF	1.499	0.673	0	0
8071	765093	speF	2.415	0.777	1	0
1622	765304	speF	3.576	0.645	1	0
19761	765608	speF	4.855	0.652	1	0
18268	765887	speF	4.323	0.603	1	0
5787	766056		4.127	0.702	1	0
2705	766170		3.729	0.606	1	0
6268	766477	kdpE	3.447	0.745	1	0
6145	766779	kdpE	1.907	1.024	0	0
4725	766974	kdpE	1.139	0.842	0	0
14344	767247	kdpE, kdpD	1.093	0.831	0	0
3964	767488	kdpD	1.503	0.993	0	0
1606	767758	kdpD	1.559	1.003	0	0
3814	767829	kdpD	1.273	0.759	0	0
1456	768211	kdpD	0.763	0.619	0	0

16764	768360	kdpD	0.735	0.751	0	0
8522	768496	kdpD	0.770	0.751	0	0
12202	768909	kdpD	0.538	0.465	0	0
8141	769067	kdpD	1.407	0.981	0	0
6017	769241	kdpD	2.845	0.972	0	0
14870	769463	kdpD	0.790	0.612	0	0
20706	769648	kdpD	1.944	1.030	0	0
7892	769779	kdpD, kdpC	0.803	0.537	0	0
11190	770229	kdpC	1.630	0.729	0	0
11761	770322	kdpC, kdpB	0.330	0.533	0	0
15469	770639	kdpC, kdpB	1.745	0.849	0	0
10797	770919	kdpB	1.471	0.758	0	0
20490	771148	kdpB	2.023	0.918	0	0
19906	771252	kdpB	1.088	0.675	0	0
20738	771365	kdpB	1.078	0.722	0	0
15604	771632	kdpB	1.110	0.568	0	0
9514	771717	kdpB	2.114	0.836	0	0
19465	772256	kdpB	1.894	0.736	0	0
14090	772327	kdpB, kdpA	2.332	0.701	0	0
8737	772540	kdpB, kdpA	1.525	0.810	0	0
7161	772805	kdpA	1.453	0.723	0	0
20044	773117	kdpA	0.883	0.609	0	0
570	773429	kdpA	2.121	0.749	0	0
7822	773500	kdpA	1.308	0.542	0	0
4667	773844	kdpA	1.697	0.674	0	0
5168	774130	kdpA, STM0707	1.896	0.606	0	0
3232	774305	kdpA, STM0707	1.772	0.701	0	0
13708	774444	kdpA, STM0707	1.366	0.774	0	0
13957	774570	ybfA, phrB	1.279	0.655	0	0
1328	774720	phrB	1.438	0.900	0	0
1209	775049	phrB	1.241	0.694	0	0
8396	775193	phrB	1.740	0.839	0	0
2530	775616	phrB	1.724	0.869	0	0
10223	775826	phrB	0.697	0.536	0	0
15888	776096	phrB	1.202	0.603	0	0
21145	776262	ybgH	1.127	0.809	0	0

19488	776463	ybgH	1.629	0.925	0	0
20902	776794	ybgH	1.683	0.777	0	0
781	776992	ybgH	1.217	0.681	0	0
18676	777136	ybgH	1.066	0.717	0	0
1097	777443	ybgH	1.059	0.759	0	0
1762	777573	ybgH, ybgI	1.415	0.800	0	0
8306	777942	ybgH, ybgI	0.821	0.672	0	0
1591	778018	ybgH, ybgI	1.048	0.801	0	0
3663	778107	ybgH, ybgI	1.201	0.802	0	0
12670	778280	ybgI	1.071	1.004	0	0
10513	778722	ybgI, ybgJ	1.353	1.084	0	0
4298	779136	ybgJ, ybgK	1.042	0.931	0	0
572	779349	ybgJ, ybgK	1.502	1.181	0	0
13724	779427	ybgJ, ybgK	1.350	1.007	0	0
10729	779614	ybgK	1.386	1.072	0	0
3018	779832	ybgK	1.235	1.058	0	0
16018	780060	ybgK, ybgL	1.235	1.065	0	0
12748	780346	ybgK, ybgL	1.326	1.111	0	0
20385	780419	ybgL	0.803	1.002	0	0
7903	780723	ybgL	1.789	1.141	0	0
868	781078	ybgL, STM0715	3.213	1.048	1	0
755	781361	STM0715, STM0716	3.119	0.774	1	0
6693	781441	STM0715, STM0716	2.840	0.621	1	0
10159	781817	STM0716	1.664	0.956	0	0
21874	782061	STM0716	1.142	0.980	0	0
19203	782229	STM0717	1.173	0.724	0	0
17498	782326	STM0717	1.202	0.764	0	0
3236	782680	STM0717, STM0718	0.894	0.520	0	0
4149	782954	STM0717, STM0718	1.213	0.468	0	0
3492	783192	STM0718	1.345	0.553	0	0
8871	783346	STM0718	1.439	0.567	0	0
3206	783593	STM0718, STM0719	2.708	0.538	1	0
10410	783826	STM0719	4.151	0.487	1	0
18583	784105	STM0719	3.629	0.420	1	0
17551	784364	STM0719	4.089	0.457	1	0
16509	784581	STM0719	2.803	0.529	1	0

5853	784828	STM0719, STM0720	2.846	0.415	1	0
7417	784907	STM0719, STM0720	2.628	0.437	1	0
17800	785184	STM0720	2.875	0.642	1	0
11912	785284	STM0720	2.390	0.486	0	0
15170	785607	STM0720, STM0721	2.196	0.488	0	0
1300	785708	STM0720, STM0721	1.982	0.449	0	0
13011	786077	STM0721	2.248	0.479	0	0
15671	786216	STM0721	2.242	0.496	1	0
10570	786547	STM0721	2.550	0.543	1	0
9318	786817	STM0721	2.648	0.500	1	0
12675	786894	STM0721, STM0722	2.764	0.498	1	0
18176	787299	STM0722	2.016	0.418	0	0
21663	787495	STM0722	2.341	0.559	0	0
5723	787675	STM0722, STM0723	2.162	0.529	0	0
15845	787847	STM0722, STM0723	2.455	0.437	0	0
12168	788059	STM0722, STM0723	2.268	0.508	1	0
3365	788170	STM0723	2.475	0.518	1	0
2098	788561	STM0723, STM0724	1.755	0.545	0	0
12567	788751	STM0723, STM0724	2.296	0.510	0	0
20973	789053	STM0724	2.165	0.504	0	0
19238	789158	STM0724	1.775	0.350	0	0
19209	789545	STM0724	2.247	0.494	0	0
1412	789648	STM0724	2.255	0.501	0	0
11955	789793	STM0724	2.497	0.559	0	0
985	790166	STM0724	2.017	0.537	0	0
1719	790308	STM0724	1.649	0.539	0	0
22265	790416	STM0724, STM0725	2.089	0.512	0	0
4736	790866	STM0725	2.030	0.437	0	0
1679	791001	STM0725	2.115	0.490	0	0
14403	791171	STM0725	2.523	0.448	0	0
14394	791430	STM0725, STM0726	2.153	0.439	0	0
2036	791624	STM0725, STM0726	1.691	0.421	0	0
15932	791743	STM0726	2.119	0.472	0	0
19794	792091	STM0726	2.477	0.529	0	0
14626	792382	STM0726	2.046	0.387	0	0
9914	792740	STM0726	2.362	0.430	0	0

2411	792838	STM0726	2.948	0.597	1	0
18563	793208	STM0726	3.339	0.429	1	0
11746	793412	STM0726, STM0727	3.067	0.491	1	0
16957	793520	STM0726, STM0727	4.012	0.618	1	0
16262	793651	STM0727, nei	3.360	0.558	1	0
10345	793847	nei	2.501	0.647	1	0
7469	793986	nei	1.856	0.642	0	0
11482	794196	nei	1.593	0.923	0	0
12450	794440	nei	1.225	1.095	0	0
16388	794927	abrB	0.790	0.877	0	0
6872	795054	abrB	0.607	0.748	0	0
10269	795244	abrB	1.034	1.018	0	0
17171	795586	abrB	0.802	1.212	0	0
5855	795738	abrB	1.100	1.555	0	0
12093	795812	abrB	0.882	1.616	0	0
1597	796302	gltA	0.536	1.804	0	0
10111	796589	gltA	0.668	2.360	0	1
2477	796660	gltA	0.539	2.248	0	1
22031	797057	STM0731, gltA	0.606	1.745	0	0
20092	797153	STM0731, gltA	0.409	1.927	0	1
21659	797316	STM0731, gltA	0.406	2.103	0	1
971	797467	STM0731, gltA	0.345	1.612	0	0
8928	797924	sdhC, sdhD	0.497	2.395	0	1
8208	798007	sdhC, sdhD	0.393	2.411	0	1
12425	798245	sdhC, sdhD	0.416	2.825	0	1
17805	798320	sdhC, sdhD	0.369	3.086	0	1
7205	798411	sdhD, sdhA	0.339	2.548	0	1
4305	798951	sdhA	0.547	2.915	0	1
2669	799153	sdhA	0.295	1.292	0	0
14661	799559	sdhA	0.465	2.964	0	1
6999	799711	sdhA	0.344	2.437	0	1
17590	799782	sdhA	0.520	4.031	0	1
14313	800229	sdhA, sdhB	0.416	1.915	0	1
16368	800362	sdhA, sdhB	0.434	2.480	0	1
3576	800681	sdhB	0.428	2.703	0	1
372	800805	sdhB	0.736	3.469	0	1

20099	801037	sdhB	0.507	3.129	0	1
17898	801322	sucA	0.550	2.594	0	1
13009	801450	sucA	0.460	2.615	0	1
13924	801757	sucA	0.479	2.508	0	1
6182	801962	sucA	0.437	2.902	0	1
5511	802107	sucA	0.464	3.017	0	1
14391	802528	sucA	0.503	2.826	0	1
18304	802599	sucA	0.453	2.432	0	1
4443	802888	sucA	0.505	2.551	0	1
10126	803055	sucA	0.442	2.085	0	1
8713	803346	sucA	0.620	2.868	0	1
16569	803671	sucA	0.440	2.111	0	1
21379	803742	sucA	0.543	2.982	0	1
10491	804105	sucA, sucB	0.540	2.548	0	1
13649	804176	sucA, sucB	0.553	2.240	0	1
3787	804494	sucA, sucB	0.599	2.461	0	1
6363	804649	sucB	0.772	2.426	0	1
21237	804724	sucB	0.499	2.212	0	1
15572	805154	sucB	0.557	2.701	0	1
20401	805406	sucB	0.397	1.205	0	0
15895	805683	sucB, sucC	0.388	1.452	0	0
2021	805897	sucC	0.386	1.330	0	0
15705	806148	sucC	0.637	2.520	0	0
4194	806379	sucC	0.524	1.776	0	0
5438	806491	sucC	0.631	2.483	0	1
10938	806814	sucC, sucD	0.639	2.626	0	1
4133	807040	sucC, sucD	0.702	1.989	0	0
13205	807299	sucD	0.753	2.235	0	1
16141	807470	sucD	0.799	2.362	0	1
17522	807631	sucD	1.238	1.902	0	1
7222	807937		2.276	0.983	1	0
13644	808010		2.184	0.837	1	0
1271	808292		2.662	0.609	1	0
13726	808625		2.982	0.540	1	0
10325	808752		3.228	0.591	1	0
17079	809032	cydA	1.986	0.624	0	0

9537	809201	cydA	2.076	0.519	0	0
17312	809486	cydA	2.000	0.844	0	0
4580	809647	cydA	1.450	0.793	0	0
17134	809934	cydA	0.977	0.970	0	0
14159	810138	cydA	0.793	0.893	0	0
12971	810446	cydA	0.979	1.027	0	0
6531	810527	cydA	0.860	1.036	0	0
19540	810733	cydA, cydB	1.036	1.250	0	0
20291	811088	cydB	0.974	1.237	0	0
2531	811222	cydB	0.899	1.128	0	0
8922	811544	cydB	0.905	1.461	0	0
15071	811770	cydB, ybgT	0.598	0.850	0	0
17847	812032	cydB, ybgT	0.871	1.237	0	0
17199	812237	ybgT, ybgE	0.786	1.008	0	0
21988	812341	ybgT, ybgE	0.962	1.067	0	0
3193	812752	ybgE, ybgC	0.941	1.304	0	0
13085	812830	ybgC	0.713	1.242	0	0
1166	812928	ybgC, tolQ	0.959	1.550	0	0
19156	813415	tolQ	0.733	1.497	0	0
4199	813493	tolQ	0.522	1.120	0	0
7359	813797	tolQ, tolR	0.704	1.592	0	0
14319	814022	tolQ, tolR	0.752	1.672	0	0
15128	814318	tolR, tolA	0.731	1.438	0	0
20568	814389	tolR, tolA	0.882	1.672	0	0
4741	814681	tolA	0.843	1.309	0	0
8889	814922	tolA	0.751	1.177	0	0
11452	815093	tolA	1.118	1.812	0	0
15161	815442	tolA	1.134	1.926	0	0
10191	815654	tolA, tolB	0.951	2.089	0	0
18032	815765	tolA, tolB	0.883	2.013	0	1
835	815946	tolB	0.709	1.774	0	1
21350	816342	tolB	0.607	1.792	0	0
3773	816488	tolB	0.500	1.696	0	0
7531	816727	tolB	0.613	1.958	0	1
16199	816960	tolB, pal	0.648	2.257	0	1
6413	817123	tolB, pal	0.558	1.917	0	1

18856	817485	pal, ybgF	0.333	0.952	0	0
9263	817677	pal, ybgF	0.562	1.768	0	0
8655	817795	ybgF	0.567	2.121	0	1
5301	818019	ybgF	0.653	2.501	0	1
2726	818304	ybgF, lysT	0.619	2.199	0	1
18106	818501	ybgF, lysT	0.523	2.318	0	1
7696	818665	lysT, valT	0.429	3.481	0	1
8934	819132	lysY, lysZ	0.370	4.120	0	1
11424	819256	lysZ	0.499	3.454	0	1
7520	819362	lysZ	0.412	3.679	0	1
2769	819776	nadA	0.569	3.355	0	1
7223	819936	nadA	0.783	0.870	0	0
19817	820197	nadA	1.065	1.009	0	0
2235	820291	nadA	1.222	0.961	0	0
9266	820689	nadA	0.487	0.402	0	0
12023	820824	nadA, pnuC	1.216	0.798	0	0
7241	821016	nadA, pnuC	1.222	0.932	0	0
18160	821257	pnuC	1.155	0.909	0	0
20382	821419	pnuC	1.387	0.829	0	0
3163	821836	pnuC	0.848	0.950	0	0
9627	822068	ybgR	1.077	0.893	0	0
5364	822286	ybgR	1.204	0.938	0	0
8136	822499	ybgR	1.136	0.799	0	0
19053	822622	ybgR	1.049	0.683	0	0
18865	822950	ybgR	1.476	0.717	0	0
16649	823167	ybgR, ybgS	1.432	0.791	0	0
7042	823406	ybgS, aroG	1.398	0.807	0	0
17433	823480	ybgS, aroG	1.601	0.950	0	0
11053	823586	ybgS, aroG	1.489	0.899	0	0
935	823696	ybgS, aroG	2.334	0.825	0	0
3877	823769	ybgS, aroG	1.694	1.030	0	0
12348	824555	aroG	1.224	0.724	0	0
11950	824648	aroG	1.498	0.656	0	0
15577	824923	STM0761	2.323	0.661	1	0
13680	825159	STM0761	2.618	0.626	1	0
4408	825279	STM0761	2.559	0.730	0	0

8593	825631	STM0761, STM0762	2.961	0.593	1	0
10246	825779	STM0761, STM0762	2.290	0.569	1	0
5237	826063	STM0762, STM0763	2.036	0.445	0	0
20807	826314	STM0762, STM0763	1.930	0.442	0	0
1025	826497	STM0762, STM0763	2.149	0.513	0	0
3348	826738	STM0762, STM0763	1.870	0.523	0	0
7474	826942	STM0763	1.890	0.477	0	0
17159	827178	STM0763	2.065	0.471	0	0
805	827346	STM0764	2.165	0.535	0	0
3641	827611	STM0764	2.414	0.487	0	0
17153	827981	STM0764	2.375	0.548	0	0
4454	828195	STM0764, STM0765	2.018	0.449	0	0
13819	828366	STM0764, STM0765	1.682	0.437	0	0
3098	828639	STM0764, STM0765	2.427	0.388	0	0
11052	828843	STM0765	2.773	0.406	1	0
1338	829061	STM0765	3.345	0.520	1	0
17610	829132	STM0765	3.605	0.553	1	0
6997	829509	STM0765, dcoC	4.837	0.457	1	0
20593	829625	STM0765, dcoC	4.305	0.560	1	0
3353	829739	STM0765, dcoC	4.010	0.563	1	0
7790	830082	dcoC	1.174	0.677	0	0
20117	830198		2.341	0.811	0	0
16596	830658		1.877	0.578	0	0
19485	830863		1.057	0.611	0	0
17509	830944		1.173	0.654	0	0
21180	831183		2.193	0.712	0	0
18247	831530	dcoB	0.736	0.609	0	0
5951	831777	dcoB	2.420	0.770	0	0
14236	831885	dcoB	1.952	0.728	0	0
5860	832192	dcoB	2.023	0.822	0	0
3830	832423	dcoB	1.533	0.755	0	0
8250	832698	dcoB	1.346	0.679	0	0
18353	832989	dcoB, STM0769	0.959	0.590	0	0
9120	833176	dcoB, STM0769	1.133	0.656	0	0
10617	833375	STM0769	0.916	0.532	0	0
19375	833649	STM0769	0.505	0.365	0	0

6864	833899	STM0769	1.710	0.948	0	0
2008	834115	STM0770	2.026	0.721	0	0
3949	834369	STM0770	2.615	0.766	1	0
5739	834599	STM0770	2.187	0.708	1	0
15954	834684	STM0770	1.163	0.390	0	0
20178	834859	STM0770	1.783	0.845	0	0
996	835069	STM0770, STM0771	0.884	0.555	0	0
12732	835449	STM0770, STM0771	1.225	0.798	0	0
12409	835679	STM0771	1.526	1.189	0	0
9393	835840	STM0771	1.162	1.020	0	0
20180	836076	STM0771	0.944	1.302	0	0
16007	836179	STM0771	0.985	1.877	0	1
3917	836486	gpmA	0.947	1.950	0	1
15749	836674	gpmA	0.978	1.969	0	1
19124	836934	gpmA	0.839	1.475	0	0
2480	837218	gpmA	0.607	0.922	0	0
11514	837312	gpmA, galM	0.794	1.019	0	0
21315	837685	galM	1.334	1.244	0	0
19923	837835	galM	1.125	0.987	0	0
15702	838095	galM	1.463	1.328	0	0
7239	838388	galM, galK	1.147	1.365	0	0
4238	838552	galM, galK	1.742	1.138	0	0
227	838875	galK	0.911	1.139	0	0
21409	838949	galK	1.414	1.268	0	0
4465	839355	galK	0.948	0.809	0	0
17275	839499	galK, galT	1.393	1.115	0	0
22058	839736	galK, galT	0.672	0.568	0	0
9424	839820	galK, galT	1.157	1.031	0	0
11701	840000	galT	1.308	1.135	0	0
22194	840334	galT	1.796	1.235	0	0
5313	840525	galT	1.313	1.001	0	0
4113	840814	galT, galE	1.088	1.132	0	0
20027	841101	galE	0.952	1.014	0	0
20197	841366	galE	1.035	1.045	0	0
21062	841550	galE	0.710	0.755	0	0
9200	841720	galE	1.094	0.688	0	0

10665	841883	galE, STM0777	1.181	0.657	0	0
7826	842030	galE, STM0777	1.968	0.554	0	0
15622	842263	STM0777	2.750	0.570	1	0
2323	842707	STM0777	4.095	0.611	1	0
14811	842899	STM0777, modF	3.912	0.627	1	0
22188	842990	STM0777, modF	3.403	0.675	1	0
13032	843264	modF	1.857	0.944	0	0
13258	843534	modF	0.848	1.248	0	0
19567	843882	modF	0.842	1.697	0	0
6054	844104	modF	0.931	1.679	0	0
15186	844317	modF	0.917	1.414	0	0
1425	844429	modF, modE	0.996	1.597	0	0
21670	844660	modF, modE	0.558	0.887	0	0
9806	844919	modE, STM0780	1.030	1.263	0	0
10107	845206	modE, STM0780	1.205	2.103	0	1
18540	845303	modE, STM0780	0.781	2.048	0	1
13660	845553	modE, modA	0.771	1.755	0	1
20827	845813	modA	0.489	1.071	0	0
19301	845902	modA	1.086	1.799	0	0
7897	846304	modA, modB	0.805	1.454	0	0
7106	846411	modA, modB	1.027	1.726	0	0
14825	846661	modB, modC	0.855	1.700	0	0
17947	847043	modB, modC	0.519	0.908	0	0
21047	847133	modC	1.014	1.591	0	0
7315	847333	modC	1.214	1.666	0	0
17510	847663	modC	0.945	1.695	0	0
4376	847916	modC	0.872	1.826	0	0
18427	848029	modC	0.821	1.648	0	0
965	848327	ybhA	0.707	1.756	0	0
14705	848674	ybhA	0.970	1.298	0	0
21701	848785	ybhA, ybhE	0.911	1.134	0	0
723	849133	ybhA, ybhE	1.256	1.095	0	0
3652	849276	ybhA, ybhE	0.710	0.742	0	0
9759	849429	ybhA, ybhE	1.035	0.992	0	0
317	849716	ybhE	1.279	1.059	0	0
21353	849930	ybhE	1.077	1.017	0	0

17195	850014	ybhE	1.381	1.248	0	0
6296	850386	ybhC	0.815	1.303	0	0
16973	850661	ybhC	0.589	0.803	0	0
11377	850833	ybhC	0.855	1.167	0	0
633	851035	ybhC	1.014	1.270	0	0
7190	851121	ybhC	0.833	1.169	0	0
1796	851577	ybhC, hutI	1.183	1.826	0	1
3254	851774	ybhC, hutI	1.061	1.756	0	1
10505	852004	ybhC, hutI	1.132	1.676	0	0
8370	852248	hutI	1.108	1.178	0	0
11968	852430	hutI	1.056	1.217	0	0
9807	852602	hutI, hutG	1.416	1.384	0	0
9952	852985	hutI, hutG	1.405	1.569	0	0
2771	853158	hutG	1.342	1.413	0	0
1432	853288	hutG	0.515	0.641	0	0
12342	853494	hutG	1.455	1.652	0	0
17379	853753	hutG, hutC	1.171	1.265	0	0
6001	853999	hutC	1.342	1.368	0	0
2515	854150	hutC	1.170	1.161	0	0
1941	854413	hutC	1.188	1.470	0	0
8575	854635	hutC	1.034	1.592	0	0
6602	854933		1.135	1.383	0	0
6783	855060		1.098	1.150	0	0
607	855367		1.298	1.020	0	0
3741	855544		0.792	0.861	0	0
3124	855918		1.002	0.969	0	0
16324	856092		1.179	1.107	0	0
3341	856189	hutH	0.825	0.779	0	0
7104	856570	hutH	1.320	1.149	0	0
17932	856651	hutH	0.442	0.603	0	0
16541	856847	hutH	1.076	0.917	0	0
6058	856947	hutH	1.672	1.170	0	0
2752	857452	hutH	0.916	0.706	0	0
4641	857721	hutH	1.622	0.878	0	0
22134	857986	hutH	0.856	0.939	0	0
2288	858134	hutH	0.803	0.873	0	0

8096	858336	ybhB	1.259	1.327	0	0
18634	858673	ybhB	1.209	0.921	0	0
4765	858768	ybhB, bioA	1.123	0.796	0	0
12543	858843	ybhB, bioA	0.836	0.689	0	0
14711	859360	bioA	1.524	0.810	0	0
2271	859574	bioA	0.989	0.528	0	0
19087	859812	bioA, bioB	1.361	0.649	0	0
17654	859889	bioA, bioB	1.280	0.771	0	0
17554	860189	bioA, bioB	1.470	0.695	0	0
20337	860379	bioA, bioB	1.241	0.684	0	0
22359	860609	bioB	0.610	0.661	0	0
11634	860857	bioB, bioF	1.261	0.723	0	0
15805	860988	bioB, bioF	1.312	0.771	0	0
11581	861320	bioF	1.597	0.820	0	0
8366	861549	bioF	1.321	0.617	0	0
2320	861642	bioF	1.616	0.705	0	0
13083	861950	bioF, bioC	1.526	0.774	0	0
18366	862108	bioF, bioC	1.092	0.722	0	0
5554	862308	bioF, bioC	0.711	0.482	0	0
20576	862605	bioC, bioD	1.903	0.932	0	0
4476	862677	bioC, bioD	0.369	0.382	0	0
14816	863078	bioD	1.474	0.605	0	0
8056	863294	bioD	0.641	0.538	0	0
20759	863454	bioD	0.783	0.602	0	0
10208	863888		1.425	0.797	0	0
15686	864019	uvrB	1.580	0.963	0	0
21876	864154	uvrB	0.414	0.399	0	0
5207	864295	uvrB	0.924	1.130	0	0
16468	864730	uvrB	1.054	1.055	0	0
20232	864879	uvrB	1.013	1.020	0	0
5249	865127	uvrB	0.971	1.000	0	0
2884	865302	uvrB	0.972	1.117	0	0
15472	865549	uvrB	1.532	1.431	0	0
919	865886	uvrB	0.775	0.713	0	0
6656	866148	uvrB	1.133	0.843	0	0
11927	866334	uvrB, STM0799	1.787	0.867	0	0

10870	866563	STM0799, slrP	2.059	0.566	0	0
18182	866830	STM0799, slrP	2.588	0.483	1	0
17835	867053	slrP	2.727	0.491	1	0
5322	867239	slrP	2.606	0.638	1	0
15713	867458	slrP	2.222	0.732	0	0
22007	867652	slrP	2.247	0.727	0	0
14969	867774	slrP	1.774	1.063	0	0
18886	868154	slrP	1.380	1.319	0	0
5445	868435	slrP	1.155	1.007	0	0
18687	868530	slrP	1.218	0.922	0	0
299	868855	slrP	1.470	0.674	0	0
17327	869088	slrP	0.994	0.802	0	0
4318	869236		1.088	0.708	0	0
11121	869486	ybhK	1.021	0.990	0	0
421	869763	ybhK	0.972	1.113	0	0
1583	869918	ybhK	1.209	0.902	0	0
11042	870204	ybhK, moaA	0.721	0.683	0	0
8603	870385	ybhK, moaA	1.222	0.956	0	0
4832	870674	ybhK, moaA	0.816	0.985	0	0
19839	870857	moaA	0.854	1.195	0	0
17869	871142	moaA	0.871	1.295	0	0
9793	871233	moaA, moaB	1.114	1.187	0	0
8702	871562	moaA, moaB	1.206	0.984	0	0
21837	871838	moaB, moaC	0.837	0.907	0	0
19932	871930	moaB, moaC	1.081	0.995	0	0
17170	872108	moaB, moaC	0.889	0.834	0	0
13839	872529	moaC, moaD	1.274	1.147	0	0
21687	872612	moaC, moaD	0.943	1.124	0	0
15974	872697	moaD, moaE	1.337	1.268	0	0
11062	873218	moaE, ybhL	1.205	1.087	0	0
20651	873346	ybhL	1.142	1.215	0	0
10741	873668	ybhL	1.464	1.094	0	0
8912	873785	ybhL, ybhM	2.306	1.010	0	0
10643	874029	ybhL, ybhM	2.881	0.585	1	0
5865	874192	ybhM	3.363	0.566	1	0
2802	874375	ybhM	2.787	0.590	1	0

18421	874734	ybhM, STM0809	2.885	0.522	1	0
14672	874924	STM0809	2.440	0.675	1	0
14086	875189	STM0809, STM0810	3.521	0.524	1	0
20980	875379	STM0809, STM0810	3.734	0.518	1	0
3454	875544	STM0809, STM0810	2.557	0.560	0	0
20729	875758	STM0810	2.403	0.618	0	0
11974	876020	STM0810	2.063	0.582	0	0
5054	876105	STM0810	2.216	0.617	0	0
1553	876564	ybhN	1.592	0.966	0	0
6658	876692	ybhN	1.426	1.082	0	0
21664	877005	ybhN	0.925	0.913	0	0
18918	877108	ybhN, ybhO	1.230	0.949	0	0
21075	877357	ybhN, ybhO	1.193	0.655	0	0
18919	877677	ybhO	1.172	0.726	0	0
6688	877975	ybhO	1.286	0.753	0	0
19579	878187	ybhO	1.344	0.615	0	0
5996	878270	ybhO	1.047	0.608	0	0
13512	878345	ybhO, ybhP	0.790	0.564	0	0
15943	878779	ybhO, ybhP	1.226	0.920	0	0
6406	879016	ybhP, ybhQ	0.947	1.323	0	0
22156	879184	ybhP, ybhQ	1.033	1.779	0	0
15167	879275	ybhP, ybhQ	0.699	1.728	0	0
12131	879681	ybhR	0.922	1.566	0	0
2379	879893	ybhR	0.874	1.419	0	0
13406	880197	ybhR	0.959	0.911	0	0
11332	880445	ybhR	1.074	0.918	0	0
11655	880667	ybhR	1.179	0.972	0	0
7872	880812	ybhR, ybhS	0.990	0.833	0	0
11031	881125	ybhR, ybhS	1.018	0.812	0	0
6403	881251	ybhS	0.967	0.849	0	0
11891	881374	ybhS	1.372	1.062	0	0
3722	881679	ybhS	1.477	1.013	0	0
13818	881951	ybhS, ybhF	1.342	1.018	0	0
13873	882144	ybhS, ybhF	1.229	0.875	0	0
10263	882428	ybhF	1.257	0.877	0	0
21596	882635	ybhF	1.297	1.010	0	0

8808	882939	ybhF	1.345	1.002	0	0
7109	883073	ybhF	1.268	0.974	0	0
8384	883379	ybhF	1.376	1.001	0	0
16274	883634	ybhF	1.354	1.194	0	0
473	883848	ybhF, STM0818	1.636	1.483	0	0
17911	884139	STM0818	1.326	1.501	0	0
13987	884254	STM0818	1.249	1.102	0	0
20984	884560	STM0818	0.910	0.954	0	0
8402	884713	STM0818, ybiH	1.118	0.877	0	0
20486	884902	STM0818, ybiH	1.224	0.963	0	0
7091	885173	ybiH, rhlE	1.255	1.041	0	0
19142	885394	ybiH, rhlE	1.009	1.043	0	0
19077	885494	ybiH, rhlE	1.183	1.087	0	0
14488	885862	rhlE	0.757	0.797	0	0
16540	885993	rhlE	1.391	0.968	0	0
14215	886259	rhlE	1.575	0.923	0	0
6517	886527	rhlE	1.845	1.012	0	0
12488	886625	rhlE	0.768	0.450	0	0
7179	887069	dinG	0.885	0.842	0	0
21210	887256	dinG	1.028	0.791	0	0
4222	887377	dinG	1.181	0.854	0	0
7221	887713	dinG	1.302	0.936	0	0
11324	887968	dinG	1.173	0.876	0	0
18703	888081	dinG	1.262	0.952	0	0
1476	888442	dinG	1.290	0.953	0	0
3282	888651	dinG	1.337	0.907	0	0
13206	888724	dinG	1.293	0.990	0	0
7746	889033	dinG, ybiB	1.157	1.029	0	0
15928	889302	ybiB	1.127	1.154	0	0
13388	889495	ybiB	1.259	1.292	0	0
4180	889701	ybiB	1.192	0.972	0	0
8738	889918	ybiB	0.821	0.791	0	0
8342	890098	ybiB	0.766	0.722	0	0
5042	890386		1.307	0.937	0	0
7412	890674	ybiJ	1.049	0.904	0	0
10252	890876	ybiJ	1.119	0.994	0	0

16590	891046	ybiJ, ybiI	0.822	0.962	0	0
2114	891209	ybiI, ybiN	0.852	0.939	0	0
6317	891367	ybiI, ybiN	1.046	0.946	0	0
16170	891819	ybiN	1.490	0.904	0	0
1012	891992	ybiN	0.889	0.609	0	0
20814	892172	ybiN	0.498	0.615	0	0
9689	892499	ybiO	0.592	0.607	0	0
12856	892756	ybiO	1.379	0.807	0	0
8906	892935	ybiO	1.316	0.800	0	0
3749	893028	ybiO	1.158	0.835	0	0
18228	893189	ybiO	1.463	0.914	0	0
15803	893505	ybiO	1.392	0.816	0	0
16670	893769	ybiO	1.015	0.631	0	0
19982	893963	ybiO	1.578	0.764	0	0
12949	894194	ybiO	1.327	0.862	0	0
15762	894587	ybiO	0.811	0.974	0	0
8549	894723	ybiO, glnQ	0.762	0.952	0	0
20371	894813	ybiO, glnQ	1.304	1.517	0	0
12215	895085	glnQ	0.656	1.166	0	0
1179	895238	glnQ	0.984	1.384	0	0
18317	895609	glnQ, glnP	0.959	1.147	0	0
11709	895891	glnQ, glnP	1.083	1.420	0	0
10094	896102	glnP	0.783	1.260	0	0
20865	896266	glnP, glnH	0.808	1.167	0	0
1003	896606	glnH	0.849	1.073	0	0
17745	896751	glnH	0.810	1.076	0	0
1197	896958	glnH	0.848	1.030	0	0
8947	897086	glnH	0.908	1.072	0	0
1540	897168	glnH	0.965	0.956	0	0
7442	897546	dps	1.060	0.827	0	0
8789	897916	dps	2.470	0.563	1	0
10463	897992	dps	2.798	0.479	1	0
11382	898291	dps, ybiF	2.848	0.616	1	0
11118	898495	ybiF	2.332	0.762	1	0
10455	898738	ybiF	0.808	0.590	0	0
9015	899103	ybiF, ompX	1.066	1.061	0	0

17723	899180	ybiF, ompX	0.907	0.971	0	0
2721	899365	ybiF, ompX	1.100	1.398	0	0
8519	899505	ybiF, ompX	0.796	1.435	0	0
21454	899865	ompX	0.847	1.870	0	0
16616	900225	ybiP	1.339	1.114	0	0
2128	900297	ybiP	1.370	1.033	0	0
14586	900486	ybiP	1.123	0.829	0	0
1685	900834	ybiP	1.102	0.850	0	0
5870	901026	ybiP	0.966	0.809	0	0
7045	901332	ybiP	1.000	0.896	0	0
19431	901564	ybiP	1.009	1.348	0	0
2622	901661	ybiP	1.038	1.888	0	0
9943	901998	ybiP, STM0835	0.654	1.392	0	0
18050	902290	STM0835, ybiR	0.944	1.612	0	0
3136	902371	STM0835, ybiR	0.805	1.357	0	0
17103	902597	STM0835, ybiR	1.037	1.304	0	0
2210	902773	ybiR	0.616	0.867	0	0
1894	902854	ybiR	0.490	0.644	0	0
19797	903158	ybiR	1.079	1.396	0	0
14004	903605	ybiR	0.990	1.589	0	0
11111	903815	ybiR	0.957	1.273	0	0
790	903941	ybiS	0.878	1.987	0	0
11584	904369	ybiS	0.925	1.638	0	0
12531	904455	ybiS	0.841	1.379	0	0
18987	904812	ybiS, ybiT	0.762	1.728	0	0
6258	904909	ybiS, ybiT	0.705	1.649	0	0
3138	905004	ybiS, ybiT	0.756	2.111	0	0
12740	905242	ybiS, ybiT	0.685	1.244	0	0
11590	905346	ybiT	1.129	1.479	0	0
2341	905793	ybiT	1.077	0.824	0	0
6800	906008	ybiT	1.117	0.789	0	0
12326	906201	ybiT	1.011	0.714	0	0
6190	906587	ybiT	1.812	0.778	0	0
17272	906671		2.169	0.803	0	0
11257	907053	STM0839	2.667	0.694	1	0
583	907286	STM0839	3.299	0.672	1	0

7519	907466	STM0839	2.834	0.616	1	0
13394	907669	STM0839	2.491	0.600	0	0
8643	907740	STM0839	1.102	0.441	0	0
8809	908069	ybiV(2)	2.415	0.598	0	0
4279	908371	ybiV(2)	2.176	0.658	0	0
14666	908626	ybiV(2)	2.010	1.200	0	0
21628	908756	ybiV(2)	1.810	1.140	0	0
10434	908923	ybiV(2), ybiU	1.304	1.454	0	0
18095	909074	ybiV(2), ybiU	1.244	1.468	0	0
1116	909415	ybiU	1.106	1.136	0	0
15767	909812	ybiU	1.379	0.863	0	0
12062	909883	ybiU	0.827	0.582	0	0
6322	910171	ybiU, ybiV(1)	1.234	0.845	0	0
12120	910418	ybiU, ybiV(1)	0.535	0.648	0	0
13369	910600	ybiU, ybiV(1)	1.214	1.273	0	0
20095	910855	ybiV(1)	0.917	1.103	0	0
11026	911002	ybiV(1)	1.080	1.269	0	0
15052	911162	ybiV(1)	1.151	1.181	0	0
15109	911508	pflF	0.908	0.974	0	0
4676	911825	pflF	1.222	0.802	0	0
4470	912003	pflF	0.624	0.488	0	0
499	912175	pflF	1.633	0.823	0	0
19068	912487	pflF	1.540	0.767	0	0
7265	912667	pflF	1.182	0.675	0	0
9823	912926	pflF	1.304	0.685	0	0
2892	913020	pflF	1.243	0.860	0	0
1699	913315	pflF	1.176	0.681	0	0
5694	913536	pflF	1.337	0.818	0	0
7172	913757	pflF, pflE	0.916	0.589	0	0
12381	914129	pflF, pflE	1.099	0.572	0	0
18552	914320	pflE	1.270	0.869	0	0
20682	914581	pflE	0.779	0.649	0	0
9832	914749	pflE	1.291	0.924	0	0
14693	914860	pflE	1.194	0.986	0	0
11563	915191	moeB	1.240	1.171	0	0
5958	915273	moeB	1.097	1.095	0	0

4035	915474	moeB	1.459	1.111	0	0
3753	915785	moeB, moeA	1.327	1.201	0	0
12565	916109	moeB, moeA	1.019	0.962	0	0
2265	916296	moeA	1.098	0.958	0	0
2397	916578	moeA	0.766	1.116	0	0
3204	916760	moeA, ybiK	1.022	0.962	0	0
8974	916940	moeA, ybiK	1.293	1.723	0	0
15701	917116	moeA, ybiK	1.001	1.175	0	0
6254	917201	moeA, ybiK	0.831	1.147	0	0
18792	917367	moeA, ybiK	0.910	1.774	0	0
9888	917975	ybiK, yliA	1.526	1.990	0	0
18102	918130	yliA	0.932	1.328	0	0
14258	918445	yliA	1.269	1.823	0	0
1999	918537	yliA	1.246	1.510	0	0
10627	918718	yliA	0.702	0.761	0	0
3102	919083	yliA	1.256	1.231	0	0
243	919351	yliA	1.362	1.306	0	0
12461	919598	yliA, yliB	1.212	1.176	0	0
18789	919816	yliA, yliB	1.052	0.975	0	0
13442	919940	yliA, yliB	0.595	0.673	0	0
12580	920196	yliB	1.377	1.253	0	0
13650	920350	yliB	1.443	1.259	0	0
4845	920559	yliB	1.439	1.083	0	0
16445	920793	yliB	1.172	1.053	0	0
6137	921054	yliB	0.801	0.782	0	0
19538	921356	yliB, yliC	1.140	1.157	0	0
2982	921441	yliB, yliC	0.864	1.058	0	0
11159	921629	yliC	0.532	0.958	0	0
2673	922019	yliC	0.967	1.240	0	0
5088	922297	yliC, yliD	1.109	1.130	0	0
17701	922409	yliC, yliD	0.789	0.848	0	0
14725	922720	yliD	1.047	1.253	0	0
951	922901	yliD	0.630	0.735	0	0
1142	923151	yliD	1.496	1.501	0	0
13879	923351	yliD	0.637	0.762	0	0
1646	923673	yliG	0.485	0.698	0	0

14263	923885	yliG	0.786	0.678	0	0
20549	924100	yliG	0.604	0.643	0	0
17157	924171	yliG	0.491	0.657	0	0
14010	924473	yliG	0.834	0.671	0	0
8382	924809	yliG, yliH	0.920	0.955	0	0
1316	924883	yliG, yliH	0.632	0.738	0	0
938	925073	yliG, yliH	0.978	0.900	0	0
11512	925393	yliH	1.165	0.694	0	0
8512	925647		1.443	0.535	0	0
10413	925914	STM0854	2.548	0.507	0	0
21714	926149	STM0854, STM0855	2.416	0.480	0	0
15560	926318	STM0854, STM0855	2.975	0.544	1	0
15119	926518	STM0854, STM0855	2.721	0.524	1	0
6087	926801	STM0855	2.349	0.491	0	0
2106	926881	STM0855	1.832	0.635	0	0
6263	927229	STM0855, STM0856	2.541	0.470	0	0
5230	927498	STM0856	2.416	0.386	0	0
13659	927671	STM0856	2.396	0.448	0	0
2131	927889	STM0856	2.095	0.430	0	0
21525	928100	STM0856	2.278	0.566	1	0
19529	928345	STM0856, STM0857	2.508	0.437	1	0
10639	928635	STM0857	2.141	0.547	0	0
17901	928886	STM0857	1.161	0.413	0	0
5348	929135	STM0857	1.901	0.567	0	0
16464	929237	STM0857	1.959	0.604	0	0
9849	929328	STM0857	1.824	0.559	0	0
13157	929738	STM0857	2.044	0.520	0	0
17525	929908	STM0858	3.144	0.542	1	0
12619	930020	STM0858	3.385	0.494	1	0
21443	930439	STM0858	4.098	0.590	1	0
1074	930651	STM0858	2.887	0.602	1	0
17120	930793	STM0858	2.819	0.617	1	0
16349	930923	STM0858	2.427	0.592	1	0
20288	931339	STM0858	2.235	0.494	0	0
21994	931421	STM0858	2.062	0.489	0	0
10439	931696	STM0858	2.426	0.445	1	0

17348	931989		2.854	0.496	1	0
12637	932260	STM0859	2.259	0.558	0	0
2388	932464	STM0859	2.162	0.566	0	0
18064	932725	STM0859	2.208	0.604	0	0
2045	932947	STM0859	1.932	0.740	0	0
1993	933089	STM0859	1.638	0.705	0	0
6280	933347	STM0859, STM0860	1.510	0.553	0	0
11699	933606	STM0860, yliI	1.426	0.582	0	0
16037	933835	STM0860, yliI	1.605	0.685	0	0
20527	933913	STM0860, yliI	1.564	0.655	0	0
18259	934280	yliI	1.026	0.855	0	0
20899	934517	yliI	0.758	0.840	0	0
15963	934734	yliJ	1.107	1.485	0	0
3675	934814	yliJ	0.712	1.104	0	0
8753	935029	yliJ, dacC	1.028	1.104	0	0
16696	935169	yliJ, dacC	0.880	1.037	0	0
6879	935309	yliJ, dacC	1.365	1.197	0	0
14897	935393	yliJ, dacC	1.026	1.122	0	0
10069	936073	dacC	0.930	0.893	0	0
16678	936265	dacC	0.823	0.717	0	0
19901	936597	dacC	0.435	0.648	0	0
11438	936742	deoR	1.055	1.723	0	0
8492	936996	deoR	1.065	1.695	0	0
5454	937112	deoR	0.773	1.487	0	0
6094	937528	deoR	0.464	0.853	0	0
11415	937692	deoR, ybjG	0.435	0.805	0	0
11774	937884	deoR, ybjG	0.942	1.885	0	1
12306	938161	ybjG, mdfA	1.067	1.991	0	1
11321	938407	ybjG, mdfA	0.835	1.487	0	0
12302	938538	ybjG, mdfA	0.588	0.813	0	0
6109	938797	mdfA	1.113	1.319	0	0
18338	938992	mdfA	0.990	1.032	0	0
16976	939178	mdfA	0.784	0.796	0	0
715	939549	mdfA	1.617	1.142	0	0
8199	939643	mdfA	1.060	0.946	0	0
3812	939752	STM0867	1.078	1.075	0	0

15936	940108	STM0867	0.738	0.451	0	0
19003	940180	STM0867	1.489	0.937	0	0
1124	940578	STM0867, STM0868	1.529	0.849	0	0
1815	940863	STM0867, STM0868	1.340	1.135	0	0
16357	941140	STM0868	1.229	0.866	0	0
7432	941355	STM0868	1.216	0.797	0	0
12038	941603	STM0868, STM0869	0.974	0.992	0	0
3723	941812	STM0868, STM0869	1.026	0.862	0	0
6555	941930	STM0868, STM0869	0.994	0.950	0	0
3971	942146	STM0868, STM0869	1.030	1.006	0	0
16183	942305	STM0869	1.097	1.084	0	0
783	942663		1.078	1.128	0	0
15067	942777	STM0870	1.096	1.118	0	0
19957	943184	STM0870	1.295	0.897	0	0
10020	943321	STM0870	0.837	0.687	0	0
7373	943494	STM0870	1.226	0.848	0	0
2958	943881	STM0870	1.185	0.774	0	0
18133	944101	STM0870	1.178	0.546	0	0
3537	944250	STM0870, ybjM	2.230	0.859	0	0
21637	944534	STM0870, ybjM	1.656	0.875	0	0
17916	944684	STM0870, ybjM	1.459	1.192	0	0
3340	944773	STM0870, ybjM	1.042	1.082	0	0
20615	945161	grxA, ybjC	0.998	1.198	0	0
3686	945396	grxA, ybjC	0.868	1.335	0	0
1930	945513	grxA, ybjC	0.857	1.034	0	0
6676	945751	grxA, ybjC	1.328	0.998	0	0
5079	945883	mdaA	0.961	0.920	0	0
12017	946172	mdaA, rimK	1.325	1.055	0	0
7083	946418	mdaA, rimK	1.231	1.141	0	0
15468	946554	rimK	1.297	1.313	0	0
1427	947068	rimK, ybjN	1.084	1.135	0	0
22163	947219	rimK, ybjN	0.958	1.197	0	0
19355	947528	ybjN	0.996	1.256	0	0
11288	947670	ybjN	0.996	1.089	0	0
1404	947899	ybjN, potF	0.920	1.006	0	0
16778	948208	potF	1.168	0.885	0	0

6050	948312	potF	1.008	0.735	0	0
10613	948521	potF	1.098	0.684	0	0
15421	948848	potF	1.235	0.628	0	0
18520	948930	potF	1.192	0.593	0	0
21660	949285	potF, potG	1.610	0.674	0	0
10880	949467	potF, potG	1.496	0.655	0	0
18226	949667	potG	1.021	0.490	0	0
3285	949844	potG	0.885	0.504	0	0
8439	950077	potG	1.280	0.724	0	0
3901	950409	potG, potH	1.009	0.737	0	0
18890	950547	potG, potH	1.211	0.764	0	0
21781	950811	potH	1.291	0.744	0	0
13454	951007	potH	1.409	0.808	0	0
22187	951284	potH, potI	1.598	0.974	0	0
10940	951593	potH, potI	0.918	0.708	0	0
6358	951742	potI	1.230	0.936	0	0
8065	951836	potI	1.149	0.926	0	0
18548	952219	potI, ybjO	1.399	1.212	0	0
14458	952409	potI, ybjO	1.454	1.500	0	0
7295	952570	ybjO	1.477	1.444	0	0
11845	952978	ybjO, ybjF	1.034	1.051	0	0
5165	953152	ybjF	1.610	1.240	0	0
5044	953261	ybjF	1.310	0.853	0	0
5741	953629	ybjF	1.453	0.732	0	0
13556	953701	ybjF	1.404	0.660	0	0
17405	954134	ybjF, STM0884	3.683	0.636	1	0
20295	954340	STM0884	3.533	0.511	1	0
18214	954430	STM0884	3.971	0.464	1	0
3760	954822	STM0884	2.135	0.521	0	0
22189	954913	STM0884	2.304	0.590	0	0
17629	955050	STM0884	1.836	0.634	0	0
15754	955406	STM0884, STM0885	1.998	0.666	0	0
17058	955672	STM0884, STM0885	3.810	0.644	1	0
22147	955820	STM0884, STM0885	3.834	0.566	1	0
3122	956073	STM0885, STM0886	3.527	0.516	1	0
14468	956203	STM0886	3.362	0.575	1	0

18847	956461	STM0886	1.781	0.586	0	0
3541	956816	STM0886	1.424	0.704	0	0
18825	956967	STM0886	1.232	0.682	0	0
13368	957094	STM0886	1.308	0.794	0	0
19132	957525	STM0886	0.800	0.611	0	0
3491	957616	STM0886	1.172	0.782	0	0
12978	957876		0.964	0.760	0	0
3912	958217	artJ	0.742	0.740	0	0
17368	958431	artJ	1.009	0.853	0	0
10152	958512	artJ	0.932	0.833	0	0
13721	958626	artJ	0.700	0.771	0	0
12309	959017	artJ, artM	0.708	1.047	0	0
9578	959325	artM	0.910	1.238	0	0
13719	959599	artM, artQ	0.978	1.322	0	0
11529	959704	artM, artQ	0.944	1.243	0	0
15826	959836	artM, artQ	0.586	0.904	0	0
6444	960053	artQ	0.808	1.135	0	0
20722	960295	artQ, artI	0.968	1.342	0	0
15174	960554	artQ, artI	0.890	1.086	0	0
2360	960638	artQ, artI	0.730	0.988	0	0
982	961023	artI, artP	0.805	1.088	0	0
19707	961189	artI, artP	0.737	0.968	0	0
1312	961488	artP	0.796	1.421	0	0
2092	961750	artP	0.808	1.147	0	0
12078	961956	artP	0.663	1.131	0	0
8790	962085	artP, ybjP	0.725	1.345	0	0
12073	962481	ybjP	0.885	1.157	0	0
13014	962699	ybjP, STM0893	0.715	0.935	0	0
16657	962922	ybjP, STM0893	0.817	1.037	0	0
3783	963124	STM0893	0.911	1.148	0	0
6530	963388	STM0893	0.942	0.933	0	0
259	963485	STM0893	1.250	1.043	0	0
15565	963591	STM0893	0.995	1.077	0	0
4754	963920	STM0893	1.136	0.953	0	0
361	964334	STM0893, STM0894	0.820	0.639	0	0
18389	964599	STM0894, STM0895	0.710	0.496	0	0

20014	964824	STM0895	0.978	0.715	0	0
8898	964938	STM0895	1.078	0.549	0	0
20107	965081	STM0895	1.422	0.560	0	0
4430	965451	STM0896	1.550	0.573	0	0
5331	965658	STM0896	1.214	0.581	0	0
5638	965729	STM0896	1.642	0.617	0	0
14345	966110	STM0896	2.331	0.535	1	0
11980	966290	STM0897	2.938	0.502	1	0
12602	966427	STM0897	3.033	0.394	1	0
3685	966687	STM0897	2.431	0.539	0	0
15945	966851	STM0897	2.598	0.507	0	0
21504	967196		2.010	0.549	0	0
2279	967379		1.689	0.441	0	0
15655	967493		1.792	0.461	0	0
501	967947	STM0898	0.680	0.530	0	0
13578	968219	STM0898, STM0898A	0.712	0.629	0	0
11108	968296	STM0898, STM0898A	0.559	0.564	0	0
318	968565	STM0898, STM0898A	0.619	0.526	0	0
10342	968876	STM0898, STM0898A	0.882	0.498	0	0
10077	968977	STM0898, STM0899	0.831	0.454	0	0
21349	969364	STM0899	0.879	0.503	0	0
21316	969444	STM0899	1.019	0.521	0	0
5814	969527	STM0899, STM0900	1.242	0.645	0	0
10322	969719	STM0899, STM0900	1.376	0.794	0	0
18451	970162	STM0900	1.243	0.707	0	0
22201	970282	STM0900	1.359	0.751	0	0
15748	970479	STM0900	1.693	0.824	0	0
11067	970726	STM0900	1.365	0.733	0	0
1297	970993	STM0900	1.522	0.868	0	0
18502	971377	STM0900, STM0901	1.421	0.755	0	0
10391	971494	STM0900, STM0901	1.185	0.659	0	0
15070	971789	STM0901	1.742	0.746	0	0
17664	972008	STM0901	0.786	0.396	0	0
18761	972145	STM0901, STM0902	1.472	0.656	0	0
9614	972529	STM0902	1.005	0.530	0	0
14786	972675	STM0902	1.251	0.645	0	0

14428	972928	STM0902, STM0903	1.366	0.590	0	0
2030	973183	STM0902, STM0903	1.262	0.530	0	0
12428	973378	STM0903	1.384	0.698	0	0
8390	973462	STM0903	1.350	0.681	0	0
8752	973636	STM0903	0.752	0.734	0	0
5101	973978	STM0903	0.960	1.159	0	0
14641	974366	STM0904	0.816	0.731	0	0
11652	974599	STM0904	1.485	0.876	0	0
7294	974711	STM0904	1.322	0.741	0	0
4059	974938	STM0904	1.265	0.699	0	0
19950	975069	STM0904	1.480	0.823	0	0
16900	975349	STM0905	1.051	0.725	0	0
2683	975680	STM0905	1.251	0.611	0	0
13223	975868	STM0905	1.678	0.668	0	0
10235	975988	STM0905	1.675	0.687	0	0
9028	976279		1.348	0.732	0	0
13075	976475	STM0906	1.030	0.817	0	0
7609	976807	STM0906, STM0907	0.967	0.776	0	0
3191	977060	STM0906, STM0907	0.964	0.687	0	0
12763	977287	STM0907, STM0908	1.202	0.684	0	0
18083	977359	STM0907, STM0908	0.942	0.609	0	0
11297	977758	STM0908	1.165	0.613	0	0
11242	977829	STM0908	1.097	0.623	0	0
2687	978219	STM0908	2.508	0.665	0	0
2970	978439		1.581	0.604	0	0
5500	978626	STM0909	2.019	0.493	0	0
22260	978787	STM0909	2.052	0.623	0	0
15633	978860	STM0909	1.323	0.554	0	0
22095	979002	STM0909	0.962	0.611	0	0
6077	979494	STM0909, STM0910	1.057	0.515	0	0
17191	979724	STM0910	1.658	0.703	0	0
11168	979929	STM0910	1.096	0.674	0	0
14579	980014	STM0910	1.244	0.599	0	0
12469	980092	STM0910	1.498	0.793	0	0
5068	980672	STM0910	0.781	0.443	0	0
2599	980858	STM0910	1.188	0.645	0	0

20423	981144	STM0910	1.254	0.641	0	0
18123	981326	STM0910	0.286	0.299	0	0
652	981574	STM0910, STM0911	0.895	0.434	0	0
15840	981834	STM0911	1.743	0.686	0	0
12983	981988	STM0911	0.905	0.534	0	0
229	982174	STM0911	0.849	0.507	0	0
15615	982396	STM0911	0.693	0.510	0	0
18469	982736	STM0911	1.223	0.650	0	0
15391	982967	STM0911, STM0912	0.842	0.542	0	0
10383	983054	STM0911, STM0912	0.727	0.542	0	0
6619	983306	STM0911, STM0912	2.084	0.726	0	0
6971	983592	STM0912	0.352	0.467	0	0
4109	983784	STM0912	1.539	0.744	0	0
4413	983965	STM0912	1.536	0.686	0	0
7580	984159	STM0912	1.120	0.602	0	0
4691	984343	STM0912	0.320	0.296	0	0
11973	984654	STM0912	1.313	0.699	0	0
15184	984931	STM0912	0.532	0.600	0	0
13467	985145	STM0912	1.818	0.736	0	0
13940	985444	STM0913	1.496	0.745	0	0
9264	985520	STM0913	1.279	0.620	0	0
19052	985716	STM0914, STM0913	0.893	0.536	0	0
20944	986096	STM0914, STM0913	1.238	0.704	0	0
18568	986284	STM0914, STM0913	1.782	0.699	0	0
1000	986609	STM0914, STM0915	1.980	0.784	0	0
2420	986681	STM0914, STM0915	0.894	0.624	0	0
20212	986966	STM0915, STM0916	1.653	0.738	0	0
796	987099	STM0916	1.188	0.639	0	0
11353	987494	STM0916	1.686	0.840	0	0
14482	987667	STM0916	0.755	0.516	0	0
13665	987895	STM0917	1.644	0.637	0	0
4781	988134	STM0917, STM0918	1.577	0.623	0	0
18988	988235	STM0917, STM0918	1.087	0.539	0	0
2113	988529	STM0917, STM0918	2.050	0.852	0	0
7231	988671	STM0918	1.738	0.727	0	0
2989	988750	STM0918	1.329	0.564	0	0

10398	989291	STM0918	2.112	0.819	0	0
17502	989524	STM0918	1.192	0.605	0	0
6836	989782	STM0918	1.044	0.607	0	0
7677	989940	STM0918	1.249	0.744	0	0
10226	990139	STM0918	1.644	0.958	0	0
9341	990352	STM0918	1.280	0.888	0	0
16762	990631	STM0918	0.927	0.644	0	0
11851	990852	STM0918	1.785	0.829	0	0
14990	990974	STM0918	1.320	0.782	0	0
14073	991283	STM0918	1.370	0.778	0	0
15055	991363	STM0918, STM0919	0.994	0.654	0	0
459	991761	STM0919, STM0920	1.799	1.370	0	0
2637	992068	STM0919, STM0920	1.071	1.165	0	0
9401	992232	STM0920	0.876	1.027	0	0
18862	992405	STM0920, STM0921	1.025	1.354	0	0
8323	992662	STM0920, STM0921	1.242	1.267	0	0
14779	992780	STM0921	1.226	1.210	0	0
21814	992980	STM0921	1.252	1.106	0	0
2922	993052	STM0921	1.230	1.128	0	0
4644	993559	STM0922	1.381	0.891	0	0
1695	993678	STM0922	1.230	0.996	0	0
15028	994106	STM0922, STM0923	1.422	0.785	0	0
12976	994316	STM0923	1.394	0.843	0	0
15209	994487	STM0923	0.438	0.384	0	0
8124	994712	STM0923	0.690	0.658	0	0
8236	994936	STM0924	0.883	0.755	0	0
20045	995142	STM0924, STM0925	1.017	0.811	0	0
12968	995304	STM0924, STM0925	1.086	0.666	0	0
3860	995398	STM0924, STM0925	1.116	0.574	0	0
2766	995754	STM0924, STM0925	1.207	0.683	0	0
21934	996084	STM0925	1.533	0.787	0	0
17470	996233	STM0925	1.388	0.608	0	0
13525	996384	STM0925	1.646	0.668	0	0
3972	996698	STM0925	0.894	0.505	0	0
5587	996969	STM0925	0.846	0.529	0	0
13147	997111	STM0925	1.405	0.708	0	0

20510	997388	STM0925	0.970	0.506	0	0
3649	997561	STM0925	0.890	0.483	0	0
15178	997849	STM0925	2.021	0.703	0	0
6893	998090	STM0925	2.020	0.563	0	0
3615	998219	STM0925	0.758	0.454	0	0
8615	998520	STM0925	1.662	0.763	0	0
9420	998702	STM0925, STM0926	1.430	0.761	0	0
5119	998937	STM0925, STM0926	0.594	0.335	0	0
10225	999008	STM0926	2.034	0.657	0	0
21031	999274	STM0926	2.060	0.695	0	0
9124	999646	STM0926	1.856	0.685	0	0
9351	999895	STM0926	0.776	0.516	0	0
3494	1000103	STM0926	1.698	0.640	0	0
14699	1000400	STM0926	1.609	0.718	0	0
6091	1000509	STM0926	1.950	0.754	0	0
16905	1000671	STM0926	1.743	0.881	0	0
6321	1001129	STM0926, STM0927	1.966	0.788	0	0
3275	1001200	STM0926, STM0927	1.986	0.740	0	0
6093	1001433	STM0927	2.405	0.608	1	0
19128	1001640	STM0927	2.266	0.640	1	0
20139	1001991		2.362	0.502	1	0
10886	1002106	nanH	2.308	0.517	0	0
17961	1002382	nanH	2.463	0.524	0	0
10634	1002637	nanH	2.166	0.540	0	0
6516	1002775	nanH	2.015	0.553	0	0
15054	1003092	nanH	1.625	0.543	0	0
11123	1003228	nanH	0.994	0.665	0	0
8471	1003535	nanH	1.959	0.619	0	0
2385	1003818		1.750	0.630	0	0
10292	1004028	STM0929	1.723	0.601	0	0
3149	1004340	STM0929	1.587	0.592	0	0
16251	1004418	STM0929	1.748	0.600	0	0
8287	1004805		1.625	0.468	0	0
5783	1004889	orfB	1.490	0.563	0	0
17194	1005005	orfB	1.249	0.630	0	0
20869	1005295	orfB, ybjR	1.111	0.884	0	0

328	1005602	orfB, ybjR	1.072	1.098	0	0
3574	1005722	ybjR	1.257	1.089	0	0
7318	1006106	ybjR	1.153	1.352	0	0
1194	1006325	ybjR	1.008	1.938	0	1
10978	1006477	ybjR	0.818	1.805	0	1
17856	1006760	STM0932	0.866	2.239	0	1
1435	1006988	STM0932	0.938	1.648	0	0
16021	1007152	STM0932	0.756	0.876	0	0
5092	1007514	STM0932	0.617	3.908	0	1
11580	1007592	STM0932	0.548	3.927	0	1
1079	1007832	STM0932, ybjT	0.497	1.916	0	0
10740	1007976	ybjT	0.635	1.319	0	0
13951	1008260	ybjT	1.077	1.038	0	0
5082	1008443	ybjT	0.932	0.795	0	0
19838	1008825	ybjT	1.135	1.207	0	0
2639	1008896	ybjT	1.131	1.180	0	0
6041	1009135	ybjT, ltaA	0.928	0.982	0	0
17935	1009466	ybjT, ltaA	0.797	0.739	0	0
11867	1009803	ltaA	0.685	0.774	0	0
17487	1009876	ltaA	0.575	0.679	0	0
9916	1010038	ltaA	1.098	0.915	0	0
3973	1010439	ltaA, poxB	1.525	0.804	0	0
7324	1010599	poxB	1.328	0.768	0	0
3338	1010738	poxB	1.177	0.538	0	0
10206	1010907	poxB	0.912	0.612	0	0
834	1011009	poxB	1.288	0.601	0	0
2224	1011414	poxB	2.592	0.785	0	0
9026	1011795	poxB	1.363	0.912	0	0
3244	1011873	poxB	1.178	0.750	0	0
16146	1012150	poxB, hcr	0.991	0.938	0	0
4649	1012435	hcr	1.207	1.093	0	0
1902	1012616	hcr	0.991	0.925	0	0
18192	1012979	hcr, hcp	1.068	1.239	0	0
3342	1013085	hcr, hcp	0.985	1.302	0	0
5745	1013277	hcr, hcp	1.127	1.388	0	0
7873	1013626	hcp	1.148	1.063	0	0

9563	1013848	hcp	1.282	0.866	0	0
20714	1013958	hcp	1.051	0.704	0	0
5342	1014045	hcp	0.746	0.521	0	0
784	1014575	hcp	0.701	0.732	0	0
21840	1014653	hcp	0.890	0.637	0	0
10873	1014749	hcp, ybjE	0.594	0.687	0	0
18157	1015200	ybjE	1.393	1.189	0	0
3717	1015392	ybjE, ybjD	1.505	1.033	0	0
14110	1015673	ybjE, ybjD	1.365	1.143	0	0
13341	1015831	ybjE, ybjD	1.375	0.939	0	0
6825	1015981	ybjE, ybjD	1.144	0.873	0	0
6291	1016241	ybjD	1.070	0.814	0	0
19726	1016566	ybjD	1.398	1.047	0	0
2695	1016717	ybjD	0.916	0.995	0	0
12538	1017049	ybjD	0.765	1.088	0	0
2278	1017267	ybjD	0.777	1.731	0	1
21754	1017338	ybjD	0.780	1.638	0	1
17204	1017753	ybjX	0.831	1.353	0	0
2111	1017912	ybjX	0.745	1.331	0	0
3782	1018003	ybjX	0.746	1.403	0	0
719	1018177	ybjX, ybjY	0.919	1.857	0	0
12183	1018594	ybjX, ybjY	0.665	2.301	0	1
11718	1018822	ybjX, ybjY	0.744	1.657	0	1
13806	1019103	ybjY	0.845	1.204	0	0
12605	1019341	ybjY, ybjZ	0.710	0.877	0	0
13997	1019489	ybjY, ybjZ	1.155	1.143	0	0
18044	1019619	ybjY, ybjZ	0.860	0.968	0	0
15528	1019724	ybjZ	1.093	1.313	0	0
8529	1020101	ybjZ	0.932	1.241	0	0
7445	1020338	ybjZ	1.103	1.177	0	0
3806	1020516	ybjZ	0.959	1.022	0	0
1319	1020846	ybjZ	0.926	1.882	0	1
9027	1021110	ybjZ	0.798	2.519	0	1
10054	1021243	ybjZ	0.737	2.030	0	1
5234	1021342	ybjZ	0.770	2.115	0	1
8380	1021774	cspD	0.569	1.864	0	1

20541	1021889	cspD, yljA	0.667	2.293	0	1
1615	1022236	cspD, yljA	0.627	1.182	0	0
8142	1022318	cspD, yljA	0.626	1.336	0	0
14702	1022498	yljA, clpA	0.713	1.400	0	0
5547	1022665	clpA	0.679	1.353	0	0
6301	1022869	clpA	0.494	1.348	0	0
14269	1023089	clpA	0.704	1.889	0	0
11250	1023649	clpA	0.967	1.480	0	0
12622	1023720	clpA	1.013	1.363	0	0
21154	1023851	clpA	0.743	1.249	0	0
9796	1024235	clpA	0.908	1.530	0	0
6780	1024522	clpA	0.832	1.411	0	0
1110	1024667	clpA, tnpA_1	0.726	1.778	0	0
1112	1024899	clpA, tnpA_1	1.264	1.309	0	0
18729	1025100	tnpA_1	1.683	0.852	0	0
337	1025451	tnpA_1	1.232	0.860	0	0
17959	1025686		1.209	0.844	0	0
14798	1025817		1.047	0.805	0	0
18162	1026061	STM0947	0.843	0.794	0	0
15428	1026333	STM0947	1.050	0.549	0	0
21365	1026449	STM0947	1.149	0.797	0	0
17736	1026838	STM0947	0.875	1.102	0	0
12129	1027009	STM0947, STM0948	0.904	1.624	0	0
5969	1027253	STM0948	0.875	2.772	0	1
10998	1027352	STM0948	0.761	3.009	0	1
12582	1027580	STM0948, serW	0.681	3.212	0	1
1135	1027684	STM0948, serW	0.487	1.585	0	0
8060	1027900	serW, STM0950	0.742	1.554	0	0
21585	1028335	STM0950	1.003	0.835	0	0
12525	1028423	STM0950, STM0951	0.802	0.792	0	0
21770	1028792	STM0950, STM0951	0.907	0.750	0	0
9397	1029121	STM0951, STM0952	1.046	0.695	0	0
13882	1029218	STM0951, STM0952	0.534	0.556	0	0
12207	1029334	STM0951, STM0952	0.523	0.608	0	0
1460	1029512	STM0951, STM0952	1.053	0.911	0	0
4186	1029685	STM0952	0.922	1.023	0	0

21184	1030225	STM0952	0.694	2.339	0	1
9145	1030431	infA	0.570	2.164	0	1
3560	1030548	infA, STM0954	0.507	2.045	0	1
17769	1030625	infA, STM0954	0.599	2.389	0	1
6789	1030731	infA, STM0954	0.741	1.969	0	1
14904	1031010	STM0954, aat	0.853	1.770	0	1
9599	1031587	aat	0.972	1.857	0	1
3229	1031755	aat, cydC	0.670	1.418	0	0
11786	1031921	aat, cydC	0.612	1.014	0	0
18005	1032261	cydC	1.191	1.826	0	0
1447	1032368	cydC	1.198	1.494	0	0
7304	1032615	cydC	0.639	1.069	0	0
21078	1032864	cydC	1.141	1.373	0	0
10548	1033027	cydC	0.845	0.930	0	0
17474	1033280	cydC	1.157	1.480	0	0
7658	1033446	cydC, cydD	0.879	1.264	0	0
13435	1033893	cydD	1.289	1.383	0	0
16137	1034024	cydD	1.154	1.357	0	0
12965	1034258	cydD	1.133	1.524	0	0
3896	1034408	cydD	0.947	1.558	0	0
14444	1034682	cydD	1.071	1.379	0	0
10512	1034796	cydD	0.854	1.267	0	0
13144	1035084	cydD	0.789	1.209	0	0
3461	1035191	cydD	0.810	1.414	0	0
422	1035566	trxB	0.715	1.274	0	0
14221	1035903	trxB	0.667	1.301	0	0
17734	1036039	trxB	0.597	1.226	0	0
9847	1036191	trxB	0.599	1.165	0	0
10204	1036430	trxB, lrp	0.542	1.212	0	0
12028	1036702	lrp	0.668	1.263	0	0
14286	1036797	lrp	0.541	1.614	0	1
12125	1037205	lrp, ftsK	0.462	1.685	0	1
4364	1037356	ftsK	0.551	2.217	0	1
3284	1037584	ftsK	0.510	2.209	0	1
14571	1037988	ftsK	0.911	2.265	0	1
952	1038097	ftsK	0.840	2.737	0	1

907	1038238	ftsK	1.087	2.669	0	1
2560	1038596	ftsK	0.547	1.081	0	0
9655	1038765	ftsK	0.292	0.633	0	0
8498	1038870	ftsK	1.038	2.904	0	1
18509	1039296	ftsK	1.019	3.542	0	1
13575	1039439	ftsK	0.933	2.719	0	1
20005	1039653	ftsK	1.090	2.102	0	1
5886	1039882	ftsK	0.624	1.350	0	0
17825	1040205	ftsK	0.893	1.928	0	1
15734	1040415	ftsK	0.725	1.700	0	1
11070	1040486	ftsK	0.808	1.912	0	1
3781	1040915	ftsK	0.687	1.748	0	1
15352	1041018	ftsK	0.562	1.550	0	0
15993	1041220	ftsK, lolA	0.728	1.840	0	0
3184	1041519	lolA	0.730	1.320	0	0
16974	1041781	lolA, ycaJ	0.856	1.696	0	0
16319	1041863	lolA, ycaJ	0.769	1.616	0	1
5252	1042166	ycaJ	0.871	2.225	0	1
15241	1042525	ycaJ	0.715	1.502	0	0
21618	1042663	ycaJ	0.815	1.513	0	0
15226	1042923	ycaJ	0.870	1.811	0	0
11649	1043195	ycaJ	0.669	1.413	0	0
9421	1043423	ycaJ, serS	0.772	1.847	0	1
2159	1043531	serS	0.761	2.247	0	1
22082	1043752	serS	0.737	2.251	0	1
16454	1044132	serS	0.794	1.656	0	0
1633	1044314	serS	0.509	1.120	0	0
21486	1044599	serS	0.761	1.383	0	0
18690	1044729	serS	0.721	1.275	0	0
8890	1044939	serS, dmsA	0.364	0.512	0	0
13103	1045051	serS, dmsA	0.674	0.640	0	0
5181	1045286	dmsA	0.975	0.514	0	0
13437	1045533	dmsA	1.150	0.664	0	0
671	1045714	dmsA	1.389	0.873	0	0
21900	1045983	dmsA	0.818	0.702	0	0
20668	1046237	dmsA	1.474	0.655	0	0

15267	1046602	dmsA	1.311	0.757	0	0
12160	1046807	dmsA	1.337	0.662	0	0
19476	1046968	dmsA	0.966	0.554	0	0
18235	1047122	dmsA	1.083	0.591	0	0
4926	1047328	dmsA, dmsB	1.132	0.639	0	0
9001	1047408	dmsA, dmsB	1.249	0.672	0	0
10717	1047486	dmsA, dmsB	1.296	0.674	0	0
10525	1047634	dmsA, dmsB	1.080	0.594	0	0
13869	1047705	dmsA, dmsB	1.249	0.617	0	0
3599	1048531	dmsC	0.348	0.328	0	0
9394	1048776	dmsC	1.572	0.700	0	0
10746	1049123	dmsC, ycaD	1.384	0.726	0	0
4692	1049369	ycaD	2.140	0.787	0	0
2424	1049559	ycaD	1.868	0.772	0	0
9243	1049794	ycaD	1.864	0.952	0	0
8525	1049865	ycaD	0.483	0.720	0	0
9809	1050034	ycaD	1.454	0.787	0	0
17712	1050127	ycaD	1.350	0.794	0	0
15747	1050387	ycaD	1.173	0.534	0	0
3941	1050858	ycaM	2.799	0.752	1	0
9017	1051028	ycaM	2.625	0.758	1	0
4006	1051379	ycaM	1.625	0.550	0	0
13969	1051513	ycaM	1.324	0.630	0	0
8103	1051760	ycaM	2.118	0.611	0	0
4841	1051986	ycaM	1.819	0.526	0	0
5978	1052059	ycaM	1.974	0.635	0	0
19942	1052493		1.241	0.832	0	0
606	1052698	pflA	0.962	0.797	0	0
16184	1052983	pflA	0.689	0.739	0	0
20548	1053215	pflA	0.968	0.824	0	0
5136	1053397	pflA	1.020	0.770	0	0
11270	1053595	pflA, STM0971	1.011	0.687	0	0
20199	1053829	pflA, STM0971	1.472	0.629	0	0
8123	1054122	STM0971, STM0972	1.781	0.582	0	0
20282	1054230	STM0971, STM0972	2.035	0.572	0	0
13361	1054430	STM0972	2.227	0.499	0	0

12070	1054631	STM0972	2.545	0.528	0	0
6907	1054760	STM0972	2.512	0.458	0	0
12780	1055036		2.737	0.575	0	0
20919	1055377	pflB	1.762	0.732	0	0
6108	1055512	pflB	1.096	0.911	0	0
16086	1055605	pflB	0.934	0.788	0	0
2282	1055697	pflB	0.745	0.668	0	0
6039	1056173	pflB	1.014	0.834	0	0
21655	1056433	pflB	0.845	1.006	0	0
5090	1056753	pflB	0.941	0.972	0	0
2780	1056944	pflB	0.862	0.927	0	0
19258	1057262	pflB	0.854	0.908	0	0
10386	1057338	pflB	0.775	0.659	0	0
2693	1057622	pflB, focA	1.085	0.891	0	0
16990	1057924	focA	1.699	0.850	0	0
8308	1058040	focA	1.788	0.737	0	0
2777	1058261	focA	2.487	0.667	0	0
19056	1058663	focA	2.693	0.765	0	0
7731	1058865	ycaO	2.148	0.766	0	0
7753	1059139	ycaO	1.663	0.836	0	0
5762	1059261	ycaO	1.194	0.791	0	0
12889	1059343	ycaO	1.155	0.766	0	0
14735	1059812	ycaO	1.191	0.844	0	0
8486	1059919	ycaO	1.184	0.710	0	0
16741	1060184	ycaO, ycaP	1.012	0.751	0	0
5800	1060428	ycaO, ycaP	1.169	0.862	0	0
21140	1060665	ycaO, ycaP	0.838	0.564	0	0
638	1060883	ycaO, ycaP	1.045	0.901	0	0
2454	1061050	ycaP, serC	1.008	0.810	0	0
8216	1061286	serC	1.076	1.051	0	0
8926	1061561	serC	1.172	1.175	0	0
8496	1061791	serC	1.167	1.327	0	0
14473	1061879	serC	1.120	1.504	0	0
20790	1062269	serC, aroA	0.953	1.401	0	0
10304	1062367	serC, aroA	0.810	1.197	0	0
10919	1062587	aroA	0.677	0.820	0	0

1454	1062676	aroA	0.738	0.780	0	0
12626	1063210	aroA	1.060	1.103	0	0
10629	1063287	aroA	0.791	0.927	0	0
20777	1063599	aroA, ycaL	1.117	1.028	0	0
13695	1063834	aroA, ycaL	1.059	0.917	0	0
21331	1063961	ycaL	1.076	1.165	0	0
11160	1064223	ycaL	0.968	0.938	0	0
4643	1064295	ycaL	1.010	0.956	0	0
5073	1064691	ycaL, cmk	0.905	0.970	0	0
7821	1064868	cmk	0.791	1.036	0	0
8672	1065008	cmk	0.734	1.176	0	0
19282	1065458	cmk, rpsA	0.488	3.018	0	1
22115	1065587	cmk, rpsA	0.477	3.146	0	1
15582	1065786	rpsA	0.618	2.253	0	1
15529	1066069	rpsA	0.468	2.164	0	1
10256	1066345	rpsA	0.539	1.984	0	1
9710	1066472	rpsA	0.575	2.484	0	1
20901	1066699	rpsA	0.400	1.274	0	0
21359	1066884	rpsA	0.524	2.328	0	1
14150	1067083	rpsA	0.565	2.018	0	1
5838	1067465	himD	0.670	1.851	0	0
2549	1067587	himD	0.746	2.097	0	0
7664	1067885	ycaI	0.806	1.350	0	0
12448	1068001	ycaI	0.938	1.142	0	0
18130	1068179	ycaI	0.727	0.814	0	0
3505	1068621	ycaI	0.946	1.246	0	0
4576	1068849	ycaI	1.267	0.830	0	0
13293	1069070	ycaI	1.264	0.904	0	0
12067	1069357	ycaI	0.923	0.662	0	0
17193	1069435	ycaI	0.947	0.665	0	0
16282	1069790	ycaI	0.826	0.878	0	0
19130	1069885	ycaI	0.925	0.929	0	0
4595	1070101	ycaI, msbA	0.906	1.502	0	0
5218	1070416	msbA	0.860	2.240	0	0
18053	1070661	msbA	0.643	1.389	0	0
21730	1070877	msbA	0.738	1.354	0	0

15809	1071114	msbA	0.758	1.338	0	0
9782	1071375	msbA	1.046	1.429	0	0
20367	1071574	msbA	0.785	1.292	0	0
7051	1071806	msbA, lpxK	0.906	1.439	0	0
4650	1072047	msbA, lpxK	0.793	1.437	0	0
20803	1072151	msbA, lpxK	0.965	1.959	0	0
3100	1072468	lpxK	0.798	1.299	0	0
9064	1072599	lpxK	0.909	1.702	0	0
11539	1072980	lpxK, ycaQ	0.800	1.244	0	0
16371	1073174	ycaQ	0.950	1.659	0	0
18531	1073325	ycaQ	0.812	1.167	0	0
3090	1073484	ycaQ	1.204	1.055	0	0
17518	1073711	ycaQ	1.258	1.143	0	0
12835	1074083	ycaQ, ycaR	1.184	0.969	0	0
17828	1074223	ycaQ, ycaR	1.317	1.153	0	0
14717	1074529	ycaR, kdsB	1.132	1.328	0	0
1063	1074691	kdsB	0.982	1.044	0	0
10969	1074897	kdsB	0.807	0.912	0	0
12931	1075070	kdsB	0.911	1.241	0	0
22264	1075165	kdsB, STM0989	1.221	1.477	0	0
3363	1075520	STM0989	0.875	1.460	0	0
16279	1075672	STM0989	0.914	1.150	0	0
16830	1076127	STM0989	0.534	0.582	0	0
11667	1076307	STM0989	1.057	1.113	0	0
15904	1076439	STM0989	1.416	1.222	0	0
6222	1076761	ycbC	1.232	1.244	0	0
12673	1077061	ycbC, smtA	0.895	1.240	0	0
13306	1077242	ycbC, smtA	0.931	1.801	0	1
16260	1077400	ycbC, smtA	0.904	2.438	0	1
3484	1077721	smtA, mukF	0.513	1.273	0	0
9487	1077844	smtA, mukF	0.721	1.893	0	0
20851	1078174	smtA, mukF	0.893	2.214	0	0
9258	1078420	mukF	0.895	1.942	0	0
3391	1078492	mukF	0.898	1.819	0	0
15009	1078909	mukF	0.467	0.867	0	0
2523	1079033	mukF, mukE	0.859	1.446	0	0

2547	1079233	mukF, mukE	0.892	1.534	0	0
19952	1079475	mukF, mukE	0.751	1.214	0	0
13641	1079622	mukE	0.982	1.603	0	0
18867	1079771	mukE, mukB	0.616	1.100	0	0
11591	1080127	mukE, mukB	0.774	1.456	0	0
9982	1080200	mukB	0.991	1.814	0	0
16722	1080676	mukB	0.676	1.359	0	0
8784	1080753	mukB	0.747	1.448	0	0
5841	1080957	mukB	0.827	1.183	0	0
12606	1081250	mukB	1.065	1.745	0	0
10586	1081541	mukB	0.801	1.353	0	0
3427	1081828	mukB	1.020	2.257	0	0
13540	1081947	mukB	0.732	1.659	0	0
2675	1082263	mukB	0.839	2.078	0	0
22243	1082401	mukB	0.717	1.603	0	0
18340	1082483	mukB	1.108	1.795	0	1
20963	1082982	mukB	1.293	1.722	0	1
19560	1083112	mukB	0.710	0.928	0	0
7605	1083247	mukB	0.713	0.949	0	0
17776	1083433	mukB	0.990	1.492	0	0
16367	1083794	mukB	1.035	1.606	0	0
15381	1083929	mukB	0.901	1.696	0	0
14753	1084209	mukB	0.574	0.990	0	0
20811	1084528	mukB, ycbB	0.694	1.258	0	0
10441	1084829	ycbB	0.832	1.179	0	0
4711	1084900	ycbB	1.100	1.432	0	0
16471	1085013	ycbB	0.456	0.936	0	0
3625	1085249	ycbB	0.982	1.735	0	0
8489	1085468	ycbB	0.758	1.482	0	0
18661	1085784	ycbB	0.786	1.195	0	0
16207	1086133	ycbB	0.993	1.592	0	0
1042	1086310	ycbB	0.714	1.098	0	0
3449	1086507	ycbB	0.634	1.497	0	0
425	1086832	ycbK	1.045	1.661	0	0
11695	1087053	ycbK	0.480	1.822	0	1
22353	1087291	ycbK, ycbL	0.639	2.514	0	1

6331	1087421	ycbK, ycbL	0.710	2.133	0	1
14158	1087658	ycbL	0.741	1.985	0	1
14024	1087903	ycbL	0.759	2.355	0	1
12075	1088204	ycbL	0.750	2.295	0	1
11248	1088302	ycbL	0.774	1.932	0	1
1781	1088527	aspC	0.698	1.375	0	0
21963	1088833	aspC	0.973	1.577	0	0
3944	1088982	aspC	0.496	0.826	0	0
19489	1089369	aspC	0.865	1.564	0	0
6149	1089469	aspC	0.972	1.833	0	0
5641	1089588	aspC	1.077	1.418	0	0
18951	1089737	aspC	0.760	1.201	0	0
7577	1090051	ompF	0.773	1.080	0	0
14425	1090309	ompF	0.693	0.903	0	0
17702	1090462	ompF	0.702	0.968	0	0
13790	1090848	ompF	0.935	0.929	0	0
14731	1090963	ompF	0.648	0.556	0	0
19082	1091332		0.761	0.512	0	0
7730	1091595	asnS	0.903	1.141	0	0
2704	1091764	asnS	0.835	1.192	0	0
16287	1092015	asnS	0.627	0.898	0	0
2563	1092257	asnS	0.556	0.955	0	0
6345	1092376	asnS	0.934	1.269	0	0
14108	1092690	asnS	1.059	1.625	0	0
17126	1092904	asnS	0.766	1.195	0	0
6209	1093124	asnS, STM1001	1.396	1.002	0	0
13189	1093289	asnS, STM1001	1.373	0.951	0	0
15355	1093526	STM1001, STM1002	2.666	0.812	0	0
6164	1093742	STM1001, STM1002	2.455	0.558	0	0
11573	1094033	STM1002	2.575	0.544	0	0
14026	1094284	STM1002	2.594	0.558	0	0
7953	1094443	STM1002	2.038	0.616	0	0
4838	1094634	STM1002	1.922	0.517	0	0
11582	1094929	STM1002, STM1003	2.809	0.483	1	0
10696	1095172	STM1003	3.270	0.486	1	0
7743	1095301	STM1003	3.803	0.520	1	0

2977	1095487	STM1003	3.205	0.497	1	0
2804	1095858	STM1003	2.755	0.587	1	0
3157	1095931	STM1003	3.196	0.587	1	0
5219	1096269	STM1003	3.221	0.518	1	0
2505	1096387	STM1003	2.943	0.462	1	0
20259	1096561	STM1003	3.693	0.606	1	0
9822	1096940	pncB	2.074	0.941	1	0
8822	1097082	pncB	1.582	1.018	0	0
11306	1097235	pncB	1.287	1.050	0	0
4052	1097742	pncB	1.132	1.500	0	0
17792	1097976	pncB	0.963	1.334	0	0
2226	1098180	pncB	0.797	1.418	0	0
9163	1098293	pncB, STM1005	0.800	1.270	0	0
3940	1098483	STM1005	0.885	1.329	0	0
17414	1098556	STM1005	0.897	1.468	0	0
19456	1099103	STM1005	0.711	1.003	0	0
17346	1099327	STM1005	0.935	1.048	0	0
16041	1099539	STM1005, STM1006	1.032	1.142	0	0
3228	1099755	STM1005, STM1006	0.985	0.806	0	0
20390	1099928	STM1005, STM1006	1.387	0.728	0	0
13848	1100128	STM1006, STM1007	1.275	0.713	0	0
13676	1100342	STM1007, STM1008	1.374	0.614	0	0
5652	1100647	STM1008	1.776	0.682	0	0
16156	1100831	STM1008	1.238	0.611	0	0
21018	1101128	STM1008	1.453	0.613	0	0
17268	1101208	STM1008	1.301	0.557	0	0
21877	1101426	STM1008, STM1009	1.312	0.604	0	0
3386	1101644	STM1008, STM1009	1.225	0.469	0	0
5862	1102039	STM1009	1.030	0.639	0	0
20433	1102110	STM1009	1.527	0.606	0	0
3969	1102358	STM1009	1.130	0.661	0	0
16066	1102751	STM1009	1.631	0.744	0	0
17512	1102967	STM1009	1.269	0.625	0	0
12922	1103136	STM1009	1.691	0.660	0	0
20162	1103445	STM1009	1.500	0.700	0	0
11771	1103602	STM1009	1.623	0.771	0	0

12153	1103874	STM1009	1.764	0.748	0	0
8162	1104090	STM1009	1.566	0.608	0	0
7987	1104265	STM1009, STM1010	1.946	0.668	0	0
14924	1104409	STM1009, STM1010	1.818	0.695	0	0
18315	1104660	STM1010	1.435	0.643	0	0
17841	1104933	STM1010, STM1011	0.706	0.759	0	0
16138	1110157	STM1020	1.197	1.034	0	0
1625	1110443	STM1020, STM1021	1.009	0.746	0	0
5411	1110652	STM1021	1.035	0.684	0	0
2380	1110768	STM1021	1.125	0.955	0	0
13952	1111149	STM1021, STM1022	1.279	0.688	0	0
7584	1111375	STM1022	1.654	0.720	0	0
7859	1111577	STM1022	1.443	0.607	0	0
1474	1111752	STM1022	1.365	0.753	0	0
6745	1111857	STM1022	0.993	0.713	0	0
1603	1112141	STM1022, STM1023	1.014	1.643	0	0
7006	1112253	STM1022, STM1023	1.043	1.891	0	0
15815	1112349	STM1023	0.925	1.881	0	0
19834	1112858	STM1024	1.044	0.854	0	0
10955	1113097	STM1025	1.211	0.736	0	0
720	1113302	STM1025	1.071	0.471	0	0
9402	1113559	STM1025	2.185	0.652	0	0
6255	1113843	STM1025	1.722	0.542	0	0
1711	1113995	STM1025, STM1026	1.583	0.655	0	0
17383	1114103	STM1026	1.616	0.751	0	0
15804	1114516	STM1026, STM1027	2.959	0.586	1	0
8693	1114599	STM1026, STM1027	2.994	0.541	1	0
17566	1114815	STM1026, STM1027	2.303	0.572	1	0
3215	1115179	STM1027, STM1028	2.117	0.633	0	0
22166	1115400	STM1028, STM1029	1.295	0.672	0	0
13106	1115640	STM1029	0.868	0.584	0	0
19445	1115729	STM1029	0.956	0.677	0	0
17997	1116108	STM1029, STM1030	0.858	0.621	0	0
15185	1116299	STM1030	1.033	0.581	0	0
17122	1116595	STM1030, STM1031	1.256	0.728	0	0
14240	1116767	STM1030, STM1031	1.274	0.841	0	0

13256	1116842	STM1030, STM1031	1.118	0.763	0	0
17349	1117266	STM1031	1.244	0.787	0	0
10850	1117361	STM1031	1.055	0.727	0	0
19174	1117651	STM1031	1.731	0.970	0	0
8291	1117732	STM1031	0.821	0.575	0	0
11993	1118187	STM1031	1.115	0.563	0	0
19255	1118279	STM1031	0.534	0.563	0	0
11114	1118463	STM1031	0.599	0.487	0	0
10286	1118896	STM1031	0.761	0.543	0	0
13151	1119040	STM1032	1.793	0.660	0	0
12080	1119169	STM1032	1.738	0.779	0	0
5172	1119267	STM1032	1.330	0.664	0	0
22003	1119709	STM1032	0.566	0.436	0	0
7598	1119845	STM1032	1.431	0.728	0	0
1257	1120126	STM1032	0.474	0.355	0	0
5312	1120291	STM1032, STM1033	1.071	0.631	0	0
6801	1120634	STM1032, STM1033	1.040	0.681	0	0
21023	1120837	STM1033	0.575	0.543	0	0
22216	1121135	STM1033	1.580	0.818	0	0
1035	1121260	STM1033	1.560	0.804	0	0
12564	1121496	STM1033	1.469	0.805	0	0
17820	1121641	STM1033	1.456	0.871	0	0
18167	1121952	STM1033	1.247	0.809	0	0
17168	1122231	STM1033	0.967	0.675	0	0
4573	1122427	STM1033, STM1034	0.652	0.557	0	0
6847	1122715	STM1033, STM1034	1.144	0.703	0	0
13045	1122823	STM1034, STM1035	1.382	0.794	0	0
20787	1123142	STM1035, STM1036	1.082	0.634	0	0
11604	1123414	STM1035, STM1036	1.625	0.606	0	0
4721	1123661	STM1036, STM1037	1.074	0.625	0	0
13948	1123738	STM1036, STM1037	1.126	0.624	0	0
20028	1123963	STM1036, STM1037	1.171	0.615	0	0
1570	1124363	STM1037, STM1038	1.481	0.675	0	0
20868	1124560	STM1038	1.230	0.607	0	0
20094	1124796	STM1038, STM1039	1.210	0.731	0	0
19983	1124872	STM1038, STM1039	1.433	0.734	0	0

9826	1125078	STM1038, STM1039	0.692	0.328	0	0
1821	1125271	STM1039, STM1040	1.798	0.731	0	0
9444	1125668	STM1040, STM1041	1.092	0.581	0	0
7659	1125899	STM1040, STM1041	1.304	0.690	0	0
8094	1126001	STM1041	1.019	0.582	0	0
4268	1126247	STM1041	1.576	0.858	0	0
6476	1126569	STM1041	0.469	0.457	0	0
21390	1126812	STM1041	1.313	0.609	0	0
17358	1126883	STM1041	1.630	0.812	0	0
391	1127236	STM1041	2.247	0.746	0	0
13017	1127434	STM1041	1.523	0.785	0	0
2169	1127687	STM1041	1.515	0.799	0	0
21362	1127988	STM1041	1.603	0.796	0	0
12329	1128136	STM1041	1.114	0.857	0	0
3601	1128215	STM1041	1.848	0.864	0	0
20950	1128673	STM1041, STM1042	0.982	0.711	0	0
6319	1128900	STM1041, STM1042	1.165	0.725	0	0
14318	1129101	STM1042, STM1043	1.253	0.709	0	0
11171	1129337	STM1043	0.870	0.595	0	0
19959	1129414	STM1043	0.795	0.714	0	0
14588	1129732	STM1043	1.397	0.715	0	0
9831	1129845	STM1043	1.191	0.700	0	0
1966	1130012		0.953	0.664	0	0
461	1130404	STM1045	1.205	0.801	0	0
3618	1130490	STM1045	1.040	0.668	0	0
11192	1130659	STM1045	1.276	0.753	0	0
508	1131078	STM1045, STM1046	1.295	0.780	0	0
21245	1131344	STM1046	1.130	0.775	0	0
16650	1131465	STM1046	1.214	0.820	0	0
6293	1131773	STM1046, STM1047	1.131	0.665	0	0
3024	1131958	STM1046, STM1047	1.018	0.777	0	0
8110	1132078	STM1046, STM1047	1.267	0.929	0	0
311	1132464	STM1047, STM1048	1.196	0.636	0	0
7449	1132701	STM1048	0.822	0.620	0	0
5746	1132815	STM1048	1.134	0.752	0	0
2109	1133216	STM1048	1.510	1.092	0	0

11259	1133376	STM1048	1.043	0.729	0	0
16944	1133509	STM1048	1.191	0.970	0	0
9814	1133763	STM1048	1.362	0.779	0	0
8277	1134021	STM1048	1.457	0.710	0	0
14092	1134259	STM1048	1.015	0.633	0	0
7453	1134486	STM1048	1.197	0.756	0	0
20313	1134639	STM1048	1.001	0.646	0	0
5232	1134939	STM1048	1.539	1.115	0	0
20289	1135228		2.258	0.776	0	0
4217	1135343		1.338	0.639	0	0
15967	1135668		1.617	0.759	0	0
7466	1135915		1.529	0.645	0	0
18384	1136143	STM1049	1.913	0.702	0	0
9531	1136255	STM1049	1.799	0.597	0	0
771	1136390	STM1049	1.951	0.567	0	0
19517	1136685	STM1049	2.215	0.710	0	0
2626	1136788	STM1049	2.063	0.668	0	0
16028	1137298	STM1049	1.197	0.599	0	0
5747	1137518	STM1049	1.663	0.644	0	0
14630	1137676	STM1049	0.675	0.615	0	0
1542	1137929	STM1049	2.136	0.865	0	0
7196	1138004	STM1049	2.028	0.774	0	0
4463	1138321	STM1049	0.924	0.683	0	0
20399	1138562	STM1049, STM1050	1.113	0.676	0	0
9040	1138903	STM1050	3.056	0.622	1	0
12360	1139058	STM1050	2.868	0.652	1	0
5424	1139226	STM1050	2.616	0.576	0	0
6966	1139526	sseI	2.454	0.511	0	0
4726	1139668	sseI	2.516	0.712	0	0
6135	1139767	sseI	2.588	0.639	0	0
16159	1140222	sseI	2.698	0.644	1	0
3017	1140369	sseI	3.005	0.540	1	0
6153	1140619	sseI	3.699	0.463	1	0
4611	1140831	sseI	4.301	0.500	1	0
6741	1140912		3.740	0.551	1	0
15158	1140983		2.602	0.653	1	0

20418	1141567	STM1053	1.087	0.718	0	0
3051	1141646	STM1053	0.924	0.723	0	0
18514	1141853	STM1053	1.525	0.897	0	0
16019	1142159	STM1053	2.490	0.687	1	0
9777	1142326	STM1053, STM1054	2.566	0.573	1	0
2658	1142758	STM1054, STM1055	2.465	0.424	1	0
2258	1142838	STM1054, STM1055	2.429	0.505	1	0
19507	1143141	STM1055	3.278	0.572	1	0
4370	1143438	STM1055	2.582	0.546	0	0
21187	1143543	STM1055, STM1056	2.105	0.626	0	0
4982	1143897	STM1056, pepN	1.188	0.634	0	0
18710	1144109	STM1056, pepN	0.957	0.630	0	0
4690	1144187	pepN	1.056	0.726	0	0
11759	1144557	pepN	1.198	0.769	0	0
17560	1144829	pepN	1.311	0.847	0	0
21135	1144914	pepN	1.257	0.769	0	0
13453	1145221	pepN	0.757	0.617	0	0
3055	1145387	pepN	1.089	0.906	0	0
6527	1145474	pepN	1.466	1.147	0	0
16853	1145584	pepN	1.427	1.063	0	0
1862	1146098	pepN	1.075	0.859	0	0
18617	1146341	pepN	0.778	0.674	0	0
11656	1146597	pepN, pyrD	1.143	0.948	0	0
2231	1146837	pyrD	1.258	0.970	0	0
6197	1146943	pyrD	1.201	1.054	0	0
14888	1147255	pyrD	1.755	0.815	0	0
5405	1147398	pyrD	2.710	0.725	1	0
11830	1147527	pyrD	3.054	0.789	1	0
2529	1147936	pyrD, ycbW	3.632	1.016	1	0
17163	1148090	ycbW	3.028	1.129	1	0
5482	1148301	ycbW	2.819	1.469	1	0
9594	1148635	ycbW	1.005	1.334	0	0
8481	1148815	STM1060	1.027	1.415	0	0
18554	1149138	STM1060	0.554	0.576	0	0
5625	1149226	STM1060	0.968	0.942	0	0
2083	1149578	STM1060, ycbY	1.024	1.307	0	0

5950	1149781	STM1060, ycbY	1.144	2.186	0	1
5761	1149852	STM1060, ycbY	1.054	2.002	0	1
18995	1150081	STM1060, ycbY	1.256	1.815	0	0
13362	1150185	STM1060, ycbY	1.114	1.638	0	0
14487	1150544	ycbY	0.852	1.196	0	0
11188	1150759	ycbY	1.047	1.225	0	0
11284	1151114	ycbY	1.065	1.153	0	0
4060	1151345	ycbY	1.660	1.341	0	0
18459	1151468	ycbY	1.205	1.176	0	0
18815	1151708	ycbY, uup	1.114	1.249	0	0
11059	1151988	uup	0.853	1.116	0	0
3516	1152272	uup	0.956	1.050	0	0
9560	1152371	uup	0.665	0.746	0	0
3589	1152669	uup	1.120	1.004	0	0
20231	1152974	uup	1.077	1.103	0	0
1965	1153192	uup	0.947	1.009	0	0
1222	1153422	uup	1.076	1.068	0	0
19730	1153624	uup, pqiA	1.042	1.183	0	0
15522	1153845	uup, pqiA	0.911	1.521	0	0
9073	1154096	pqiA	1.035	1.685	0	0
2395	1154225	pqiA	0.767	1.302	0	0
4568	1154316	pqiA	1.098	1.268	0	0
10501	1154735	pqiA, pqiB	1.079	1.141	0	0
18860	1154922	pqiA, pqiB	0.821	0.917	0	0
21303	1155165	pqiB	0.900	0.774	0	0
5588	1155278	pqiB	0.633	0.878	0	0
4148	1155449	pqiB	0.886	0.988	0	0
18524	1155812	pqiB	0.767	1.053	0	0
21700	1155990	pqiB	0.901	1.151	0	0
14089	1156132	pqiB	0.882	1.007	0	0
18909	1156451	pqiB, ymbA	0.621	0.780	0	0
18357	1156693	pqiB, ymbA	0.865	1.022	0	0
5083	1157003	ymbA	0.625	0.671	0	0
9277	1157157	ymbA, rmf	0.987	1.122	0	0
2063	1157468	rmf	0.716	1.013	0	0
12728	1157604	rmf	0.715	0.940	0	0

6466	1157855		0.739	1.390	0	0
21774	1158015	fabA	0.714	1.768	0	0
5562	1158359	fabA	0.549	1.480	0	0
8704	1158669	fabA, lonH	0.885	1.530	0	0
10133	1158764	fabA, lonH	0.829	1.406	0	0
14508	1159013	lonH	0.824	1.143	0	0
8433	1159084	lonH	0.747	1.094	0	0
20034	1159513	lonH	0.660	1.135	0	0
21696	1159598	lonH	0.698	1.278	0	0
7486	1159738	lonH	1.001	1.708	0	0
20694	1160102	lonH, ycbG	0.698	2.043	0	0
7353	1160413	lonH, ycbG	0.798	1.685	0	0
3651	1160620	lonH, ycbG	0.710	1.610	0	0
21064	1160852	ycbG	0.695	1.614	0	0
20024	1161117	ompA	0.537	2.274	0	0
4214	1161315	ompA	0.445	1.504	0	0
16421	1161505	ompA	0.522	2.420	0	1
15374	1161779	ompA	0.473	2.191	0	1
5538	1162030	ompA	0.552	2.188	0	1
6099	1162147	ompA	0.545	1.468	0	0
4665	1162223	ompA	0.269	0.529	0	0
15837	1162600	sulA, yccR	0.687	0.901	0	0
9972	1162834	sulA, yccR	0.878	0.739	0	0
653	1163131	sulA, yccR	0.916	0.795	0	0
11854	1163449	yccR	1.052	0.839	0	0
14703	1163546	yccR	0.941	0.835	0	0
6640	1163745	yccS	1.059	1.002	0	0
3025	1164130	yccS	0.612	0.847	0	0
20954	1164339	yccS	0.825	0.833	0	0
10999	1164429	yccS	1.073	0.938	0	0
21309	1164816	yccS	1.123	1.017	0	0
10489	1164888	yccS	1.026	0.936	0	0
8760	1165229	yccS	0.906	0.989	0	0
3598	1165432	yccS	1.112	0.921	0	0
17477	1165585	yccS	0.729	0.626	0	0
3889	1165800	yccS	1.130	0.836	0	0

4108	1166120	yccS, yccF	1.084	0.886	0	0
18011	1166356	yccF, helD	1.038	0.977	0	0
18991	1166523	yccF, helD	0.886	0.782	0	0
20574	1166622	yccF, helD	1.228	1.186	0	0
20625	1166870	helD	0.730	0.773	0	0
21327	1167270	helD	1.194	0.927	0	0
21302	1167417	helD	1.003	0.928	0	0
19271	1167691	helD	1.440	1.209	0	0
6819	1167984	helD	1.107	1.114	0	0
7589	1168065	helD	1.447	1.434	0	0
11392	1168407	helD	1.181	1.853	0	0
9910	1168555	mgsA	1.050	1.289	0	0
11019	1168889	mgsA	0.763	1.052	0	0
18368	1168960	mgsA	0.869	1.088	0	0
11348	1169300	mgsA, yccT	1.217	0.844	0	0
4622	1169450	yccT, STM1078	1.229	0.842	0	0
14562	1169815	yccT, STM1078	0.840	0.912	0	0
18603	1169901	yccT, STM1078	0.749	0.974	0	0
21974	1170106	yccT, STM1078	0.835	1.470	0	0
10067	1170436	yccV	0.488	0.964	0	0
19138	1170588	yccV	0.474	0.880	0	0
11948	1170871	yccV, yccW	1.021	1.622	0	0
19998	1171004	yccV, yccW	1.079	1.458	0	0
5314	1171348	yccW	1.169	0.983	0	0
876	1171549	yccW	1.091	0.870	0	0
827	1171685	yccW, STM1081	1.473	0.903	0	0
15653	1171920	yccW, STM1081	1.289	1.038	0	0
6629	1172129	yccW, STM1081	1.513	1.055	0	0
17326	1172278	yccW, STM1081	1.263	1.036	0	0
1744	1172711	STM1082	1.100	0.974	0	0
14535	1172974	STM1082	1.492	0.950	0	0
15872	1173060	STM1082	0.697	0.672	0	0
19160	1173329	STM1082, yccX	1.113	1.093	0	0
11510	1173534	yccX	0.929	1.275	0	0
4024	1173804	yccX	0.607	0.779	0	0
18471	1174134	yccK	0.848	1.873	0	1

15820	1174369	yccK, yccA	0.798	1.981	0	1
19922	1174525	yccK, yccA	0.730	1.928	0	1
18313	1174712	yccA	0.470	1.154	0	0
4511	1174888	yccA, serT	0.613	1.842	0	0
15519	1175000	yccA, serT	0.581	1.842	0	0
19898	1175230	yccA, serT	0.453	2.363	0	0
9743	1175531	pipA	0.611	2.325	0	0
405	1175918	pipA	1.634	1.385	0	0
10280	1176119	pipA	1.908	0.774	0	0
13015	1176292	pipA	2.256	0.632	0	0
21055	1176460	pipA, pipB	1.979	0.708	0	0
1361	1176659	pipA, pipB	2.073	0.529	0	0
15120	1176854	pipB	2.001	0.576	0	0
19706	1177130	pipB, STM1089	2.049	0.506	0	0
2375	1177294	pipB, STM1089	2.011	0.548	0	0
11796	1177744	pipB, STM1089	2.241	0.460	0	0
16950	1177872	pipC	2.269	0.440	0	0
18579	1178065	pipC	1.880	0.471	0	0
20785	1178449	pipC, sopB	2.775	0.594	1	0
15503	1178634	pipC, sopB	2.799	0.545	1	0
5954	1178890	sopB	2.328	0.541	1	0
5260	1179093	sopB	1.992	0.576	0	0
11910	1179185	sopB	1.302	0.580	0	0
772	1179514	sopB	1.289	0.587	0	0
17930	1179809	sopB, orfX	1.547	0.444	0	0
3053	1179887	sopB, orfX	1.297	0.535	0	0
2653	1180164	sopB, orfX	2.056	0.555	0	0
2556	1180447	STM1093	2.396	0.545	0	0
18482	1180664	STM1093, pipD	2.431	0.557	0	0
20126	1180807	STM1093, pipD	1.808	0.550	0	0
10317	1181092	pipD	1.910	0.635	0	0
4993	1181243	pipD	2.098	0.719	0	0
13344	1181397	pipD	2.155	0.817	0	0
3488	1181539	pipD	1.889	0.787	0	0
16551	1181904	pipD	1.502	0.924	0	0
22323	1182116	pipD	1.514	0.881	0	0

4637	1182509	pipD, copS	2.319	0.809	1	0
15103	1182634	copS	2.117	0.931	1	0
20119	1182754	copS	1.924	0.868	0	0
18928	1182918	copS	1.460	0.674	0	0
12339	1183383	copS	1.399	0.866	0	0
14930	1183662	copS, copR	1.223	0.799	0	0
5226	1183736	copS, copR	1.313	0.817	0	0
6427	1183898	copS, copR	1.249	0.899	0	0
7715	1184328	copR, STM1097	0.885	0.748	0	0
11477	1184439	copR, STM1097	1.342	0.879	0	0
2499	1184756	copR, STM1097	1.890	1.042	0	0
21480	1184827	STM1097	1.990	1.073	0	0
5508	1185195	hpaC	1.674	0.944	0	0
17104	1185274	hpaC	0.721	0.578	0	0
2307	1185689	hpaC	1.114	0.601	0	0
17994	1185789	hpaC, hpaB	0.974	0.547	0	0
4084	1185954	hpaC, hpaB	1.273	0.689	0	0
9875	1186065	hpaC, hpaB	1.224	0.616	0	0
4239	1186433	hpaB	1.599	0.630	0	0
6866	1186810	hpaB	1.199	0.704	0	0
1250	1187066	hpaB	1.311	0.702	0	0
15031	1187216	hpaB	1.457	0.714	0	0
17876	1187339	hpaB	1.239	0.709	0	0
4904	1187648	hpaB, hpaR	0.878	0.751	0	0
9323	1187986	hpaR, hpaG	1.061	0.587	0	0
19075	1188179	hpaR, hpaG	0.804	0.537	0	0
19093	1188345	hpaR, hpaG	2.429	0.706	0	0
21110	1188600	hpaG	0.921	0.597	0	0
6367	1188787	hpaG	1.382	0.696	0	0
4119	1188951	hpaG	0.904	0.637	0	0
5711	1189320	hpaG, hpaE	1.218	0.546	0	0
16411	1189518	hpaE	1.249	0.721	0	0
18413	1189821	hpaE	1.524	0.742	0	0
14409	1189931	hpaE	1.312	0.664	0	0
8234	1190192	hpaE	1.467	0.711	0	0
4406	1190459	hpaE	1.059	0.619	0	0

22217	1190689	hpaE, hpaD	1.174	0.734	0	0
19712	1190889	hpaE, hpaD	1.707	0.798	0	0
13323	1190989	hpaD	1.631	0.859	0	0
20580	1191376	hpaD, hpaF	1.367	0.842	0	0
16192	1191462	hpaD, hpaF	1.414	0.802	0	0
12621	1191821	hpaF	1.054	0.514	0	0
11025	1191969	hpaF, hpaH	0.582	0.436	0	0
17648	1192169	hpaF, hpaH	1.341	0.838	0	0
10854	1192454	hpaH	1.641	0.642	0	0
22027	1192634	hpaH	1.137	0.779	0	0
19837	1192793	hpaH, hpaI	0.871	0.660	0	0
407	1193139	hpaI	1.954	0.607	0	0
12046	1193369	hpaI	1.261	0.483	0	0
20796	1193629	hpaI, hpaX	5.205	0.751	0	0
2291	1193801	hpaI, hpaX	0.798	0.483	0	0
1880	1193996	hpaX	1.691	0.746	0	0
4152	1194132	hpaX	1.400	0.762	0	0
6344	1194597	hpaX	1.151	0.560	0	0
8111	1194829	hpaX	1.209	0.677	0	0
6056	1194900	hpaX	1.155	0.644	0	0
5807	1195102	hpaX, hpaA	1.093	0.731	0	0
3546	1195445	hpaA	1.233	0.732	0	0
1748	1195547	hpaA	1.117	0.911	0	0
10623	1195674	hpaA	1.318	1.137	0	0
15305	1195981	hpaA, STM1109	1.413	1.686	0	0
15511	1196287	hpaA, STM1109	1.543	0.944	0	0
15478	1196581	STM1109	0.630	0.720	0	0
6765	1196714	STM1109	1.288	0.882	0	0
9390	1196994	STM1109	1.484	0.703	0	0
15358	1197102	STM1109	1.770	0.763	0	0
5406	1197231	STM1109	2.127	0.712	0	0
3785	1197703	STM1110	2.432	0.416	0	0
3004	1197974	STM1110	2.555	0.491	0	0
1310	1198090	STM1110	2.512	0.535	0	0
21137	1198361	STM1110	2.703	0.555	1	0
636	1198572		3.105	0.857	1	0

12084	1198682	yccD	2.537	0.753	1	0
5634	1198954	yccD	1.024	0.736	0	0
5026	1199098	yccD, cbpA	0.967	0.792	0	0
20436	1199585	cbpA	0.921	0.906	0	0
21267	1199770	cbpA, scsA	1.002	1.188	0	0
729	1199924	cbpA, scsA	1.155	1.155	0	0
3220	1200007	cbpA, scsA	1.392	1.216	0	0
15984	1200160	cbpA, scsA	0.375	0.665	0	0
10396	1200476	scsA, scsB	1.014	1.377	0	0
637	1200557	scsB	0.796	1.380	0	0
5013	1200946	scsB	0.971	0.996	0	0
9513	1201152	scsB	1.513	1.036	0	0
4510	1201473	scsB	1.649	0.822	0	0
14677	1201692	scsB	1.313	0.849	0	0
7586	1201974	scsB, scsC	0.602	0.562	0	0
22314	1202174	scsB, scsC	1.390	0.634	0	0
4285	1202414	scsB, scsC	1.757	0.606	0	0
11202	1202662	scsC, scsD	1.477	0.663	0	0
2206	1202808	scsC, scsD	1.590	0.659	0	0
12012	1202896	scsC, scsD	1.238	0.743	0	0
20115	1203016	scsC, scsD	1.051	0.639	0	0
742	1203642	agp	1.493	0.824	0	0
11017	1203765	agp	1.145	0.962	0	0
7238	1204094	agp	0.966	0.819	0	0
14283	1204219	agp	1.480	1.033	0	0
12850	1204373	agp	1.144	0.969	0	0
21300	1204476	agp	1.262	1.064	0	0
5528	1204888	agp	0.984	0.995	0	0
5463	1205022	yccJ	0.941	1.037	0	0
13832	1205180	yccJ	1.190	1.174	0	0
16612	1205254	yccJ, wraB	1.014	1.019	0	0
10789	1205799	wraB, ymdF	1.385	0.619	0	0
16197	1206014	wraB, ymdF	1.436	0.753	0	0
6428	1206138	wraB, ymdF	1.512	0.874	0	0
3359	1206444	ycdC	1.093	1.106	0	0
1562	1206761	ycdC	0.751	2.156	0	1

9647	1207068	ycdC	0.642	3.328	0	1
4888	1207178	STM1123	0.550	2.668	0	1
13977	1207518	STM1123	0.911	2.886	0	1
9974	1207635	STM1123	0.593	1.922	0	0
16753	1207953	STM1123	0.709	2.717	0	1
10295	1208139		0.819	3.364	0	1
16354	1208414	putA	0.781	2.790	0	1
10243	1208498	putA	0.873	3.266	0	1
9638	1208623	putA	0.664	2.808	0	1
4799	1208747	putA	0.535	1.528	0	0
11428	1209266	putA	0.799	2.897	0	0
19071	1209489	putA	0.420	1.268	0	0
16964	1209638	putA	0.625	1.843	0	0
3366	1209938	putA	0.615	2.228	0	1
11828	1210136	putA	0.774	2.632	0	1
9654	1210299	putA	0.704	2.763	0	1
5731	1210378	putA	0.665	2.344	0	1
7350	1210681	putA	0.683	3.022	0	1
7510	1210956	putA	0.621	2.431	0	1
2455	1211288	putA	0.696	2.320	0	1
7279	1211549	putA, putP	0.593	1.293	0	0
5519	1211765	putA, putP	0.823	1.380	0	0
6725	1211973	putA, putP	0.733	1.493	0	0
4176	1212282	putP	0.669	1.571	0	0
9383	1212487	putP	0.744	1.393	0	0
5654	1212639	putP	0.831	1.857	0	1
11870	1212897	putP	0.765	1.989	0	1
4018	1213001	putP	0.695	1.539	0	0
13058	1213298	putP	0.465	0.752	0	0
19205	1213568		0.918	1.177	0	0
15380	1213696		1.005	1.341	0	0
9704	1213953	phoH	0.729	0.785	0	0
9319	1214353	phoH	0.678	1.067	0	0
2204	1214555	phoH	0.654	0.928	0	0
8775	1214715	phoH	0.790	1.078	0	0
19469	1214851	phoH	0.782	0.994	0	0

16617	1215062	phoH	0.730	0.846	0	0
20125	1215224		0.711	1.016	0	0
5866	1215425	STM1127	0.890	1.193	0	0
17167	1215740	STM1127	0.826	1.025	0	0
6368	1215919	STM1127	1.030	1.122	0	0
12753	1216261	STM1127	0.836	0.898	0	0
417	1216404	STM1127	1.098	1.015	0	0
11402	1216685	STM1128	1.941	0.819	0	0
4875	1216860	STM1128	1.962	0.608	0	0
11261	1217188	STM1128	2.230	0.580	0	0
12109	1217370	STM1128	2.598	0.779	1	0
2357	1217665	STM1128	2.750	0.807	1	0
6083	1217816	STM1128	2.675	0.804	1	0
2168	1217945	STM1128	1.523	0.787	0	0
7760	1218222	STM1128	1.854	0.825	0	0
20293	1218478	STM1129	1.038	0.681	0	0
1392	1218714	STM1129	1.432	0.908	0	0
4854	1219000	STM1129, STM1130	2.077	0.630	0	0
3485	1219320	STM1129, STM1130	2.106	0.420	0	0
19968	1219526	STM1130	2.154	0.558	0	0
16338	1219687	STM1130	2.514	0.584	1	0
12371	1219797	STM1130	2.596	0.544	1	0
19597	1220149	STM1130	2.403	0.636	0	0
17146	1220339	STM1130, STM1131	2.181	0.611	1	0
4722	1220544	STM1130, STM1131	2.410	0.539	1	0
10816	1220824	STM1131	2.238	0.528	0	0
20783	1221048	STM1131	2.511	0.384	0	0
4237	1221306	STM1131, STM1132	1.923	0.463	0	0
2532	1221415	STM1132	1.620	0.418	0	0
7799	1221659	STM1132	1.923	0.526	0	0
14671	1222068	STM1132	1.728	0.651	0	0
5707	1222256	STM1132	2.097	0.494	0	0
14720	1222479	STM1132, STM1133	2.367	0.581	0	0
1521	1222676	STM1132, STM1133	2.030	0.599	0	0
14233	1222833	STM1132, STM1133	2.230	0.585	0	0
20650	1223206	STM1133	1.364	0.523	0	0

17626	1223433	STM1133	0.908	0.742	0	0
17893	1223530	STM1133	0.939	0.839	0	0
1341	1223857	STM1133	1.004	0.854	0	0
9727	1224010		0.455	0.720	0	0
11134	1224308		0.695	1.456	0	0
5558	1224492		0.678	1.349	0	0
5078	1224675	ycdW	0.779	1.694	0	0
11585	1224919	serX, ycdW	0.689	2.350	0	1
11195	1225032	serX, ycdW	0.811	1.673	0	1
1900	1225357	ycdW	0.900	0.893	0	0
15333	1225660	ycdW, ycdX	1.142	1.116	0	0
443	1225781	ycdW, ycdX	1.223	1.222	0	0
18528	1226045	ycdX	0.892	1.147	0	0
3570	1226330	ycdX	1.043	1.310	0	0
13108	1226458	ycdX, ycdY	1.128	1.156	0	0
5202	1226838	ycdY	0.628	0.701	0	0
8839	1227001	ycdY, ycdZ	0.928	0.869	0	0
21001	1227244	ycdY, ycdZ	0.811	0.956	0	0
11599	1227351	ycdY, ycdZ	0.456	0.492	0	0
12434	1227759	ycdZ	0.876	1.095	0	0
18027	1227947	ycdZ	0.976	0.887	0	0
15874	1228038	csgG	0.933	0.670	0	0
2475	1228160	csgG	0.586	0.623	0	0
11489	1228527	csgG	1.661	0.611	0	0
3251	1228869	csgG, csgF	1.817	0.604	0	0
15253	1228980	csgG, csgF	2.529	0.664	1	0
16693	1229186	csgG, csgF	2.320	0.564	1	0
4747	1229549	csgF, csgE	2.022	0.506	0	0
9797	1229829	csgE, csgD	2.264	0.496	1	0
18716	1229927	csgE, csgD	2.515	0.500	1	0
7274	1230190	csgD	2.445	0.549	0	0
8044	1230306	csgD	2.422	0.478	0	0
9672	1230421	csgD	1.996	0.464	0	0
7795	1230756	csgD, csgB	1.718	0.473	0	0
4766	1230872	csgB	1.212	0.459	0	0
17111	1231075	csgB	2.404	0.512	1	0

11698	1231503	csgB, csgA	2.654	0.545	1	0
12575	1231602	csgA	2.621	0.632	1	0
6287	1231868	csgA, csgC	1.209	0.524	0	0
14489	1232172	csgC, ymdA	1.879	1.049	0	0
1512	1232382	csgC, ymdA	1.565	1.108	0	0
15796	1232592	ymdA, STM1147	1.415	1.157	0	0
7073	1232941	STM1147	1.166	1.188	0	0
17925	1233018	STM1147, ymdC	0.948	1.213	0	0
21626	1233444	STM1147, ymdC	0.930	1.310	0	0
11496	1233663	ymdC	1.009	1.341	0	0
19664	1233836	ymdC	0.974	1.972	0	0
19786	1234109	ymdC	1.056	1.254	0	0
609	1234215	ymdC	0.977	1.247	0	0
3268	1234441	ymdC	0.880	1.050	0	0
6711	1234700	ymdC	1.094	0.922	0	0
10310	1234849	ymdC	1.359	0.987	0	0
4324	1235166	mdoC	1.780	0.956	0	0
14049	1235270	mdoC	1.208	0.947	0	0
16059	1235482	mdoC	1.453	0.710	0	0
1995	1235856	mdoC	1.102	0.820	0	0
16406	1236034	mdoC, mdoG	0.906	0.945	0	0
16241	1236300	mdoC, mdoG	0.682	1.619	0	1
16478	1236371	mdoC, mdoG	0.703	1.697	0	1
6009	1236817	mdoG	0.705	1.258	0	0
7784	1236992	mdoG	0.742	1.465	0	0
2146	1237287	mdoG	0.691	1.617	0	0
9197	1237450	mdoG, mdoH	0.796	1.404	0	0
8105	1237758	mdoG, mdoH	0.685	1.338	0	0
5532	1237904	mdoG, mdoH	0.754	1.551	0	0
16846	1238126	mdoH	0.547	1.047	0	0
18595	1238406	mdoH	0.572	1.317	0	0
676	1238500	mdoH	0.521	1.750	0	0
1426	1238774	mdoH	0.976	1.944	0	0
6166	1238952	mdoH	0.587	0.915	0	0
17721	1239166	mdoH	0.567	1.432	0	0
22137	1239409	mdoH	0.436	0.912	0	0

15193	1239687	mdoH	0.918	1.896	0	0
19181	1239840	mdoH	0.303	0.635	0	0
17771	1240101	mdoH, yceK	0.778	1.453	0	0
1602	1240397	mdoH, yceK	0.827	1.644	0	0
20501	1240543	yceK	0.878	1.305	0	0
12577	1240847	msyB	0.917	1.411	0	0
21718	1241147	msyB	1.054	0.907	0	0
2542	1241247	msyB, yceE	0.950	0.729	0	0
9844	1241543	msyB, yceE	1.120	0.792	0	0
20909	1241677	yceE	1.064	0.848	0	0
9072	1241871	yceE	1.453	0.872	0	0
12010	1242236	yceE	1.173	0.983	0	0
15214	1242408	yceE	0.922	0.958	0	0
13600	1242536	yceE	0.870	0.932	0	0
21917	1242960	htrB	0.666	1.124	0	0
9014	1243053	htrB	0.759	1.287	0	0
11073	1243325	htrB, yceA	0.800	1.324	0	0
19884	1243502	htrB, yceA	0.775	1.513	0	0
21745	1243650	htrB, yceA	0.746	1.345	0	0
7504	1243723	htrB, yceA	0.553	1.010	0	0
22225	1244324	yceA	0.975	1.017	0	0
2786	1244486	yceA	1.253	1.004	0	0
7980	1244733	yceA	0.993	1.163	0	0
11843	1244918	yceI	0.993	1.268	0	0
6499	1245198	yceI	0.798	0.630	0	0
11299	1245408	yceI, STM1158	0.955	0.546	0	0
7286	1245665	yceI, STM1158	1.251	0.609	0	0
12946	1245878	STM1158	1.442	0.653	0	0
614	1246101	STM1158	1.700	0.641	0	0
16369	1246238	STM1158, yceO	2.181	0.685	0	0
11235	1246351	STM1158, yceO	1.741	0.795	0	0
13833	1246559	yceO, solA	1.210	0.664	0	0
3678	1246969	solA	0.691	0.828	0	0
18925	1247085	solA	1.074	0.912	0	0
8279	1247512	solA	1.304	1.495	0	0
3966	1247722	solA, yceP	1.137	1.476	0	0

16256	1247913	solA, yceP	1.339	1.428	0	0
7468	1248179	yceP, dinI	1.015	1.058	0	0
12846	1248406	dinI	0.970	1.070	0	0
13298	1248490	dinI, pyrC	0.910	1.113	0	0
20261	1248855	dinI, pyrC	0.556	0.666	0	0
20695	1249109	pyrC	0.752	1.255	0	0
7871	1249180	pyrC	0.640	1.061	0	0
7767	1249576	pyrC	0.903	1.239	0	0
1119	1249676	pyrC, yceB	0.824	1.320	0	0
10878	1249928	pyrC, yceB	0.766	1.346	0	0
13071	1250126	yceB	0.738	1.152	0	0
21153	1250355	yceB, grxB	0.895	1.228	0	0
3968	1250585	yceB, grxB	0.902	1.049	0	0
7915	1250806	grxB	0.793	0.981	0	0
11410	1250961	grxB	0.942	0.915	0	0
8667	1251103	grxB, yceL	0.776	0.763	0	0
5877	1251331	grxB, yceL	1.064	0.867	0	0
21593	1251837	yceL	0.758	0.699	0	0
5552	1251944	yceL	0.649	0.536	0	0
21524	1252201	yceL, rimJ	1.278	1.228	0	0
5117	1252460	yceL, rimJ	0.933	0.825	0	0
12270	1252623	yceL, rimJ	1.044	1.135	0	0
7532	1252978	rimJ, yceH	1.070	0.954	0	0
22228	1253094	yceH	1.017	1.118	0	0
12772	1253356	yceH, mviM	1.019	1.110	0	0
9033	1253677	yceH, mviM	1.328	1.357	0	0
8436	1253748	mviM	1.023	1.482	0	0
16068	1253858	mviM	0.814	1.189	0	0
1139	1254240	mviM	0.879	1.073	0	0
16862	1254516	mviM, mviN	0.822	1.234	0	0
4101	1254816	mviN	0.685	1.132	0	0
12943	1254940	mviN	0.933	1.275	0	0
10705	1255177	mviN	1.116	1.148	0	0
15118	1255386	mviN	0.969	1.178	0	0
7303	1255590	mviN	0.794	0.919	0	0
13932	1255719	mviN	0.886	1.364	0	0

17053	1256124	mviN	0.922	2.010	0	1
20795	1256292	mviN	0.630	2.264	0	1
7472	1256388	mviN	0.574	2.581	0	1
6028	1256611	flgN	0.930	2.615	0	1
11936	1257002	flgN	0.507	2.295	0	1
8463	1257290	flgN, flgM	0.526	2.399	0	1
15896	1257438	flgN, flgM	0.651	2.887	0	1
13631	1257537	flgM, flgA	0.520	2.457	0	1
6906	1257650	flgM, flgA	0.535	2.448	0	1
377	1258067	flgA, flgB	0.618	3.553	0	1
2552	1258368	flgA, flgB	0.541	0.948	0	0
8186	1258495	flgA, flgB	0.650	2.375	0	1
7153	1258604	flgB, flgC	0.653	2.475	0	1
8621	1259017	flgC, flgD	0.695	2.024	0	1
10053	1259205	flgD	0.548	1.579	0	0
22084	1259568	flgD, flgE	0.635	1.958	0	1
13913	1259749	flgD, flgE	0.656	2.202	0	1
11208	1259848	flgE	0.596	2.001	0	1
19868	1260033	flgE	0.669	2.209	0	1
20432	1260371	flgE	0.592	1.474	0	0
9501	1260487	flgE	0.581	1.408	0	0
19437	1260578	flgE, flgF	0.380	1.111	0	0
10290	1260941	flgE, flgF	0.646	2.249	0	1
20570	1261325	flgF, flgG	0.852	2.418	0	1
7330	1261517	flgF, flgG	0.671	2.004	0	1
20316	1261788	flgG	0.751	2.299	0	1
9549	1261859	flgG	0.563	1.715	0	0
5362	1262069	flgG	0.545	1.252	0	0
20888	1262524	flgG, flgH	0.768	1.822	0	1
6130	1262622	flgH	0.809	2.139	0	1
20621	1262946	flgH, flgI	0.881	1.883	0	0
11537	1263116	flgH, flgI	0.750	1.754	0	1
21402	1263335	flgI	0.682	2.327	0	1
21249	1263514	flgI	0.628	2.254	0	1
19963	1263900	flgI	0.781	1.818	0	1
18197	1264065	flgI, flgJ	0.873	1.951	0	1

18367	1264188	flgI, flgJ	0.388	1.074	0	0
9116	1264577	flgJ	0.659	2.291	0	1
18454	1264791	flgJ	0.677	2.154	0	1
21205	1264926	flgJ	0.717	2.052	0	1
12950	1265137	flgJ, flgK	0.696	2.382	0	1
9292	1265427	flgK	0.707	2.176	0	1
11380	1265625	flgK	0.641	2.202	0	1
14831	1265886	flgK	0.636	2.168	0	1
5637	1265967	flgK	0.587	1.896	0	1
7317	1266311	flgK	0.664	1.999	0	1
8735	1266549	flgK	0.601	1.699	0	1
9834	1266715	flgK, flgL	0.597	1.871	0	1
8517	1266973	flgK, flgL	0.534	1.959	0	1
11700	1267115	flgL	0.577	2.137	0	1
4398	1267187	flgL	0.572	2.116	0	1
658	1267721	flgL	0.649	2.494	0	1
20836	1267959	flgL	0.583	2.101	0	1
5585	1268059	flgL	0.713	2.131	0	1
13053	1268223		0.619	1.500	0	0
17921	1268619	rne	0.501	1.586	0	0
6169	1268854	rne	0.484	1.544	0	0
19673	1268986	rne	0.699	2.109	0	0
8261	1269332	rne	0.644	1.645	0	0
9825	1269427	rne	0.498	1.466	0	0
5729	1269797	rne	0.936	9.621	0	0
9174	1269877	rne	0.479	1.138	0	0
18587	1270222	rne	0.480	1.872	0	1
22326	1270385	rne	0.909	2.182	0	1
10329	1270633	rne	0.740	1.726	0	0
3187	1270838	rne	0.635	1.890	0	1
18171	1271063	rne	0.566	1.996	0	1
7786	1271232	rne	0.594	2.102	0	1
17363	1271401	rne	0.548	2.599	0	1
1352	1271728	rne, rluC	0.463	2.096	0	1
1424	1271849	rne, rluC	0.469	1.897	0	0
1487	1272210	rluC	0.797	1.360	0	0

8844	1272397	rluC	0.681	1.359	0	0
12097	1272676	rluC	0.614	1.152	0	0
20588	1272853	rluC, STM1188	0.643	1.201	0	0
12842	1273103	STM1188	0.599	0.964	0	0
14013	1273225	STM1188	0.889	1.181	0	0
5188	1273514	STM1188	0.899	0.834	0	0
15292	1273631	STM1188	0.779	0.755	0	0
18379	1274063		0.816	0.843	0	0
257	1274147		1.099	1.246	0	0
3463	1274412		0.845	1.100	0	0
16578	1274645	yceF	0.916	1.396	0	0
9624	1274840	yceF, yceD	0.778	1.595	0	0
19297	1275124	yceF, yceD	0.547	2.910	0	0
10527	1275377	yceF, yceD	0.378	1.852	0	0
15024	1275502	yceF, yceD	0.399	2.406	0	1
3954	1275817	rpmF, plsX	0.452	2.666	0	1
10654	1276054	plsX	0.475	1.722	0	0
2267	1276251	plsX	0.430	3.553	0	1
15595	1276577	plsX	0.564	2.063	0	1
14860	1276726	plsX	0.593	2.242	0	1
12388	1276856	plsX, fabH	0.416	1.116	0	0
8360	1277287	fabH	0.634	1.980	0	1
20272	1277376	fabH	0.616	1.956	0	1
3121	1277558	fabH	0.609	1.827	0	1
17731	1277799	fabH, fabD	0.636	2.242	0	1
12712	1277871	fabH, fabD	0.416	1.073	0	0
6495	1278351	fabD	0.625	2.482	0	1
20493	1278671	fabD	0.630	1.833	0	1
10063	1278889	fabD, fabG	0.546	1.651	0	1
17929	1279086	fabD, fabG	0.537	1.824	0	1
8404	1279345	fabG	0.545	1.734	0	1
1100	1279509	fabG	0.475	1.919	0	1
16330	1279591	fabG	0.419	1.458	0	0
16155	1279936	acpP, fabF	0.483	2.321	0	1
6929	1280022	acpP, fabF	0.466	2.639	0	1
20815	1280250	acpP, fabF	0.420	3.410	0	1

11687	1280380	fabF	0.352	3.034	0	1
19917	1280542	fabF	0.412	2.664	0	1
17305	1280829	fabF	0.313	0.762	0	0
1072	1281189	fabF	0.990	2.118	0	0
21360	1281609	fabF, pabC	0.753	1.479	0	0
2528	1281752	pabC	0.568	0.968	0	0
19153	1281948	pabC	0.831	1.600	0	0
1318	1282081	pabC	0.483	1.118	0	0
6560	1282199	pabC, yceG	0.718	1.445	0	0
5934	1282600	yceG	0.501	0.946	0	0
16839	1282690	yceG	0.912	1.383	0	0
593	1283206	yceG, tmk	0.880	1.426	0	0
2723	1283333	yceG, tmk	0.792	1.601	0	0
433	1283576	yceG, tmk	1.838	1.372	0	0
15887	1283758	tmk	0.663	0.828	0	0
12741	1284009	tmk, holB	0.623	1.082	0	0
2176	1284254	holB	1.213	1.751	0	0
4575	1284378	holB	0.939	1.566	0	0
1846	1284765	holB	1.154	1.476	0	0
4425	1284918	holB, ycfH	0.978	1.226	0	0
10788	1285124	holB, ycfH	1.104	1.311	0	0
16958	1285240	ycfH	0.670	0.869	0	0
4448	1285480	ycfH	1.048	1.269	0	0
4776	1285780	ycfH	0.811	0.963	0	0
13080	1286141	ptsG	0.722	1.016	0	0
10646	1286322	ptsG	0.718	0.776	0	0
20461	1286424	ptsG	0.585	0.739	0	0
11420	1286749	ptsG	0.839	0.981	0	0
22050	1286926	ptsG	1.072	1.296	0	0
21965	1287252	ptsG	1.034	1.128	0	0
11185	1287455	ptsG	0.985	0.899	0	0
10995	1287728	ptsG	0.447	0.291	0	0
20507	1287980	fhuE	1.215	0.719	0	0
21834	1288171	fhuE	1.037	0.864	0	0
6447	1288439	fhuE	1.322	0.819	0	0
15957	1288523	fhuE	1.280	0.794	0	0

20312	1288600	fhuE	0.837	0.579	0	0
14310	1289034	fhuE	0.943	0.674	0	0
7516	1289250	fhuE	0.830	0.608	0	0
21278	1289493	fhuE	0.840	0.525	0	0
18888	1289638	fhuE	0.963	0.608	0	0
11384	1289731	fhuE	0.889	0.772	0	0
17963	1290069	fhuE, ycfF	0.799	0.651	0	0
21102	1290400	fhuE, ycfF	1.089	1.004	0	0
20654	1290621	ycfF, ycfL	1.090	1.017	0	0
14273	1290746	ycfL, ycfM	0.833	1.108	0	0
370	1290948	ycfL, ycfM	1.202	1.357	0	0
13971	1291127	ycfM	1.153	1.463	0	0
8495	1291567	ycfM, ycfN	0.948	1.238	0	0
12252	1291740	ycfM, ycfN	0.848	1.209	0	0
14118	1292006	ycfN	0.464	0.778	0	0
7801	1292270	ycfN, nagZ	1.234	1.816	0	0
10447	1292459	ycfN, nagZ	0.807	1.093	0	0
4992	1292578	nagZ	1.375	0.918	0	0
7681	1292897	nagZ	0.368	0.492	0	0
15393	1293179	nagZ, ycfP	0.916	1.244	0	0
6314	1293406	nagZ, ycfP	0.835	1.078	0	0
18768	1293607	ycfP	0.854	1.200	0	0
13338	1293703	ycfP	0.956	1.267	0	0
18092	1294099	ycfP, ndh	0.767	0.809	0	0
5628	1294271	ndh	1.112	0.735	0	0
15621	1294342	ndh	0.303	0.161	0	0
15364	1294428	ndh	0.827	0.542	0	0
11320	1294704	ndh	0.882	0.646	0	0
19611	1295013	ndh	0.874	0.654	0	0
11949	1295285	ndh	1.077	0.747	0	0
11770	1295527	ndh, ycfJ	0.360	0.530	0	0
15976	1295865	ycfJ	1.136	0.966	0	0
1052	1296011	ycfJ	1.142	1.252	0	0
17197	1296318	ycfJ	0.919	1.613	0	0
4672	1296443	ycfJ	0.856	1.590	0	0
6942	1296635	ycfQ	0.980	1.752	0	0

8654	1296912	ycfQ	0.792	1.305	0	0
13102	1297189	ycfQ, ycfR	1.092	1.465	0	0
6633	1297395	ycfQ, ycfR	0.911	0.993	0	0
7781	1297645	ycfR	1.037	0.758	0	0
9994	1297901	ycfS	0.332	0.522	0	0
16006	1298017	ycfS	0.906	0.750	0	0
16167	1298386	ycfS	1.410	0.912	0	0
13983	1298507	ycfS	1.065	0.569	0	0
5345	1298780	ycfS	0.576	0.726	0	0
16879	1298928	ycfS, mfd	1.128	1.258	0	0
21936	1299253	mfd	1.183	1.302	0	0
14055	1299387	mfd	1.183	1.311	0	0
7769	1299683	mfd	0.854	0.993	0	0
5204	1299925	mfd	0.714	0.845	0	0
20772	1300181	mfd	1.246	1.269	0	0
12380	1300357	mfd	0.764	0.808	0	0
17310	1300551	mfd	1.055	1.051	0	0
16469	1300745	mfd	0.809	1.000	0	0
1857	1300992	mfd	0.530	0.532	0	0
11520	1301162	mfd	1.218	1.119	0	0
3775	1301495	mfd	1.317	1.211	0	0
22258	1301746	mfd	1.257	1.346	0	0
21266	1302069	mfd, ycfU	0.814	1.411	0	0
5432	1302154	mfd, ycfU	0.882	1.424	0	0
14548	1302315	mfd, ycfU	0.776	1.477	0	0
12072	1302632	mfd, ycfU	0.415	0.927	0	0
5265	1302846	ycfU	0.733	1.977	0	0
22086	1303057	ycfU	0.466	0.902	0	0
16531	1303234	ycfU	1.012	1.992	0	0
10514	1303514	ycfU, ycfV	0.504	0.803	0	0
15669	1303861	ycfV	0.898	1.595	0	0
21198	1304092	ycfV, ycfW	0.984	1.596	0	0
21202	1304182	ycfV, ycfW	1.171	1.603	0	0
11162	1304413	ycfV, ycfW	0.940	1.798	0	1
2005	1304691	ycfW	0.989	1.774	0	1
20716	1304907	ycfW	0.730	1.443	0	0

15066	1305034	ycfW	0.862	1.714	0	0
21434	1305310	ycfW	0.673	1.093	0	0
9540	1305665	ycfW, ycfX	0.775	1.234	0	0
9799	1305823	ycfX	0.984	1.273	0	0
18462	1306149	ycfX	0.767	1.370	0	0
10698	1306351	ycfX, cobB	0.531	1.090	0	0
2218	1306531	ycfX, cobB	0.701	1.561	0	0
4450	1306750	cobB	0.835	1.516	0	0
8256	1306891	cobB	0.792	1.790	0	0
3213	1307251	cobB	0.651	1.543	0	0
4218	1307478	cobB	0.710	1.714	0	0
6933	1307663	potD	0.776	1.758	0	0
10397	1307920	potD	0.692	1.243	0	0
10882	1308200	potD	0.894	1.073	0	0
19985	1308396	potD	0.783	0.878	0	0
630	1308542	potD	0.835	1.022	0	0
21575	1308843	potD, potC	1.009	0.914	0	0
4362	1309113	potD, potC	0.848	0.980	0	0
8595	1309362	potC	1.007	1.045	0	0
22268	1309435	potC	1.015	0.988	0	0
13358	1309516	potC	0.962	0.893	0	0
5024	1309930	potC, sifA	1.138	0.636	0	0
21147	1310115	sifA	2.046	0.698	0	0
10800	1310236	sifA	2.308	0.687	0	0
11793	1310492	sifA	2.245	0.578	0	0
18010	1310761	sifA	2.607	0.550	0	0
16700	1311023	sifA	2.539	0.579	1	0
20130	1311296	potB	2.417	0.681	1	0
6063	1311503	potB	1.371	0.644	0	0
2544	1311655	potB	1.141	0.707	0	0
20015	1312004	potB, potA	0.828	0.857	0	0
21901	1312118	potB, potA	0.856	0.907	0	0
13712	1312295	potB, potA	0.946	0.916	0	0
8101	1312549	potA	0.859	0.764	0	0
2872	1312891	potA	1.076	0.795	0	0
10773	1313052	potA, pepT	1.167	1.016	0	0

2237	1313395	potA, pepT	0.816	0.633	0	0
11552	1313630	pepT	1.223	0.728	0	0
8852	1313769	pepT	0.688	0.488	0	0
7032	1314018	pepT	1.183	0.781	0	0
18000	1314328	pepT, STM1228	1.369	0.742	0	0
11647	1314541	pepT, STM1228	1.116	0.649	0	0
6513	1314759	STM1228	1.541	0.721	0	0
12614	1314835	STM1228	1.797	0.672	0	0
15605	1315131	STM1228	1.749	0.786	0	0
18688	1315352	STM1228	1.631	0.912	0	0
6385	1315592	STM1228	1.114	1.013	0	0
890	1315686	STM1228	1.166	1.028	0	0
6119	1315776	STM1228	1.110	1.176	0	0
5905	1316226	ycfD	0.855	1.340	0	0
17214	1316447	ycfD	0.677	1.047	0	0
5998	1316623	ycfD	0.867	1.331	0	0
19021	1316903	ycfD	0.699	1.818	0	1
11369	1317172	ycfD	0.896	1.737	0	1
2635	1317484	ycfD, phoQ	0.640	1.324	0	0
8085	1317604	phoQ	0.757	1.604	0	0
13550	1317886	phoQ	0.757	1.965	0	0
13547	1318196	phoQ	0.645	1.835	0	0
10864	1318322	phoQ	0.640	1.896	0	1
21104	1318445	phoQ	0.646	2.011	0	1
6561	1318846	phoQ, phoP	0.609	1.888	0	1
19779	1319012	phoQ, phoP	0.466	1.581	0	0
2576	1319361	phoP	0.544	1.874	0	0
626	1319455	phoP	0.864	1.477	0	0
1661	1319696	phoP, purB	0.700	1.295	0	0
4215	1319902	purB	0.754	1.122	0	0
13353	1320076	purB	0.415	0.629	0	0
8325	1320455	purB	0.710	1.072	0	0
20692	1320556	purB	0.739	1.213	0	0
22236	1320727	purB	0.748	1.407	0	0
11592	1320830	purB	0.687	0.938	0	0
920	1321337	ycfC	0.875	1.365	0	0

19743	1321472	ycfC	0.563	1.162	0	0
11349	1321690	ycfC, trmU	0.877	1.750	0	0
8332	1321879	ycfC, trmU	0.598	1.412	0	0
7461	1322195	trmU	0.826	1.789	0	0
1144	1322351	trmU	0.906	1.612	0	0
8941	1322549	trmU	0.636	1.242	0	0
2305	1322663	trmU	0.744	1.691	0	0
12586	1323211	ymfB, STM1236	0.908	0.915	0	0
18165	1323375	ymfB, STM1236	0.803	0.856	0	0
10167	1323513	ymfB, STM1236	0.796	0.931	0	0
10838	1323688	STM1236, ymfC	0.866	1.101	0	0
19926	1324138	ymfC, icdA	1.002	1.120	0	0
10109	1324247	ymfC, icdA	0.812	1.365	0	0
16366	1324561	ymfC, icdA	0.701	1.792	0	1
12291	1324803	icdA	0.689	1.626	0	1
5220	1324937	icdA	0.765	1.706	0	0
11283	1325062	icdA	0.884	1.225	0	0
1819	1325197	icdA	0.892	1.358	0	0
18581	1325579	icdA	1.463	0.779	0	0
9315	1325721	STM1239	2.072	0.693	0	0
17915	1326074	STM1239	2.274	0.536	0	0
14256	1326159	STM1239	2.214	0.605	0	0
18527	1326420	STM1239	1.121	0.374	0	0
19836	1326624	STM1239	2.550	0.534	1	0
21991	1326695	STM1239	2.616	0.531	1	0
17232	1326885	STM1239	2.265	0.475	0	0
11350	1327487		2.121	0.510	0	0
21417	1327558		2.156	0.520	0	0
10042	1327771		2.247	0.436	0	0
19001	1328080		2.040	0.500	0	0
7508	1328415	envF	2.087	0.481	0	0
3104	1328504	envF	2.030	0.483	0	0
4247	1328786	envF	2.160	0.437	0	0
11336	1329010	envF	1.847	0.514	0	0
8052	1329349	envF	1.359	0.638	0	0
15454	1329579	msgA	1.165	0.705	0	0

4955	1329804	msgA	1.082	0.550	0	0
20605	1329940	msgA, envE	1.335	0.611	0	0
13612	1330218	envE	1.744	0.556	0	0
3640	1330297	envE	2.494	0.512	0	0
17600	1330606	envE	2.820	0.586	0	0
10258	1330714	envE	2.022	0.569	0	0
4103	1331029	STM1243	1.617	0.494	0	0
14274	1331371	STM1243, pagD	2.233	0.428	0	0
10414	1331460	STM1243, pagD	2.243	0.424	0	0
7527	1331811	pagD, pagC	2.132	0.509	0	0
16307	1331936	pagC	2.257	0.477	0	0
17462	1332278	pagC	2.413	0.545	0	0
5180	1332473	pagC	1.753	0.430	0	0
2110	1332624	pagC	1.816	0.567	0	0
15724	1332869	STM1247	0.724	0.457	0	0
9750	1332953	STM1247	1.028	0.532	0	0
17329	1333285		1.212	0.754	0	0
802	1333677		1.715	0.831	0	0
19107	1333868		1.326	0.674	0	0
12953	1334048	STM1249	1.238	0.727	0	0
9547	1334175	STM1249	1.428	0.652	0	0
17797	1334346	STM1249	1.535	0.647	0	0
21034	1334769	STM1249, STM1250	1.348	0.528	0	0
13209	1334865	STM1250	1.534	0.657	0	0
9902	1335111	STM1250, agsA	1.208	0.548	0	0
11838	1335450	agsA	0.896	0.762	0	0
21703	1335537	agsA	0.790	0.916	0	0
19433	1335844	agsA, STM1252	0.924	0.761	0	0
13812	1335921	agsA, STM1252	1.032	0.931	0	0
6670	1336055	STM1252	0.925	0.798	0	0
15004	1336286	STM1252	1.122	0.793	0	0
8174	1336788	STM1252	1.393	0.768	0	0
16504	1336878	STM1252	0.662	0.603	0	0
17066	1337296	STM1252	1.444	0.938	0	0
10663	1337454	STM1253	1.447	0.866	0	0
12729	1337580	STM1253	1.450	0.754	0	0

8321	1337959	STM1253	1.368	0.533	0	0
17526	1338150	STM1253, STM1254	1.845	0.677	0	0
14518	1338354	STM1253, STM1254	1.962	0.583	0	0
13265	1338521	STM1254	2.622	0.554	1	0
16244	1338682	STM1254, STM1255	2.674	0.626	1	0
662	1339130	STM1255	2.058	0.874	0	0
20748	1339355	STM1255	1.453	0.714	0	0
3096	1339456	STM1255	1.454	0.788	0	0
6598	1339585	STM1255	0.808	0.591	0	0
17533	1339775	STM1255	1.354	0.963	0	0
9098	1340004	STM1255	1.301	1.010	0	0
19370	1340118	STM1255	1.458	1.108	0	0
11530	1340472	STM1255, STM1256	1.182	0.797	0	0
21925	1340714	STM1255, STM1256	1.153	0.824	0	0
19738	1341149	STM1256	1.539	0.888	0	0
13827	1341266	STM1256, STM1257	1.548	0.873	0	0
12091	1341358	STM1256, STM1257	1.682	0.843	0	0
990	1341635	STM1256, STM1257	1.193	0.698	0	0
9705	1341880	STM1257	1.017	0.674	0	0
11924	1342268	STM1257, STM1258	1.622	1.237	0	0
15234	1342497	STM1257, STM1258	1.418	1.137	0	0
13161	1342742	STM1258	1.061	0.881	0	0
5436	1342889	STM1258, STM1259	1.552	0.944	0	0
8276	1343192	STM1258, STM1259	1.617	0.827	0	0
849	1343268	STM1258, STM1259	1.873	0.805	0	0
20594	1343517	STM1259	1.078	0.971	0	0
7102	1343825	STM1259	1.379	0.892	0	0
2712	1344005	STM1260	1.196	0.721	0	0
15201	1344093	STM1260	1.419	0.777	0	0
15909	1344441	STM1260	1.046	0.856	0	0
2099	1344741	STM1260	1.009	0.887	0	0
13174	1344886	STM1260, STM1261	1.156	1.014	0	0
22328	1345116	STM1260, STM1261	0.949	1.115	0	0
17758	1345383	STM1260, STM1261	1.013	0.805	0	0
9411	1345656	STM1262, STM1263	1.162	0.746	0	0
4863	1345845	STM1263	1.068	0.673	0	0

4395	1346065	STM1263	1.933	0.771	0	0
2636	1346142	STM1263, aadA	2.210	0.712	1	0
10855	1346450	STM1263, aadA	2.250	0.658	1	0
8870	1346649	aadA	1.624	0.711	0	0
10846	1346981	aadA	1.409	0.703	0	0
4599	1347149	aadA, STM1265	1.698	0.768	0	0
3413	1347499	STM1265	2.173	0.535	0	0
9148	1347757	STM1265, STM1266	2.728	0.483	1	0
21562	1347847	STM1265, STM1266	2.916	0.575	1	0
9662	1348142	STM1266	1.996	0.595	0	0
2912	1348347	STM1266	1.677	0.693	0	0
15495	1348428	STM1266	2.042	0.807	0	0
21463	1348798	STM1266	1.599	0.929	0	0
6794	1348888	STM1266	1.477	0.729	0	0
19188	1349296	STM1267	2.136	0.586	0	0
14696	1349421	STM1267, STM1269	2.170	0.587	0	0
9106	1349709	STM1267, STM1268	1.932	0.651	0	0
11021	1349859	STM1267, STM1268	1.693	0.735	0	0
16211	1350008	STM1268, STM1269	1.757	0.828	0	0
13955	1350327	STM1269, yeaS	0.849	0.729	0	0
2394	1350541	yeaS	0.913	1.156	0	0
17207	1350918	yeaS, yeaR	1.292	1.062	0	0
13629	1351058	yeaS, yeaR	0.890	0.820	0	0
16435	1351379	yeaR, yoaG	1.059	0.974	0	0
11532	1351555	yeaR, yoaG	1.105	0.803	0	0
19858	1351628	yeaR, yoaG	1.057	0.754	0	0
14690	1352067	STM1273	0.983	0.519	0	0
22107	1352162	STM1273	0.924	0.588	0	0
20374	1352455	STM1273	0.958	0.707	0	0
18250	1352684	STM1273, yeaQ	0.834	0.624	0	0
5351	1352954	yeaQ	0.892	0.753	0	0
14277	1353097	yeaQ	0.926	0.653	0	0
3335	1353258	yeaQ	0.764	0.620	0	0
7077	1353579	yaoF	1.007	0.969	0	0
14744	1353782	yaoF, STM1276	1.121	1.049	0	0
14894	1354014	yaoF, STM1276	0.769	1.151	0	0

14685	1354244	STM1276	0.980	1.930	0	0
19341	1354597	yeaO	0.762	1.738	0	0
9489	1354770	yeaO	0.681	1.140	0	0
4866	1354878	yeaO, yeaN	0.831	1.173	0	0
1547	1355226	yeaN	1.184	0.914	0	0
19025	1355389	yeaN	1.383	0.760	0	0
12908	1355679	yeaN	1.111	0.661	0	0
3176	1355753	yeaN, yeaM	0.902	0.603	0	0
16808	1356091	yeaN, yeaM	1.054	0.828	0	0
21461	1356163	yeaN, yeaM	1.402	1.385	0	0
20422	1356265	yeaN, yeaM	1.167	1.282	0	0
21897	1356625	yeaM	1.580	1.234	0	0
5365	1356863	yeaM	1.303	1.126	0	0
2829	1357260	yeaL, STM1281	1.560	1.210	0	0
9512	1357389	yeaL, STM1281	1.203	0.949	0	0
18791	1357579	yeaL, STM1281	1.212	0.782	0	0
8168	1357652	yeaL, STM1281	0.609	0.559	0	0
18291	1358217	yeaK	1.900	0.713	0	0
20243	1358331	yeaK, yeaJ	2.221	0.679	0	0
8594	1358592	yeaK, yeaJ	3.024	0.648	1	0
5159	1358741	yeaJ	3.601	0.724	1	0
11048	1358954	yeaJ	3.436	0.602	1	0
6300	1359196	yeaJ	2.638	0.595	1	0
19705	1359597	yeaJ	2.496	0.702	1	0
7619	1359776	yeaJ	2.515	0.599	1	0
1637	1359928	yeaJ	3.000	0.586	1	0
12427	1360155	yeaJ	2.852	0.632	1	0
15553	1360393	yeaH	1.750	0.659	0	0
973	1360682	yeaH	1.676	0.943	0	0
9628	1360862	yeaH	1.113	0.867	0	0
22301	1360991	yeaH	1.219	1.019	0	0
9933	1361311	yeaH	1.177	0.965	0	0
10531	1361507	yeaH	1.146	1.166	0	0
17543	1361587	yeaH	1.172	1.114	0	0
11365	1361866	yeaH, yeaG	0.968	0.905	0	0
18093	1362207	yeaG	0.930	0.691	0	0

7923	1362312	yeaG	0.933	0.735	0	0
8118	1362727	yeaG	0.939	0.657	0	0
8645	1362798	yeaG	1.112	0.812	0	0
9657	1362942	yeaG	1.016	0.820	0	0
13081	1363442	yeaG	0.970	1.189	0	0
19419	1363677	yeaG	0.800	0.943	0	0
3659	1363902	yeaG, mipA	0.805	0.948	0	0
19668	1363986	yeaG, mipA	0.858	0.876	0	0
19734	1364177	yeaG, mipA	0.765	1.069	0	0
7065	1364278	mipA	0.627	0.919	0	0
1912	1364663	mipA	0.764	1.375	0	0
4160	1364750	mipA, STM1287	0.669	1.175	0	0
5115	1365180	STM1287	1.057	0.959	0	0
12307	1365251	STM1287	0.714	0.722	0	0
4891	1365479	STM1287	1.012	0.668	0	0
12105	1365900	STM1287	1.115	0.759	0	0
22140	1366081	STM1287, STM1288	0.996	0.740	0	0
14867	1366281	STM1287, STM1288	1.057	0.692	0	0
6845	1366452	STM1288	1.158	0.828	0	0
18398	1366799	STM1288	1.000	0.799	0	0
4801	1367041	STM1288	0.944	0.936	0	0
20498	1367112	STM1288	0.909	1.010	0	0
13688	1367428		0.872	1.207	0	0
20265	1367745	yeaD	0.997	1.125	0	0
5116	1367983	yeaD	0.796	0.939	0	0
8421	1368163	yeaD	0.816	0.898	0	0
6152	1368306	yeaD	0.842	1.202	0	0
18500	1368661	yeaD	0.690	1.508	0	0
4706	1368800	gapA	0.647	1.857	0	0
16398	1368916	gapA	0.560	1.768	0	1
21527	1369153	gapA	0.565	2.260	0	1
21808	1369536	gapA, yeaA	0.490	2.269	0	1
16093	1369643	gapA, yeaA	0.486	2.313	0	1
1565	1369922	gapA, yeaA	0.466	1.672	0	0
16653	1370037	gapA, yeaA	0.502	1.942	0	0
2742	1370453	yeaC	0.652	1.571	0	0

800	1370640	yeaC	0.723	2.364	0	1
418	1370891		0.642	2.259	0	1
7558	1371052		0.724	1.852	0	0
16456	1371361	pncA	0.846	1.184	0	0
13316	1371432	pncA	0.830	1.060	0	0
8057	1371729	pncA	0.934	1.004	0	0
12056	1371860	pncA, ansA	0.509	0.673	0	0
5186	1372081	pncA, ansA	1.371	0.988	0	0
816	1372337	ansA	1.288	0.900	0	0
12045	1372549	ansA	1.053	1.121	0	0
5845	1372779	ansA	0.824	0.860	0	0
19277	1373072	ansA, sppA	1.034	0.953	0	0
20911	1373293	sppA	1.097	1.360	0	0
21743	1373416	sppA	1.134	1.297	0	0
5085	1373815	sppA	1.160	0.964	0	0
8931	1374133	sppA	1.034	0.904	0	0
289	1374258	sppA	0.713	0.703	0	0
13355	1374427	sppA	1.433	1.158	0	0
21253	1374758	sppA, ydjA	1.112	1.051	0	0
13123	1374915	sppA, ydjA	1.094	1.062	0	0
21758	1375036	sppA, ydjA	0.893	0.928	0	0
18740	1375211	ydjA, selD	0.535	0.621	0	0
7835	1375583	selD	0.938	1.407	0	0
20454	1375793	selD	0.451	0.725	0	0
5300	1376123	selD	0.745	0.991	0	0
5720	1376252	selD, topB	1.015	1.777	0	0
8534	1376351	selD, topB	0.839	1.241	0	0
14538	1376734	topB	0.657	1.105	0	0
20228	1376878	topB	0.873	1.428	0	0
14248	1377284	topB	1.238	1.762	0	0
1700	1377442	topB	0.884	1.335	0	0
11130	1377584	topB	0.997	1.283	0	0
21712	1377821	topB	1.189	1.484	0	0
21048	1377907	topB	1.055	1.335	0	0
5484	1378254	topB	1.104	1.576	0	0
4276	1378372	topB	1.065	1.409	0	0

310	1378880	gdhA	0.930	1.628	0	0
1102	1379027	gdhA	0.555	0.812	0	0
4836	1379278	gdhA	1.076	1.009	0	0
8622	1379397	gdhA	1.056	1.034	0	0
14788	1379632	gdhA	1.389	1.109	0	0
13371	1379929	gdhA, STM1300	1.342	1.080	0	0
14540	1380037	gdhA, STM1300	1.398	1.178	0	0
18292	1380182	gdhA, STM1300	1.212	1.549	0	0
21248	1380355	gdhA, STM1300	1.093	2.032	0	0
3755	1380844	STM1301	0.702	1.388	0	0
5494	1381079	STM1301, xthA	0.896	1.047	0	0
10747	1381311	xthA	1.152	1.071	0	0
3727	1381559	xthA	0.988	1.030	0	0
14271	1381732	xthA, astC	1.032	0.877	0	0
9766	1382042	xthA, astC	0.991	0.704	0	0
7488	1382191	astC	0.889	0.691	0	0
4423	1382376	astC	1.254	0.709	0	0
9295	1382479	astC	1.575	0.858	0	0
17977	1382683	astC	1.244	0.894	0	0
17338	1383125	astC, astA	1.256	0.953	0	0
14909	1383201	astC, astA	0.504	0.417	0	0
14809	1383663	astA	1.575	0.912	0	0
16362	1383735	astA	1.299	0.840	0	0
6233	1384011	astA, astD	1.522	0.807	0	0
16026	1384329	astA, astD	1.232	0.747	0	0
5275	1384400	astA, astD	1.178	0.597	0	0
4942	1384743	astD	1.227	0.815	0	0
5507	1384927	astD	1.838	0.850	0	0
10211	1385176	astD	1.153	0.576	0	0
6662	1385420	astD, astB	0.963	0.508	0	0
13599	1385595	astD, astB	1.783	1.073	0	0
857	1385680	astD, astB	1.877	0.798	0	0
19643	1386009	astB	0.986	0.684	0	0
9857	1386279	astB	1.716	0.904	0	0
8781	1386609	astB	1.459	0.785	0	0
7616	1386795	astB, astE	0.935	0.547	0	0

4261	1387021	astB, astE	1.358	1.107	0	0
4659	1387208	astB, astE	1.107	0.664	0	0
18107	1387447	astE	0.863	0.650	0	0
19879	1387674	astE	0.735	0.636	0	0
13153	1387831	astE	2.009	0.782	0	0
18382	1388194	spy	3.637	0.661	1	0
4107	1388265	spy	3.716	0.658	1	0
15264	1388536	spy	4.332	0.818	1	0
5674	1388694	spy	3.180	0.731	1	0
19624	1389127	STM1309	1.854	0.573	0	0
18034	1389265	STM1309	1.225	0.867	0	0
3383	1389479	STM1309	0.708	0.444	0	0
19126	1389605	STM1309	1.387	0.777	0	0
10930	1389912	STM1309	1.211	0.776	0	0
17903	1390013	STM1309	0.837	0.713	0	0
9685	1390097	STM1309, nadE	1.089	0.617	0	0
21200	1390193	STM1309, nadE	1.078	0.852	0	0
12572	1390915	nadE, osmE	1.427	1.087	0	0
15597	1391123	nadE, osmE	1.116	0.874	0	0
3590	1391238	nadE, osmE	1.328	0.949	0	0
17366	1391556	celA	1.162	0.739	0	0
16691	1391765	celA, celB	2.238	0.874	1	0
4270	1392006	celA, celB	3.025	0.788	1	0
1284	1392241	celB	2.030	0.521	0	0
10547	1392391	celB	3.818	0.678	0	0
15443	1392615	celB	1.241	0.529	0	0
7198	1392904	celB	1.774	0.580	0	0
13698	1393213	celB, celC	1.480	0.671	0	0
18754	1393318	celB, celC	1.684	0.783	0	0
12745	1393618	celC, celD	1.414	0.719	0	0
12314	1393854	celC, celD	0.511	0.379	0	0
8562	1394029	celD	1.992	0.627	0	0
13191	1394326	celD	2.701	0.527	1	0
16553	1394446	celD, celF	3.186	0.617	1	0
9977	1394661	celD, celF	3.395	0.599	1	0
8742	1394801	celF	2.840	0.521	1	0

21169	1395013	celF	3.287	0.555	1	0
7936	1395316	celF	3.186	0.663	1	0
2223	1395647	celF	3.183	0.577	1	0
21054	1395800	celF	3.139	0.637	1	0
2163	1395933	celF, celG	2.341	0.606	0	0
1923	1396310	celG	1.443	0.545	0	0
5200	1396470	celG	1.065	0.595	0	0
2094	1396814	celG	1.476	0.741	0	0
15578	1397022	celG	1.290	0.854	0	0
3716	1397206	katE	1.041	0.701	0	0
4881	1397424	katE	1.038	0.685	0	0
13427	1397619	katE	0.998	0.668	0	0
21497	1397967	katE	0.939	0.737	0	0
17564	1398208	katE	1.418	0.777	0	0
382	1398312	katE	1.454	0.911	0	0
4600	1398497	katE	1.382	0.713	0	0
14942	1398768	katE	1.491	0.789	0	0
7779	1398864	katE	1.427	0.732	0	0
16189	1399368	katE, cedA	0.844	0.852	0	0
15849	1399512	katE, cedA	1.141	1.043	0	0
16020	1399703	katE, cedA	1.480	1.166	0	0
3506	1399835	cedA	1.183	0.977	0	0
21241	1400018		0.752	0.864	0	0
11051	1400096	ydjN	0.936	0.945	0	0
979	1400652	ydjN	1.149	0.922	0	0
13396	1400902	ydjN	1.005	0.736	0	0
13013	1401113	ydjN	1.090	0.719	0	0
16236	1401260	ydjN	1.190	0.627	0	0
10763	1401512	ydjN	1.254	0.681	0	0
4964	1401611	ydjN, ydjM	1.569	0.714	0	0
4386	1401995	ydjM	0.880	0.980	0	0
9092	1402224	ydjM	0.649	0.803	0	0
20867	1402295	ydjM	0.639	0.647	0	0
5150	1402537	ydjM, yniC	0.891	0.804	0	0
8312	1402953	yniC, yniB	0.877	0.786	0	0
12182	1403176	yniC, yniB	0.991	0.898	0	0

1791	1403407	yniC, yniB	0.648	0.671	0	0
11144	1403576	yniB	0.932	1.172	0	0
4668	1403648	yniB	1.138	1.272	0	0
564	1404074	STM1324	1.073	1.255	0	0
11925	1404315	STM1324	0.646	0.720	0	0
12989	1404577	STM1324	0.905	1.003	0	0
6047	1404820	STM1324, ydiZ	0.952	0.797	0	0
16382	1404900	STM1324, ydiZ	1.288	0.908	0	0
14230	1405098	ydiZ, pfkB	0.319	0.259	0	0
9002	1405266	ydiZ, pfkB	0.814	0.739	0	0
17032	1405614	pfkB	1.652	0.979	0	0
891	1405764	pfkB	1.347	0.748	0	0
9021	1406024	pfkB, ydiY	2.322	0.861	0	0
16440	1406177	pfkB, ydiY	1.908	0.959	0	0
13842	1406542	ydiY	1.529	0.884	0	0
4911	1406614	ydiY	1.442	0.846	0	0
20820	1406944	ydiY	1.875	0.866	0	0
18854	1407115	STM1328	2.191	0.766	0	0
17687	1407272	STM1328	2.216	0.800	0	0
5658	1407695	STM1328	2.459	0.579	1	0
11155	1407950	STM1328, STM1329	2.802	0.540	1	0
3852	1408189	STM1328, STM1329	2.349	0.448	0	0
12141	1408349	STM1328, STM1329	3.219	0.430	0	0
2378	1408565	STM1330	1.719	0.497	0	0
21079	1408678	STM1330	2.086	0.498	0	0
2632	1408949	STM1330	1.600	0.678	0	0
11158	1409175	STM1330, STM1331	1.337	0.734	0	0
16457	1409399	STM1331	1.648	0.670	0	0
11642	1409624	STM1331	2.000	0.683	0	0
8138	1409815		2.410	0.592	0	0
2047	1410006		2.318	0.504	0	0
11865	1410320	rfc	2.468	0.551	0	0
16802	1410526	rfc	2.280	0.493	0	0
6459	1410601	rfc	2.058	0.424	0	0
12883	1411137	rfc	2.264	0.563	0	0
20846	1411350	rfc	1.698	0.519	0	0

10022	1411594	rfc	1.366	0.620	0	0
11919	1411778	thrS	0.858	0.668	0	0
14083	1411944	thrS	0.558	0.548	0	0
20989	1412167	thrS	0.810	0.911	0	0
1614	1412495	thrS	0.747	1.171	0	0
1269	1412694	thrS	0.795	0.952	0	0
21582	1412822	thrS	0.750	0.944	0	0
16765	1413050	thrS	0.850	1.098	0	0
21235	1413391	thrS	0.779	1.589	0	0
902	1413552	thrS	0.719	1.845	0	1
6453	1413821	thrS, infC	1.288	2.070	0	1
2554	1413897	thrS, infC	0.602	2.586	0	1
8613	1414232	infC	0.527	2.447	0	1
4245	1414408	infC, rpmI	0.494	1.928	0	0
6570	1414633	infC, rpmI	0.362	2.147	0	0
486	1414833	rpmI, rplT	0.349	2.149	0	0
12588	1415226	rplT, pheS	0.414	2.286	0	1
17097	1415445	pheS	0.459	2.043	0	1
11976	1415516	pheS	0.487	1.999	0	0
3455	1415592	pheS	2.217	1.525	0	0
7626	1415745	pheS	0.677	1.648	0	0
3433	1416282	pheS, pheT	0.987	1.137	0	0
7191	1416372	pheS, pheT	0.857	1.127	0	0
20719	1416605	pheS, pheT	1.081	1.152	0	0
3607	1416676	pheT	1.048	1.045	0	0
11738	1417102	pheT	1.067	1.259	0	0
5154	1417432	pheT	0.956	1.224	0	0
8246	1417646	pheT	0.715	0.932	0	0
21189	1417905	pheT	1.050	1.404	0	0
10551	1418196	pheT	0.859	1.149	0	0
4952	1418375	pheT	0.863	1.163	0	0
14670	1418494	pheT	0.840	1.359	0	0
2312	1418649	pheT, himA	0.730	1.208	0	0
21038	1418850	pheT, himA	0.540	1.350	0	0
16980	1419191	himA, btuC	0.755	2.095	0	0
14939	1419515	btuC	0.679	1.353	0	0

13820	1419624	btuC	0.346	0.737	0	0
15638	1419797	btuC	0.969	1.596	0	0
20057	1420055	btuC	1.280	1.554	0	0
17661	1420325	btuC, btuE	0.969	1.181	0	0
19150	1420639	btuE	1.311	1.080	0	0
13620	1420765	btuE, btuD	0.501	0.586	0	0
16079	1421061	btuE, btuD	0.567	0.757	0	0
2075	1421173	btuD	1.325	1.254	0	0
1099	1421609	btuD, nlpC	0.963	1.079	0	0
2814	1421695	btuD, nlpC	0.982	1.007	0	0
21668	1421995	nlpC	1.474	1.092	0	0
2500	1422249	nlpC	1.963	0.888	0	0
16817	1422347	nlpC, ydiV	2.265	0.932	0	0
6138	1422706	ydiV	1.882	0.671	0	0
20713	1422803	ydiV	2.646	0.663	1	0
6982	1422958	ydiV	2.659	0.665	1	0
21914	1423163	ydiV, ydiU	2.617	0.670	1	0
4138	1423237	ydiV, ydiU	2.970	0.710	1	0
21381	1423311	ydiV, ydiU	2.314	0.777	1	0
11481	1424095	ydiU	1.000	1.177	0	0
2303	1424302	ydiU	0.797	0.990	0	0
22158	1424579	ydiU	1.077	1.310	0	0
491	1424650	ydiU	0.874	1.079	0	0
11606	1424900	ydiU	0.850	1.479	0	0
19322	1425214	ydiE	0.992	1.075	0	0
7634	1425434	ydiE, aroH	1.151	0.973	0	0
21819	1425506	ydiE, aroH	1.058	1.102	0	0
457	1425773	aroH	1.134	1.299	0	0
20597	1426070	aroH	1.022	1.070	0	0
20933	1426262	aroH	0.800	0.959	0	0
1921	1426552	aroH	0.824	0.881	0	0
22286	1426623	aroH, ydiA	1.026	0.990	0	0
10487	1426817	aroH, ydiA	0.999	0.900	0	0
20369	1427171	ydiA	1.443	0.962	0	0
20739	1427412	ydiA, pps	1.986	0.953	0	0
18750	1427728	ydiA, pps	1.854	1.194	0	0

13616	1427896	pps	1.401	1.423	0	0
13799	1428164	pps	1.005	1.394	0	0
2516	1428374	pps	0.660	1.188	0	0
13382	1428672	pps	0.749	1.743	0	0
3512	1428815	pps	0.805	1.849	0	0
15321	1429132	pps	0.840	1.936	0	0
12947	1429353	pps	0.707	1.519	0	0
18562	1429471	pps	0.735	1.403	0	0
18150	1429827	pps	0.770	1.857	0	1
11878	1429902	pps	0.861	1.768	0	1
17431	1430095	pps	0.531	0.809	0	0
4758	1430274	ydiD	1.088	2.003	0	0
4543	1430475	ydiD	0.844	1.145	0	0
13811	1430771	ydiD	1.349	0.841	0	0
16350	1430901	ydiD	0.935	0.514	0	0
7175	1430983	ydiD	1.045	0.599	0	0
8322	1431420	ydiD	1.053	0.623	0	0
3472	1431806	ydiD	2.119	0.878	0	0
1462	1431957	ydiD, ydiT	2.377	0.781	1	0
13264	1432221	ydiD, ydiT	2.461	0.687	1	0
12886	1432359	ydiT, ydiS	2.045	0.662	0	0
18534	1432649	ydiT, ydiS	1.145	0.613	0	0
18698	1432904	ydiS	1.424	0.698	0	0
6824	1433152	ydiS	1.201	0.629	0	0
2434	1433223	ydiS	2.256	0.789	0	0
14243	1433537	ydiS, ydiR	1.746	0.837	0	0
22261	1433689	ydiS, ydiR	1.807	0.889	0	0
18621	1433886	ydiS, ydiR	0.997	0.664	0	0
21523	1434351	ydiR	0.768	1.097	0	0
15650	1434429	ydiR	0.777	0.968	0	0
12384	1434764	ydiR, ydiQ	1.248	1.059	0	0
4175	1434902	ydiR, ydiQ	1.296	0.799	0	0
5895	1435216	ydiQ	2.541	0.494	1	0
8719	1435374	ydiQ, ydiP	2.971	0.499	1	0
14332	1435626	ydiQ, ydiP	2.814	0.436	1	0
5285	1435768	ydiP	1.829	0.465	0	0

11432	1436020	ydiP	2.545	0.504	1	0
15508	1436160	ydiP	2.732	0.514	1	0
1853	1436396	ydiP	3.202	0.526	1	0
10064	1436668		2.166	0.625	0	0
11282	1436818	ydiO	0.949	0.568	0	0
10911	1437122	ydiO	1.539	0.693	0	0
9940	1437215	ydiO	1.624	0.662	0	0
17540	1437349	ydiO	1.908	0.576	0	0
18955	1437939	ydiO, ydiF	3.930	0.592	1	0
20921	1438128	ydiO, ydiF	3.426	0.599	1	0
16077	1438266	ydiO, ydiF	3.831	0.666	1	0
17021	1438612	ydiF	2.196	0.630	1	0
1321	1438764	ydiF	1.881	0.659	0	0
6330	1438960	ydiF	2.661	0.610	1	0
899	1439367	ydiF	4.448	0.606	1	0
20990	1439590	ydiF, aroD	4.345	0.536	1	0
6350	1439733	ydiF, aroD	3.635	0.552	1	0
8868	1439934	aroD	1.477	0.546	0	0
6440	1440115	aroD	1.870	0.708	0	0
2553	1440341	aroD	1.174	0.717	0	0
1898	1440557	aroD, ydiB	1.321	0.641	0	0
6797	1440800	aroD, ydiB	1.567	0.567	0	0
8599	1441139	ydiB	2.583	2.218	1	0
15970	1441331	ydiB, ydiN	3.237	0.559	1	0
3890	1441483	ydiB, ydiN	3.259	0.521	1	0
3739	1441580	ydiB, ydiN	3.667	0.524	1	0
1582	1441965	ydiN	2.318	0.555	0	0
14132	1442122	ydiN	2.498	0.544	1	0
5018	1442446	ydiN	2.782	0.485	1	0
2485	1442595	ydiN	2.839	0.431	1	0
5149	1442882	ydiN	2.981	0.424	1	0
4906	1443061	ydiM	3.276	0.645	1	0
15464	1443222	ydiM	2.409	0.509	1	0
9963	1443377	ydiM	2.556	0.536	1	0
4014	1443500	ydiM	2.377	0.586	1	0
7101	1444063	ydiM	1.926	0.538	0	0

6956	1444187	ydiM	1.926	0.427	0	0
13753	1444580	ydiM, ydiL	2.343	0.597	0	0
6497	1444706	ydiL	1.745	0.620	0	0
10376	1444883	ydiL, rprA	1.743	0.712	0	0
5125	1445036	ydiL, rprA	1.374	0.767	0	0
13482	1445226	rprA, ydiK	1.177	0.897	0	0
20591	1445468	ydiK	1.030	0.938	0	0
2778	1445868	ydiK	1.406	0.996	0	0
10072	1446100	ydiK, ydiJ	1.272	0.974	0	0
22214	1446365	ydiK, ydiJ	1.450	1.316	0	0
12960	1446471	ydiK, ydiJ	1.515	1.513	0	0
12043	1446794	ydiJ	1.952	1.270	0	0
13222	1446872	ydiJ	1.442	1.213	0	0
9131	1447177	ydiJ	1.603	1.081	0	0
20455	1447332	ydiJ	1.437	1.123	0	0
11887	1447662	ydiJ	1.750	1.078	0	0
20091	1447901	ydiJ	1.316	1.061	0	0
1727	1448066	ydiJ	1.424	1.181	0	0
19512	1448447	ydiJ	1.473	1.104	0	0
3997	1448559	ydiJ	1.158	0.997	0	0
9736	1448729	ydiJ	1.106	0.922	0	0
1238	1449003	ydiJ	1.569	0.956	0	0
3712	1449365	ydiJ, STM1366	1.143	0.995	0	0
20979	1449537	STM1366, ydiH	1.023	1.134	0	0
7836	1449741	STM1366, ydiH	0.622	0.759	0	0
7382	1449893	STM1366, ydiH	0.787	0.868	0	0
19945	1450031	ydiH	1.007	0.977	0	0
21726	1450170	ydiH	0.927	0.990	0	0
8235	1450305		1.013	0.879	0	0
15491	1450409		1.282	0.808	0	0
14091	1451064	STM1368	1.714	0.632	0	0
754	1451376	STM1368	1.616	0.611	0	0
19548	1451568	STM1368	1.948	0.619	0	0
20022	1451793	STM1368	2.101	0.559	0	0
2469	1451902	STM1368, sufA	1.770	0.582	0	0
6889	1452283	STM1368, sufA	1.596	0.575	0	0

3111	1452468	sufA, sufB	1.162	0.568	0	0
5209	1452744	sufB	1.088	0.643	0	0
5420	1452909	sufB	1.144	0.634	0	0
8286	1453179	sufB	0.844	0.643	0	0
17549	1453351	sufB	1.409	0.887	0	0
20523	1453500	sufB	1.344	0.908	0	0
10618	1453762	sufB, sufC	1.215	0.862	0	0
17836	1454053	sufB, sufC	1.413	0.814	0	0
17738	1454191	sufC	1.126	0.618	0	0
17833	1454535	sufC, sufD	1.637	0.751	0	0
3283	1454783	sufC, sufD	0.978	0.618	0	0
20794	1454937	sufD	1.322	0.693	0	0
12816	1455007	sufD	1.210	0.702	0	0
3963	1455176	sufD	1.032	0.765	0	0
2993	1455558	sufD	1.238	0.869	0	0
21764	1455746	sufD, sufS	1.222	0.867	0	0
18286	1455909	sufD, sufS	1.208	0.726	0	0
17165	1456281	sufS	1.193	0.930	0	0
18282	1456475	sufS	1.673	0.984	0	0
6786	1456651	sufS	0.795	0.643	0	0
9872	1457053	sufS, ynhA	0.989	0.742	0	0
14934	1457230	sufS, ynhA	1.311	0.717	0	0
1390	1457451	ynhA	1.804	0.953	0	0
5025	1457657	ynhA, ynhG	1.592	0.888	0	0
5466	1457819	ynhG	1.194	0.828	0	0
1801	1458085	ynhG	1.283	0.771	0	0
5679	1458356	ynhG	1.390	1.027	0	0
1356	1458645	ynhG	0.877	0.927	0	0
3407	1458722	ynhG	1.087	1.508	0	0
4789	1459042	lppB	0.682	1.553	0	0
5493	1459186	lppB	0.545	1.621	0	0
874	1459486	lppB, lpp	0.754	2.077	0	0
4012	1459722	lppB, lpp	0.853	2.368	0	0
4447	1459897	lpp, pykF	0.676	0.898	0	0
5327	1460020	lpp, pykF	0.658	1.053	0	0
13970	1460097	pykF	0.559	0.838	0	0

1773	1460627	pykF	0.857	1.245	0	0
10898	1460709	pykF	0.975	1.366	0	0
9663	1461102	pykF	0.782	1.181	0	0
19663	1461326	pykF	0.766	1.130	0	0
21878	1461609	pykF	0.978	1.360	0	0
14921	1461831	orf48	1.101	0.972	0	0
14974	1461942	orf48	0.496	0.635	0	0
16245	1462078	orf48	0.703	0.687	0	0
8252	1462519	orf48	1.427	0.988	0	0
1909	1462631	orf48	1.308	0.850	0	0
2230	1462776	orf48	1.692	0.990	0	0
2690	1463011	orf48	0.614	0.576	0	0
12647	1463401	orf48, orf32	1.669	0.793	0	0
2156	1463666	orf32	2.738	0.692	1	0
6777	1463908	orf32	3.746	0.571	1	0
20952	1464139	orf32, orf245	3.784	0.533	1	0
469	1464279	orf32, orf245	3.166	0.612	1	0
9767	1464536	orf245	3.210	0.521	1	0
6838	1464715	orf245	2.905	0.384	1	0
20920	1464811	orf245, orf408	3.103	0.344	1	0
22054	1465129	orf245, orf408	3.308	0.439	1	0
8968	1465286	orf408	3.767	0.485	1	0
4429	1465507	orf408	2.936	0.540	1	0
15687	1465881	orf408	4.069	0.470	1	0
15795	1466039	orf408	3.805	0.383	1	0
5621	1466217	orf408	3.668	0.528	1	0
17614	1466288	orf408	3.935	0.630	1	0
3626	1466752	ttrA	1.961	0.632	0	0
15141	1466906	ttrA	1.835	0.685	0	0
9534	1467134	ttrA	1.149	0.483	0	0
19715	1467516	ttrA	2.509	0.619	0	0
22221	1467668	ttrA	1.930	0.841	0	0
14261	1467831	ttrA	1.495	0.803	0	0
19000	1468120	ttrA	1.392	0.847	0	0
1623	1468311	ttrA	1.691	0.819	0	0
15780	1468560	ttrA	1.260	0.663	0	0

11143	1468748	ttrA	1.192	0.637	0	0
4984	1469054	ttrA	1.108	0.602	0	0
21864	1469162	ttrA	1.059	0.633	0	0
1048	1469410	ttrA, ttrC	1.449	0.675	0	0
8864	1469530	ttrA, ttrC	1.569	0.643	0	0
19315	1469670	ttrA, ttrC	0.705	0.554	0	0
15423	1470045	ttrC	1.815	0.661	0	0
6674	1470379	ttrC	1.054	0.524	0	0
13739	1470682	ttrC, ttrB	1.528	0.663	0	0
19629	1470880	ttrB, ttrS	0.861	0.766	0	0
9764	1471143	ttrB, ttrS	1.139	1.194	0	0
16313	1471311	ttrB, ttrS	0.765	0.747	0	0
9447	1471480	ttrB, ttrS	0.808	1.149	0	0
1403	1471839	ttrS	1.440	1.719	0	0
19365	1471953	ttrS	1.464	1.683	0	0
17436	1472276	ttrS	0.882	1.050	0	0
13621	1472408	ttrS	0.990	1.090	0	0
9996	1472675	ttrS, ttrR	1.186	1.184	0	0
15261	1472835	ttrS, ttrR	1.262	1.247	0	0
17716	1473064	ttrS, ttrR	0.553	0.804	0	0
15482	1473155	ttrR	0.938	1.039	0	0
10672	1473667	ttrR, orf70	0.905	2.243	0	1
14824	1473745	orf70	0.807	2.020	0	1
12330	1474124	orf319	0.716	2.012	0	1
9362	1474323	orf319	0.614	1.389	0	0
10196	1474394	orf319	0.845	1.874	0	1
7901	1474570	orf319	0.897	1.868	0	1
10775	1474796	orf319	1.194	1.807	0	1
11767	1475050	orf319	1.300	1.114	0	0
15221	1475435	orf319, orf242	1.131	0.847	0	0
14200	1475513	orf319, orf242	1.343	0.873	0	0
18620	1475765	orf242	1.341	0.810	0	0
1736	1476098	orf242	1.560	0.694	0	0
19436	1476206	orf242, ssrB	1.437	0.541	0	0
16759	1476382	orf242, ssrB	2.474	0.498	1	0
11233	1476650	ssrB	2.558	0.409	1	0

6416	1476967	ssrB, ssrA	2.352	0.483	0	0
17420	1477183	ssrB, ssrA	2.701	0.498	0	0
12764	1477275	ssrA	2.064	0.390	0	0
17571	1477574	ssrA	2.655	0.512	0	0
20445	1477935	ssrA	2.155	0.593	0	0
14738	1478170	ssrA	2.146	0.442	0	0
976	1478329	ssrA	1.886	0.448	0	0
18108	1478476	ssrA	1.872	0.473	0	0
17798	1478859	ssrA	1.872	0.586	0	0
9349	1479079	ssrA	1.859	0.527	0	0
13587	1479206	ssrA	1.979	0.558	0	0
8944	1479576	ssrA, ssaB	2.134	0.415	0	0
3196	1479802	ssrA, ssaB	1.796	0.457	0	0
9555	1479912	ssrA, ssaB	2.130	0.447	0	0
16524	1480149	ssaB, ssaC	2.369	0.597	0	0
16240	1480249	ssaB, ssaC	2.239	0.598	0	0
9622	1480466	ssaC	2.229	0.404	0	0
3029	1480869	ssaC	2.097	0.622	0	0
6677	1481149	ssaC	1.867	0.506	0	0
10415	1481333	ssaC	2.095	0.502	0	0
21821	1481492	ssaC, ssaD	2.465	0.555	1	0
18956	1481707	ssaC, ssaD	2.658	0.511	1	0
16188	1481947	ssaD	2.180	0.582	0	0
5422	1482156	ssaD	2.577	0.476	0	0
5713	1482251	ssaD	2.057	0.471	0	0
17422	1482452	ssaD	1.784	0.551	0	0
15497	1482768	ssaD, ssaE	2.104	0.503	0	0
18972	1483078	ssaE	2.307	0.561	0	0
241	1483180	ssaE	1.955	0.581	0	0
9090	1483658		2.063	0.538	0	0
1492	1483903		2.288	0.546	0	0
1972	1484134	sscA	2.077	0.652	0	0
17821	1484360	sscA	1.875	0.683	0	0
1066	1484488	sscA	1.844	0.799	0	0
10490	1484693	sscA, sseC	1.167	0.634	0	0
1530	1484926	sscA, sseC	1.243	0.697	0	0

14591	1485032	sseC	0.985	0.582	0	0
5132	1485371	sseC	1.579	0.627	0	0
6257	1485629	sseC	1.206	0.434	0	0
363	1485790	sseC	1.183	0.786	0	0
867	1486054	sseC, sseD	1.940	0.618	0	0
20209	1486307	sseC, sseD	1.516	0.458	0	0
885	1486562	sseD, sseE	2.535	0.727	0	0
6951	1486669	sseD, sseE	1.895	0.594	0	0
5836	1487024	sseD, sseE	2.370	0.585	0	0
12088	1487286	sseE, sscB	2.072	0.621	0	0
17085	1487443	sseE, sscB	2.306	0.606	1	0
11873	1487730	sscB, sseF	2.345	0.685	1	0
18082	1487897	sscB, sseF	2.213	0.688	0	0
2831	1488007	sseF	2.147	0.700	0	0
12979	1488270	sseF, sseG	1.876	0.530	0	0
6029	1488446	sseF, sseG	2.223	0.631	0	0
8410	1488705	sseF, sseG	1.493	0.520	0	0
464	1488833	sseG	1.513	0.638	0	0
12068	1489310	sseG, ssaG	1.728	0.493	0	0
9597	1489593	ssaG, ssaH	2.523	0.477	1	0
14808	1489724	ssaH, ssaI	2.515	0.506	1	0
10334	1489823	ssaH, ssaI	3.241	0.410	1	0
14866	1490104	ssaI, ssaJ	2.486	0.478	1	0
17706	1490259	ssaJ	2.376	0.428	1	0
1795	1490579	ssaJ, STM1410	2.801	0.552	1	0
3839	1490917	ssaJ, STM1410	2.160	0.511	0	0
11421	1491049	STM1410	2.219	0.553	0	0
17662	1491277	STM1410, ssaK	1.968	0.542	0	0
16954	1491355	STM1410, ssaK	2.405	0.549	0	0
4278	1491704	ssaK	2.140	0.441	0	0
19820	1492050	ssaK, ssaL	2.439	0.572	1	0
17622	1492194	ssaK, ssaL	2.547	0.554	1	0
3071	1492307	ssaL	1.553	0.429	0	0
9053	1492532	ssaL	2.380	0.577	0	0
3046	1492837	ssaL, ssaM	1.827	0.614	0	0
21436	1493070	ssaL, ssaM	2.702	0.572	0	0

18302	1493418	ssaM, ssaV	2.100	0.450	0	0
21242	1493624	ssaM, ssaV	1.639	0.586	0	0
9230	1493809	ssaV	2.019	0.624	0	0
9429	1493881	ssaV	2.117	0.561	0	0
3888	1493958	ssaV	2.125	0.536	0	0
10101	1494537	ssaV	2.080	0.625	0	0
3765	1494739	ssaV	2.458	0.527	1	0
6759	1494841	ssaV	2.423	0.602	1	0
10694	1495107	ssaV	2.347	0.463	1	0
14387	1495227	ssaV, ssaN	2.277	0.520	1	0
14246	1495624	ssaV, ssaN	1.462	0.575	0	0
10830	1495853	ssaN	1.331	0.670	0	0
3450	1496043	ssaN	0.993	0.770	0	0
5019	1496278	ssaN	1.160	0.731	0	0
11760	1496545	ssaN, ssaO	1.503	0.720	0	0
10871	1496654	ssaN, ssaO	0.778	0.568	0	0
507	1496857	ssaN, ssaO	1.673	0.883	0	0
4184	1496961	ssaN, ssaO	1.580	0.629	0	0
11141	1497456	ssaP, ssaQ	1.252	0.550	0	0
21586	1497549	ssaP, ssaQ	0.755	0.480	0	0
12005	1497824	ssaQ	0.769	0.688	0	0
7894	1498026	ssaQ	1.510	0.743	0	0
7597	1498332	ssaQ, ssaR	1.975	0.722	0	0
20489	1498409	ssaQ, ssaR	2.155	0.659	1	0
19909	1498721	ssaR	2.954	0.636	1	0
16695	1499038	ssaR, ssaS	2.959	0.599	1	0
17333	1499365	ssaS, ssaT	2.831	0.555	1	0
9077	1499559	ssaS, ssaT	2.603	0.477	1	0
833	1499630	ssaT	2.315	0.448	0	0
6355	1499859	ssaT	1.760	0.506	0	0
6397	1500124	ssaT, ssaU	2.574	0.527	0	0
4549	1500311	ssaT, ssaU	2.472	0.551	0	0
10421	1500639	ssaU	2.042	0.613	0	0
18341	1500759	ssaU	2.573	0.571	0	0
13152	1501074	ssaU	1.852	0.870	0	0
10681	1501162	ssaU	1.432	1.034	0	0

575	1501518		0.536	2.811	0	1
1507	1501834	valW, valV	0.551	3.553	0	1
18371	1502054	valW, valV	0.500	1.418	0	0
7255	1502284	ydhE	0.953	1.415	0	0
20910	1502475	ydhE	1.091	0.964	0	0
19147	1502628	ydhE	1.131	0.791	0	0
15656	1502799	ydhE	0.794	0.768	0	0
1537	1502973	ydhE	1.001	0.969	0	0
19588	1503285	ydhE, ribE	1.230	1.099	0	0
16175	1503676	ydhE, ribE	1.202	1.212	0	0
19191	1503908	ribE	0.779	0.792	0	0
12089	1504024	ribE	1.138	1.280	0	0
5924	1504322	cfa	0.958	0.958	0	0
18308	1504599	cfa	0.827	0.860	0	0
14084	1504687	cfa	1.136	0.883	0	0
22074	1504940	cfa	1.140	0.959	0	0
16467	1505190	cfa	0.803	1.079	0	0
8936	1505406	cfa	1.096	1.332	0	0
5281	1505591	cfa	1.152	1.258	0	0
15838	1505816	cfa, ydhC	0.598	0.800	0	0
8940	1505948	ydhC	1.151	0.932	0	0
20710	1506260	ydhC	1.304	0.861	0	0
22022	1506602	ydhC, ydhB	0.487	0.848	0	0
14379	1506832	ydhC, ydhB	1.232	0.735	0	0
17336	1506904	ydhC, ydhB	0.973	0.653	0	0
1315	1507169	ydhC, ydhB	1.797	0.893	0	0
2143	1507323	ydhB	0.971	0.653	0	0
17694	1507583	ydhB	1.087	1.060	0	0
2087	1507974	purR	1.177	1.371	0	0
1896	1508093	purR	0.969	1.418	0	0
5967	1508229	purR	0.839	1.185	0	0
17451	1508671	purR	1.025	1.652	0	0
14210	1508854	purR	0.543	0.774	0	0
13127	1508925	purR	0.671	1.475	0	0
13918	1509193	purR	0.918	1.820	0	0
17463	1509570	sodB	0.774	1.557	0	0

7553	1509825	sodB	0.839	1.093	0	0
1644	1509935	sodB	0.849	1.107	0	0
2566	1510156	sodB, ydhO	0.686	0.968	0	0
7545	1510457	ydhO	0.775	1.153	0	0
18978	1510673	ydhO	0.908	0.831	0	0
15331	1510869	ydhO, ydhD	0.662	1.068	0	0
10239	1511048	ydhO, ydhD	0.844	1.203	0	0
16455	1511245	ydhO, ydhD	0.772	1.022	0	0
13022	1511316	ydhO, ydhD	0.775	1.140	0	0
8446	1511419	ydhD	0.971	1.338	0	0
19778	1512024	rnt	0.715	1.502	0	0
16233	1512246	rnt	0.893	1.965	0	0
21689	1512318	rnt	0.915	1.854	0	0
15238	1512743	rnt, gloA	0.962	1.332	0	0
18664	1512833	gloA	1.108	1.348	0	0
5563	1512964	gloA, nemA	1.028	1.062	0	0
21564	1513245	gloA, nemA	1.171	0.818	0	0
8160	1513411	nemA	0.659	0.670	0	0
10237	1513810	nemA	0.788	0.539	0	0
2813	1513999	nemA	1.281	0.740	0	0
16768	1514296	nemA, ydhM	1.168	1.108	0	0
9784	1514384	nemA, ydhM	1.259	1.031	0	0
20817	1514720	ydhM, ydhL	0.981	1.250	0	0
237	1514885	ydhM, ydhL	1.318	1.438	0	0
19155	1515025	ydhM, ydhF	1.153	1.391	0	0
2866	1515420	ydhF	1.440	1.062	0	0
11617	1515649	ydhF, sodC	1.087	0.995	0	0
5473	1515766	ydhF, sodC	1.113	1.072	0	0
2142	1515966	sodC	0.978	1.166	0	0
4063	1516166	sodC	0.935	1.496	0	0
11859	1516401	sodC	1.122	1.711	0	0
18997	1516666	STM1441	0.491	0.756	0	0
3474	1516905	STM1441	0.600	0.890	0	0
14182	1517296	STM1441	0.543	0.568	0	0
2512	1517476	STM1441	0.529	0.507	0	0
13499	1517580	STM1441	0.925	0.673	0	0

17303	1517805	STM1441	0.970	0.840	0	0
10114	1518115	STM1441	1.147	0.954	0	0
11228	1518449	STM1441	1.156	1.027	0	0
10353	1518610	STM1441, ydhJ	1.091	0.924	0	0
8258	1518824	STM1441, ydhJ	0.903	0.870	0	0
13637	1519049	ydhJ	0.864	0.896	0	0
7152	1519243	ydhJ	0.423	0.341	0	0
20341	1519490	ydhJ, ydhI	0.681	0.934	0	0
20276	1519686	ydhJ, ydhI	0.627	0.962	0	0
15337	1519860	ydhJ, ydhI	0.575	1.309	0	0
4689	1520229	slyA	0.606	2.280	0	1
18601	1520440	slyB	0.465	1.876	0	1
3224	1520680	slyB, ydhH	0.628	2.281	0	1
14598	1520794	slyB, ydhH	0.557	1.632	0	0
13605	1521068	slyB, ydhH	0.854	1.528	0	0
16715	1521223	slyB, ydhH	0.876	1.148	0	0
9858	1521436	ydhH	0.964	1.005	0	0
8698	1521746	ydhH	1.276	1.127	0	0
21265	1521820	ydhH, ydhA	1.031	1.051	0	0
19129	1522297	ydhA, pdxH	1.238	1.018	0	0
17594	1522442	ydhA, pdxH	1.092	1.051	0	0
7287	1522604	ydhA, pdxH	0.716	0.782	0	0
17100	1522873	pdxH	0.790	1.204	0	0
16606	1522972	pdxH	0.764	1.394	0	0
4390	1523448	tyrS	0.642	2.628	0	1
21576	1523603	tyrS	0.646	1.925	0	1
12910	1523909	tyrS	0.886	1.514	0	0
10450	1523980	tyrS	0.656	1.414	0	0
19541	1524234	tyrS	0.687	1.245	0	0
8448	1524316	tyrS	1.107	1.340	0	0
3483	1524633	tyrS, pdxY	0.988	1.373	0	0
20935	1524809	pdxY	0.934	1.324	0	0
3318	1525048	pdxY	0.779	1.322	0	0
18435	1525247	pdxY	0.893	1.375	0	0
16035	1525565	pdxY	0.836	1.592	0	0
3861	1525905	gst	0.646	1.362	0	0

18207	1526030	gst	0.744	1.694	0	0
7448	1526329	gst	0.488	1.574	0	0
13430	1526427	gst, ydgR	0.642	2.484	0	1
379	1526556	gst, ydgR	0.646	2.873	0	1
2342	1527002	ydgR	0.689	1.909	0	1
5317	1527259	ydgR	0.581	1.420	0	0
4645	1527448	ydgR	0.885	1.492	0	0
10587	1527757	ydgR	0.657	1.553	0	0
15868	1527850	ydgR	0.723	1.235	0	0
17692	1528189	ydgR	0.805	0.998	0	0
10429	1528429		0.755	0.806	0	0
11553	1528532	nth	0.684	0.874	0	0
11220	1528628	nth	0.651	0.823	0	0
18803	1528933	nth	0.960	1.119	0	0
8058	1529160	nth	0.718	0.844	0	0
14380	1529581	nth, ydgQ	0.707	0.876	0	0
22302	1529827	ydgQ	1.067	1.279	0	0
5201	1529903	ydgQ, ydgP	0.745	0.959	0	0
5139	1530038	ydgQ, ydgP	1.190	1.293	0	0
21287	1530377	ydgP	0.743	0.885	0	0
21089	1530471	ydgP	1.073	1.290	0	0
7154	1530543	ydgP, ydgO	0.965	1.236	0	0
14659	1531124	ydgO	1.409	1.162	0	0
5705	1531228	ydgO	1.137	1.081	0	0
18743	1531533	ydgO	1.637	1.862	0	1
3231	1531725	ydgO, STM1457	1.442	2.038	0	1
9841	1531922	ydgO, STM1457	0.862	1.184	0	0
16218	1532219	STM1457	0.640	0.830	0	0
19688	1532422	STM1457	1.672	1.423	0	0
5302	1532521	STM1457	1.141	1.149	0	0
4978	1532697	STM1457	1.180	1.084	0	0
4930	1533113	STM1457	1.275	1.517	0	0
3438	1533231	STM1457	1.013	1.304	0	0
15552	1533396	STM1457	0.340	0.626	0	0
8024	1533759	STM1457, ydgM	0.889	1.058	0	0
15539	1533976	STM1457, ydgM	0.783	1.733	0	0

18558	1534322	ydgM, STM1459	0.675	1.279	0	0
20067	1534535	ydgM, STM1459	0.587	1.331	0	0
13515	1534646	ydgM, STM1459	0.599	1.803	0	1
20985	1534867	STM1459	0.525	2.280	0	1
7061	1535224	STM1459, ydgK	0.416	2.090	0	1
17976	1535458	ydgK	0.615	2.565	0	1
21176	1535643	ydgK, ydgT	0.693	1.671	0	0
16604	1535908	ydgT, ydgJ	0.682	1.824	0	0
15969	1536068	ydgT, ydgJ	0.393	0.671	0	0
21853	1536267	ydgJ	0.761	1.443	0	0
2835	1536399	ydgJ	0.745	1.549	0	0
5579	1536552	ydgJ	0.615	1.010	0	0
21230	1536632	ydgJ	0.747	1.156	0	0
18457	1537101	ydgJ	0.869	1.355	0	0
540	1537315	ydgJ	0.803	1.736	0	1
12547	1537749	add	0.820	1.581	0	1
16214	1537825	add	0.720	1.764	0	1
13936	1538198	add	0.805	1.341	0	0
12362	1538311	add	0.715	1.106	0	0
1608	1538520	add	0.986	1.324	0	0
15357	1538825	add	0.818	1.302	0	0
2425	1538968		0.836	1.397	0	0
7421	1539314	ydgA	0.949	1.360	0	0
8082	1539429	ydgA	0.881	1.402	0	0
14529	1539823	ydgA	0.860	0.889	0	0
10340	1539894	ydgA	0.968	1.109	0	0
6659	1540018	ydgA	1.173	1.119	0	0
7165	1540299	ydgA	0.956	1.075	0	0
519	1540681	ydgA	1.492	1.281	0	0
13920	1540785	ydgA, manA	1.036	1.135	0	0
16676	1541099	ydgA, manA	0.967	0.982	0	0
20020	1541273	manA	0.998	0.925	0	0
6438	1541557	manA	0.820	0.712	0	0
5308	1541648	manA	1.003	1.022	0	0
16322	1541983	manA, fumA	0.890	1.285	0	0
16120	1542170	manA, fumA	0.805	1.529	0	0

5304	1542243	manA, fumA	0.860	1.584	0	0
7538	1542682	fumA	0.928	1.432	0	0
1822	1542983	fumA	0.856	1.678	0	0
5377	1543150	fumA	1.111	1.907	0	0
5929	1543390	fumA	0.806	1.426	0	0
8867	1543665	fumA, fumC	0.718	1.245	0	0
3875	1543906	fumC	0.884	1.561	0	0
3373	1544075	fumC	0.932	1.606	0	0
18204	1544366	fumC	0.963	1.225	0	0
10449	1544596	fumC	0.783	1.159	0	0
7905	1544785	fumC	1.028	1.388	0	0
2548	1544913	fumC	0.947	1.327	0	0
3140	1545060	fumC	1.054	1.650	0	0
6968	1545199	fumC	0.712	1.289	0	0
15181	1545571	tus	0.684	1.574	0	0
11345	1545741	tus	1.055	1.731	0	0
8151	1545983	tus	1.030	1.147	0	0
22047	1546296	tus	1.002	1.321	0	0
2925	1546550	tus, rstB	0.862	0.861	0	0
2991	1546674	tus, rstB	0.841	0.746	0	0
2229	1547040	rstB	1.227	0.843	0	0
3495	1547122	rstB	0.972	0.716	0	0
5062	1547466	rstB	0.961	0.744	0	0
4338	1547639	rstB	0.848	0.767	0	0
18300	1547875	rstB, STM1472	1.358	1.194	0	0
13329	1548076	rstB, STM1472	1.188	0.999	0	0
21634	1548337	STM1472	1.456	0.782	0	0
10289	1548614	STM1472	0.440	0.491	0	0
21065	1548824	STM1472	1.476	0.711	0	0
14012	1548972	STM1472	1.681	0.622	0	0
2621	1549145	STM1472	1.100	0.530	0	0
16483	1549551	STM1472	2.008	0.615	0	0
3822	1549807	STM1472, ompN	2.592	0.498	1	0
12496	1549916	STM1472, ompN	3.056	0.631	1	0
10591	1550010	ompN	3.246	0.561	1	0
12876	1550242	ompN	1.516	0.581	0	0

3392	1550637	ompN	2.488	0.563	1	0
7018	1550816	ompN	2.906	0.595	1	0
19286	1551113	ompN	2.528	0.660	1	0
18585	1551246	ompN	2.089	0.965	0	0
7376	1551320	rstA	1.627	0.889	0	0
3876	1551815	rstA, ydgC	0.982	2.139	0	1
17995	1552056	rstA, ydgC	1.242	1.758	0	1
19232	1552212	rstA, ydgC	1.266	1.463	0	0
13458	1552318	rstA, ydgC	1.474	1.287	0	0
1064	1552451	rstA, ydgC	1.672	1.023	0	0
5167	1552807	ydgI	0.953	0.632	0	0
5307	1553018	ydgI	0.361	0.481	0	0
221	1553350	ydgI	1.335	0.657	0	0
18376	1553574	ydgI	1.328	0.773	0	0
11243	1553893	ydgI	1.152	1.033	0	0
4971	1554052	ydgI	1.286	1.272	0	0
9838	1554327	ydgI, ydgH	0.950	1.269	0	0
4104	1554403	ydgH	0.550	0.868	0	0
1934	1554813	ydgH	0.872	1.327	0	0
15684	1554927	ydgH	0.672	0.858	0	0
17790	1555183	ydgH	1.004	1.317	0	0
15525	1555410	ydgH, pntA	1.003	1.120	0	0
7349	1555646	pntA	1.068	0.961	0	0
17695	1555889	pntA	1.097	1.221	0	0
18894	1555983	pntA	0.691	0.856	0	0
11086	1556288	pntA	0.574	0.585	0	0
11677	1556583	pntA	1.089	1.371	0	0
5828	1556662	pntA	0.699	0.794	0	0
1683	1557047	pntA, pntB	1.163	1.273	0	0
7204	1557198	pntB	1.284	1.162	0	0
2074	1557458	pntB	0.885	1.073	0	0
19283	1557612	pntB	0.989	1.209	0	0
6445	1557988	pntB	1.011	1.250	0	0
21734	1558121	pntB	0.613	0.858	0	0
12690	1558319	pntB	1.057	1.124	0	0
20704	1558467	pntB	0.640	0.742	0	0

11507	1558783	STM1481	0.907	0.761	0	0
21556	1558921	STM1481	1.291	0.990	0	0
6142	1559353	STM1481	1.585	0.807	0	0
6886	1559530	STM1481	1.710	0.704	0	0
1517	1559789	STM1481, ydgF	1.455	1.023	0	0
9370	1559939	STM1481, ydgF	2.142	1.156	0	0
1743	1560229	STM1481, ydgF	1.980	1.430	0	0
679	1560382	ydgF, ydgE	2.551	1.342	1	0
4704	1560460	ydgF, ydgE	2.729	1.209	1	0
8642	1560636	ydgE	1.669	0.909	0	0
11203	1560882	ydgE	1.142	1.193	0	0
5918	1561296	STM1484	1.500	1.267	0	0
15361	1561501	STM1484	0.907	0.943	0	0
11184	1561727	STM1484	0.920	0.645	0	0
4061	1561851	STM1484	1.650	0.657	0	0
21473	1562276	STM1485	0.380	0.534	0	0
15675	1562351	STM1485	1.260	0.995	0	0
16943	1562518	STM1485	1.374	0.965	0	0
3821	1562589	STM1485	1.304	0.988	0	0
16801	1562825	ynfM	1.027	0.903	0	0
6118	1563444	ynfM	1.417	0.863	0	0
1780	1563573	ynfM	1.059	0.688	0	0
6679	1563860	ynfM, ynfL	1.485	0.788	0	0
10707	1563932	ynfM, ynfL	1.690	0.818	0	0
6110	1564077	ynfM, ynfL	1.281	0.651	0	0
18019	1564408	ynfL	0.989	0.682	0	0
7973	1564622	ynfL, mlc	1.077	0.892	0	0
3974	1564939	ynfL, mlc	1.050	1.054	0	0
14566	1565039	mlc	1.191	1.189	0	0
19163	1565241	mlc	1.215	1.413	0	0
22356	1565515	mlc	0.918	1.383	0	0
2232	1565586	mlc	0.948	1.231	0	0
2694	1566067	mlc, ynfK	1.095	1.441	0	0
21149	1566338	ynfK	0.863	1.082	0	0
7656	1566445	ynfK	0.901	1.033	0	0
21312	1566839	ynfK	0.846	0.936	0	0

4703	1566958	ynfK	1.105	1.094	0	0
22247	1567283	STM1490	1.130	1.117	0	0
5981	1567488	STM1490	1.301	1.020	0	0
5164	1567715	STM1490	1.048	0.626	0	0
20925	1567793	STM1490	1.207	0.843	0	0
14922	1568040	STM1490	1.310	1.133	0	0
5847	1568366	STM1490	1.188	0.981	0	0
16017	1568647	STM1490, STM1491	0.909	0.940	0	0
19455	1568909	STM1491	0.940	0.941	0	0
776	1569068	STM1491	0.749	0.573	0	0
7163	1569359	STM1491	1.605	0.872	0	0
16734	1569440	STM1491	1.236	0.819	0	0
7949	1569653	STM1491, STM1492	1.242	0.847	0	0
1979	1569857	STM1491, STM1492	1.311	0.922	0	0
1265	1570083	STM1491, STM1492	1.659	1.033	0	0
14817	1570302	STM1492, STM1493	1.063	0.783	0	0
1901	1570511	STM1492, STM1493	1.077	0.881	0	0
13536	1570914	STM1493	1.690	0.678	0	0
21412	1571066	STM1493	1.882	0.705	0	0
6103	1571190	STM1493	1.414	0.624	0	0
18755	1571345	STM1493, STM1494	1.416	0.599	0	0
17295	1571792	STM1494	1.042	0.609	0	0
4347	1572047	STM1494	1.113	0.652	0	0
19372	1572261	STM1494, ynfI	1.141	0.890	0	0
1851	1572488	ynfI	1.380	0.874	0	0
4814	1572559	ynfI	1.266	0.908	0	0
2283	1572846	ynfI	1.417	1.209	0	0
10485	1573149	ynfI, STM1496	1.119	1.096	0	0
1939	1573346	STM1496	1.378	1.770	0	0
463	1573590	STM1496	0.424	0.796	0	0
12736	1573671	STM1496	0.409	0.587	0	0
20329	1573985	STM1496, STM1497	1.202	0.764	0	0
10157	1574203	STM1496, STM1497	1.443	0.692	0	0
1872	1574410	STM1497, STM1498	1.319	0.559	0	0
17491	1574827	STM1497, STM1498	1.349	0.814	0	0
7672	1574903	STM1498	0.703	0.651	0	0

13961	1575000	STM1498	0.975	0.687	0	0
14443	1575255	STM1498	1.246	0.610	0	0
8367	1575529	STM1498	1.669	0.710	0	0
12440	1575891	STM1498	1.448	0.754	0	0
8243	1576132	STM1498	0.744	0.683	0	0
8804	1576351	STM1498	1.275	0.680	0	0
4388	1576437	STM1498	1.169	0.688	0	0
7329	1576820	STM1498	1.072	0.493	0	0
11896	1576941	STM1498, STM1499	1.124	0.651	0	0
16615	1577250	STM1498, STM1499	1.358	0.598	0	0
1401	1577494	STM1499	1.505	0.710	0	0
1514	1577752	STM1499	1.170	0.730	0	0
4156	1577826	STM1499	1.579	0.770	0	0
13311	1577943	STM1499	1.005	0.681	0	0
13527	1578431	STM1499	1.377	0.791	0	0
10175	1578674	STM1499	1.303	0.788	0	0
20769	1578896	STM1499	1.247	0.726	0	0
18615	1579120	STM1499	1.259	0.721	0	0
13785	1579364	STM1499	0.682	0.606	0	0
12800	1579467	STM1499, ynfC	1.550	0.715	0	0
8835	1579823	STM1499, ynfD	1.181	0.963	0	0
13885	1579940	ynfD, ynfC	1.022	0.836	0	0
13028	1580156	ynfD, ynfC	1.031	1.053	0	0
5336	1580234	ynfD, ynfC	0.967	1.157	0	0
7168	1580605	ynfC	0.721	1.489	0	0
6699	1580791	speG	0.738	1.120	0	0
15008	1581126	speG	0.851	1.253	0	0
10283	1581252	speG	0.662	1.113	0	0
15651	1581532	speG, ynfB	0.961	1.272	0	0
13239	1581630	speG, ynfB	0.918	1.095	0	0
5633	1581772	ynfB, ynfA	0.883	1.120	0	0
18812	1582162	rspA	0.803	0.671	0	0
21536	1582264	rspA	1.021	0.771	0	0
1989	1582511	rspA	1.288	0.668	0	0
2070	1582780	rspA	1.200	0.627	0	0
21784	1583021	rspA, rspB	1.476	0.711	0	0

3013	1583442	rspB	4.254	0.700	0	0
12754	1583574	rspB	1.555	0.646	0	0
7761	1583726	rspB	0.835	0.460	0	0
10312	1584009	rspB	1.220	0.578	0	0
16271	1584341	rspB, ydfJ	2.264	0.478	0	0
18173	1584516	ydfJ	3.144	0.469	0	0
21595	1584799	ydfJ	1.265	0.523	0	0
15125	1584871	ydfJ	2.886	0.723	1	0
699	1585165	ydfJ	4.968	0.779	1	0
21508	1585389	ydfJ	4.866	0.553	1	0
21673	1585499		4.623	0.711	1	0
3884	1585768	ydfI	2.229	0.661	0	0
11577	1586037	ydfI	2.143	0.852	0	0
4519	1586189	ydfI	3.809	0.697	0	0
17089	1586401	ydfI	1.311	0.987	0	0
5809	1586683	ydfI	1.329	0.882	0	0
8805	1586952	ydfI	0.968	0.740	0	0
7992	1587076	ydfI	0.771	0.628	0	0
16405	1587444	ydfI	1.459	1.196	0	0
18986	1587759	ydfZ	1.074	1.203	0	0
6873	1587860	ydfZ	1.048	1.136	0	0
14040	1588155	ydfZ, ydfH	1.144	1.318	0	0
19172	1588397	ydfH	0.732	0.853	0	0
15230	1588673	ydfH	0.904	1.046	0	0
14543	1588816	ydfH, ydfG	0.750	0.862	0	0
1524	1588938	ydfH, ydfG	1.215	1.503	0	0
17635	1589220	ydfG, dcp	1.191	1.221	0	0
13260	1589534	ydfG, dcp	0.770	0.798	0	0
10744	1589664	ydfG, dcp	1.305	1.046	0	0
20876	1589739	ydfG, dcp	1.160	0.876	0	0
21383	1590150	dcp	1.638	0.904	0	0
8969	1590432	dcp	0.988	0.728	0	0
11254	1590607	dcp	1.224	0.767	0	0
5851	1590725	dcp	1.542	0.834	0	0
2587	1591010	dcp	1.419	0.776	0	0
13170	1591279	dcp	1.825	0.786	0	0

16736	1591608	dcp, STM1513	2.914	0.675	0	0
9583	1591759	STM1513	3.373	0.512	0	0
11199	1591961	STM1513	5.192	0.570	1	0
528	1592215	STM1513	3.786	0.584	1	0
14355	1592420	ydeJ	3.601	0.552	1	0
8982	1592669	ydeJ	2.693	0.608	1	0
7687	1592861	ydeJ, ydeI	2.563	0.530	1	0
10533	1593099	ydeJ, ydeI	3.360	0.624	1	0
16535	1593286	ydeJ, ydeI	2.943	0.637	1	0
5271	1593429	ydeI	2.438	0.785	1	0
19843	1593830		0.522	0.725	0	0
20065	1593905		1.384	1.639	0	0
9183	1594162		2.601	0.926	1	0
3571	1594246		2.834	0.955	1	0
7585	1594672	ydeE	4.222	0.953	1	0
9123	1594815	ydeE	2.349	0.780	1	0
8119	1595170	ydeE	1.247	0.708	0	0
1232	1595277	ydeE	1.120	0.742	0	0
21252	1595565	ydeE, ydeD	1.034	0.973	0	0
14767	1595683	ydeE, ydeD	0.902	0.734	0	0
16789	1595771	ydeE, ydeD	1.048	0.781	0	0
22125	1596216	ydeD	0.791	0.647	0	0
13337	1596433	ydeD	1.278	0.825	0	0
17107	1596715		1.193	0.961	0	0
20763	1596980	marB	1.380	0.930	0	0
22169	1597173	marB, marA	1.401	0.949	0	0
19753	1597409	marB, marA	1.449	0.862	0	0
4480	1597715	marA, marR	0.815	0.604	0	0
12132	1597860	marA, marR	1.849	0.912	0	0
9776	1598177	marR, marC	0.864	0.851	0	0
9656	1598308	marR, marC	1.039	1.082	0	0
19100	1598500	marC	0.959	1.167	0	0
3754	1598813	marC	1.269	1.152	0	0
15510	1598889	ydeA	1.218	1.102	0	0
12530	1599126	ydeA	1.157	1.075	0	0
17907	1599394	ydeA	1.009	0.903	0	0

8309	1599804	ydeA	0.799	0.492	0	0
9096	1599878	ydeA	1.093	0.713	0	0
13111	1600066	ydeA	1.495	0.983	0	0
17110	1600137	ydeA	1.125	1.065	0	0
8207	1600481	ydeA, yneJ	1.537	0.868	0	0
4887	1600699	yneJ, yneI	1.309	0.677	0	0
16373	1601145	yneJ, yneI	1.590	0.707	0	0
12297	1601378	yneJ, yneI	1.592	0.768	0	0
12852	1601498	yneJ, yneI	1.424	0.718	0	0
5843	1601729	yneI	1.251	0.571	0	0
4635	1601862	yneI	0.914	0.542	0	0
21835	1602231	yneI, yneH	0.684	0.522	0	0
4601	1602514	yneI, yneH	1.171	0.528	0	0
12974	1602627	yneH	0.634	0.407	0	0
3426	1602931	yneH	1.076	0.703	0	0
18837	1603150	yneH, yneG	1.133	0.772	0	0
7850	1603225	yneH, yneG	0.976	0.711	0	0
16315	1603611	yneG	1.245	1.174	0	0
19774	1603865	yneG, STM1527	0.824	0.781	0	0
6983	1604123	STM1527	1.022	0.763	0	0
6622	1604270	STM1527	0.929	0.702	0	0
6311	1604485	STM1527	1.099	0.755	0	0
15356	1604824	STM1527	1.268	0.667	0	0
562	1604937	STM1527	1.451	0.747	0	0
12507	1605001	STM1527	1.448	0.689	0	0
2701	1605457	STM1529, STM1528	1.652	0.552	0	0
2433	1605538	STM1529, STM1528	1.819	0.514	0	0
10807	1605700	STM1529, STM1528	2.083	0.445	0	0
17040	1606112	STM1528, STM1530	2.279	0.547	0	0
740	1606307	STM1530	1.860	0.455	0	0
2797	1606571	STM1530	1.836	0.678	0	0
10757	1606716	STM1530	1.428	0.510	0	0
7572	1606805	STM1530	1.595	0.471	0	0
10123	1607260	STM1530	2.483	0.546	1	0
17728	1607512		2.519	0.629	1	0
7342	1607733	STM1531	1.462	0.415	0	0

4202	1607928	STM1531	1.710	0.628	0	0
17227	1608051	STM1531, STM1532	1.883	0.610	0	0
22291	1608421	STM1531, STM1532	0.877	0.587	0	0
13985	1608552	STM1532	0.810	0.459	0	0
10606	1608896	STM1532, STM1533	1.345	0.783	0	0
4421	1609081	STM1532, STM1533	1.148	0.661	0	0
2879	1609295	STM1532, STM1533	1.469	0.663	0	0
14516	1609382	STM1533	1.277	0.741	0	0
20643	1609685	STM1533	1.645	0.739	0	0
11448	1609915	STM1533	1.585	0.796	0	0
14386	1610121	STM1533, STM1534	1.747	0.952	0	0
6894	1610353	STM1533, STM1534	1.364	0.922	0	0
21068	1610648	STM1534, STM1535	1.173	1.192	0	0
713	1610752	STM1534, STM1535	1.443	1.047	0	0
9329	1610970	STM1535, STM1536	0.982	0.708	0	0
14584	1611086	STM1535, STM1536	1.171	0.848	0	0
16255	1611468	STM1536, STM1537	1.227	0.749	0	0
949	1611783	STM1537	1.305	0.842	0	0
2936	1611928	STM1537	1.276	0.733	0	0
1887	1612192	STM1537, STM1538	0.582	0.424	0	0
15627	1612450	STM1537, STM1538	1.581	0.669	0	0
6713	1612605	STM1538	0.899	0.555	0	0
13212	1612935	STM1538	1.476	0.788	0	0
15824	1613113	STM1538	1.320	0.620	0	0
18025	1613438	STM1538	1.339	0.719	0	0
17967	1613640	STM1538	0.868	0.445	0	0
7843	1613761	STM1538	1.269	0.566	0	0
18004	1613973	STM1538, STM1539	1.436	0.796	0	0
305	1614193	STM1538, STM1539	1.528	0.762	0	0
20508	1614329	STM1539	1.423	0.740	0	0
10158	1614755	STM1539	1.968	0.522	0	0
9340	1614841	STM1539, STM1540	1.570	0.468	0	0
19088	1615140	STM1539, STM1540	2.996	0.461	1	0
16947	1615442	STM1540	3.074	0.537	1	0
6774	1615579	STM1540	2.665	0.534	1	0
14129	1615883	STM1540	1.849	0.596	0	0

18433	1615963	STM1540	1.990	0.687	0	0
15329	1616187	STM1540, STM1541	1.555	0.633	0	0
3181	1616402	STM1541	1.478	0.623	0	0
4791	1616773	STM1541	1.168	0.693	0	0
12718	1616901	STM1541	1.190	0.717	0	0
2298	1617116	STM1541	0.939	0.576	0	0
22330	1617381	STM1542	0.947	0.718	0	0
12848	1617549	STM1542	1.062	0.604	0	0
19634	1617980	STM1542	1.603	0.487	0	0
6574	1618135	STM1542	2.928	0.557	1	0
3074	1618344	STM1542, STM1543	3.535	0.491	1	0
992	1618566	STM1542, STM1543	2.876	1.567	1	0
15714	1618681	STM1542, STM1543	2.402	0.472	0	0
16815	1619029	STM1543	2.921	0.640	1	0
22012	1619319	STM1543	3.097	0.632	1	0
21769	1619480	STM1543	1.689	0.549	0	0
18256	1619572	STM1543	0.777	0.482	0	0
10218	1619904	STM1543, pqaA	2.376	0.557	0	0
6045	1620146	pqaA	2.511	0.568	1	0
21944	1620349	pqaA	2.827	0.583	1	0
15144	1620669	pqaA	2.518	0.583	1	0
10975	1620813	pqaA	2.450	0.481	1	0
1123	1621029	pqaA	2.767	0.497	1	0
7138	1621151	pqaA	2.468	0.489	1	0
18834	1621397	pqaA, STM1545	2.260	0.520	0	0
6084	1621771	pqaA, STM1545	1.782	0.693	0	0
1421	1621891	STM1545	3.077	0.772	0	0
3668	1622031	STM1545	0.777	0.508	0	0
19630	1622167	STM1545	0.897	0.673	0	0
8920	1622670	STM1545, STM1546	1.252	0.602	0	0
2121	1622862	STM1545, STM1546	1.325	0.737	0	0
4948	1623037	STM1545, STM1546	1.182	0.763	0	0
780	1623342	STM1546	1.572	0.739	0	0
18626	1623509	STM1546	1.229	0.748	0	0
4616	1623772	STM1546, STM1547	1.196	0.681	0	0
15755	1624111	STM1546, STM1547	0.750	0.565	0	0

2744	1624322	STM1546, STM1547	1.523	0.649	0	0
14574	1624575	STM1546, STM1547	1.787	0.593	0	0
18218	1624768	STM1548	2.273	0.502	0	0
16473	1624940	STM1548	2.179	0.513	1	0
20471	1625197	STM1548	2.948	0.542	1	0
13392	1625306	STM1548	2.829	0.671	1	0
9971	1625534	STM1548	2.185	0.585	0	0
5505	1625884	STM1548	1.341	0.655	0	0
3658	1626128	STM1549	0.840	0.660	0	0
17339	1626297	STM1549	0.895	0.746	0	0
3089	1626526	STM1549	0.827	0.760	0	0
13245	1626598	STM1549, STM1550	0.876	0.799	0	0
10145	1627011	STM1550, STM1551	1.066	0.723	0	0
13529	1627268	STM1550, STM1551	1.383	0.665	0	0
563	1627527	STM1551, STM1552	1.389	0.587	0	0
15457	1627628	STM1552	1.146	0.575	0	0
404	1627934	STM1552	2.053	0.574	0	0
1792	1628154	STM1552	1.984	0.540	0	0
11986	1628432	STM1552	1.694	0.621	0	0
16300	1628523	STM1552	1.888	0.601	0	0
21531	1628664	STM1552	1.581	0.676	0	0
12403	1628797	STM1552	1.484	0.714	0	0
3713	1629342		1.231	0.581	0	0
3227	1629590		1.206	0.721	0	0
12744	1629726		1.056	0.612	0	0
10645	1629803		0.674	0.600	0	0
21194	1630144		2.104	0.714	0	0
20934	1630339		2.859	0.521	1	0
19085	1630569	STM1554	2.860	0.477	1	0
17919	1630747	STM1554	2.365	0.436	0	0
6851	1631089	STM1554	1.910	0.513	0	0
20512	1631193	STM1554	1.886	0.493	0	0
13965	1631480	STM1554	2.616	0.515	1	0
4813	1631672	STM1554, STM1555	2.261	0.517	1	0
20771	1631891	STM1554, STM1555	2.182	0.415	0	0
354	1632047	STM1554, STM1555	2.367	0.628	0	0

7384	1632425	STM1555	1.494	0.649	0	0
4234	1632668	STM1555	1.156	0.593	0	0
11987	1632739	STM1555	1.170	0.570	0	0
19031	1633179	STM1556	1.153	0.623	0	0
15003	1633319	STM1556	1.028	0.712	0	0
17943	1633595	STM1556	1.221	0.903	0	0
13767	1633831	STM1556	1.239	0.745	0	0
18984	1634114	STM1556	1.104	0.703	0	0
21420	1634237	STM1556	1.198	0.802	0	0
20063	1634315	STM1556, STM1557	0.689	0.594	0	0
21617	1634633	STM1556, STM1557	1.561	0.896	0	0
19978	1634715	STM1557	1.470	0.649	0	0
20518	1635101	STM1557	1.226	0.783	0	0
17609	1635449	STM1557	1.691	1.068	0	0
1077	1635685	STM1557	2.455	1.089	0	0
2762	1635833	STM1557	1.629	1.018	0	0
15949	1635904	STM1557	1.668	0.979	0	0
18649	1636265	STM1558	1.094	0.893	0	0
10272	1636459	STM1558	1.159	0.817	0	0
19028	1636698	STM1558	1.383	0.849	0	0
12273	1636852	STM1558	1.138	0.905	0	0
10687	1637163	STM1558	0.797	0.684	0	0
2072	1637235	STM1558	0.963	0.778	0	0
19699	1637732	STM1558	1.388	0.695	0	0
19775	1637931	STM1558	1.311	0.704	0	0
6867	1638086	STM1558	1.432	0.596	0	0
8771	1638263	STM1558, STM1559	1.289	0.628	0	0
6126	1638650	STM1559	1.321	0.666	0	0
22338	1638837	STM1559	1.072	0.812	0	0
22080	1638917	STM1559	1.376	0.826	0	0
5464	1639211	STM1559	1.075	0.692	0	0
12282	1639465	STM1559	1.244	0.576	0	0
9287	1639642	STM1559	1.525	0.758	0	0
13643	1639921	STM1559	1.947	0.867	0	0
19553	1640041	STM1559	0.536	0.552	0	0
18746	1640457	STM1559	1.225	0.715	0	0

17861	1640594	STM1559	1.362	0.613	0	0
9746	1640714	STM1559, STM1560	1.316	0.676	0	0
10528	1641146	STM1559, STM1560	1.655	0.660	0	0
13246	1641319	STM1560	1.198	0.600	0	0
18722	1641571	STM1560	1.520	1.026	0	0
19342	1641703	STM1560	1.529	0.622	0	0
16202	1641867	STM1560	1.600	0.839	0	0
8240	1642127	STM1560	0.678	0.585	0	0
7632	1642528	STM1560	1.234	0.566	0	0
4751	1642600	STM1560, STM1561	1.388	0.588	0	0
682	1642918	STM1560, STM1561	0.987	0.469	0	0
15156	1643188	STM1561	2.163	0.590	1	0
5415	1643412	STM1561	2.435	0.568	1	0
3878	1643662	STM1561, STM1562	2.448	0.663	0	0
7804	1643846	STM1561, STM1562	3.287	0.512	1	0
15988	1643949	STM1562	2.904	0.725	1	0
21439	1644022	STM1562	3.578	0.745	1	0
17589	1644598	osmC	1.735	0.694	0	0
12805	1644670	osmC	2.307	0.582	0	0
20331	1644775	osmC, yddX	2.687	0.685	1	0
19475	1645161	yddX, rpsV	3.610	0.606	1	0
14907	1645447	rpsV, sfcA	1.868	0.697	0	0
19261	1645619	sfcA	1.318	0.743	0	0
7797	1645776	sfcA	1.456	0.994	0	0
14453	1645987	sfcA	1.137	0.830	0	0
21494	1646214	sfcA	1.164	0.960	0	0
4615	1646460	sfcA	1.252	1.005	0	0
7718	1646797	sfcA	1.149	1.147	0	0
21322	1646868	sfcA	1.135	1.051	0	0
4990	1647151	sfcA	1.678	1.100	0	0
859	1647529	adhP	1.713	0.944	0	0
11060	1647734	adhP	1.851	0.781	0	0
17689	1647867	adhP	0.874	0.384	0	0
11723	1648040	adhP	1.377	0.675	0	0
12699	1648411	adhP	1.407	0.825	0	0
16841	1648621	adhP	0.822	0.806	0	0

4041	1648778		0.787	0.604	0	0
14550	1648987	fdnI	1.373	0.901	0	0
5296	1649291	fdnI	1.423	0.786	0	0
1749	1649524	fdnI, fdnH	1.074	0.809	0	0
8578	1649666	fdnI, fdnH	1.516	0.819	0	0
7560	1649846	fdnI, fdnH	1.092	0.702	0	0
19158	1650225	fdnH	0.892	0.719	0	0
20538	1650422	fdnH, fdnG	1.062	0.770	0	0
6455	1650647	fdnH, fdnG	0.900	0.813	0	0
8078	1650751	fdnH, fdnG	1.251	0.720	0	0
14328	1651045	fdnG	1.065	0.908	0	0
21911	1651366	fdnG	1.168	0.803	0	0
13500	1651479	fdnG	1.341	0.825	0	0
17393	1651837	fdnG	1.429	0.848	0	0
2093	1651941	fdnG	1.703	0.847	0	0
7217	1652086	fdnG	1.074	0.637	0	0
18637	1652429	fdnG	1.333	0.932	0	0
16168	1652579	fdnG	1.250	0.942	0	0
4424	1652699	fdnG	1.054	0.967	0	0
12954	1653075	fdnG	1.287	1.320	0	0
10824	1653254	fdnG	1.177	0.897	0	0
5048	1653454	fdnG, yddG	1.072	0.619	0	0
7401	1653743	fdnG, yddG	1.204	0.633	0	0
10076	1654105	yddG	2.832	0.706	1	0
13218	1654185	yddG	3.613	0.778	1	0
6265	1654460	yddG	3.018	0.867	1	0
7639	1654719	nmpC	2.041	0.829	0	0
18388	1654931	nmpC	1.708	0.983	0	0
6504	1655092	nmpC	2.381	1.523	0	0
10776	1655283	nmpC	1.740	1.757	0	0
1189	1655398	nmpC	1.452	2.418	0	1
19755	1655756	nmpC	0.762	2.568	0	1
7803	1655931	nmpC, STM1573	0.678	2.354	0	1
14979	1656240	STM1573	0.681	2.086	0	1
12019	1656592	STM1573	0.962	1.680	0	0
5564	1656779		0.874	1.289	0	0

18299	1657036	smvA	1.380	1.429	0	0
7692	1657238	smvA	1.060	0.757	0	0
8547	1657528	smvA	1.205	0.821	0	0
13282	1657657	smvA	1.170	0.835	0	0
1498	1657783	smvA	1.375	0.839	0	0
16343	1658120	smvA, STM1575	0.420	0.564	0	0
3792	1658236	smvA, STM1575	1.302	1.170	0	0
19571	1658328	smvA, STM1575	0.932	0.938	0	0
5718	1658753	STM1575, narU	1.523	1.274	0	0
17341	1658942	STM1575, narU	2.026	1.121	0	0
12737	1659190	narU	1.950	0.745	0	0
15968	1659487	narU	2.597	0.697	0	0
14420	1659650	narU	1.054	0.404	0	0
14278	1659759	narU	1.876	0.647	0	0
6973	1660158	narU	1.284	0.669	0	0
12184	1660248	narU, narZ	0.943	0.689	0	0
18458	1660647	narZ	1.225	0.896	0	0
18950	1660718	narZ	1.223	1.087	0	0
16825	1660990	narZ	1.334	0.987	0	0
6895	1661204	narZ	1.144	0.759	0	0
580	1661606	narZ	2.039	0.916	0	0
14803	1661751	narZ	0.758	0.516	0	0
21760	1661920	narZ	0.777	0.635	0	0
12992	1662135	narZ	2.061	1.018	0	0
14933	1662310	narZ	1.772	0.899	0	0
6239	1662758	narZ	1.253	0.623	0	0
20849	1662985	narZ	0.651	0.590	0	0
1169	1663176	narZ	0.918	0.479	0	0
20132	1663437	narZ	1.606	0.720	0	0
3704	1663627	narZ	0.799	0.615	0	0
15312	1663722	narZ	1.640	0.869	0	0
20758	1664069	narZ, narY	1.337	0.658	0	0
9660	1664308	narZ, narY	1.570	0.783	0	0
22294	1664401	narY	1.052	0.650	0	0
21276	1664693	narY	1.308	0.675	0	0
10884	1664797	narY	0.644	0.426	0	0

14749	1665064	narY	1.307	0.698	0	0
6189	1665269	narY	1.100	0.650	0	0
886	1665687	narY, narW	1.381	0.683	0	0
8572	1665897	narY, narW	1.458	0.748	0	0
13247	1665972	narW	1.214	0.559	0	0
3776	1666090	narW	0.948	0.589	0	0
6086	1666542	narW, narV	1.175	0.768	0	0
1710	1666653	narV	1.046	0.735	0	0
22279	1667020	narV, yddE	0.906	0.815	0	0
4797	1667205	narV, yddE	0.812	0.725	0	0
12004	1667421	yddE	1.117	0.954	0	0
15379	1667589	yddE	1.251	0.833	0	0
16486	1667924	yddE	1.025	0.938	0	0
14030	1668013	yddE	1.321	0.979	0	0
14475	1668438	nhoA	1.059	0.940	0	0
18896	1668513	nhoA	0.945	0.769	0	0
3800	1668693	nhoA	0.920	0.729	0	0
15256	1669058	nhoA	1.025	0.740	0	0
15908	1669202	nhoA	1.422	0.966	0	0
19979	1669283	nhoA	1.603	0.884	0	0
12355	1669783	STM1583	1.362	0.773	0	0
21597	1669928	STM1583	1.281	0.692	0	0
4778	1670064	STM1583	1.380	0.642	0	0
11720	1670432	STM1583, ansP	1.050	0.758	0	0
741	1670596	ansP	1.526	0.742	0	0
19316	1670710	ansP	0.944	0.687	0	0
20852	1670968	ansP	0.804	0.506	0	0
447	1671192	ansP	1.023	0.786	0	0
9467	1671416	ansP	1.064	0.689	0	0
18249	1671745	ansP	0.852	0.872	0	0
8228	1671938	ansP	1.186	0.844	0	0
2964	1672286	STM1585	1.037	0.695	0	0
20049	1672457	STM1585	1.239	0.827	0	0
2120	1672693	STM1585, STM1586	1.197	1.027	0	0
3076	1672851	STM1585, STM1586	1.023	0.771	0	0
5584	1672930	STM1586	1.100	0.858	0	0

12701	1673078	STM1586	0.888	0.801	0	0
19716	1673636	STM1586, yncD	1.348	0.805	0	0
13513	1673841	STM1586, yncD	1.176	0.646	0	0
7337	1673912	STM1586, yncD	0.982	0.651	0	0
6419	1674272	yncD	1.413	0.792	0	0
13432	1674588	yncD	1.060	0.762	0	0
20315	1674725	yncD	1.167	0.903	0	0
13002	1674828	yncD	0.820	0.853	0	0
19897	1675099	yncD	1.305	1.010	0	0
21231	1675310	yncD	1.028	0.747	0	0
1891	1675490	yncD	1.412	0.635	0	0
17250	1675822	yncD	3.599	0.659	1	0
17229	1675984	yncD	2.708	0.573	1	0
15471	1676154	yncC	1.455	0.462	0	0
2838	1676477	yncC	1.638	0.656	0	0
8267	1676698	yncC	1.384	0.558	0	0
5016	1676918	yncC	2.197	0.706	0	0
9232	1677175	yncC, yncB	0.982	0.553	0	0
19422	1677267	yncB	1.948	0.802	0	0
4142	1677737	yncB	1.133	0.855	0	0
11099	1677809	yncB	0.924	0.870	0	0
3061	1678159	yncB, yncA	1.407	0.915	0	0
15698	1678307	yncB, yncA	0.960	0.864	0	0
22245	1678382	yncB, yncA	1.307	1.118	0	0
680	1678902	ydcZ	1.058	1.517	0	0
18879	1678973	ydcZ	0.694	0.964	0	0
19444	1679077	ydcZ	0.705	1.062	0	0
8100	1679525	ydcY, srfA	0.879	1.525	0	0
17187	1679723	ydcY, srfA	1.342	2.105	0	1
19924	1679913	ydcY, srfA	1.015	1.679	0	1
6830	1680144	srfA	0.991	1.485	0	0
8068	1680424	srfA	0.992	1.778	0	0
4275	1680686	srfA, srfB	0.887	1.309	0	0
1892	1680791	srfA, srfB	1.059	1.818	0	0
14536	1681059	srfA, srfB	0.830	1.282	0	0
5227	1681335	srfB	0.625	1.280	0	0

13755	1681470	srfB	0.287	0.580	0	0
9892	1681713	srfB	0.922	1.503	0	0
9050	1681933	srfB	0.968	1.880	0	0
6655	1682008	srfB	0.823	1.606	0	0
22023	1682291	srfB	1.036	1.903	0	0
3067	1682580	srfB	1.116	1.176	0	0
10942	1682888	srfB	1.129	1.726	0	0
5849	1683218	srfB	0.799	1.355	0	0
7986	1683448	srfB	0.745	0.796	0	0
7496	1683668	srfB, srfC	1.078	1.723	0	1
20962	1683891	srfB, srfC	1.104	1.778	0	1
471	1684037	srfB, srfC	1.140	1.168	0	0
13772	1684265	srfC	1.068	1.898	0	1
16071	1684505	srfC	0.776	1.755	0	1
21368	1684640	srfC	0.289	0.788	0	0
13440	1684898	srfC	0.859	1.565	0	0
11726	1685001	srfC	0.986	1.872	0	0
5989	1685377	srfC	0.860	1.723	0	0
6198	1685627	srfC	0.725	1.484	0	0
11999	1685839	srfC	0.836	1.511	0	0
12424	1686099	srfC	0.782	1.469	0	0
11984	1686327	ydcX	0.923	1.879	0	0
1260	1686483	ydcX	0.703	1.995	0	0
5072	1686819	ydcX, ydcW	0.556	1.185	0	0
14868	1686931	ydcX, ydcW	0.772	1.662	0	0
16157	1687052	ydcW	0.673	1.151	0	0
17951	1687178	ydcW	0.961	1.385	0	0
19294	1687591	ydcW	0.708	0.699	0	0
9372	1687923	ydcW	1.313	1.023	0	0
1070	1688152	ydcW	1.009	0.851	0	0
3194	1688232	ydcW	1.061	0.820	0	0
21790	1688598	ydcW, ydcR	0.999	0.960	0	0
1905	1688751	ydcR	0.951	0.707	0	0
21613	1688857	ydcR	1.010	0.871	0	0
8692	1689030	ydcR	0.691	0.593	0	0
11596	1689523	ydcR	1.092	0.769	0	0

15422	1689647	ydcR	1.027	0.766	0	0
18566	1689889	ydcR	0.568	0.444	0	0
4586	1690093	ydcR, pdgL	1.071	0.772	0	0
4446	1690339	pdgL	1.162	0.677	0	0
21639	1690674	pdgL	0.832	0.554	0	0
18934	1690821	pdgL	1.911	0.752	0	0
6643	1691130	pdgL, STM1600	2.248	0.685	0	0
19811	1691259	STM1600, ugtL	2.344	0.630	0	0
3835	1691540	STM1600, ugtL	2.269	0.497	0	0
2504	1691639	STM1600, ugtL	3.110	0.611	0	0
2919	1692004	ugtL, sifB	2.303	0.499	0	0
1926	1692086	sifB	2.343	0.479	0	0
5527	1692295	sifB	2.356	0.523	0	0
2859	1692542	sifB	2.557	0.448	1	0
15832	1692986	sifB, yncJ	3.262	0.442	1	0
9893	1693079	sifB, yncJ	3.798	0.548	1	0
16096	1693371	yncJ	2.561	0.485	1	0
20147	1693584	yncJ	0.821	0.586	0	0
10473	1693823	ydcP	1.514	0.784	0	0
11094	1693967	ydcP	1.253	0.761	0	0
14298	1694223	ydcP	0.824	0.744	0	0
21568	1694398	ydcP	0.977	0.969	0	0
14927	1694810	ydcP	1.060	0.962	0	0
4045	1694913	ydcP	0.986	1.128	0	0
2423	1695115	ydcP	0.945	0.761	0	0
18810	1695186	ydcP	1.019	1.007	0	0
15206	1695662	ydcP, ydcN	1.064	1.261	0	0
11016	1695906	ydcP, ydcN	0.981	1.333	0	0
21066	1696062	ydcN, STM1606	0.828	1.621	0	0
14728	1696240	ydcN, STM1606	0.829	1.500	0	0
18611	1696456	ydcN, STM1606	0.920	1.258	0	0
12473	1696737	STM1606	0.797	0.803	0	0
18077	1697000	STM1606	0.583	0.689	0	0
17983	1697279	STM1606	1.343	1.314	0	0
10136	1697527	STM1607	1.012	1.259	0	0
11574	1697675	STM1607	0.846	1.267	0	0

1831	1697953	STM1607	0.710	0.764	0	0
8952	1698157	STM1607	0.716	0.927	0	0
21106	1698249	STM1607	0.911	1.015	0	0
5152	1698618	STM1607, tehB	1.386	1.028	0	0
9509	1698892	tehB	1.417	0.774	0	0
20011	1699026	tehB, tehA	1.983	0.834	0	0
21558	1699206	tehB, tehA	1.329	0.794	0	0
7943	1699489	tehA	0.720	0.714	0	0
9279	1699694	tehA, ydcK	0.940	0.911	0	0
3869	1699914	tehA, ydcK	0.881	0.813	0	0
3066	1700053	tehA, ydcK	1.153	0.717	0	0
21105	1700124	tehA, ydcK	1.426	0.843	0	0
19364	1700315	tehA, ydcK	1.160	0.724	0	0
20525	1700760	ydcK	0.996	0.737	0	0
15828	1701065	rimL	1.015	0.906	0	0
7186	1701239	rimL	1.248	0.781	0	0
16239	1701562	rimL, STM1612	1.397	0.809	0	0
8663	1701716	rimL, STM1612	1.216	0.720	0	0
16784	1701979	rimL, STM1612	1.129	0.672	0	0
7708	1702124	STM1612	0.870	0.501	0	0
18075	1702225	STM1612	1.091	0.756	0	0
22075	1702709	STM1612, STM1613	1.133	0.839	0	0
18322	1702883	STM1612, STM1613	0.747	0.494	0	0
11032	1703170	STM1614	1.108	0.602	0	0
5882	1703246	STM1614	1.166	0.673	0	0
10877	1703514	STM1614	0.833	0.471	0	0
2716	1703798	STM1614	1.195	0.559	0	0
13998	1704117	STM1614, STM1615	1.693	0.684	0	0
14214	1704274	STM1614, STM1615	1.172	0.670	0	0
3513	1704477	STM1614, STM1615	1.616	0.561	0	0
308	1704689	STM1615	1.181	0.592	0	0
20411	1704769	STM1615	1.341	0.717	0	0
16413	1705019	STM1615, STM1616	1.228	0.851	0	0
9820	1705300	STM1615, STM1616	1.398	1.003	0	0
594	1705430	STM1616, STM1617	1.431	1.091	0	0
21364	1705577	STM1616, STM1617	0.679	0.632	0	0

4009	1706004	STM1617	1.897	0.844	0	0
9846	1706268	STM1617, STM1618	1.245	0.703	0	0
5712	1706605	STM1618	0.417	0.472	0	0
16353	1706728	STM1618	1.443	0.718	0	0
16359	1706915	STM1618, STM1619	1.303	0.943	0	0
14085	1707170	STM1618, STM1619	1.368	0.877	0	0
3508	1707322	STM1618, STM1619	1.095	0.643	0	0
20601	1707748	STM1619	2.759	0.606	0	0
19854	1707843	STM1620	2.051	0.586	0	0
1513	1708076	STM1620	2.904	0.696	0	0
7771	1708315	STM1620	2.554	0.617	0	0
8895	1708389	STM1620	3.201	0.627	0	0
3882	1708536	STM1620	2.386	0.610	0	0
1502	1709050	STM1620, STM1621	2.163	0.546	0	0
15151	1709327	STM1620, STM1621	2.852	0.666	0	0
7001	1709563	STM1621	1.314	0.542	0	0
22280	1709684	STM1621	2.349	0.954	0	0
8553	1709937	STM1621, ydcG	1.723	1.291	0	0
8020	1710000	STM1621, ydcG	1.224	1.106	0	0
4901	1710300	ydcG	0.769	0.868	0	0
11640	1710665	ydcG	1.512	1.555	0	0
1082	1710823	ydcG	1.182	1.208	0	0
1243	1711139	ydcG	1.009	1.406	0	0
22101	1711322	ydcG	1.007	1.455	0	0
14421	1711461	ydcG	1.160	1.317	0	0
3609	1711534	ydcG	0.994	1.025	0	0
10977	1711674	ydcG, STM1623	1.091	0.965	0	0
5041	1711980	STM1623	1.039	0.891	0	0
17410	1712094	STM1623	1.141	1.047	0	0
12343	1712527	STM1623	1.293	0.939	0	0
21620	1712697	STM1623	1.454	1.419	0	0
9504	1713192	STM1623	1.077	0.990	0	0
6011	1713279	STM1623, STM1624	1.013	1.066	0	0
13674	1713632	STM1623, STM1624	0.955	0.891	0	0
20802	1713815	STM1624	0.815	0.888	0	0
15431	1713925	STM1624	1.225	1.179	0	0

11969	1714297	STM1624	0.977	1.312	0	0
2976	1714594	STM1624, ydcI	0.864	1.335	0	0
20453	1714671	STM1624, ydcI	0.948	1.532	0	0
7025	1714861	STM1624, ydcI	0.677	1.117	0	0
1278	1715095	ydcI	0.986	2.140	0	1
9080	1715362	ydcI	0.735	2.018	0	1
19347	1715583	ydcI	0.657	1.924	0	1
14649	1715874	trg	0.692	2.265	0	1
4983	1716013	trg	0.604	1.782	0	0
20223	1716373	trg	1.051	1.834	0	0
12022	1716562	trg	0.919	1.189	0	0
8856	1716832	trg	0.822	1.214	0	0
20458	1716989	trg	0.705	0.964	0	0
7655	1717278	trg	0.843	1.396	0	0
21185	1717479	trg	0.441	0.678	0	0
20620	1717674	trg, STM1627	1.047	1.155	0	0
17910	1717765	trg, STM1627	1.232	1.291	0	0
704	1718025	STM1627	1.292	0.819	0	0
10957	1718337	STM1627	1.268	0.842	0	0
9758	1718655	STM1627	1.334	0.781	0	0
17337	1718799	STM1627, STM1628	1.565	0.857	0	0
6156	1719054	STM1627, STM1628	2.049	0.589	0	0
2356	1719223	STM1627, STM1628	1.850	0.533	0	0
1754	1719493	STM1628, STM1629	1.810	0.490	0	0
15308	1719651	STM1629	2.704	0.463	0	0
765	1719811	STM1629	2.060	0.536	0	0
8088	1720116		2.732	0.545	1	0
20230	1720378	STM1630	2.302	0.484	1	0
1157	1720520	STM1630	2.699	0.535	1	0
8728	1720875	STM1630, sseJ	2.347	0.505	0	0
19049	1720947	STM1630, sseJ	2.396	0.510	0	0
4219	1721296	STM1630, sseJ	2.488	0.503	0	0
6275	1721607	sseJ	2.136	0.429	0	0
18483	1721839	sseJ	2.428	0.536	1	0
13984	1721919	sseJ	2.484	0.553	1	0
12138	1722122	sseJ	2.233	0.507	0	0

15995	1722440	sseJ	1.834	0.567	0	0
660	1722606		1.669	0.684	0	0
14667	1722713	STM1632	1.487	0.598	0	0
21772	1723188	STM1632	2.045	0.430	0	0
18652	1723343	STM1632	2.019	0.414	0	0
1674	1723475	STM1633	2.091	0.382	0	0
7579	1723896	STM1633	1.789	0.542	0	0
5293	1724138	STM1633	1.503	0.439	0	0
16948	1724302	STM1633, STM1634	2.203	0.503	0	0
11186	1724395	STM1633, STM1634	2.129	0.424	0	0
17001	1724792	STM1634	2.815	0.563	1	0
10640	1724869	STM1634, STM1635	3.243	0.534	1	0
2920	1725032	STM1634, STM1635	3.013	0.473	1	0
8516	1725271	STM1634, STM1635	3.071	0.485	1	0
15436	1725545	STM1635	2.105	0.488	0	0
1332	1725861	STM1635, STM1636	2.045	0.501	0	0
8299	1726087	STM1636	2.231	0.452	0	0
20751	1726215	STM1636	2.663	0.561	0	0
13858	1726398	STM1636, STM1637	2.306	0.502	0	0
8610	1726811	STM1637	3.041	0.425	0	0
21924	1726910	STM1637	2.332	0.597	0	0
278	1727079	STM1637	2.128	0.663	0	0
17400	1727408	STM1637	1.980	0.624	0	0
20961	1727732	STM1637	2.155	0.571	0	0
14953	1727807	STM1637	2.157	0.580	0	0
12397	1728210	STM1637	3.011	0.454	1	0
10160	1728331	STM1637	2.849	0.536	1	0
18653	1728410		3.000	0.517	1	0
12584	1728898	STM1638	3.320	0.672	1	0
18314	1728970	STM1638	2.774	0.672	1	0
7772	1729230	STM1638	1.778	0.885	0	0
11113	1729349	STM1638	1.268	0.842	0	0
12490	1729730		0.954	0.954	0	0
6141	1729853	cybB	1.187	1.082	0	0
12756	1730181	cybB	1.480	1.044	0	0
18463	1730401	cybB	1.197	1.023	0	0

13185	1730552	cybB, ydcF	1.035	0.979	0	0
18776	1730893	ydcF	1.075	0.838	0	0
10818	1731049	ydcF	1.227	0.863	0	0
22120	1731258	ydcF	1.238	1.034	0	0
18507	1731410	ydcF, hrpA	1.112	1.069	0	0
21126	1731725	hrpA	1.073	0.926	0	0
17884	1731948	hrpA	0.898	0.686	0	0
233	1732278	hrpA	0.942	0.916	0	0
19595	1732478	hrpA	1.426	1.003	0	0
9466	1732637	hrpA	1.362	0.874	0	0
11861	1732772	hrpA	1.130	0.754	0	0
3740	1732886	hrpA	1.345	0.908	0	0
1952	1733391	hrpA	1.060	0.889	0	0
7348	1733540	hrpA	1.663	0.973	0	0
12890	1733648	hrpA	1.002	0.893	0	0
10575	1733964	hrpA	1.511	1.094	0	0
16834	1734346	hrpA	1.149	1.054	0	0
16746	1734594	hrpA	1.241	0.992	0	0
21957	1734711	hrpA	1.027	0.963	0	0
374	1734848	hrpA	0.933	0.820	0	0
9516	1735074	hrpA, acpD	0.907	0.968	0	0
21121	1735289	hrpA, acpD	1.121	1.032	0	0
10099	1735406	hrpA, acpD	0.841	0.944	0	0
22129	1735716	acpD	0.979	1.326	0	0
14494	1735868	acpD	0.824	0.985	0	0
21513	1736193	STM1643	1.347	0.902	0	0
17553	1736514	STM1643	1.358	0.942	0	0
11515	1736780	STM1643, ydbL	1.382	0.894	0	0
15139	1737008	ydbL, ynbE	1.163	0.923	0	0
12177	1737123	ydbL, ynbE	0.876	0.675	0	0
3761	1737360	ydbL, ynbE	1.252	0.837	0	0
15603	1737497	ynbE, ydbH	1.065	0.856	0	0
12959	1737590	ynbE, ydbH	1.295	0.839	0	0
22278	1737702	ydbH	0.606	0.575	0	0
19888	1738355	ydbH	1.228	1.094	0	0
939	1738529	ydbH	1.432	1.154	0	0

17055	1738757	ydbH	1.297	1.105	0	0
4201	1738932	ydbH	1.190	1.118	0	0
11285	1739278	ydbH	1.017	1.744	0	0
22016	1739564	ydbH, ldhA	1.089	1.815	0	0
7427	1739721	ydbH, ldhA	0.946	1.244	0	0
20737	1739826	ydbH, ldhA	0.935	1.212	0	0
5450	1740050	ydbH, ldhA	0.969	0.992	0	0
2973	1740144	ydbH, ldhA	0.941	1.105	0	0
960	1740670	ldhA, hslJ	0.992	1.128	0	0
5884	1740790	ldhA, hslJ	0.635	0.841	0	0
22319	1741104	hslJ	0.790	1.608	0	0
13240	1741351	hslJ, STM1650	0.787	2.049	0	1
7686	1741510	STM1650	0.908	2.067	0	1
19896	1741839	STM1649, STM1650	0.798	1.298	0	0
7559	1742007	STM1649, STM1650	1.119	0.982	0	0
16442	1742078	STM1649, nifJ	1.145	1.044	0	0
923	1742349	nifJ	0.546	0.426	0	0
1564	1742639	nifJ	1.161	0.689	0	0
10238	1742784	nifJ	1.179	0.730	0	0
15414	1743005	nifJ	0.549	0.625	0	0
11817	1743447	nifJ	2.117	0.898	0	0
15571	1743579	nifJ	1.910	0.802	0	0
16265	1743870	nifJ	1.359	0.618	0	0
14587	1744139	nifJ	1.565	0.722	0	0
5632	1744362	nifJ	1.494	0.652	0	0
2986	1744441	nifJ	1.967	0.980	0	0
4404	1744596	nifJ	1.927	0.787	0	0
694	1744739	nifJ	2.030	1.190	0	0
13981	1745176	nifJ	1.402	0.652	0	0
6042	1745459	nifJ	1.314	0.494	0	0
5280	1745638	ynaF	3.294	0.662	1	0
6919	1745913	ynaF	2.872	0.643	1	0
17044	1746083	ynaF	2.318	0.900	0	0
12106	1746167	ynaF	1.915	0.966	0	0
4003	1746392	ynaF	1.198	1.250	0	0
2009	1746695	STM1653	1.094	1.092	0	0

6225	1746998	STM1653, ydaO	1.034	1.065	0	0
15531	1747185	STM1653, ydaO	1.098	1.182	0	0
13725	1747310	STM1653, ydaO	0.926	0.858	0	0
9069	1747609	ydaO	1.217	0.997	0	0
11938	1747881	ydaO	0.785	0.875	0	0
2545	1748067	ydaO	0.909	1.247	0	0
10794	1748277	dbpA	1.007	1.177	0	0
19852	1748459	dbpA	1.360	1.138	0	0
17377	1748865	dbpA	0.846	0.713	0	0
13797	1748952	dbpA	1.372	0.792	0	0
20229	1749201	dbpA	1.322	0.797	0	0
14568	1749433	dbpA	1.090	0.691	0	0
2927	1749826	dbpA	1.418	0.872	0	0
8462	1749897	dbpA	1.237	0.822	0	0
9234	1750174	STM1656	0.880	0.827	0	0
8282	1750270	STM1656	1.254	0.874	0	0
2324	1750581	STM1656	0.772	0.631	0	0
7930	1750771	STM1656	1.032	1.052	0	0
576	1750997	STM1656	0.974	1.148	0	0
14686	1751150	STM1656	1.166	1.237	0	0
8197	1751353	STM1656, STM1657	1.112	0.901	0	0
22297	1751803	STM1657	1.463	0.859	0	0
8722	1752069	STM1657	1.959	0.820	0	0
14020	1752250	STM1657	2.661	0.656	0	0
15368	1752500	STM1657	2.016	0.698	0	0
3243	1752738	STM1657	1.613	0.801	0	0
962	1752951	ydaL	1.204	0.913	0	0
15387	1753212	ydaL, ogt	1.020	0.848	0	0
16904	1753446	ydaL, ogt	1.186	0.932	0	0
9189	1753636	ydaL, ogt	1.191	0.858	0	0
6810	1753767	ydaL, ogt	1.016	1.050	0	0
17931	1754129	ogt, fnr	1.027	1.333	0	0
10673	1754288	fnr	0.916	1.541	0	0
7587	1754528	fnr	0.716	1.831	0	1
7127	1754599	fnr	0.599	1.671	0	1
22001	1754793	fnr, ydaA	0.733	1.867	0	1

6814	1755226	ydaA	0.662	1.486	0	0
18146	1755380	ydaA	0.684	1.506	0	0
3700	1755485	ydaA	0.846	1.825	0	0
12948	1755765	ydaA	0.771	1.545	0	0
15771	1755981	ydaA	0.881	1.715	0	0
18033	1756362	ynaJ, ynaI	1.486	1.525	0	0
15446	1756521	ynaJ, ynaI	1.977	0.965	0	0
2602	1756723	ynaJ, ynaI	2.489	0.895	0	0
13722	1757067	ynaI	2.456	0.528	0	0
8908	1757287	ynaI	2.620	0.664	0	0
12894	1757528	ynaI	1.821	0.777	0	0
18758	1757622	ynaI	1.678	0.685	0	0
6143	1757988	STM1664	1.242	0.766	0	0
16356	1758079	STM1664, STM1665	1.335	0.934	0	0
3653	1758375	STM1664, STM1665	1.268	0.753	0	0
1080	1758511	STM1664, STM1665	0.867	0.774	0	0
3112	1758899	STM1664, STM1665	1.444	0.613	0	0
18479	1759079	STM1665	2.276	0.656	1	0
3557	1759272		2.597	0.633	1	0
11262	1759472		3.343	0.771	1	0
4262	1759668	STM1667	2.305	0.696	0	0
21246	1759749	STM1667	3.063	0.738	1	0
14542	1760144	STM1667	2.916	0.480	1	0
22355	1760352	STM1667, STM1668	2.885	0.500	1	0
11115	1760652	STM1667, STM1668	3.131	0.440	1	0
12849	1760764	STM1668	3.468	0.400	1	0
19285	1760975	STM1668	3.049	0.516	1	0
6125	1761345	STM1668, STM1669	3.263	0.716	1	0
14560	1761550	STM1668, STM1669	1.624	0.773	0	0
8907	1761829	STM1669	1.431	0.645	0	0
19140	1761929	STM1669	1.353	0.599	0	0
4680	1762280	STM1669	1.752	0.712	0	0
20630	1762450	STM1669	1.880	0.646	0	0
3081	1762612	STM1669	2.479	0.624	1	0
5086	1762856	STM1669	3.254	0.565	1	0
4556	1763077	STM1669	4.057	0.580	1	0

10779	1763205	STM1669	3.375	0.518	1	0
15504	1763540	STM1669, STM1670	2.763	0.551	1	0
9939	1763903	STM1670, STM1671	2.968	0.550	1	0
6943	1764037	STM1670, STM1671	2.855	0.506	1	0
20155	1764238	STM1670, STM1671	3.747	0.508	1	0
639	1764456	STM1670, STM1671	2.999	0.611	1	0
11247	1764744	STM1671	2.462	0.444	1	0
901	1764912	STM1671	2.274	0.620	0	0
3767	1765146	STM1671, STM1672	2.158	0.458	0	0
1173	1765236	STM1672	1.800	0.520	0	0
1277	1765539	STM1672	1.753	0.564	0	0
14052	1765725	STM1672	1.613	0.647	0	0
15616	1765966	STM1672	1.923	0.610	0	0
21496	1766163	STM1672	1.943	0.695	0	0
14656	1766427	STM1672	2.084	0.693	0	0
12521	1766814		1.553	0.826	0	0
19526	1766985	STM1673	1.321	0.856	0	0
14730	1767157	STM1673	1.161	0.929	0	0
18968	1767259	STM1673	1.073	0.889	0	0
9950	1767699	STM1674, STM1675	0.995	0.819	0	0
1236	1767985	STM1674, STM1675	0.728	0.566	0	0
13342	1768179	STM1674, STM1675	0.968	0.674	0	0
6583	1768277	STM1674, STM1675	1.183	0.841	0	0
5516	1768566	STM1675	1.024	0.743	0	0
7515	1768876	STM1676	1.340	0.812	0	0
11106	1769029	STM1676	0.938	0.652	0	0
7182	1769166	STM1676	1.668	0.704	0	0
1611	1769389	STM1676	1.723	0.603	0	0
5050	1769570	STM1676	1.117	0.516	0	0
18873	1769837	STM1676	2.397	0.608	0	0
1342	1770131	STM1677	2.062	0.817	0	0
13043	1770424	STM1677	1.239	0.706	0	0
7247	1770689	STM1677, STM1678	1.315	0.800	0	0
503	1770906	STM1677, STM1678	1.109	0.744	0	0
14023	1771103	STM1677, STM1678	1.044	0.832	0	0
1938	1771193	STM1677, STM1678	1.021	1.085	0	0

7541	1771423	STM1678	0.727	0.817	0	0
17807	1771586	STM1678	0.392	0.549	0	0
1280	1771973	mppA	1.277	0.990	0	0
17691	1772200	mppA	1.714	1.101	0	0
13703	1772419	mppA	1.438	0.891	0	0
4937	1772509	mppA	1.708	0.868	0	0
13486	1772855	mppA	1.763	0.796	0	0
2248	1773023	mppA	1.457	0.867	0	0
21162	1773373	mppA, ycjI	0.831	1.032	0	0
20168	1773577	mppA, ycjI	0.726	0.775	0	0
6761	1773745	mppA, ycjI	0.979	1.217	0	0
6579	1774033	mppA, ycjI	0.843	1.234	0	0
842	1774219	ycjI	0.743	1.276	0	0
1787	1774459	ycjI	1.066	1.301	0	0
14729	1774768	ycjG	0.965	0.945	0	0
6318	1774841	ycjG	1.022	1.032	0	0
13544	1775228	ycjG, tpx	1.045	1.358	0	0
17088	1775427	ycjG, tpx	0.920	1.527	0	0
18981	1775662	ycjG, tpx	1.061	1.556	0	0
9074	1775776	ycjG, tpx	0.843	1.744	0	0
10648	1775892	ycjG, tpx	1.069	1.830	0	0
17213	1776280	tyrR	0.502	0.576	0	0
13871	1776609	tyrR	1.247	1.045	0	0
3850	1776689	tyrR	0.675	0.720	0	0
20283	1776821	tyrR	0.368	0.588	0	0
13576	1777208	tyrR	1.073	1.201	0	0
5656	1777382	tyrR	1.030	1.149	0	0
18624	1777570	tyrR	1.231	1.159	0	0
3370	1777691	tyrR	1.238	1.281	0	0
19674	1778170	ycjF	1.171	0.987	0	0
8041	1778361	ycjF	1.052	0.907	0	0
15548	1778623	ycjF	0.531	0.749	0	0
12222	1778900	ycjF, ycjX	1.236	1.139	0	0
1668	1778974	ycjF, ycjX	1.015	1.013	0	0
15541	1779109	ycjF, ycjX	1.271	1.202	0	0
11816	1779473	ycjX	1.081	1.126	0	0

22066	1779681	ycjX	1.373	1.017	0	0
2810	1779901	ycjX	0.892	0.858	0	0
1008	1780056	ycjX	1.572	1.092	0	0
10749	1780294	ycjX	1.684	1.213	0	0
19318	1780599	ycjX, pspE	1.426	0.879	0	0
21419	1780776	pspE	1.321	0.808	0	0
19299	1781075	pspE, pspD	1.184	0.898	0	0
10350	1781255	pspD, pspC	1.135	0.789	0	0
8510	1781432	pspD, pspC	0.836	0.683	0	0
14633	1781607	pspC, pspB	0.380	0.572	0	0
4540	1781854	pspC, pspB	1.175	0.841	0	0
16032	1782090	pspB, pspA	0.936	0.893	0	0
3923	1782381	pspA, pspF	0.987	1.067	0	0
2981	1782554	pspA, pspF	0.987	1.082	0	0
19911	1782989	pspF	1.015	1.231	0	0
1239	1783185	pspF, sapA	0.979	1.267	0	0
13016	1783295	pspF, sapA	0.858	1.117	0	0
16625	1783550	sapA	0.932	1.517	0	0
17725	1783869	sapA	0.816	1.472	0	0
1162	1784120	sapA	0.893	1.591	0	0
6720	1784208	sapA	0.457	0.797	0	0
15807	1784564	sapA	0.729	0.972	0	0
20774	1784703	sapA	1.208	1.372	0	0
13709	1784927	sapA, sapB	1.206	1.468	0	0
1761	1785201	sapA, sapB	0.659	1.036	0	0
3052	1785353	sapB	0.719	1.212	0	0
2264	1785667	sapB	0.658	0.950	0	0
11996	1785911	sapB, sapC	1.084	1.085	0	0
13686	1786045	sapB, sapC	1.062	1.324	0	0
20524	1786238	sapB, sapC	1.014	1.189	0	0
4842	1786428	sapC	0.324	0.549	0	0
16269	1786789	sapC, sapD	1.193	1.128	0	0
16978	1786876	sapC, sapD	1.325	1.280	0	0
12919	1787159	sapD	1.416	1.257	0	0
2917	1787333	sapD	0.990	1.001	0	0
7705	1787617	sapD	0.871	0.955	0	0

19617	1787767	sapD, sapF	0.941	0.981	0	0
10721	1788115	sapD, sapF	0.747	0.811	0	0
6201	1788444	sapF	0.809	1.161	0	0
9125	1788539	sapF	0.824	1.004	0	0
13567	1788794	sapF	0.781	1.002	0	0
6259	1789010		1.009	1.200	0	0
19557	1789341	STM1697	1.327	0.969	0	0
14467	1789448	STM1697	1.483	0.996	0	0
18356	1789665	STM1697	1.403	0.742	0	0
4521	1789913	STM1697	1.690	0.548	0	0
19204	1790062	STM1697	2.140	0.583	0	0
19583	1790238	STM1698	2.679	0.593	1	0
8218	1790659	STM1698	2.896	0.433	1	0
15192	1790741	STM1698	2.997	0.527	1	0
10229	1791108	STM1698, STM1698A	2.749	0.559	1	0
3195	1791265	STM1698, STM1698A	2.788	0.604	0	0
4840	1791489	STM1698, STM1698A	2.770	0.443	0	0
8470	1791739	STM1698, ycjE	2.478	0.558	0	0
6246	1792012	fabI	0.912	0.496	0	0
10240	1792084	fabI	1.533	0.826	0	0
8485	1792440	fabI	1.032	1.406	0	0
8824	1792759	fabI, yciW	0.916	0.956	0	0
14316	1792962	yciW	0.664	0.828	0	0
15740	1793135	yciW	1.147	1.054	0	0
21938	1793206	yciW	1.170	0.904	0	0
6972	1793593	yciW	1.225	0.963	0	0
14976	1793905	yciW, rnb	0.386	0.604	0	0
20535	1794138	rnb	0.827	1.306	0	0
557	1794223	rnb	1.063	1.748	0	0
17105	1794300	rnb	1.066	1.723	0	0
21342	1794780	rnb	0.696	1.215	0	0
2848	1794938	rnb	1.046	1.553	0	0
10708	1795025	rnb	0.965	1.583	0	0
19740	1795347	rnb	1.023	1.766	0	0
307	1795560	rnb	1.116	1.856	0	0
21234	1795793	rnb	0.927	1.548	0	0

5282	1796147	rnb, yciR	1.127	0.924	0	0
5290	1796321	yciR	1.002	0.912	0	0
10234	1796452	yciR	1.146	0.942	0	0
12715	1796691	yciR	1.310	0.809	0	0
17222	1796940	yciR	1.370	0.760	0	0
13025	1797048	yciR	1.474	0.770	0	0
3629	1797326	yciR	1.083	0.571	0	0
14981	1797720	yciR	1.281	0.755	0	0
7789	1797793	yciR	1.239	0.718	0	0
11129	1798069	yciR	1.173	0.679	0	0
11777	1798252	yciR	1.335	0.734	0	0
14632	1798614	yciT	1.144	0.784	0	0
11548	1798863	yciT	0.410	0.495	0	0
22072	1798992	yciT	1.118	1.041	0	0
11340	1799283	yciT	1.297	1.044	0	0
3264	1799356	yciT	1.069	0.922	0	0
8859	1799697	osmB	2.439	0.823	0	0
11366	1799825	osmB	2.397	0.790	0	0
4178	1800163		2.435	0.851	0	0
9135	1800395	yciH	2.143	0.893	0	0
11342	1800557	yciH, pyrF	1.363	0.864	0	0
4598	1800899	yciH, pyrF	0.853	0.975	0	0
8932	1801097	pyrF	0.792	1.005	0	0
5655	1801286	pyrF	0.811	1.299	0	0
8888	1801502	pyrF, yciM	0.749	1.395	0	0
5903	1801769	yciM	0.757	1.402	0	0
4240	1801940	yciM	0.674	0.996	0	0
16723	1802110	yciM	0.736	1.504	0	0
4297	1802483	yciM	0.594	1.368	0	0
2697	1802652	yciM	0.549	1.270	0	0
13667	1802964	yciM, yciS	0.773	2.080	0	1
19359	1803102	yciM, yciS	0.857	2.104	0	1
4428	1803411	yciS, pgpB	0.685	1.166	0	0
7523	1803540	pgpB	0.940	1.197	0	0
8400	1803762	pgpB, ribA	0.843	1.047	0	0
3770	1803941	pgpB, ribA	0.890	1.069	0	0

5030	1804080	pgpB, ribA	1.062	1.284	0	0
16679	1804551	ribA	1.290	1.387	0	0
4456	1804762	acnA	0.828	1.059	0	0
20537	1804907	acnA	0.835	1.590	0	0
1086	1805149	acnA	0.610	0.905	0	0
20026	1805237	acnA	1.168	1.379	0	0
17300	1805550	acnA	0.877	0.894	0	0
2259	1805916	acnA	0.997	0.837	0	0
11939	1806027	acnA	0.721	0.783	0	0
10404	1806157	acnA	1.034	1.055	0	0
19217	1806401	acnA	1.263	1.033	0	0
9548	1806554	acnA	0.772	0.797	0	0
9132	1806995	acnA	0.832	0.808	0	0
10916	1807238	acnA	0.872	0.853	0	0
11638	1807518	acnA	1.032	1.135	0	0
8649	1807630	acnA	0.979	1.340	0	0
19543	1807750	acnA	0.959	1.619	0	0
21413	1808089		0.868	1.707	0	0
22102	1808378	cysB	0.643	0.958	0	0
7357	1808525	cysB	0.684	0.897	0	0
20766	1808864	cysB	0.575	0.800	0	0
4145	1809136	cysB	0.845	1.076	0	0
15950	1809312	cysB	1.123	1.009	0	0
4000	1809496	cysB	1.229	0.876	0	0
12462	1809699	cysB	0.744	0.592	0	0
12051	1809869	topA	1.017	0.881	0	0
19638	1810157	topA	0.917	0.980	0	0
21581	1810326	topA	0.518	0.888	0	0
15894	1810644	topA	1.047	1.427	0	0
4464	1810884	topA	0.428	0.692	0	0
15499	1811143	topA	0.837	1.189	0	0
14033	1811349	topA	0.702	1.532	0	0
14594	1811559	topA	0.723	1.300	0	0
2916	1811707	topA	0.852	1.706	0	0
5081	1811930	topA	0.840	1.349	0	0
17164	1812288	topA	0.517	1.181	0	0

6323	1812366	topA, yciN	0.603	1.390	0	0
3371	1812701	topA, yciN	0.533	1.220	0	0
17399	1812811	topA, yciN	0.636	1.485	0	0
20740	1812988	yciN	0.534	1.142	0	0
8467	1813345	sohB	0.727	1.369	0	0
7758	1813457	sohB	0.683	0.797	0	0
10411	1813767	sohB	0.858	1.177	0	0
14997	1814078	sohB, yciK	0.822	1.092	0	0
11337	1814182	sohB, yciK	0.791	1.047	0	0
2254	1814357	sohB, yciK	0.749	1.272	0	0
22199	1814687	yciK, btuR	0.993	1.670	0	0
8511	1814770	yciK, btuR	0.868	1.370	0	0
21030	1815192	btuR	0.622	0.888	0	0
20566	1815370	btuR	1.026	1.734	0	0
16794	1815478	btuR	0.834	1.693	0	1
12880	1815759		0.775	1.745	0	1
10927	1815875	yciL	0.794	2.096	0	1
14906	1816304	yciL	0.833	1.884	0	1
16030	1816495	yciL	0.920	2.064	0	1
7926	1816837	yciL, yciO	0.699	2.010	0	1
1735	1816912	yciL, yciO	0.758	1.956	0	1
4977	1817259	yciO	1.415	1.353	0	0
4136	1817357	yciO	0.938	1.069	0	0
4523	1817689	yciO, trpH	0.536	0.608	0	0
13830	1817989	trpH, trpL	0.936	1.113	0	0
846	1818206	trpH, trpL	0.958	0.917	0	0
2755	1818344	trpH, trpL	1.291	0.889	0	0
2764	1818526	trpH, trpE	0.513	0.495	0	0
5477	1818660	trpH, trpE	0.728	0.506	0	0
11430	1819085	trpE	1.419	0.761	0	0
20465	1819355	trpE	0.350	0.536	0	0
21082	1819586	trpE	1.113	0.578	0	0
3145	1819691	trpE, trpD	1.358	0.693	0	0
21155	1819929	trpE, trpD	1.279	1.134	0	0
10095	1820132	trpD	1.576	0.848	0	0
17267	1820314	trpD	0.500	0.530	0	0

21756	1820680	trpD	0.538	0.645	0	0
1970	1820918	trpD	1.455	0.633	0	0
10929	1821081	trpD	1.357	0.543	0	0
4236	1821198	trpD	1.002	0.583	0	0
18912	1821425	trpD, trpC	1.205	0.731	0	0
19689	1821702	trpC	1.571	0.759	0	0
21844	1822034	trpC	1.613	0.929	0	0
5549	1822106	trpC	0.809	0.605	0	0
17559	1822336	trpC	1.350	0.690	0	0
5258	1822636	trpC, trpB	0.945	0.565	0	0
18913	1822791	trpC, trpB	0.893	0.628	0	0
19531	1823057	trpB	1.003	0.782	0	0
22172	1823258	trpB	1.255	0.910	0	0
19211	1823678	trpB	1.479	0.798	0	0
2538	1823805	trpB, trpA	1.024	0.715	0	0
16782	1823920	trpB, trpA	1.369	0.799	0	0
358	1824216	trpB, trpA	1.464	0.952	0	0
14694	1824557	trpA	1.180	0.755	0	0
10319	1824825	trpA	1.710	0.879	0	0
5347	1824896	trpA	0.855	0.608	0	0
5311	1825125	yciG	2.019	0.549	0	0
7087	1825263	yciG	3.023	0.544	1	0
18633	1825514	yciG, yciF	4.282	0.454	1	0
4720	1825837	yciF	3.326	0.543	1	0
21614	1825954	yciF, yciE	2.852	0.473	1	0
19481	1826230	yciF, yciE	2.898	0.462	1	0
7911	1826524	yciE, STM1731	4.087	0.469	1	0
19249	1826662	yciE, STM1731	3.699	0.493	1	0
2584	1826918	STM1731	2.383	0.512	0	0
15793	1827118	STM1731	1.849	0.531	0	0
12871	1827415	STM1731	1.168	0.847	0	0
6449	1827592	STM1731	1.490	0.908	0	0
21935	1827793		1.047	0.858	0	0
1397	1827868	ompW	1.074	0.750	0	0
20781	1828331	ompW	1.295	0.748	0	0
11886	1828679	ompW, STM1733	1.794	0.715	0	0

5177	1828826	ompW, STM1733	1.935	0.755	0	0
16238	1829067	STM1733, yciC	1.498	0.967	0	0
19847	1829188	STM1733, yciC	0.638	0.552	0	0
12594	1829265	yciC	1.066	0.829	0	0
8683	1829702	yciC, yciB	0.909	1.092	0	0
13856	1829939	yciC, yciB	0.794	1.024	0	0
15287	1830071	yciB	1.043	1.251	0	0
2144	1830360	yciB, yciA	1.412	1.124	0	0
15568	1830595	yciB, yciA	0.921	1.190	0	0
18939	1830707	yciA	0.979	1.409	0	0
14163	1830786	yciA	1.139	1.394	0	0
16791	1831057	yciA	1.087	1.547	0	0
5671	1831394	tonB	0.807	1.314	0	0
3540	1831661	tonB	0.462	0.661	0	0
7940	1831874	tonB, yciI	0.754	1.071	0	0
1958	1832154	tonB, yciI	0.633	1.113	0	0
15285	1832475	yciI, cls	0.700	1.734	0	0
2287	1832577	cls	0.591	1.627	0	0
15526	1832782	cls	0.762	1.781	0	0
8266	1832858	cls	0.502	0.980	0	0
9465	1832977	cls	0.768	1.201	0	0
12869	1833542	cls	0.985	0.938	0	0
5784	1833870	cls, yciU	0.701	0.816	0	0
12925	1834125	cls, yciU	0.918	1.089	0	0
590	1834294	cls, yciU	0.943	1.351	0	0
19506	1834511	yciU	0.592	1.274	0	0
932	1834734	STM1741	0.677	1.677	0	1
19086	1834858	STM1741	0.836	1.899	0	1
331	1835054	STM1741	0.962	1.647	0	0
12025	1835372	STM1741	0.551	1.131	0	0
14288	1835553	STM1741	0.650	1.622	0	0
19004	1835856	STM1741, oppF	0.622	1.696	0	0
6049	1835963	STM1741, oppF	0.492	1.480	0	0
15087	1836219	oppF	0.669	2.249	0	1
19687	1836571	oppF	0.690	1.897	0	1
13492	1836738	oppF, oppD	0.694	2.022	0	1

22032	1836955	oppF, oppD	0.818	1.812	0	1
17205	1837200	oppD	0.667	1.872	0	1
18538	1837337	oppD	0.772	1.897	0	1
5540	1837678	oppD, oppC	0.616	1.661	0	0
4033	1837963	oppD, oppC	0.539	1.800	0	1
4633	1838085	oppC	0.721	1.900	0	1
3101	1838352	oppC	0.985	2.065	0	1
14559	1838554	oppC, oppB	0.750	1.697	0	1
14765	1838689	oppC, oppB	0.641	1.544	0	0
4853	1838918	oppC, oppB	0.743	1.604	0	0
315	1839291	oppB	0.727	1.777	0	0
7573	1839407	oppB	0.866	1.670	0	0
12060	1839579	oppB, oppA	0.696	1.283	0	0
978	1839845	oppB, oppA	0.648	1.542	0	0
14354	1840226	oppA	0.730	1.461	0	0
2820	1840366	oppA	0.605	1.458	0	0
5756	1840648	oppA	0.744	1.484	0	0
14426	1840919	STM1747, oppA	0.678	1.509	0	0
5541	1841064	STM1747, oppA	0.734	1.502	0	0
6935	1841328	STM1747, oppA	0.795	1.053	0	0
1383	1841471	STM1747, oppA	0.982	0.882	0	0
6528	1841731	oppA	1.241	0.754	0	0
14555	1841903		1.360	0.743	0	0
5596	1842000	ychE	1.318	0.688	0	0
17240	1842297	ychE	0.722	0.873	0	0
4070	1842717	ychE, adhE	0.616	0.601	0	0
7305	1842879	ychE, adhE	0.699	0.689	0	0
22081	1843110	adhE	0.753	0.863	0	0
19948	1843423	adhE	0.652	0.836	0	0
19499	1843615	adhE	0.786	0.975	0	0
13836	1843855	adhE	0.567	0.831	0	0
12014	1844053	adhE	0.717	0.937	0	0
13840	1844143	adhE	1.037	1.206	0	0
18045	1844455	adhE	0.930	1.296	0	0
1535	1844813	adhE	0.848	1.092	0	0
15463	1844930	adhE	1.030	1.226	0	0

9445	1845089	adhE	0.730	1.134	0	0
21053	1845356	adhE	0.676	1.214	0	0
4080	1845656	adhE	0.513	0.803	0	0
19114	1845822		0.710	0.899	0	0
8807	1846143	tdk	1.019	0.979	0	0
17074	1846252	tdk	1.094	0.939	0	0
5613	1846548	tdk	1.210	0.801	0	0
10043	1846619	tdk	1.229	0.756	0	0
13000	1847046	tdk, hns	1.263	0.952	0	0
5733	1847294	hns	1.426	1.404	0	0
18134	1847421	hns	1.266	1.327	0	0
8166	1847627	hns	1.320	1.328	0	0
14368	1847951	galU	1.254	1.008	0	0
19721	1848186	galU	0.829	1.041	0	0
19746	1848284	galU	0.954	1.150	0	0
7596	1848497	galU	0.900	0.992	0	0
22149	1848768	galU	0.944	1.015	0	0
19777	1849116	galU, hnr	0.980	1.113	0	0
20644	1849228	hnr	0.948	0.924	0	0
11089	1849566	hnr	0.956	1.208	0	0
9023	1849744	hnr	0.752	1.200	0	0
733	1849895	hnr	0.654	1.173	0	0
18733	1850194	hnr, ychK	0.695	1.391	0	0
2938	1850335	hnr, ychK	0.686	1.266	0	0
11030	1850628	ychK, ychJ	0.845	1.587	0	0
15249	1850737	ychK, ychJ	0.571	1.049	0	0
616	1850926	ychK, ychJ	0.739	1.711	0	0
19425	1851174	ychK, ychJ	0.671	1.157	0	0
20187	1851564	purU	0.720	1.322	0	0
2382	1851649	purU	0.653	1.318	0	0
2184	1851948	purU	0.547	1.460	0	0
4440	1852232	purU, tyrT	0.488	1.754	0	1
9453	1852439	tyrT, rtT	0.701	2.487	0	1
1360	1852653	rtT, tyrV	0.541	3.379	0	1
14804	1852975		0.593	2.743	0	1
4402	1853143		0.594	1.712	0	0

21334	1853317		0.942	1.948	0	1
8634	1853492		0.670	2.405	0	1
4989	1853838	STM1760	1.101	0.953	0	0
5276	1853909	STM1760	1.004	0.837	0	0
2865	1854359	STM1760	1.062	0.901	0	0
17488	1854529	STM1760	0.802	0.717	0	0
14790	1854827	STM1760	1.265	0.917	0	0
5937	1854921	STM1760	1.482	0.954	0	0
16663	1855102	STM1760	1.211	0.801	0	0
12009	1855321	STM1760	1.091	0.584	0	0
3031	1855467	STM1760, narI	1.692	0.779	0	0
1475	1855572	STM1760, narI	0.774	0.560	0	0
15187	1856133	narI	1.066	0.674	0	0
855	1856330	narI, narJ	1.154	0.651	0	0
15598	1856476	narI, narJ	1.364	0.623	0	0
12069	1856755	narJ	1.147	0.590	0	0
9707	1857059	narJ, narH	1.345	0.620	0	0
2171	1857252	narJ, narH	1.134	0.633	0	0
16614	1857508	narH	1.196	0.688	0	0
5106	1857675	narH	1.270	0.671	0	0
1307	1857903	narH	0.887	0.562	0	0
9623	1858204	narH	1.272	0.610	0	0
13403	1858439	narH, narG	0.847	0.680	0	0
4158	1858616	narH, narG	1.267	0.712	0	0
13039	1858748	narH, narG	1.174	0.730	0	0
7757	1859060	narG	1.261	0.665	0	0
13691	1859153	narG	1.395	0.781	0	0
5893	1859343	narG	1.499	0.758	0	0
19363	1859624	narG	1.243	0.630	0	0
7732	1859915	narG	1.060	0.645	0	0
16053	1860076	narG	1.435	0.773	0	0
21694	1860452	narG	0.364	0.649	0	0
13759	1860635	narG	1.430	0.926	0	0
5056	1860778	narG	1.437	0.736	0	0
9382	1861088	narG	0.760	0.643	0	0
7674	1861293	narG	1.018	0.777	0	0

6730	1861515	narG	1.104	0.777	0	0
13893	1861793	narG	1.220	0.796	0	0
12760	1862069	narG	0.824	0.650	0	0
6918	1862143	narG	1.571	0.572	0	0
6661	1862365	narG	1.666	0.607	0	0
15738	1862607	narK	2.065	0.649	0	0
19279	1862918	narK	0.867	0.457	0	0
9925	1863129	narK	0.826	0.499	0	0
17772	1863449	narK	1.164	0.684	0	0
10532	1863530	narK	1.310	0.574	0	0
18895	1863705	narK	1.190	0.817	0	0
20304	1863924	narK, narX	0.951	0.901	0	0
2990	1864255	narK, narX	0.952	1.027	0	0
12796	1864412	narX	1.117	1.381	0	0
12340	1864751	narX	1.020	0.987	0	0
11747	1864917	narX	1.175	0.924	0	0
10664	1865160	narX	1.171	1.144	0	0
15276	1865456	narX	0.720	1.124	0	0
19179	1865540	narX	0.941	1.426	0	0
5892	1865832	narX, narL	0.453	0.525	0	0
13866	1866029	narX, narL	0.998	0.980	0	0
273	1866260	narL	1.508	1.225	0	0
18686	1866604	narL	1.181	1.638	0	0
17506	1866697	ychP	1.010	1.353	0	0
6104	1866938	ychP	0.725	1.110	0	0
16755	1867255	ychP	0.781	0.701	0	0
16506	1867354	ychP	1.029	1.055	0	0
18441	1867714	ychP	0.992	1.002	0	0
16046	1867888	ychP, ychN	0.921	1.032	0	0
1806	1868110	ychP, ychN	1.077	0.892	0	0
4417	1868290	ychP, ychN	1.028	1.036	0	0
16160	1868412	ychP, ychN	0.964	1.054	0	0
10224	1868895	chaB, chaA	1.026	0.665	0	0
3352	1869067	chaB, chaA	1.382	0.905	0	0
10832	1869203	chaB, chaA	1.013	0.675	0	0
267	1869350	chaB, chaA	1.276	0.753	0	0

8268	1869810	chaA	1.648	0.765	0	0
8120	1869920	chaA	1.445	0.918	0	0
9762	1870079	chaA	1.221	1.267	0	0
5283	1870428	kdsA	0.855	0.975	0	0
10346	1870683	kdsA	0.860	1.345	0	0
6594	1870764	kdsA	0.800	1.266	0	0
10055	1870967	kdsA	0.857	1.181	0	0
6709	1871058	kdsA	1.016	1.134	0	0
5393	1871345	kdsA, ychA	0.826	0.973	0	0
2811	1871618	kdsA, ychA	0.846	1.225	0	0
2452	1871740	ychA	0.807	1.113	0	0
14714	1872232	ychA, sirC	0.846	1.248	0	0
15996	1872308	ychA, sirC	0.829	1.204	0	0
3797	1872526	ychA, sirC	0.738	1.016	0	0
19298	1872819	sirC, hemK	0.910	1.429	0	0
13566	1873198	hemK	0.962	1.491	0	0
5352	1873386	hemK, prfA	1.094	1.446	0	0
1838	1873644	hemK, prfA	0.828	0.859	0	0
6606	1873880	prfA	0.305	0.525	0	0
1973	1874116	prfA	0.603	1.019	0	0
10497	1874365	prfA	0.673	1.268	0	0
2905	1874597	prfA, hemA	0.675	1.992	0	0
1493	1874774	prfA, hemA	1.248	1.661	0	0
15844	1874921	hemA	0.407	0.828	0	0
16956	1875228	hemA	0.931	2.658	0	0
9472	1875428	hemA	0.467	1.329	0	0
2135	1875517	hemA	0.382	1.054	0	0
15042	1875730	hemA, lolB	0.658	2.068	0	1
20294	1875806	hemA, lolB	0.806	2.236	0	1
3679	1876194	lolB, ipk	0.733	1.736	0	0
8627	1876501	lolB, ipk	0.730	1.107	0	0
2511	1876606	lolB, ipk	0.702	1.233	0	0
9365	1876791	ipk	0.854	1.456	0	0
19514	1877224	ipk	0.552	3.120	0	1
9422	1877473	prsA	0.597	2.775	0	1
13005	1877746	prsA	0.465	3.105	0	1

4441	1877883	prsA	0.496	2.505	0	1
4088	1878187	prsA	0.459	2.396	0	1
4980	1878414	prsA, ychM	0.641	2.339	0	1
625	1878653	prsA, ychM	0.614	1.864	0	0
13324	1878752	ychM	0.561	1.342	0	0
17282	1878828	ychM	0.633	1.491	0	0
3842	1879233	ychM	1.065	0.970	0	0
1658	1879522	ychM	0.973	0.863	0	0
21475	1879597	ychM	0.236	0.475	0	0
5647	1879872	ychM	0.706	1.004	0	0
3418	1880050	ychM	0.630	0.873	0	0
13422	1880257	ychM	0.837	1.472	0	0
12831	1880597	ychH, pth	0.577	1.110	0	0
6874	1880830	ychH, pth	0.792	0.956	0	0
10370	1881092	ychH, pth	0.867	0.914	0	0
9201	1881235	ychH, pth	0.705	0.828	0	0
22229	1881534	pth, ychF	0.817	1.088	0	0
19749	1881787	ychF	0.707	1.167	0	0
22053	1882057	ychF	0.782	1.534	0	0
18104	1882152	ychF	0.923	1.215	0	0
12197	1882426	ychF	1.041	1.126	0	0
806	1882742	ychF	1.864	0.879	0	0
737	1882917		2.168	0.749	0	0
4189	1883140	STM1785	2.256	0.628	0	0
5288	1883249	STM1785	2.416	0.582	0	0
552	1883540	STM1785	2.632	0.572	0	0
3582	1883688	STM1785	2.551	0.534	0	0
19306	1884125	STM1785	3.787	0.538	1	0
3315	1884202	STM1785	2.838	0.514	1	0
9887	1884598	STM1785, STM1786	1.060	0.599	0	0
7019	1884796	STM1786	1.712	0.513	0	0
17125	1884869	STM1786	1.016	0.630	0	0
18692	1885122	STM1786	1.502	0.835	0	0
14687	1885421	STM1786	1.431	0.758	0	0
6706	1885545	STM1786, STM1787	1.053	0.632	0	0
21883	1885919	STM1787	1.067	0.698	0	0

2459	1886149	STM1787	0.598	0.451	0	0
12283	1886241	STM1787	0.967	0.580	0	0
12854	1886436	STM1787	1.049	0.614	0	0
5433	1886507	STM1787	1.414	0.715	0	0
3891	1886929	STM1787	1.236	0.681	0	0
5291	1887241	STM1787	1.055	0.672	0	0
19622	1887319	STM1787, STM1788	1.318	0.554	0	0
21976	1887710	STM1788	1.821	0.712	0	0
6486	1887910	STM1788	1.059	0.575	0	0
12276	1888173	STM1788, STM1789	1.054	0.726	0	0
21822	1888382	STM1788, STM1789	1.451	0.781	0	0
1696	1888559	STM1789	1.315	0.700	0	0
14669	1888725	STM1789, STM1790	1.673	0.897	0	0
824	1888991	STM1789, STM1790	2.255	0.692	0	0
8259	1889132	STM1790, STM1791	1.276	0.624	0	0
8253	1889521	STM1791	1.181	0.733	0	0
11792	1889700	STM1791	1.424	0.718	0	0
4355	1889822	STM1791	1.323	0.687	0	0
16225	1890083	STM1791, STM1792	1.422	0.674	0	0
4764	1890345	STM1792	1.021	0.539	0	0
12178	1890443	STM1792	0.928	0.621	0	0
693	1890863	STM1792	1.827	0.702	0	0
8504	1890978	STM1792	0.889	0.552	0	0
22340	1891270	STM1792	0.957	0.677	0	0
8158	1891586	STM1792, STM1793	0.726	0.556	0	0
12904	1891817	STM1792, STM1793	1.282	0.728	0	0
21588	1891924	STM1793	1.182	0.714	0	0
10460	1892007	STM1793	0.768	0.632	0	0
22070	1892434	STM1793	0.638	0.749	0	0
3815	1892759	STM1793, STM1794	0.779	0.740	0	0
5736	1892958	STM1793, STM1794	0.750	0.800	0	0
4472	1893063	STM1794, STM1795	0.764	0.957	0	0
10985	1893421	STM1795	0.734	1.100	0	0
4272	1893579	STM1795	0.640	1.226	0	0
4517	1893719	STM1795	0.920	1.938	0	0
19913	1894037	STM1795	0.407	0.782	0	0

4438	1894355	STM1795	0.443	0.980	0	0
14260	1894572	STM1795, treA	0.439	1.269	0	0
20898	1894827	treA	0.840	1.623	0	0
19061	1894931	treA	0.851	1.338	0	0
3041	1895225	treA	0.911	1.212	0	0
12957	1895337	treA	0.798	0.960	0	0
11625	1895471	treA	0.965	0.971	0	0
4132	1895751	treA	1.052	0.910	0	0
16931	1896059	treA	0.549	0.680	0	0
2882	1896205	treA	1.083	0.904	0	0
20599	1896529	treA	0.639	0.793	0	0
12217	1896666	treA	0.965	1.290	0	0
20745	1897067	ymgE, ycgR	1.248	2.539	0	1
1805	1897262	ymgE, ycgR	1.235	2.258	0	1
17023	1897371	ymgE, ycgR	1.264	2.397	0	1
7178	1897754	ycgR	0.820	1.556	0	0
21919	1897826	ycgR	0.965	1.655	0	0
5808	1898091	emtA	0.901	1.707	0	0
4432	1898391	emtA, ycgQ	0.932	1.355	0	0
10406	1898586	emtA, ycgQ	0.970	1.419	0	0
2225	1898730	emtA, ycgQ	1.130	1.359	0	0
8238	1899086	ycgQ	1.063	1.187	0	0
17902	1899349	ycgQ, ycgO	0.845	0.870	0	0
13548	1899527	ycgQ, ycgO	1.122	1.174	0	0
12464	1899679	ycgQ, ycgO	1.411	1.505	0	0
21298	1899865	ycgO	1.376	1.630	0	0
13377	1900123	ycgO	0.783	1.150	0	0
7072	1900290	ycgO	1.058	1.536	0	0
19993	1900683	ycgO	0.660	1.358	0	0
4336	1900879	ycgO	0.439	1.118	0	0
12664	1901014	ycgO	0.623	1.193	0	0
8921	1901269	ycgO	0.918	2.086	0	1
1365	1901350	ycgO	1.501	2.529	0	1
6316	1901458	ycgO	0.727	2.019	0	1
13232	1901949	dadX	0.785	1.990	0	1
17082	1902168	dadX	0.812	1.946	0	1

5898	1902264	dadX	0.794	1.634	0	0
9099	1902635	dadX	0.733	1.406	0	0
2460	1902839	dadX, dadA	0.498	1.134	0	0
8619	1903144	dadA	0.731	1.420	0	0
6487	1903286	dadA	0.994	1.442	0	0
17439	1903671	dadA	0.806	1.863	0	0
7122	1903881	dadA, ycgB	0.695	1.546	0	0
14183	1904065	dadA, ycgB	0.722	1.150	0	0
15435	1904168	dadA, ycgB	0.732	1.023	0	0
10791	1904585	ycgB	0.832	0.751	0	0
8140	1904768	ycgB	0.963	0.847	0	0
274	1904894	ycgB	1.269	0.878	0	0
4919	1905189	ycgB	1.081	0.872	0	0
3951	1905321	ycgB	1.011	0.923	0	0
8247	1905577	ycgB	0.756	1.119	0	0
19842	1905731	ycgB	0.712	1.510	0	0
1924	1906078	fadR	0.726	2.004	0	0
21833	1906357	fadR, nhaB	0.698	2.164	0	1
3355	1906600	fadR, nhaB	0.765	1.700	0	1
9551	1906788	fadR, nhaB	0.734	1.203	0	0
390	1906912	fadR, nhaB	0.669	1.144	0	0
18363	1907162	nhaB	0.712	1.044	0	0
10096	1907346	nhaB	0.302	0.484	0	0
18547	1907572	nhaB	0.499	0.663	0	0
12057	1907989	nhaB	0.672	0.890	0	0
20266	1908219	nhaB, dsbB	0.728	1.202	0	0
18704	1908398	dsbB	0.747	1.750	0	1
2175	1908483	dsbB	0.794	2.002	0	1
7112	1908599	dsbB	0.644	1.445	0	0
8248	1909113	STM1808	0.570	1.226	0	0
13897	1909300	STM1808	0.776	1.428	0	0
15404	1909560	STM1809	0.703	1.294	0	0
4083	1909693	STM1809	0.643	0.977	0	0
1343	1909909	STM1809, STM1810	0.740	0.922	0	0
16661	1910023	STM1809, STM1810	0.744	1.001	0	0
16559	1910370	STM1810	0.924	1.638	0	0

21471	1910608	ycgN	0.828	1.723	0	0
1813	1910814	ycgN	0.715	1.744	0	0
7220	1910952	ycgN, ycgM	0.837	1.632	0	0
8718	1911067	ycgN, ycgM	0.969	1.303	0	0
8533	1911589	ycgM, minC	0.811	1.108	0	0
11678	1911779	ycgM, ycgL	0.907	1.715	0	0
4392	1911977	ycgM, ycgL	0.605	1.294	0	0
378	1912085	ycgL, minC	0.745	1.680	0	0
19680	1912343	ycgL, minC	0.792	1.497	0	0
5099	1912752	minC, minD	0.626	1.593	0	0
5609	1912900	minD	0.597	1.639	0	0
3399	1913019	minD	0.534	1.379	0	0
2157	1913404	minD, minE	1.012	1.610	0	0
16106	1913536	minD, minE	1.019	1.530	0	0
8469	1913772	minE	1.317	1.253	0	0
5061	1914012	rnd	1.463	1.016	0	0
15756	1914099	rnd	1.521	1.127	0	0
11293	1914216	rnd	1.060	0.836	0	0
10271	1914626	rnd	1.065	0.834	0	0
15036	1914840	rnd	1.062	0.719	0	0
4044	1915018	rnd	1.016	1.041	0	0
22019	1915259	rnd, fadD	1.023	0.937	0	0
2665	1915513	rnd, fadD	1.014	0.804	0	0
20093	1915912	fadD	1.122	0.869	0	0
13931	1916055	fadD	1.063	0.953	0	0
420	1916372	fadD	0.934	0.883	0	0
15492	1916473	fadD	0.996	1.020	0	0
18804	1916729	fadD	0.975	1.139	0	0
8929	1916876	fadD	1.054	1.018	0	0
20234	1917099	fadD, slp	1.094	1.254	0	0
2819	1917253	fadD, slp	0.798	1.535	0	0
20215	1917729	slp	0.588	0.889	0	0
10612	1917858	slp, yeaZ	0.699	1.621	0	0
8036	1918144	yeaZ	0.441	1.129	0	0
20083	1918343	yeaZ	0.745	2.244	0	1
18945	1918430	yeaZ	0.800	2.015	0	1

19276	1918899	yeaZ, yoaA	1.005	1.299	0	0
9455	1919007	yoaA	1.041	1.222	0	0
2376	1919201	yoaA	1.690	1.117	0	0
17785	1919488	yoaA	0.804	0.972	0	0
4674	1919616	yoaA	0.532	0.640	0	0
10620	1919749	yoaA	0.976	1.076	0	0
388	1920008	yoaA	1.056	1.353	0	0
16911	1920436	yoaA, yoaB	0.812	0.986	0	0
20071	1920558	yoaA, yoaB	0.894	1.170	0	0
15395	1920720	yoaA, yoaB	0.972	1.546	0	0
11717	1920999	yoaH, pabB	0.708	1.451	0	0
5781	1921142	yoaH, pabB	0.856	1.450	0	0
7151	1921262	yoaH, pabB	0.495	0.721	0	0
2069	1921791	pabB	1.014	1.195	0	0
3401	1921920	pabB	0.924	1.012	0	0
20481	1922059	pabB, yeaB	1.078	1.117	0	0
14264	1922492	pabB, yeaB	0.898	1.248	0	0
4821	1922563	yeaB	1.125	1.293	0	0
7543	1922989	yeaB, sdaA	0.890	1.334	0	0
15864	1923175	sdaA	1.047	1.399	0	0
14220	1923379	sdaA	0.795	1.452	0	0
6716	1923453	sdaA	0.938	1.742	0	0
8647	1923872	sdaA	0.975	1.618	0	0
21141	1923981	sdaA	0.808	1.458	0	0
6380	1924265	sdaA	0.787	1.564	0	0
21449	1924533	sdaA, STM1827	0.477	0.881	0	0
21649	1924772	STM1827	0.679	1.207	0	0
7600	1924886	STM1827	0.815	1.227	0	0
400	1925163	STM1827	1.414	1.010	0	0
17190	1925280	STM1827	1.153	0.838	0	0
7813	1925508	STM1827	1.345	0.764	0	0
1220	1925915	STM1827	1.117	0.692	0	0
8686	1925986	STM1827	1.338	0.825	0	0
2290	1926104	STM1827	1.235	0.944	0	0
20375	1926528	yoaE	1.239	1.320	0	0
7627	1926644	yoaE	0.678	0.790	0	0

12212	1927057	yoaE	0.944	0.749	0	0
5650	1927182	yoaE	1.262	1.017	0	0
2097	1927366	yoaE	1.342	0.943	0	0
13363	1927651	yoaE	0.916	0.938	0	0
13754	1927966	yoaE	1.060	1.057	0	0
11433	1928097	yoaE, STM1829	0.940	0.939	0	0
3565	1928256	yoaE, STM1829	0.680	0.828	0	0
5520	1928510	STM1829, manX	0.712	0.874	0	0
10058	1928683	STM1829, manX	0.763	1.003	0	0
7291	1929064	manX, manY	0.870	0.958	0	0
8548	1929358	manX, manY	1.005	0.898	0	0
5983	1929515	manY	1.072	1.069	0	0
16090	1929609	manY	1.052	1.165	0	0
1681	1929687	manY	0.924	1.016	0	0
675	1930045	manY, manZ	1.078	1.252	0	0
9761	1930358	manZ	1.154	1.037	0	0
19500	1930633	manZ	1.775	1.152	0	0
11234	1930845	manZ, STM1833	1.107	0.751	0	0
17286	1931125	manZ, STM1833	1.853	1.121	0	0
13483	1931254	STM1833	2.468	0.877	0	0
4790	1931602	STM1833, yebN	5.035	0.863	1	0
4287	1931838	yebN	3.268	0.960	1	0
1852	1931946	yebN	2.622	1.486	1	0
1446	1932137	yebN	1.416	2.088	0	1
1715	1932325	yebN	0.897	1.928	0	1
9471	1932575	yebN	0.782	1.168	0	0
9669	1932911	rrmA	0.824	1.425	0	0
6631	1933047	rrmA	0.836	1.382	0	0
11316	1933448	rrmA	1.129	1.700	0	0
5566	1933572	rrmA, STM1836	0.875	1.436	0	0
9052	1933799	rrmA, STM1836	1.047	1.171	0	0
11860	1934077	STM1836	1.158	0.849	0	0
15587	1934262	STM1836	1.053	0.804	0	0
4907	1934599	STM1836	0.939	0.750	0	0
15307	1934676	STM1836	0.976	0.748	0	0
10177	1934865	STM1836	0.838	0.897	0	0

732	1935045	STM1836	0.728	1.050	0	0
18402	1935433	STM1836	0.528	1.236	0	0
9544	1935554	STM1836, cspC	0.575	1.532	0	0
8743	1935848	cspC, yobF	0.658	1.771	0	0
20896	1936096	cspC, yobF	0.778	1.481	0	0
8201	1936309	yobF	0.917	0.724	0	0
17711	1936553	STM1839	0.954	1.198	0	0
10079	1936810	STM1839, STM1841	1.033	1.104	0	0
6393	1937029	STM1839, yobG	1.209	0.928	0	0
11341	1937202	STM1839, yobG	1.634	0.898	0	0
8798	1937432	yobG	2.685	0.875	1	0
11571	1937651	kdgR	2.315	0.821	1	0
13964	1937832	kdgR	2.118	1.151	0	0
7034	1938040	kdgR	1.164	0.972	0	0
16374	1938428	kdgR, STM1843	1.407	1.236	0	0
4071	1938537	kdgR, STM1843	1.556	1.141	0	0
18543	1938861	kdgR, STM1843	1.240	0.787	0	0
13808	1939126	STM1843	0.724	0.718	0	0
5410	1939321	STM1843	0.958	0.887	0	0
22308	1939393	STM1843	0.995	1.119	0	0
14832	1939466	STM1843	1.219	1.106	0	0
10956	1939689	STM1843	0.367	0.587	0	0
6159	1940115	htpX	0.928	1.597	0	0
14104	1940438	htpX	0.770	1.573	0	0
11287	1940689	htpX	0.616	1.416	0	0
4422	1940903	htpX	0.587	1.388	0	0
6544	1941027	htpX	0.574	1.262	0	0
15697	1941378	htpX, prc	0.621	1.438	0	0
17858	1941523	prc	0.663	1.838	0	0
18977	1941696	prc	0.647	1.598	0	0
20724	1942018	prc	0.802	1.658	0	0
15252	1942091	prc	0.649	1.384	0	0
12196	1942444	prc	0.579	1.008	0	0
14077	1942620	prc	0.582	1.659	0	0
16005	1942944	prc	0.578	1.737	0	0
21651	1943214	prc, proQ	0.592	1.945	0	0

10008	1943304	prc, proQ	0.572	1.735	0	0
5067	1943663	proQ	0.951	1.338	0	0
5611	1943867	proQ	0.786	1.713	0	0
3180	1943942	proQ	0.726	1.716	0	0
1047	1944179	proQ, yebR	0.693	1.517	0	0
15744	1944579	yebR, yebS	0.701	1.153	0	0
3420	1944829	yebR, yebS	0.497	0.641	0	0
12815	1944901	yebR, yebS	0.316	0.525	0	0
17586	1945201	yebS	0.973	1.109	0	0
17831	1945355	yebS	1.032	1.122	0	0
574	1945583	yebS, STM1849	1.153	1.145	0	0
3279	1945722	yebS, STM1849	1.069	0.937	0	0
440	1946093	STM1849	1.248	1.149	0	0
7963	1946379	STM1849	1.077	0.968	0	0
2347	1946454	STM1849	0.900	0.741	0	0
4391	1946659	STM1849	0.683	0.787	0	0
4485	1946880	STM1849	1.041	1.090	0	0
17448	1947090	STM1849	1.276	1.484	0	0
4787	1947524	STM1849	1.087	1.230	0	0
6256	1947624	STM1849	0.604	0.641	0	0
3593	1947861	STM1849	1.422	2.595	0	0
10377	1948218	STM1849, yebU	1.212	1.241	0	0
19339	1948302	STM1849, yebU	0.753	0.934	0	0
19652	1948562	yebU	0.978	1.622	0	0
21251	1948687	yebU	1.183	1.874	0	0
14348	1949039	yebU	0.853	0.925	0	0
3746	1949176	yebU	0.857	0.854	0	0
18411	1949460	yebU	0.732	0.662	0	0
8670	1949615	yebU	1.034	0.908	0	0
3999	1949724	yebU, STM1851	1.108	0.981	0	0
5677	1950086	STM1851, yebW	1.193	0.851	0	0
14787	1950302	STM1851, yebW	0.965	0.923	0	0
14507	1950494	yebW	0.942	0.641	0	0
18208	1950851	prpA	1.181	0.718	0	0
12601	1950940	prpA	1.118	0.650	0	0
19330	1951184	prpA	1.489	0.661	0	0

13834	1951557	prpA, STM1854	1.716	0.640	0	0
12238	1951722	prpA, STM1854	2.409	0.583	0	0
20043	1951983	STM1854, sopE2	1.601	0.346	0	0
19034	1952169	sopE2	2.806	0.582	1	0
20081	1952529	sopE2	2.337	0.456	1	0
4191	1952686	sopE2	0.971	0.488	0	0
10236	1952760	sopE2	2.070	0.513	0	0
5263	1953084	sopE2, STM1856	1.705	0.486	0	0
8217	1953362	STM1856	1.918	0.332	0	0
11662	1953648	STM1856	2.509	0.508	0	0
7124	1953855		2.198	0.536	0	0
15411	1954111		1.884	0.680	0	0
11756	1954191		1.462	0.731	0	0
13286	1954454		1.411	0.911	0	0
16969	1954673		1.397	0.911	0	0
9399	1954940		1.437	0.873	0	0
15636	1955095	STM1857	1.371	0.846	0	0
4697	1955298	STM1857, STM1858	1.648	0.874	0	0
21680	1955524	STM1857, STM1858	1.140	0.708	0	0
14919	1955751	STM1857, STM1858	1.542	0.796	0	0
581	1956137	STM1859	0.996	0.672	0	0
9009	1956292	STM1859	0.910	0.647	0	0
10720	1956390		1.237	0.747	0	0
15861	1956618		1.398	0.822	0	0
13502	1956907	STM1860	0.841	0.501	0	0
17365	1957169	STM1860	1.132	0.616	0	0
7727	1957427	STM1860, STM1861	0.786	0.633	0	0
11142	1957683	STM1860, STM1861	0.948	0.541	0	0
1205	1957775	STM1860, STM1861	1.126	0.619	0	0
15332	1957967	STM1860, STM1861	1.585	0.765	0	0
4921	1958318		1.631	0.782	0	0
13821	1958509		1.535	0.800	0	0
13706	1958652		1.567	0.659	0	0
19523	1958802		1.165	0.593	0	0
10032	1959332	pagO	1.461	0.407	0	0
4356	1959506	pagO	1.866	0.498	0	0

16760	1959658	pagO	2.432	0.558	0	0
9283	1959894	pagO	2.051	0.439	0	0
6128	1960064	pagO, STM1863	2.227	0.528	0	0
10051	1960338	pagO, STM1863	2.496	0.467	1	0
15405	1960685	STM1864	2.647	0.532	1	0
18657	1960762	STM1864	2.076	0.468	0	0
928	1960881	STM1864	2.419	0.518	1	0
21872	1961305	STM1864	2.150	0.515	1	0
16620	1961523		1.877	0.472	0	0
7755	1961629		1.804	0.543	0	0
3873	1962055	STM1865	1.226	0.507	0	0
9186	1962194	STM1865	1.176	0.476	0	0
18307	1962327		2.219	0.545	0	0
16906	1962466		2.353	0.588	0	0
21063	1962849	pagK	2.195	0.502	0	0
490	1962954	pagK	2.315	0.511	0	0
10787	1963153	pagK	3.450	0.399	1	0
8239	1963621	mig-3	2.150	0.518	1	0
4876	1963755	mig-3	1.998	0.586	0	0
19958	1963999	mig-3	1.933	0.743	0	0
19997	1964250	mig-3	1.517	0.646	0	0
1140	1964416	mig-3	1.090	0.622	0	0
7375	1964798	STM1868A	1.679	0.607	0	0
3863	1964879	STM1868A	1.781	0.541	0	0
12058	1965218	STM1868A	2.680	0.463	1	0
7811	1965330		2.574	0.496	1	0
13926	1965515	STM1869	2.578	0.583	1	0
3208	1965590	STM1869	2.555	0.535	0	0
15212	1965971	STM1869	1.041	0.592	0	0
8452	1966338	STM1869, STM1869A	1.302	0.682	0	0
18659	1966413	STM1869, STM1869A	1.239	0.669	0	0
13140	1966612	STM1869A, STM1870	1.116	0.655	0	0
17392	1967038	STM1871	1.448	0.777	0	0
15394	1967275	STM1871	1.239	0.724	0	0
10171	1967524	STM1871	1.109	0.793	0	0
18199	1967702	STM1871	1.152	0.814	0	0

6818	1967974		1.143	1.032	0	0
20104	1968181	STM1872	0.921	1.301	0	0
11212	1968284	STM1872, STM1873	0.925	1.343	0	0
803	1968633	STM1872, STM1873	0.704	1.215	0	0
13630	1968846	STM1873, STM1874	0.736	1.333	0	0
4474	1968942	STM1873, STM1874	0.702	1.245	0	0
21797	1969156	STM1874	0.958	1.433	0	0
3169	1969477	STM1874	0.507	0.729	0	0
12510	1969558	STM1874, yobA	0.726	1.163	0	0
6610	1969875	STM1874, yobA	0.774	1.196	0	0
6251	1970226	yobA, holE	0.800	1.414	0	0
12130	1970342	yobA, STM1877	1.048	1.468	0	0
4581	1970458	STM1877	0.855	1.522	0	0
20733	1970813	STM1877, exoX	0.684	1.321	0	0
10540	1971114	exoX	0.837	1.613	0	0
12261	1971255	exoX	0.832	1.540	0	0
18354	1971392	exoX	1.015	1.706	0	0
13296	1971505	exoX	0.910	1.598	0	0
1195	1971907	ptrB	0.444	0.697	0	0
20908	1972121	ptrB	0.866	1.024	0	0
20873	1972485	ptrB	1.114	0.908	0	0
20555	1972640	ptrB	1.046	0.844	0	0
14432	1972854	ptrB	1.079	0.904	0	0
13141	1973172	ptrB	1.031	0.948	0	0
3623	1973370	ptrB	0.977	0.834	0	0
12468	1973584	ptrB	1.224	1.139	0	0
2774	1973811	ptrB	0.702	0.789	0	0
7049	1973995	ptrB	0.906	1.073	0	0
13283	1974125	ptrB, yebE	1.098	1.204	0	0
1309	1974552	yebE	1.312	1.319	0	0
22029	1974689	yebE	1.118	1.025	0	0
4332	1974898	yebE, yebF	0.766	0.956	0	0
15293	1975163	yebE, yebF	1.180	0.903	0	0
21357	1975458	yebF, yebG	1.221	0.950	0	0
10569	1975574	yebF, yebG	0.946	0.567	0	0
8494	1975860	yebG, purT	1.571	0.899	0	0

6616	1976029	purT	1.042	0.758	0	0
9171	1976226	purT	0.628	0.463	0	0
14951	1976515	purT	1.048	1.070	0	0
21261	1976751	purT	0.924	0.989	0	0
11964	1977067	eda	1.190	1.299	0	0
13242	1977184	eda	0.963	0.980	0	0
17465	1977529	eda	1.108	0.966	0	0
7661	1977755	eda, edd	0.584	0.622	0	0
13505	1977959	eda, edd	1.300	0.950	0	0
16946	1978180	edd	0.907	0.622	0	0
21099	1978312	edd	1.089	0.742	0	0
5908	1978431	edd	0.930	0.757	0	0
11641	1978814	edd	1.083	0.863	0	0
2154	1979134	edd	1.098	0.839	0	0
21043	1979209	edd	1.141	0.935	0	0
15075	1979564	edd	0.883	1.001	0	0
15150	1979638	edd, zwf	0.894	1.137	0	0
4861	1979898	zwf	0.910	1.269	0	0
18864	1980055	zwf	0.903	1.111	0	0
6994	1980393	zwf	0.887	1.068	0	0
10355	1980512	zwf	1.077	1.093	0	0
21918	1980583	zwf	0.860	1.031	0	0
13794	1980869	zwf	0.751	0.994	0	0
15800	1981199	zwf, yebK	0.671	0.692	0	0
2661	1981439	zwf, yebK	0.905	1.113	0	0
5714	1981719	yebK	0.965	1.339	0	0
21669	1981923	yebK	0.849	1.420	0	0
18063	1982289	yebK, pykA	0.968	1.304	0	0
17990	1982430	pykA	0.731	1.122	0	0
16135	1982704	pykA	0.848	1.098	0	0
7007	1982866	pykA	0.809	1.190	0	0
18648	1982995	pykA	0.789	1.198	0	0
1037	1983157	pykA	0.760	1.138	0	0
5829	1983500	pykA	0.705	1.298	0	0
11511	1983862	pykA	0.834	1.651	0	0
7372	1983971	msbB	1.049	1.212	0	0

9979	1984344	msbB	0.671	1.008	0	0
2034	1984430	msbB	0.941	1.370	0	0
8681	1984584	msbB	0.652	1.103	0	0
12967	1984935	msbB	0.855	1.444	0	0
6698	1985166	msbB, yebA	0.707	1.746	0	0
3218	1985308	msbB, yebA	0.436	0.827	0	0
12422	1985500	yebA	0.785	1.626	0	1
5256	1985694	yebA	0.760	1.763	0	1
15854	1986037	yebA	0.727	2.001	0	1
1039	1986203	yebA	0.787	2.044	0	1
19790	1986432	yebA, znuA	0.872	2.231	0	1
21642	1986697	yebA, znuA	0.794	2.255	0	1
2662	1987010	znuC, znuA	0.838	1.291	0	0
14520	1987148	znuC, znuA	0.842	1.337	0	0
9395	1987416	znuC, znuA	0.906	1.097	0	0
2105	1987657	znuC, znuA	0.725	0.897	0	0
21077	1987973	znuC, zunB	0.863	0.788	0	0
9238	1988147	znuC, zunB	0.948	0.991	0	0
20356	1988396	zunB	0.643	0.797	0	0
15239	1988557	zunB	0.889	1.408	0	0
9150	1988840	zunB	0.495	0.882	0	0
13448	1989123	ruvB	0.833	1.292	0	0
17960	1989256	ruvB	0.678	1.026	0	0
12684	1989347	ruvB	0.480	0.847	0	0
21101	1989708	ruvB	1.136	1.235	0	0
12092	1989810	ruvB	1.207	1.351	0	0
15500	1990112	ruvB, ruvA	1.383	1.136	0	0
16437	1990261	ruvB, ruvA	1.442	0.991	0	0
6278	1990689	ruvA, STM1896	4.116	0.731	1	0
12457	1990904	ruvA, STM1896	4.326	0.531	1	0
10110	1991074	ruvA, STM1896	4.621	0.578	1	0
13731	1991220	STM1896	3.943	0.648	1	0
11495	1991592	STM1896	2.636	0.525	0	0
18948	1991760	STM1896	3.166	0.515	1	0
13165	1992069		3.474	0.515	1	0
15645	1992286	yebB	4.369	0.575	1	0

8994	1992442	yebB	3.983	0.541	1	0
18212	1992619	yebB	3.830	0.524	1	0
21274	1992712	yebB	2.929	0.585	1	0
13993	1992969	yebB	2.256	0.817	1	0
14383	1993147	yebB	1.479	0.851	0	0
16339	1993536	ruvC	1.078	1.567	0	0
1619	1993798	ruvC, yebC	0.839	1.353	0	0
15134	1994093	ruvC, yebC	0.787	1.320	0	0
12358	1994249	yebC	0.792	1.231	0	0
1419	1994479	yebC	0.619	1.010	0	0
12813	1994749	yebC, ntpA	0.884	1.499	0	0
2494	1994920	yebC, ntpA	0.799	1.263	0	0
14852	1995081	ntpA, aspS	0.964	1.635	0	0
956	1995359	ntpA, aspS	0.937	1.441	0	0
21569	1995569	aspS	0.680	1.259	0	0
15940	1995824	aspS	0.905	1.329	0	0
7264	1995998	aspS	0.852	1.235	0	0
13646	1996205	aspS	0.700	0.845	0	0
4328	1996398	aspS	0.910	1.472	0	0
3900	1996839	aspS, yecD	0.769	2.834	0	1
13692	1996944	aspS, yecD	0.735	2.496	0	1
17182	1997168	aspS, yecD	0.764	1.544	0	0
20595	1997448	yecD, yecE	0.592	0.901	0	0
11335	1997750	yecE	0.933	1.362	0	0
19017	1997919	yecE	0.813	1.418	0	0
19097	1998137	yecE	0.968	1.061	0	0
11822	1998349	yecE, yecN	1.002	1.275	0	0
16908	1998567	yecN	0.775	1.353	0	0
917	1998657	yecN, yecO	0.816	1.342	0	0
21857	1998988	yecN, yecO	0.853	2.244	0	1
6857	1999164	yecO	0.722	2.034	0	1
700	1999435	yecO, yecP	0.828	1.568	0	0
11458	1999814	yecP	0.914	1.598	0	0
21325	1999922	yecP	0.999	1.459	0	0
13518	2000032	yecP	0.869	1.501	0	0
1428	2000447	yecP	0.906	0.961	0	0

7542	2000584	yecP	0.952	1.534	0	0
5430	2000779		0.974	1.434	0	0
9061	2001006	cutC	0.750	1.219	0	0
3286	2001248	cutC	0.988	1.104	0	0
20789	2001441	cutC	0.800	0.971	0	0
3774	2001734	cutC	0.767	0.716	0	0
11210	2002068	cutC, yecM	1.050	0.953	0	0
4613	2002295	yecM, argS	1.087	1.176	0	0
8412	2002412	yecM, argS	0.878	1.104	0	0
16460	2002565	yecM, argS	1.191	1.449	0	0
2713	2002830	argS	0.737	1.350	0	0
15689	2003156	argS	1.235	1.857	0	0
753	2003280	argS	0.852	1.670	0	0
16706	2003679	argS	0.813	1.481	0	0
14349	2003890	argS	0.791	1.493	0	0
8869	2003966	argS	0.687	1.185	0	0
19487	2004364	STM1910	0.779	0.884	0	0
10037	2004581	STM1910	0.443	0.581	0	0
20500	2004780	STM1910	0.723	0.891	0	0
7134	2004940	STM1910	0.948	0.747	0	0
1055	2005143	STM1910	1.308	0.833	0	0
20078	2005387	STM1910	1.088	0.709	0	0
3662	2005626	STM1910	1.077	0.713	0	0
14503	2005697	STM1910	1.032	0.725	0	0
10348	2005947	STM1910	0.898	0.755	0	0
22272	2006153	STM1910	1.098	0.920	0	0
6140	2006430	STM1911	0.614	0.652	0	0
21854	2006567	STM1911	0.890	1.262	0	0
19340	2006959	STM1911	0.992	1.124	0	0
11819	2007299	STM1911	0.716	0.794	0	0
5593	2007526	STM1911	0.931	0.832	0	0
2961	2007712	STM1911	0.816	0.964	0	0
13026	2007938	STM1911, flhE	0.482	0.971	0	0
8231	2008167	flhE	0.480	0.910	0	0
11885	2008240	flhE, flhA	0.563	0.964	0	0
20188	2008489	flhE, flhA	0.684	1.455	0	0

15369	2008701	flhA	0.862	1.553	0	0
15078	2009109	flhA	1.053	1.585	0	0
19697	2009183	flhA	0.748	1.187	0	0
20784	2009330	flhA	0.985	1.653	0	0
17732	2009722	flhA	0.816	1.480	0	0
18145	2009858	flhA	1.003	1.344	0	0
16914	2010114	flhA	1.144	1.723	0	1
4872	2010266	flhA, flhB	0.803	1.715	0	1
10906	2010581	flhA, flhB	0.592	1.288	0	0
18225	2010762	flhB	0.764	1.796	0	1
21952	2011051	flhB	0.837	2.498	0	1
20569	2011173	flhB	0.727	2.595	0	1
16235	2011435	flhB	0.614	2.373	0	1
15445	2011775	flhB, cheZ	0.578	2.158	0	1
11572	2011864	flhB, cheZ	0.578	2.057	0	1
20861	2012267	cheZ, cheY	0.554	1.649	0	0
17783	2012529	cheZ, cheY	0.730	2.192	0	1
20690	2012609	cheZ, cheY	0.770	2.124	0	1
2520	2012710	cheZ, cheY	0.676	1.676	0	0
11978	2013089	cheY, cheB	0.643	1.132	0	0
21981	2013371	cheB	0.792	1.691	0	0
9554	2013633	cheB	0.596	1.219	0	0
16070	2013869	cheB, cheR	0.883	1.543	0	0
4807	2013940	cheB, cheR	0.873	1.579	0	0
15148	2014340	cheR	0.557	1.361	0	0
1913	2014573	cheR	0.826	1.967	0	0
18486	2014656	cheR	0.734	1.716	0	0
7300	2014727	cheR, cheM	0.740	1.981	0	0
9414	2015122	cheM	0.716	1.570	0	0
4733	2015328	cheM	0.800	1.247	0	0
6703	2015571	cheM	0.792	1.730	0	0
6854	2015725	cheM	0.805	1.698	0	0
8701	2015995	cheM	0.766	1.796	0	1
4295	2016379	cheM	0.761	1.943	0	1
22071	2016468	cheM	0.842	2.188	0	1
21751	2016686	cheM, cheW	0.642	2.202	0	1

11589	2017046	cheW	0.654	2.484	0	1
9067	2017124	cheW	0.673	2.730	0	1
3078	2017294	cheW, cheA	0.444	1.478	0	0
10543	2017657	cheA	0.485	1.874	0	0
1244	2017893	cheA	0.737	2.106	0	1
7374	2018134	cheA	0.645	2.070	0	1
9848	2018280	cheA	0.574	1.964	0	0
16132	2018645	cheA	0.733	2.077	0	1
12836	2018861	cheA	0.759	2.178	0	1
16204	2018967	cheA	0.635	1.925	0	1
525	2019153	cheA	0.552	1.748	0	0
5661	2019256	cheA, motB	0.697	2.455	0	1
8641	2019653	motB	0.561	1.946	0	1
4785	2019930	motB	0.776	2.124	0	1
18419	2020170	motB, motA	0.722	1.683	0	0
10150	2020337	motB, motA	0.717	1.862	0	0
9541	2020547	motB, motA	0.637	2.171	0	0
2214	2020795	motA	0.800	1.822	0	0
9202	2020985	motA	0.631	1.856	0	0
8884	2021154	motA, flhC	0.621	2.010	0	1
13850	2021485	flhC	0.649	1.851	0	1
843	2021813	flhC, flhD	0.868	1.840	0	0
13514	2022043	flhC, flhD	0.740	1.611	0	0
3325	2022199	flhD	0.805	1.210	0	0
19965	2022428	flhD, STM1926	0.907	0.783	0	0
21403	2022667	STM1926, yecG	1.199	0.863	0	0
14347	2022816	STM1926, yecG	1.169	0.996	0	0
14157	2023042	yecG	1.010	1.034	0	0
22176	2023370	otsA	0.976	1.178	0	0
11690	2023659	otsA	1.475	0.933	0	0
20956	2023836	otsA	1.490	0.877	0	0
21041	2024037	otsA	0.955	0.757	0	0
14709	2024229	otsA	1.307	0.836	0	0
22143	2024464	otsA	1.209	0.717	0	0
10597	2024701	otsA, otsB	0.808	0.728	0	0
5266	2024867	otsA, otsB	0.700	0.685	0	0

15922	2025178	otsB	0.971	1.293	0	0
3360	2025411	otsB	0.777	2.323	0	0
15136	2025489	otsB	0.434	1.248	0	0
5840	2025857	otsB	0.490	0.762	0	0
17671	2026007		1.097	1.232	0	0
7156	2026212	araH	1.130	0.889	0	0
6938	2026597	araH	2.415	0.622	1	0
20123	2026777	araH, ftnB	2.679	0.551	1	0
20658	2026946	araH, ftnB	2.766	0.491	1	0
11403	2027124	araH, ftnB	2.371	0.521	0	0
4469	2027293	ftnB	1.939	0.593	0	0
20073	2027448	ftnB	1.652	0.687	0	0
15453	2027923	STM1933	0.821	0.543	0	0
14795	2028008	STM1933	1.083	0.638	0	0
12230	2028383	STM1933, STM1934	1.248	0.607	0	0
7666	2028600	STM1933, STM1934	1.093	0.666	0	0
20442	2028797	STM1933, STM1934	1.665	0.994	0	0
17804	2028924	STM1933, STM1934	1.925	0.957	0	0
7491	2029367	ftn	3.208	0.727	1	0
21693	2029441	ftn	2.504	0.847	1	0
9297	2029722	ftn	2.501	0.733	1	0
9482	2029831	ftn	2.737	1.175	1	0
9936	2030144	yecH, tyrP	1.668	1.075	0	0
17347	2030296	yecH, tyrP	1.152	0.882	0	0
2308	2030616	tyrP	1.119	0.843	0	0
8225	2030823	tyrP	1.219	1.065	0	0
3587	2030966	tyrP	1.249	0.948	0	0
17877	2031041	tyrP	1.154	1.280	0	0
12110	2031551	tyrP	0.884	1.541	0	0
14899	2031653		0.847	1.323	0	0
13096	2031986	yecA	0.930	1.122	0	0
11737	2032294	yecA	1.251	1.185	0	0
3267	2032448	yecA	1.599	1.596	0	0
20981	2032652	yecA, STM1939	2.082	1.182	0	0
13662	2032935	STM1939	1.909	1.097	0	0
7714	2033105	STM1939, STM1940	2.326	1.171	0	0

7209	2033356	STM1940	1.988	0.769	0	0
20398	2033496	STM1940	1.760	0.879	0	0
17397	2033822	STM1940	1.369	0.965	0	0
854	2034090	STM1940	1.452	0.926	0	0
19645	2034319	STM1940	2.138	0.805	0	0
13661	2034447	STM1940	1.983	0.928	0	0
15210	2034612	STM1940	2.244	0.915	0	0
18522	2034921	STM1940, STM1941	1.574	1.305	0	0
19442	2035156	STM1941	0.974	2.652	0	1
15011	2035310	STM1941	0.544	3.543	0	1
336	2035656	leuZ, cysT	0.348	3.916	0	1
7969	2035731	leuZ, cysT	0.368	3.323	0	1
5189	2035815	leuZ, cysT	0.446	3.246	0	1
5782	2036322	pgsA	0.832	1.766	0	1
20340	2036398	pgsA, uvrC	0.730	1.876	0	1
6650	2036557	pgsA, uvrC	0.848	2.114	0	1
16749	2037035	uvrC	0.882	1.463	0	0
13574	2037270	uvrC	0.905	1.553	0	0
20086	2037350	uvrC	0.781	1.454	0	0
21498	2037651	uvrC	0.531	1.254	0	0
20890	2037912	uvrC	0.691	1.239	0	0
14965	2038111	uvrC	0.702	1.511	0	0
5399	2038355	uvrC, uvrY	0.647	1.269	0	0
17607	2038467	uvrC, uvrY	0.723	1.323	0	0
20808	2038872	uvrY, STM1948	1.468	1.657	0	0
7565	2038993	uvrY, STM1948	1.392	1.141	0	0
1009	2039330	uvrY, STM1948	1.540	1.020	0	0
1406	2039522	yecF	1.195	1.085	0	0
14035	2039745	sdiA	1.049	1.233	0	0
7397	2039820	sdiA	0.957	0.962	0	0
21094	2040079	sdiA	1.058	0.951	0	0
5823	2040348	sdiA	1.510	0.945	0	0
7688	2040509	sdiA	1.462	0.935	0	0
9538	2040600	sdiA, yecC	1.573	1.111	0	0
9765	2041042	yecC	0.773	0.716	0	0
12349	2041317	yecC	1.158	1.128	0	0

16589	2041392	yecC, yecS	1.347	1.206	0	0
17237	2041764	yecC, yecS	0.807	0.683	0	0
14411	2042010	yecS	1.379	0.932	0	0
18597	2042095	yecS, yedO	1.118	1.052	0	0
22337	2042321	yecS, yedO	0.642	0.699	0	0
15662	2042662	yedO	1.066	0.993	0	0
16316	2042900	yedO	0.785	0.865	0	0
21980	2043016	yedO	0.356	0.616	0	0
8254	2043206	yedO, fliY	0.784	1.715	0	1
8427	2043622	fliY	0.701	2.449	0	1
7446	2043747	fliY	0.640	2.368	0	1
4591	2043973	fliY	0.662	3.091	0	1
19113	2044076	fliY	0.589	2.454	0	1
12186	2044384	fliY, fliZ	0.666	2.671	0	1
12265	2044511	fliZ	0.624	2.706	0	1
10118	2044746	fliZ, fliA	0.438	2.994	0	1
15580	2045019	fliZ, fliA	0.581	2.873	0	1
5657	2045432	fliA	0.653	2.142	0	1
17641	2045561	fliA	0.877	1.601	0	0
11149	2045854	fliA, tnpA_2	1.194	0.881	0	0
13356	2046055	tnpA_2	1.351	0.812	0	0
19329	2046367	tnpA_2, fliB	1.041	1.072	0	0
15585	2046456	tnpA_2, fliB	1.037	1.024	0	0
7389	2046643	fliB	0.927	0.948	0	0
8307	2047035	fliB	0.810	0.969	0	0
19069	2047121	fliB	0.840	0.941	0	0
3701	2047449	fliB	0.855	1.493	0	0
4117	2047542	fliB	0.704	1.681	0	0
13277	2047979	fliB, fliC	0.713	1.693	0	0
12190	2048202	fliC	0.467	1.035	0	0
10451	2048417	fliC	0.630	1.516	0	0
12280	2048502	fliC	0.649	1.426	0	0
1511	2048574	fliC	0.526	1.545	0	0
17425	2049136	fliC, fliD	0.846	2.063	0	1
13238	2049355	fliC, fliD	0.772	1.905	0	1
8577	2049426	fliC, fliD	0.715	2.150	0	1

5659	2049542	fliC, fliD	0.783	2.176	0	1
6548	2049786	fliD	0.901	1.823	0	1
18783	2050001	fliD	0.430	1.404	0	0
7642	2050442	fliD, fliS	0.595	2.195	0	1
5663	2050533	fliD, fliS	0.589	2.255	0	1
8984	2050707	fliD, fliS	0.498	1.496	0	0
8126	2050951	fliS, fliT	0.672	2.360	0	1
3330	2051304	fliT, amyA	0.544	2.239	0	1
10786	2051585	amyA	0.727	2.356	0	1
3608	2051825	amyA	0.791	1.669	0	1
10572	2051924	amyA	0.605	1.004	0	0
1031	2052218	amyA	1.129	0.832	0	0
20664	2052364	amyA	1.097	0.847	0	0
16074	2052470	amyA	1.070	0.859	0	0
10186	2052893	amyA	0.966	1.115	0	0
18188	2053025	amyA	0.877	1.059	0	0
8657	2053317	yedD, yedE	1.216	1.439	0	0
8602	2053609	yedD, yedE	1.304	1.288	0	0
18375	2053761	yedD, yedE	1.121	1.215	0	0
8746	2053963	yedD, yedE	1.124	0.925	0	0
20153	2054365	yedE	1.186	0.723	0	0
10065	2054598	yedE, yedF	1.184	0.791	0	0
22226	2054775	yedE, yedF	1.289	0.835	0	0
17597	2054911	yedE, yedF	1.878	1.151	0	0
14751	2055190	yedF, STM1967	2.560	1.118	0	0
19971	2055275	STM1967	2.579	0.982	0	0
8608	2055636	STM1967	2.501	1.262	1	0
5095	2055756	STM1967	2.926	1.750	1	0
6370	2055833	STM1967	2.464	1.881	1	0
14577	2056288	fliE, fliF	0.650	1.613	0	0
18135	2056418	fliE, fliF	0.923	2.276	0	1
5972	2056534	fliE, fliF	0.651	2.084	0	1
14247	2056874	fliF	0.713	2.259	0	1
16203	2057224	fliF	0.729	2.600	0	1
12825	2057317	fliF	0.782	2.780	0	1
17128	2057753	fliF, fliG	0.939	2.498	0	1

16913	2057879	fliF, fliG	0.325	0.910	0	0
6168	2058207	fliG	0.788	2.381	0	1
5697	2058350	fliG	0.801	1.973	0	1
522	2058495	fliG	0.805	2.391	0	1
8102	2058893	fliG, fliH	0.582	1.423	0	0
15775	2059098	fliG, fliH	0.688	2.045	0	1
15113	2059191	fliH	0.773	2.930	0	1
1665	2059510	fliH, fliI	0.737	2.990	0	1
4508	2059635	fliH, fliI	0.776	2.432	0	1
9760	2059940	fliI	0.811	3.045	0	1
21304	2060087	fliI	0.913	3.059	0	1
10851	2060367	fliI	0.714	2.457	0	1
6752	2060517	fliI	0.873	2.409	0	1
8919	2060866	fliI, fliJ	0.509	1.108	0	0
10250	2061147	fliI, fliJ	1.032	2.227	0	1
15947	2061252	fliJ, fliK	0.792	2.007	0	1
8337	2061413	fliJ, fliK	0.820	2.244	0	1
22167	2061687	fliJ, fliK	0.899	2.505	0	1
14843	2062019	fliK	0.835	2.748	0	1
16166	2062279	fliK	0.464	1.015	0	0
9257	2062529	fliK	0.700	2.104	0	0
21740	2062614	fliK, fliL	0.861	3.027	0	0
16034	2062985	fliL	0.686	1.777	0	0
13752	2063075	fliL, fliM	0.590	2.156	0	1
9869	2063228	fliL, fliM	0.761	3.139	0	1
4372	2063478	fliL, fliM	0.632	2.652	0	1
3632	2063759	fliM	0.609	2.784	0	1
6220	2064048	fliM, fliN	0.920	2.085	0	1
16377	2064143	fliM, fliN	0.557	1.937	0	1
12341	2064498	fliM, fliN	0.661	2.417	0	1
5517	2064668	fliN, fliO	0.873	1.772	0	0
4537	2064893	fliN, fliO	0.797	2.236	0	1
821	2065063	fliO, fliP	0.933	2.566	0	1
14612	2065283	fliO, fliP	0.736	2.775	0	1
6696	2065493	fliP	0.642	2.553	0	1
15986	2065846	fliP, fliQ	0.593	1.446	0	0

20429	2066044	fliQ, fliR	0.772	1.917	0	0
10098	2066338	fliR	0.650	1.348	0	0
3082	2066570	fliR	1.054	1.389	0	0
20834	2066728	fliR	1.361	1.140	0	0
14537	2067067	fliR, rcsA	2.376	0.708	1	0
13815	2067292	rcsA	2.952	0.675	1	0
10900	2067370	rcsA	2.740	0.621	1	0
21087	2067560	rcsA	2.634	0.514	1	0
13671	2067798	rcsA	2.621	0.632	1	0
18470	2067987	rcsA, yodD	2.003	0.765	0	0
21571	2068359	dsrB, yodD	1.058	0.907	0	0
3489	2068587	dsrB, yodD	1.135	1.182	0	0
18109	2068691	dsrB, dsrA	1.101	1.133	0	0
9575	2068928	dsrA, yedP	1.040	1.042	0	0
14436	2069251	yedP	1.102	0.762	0	0
14109	2069585	yedP	0.884	0.841	0	0
16234	2069808	STM1987	1.066	0.868	0	0
5676	2069912	STM1987	1.150	0.817	0	0
7724	2070099	STM1987	0.826	0.632	0	0
14875	2070280	STM1987	1.469	0.893	0	0
10030	2070585	STM1987	1.145	0.754	0	0
9533	2070821	STM1987	1.241	0.872	0	0
11244	2071053	STM1987	0.899	0.838	0	0
10971	2071308	STM1987	1.115	1.112	0	0
11713	2071425	STM1987	0.934	1.033	0	0
14710	2071807	STM1987, STM1988	1.216	1.396	0	0
10712	2072058	STM1988, yedI	0.901	0.910	0	0
17472	2072193	STM1988, yedI	0.707	0.698	0	0
18824	2072441	yedI	1.462	0.820	0	0
14378	2072752	yedI, yedA	1.738	0.849	0	0
12165	2072919	yedI, yedA	2.014	0.807	0	0
14472	2073003	yedI, yedA	1.410	0.599	0	0
20206	2073278	yedA	1.185	0.702	0	0
19263	2073624	yedA	1.226	1.216	0	0
12663	2073787	yedA	0.320	0.575	0	0
12301	2074114	vsr	1.016	1.059	0	0

9016	2074220	vsr	1.110	1.236	0	0
4047	2074571	vsr, dcm	0.807	0.930	0	0
5105	2074733	vsr, dcm	0.795	0.694	0	0
13963	2074854	dcm	1.396	1.231	0	0
13024	2075009	dcm	0.475	0.538	0	0
18348	2075358	dcm	1.238	0.977	0	0
20474	2075644	dcm	1.397	1.293	0	0
3534	2075775	dcm	1.051	0.873	0	0
11193	2075985	dcm, yedJ	1.387	0.891	0	0
1113	2076307	yedJ	1.257	0.864	0	0
7098	2076532	yedJ	1.518	0.891	0	0
20893	2076821	yedJ, STM1994	2.403	0.639	1	0
21794	2077067	STM1994	3.200	0.439	1	0
17964	2077163	STM1994, ompS	2.671	0.369	1	0
13664	2077277	STM1994, ompS	2.140	0.393	0	0
16886	2077554	ompS	2.739	0.502	0	0
14338	2077969	ompS	1.605	0.595	0	0
18037	2078188	ompS	1.166	0.703	0	0
20672	2078259	ompS	0.670	0.493	0	0
21997	2078561	ompS	1.337	0.620	0	0
5615	2078908		1.867	0.527	0	0
5863	2079129		1.734	0.583	0	0
13273	2079261	cspB	1.476	0.539	0	0
5885	2079419	cspB	1.894	0.591	0	0
9476	2079765	cspB	2.197	0.991	0	0
14025	2079840	cspB	1.900	1.001	0	0
13824	2080152	umuC	1.285	1.043	0	0
547	2080243	umuC	1.096	0.749	0	0
17384	2080499	umuC	1.068	0.809	0	0
7320	2080887	umuC	1.151	0.812	0	0
18484	2081091	umuC	1.105	0.764	0	0
7146	2081175	umuC, umuD	0.941	0.760	0	0
14891	2081410	umuC, umuD	0.966	0.894	0	0
10442	2081641	umuD	0.848	1.033	0	0
1878	2081974	umuD, STM1999	0.789	1.922	0	0
2483	2082116	STM1999, yeeI	0.578	1.772	0	0

15937	2082323	serU, yeeI	0.592	2.554	0	0
6576	2082522	serU, yeeI	0.613	1.621	0	0
11567	2082729	yeeI	0.584	1.452	0	0
12911	2083127		0.525	3.194	0	1
17504	2083383		0.423	3.369	0	1
17872	2083478		0.345	1.579	0	0
18494	2083786	asnT	0.634	1.746	0	1
18592	2084082	asnT, STM2005	0.472	2.511	0	1
8408	2084362	STM2005	0.453	3.853	0	1
11275	2084506	STM2005	0.539	2.652	0	1
12095	2084825	STM2005	0.669	1.315	0	0
11836	2084929	STM2006	0.599	0.995	0	0
17755	2085167	STM2006	0.855	0.719	0	0
4116	2085373	STM2006	0.695	0.636	0	0
260	2085474	STM2006	1.238	0.700	0	0
9193	2085645	STM2006	1.086	0.565	0	0
13414	2086028	STM2006	5.433	0.634	1	0
9884	2086310		4.226	0.490	1	0
9060	2086486	STM2007	4.943	0.548	1	0
14459	2086836	STM2007	3.033	0.552	1	0
3883	2086925	STM2007	2.913	0.584	0	0
6959	2087225	STM2007	3.635	0.595	1	0
22270	2087520	STM2007	2.879	0.571	1	0
2492	2087592	STM2007	2.657	0.543	1	0
20441	2087822	STM2007	1.397	0.505	0	0
14756	2087940	STM2007	1.312	0.531	0	0
11731	2088421	STM2007, STM2008	1.384	0.525	0	0
8971	2088617	STM2008	1.973	0.527	0	0
12435	2088753	STM2008	1.744	0.557	0	0
18497	2089116	STM2008	3.155	0.511	1	0
8762	2089348	STM2008	2.923	0.506	1	0
13289	2089525	STM2008	2.638	0.555	1	0
5431	2089828	STM2008	2.274	0.688	0	0
4319	2089940	STM2008	1.161	0.646	0	0
11163	2090087	STM2008	1.027	0.505	0	0
11922	2090281	STM2008	1.646	0.928	0	0

14057	2090567		1.080	1.118	0	0
18206	2090756		0.396	0.558	0	0
21985	2090979		1.209	1.440	0	0
14944	2091337	amn	1.393	1.219	0	0
342	2091449	amn	1.333	0.915	0	0
13682	2091807	amn	0.941	0.964	0	0
21384	2092044	amn	1.167	1.022	0	0
21544	2092119	amn	1.087	0.948	0	0
8524	2092417	amn, STM2010	1.002	1.140	0	0
21256	2092755	amn, STM2010	0.826	1.085	0	0
2788	2092903	amn, STM2010	0.865	1.149	0	0
15586	2093136	amn, STM2011	0.547	0.845	0	0
458	2093449	STM2011	0.743	1.589	0	0
7750	2093640	STM2011	0.516	1.443	0	0
4565	2093821		0.451	1.863	0	1
8050	2093897		0.382	2.170	0	1
4498	2094274	asnW	0.403	3.279	0	1
10948	2094457	asnW, yeeO	0.426	1.406	0	0
18635	2094661	asnW, yeeO	0.690	1.154	0	0
19427	2094753	yeeO	0.801	1.242	0	0
1091	2095174	yeeO	0.988	1.005	0	0
12687	2095383	yeeO	0.845	1.182	0	0
3938	2095658	yeeO, asnU	0.634	1.448	0	0
4127	2095889	yeeO, asnU	0.458	3.419	0	1
13533	2096084	yeeO	0.472	3.843	0	1
15005	2096234	yeeO	0.488	3.365	0	1
11575	2096557	erfK	0.744	1.761	0	1
1376	2096639	erfK	0.889	1.453	0	0
12870	2097065	erfK	1.014	1.155	0	0
14100	2097174	erfK	1.129	1.208	0	0
19432	2097256	erfK	0.676	0.960	0	0
12924	2097416	erfK, cobT	1.016	1.179	0	0
15690	2097989	cobT	1.112	0.949	0	0
785	2098209	cobT	1.096	0.893	0	0
7290	2098351	cobT	0.701	0.658	0	0
22251	2098582	cobT, cobS	1.239	0.989	0	0

20487	2098735	cobT, cobS	1.074	0.847	0	0
8872	2099116	cobS, cobU	1.092	0.801	0	0
7150	2099243	cobS, cobU	1.232	1.165	0	0
9485	2099406	cobS, cobU	1.321	1.036	0	0
1520	2099664	cobU, cbiP	0.752	0.807	0	0
20884	2099936	cobU, cbiP	1.072	1.176	0	0
6617	2100068	cobU, cbiP	1.140	0.859	0	0
8395	2100388	cbiP	1.435	0.755	0	0
10215	2100603	cbiP	1.165	0.825	0	0
2252	2100840	cbiP	1.306	0.793	0	0
12227	2101038	cbiP	1.379	0.953	0	0
17318	2101207	cbiP, cbiO	1.259	0.873	0	0
12751	2101391	cbiP, cbiO	1.164	0.758	0	0
8989	2101714	cbiO	1.142	0.780	0	0
634	2101946	cbiO	1.454	0.968	0	0
9298	2102021	cbiO, cboQ	0.913	0.800	0	0
20779	2102268	cbiO, cboQ	1.117	0.944	0	0
21263	2102684	cboQ, cbiN	1.375	0.947	0	0
329	2102938	cboQ, cbiN	1.181	0.865	0	0
6214	2103017	cboQ, cbiN	1.558	0.979	0	0
3927	2103408	cbiN, cbiM	1.093	0.825	0	0
13207	2103678	cbiM, cbiL	1.103	0.774	0	0
15853	2103867	cbiM, cbiL	0.648	0.639	0	0
2095	2104057	cbiM, cbiL	1.669	1.015	0	0
15046	2104217	cbiL	0.587	0.679	0	0
3408	2104383	cbiL	0.839	0.811	0	0
556	2104810	cbiL, cbiK	1.350	0.748	0	0
7394	2104884	cbiK	1.594	0.759	0	0
9378	2105161	cbiK	0.742	0.575	0	0
5057	2105308	cbiK, cbiJ	0.922	0.586	0	0
2281	2105656	cbiJ	1.571	0.754	0	0
21223	2105760	cbiJ	1.775	1.009	0	0
10682	2106020	cbiJ, cbiH	0.836	0.546	0	0
5992	2106246	cbiJ, cbiH	0.944	0.665	0	0
3378	2106385	cbiJ, cbiH	1.216	1.559	0	0
535	2106701	cbiH, cbiG	1.753	0.798	0	0

11462	2106834	cbiH, cbiG	1.329	0.672	0	0
17374	2107185	cbiG	0.888	0.690	0	0
10980	2107267	cbiG	1.217	0.672	0	0
10323	2107750	cbiG, cbiF	1.689	0.747	0	0
6996	2107825	cbiG, cbiF	1.561	0.788	0	0
9835	2108094	cbiG, cbiF	1.407	0.904	0	0
12401	2108377	cbiF	0.794	0.782	0	0
13412	2108508	cbiF, cbiT	1.456	0.842	0	0
3354	2108836	cbiF, cbiT	1.308	0.723	0	0
21171	2109075	cbiT, cbiE	1.177	0.832	0	0
7046	2109325	cbiT, cbiE	0.567	0.499	0	0
21401	2109399	cbiT, cbiE	1.590	0.853	0	0
10371	2109787	cbiE, cbiD	1.455	0.910	0	0
4445	2109863	cbiE, cbiD	0.943	0.749	0	0
2575	2110014	cbiE, cbiD	1.172	0.964	0	0
5537	2110274	cbiD	1.111	0.781	0	0
21602	2110504	cbiD	1.519	0.921	0	0
21212	2110803	cbiD, cbiC	1.531	0.771	0	0
6736	2111144	cbiD, cbiC	0.827	0.628	0	0
10143	2111369	cbiC	1.740	0.741	0	0
22069	2111516	cbiC, cibB	1.844	0.974	0	0
21183	2111837	cbiC, cibB	1.442	0.883	0	0
5496	2112044	cibB	1.518	0.852	0	0
2956	2112281	cibB	1.109	0.796	0	0
20311	2112374	cibB	0.927	0.666	0	0
18466	2112694	cibB, cbiA	1.263	0.841	0	0
21600	2112989	cbiA	1.491	0.920	0	0
9312	2113164	cbiA	1.380	0.837	0	0
2079	2113258	cbiA	1.062	0.593	0	0
13402	2113558	cbiA	1.206	0.720	0	0
2124	2113806	cbiA	0.974	0.609	0	0
19892	2114018	cbiA	1.981	0.647	0	0
4403	2114220	cbiA	2.186	0.426	0	0
13954	2114583	pocR	2.795	0.576	0	0
4529	2114800	pocR	2.030	0.601	0	0
16463	2114933	pocR	2.229	0.671	0	0

16002	2115039	pocR	2.344	0.694	1	0
17565	2115429	pocR	2.329	0.540	1	0
18288	2115504	pocR, pduF	2.392	0.511	1	0
13888	2115820	pduF	1.351	0.691	0	0
10734	2115960	pduF	1.477	0.758	0	0
13607	2116149	pduF	1.903	0.674	0	0
8284	2116553	pduF, pduA	3.334	0.405	1	0
19490	2116771	pduA, pudB	3.674	0.515	1	0
14438	2116842	pduA, pudB	4.081	0.490	1	0
2647	2117083	pduA, pudB	2.413	0.528	1	0
9147	2117210	pudB	1.912	0.549	0	0
16805	2117318	pudB	0.959	0.485	0	0
4221	2117923	pduC	1.285	0.493	0	0
16513	2117994	pduC	1.270	0.820	0	0
8773	2118277	pduC	1.378	0.692	0	0
13413	2118512	pduC	0.530	0.370	0	0
11226	2118908	pduC	1.648	0.681	0	0
16393	2119083	pduC	1.675	0.728	0	0
13187	2119309	pduC, pduD	1.685	0.677	0	0
22100	2119593	pduD	1.908	0.581	0	0
21120	2119664	pduD	1.881	0.722	0	0
7818	2119874	pduD, pduE	2.103	0.648	0	0
10060	2120213	pduD, pduE	1.520	0.623	0	0
1702	2120304	pduE	1.423	0.538	0	0
4449	2120669	pduE, pduG	1.577	0.636	0	0
15661	2120841	pduG	1.447	0.640	0	0
1388	2121106	pduG	0.930	0.706	0	0
12550	2121243	pduG	1.249	0.715	0	0
17620	2121464	pduG	0.855	0.645	0	0
4740	2121823	pduG	1.412	0.629	0	0
9600	2121940	pduG	1.777	0.661	0	0
2348	2122204	pduG, pduH	1.914	0.729	0	0
17562	2122391	pduG, pduH	1.779	0.718	0	0
17739	2122757	pduH, pduJ	1.255	0.629	0	0
9592	2122989	pduH, pduJ	1.904	0.768	0	0
1298	2123107	pduJ, pduK	1.370	0.749	0	0

4522	2123315	pduK, pduL	0.535	0.225	0	0
9620	2123614	pduK, pduL	1.130	0.557	0	0
14763	2123795	pduL	1.885	0.687	0	0
13133	2123973	pduL, pduM	1.396	0.675	0	0
10002	2124324	pduL, pduM	1.858	0.789	0	0
19575	2124506	pduM, pduN	1.377	0.839	0	0
12354	2124672	pduM, pduN	0.414	0.488	0	0
12320	2124889	pduM, pduN	2.300	0.836	0	0
1069	2125179	pduO	2.221	0.738	0	0
20319	2125360	pduO	0.607	0.758	0	0
11556	2125635	pduO	1.543	0.626	0	0
19724	2125785	pduO, pduP	0.557	0.493	0	0
403	2125972	pduO, pduP	1.035	0.462	0	0
3854	2126196	pduP	1.718	0.610	0	0
22332	2126453	pduP	0.719	0.486	0	0
629	2126709	pduP	1.403	0.630	0	0
19679	2127009	pduP	0.662	0.449	0	0
19043	2127284	pduP, pduQ	0.801	0.546	0	0
5446	2127359	pduP, pduQ	0.826	0.456	0	0
1753	2127588	pduQ	1.282	0.635	0	0
17624	2127674	pduQ	0.829	0.340	0	0
8788	2128018	pduQ	2.000	0.827	0	0
16054	2128195	pduQ	1.436	0.683	0	0
9039	2128531	pduQ, pduS	2.072	0.771	0	0
878	2128727	pduS	0.799	0.572	0	0
22144	2128865	pduS	1.383	0.664	0	0
22335	2129086	pduS	0.783	0.605	0	0
21042	2129378	pduS	0.798	0.449	0	0
19199	2129649	pduS, pduT	0.846	0.488	0	0
15846	2129872	pduS, pduT	1.614	0.623	0	0
1671	2130073	pduT	1.697	0.569	0	0
11729	2130170	pduT, pduU	0.958	0.434	0	0
12776	2130628	pduU, pduV	0.976	0.552	0	0
18231	2130707	pduU, pduV	1.303	0.579	0	0
16780	2130962	pduU, pduV	1.163	0.573	0	0
6335	2131070	pduV, pduW	1.863	0.684	0	0

16142	2131408	pduV, pduW	1.960	0.678	0	0
20530	2131640	pduW	0.918	0.466	0	0
2910	2132023	pduW	1.232	0.642	0	0
16636	2132227	pduW, pduX	1.092	0.822	0	0
1132	2132487	pduW, pduX	1.808	0.924	0	0
21430	2132641	pduX	1.262	0.867	0	0
9957	2132767	pduX	0.574	0.487	0	0
16288	2133094	pduX	0.827	0.871	0	0
22305	2133232	pduX	0.730	1.258	0	0
15836	2133535	pduX	0.681	1.559	0	0
19703	2133728	yeeX	0.790	1.719	0	0
18293	2133990	yeeX	0.906	1.903	0	0
17591	2134099	yeeX	0.850	1.552	0	0
11023	2134246	yeeX, yeeA	0.884	1.403	0	0
18844	2134565	yeeA	0.687	0.820	0	0
10182	2134811	yeeA	0.680	0.921	0	0
8333	2135218	yeeA	0.979	1.369	0	0
15248	2135294	yeeA	1.095	1.410	0	0
19438	2135427	yeeA, sbmC	0.982	1.400	0	0
9715	2135909	sbmC	0.842	0.972	0	0
14384	2136022	sbmC, dacD	0.891	0.963	0	0
12917	2136174	sbmC, dacD	0.911	0.963	0	0
18323	2136481	dacD	0.620	0.533	0	0
2855	2136675	dacD	1.248	0.931	0	0
21083	2137011	dacD	1.163	0.829	0	0
2301	2137226	dacD	1.472	0.821	0	0
9967	2137404	dacD, phsC	1.049	0.806	0	0
1655	2137557	dacD, phsC	0.909	0.673	0	0
20760	2137818	phsC	1.290	0.806	0	0
17817	2138069	phsC, phsB	1.338	0.829	0	0
17737	2138378	phsC, phsB	0.997	0.671	0	0
13128	2138616	phsB	1.347	0.758	0	0
20889	2138866	phsB, phsA	1.140	0.899	0	0
7336	2139002	phsB, phsA	0.796	0.549	0	0
7369	2139341	phsA	0.995	0.650	0	0
21800	2139551	phsA	0.618	0.621	0	0

6915	2139823	phsA	1.840	0.757	0	0
7907	2139931	phsA	1.585	0.725	0	0
7092	2140073	phsA	1.444	0.815	0	0
566	2140289	phsA	1.558	0.858	0	0
16826	2140445	phsA	1.533	0.667	0	0
6243	2140908	phsA	2.976	0.512	1	0
516	2141088	phsA, sopA	3.281	0.474	1	0
13901	2141369	phsA, sopA	2.962	0.578	1	0
6194	2141484	sopA	2.371	0.524	0	0
16063	2141725	sopA	2.481	0.503	0	0
13202	2141908	sopA	2.548	0.588	0	0
18640	2142176	sopA	3.646	0.528	1	0
2745	2142529	sopA	4.633	0.657	1	0
12296	2142661	sopA	2.598	0.529	0	0
20358	2142958	sopA	0.919	0.849	0	0
14850	2143206	sopA	0.850	0.682	0	0
11323	2143301	sopA	1.363	0.856	0	0
16681	2143387	sopA	1.145	0.874	0	0
21601	2143842	sopA, sbcB	1.725	1.327	0	0
1800	2144054	sbcB	1.865	0.881	0	0
16436	2144242	sbcB	1.674	0.896	0	0
6196	2144579	sbcB	1.415	0.972	0	0
7842	2144749	sbcB	1.132	0.880	0	0
2598	2144853	sbcB	1.047	0.846	0	0
13182	2145036	sbcB	1.227	0.944	0	0
3430	2145325	sbcB	0.627	0.566	0	0
10986	2145678		1.334	0.946	0	0
15084	2145909	yeeF	0.785	0.715	0	0
648	2145985	yeeF	1.154	1.212	0	0
10771	2146379	yeeF	1.203	0.995	0	0
6221	2146531	yeeF	0.998	1.167	0	0
18020	2146682	yeeF	0.437	0.700	0	0
6096	2147027	yeeF	0.836	1.136	0	0
5636	2147135	yeeF	1.036	1.163	0	0
5344	2147243	yeeF	0.872	1.260	0	0
16492	2147706	yeeY	1.099	1.100	0	0

6840	2147802	yeeY	1.105	1.049	0	0
16854	2148074	yeeY	0.633	0.796	0	0
15719	2148338	yeeY	1.079	1.085	0	0
20927	2148442	yeeY, yeeZ	1.353	1.276	0	0
15129	2148899	yeeZ	1.120	1.232	0	0
12185	2149044	yeeZ	0.796	1.327	0	0
22200	2149213	yeeZ, hisG	0.640	1.721	0	1
20144	2149587	yeeZ, hisG	0.692	1.906	0	1
7201	2149769	hisG	0.601	1.350	0	0
2321	2149842	hisG	0.894	1.475	0	0
6388	2150017	hisG	0.994	1.086	0	0
9608	2150406	hisG, hisD	0.875	0.635	0	0
8092	2150593	hisD	1.410	1.125	0	0
4934	2150690	hisD	1.391	0.984	0	0
8148	2150889	hisD	0.952	0.755	0	0
7260	2151108	hisD	0.880	0.699	0	0
2949	2151526	hisD, hisC	1.362	0.746	0	0
8515	2151692	hisD, hisC	1.206	0.760	0	0
1453	2151905	hisC	1.219	0.718	0	0
8843	2152207	hisC	0.999	0.749	0	0
7812	2152357	hisC	1.542	0.928	0	0
17245	2152597	hisC, hisB	0.851	0.671	0	0
16352	2152861	hisC, hisB	1.192	0.647	0	0
14838	2153097	hisB	1.312	0.971	0	0
10356	2153408	hisB	0.973	0.735	0	0
698	2153638	hisB, hisH	1.446	0.882	0	0
7611	2153901	hisB, hisH	1.119	0.847	0	0
13472	2154025	hisB, hisH	1.116	0.823	0	0
14524	2154234	hisH, hisA	0.885	0.602	0	0
12144	2154335	hisH, hisA	1.408	0.963	0	0
16518	2154616	hisH, hisA	1.384	0.830	0	0
13303	2154809	hisA	1.377	1.021	0	0
5295	2155110	hisA, hisF	0.946	0.800	0	0
20667	2155367	hisF	0.707	0.709	0	0
9843	2155687	hisF, hisI	0.418	0.525	0	0
14581	2155758	hisF, hisI	0.622	0.686	0	0

10086	2156060	hisF, hisI	1.181	1.054	0	0
2962	2156285	hisI	1.063	1.000	0	0
19596	2156461	hisI	0.842	0.800	0	0
22042	2156619	hisI	1.037	1.293	0	0
398	2156973	wzzB	0.852	1.710	0	0
14610	2157281	wzzB	0.912	1.191	0	0
19296	2157439	wzzB	1.117	1.481	0	0
16775	2157721	wzzB	1.212	1.225	0	0
5011	2157800	wzzB	1.422	1.082	0	0
17119	2158117	wzzB, udg	2.023	0.902	0	0
841	2158425	udg	2.653	0.890	1	0
17496	2158674	udg	2.431	0.855	1	0
8289	2158806	udg	2.113	0.621	0	0
13606	2159040	udg	2.149	0.746	0	0
12988	2159361	udg, gnd	2.303	0.642	0	0
4653	2159599	gnd	2.112	0.960	0	0
20700	2159679	gnd	1.826	0.900	0	0
10739	2159887	gnd	1.182	0.930	0	0
5644	2160175	gnd	1.109	0.972	0	0
13775	2160435	gnd	1.226	0.945	0	0
13301	2160548	gnd	1.329	0.907	0	0
21766	2160813	gnd	1.932	0.675	0	0
8983	2161003	gnd, rfbP	2.170	0.712	0	0
14018	2161204	gnd, rfbP	1.760	0.599	0	0
12844	2161319	rfbP	2.169	0.572	0	0
17789	2161666	rfbP	2.404	0.573	0	0
4837	2161968	rfbP	2.294	0.612	0	0
16499	2162053	rfbP	2.364	0.527	0	0
11781	2162432	rfbP, rfbK	2.214	0.481	0	0
6586	2162707	rfbP, rfbK	2.001	0.497	0	0
11122	2162812	rfbK	2.146	0.568	0	0
8730	2163204	rfbK	2.292	0.610	0	0
11418	2163442	rfbK	2.045	0.469	0	0
11230	2163664	rfbK	2.609	0.576	1	0
21462	2163867	rfbK, rfbM	2.475	0.691	1	0
10713	2164012	rfbK, rfbM	2.252	0.646	0	0

21761	2164310	rfbM	2.350	0.538	0	0
4940	2164425	rfbM	2.273	0.623	0	0
2123	2164512	rfbM	2.255	0.560	0	0
18158	2164893	rfbM	2.291	0.554	0	0
2358	2165116	rfbM	2.238	0.624	0	0
4965	2165358	rfbM, rfbN	2.144	0.544	0	0
8819	2165446	rfbM, rfbN	2.301	0.651	0	0
20996	2165693	rfbM, rfbN	2.308	0.643	0	0
10328	2165838	rfbN	1.986	0.519	0	0
18801	2165959	rfbN	2.141	0.601	0	0
4039	2166583	rfbN, rfbU	1.933	0.546	0	0
8924	2166793	rfbU	1.443	0.549	0	0
8543	2166949	rfbU	1.238	0.491	0	0
1833	2167208	rfbU	1.746	0.509	0	0
11301	2167279	rfbU	1.793	0.501	0	0
16447	2167674	rfbU, rfbV	1.743	0.436	0	0
6484	2167763	rfbV	1.674	0.472	0	0
974	2168190	rfbV	1.882	0.622	0	0
2765	2168368	rfbV	2.125	0.591	0	0
14259	2168449	rfbV	1.969	0.575	0	0
18127	2168709	rfbV, rfbX	2.198	0.473	0	0
3002	2169137	rfbX	1.607	0.492	0	0
7237	2169314	rfbX	1.600	0.473	0	0
3563	2169519	rfbX	1.454	0.445	0	0
6157	2169705	rfbX	1.670	0.450	0	0
12513	2169858	rfbX	1.664	0.511	0	0
17417	2170056	rfbX, rfbJ	1.683	0.555	0	0
13928	2170315	rfbX, rfbJ	1.992	0.517	0	0
15515	2170665	rfbJ	2.204	0.604	0	0
6043	2170907	rfbJ, rfbH	2.192	0.610	0	0
21942	2171033	rfbJ, rfbH	2.060	0.588	0	0
3192	2171173	rfbJ, rfbH	1.565	0.537	0	0
8074	2171397	rfbH	1.713	0.710	0	0
16939	2171644	rfbH	1.531	0.746	0	0
13049	2171988	rfbH	1.927	0.782	0	0
5696	2172271	rfbH, rfbG	1.849	0.835	0	0

5337	2172420	rfbH, rfbG	1.764	0.962	0	0
2125	2172491	rfbH, rfbG	1.734	0.919	0	0
4122	2172964	rfbG	1.160	0.805	0	0
231	2173127	rfbG	1.056	0.822	0	0
2992	2173298	rfbG	1.009	0.673	0	0
19989	2173472	rfbG, rfbF	0.806	0.629	0	0
21676	2173803	rfbF	1.297	0.662	0	0
12897	2173874	rfbF	1.394	0.724	0	0
15438	2174220	rfbF, rfbI	1.590	0.629	0	0
12098	2174599	rfbI	1.722	0.745	0	0
18906	2174765	rfbI	1.578	0.660	0	0
18674	2174902	rfbI	1.419	0.775	0	0
11728	2175193	rfbI, rfbC	1.236	0.676	0	0
6728	2175443	rfbI, rfbC	1.403	0.847	0	0
7599	2175549	rfbI, rfbC	1.422	0.775	0	0
18759	2175811	rfbC, rfbA	1.733	0.959	0	0
4618	2176037	rfbC, rfbA	1.498	0.875	0	0
1988	2176217	rfbA	1.358	0.913	0	0
21615	2176607	rfbA, rfbD	1.347	1.118	0	0
16252	2176801	rfbA, rfbD	1.008	1.152	0	0
10443	2176997	rfbD	0.911	1.270	0	0
2595	2177270	rfbD	0.631	0.937	0	0
17516	2177435	rfbD	0.771	1.108	0	0
1381	2177539	rfbD, rfbB	0.734	0.963	0	0
8091	2177707	rfbD, rfbB	0.847	0.984	0	0
21374	2177781	rfbD, rfbB	0.813	1.180	0	0
8786	2178411	rfbB	1.624	1.265	0	0
3422	2178499	rfbB	1.400	1.159	0	0
14665	2178826	rfbB	1.106	1.434	0	0
15626	2178954	rfbB, galF	0.873	1.411	0	0
2571	2179301	galF	0.795	1.498	0	0
1718	2179433	galF	0.864	1.749	0	0
7651	2179687	galF	0.710	0.997	0	0
7471	2179819	galF	0.809	0.920	0	0
6040	2180045	galF, wcaM	1.029	0.778	0	0
11258	2180418	wcaM	1.642	0.677	0	0

8935	2180518	wcaM	1.639	0.624	0	0
19528	2180749	wcaM	1.728	0.657	0	0
4723	2180911	wcaM	1.047	0.588	0	0
17863	2181360	wcaM	1.161	0.649	0	0
13523	2181458	wcaM, wcaL	0.440	0.521	0	0
14652	2181783	wcaM, wcaL	1.453	0.690	0	0
8791	2181912	wcaL	1.236	0.712	0	0
6379	2182169	wcaL	1.195	0.562	0	0
20516	2182529	wcaL	1.491	0.684	0	0
14887	2182605	wcaL	1.829	0.732	0	0
4473	2182981	wcaL, wcaK	1.574	0.588	0	0
5850	2183073	wcaL, wcaK	1.375	0.536	0	0
19520	2183439	wcaK	2.652	0.651	1	0
5462	2183643	wcaK	3.735	0.628	1	0
6439	2183882	wcaK	3.396	0.629	1	0
19846	2183966	wcaK, wzxC	3.945	0.609	1	0
19694	2184049	wcaK, wzxC	4.261	0.679	1	0
7941	2184452	wzxC	2.063	0.753	0	0
12994	2184547	wzxC	1.935	0.756	0	0
2162	2184723	wzxC	1.448	0.611	0	0
8528	2185143	wzxC	1.018	0.685	0	0
17381	2185411	wzxC	1.938	0.872	0	0
16984	2185617	wzxC, wcaJ	1.915	0.820	0	0
1090	2185749	wzxC, wcaJ	1.426	0.790	0	0
3394	2185943	wcaJ	1.524	0.672	0	0
16642	2186336	wcaJ	1.796	0.701	0	0
21484	2186570	wcaJ	1.003	1.323	0	0
18702	2186683	wcaJ	1.174	0.507	0	0
16588	2186922	wcaJ	1.211	0.592	0	0
17008	2187001	wcaJ, cpsG	1.050	0.540	0	0
17263	2187375	wcaJ, cpsG	2.118	0.889	0	0
20403	2187592	cpsG	1.347	0.829	0	0
7848	2187874	cpsG	0.891	0.501	0	0
16720	2188129	cpsG	0.574	0.457	0	0
22154	2188366	cpsG	2.402	0.863	0	0
6732	2188627	cpsG, manC	1.453	0.696	0	0

14719	2188731	cpsG, manC	0.761	0.582	0	0
5192	2188995	manC	1.102	0.537	0	0
8876	2189166	manC	1.643	0.776	0	0
20607	2189246	manC	1.496	0.724	0	0
16451	2189801	manC	1.698	0.577	0	0
20233	2189933	manC, wcaI	1.916	0.800	0	0
18545	2190151	manC, wcaI	0.487	0.600	0	0
4579	2190413	wcaI	2.041	0.735	0	0
12731	2190589	wcaI	0.719	0.634	0	0
11664	2190866	wcaI	1.932	0.640	0	0
16884	2191102	wcaI	1.594	0.782	0	0
6518	2191312	wcaI, wcaH	1.930	0.718	0	0
5114	2191608	wcaH, wcaG	1.338	0.615	0	0
1367	2191695	wcaH, wcaG	1.356	0.553	0	0
11603	2192057	wcaH, wcaG	1.230	0.606	0	0
13651	2192255	wcaG	1.692	0.584	0	0
12219	2192329	wcaG	1.603	0.639	0	0
18113	2192687	wcaG, gmd	1.827	0.860	0	0
17748	2192975	wcaG, gmd	0.696	0.482	0	0
2700	2193159	gmd	1.922	0.597	0	0
6324	2193311	gmd	0.775	0.395	0	0
17726	2193625	gmd	1.752	0.533	0	0
22358	2193709	gmd	3.108	1.494	1	0
10649	2194092	gmd, wcaF	5.134	0.625	1	0
16685	2194277	wcaF	5.019	0.653	1	0
16404	2194410	wcaF, wcaE	5.184	0.644	1	0
18222	2194607	wcaF, wcaE	1.165	0.470	0	0
1635	2194861	wcaE	3.028	0.598	1	0
6817	2195049	wcaE, wcaD	2.516	0.530	1	0
4380	2195340	wcaE, wcaD	3.664	0.485	1	0
9863	2195686	wcaD	3.492	0.477	1	0
14280	2195775	wcaD	3.285	0.570	1	0
6710	2195997	wcaD	2.157	0.502	0	0
20226	2196253	wcaD, wcaC	1.459	0.597	0	0
22290	2196581	wcaD, wcaC	1.753	0.616	0	0
13433	2196780	wcaC	1.658	0.577	0	0

9149	2197069	wcaC	1.127	0.548	0	0
16891	2197213	wcaC	1.572	0.678	0	0
13470	2197458	wcaC, wcaB	1.013	0.665	0	0
1640	2197560	wcaC, wcaB	1.384	0.651	0	0
8121	2197849	wcaC, wcaB	1.620	0.699	0	0
7396	2198116	wcaB, wcaA	2.049	0.724	0	0
18663	2198289	wcaB, wcaA	0.900	0.592	0	0
21841	2198679	wcaA	1.538	0.855	0	0
8026	2198768	wcaA	1.234	0.652	0	0
6443	2198995	wcaA, wzc	0.784	0.546	0	0
9431	2199340	wzc	1.711	0.714	0	0
6007	2199431	wzc	1.636	0.759	0	0
21288	2199736	wzc	1.888	0.705	0	0
19869	2199849	wzc	0.709	0.350	0	0
14122	2200047	wzc	1.877	0.659	0	0
16584	2200393	wzc	1.602	0.645	0	0
19970	2200606	wzc	1.106	0.689	0	0
3866	2200813	wzc	1.712	0.617	0	0
11029	2201003	wzc	1.113	0.700	0	0
16130	2201146	wzc, wzb	1.682	0.656	0	0
12560	2201458	wzc, wzb	0.517	0.519	0	0
14075	2201832	wzb, wza	1.297	0.507	0	0
12747	2202054	wza	2.032	0.655	0	0
9542	2202212	wza	2.828	0.657	1	0
5883	2202513	wza	5.777	0.673	1	0
18584	2202700	wza	5.436	0.531	1	0
429	2202883	wza, yegH	5.438	0.701	1	0
8455	2202983	wza, yegH	5.750	0.541	1	0
16305	2203279	yegH	4.154	0.582	1	0
18927	2203358	yegH	4.107	0.697	1	0
7684	2203851	yegH	1.014	0.686	0	0
15925	2204139	yegH	0.542	0.481	0	0
21852	2204308	yegH	1.077	0.764	0	0
3728	2204562	yegH	1.289	1.341	0	0
16910	2204812	yegH	1.107	1.888	0	0
5182	2204883		0.410	0.820	0	0

13531	2205071	asmA	0.863	1.462	0	0
6885	2205431	asmA	0.918	1.130	0	0
9331	2205574	asmA	1.188	1.766	0	0
16327	2205721	asmA	1.171	1.862	0	0
9152	2206026	asmA	1.068	1.812	0	0
1246	2206309	asmA	0.852	1.339	0	0
11835	2206507	asmA	0.728	1.257	0	0
15634	2206814	asmA	1.019	2.345	0	1
21208	2206885	asmA, dcd	0.921	1.842	0	1
5499	2207214	asmA, dcd	1.101	1.826	0	1
16677	2207494	dcd	0.996	1.438	0	0
16936	2207625	dcd, udk	1.185	1.457	0	0
8755	2207987	udk	0.880	1.428	0	0
21795	2208180	udk, yegE	0.776	1.027	0	0
15483	2208449	udk, yegE	1.084	1.687	0	0
3833	2208520	udk, yegE	0.783	1.428	0	0
2904	2208850	yegE	0.919	1.016	0	0
13457	2209045	yegE	0.998	0.954	0	0
7902	2209368	yegE	1.133	0.784	0	0
559	2209524	yegE	1.086	0.765	0	0
18054	2209706	yegE	1.384	0.954	0	0
12707	2209777	yegE	1.240	0.829	0	0
3670	2210158	yegE	1.395	0.951	0	0
7464	2210309	yegE	1.156	0.919	0	0
8605	2210614	yegE	0.843	0.640	0	0
1344	2210764	yegE	1.449	1.094	0	0
16196	2211094	yegE	0.915	1.009	0	0
22173	2211245	yegE	1.741	1.412	0	0
3936	2211393	yegE	1.452	1.335	0	0
7922	2211688	alkA	1.379	1.130	0	0
3561	2212008	alkA, yegD	0.781	0.817	0	0
4471	2212299	alkA, yegD	0.951	0.777	0	0
11307	2212463	alkA, yegD	1.135	0.729	0	0
20627	2212548	alkA, yegD	1.526	1.040	0	0
22034	2212771	alkA, yegD	1.456	0.837	0	0
12529	2212865	yegD	1.547	0.866	0	0

9617	2213376	yegD	1.298	0.938	0	0
22015	2213477	yegD	1.516	1.315	0	0
21320	2213862	STM2126	0.746	1.155	0	0
11665	2213947	STM2126	0.998	3.107	0	1
9995	2214205	STM2126	1.321	2.659	0	1
10018	2214276	STM2126	0.959	1.929	0	1
5780	2214728	STM2126	1.493	1.065	0	0
3958	2214940	STM2126	1.073	0.814	0	0
4228	2215146	STM2126, yegN	1.382	0.913	0	0
3170	2215309	STM2126, yegN	1.642	0.809	0	0
7592	2215476	STM2126, yegN	1.715	0.966	0	0
13508	2215891	yegN	0.985	0.789	0	0
4506	2215965	yegN	1.244	0.692	0	0
16180	2216242	yegN	1.623	0.803	0	0
16982	2216571	yegN	1.079	0.502	0	0
14296	2216660	yegN	1.139	0.616	0	0
14495	2216754	yegN	1.661	0.682	0	0
6546	2217174	yegN	1.540	0.820	0	0
7604	2217497	yegN	0.604	0.614	0	0
9894	2217728	yegN	1.326	0.766	0	0
21916	2217927	yegN	1.136	0.980	0	0
11354	2218150	yegN, yegO	1.492	0.876	0	0
7273	2218267	yegN, yegO	1.915	0.978	0	0
14987	2218531	yegN, yegO	1.274	0.724	0	0
12585	2218613	yegN, yegO	1.794	0.831	0	0
18262	2218891	yegO	0.972	0.570	0	0
3274	2219059	yegO	1.049	0.516	0	0
15730	2219336	yegO	0.969	0.620	0	0
7617	2219643	yegO	0.577	0.588	0	0
11808	2219818	yegO	0.769	0.739	0	0
21007	2220188	yegO	0.947	0.611	0	0
4093	2220421	yegO	1.228	0.579	0	0
10924	2220521	yegO	1.559	0.934	0	0
20632	2220850	yegO	1.116	0.789	0	0
14834	2220937	yegO	1.706	1.144	0	0
14813	2221234	yegO, yegB	1.097	0.950	0	0

2061	2221379	yegO, yegB	1.275	0.880	0	0
13543	2221690	yegO, yegB	1.484	0.997	0	0
5155	2221847	yegB	0.863	0.576	0	0
10028	2222192	yegB	1.095	0.633	0	0
8886	2222434	yegB	1.358	0.839	0	0
18599	2222578	yegB	1.129	0.836	0	0
6051	2222980	yegB, baeS	0.887	0.733	0	0
21612	2223085	yegB, baeS	1.126	0.936	0	0
2937	2223341	baeS	1.333	1.115	0	0
2343	2223561	baeS	0.927	0.709	0	0
14249	2223767	baeS	1.054	1.002	0	0
10635	2223960	baeS	1.470	1.273	0	0
5742	2224188	baeS, baeR	1.212	1.098	0	0
18414	2224506	baeS, baeR	1.382	1.283	0	0
7802	2224758	baeR	1.740	1.072	0	0
2489	2224933	baeR	2.004	0.741	0	0
11280	2225228	baeR	3.939	0.724	1	0
7268	2225409	STM2133	4.770	0.598	1	0
7425	2225646	STM2133	3.991	0.632	1	0
17188	2225792	STM2133	2.525	0.631	1	0
15737	2226066	STM2133	3.772	0.620	1	0
19818	2226226	STM2133	5.294	0.557	1	0
16210	2226448	STM2133, STM2134	3.736	0.590	1	0
6689	2226777	STM2134	2.273	0.612	0	0
5268	2226939	STM2134	2.364	0.739	0	0
14374	2227221	STM2134, STM2135	1.335	0.690	0	0
3186	2227407	STM2134, STM2135	2.134	1.021	0	0
21554	2227641	STM2135	1.878	0.718	0	0
4718	2227776	STM2135	1.396	0.701	0	0
21464	2228206	STM2135	2.203	0.956	0	0
19795	2228417	STM2135	1.868	0.875	0	0
3333	2228547	STM2135	1.839	0.944	0	0
7457	2228627	STM2135	1.749	0.974	0	0
15593	2229061	STM2135	1.653	0.830	0	0
22060	2229217	STM2135, yegQ	1.571	0.732	0	0
6015	2229435	STM2135, yegQ	0.665	0.732	0	0

2513	2229647	yegQ	0.806	0.545	0	0
12312	2229859	yegQ	1.068	0.950	0	0
7908	2230118	yegQ	1.454	0.795	0	0
20496	2230430	yegQ	1.198	0.662	0	0
1809	2230626	yegQ	1.003	0.661	0	0
309	2230823	yegQ	1.371	0.953	0	0
16820	2231007	yegQ, STM2137	1.437	1.273	0	0
9524	2231346	STM2137	1.940	0.779	0	0
8335	2231556	STM2137	1.973	0.560	0	0
3005	2231805	STM2137	2.092	0.538	0	0
21398	2231949	STM2137	2.137	0.540	0	0
7535	2232050	STM2137	2.049	0.500	0	0
9115	2232514		2.064	0.487	0	0
16347	2232652	STM2138	2.098	0.416	0	0
19849	2232882	STM2138	2.449	0.373	0	0
19711	2233014	STM2138	2.198	0.448	0	0
14615	2233424	STM2138, STM2139	2.143	0.552	0	0
695	2233601	STM2139, yegS	1.997	0.568	0	0
20087	2233876	STM2139, yegS	2.032	0.639	0	0
10367	2233955	yegS	2.144	0.639	0	0
17837	2234082	yegS	1.780	0.720	0	0
8204	2234372	yegS	1.587	0.742	0	0
656	2234795	yegS	1.335	0.939	0	0
20249	2234940	fbaB	1.355	0.957	0	0
19675	2235011	fbaB	0.716	0.705	0	0
14441	2235389	fbaB	1.021	0.699	0	0
10766	2235584	fbaB	1.089	0.613	0	0
11303	2235912	fbaB, yegT	1.239	0.705	0	0
14370	2236139	fbaB, yegT	1.628	0.657	0	0
20191	2236267	fbaB, yegT	0.835	0.558	0	0
9683	2236528	yegT	0.835	0.566	0	0
8688	2236714	yegT	1.089	0.634	0	0
17093	2236944	yegT	1.891	0.601	0	0
2610	2237055	yegT, yegU	1.692	0.584	0	0
17683	2237479	yegT, yegU	1.365	0.690	0	0
3635	2237754	yegU	1.218	0.864	0	0

11525	2237825	yegU	1.587	0.979	0	0
15081	2237975	yegU	0.979	0.812	0	0
16870	2238438	yegU, yegV	1.649	0.865	0	0
19549	2238511	yegU, yegV	1.798	1.050	0	0
14784	2238799	yegV	1.799	0.983	0	0
7893	2239046	yegV	1.875	1.497	0	0
7723	2239251	yegV	1.474	1.484	0	0
21050	2239482	yegW	1.213	1.365	0	0
16162	2239752	yegW	1.780	1.556	0	0
5752	2239841	yegW	1.248	1.277	0	0
10996	2240209	yegW	1.544	1.265	0	0
20253	2240339	yegW, thiD	0.759	0.825	0	0
15058	2240514	yegW, thiD	0.910	0.795	0	0
5475	2240827	thiD	1.853	0.819	0	0
16586	2241045	thiD, thiM	1.418	0.845	0	0
8414	2241302	thiD, thiM	1.247	0.826	0	0
10863	2241595	thiM	1.623	1.287	0	0
12719	2241718	thiM	1.514	1.737	0	0
12411	2242050	thiM, STM2148	1.471	1.231	0	0
17180	2242213	thiM, STM2148	1.622	1.090	0	0
19882	2242492	STM2148	2.537	0.747	0	0
18416	2242675		2.254	0.549	0	0
8676	2242953	stcD	3.867	0.536	1	0
11347	2243120	stcD	3.477	0.571	1	0
10066	2243417	stcD	2.707	0.624	1	0
3107	2243623	stcD	2.590	0.548	0	0
3120	2243886	stcD, stcC	2.433	0.482	0	0
3933	2243960	stcD, stcC	2.418	0.489	0	0
969	2244161	stcD, stcC	2.127	0.544	1	0
9439	2244566	stcC	2.144	0.569	1	0
5616	2244649	stcC	2.289	0.599	0	0
13787	2244838	stcC	2.176	0.463	0	0
3979	2245063	stcC	1.638	0.450	0	0
20140	2245459	stcC	1.607	0.588	0	0
17325	2245590	stcC	1.663	0.624	0	0
17970	2245771	stcC	1.894	0.477	0	0

8953	2246049	stcC	2.551	0.524	1	0
20646	2246302	stcC	2.770	0.465	1	0
12476	2246545	stcC, stcB	2.516	0.413	1	0
10811	2246698	stcC, stcB	2.447	0.385	0	0
13711	2246932	stcB	2.766	0.500	0	0
16844	2247253	stcB, stcA	2.507	0.555	0	0
12253	2247482	stcA	2.000	0.587	0	0
17015	2247672	stcA	3.014	0.532	0	0
14114	2247881	stcA, yehE	2.134	0.515	0	0
2050	2248118	yehE	3.597	0.660	1	0
5426	2248233	yehE	3.779	0.540	1	0
5871	2248412	yehE	3.381	0.717	0	0
5242	2248696	mrp	1.962	0.841	0	0
14372	2248767	mrp	1.375	0.778	0	0
8777	2249300	mrp, metG	1.039	1.239	0	0
836	2249571	mrp, metG	1.070	1.576	0	0
13199	2249699	mrp, metG	1.161	1.849	0	0
228	2249927	mrp, metG	1.262	1.737	0	0
19719	2250089	metG	1.109	1.390	0	0
5703	2250286	metG	1.151	1.210	0	0
12977	2250560	metG	1.063	1.163	0	0
20184	2250808	metG	0.960	1.252	0	0
20723	2251018	metG	1.096	1.364	0	0
12810	2251232	metG	1.103	1.261	0	0
6178	2251328	metG	1.305	1.363	0	0
11977	2251812	yehR	1.411	0.595	0	0
14504	2252050	yehR	2.034	0.619	0	0
11554	2252277	yehR, STM2156A	2.112	0.668	0	0
11309	2252447	STM2156A	1.559	0.646	0	0
10199	2252717	STM2156A	1.604	0.702	0	0
3133	2252981	STM2156A	1.725	0.895	0	0
20039	2253056	STM2156A	1.644	0.941	0	0
2080	2253351	yehS	1.137	1.199	0	0
18764	2253493	yehS	1.040	1.344	0	0
4902	2253702	yehS	0.897	1.498	0	0
17747	2253832	yehS, yehT	0.941	1.275	0	0

20824	2254143	yehT	1.019	1.113	0	0
1709	2254451	yehT, yehU	1.215	1.177	0	0
4569	2254689	yehT, yehU	1.075	1.076	0	0
14461	2254808	yehT, yehU	1.070	1.254	0	0
19867	2255124	yehU	0.963	1.215	0	0
9371	2255313	yehU	0.983	1.108	0	0
18636	2255620	yehU	1.032	1.390	0	0
7056	2255919	yehU, yehV	0.871	1.438	0	0
16420	2256121	yehU, yehV	0.708	1.136	0	0
18400	2256266	yehU, yehV	0.899	1.215	0	0
9275	2256367	yehU, yehV	0.800	1.156	0	0
16738	2256622	yehV	0.549	0.617	0	0
3442	2257069	yehV, STM2161	0.893	1.762	0	1
22014	2257140	STM2161	1.047	1.810	0	1
5107	2257331	yehW	1.069	2.444	0	1
21906	2257597	yehW	0.880	2.012	0	1
15430	2257917	yehW	0.885	0.880	0	0
3493	2258127	yehW, yehX	1.211	0.988	0	0
19825	2258216	yehW, yehX	1.011	0.924	0	0
9451	2258546	yehX	1.010	0.856	0	0
16718	2258875	yehX, yehY	1.729	1.082	0	0
16510	2258951	yehX, yehY	1.383	1.100	0	0
9564	2259363	yehY	0.463	0.672	0	0
7269	2259541	yehY	1.287	1.003	0	0
6409	2259799	yehY	0.965	1.014	0	0
10248	2259928	yehY	0.909	0.808	0	0
2750	2260047	yehY, yehZ	0.523	0.452	0	0
16099	2260410	yehY, yehZ	1.432	0.869	0	0
11078	2260656	yehZ	1.255	0.937	0	0
18753	2260854	yehZ	1.493	0.774	0	0
9944	2260976	yehZ	1.164	0.918	0	0
8192	2261249	yehZ, bglX	1.222	0.919	0	0
12345	2261561	bglX	1.092	0.864	0	0
4327	2261665	bglX	1.314	1.106	0	0
1180	2262029	bglX	1.436	1.092	0	0
14062	2262259	bglX	0.791	0.923	0	0

12961	2262371	bglX	0.502	0.410	0	0
16065	2262757	bglX	0.970	0.979	0	0
9539	2262891	bglX	1.164	0.954	0	0
8362	2263034	bglX	1.181	1.071	0	0
12734	2263295	bglX, dld	1.197	1.460	0	0
22008	2263445	bglX, dld	1.142	1.452	0	0
21122	2263713	bglX, dld	1.021	0.885	0	0
16422	2264012	dld	1.054	0.799	0	0
2939	2264337	dld	1.129	0.665	0	0
4835	2264529	dld	1.704	0.826	0	0
14351	2264709	dld	1.490	0.812	0	0
966	2264873	dld	1.694	0.957	0	0
11355	2265192	dld	1.134	0.996	0	0
7637	2265388	dld	1.188	1.639	0	0
8893	2265584	pbpG	1.108	1.433	0	0
20284	2265780	pbpG	1.155	1.193	0	0
5844	2266015	pbpG	1.473	1.245	0	0
5039	2266292	pbpG	1.029	1.213	0	0
15760	2266439	pbpG	1.070	1.362	0	0
998	2266707	pbpG, yohC	1.385	1.051	0	0
12475	2267034	yohC, yohD	1.036	0.720	0	0
8013	2267117	yohC, yohD	0.862	0.669	0	0
16629	2267272	yohC, yohD	1.133	0.829	0	0
13810	2267353	yohC, yohD	1.334	0.857	0	0
14889	2267533	yohC, yohD	0.864	0.936	0	0
4545	2268138	yohF	1.472	1.122	0	0
13117	2268330	yohF	1.056	0.835	0	0
12497	2268667	yohF	1.128	0.864	0	0
10987	2268785	yohF	1.626	0.868	0	0
3364	2269080	yohF, yohG	0.648	0.541	0	0
16613	2269245	yohG	1.485	0.817	0	0
21580	2269553	yohG	1.464	0.882	0	0
16185	2269635	yohG	0.800	0.595	0	0
16062	2269788	yohG	0.697	0.683	0	0
4657	2270226	yohG, STM2173	2.451	0.713	0	0
11098	2270297	yohG, STM2173	2.288	0.593	0	0

2205	2270589	yohG, STM2173	1.288	0.586	0	0
7428	2270784	yohI	2.457	0.693	1	0
21987	2270861	yohI	3.052	1.010	1	0
1018	2271203	yohI	0.877	0.559	0	0
17137	2271560	yohI	1.348	1.264	0	0
14837	2271667	yohI	1.437	1.113	0	0
8505	2271779	yohI, STM2175	1.192	0.892	0	0
14376	2272206	STM2175	0.843	0.789	0	0
11616	2272361	STM2175	1.503	0.847	0	0
9954	2272693	STM2175	1.448	0.773	0	0
2351	2272949	STM2175, STM2176	1.432	0.546	0	0
4916	2273161	STM2175, STM2176	1.306	0.731	0	0
18191	2273284	STM2175, STM2176	1.328	0.562	0	0
3294	2273591	STM2176	1.739	0.697	0	0
3287	2273726	STM2176, STM2177	1.761	0.655	0	0
1354	2274037	STM2176, STM2177	0.699	0.619	0	0
20225	2274317	STM2177	1.599	0.673	0	0
1734	2274461	STM2177, STM2178	1.331	0.609	0	0
14250	2274641	STM2177, STM2178	1.206	0.721	0	0
2608	2274845	STM2178	0.883	0.758	0	0
872	2275192	STM2178	1.236	0.534	0	0
14342	2275352	STM2178	2.058	0.725	0	0
19877	2275498	STM2178, STM2179	1.774	0.696	0	0
19799	2275912	STM2179	1.749	0.729	0	0
15190	2276022	STM2179	1.283	0.569	0	0
5303	2276343	STM2179	1.343	0.815	0	0
19631	2276578	STM2179, STM2180	1.089	0.780	0	0
2001	2276809	STM2179, STM2180	1.049	0.774	0	0
20133	2277007	STM2179, STM2180	1.029	0.815	0	0
1330	2277228	STM2180	1.075	1.103	0	0
9119	2277481	STM2180, yohJ	0.888	0.896	0	0
11829	2277729	STM2180, yohJ	1.161	0.991	0	0
9271	2277813	yohJ	0.668	0.491	0	0
9214	2277891	yohJ, yohK	1.073	0.753	0	0
18105	2278269	yohJ, yohK	0.999	0.606	0	0
1096	2278424	yohK	1.030	0.856	0	0

16100	2278579	yohK	1.012	0.751	0	0
11225	2279061	cdd	1.300	0.876	0	0
5899	2279158	cdd	0.803	0.623	0	0
13561	2279239	cdd	0.775	0.778	0	0
20829	2279664	cdd	1.133	0.943	0	0
8521	2279736	cdd, sanA	1.157	0.982	0	0
2062	2280174	sanA	0.861	2.178	0	1
16643	2280382	sanA	0.859	1.903	0	1
3622	2280600	sanA	1.223	1.602	0	0
3935	2280904	sanA, STM2186	2.795	1.066	0	0
10760	2281087	STM2186	2.468	0.794	0	0
15245	2281246	STM2186	2.555	0.754	1	0
10912	2281511	STM2186	3.492	0.801	1	0
4046	2281701	STM2186	2.763	0.778	1	0
2207	2282056	STM2186	2.604	0.627	1	0
684	2282273	STM2186, yeiA	1.926	0.601	0	0
10166	2282529	STM2186, yeiA	3.458	0.734	1	0
12777	2282660	yeiA	2.986	0.762	1	0
13110	2282791	yeiA	2.210	0.779	1	0
21820	2283213	yeiA	0.729	0.805	0	0
7805	2283284	yeiA	1.386	0.929	0	0
6923	2283456	yeiA	0.837	0.804	0	0
11082	2283805	yeiA	1.091	1.748	0	1
6298	2283899		0.874	1.838	0	1
14908	2284307	mglC	0.575	1.014	0	0
1183	2284480	mglC	0.908	1.464	0	0
14839	2284798	mglC	1.528	1.367	0	0
13535	2284912	mglC	1.595	1.398	0	0
18163	2285057	mglC, mglA	1.600	1.354	0	0
1842	2285314	mglC, mglA	1.561	1.308	0	0
9644	2285476	mglA	1.464	1.344	0	0
17605	2285889	mglA	1.195	1.357	0	0
9321	2286091	mglA	1.101	1.307	0	0
4401	2286166	mglA	1.047	1.482	0	0
767	2286346	mglA	1.164	1.823	0	1
8198	2286604	mglA, mglB	0.825	2.213	0	1

1797	2287017	mglB	0.516	1.869	0	1
12558	2287103	mglB	0.522	2.126	0	1
18573	2287356	mglB	0.610	2.164	0	1
13958	2287455	mglB	0.542	2.462	0	1
9481	2287976	mglB	0.616	2.191	0	1
5222	2288073		0.677	2.008	0	0
6648	2288294	galS	0.664	1.377	0	0
1717	2288393	galS	0.658	1.547	0	0
9810	2288756	galS	0.696	1.424	0	0
10220	2289119	galS	0.741	1.138	0	0
18203	2289246	galS	0.554	0.850	0	0
20321	2289445	galS, yeiB	0.950	1.013	0	0
16363	2289652	yeiB	0.394	0.634	0	0
3288	2289880	yeiB	0.511	0.763	0	0
947	2290101	yeiB	1.096	1.504	0	0
6566	2290395	yeiB	0.815	1.145	0	0
2858	2290677	yeiB, folE	1.011	1.152	0	0
8407	2290819	yeiB, folE	1.051	1.172	0	0
6764	2290923	folE	0.987	1.146	0	0
12011	2291252	folE, yeiG	1.095	1.306	0	0
1766	2291568	yeiG	1.165	2.035	0	0
7440	2291744	yeiG	0.441	0.805	0	0
9332	2291992	yeiG	1.071	1.756	0	0
16977	2292172		1.170	1.454	0	0
2921	2292492	STM2195	1.011	1.091	0	0
10007	2292626	STM2195	1.187	0.981	0	0
3976	2292835	STM2195, STM2196	1.053	0.669	0	0
20121	2293031	STM2195, STM2196	1.350	0.860	0	0
19406	2293384	STM2196	0.684	0.596	0	0
8502	2293625	STM2196	1.960	0.624	0	0
12981	2293896	STM2196, STM2197	3.583	0.585	1	0
11764	2294126	STM2196, STM2197	3.678	0.525	1	0
12527	2294266	STM2196, STM2197	4.463	0.561	1	0
3235	2294508	STM2197	2.098	0.587	0	0
4890	2294828	STM2197, STM2198	4.344	0.443	1	0
4546	2294936	STM2197, STM2198	3.805	0.560	1	0

12191	2295068	STM2197, STM2198	4.757	0.562	1	0
12528	2295283	STM2198	3.890	0.653	1	0
9403	2295361	STM2198	2.521	0.546	0	0
6691	2295900	STM2198	1.856	0.649	0	0
19535	2296116	STM2198	1.610	0.793	0	0
10552	2296212	STM2198	1.034	0.659	0	0
2617	2296572	cirA	1.542	0.830	0	0
21289	2296754	cirA	1.321	0.724	0	0
3189	2296924	cirA	1.182	0.687	0	0
2016	2297058	cirA	1.006	0.595	0	0
1890	2297171	cirA	1.310	0.751	0	0
21425	2297632	cirA	1.301	1.016	0	0
19272	2297777	cirA	0.921	0.777	0	0
15079	2298136	cirA	0.991	0.651	0	0
769	2298247	cirA	0.646	0.506	0	0
11201	2298514	cirA	1.058	0.713	0	0
14424	2298745	cirA	1.185	0.822	0	0
9416	2298940	cirA, lysP	0.706	0.640	0	0
7979	2299014	lysP	0.762	0.758	0	0
16378	2299571	lysP	0.917	0.875	0	0
1559	2299798	lysP	1.168	1.039	0	0
17678	2299908	lysP	1.129	1.195	0	0
9081	2300068	lysP	1.082	1.326	0	0
20699	2300328	lysP	1.167	1.297	0	0
14416	2300457	lysP	1.383	1.454	0	0
9177	2300697	lysP, yeiE	0.928	1.099	0	0
2574	2300947	yeiE	1.050	0.855	0	0
2537	2301378	yeiE, yeiH	1.185	0.622	0	0
20900	2301526	yeiE, yeiH	1.115	0.810	0	0
4745	2301673	yeiE, yeiH	1.278	0.823	0	0
1225	2301761	yeiE, yeiH	1.134	0.998	0	0
10311	2302298	yeiH, nfo	1.073	1.098	0	0
15742	2302525	yeiH, nfo	1.420	1.166	0	0
20912	2302615	nfo	1.344	0.940	0	0
12930	2302782	nfo	1.305	1.076	0	0
21124	2302963	nfo	1.251	0.994	0	0

2591	2303083	nfo	0.932	0.762	0	0
11926	2303246	nfo	1.430	0.945	0	0
6292	2303398	nfo	1.431	1.017	0	0
1006	2303561	fruA	0.919	0.870	0	0
10901	2304244	fruA	1.564	0.709	0	0
22055	2304480	fruA	1.999	0.918	0	0
16097	2304746	fruA	0.859	0.732	0	0
14381	2304857	fruA	0.874	0.565	0	0
8429	2305128	fruA	1.296	0.683	0	0
14001	2305262	fruA, fruK	1.454	0.660	0	0
1067	2305686	fruA, fruK	1.645	0.697	0	0
17575	2305891	fruK	1.427	0.667	0	0
18221	2305986	fruK	1.375	0.591	0	0
18100	2306379	fruK, fruF	1.434	0.697	0	0
10197	2306454	fruK, fruF	1.024	0.575	0	0
21702	2306625	fruK, fruF	1.225	0.610	0	0
15236	2306886	fruF	0.930	0.646	0	0
9334	2307156	fruF	0.711	0.461	0	0
4442	2307326	fruF, setB	0.523	0.415	0	0
3569	2307602	fruF, setB	0.981	0.623	0	0
21004	2307750	fruF, setB	0.753	0.592	0	0
1452	2308012	setB	1.068	0.574	0	0
14624	2308382	setB	1.161	0.560	0	0
14297	2308523	setB	1.439	0.659	0	0
15376	2308884		2.632	0.594	0	0
3381	2309039	STM2208	2.275	0.584	0	0
4479	2309150	STM2208	2.718	0.569	0	0
12474	2309477	STM2208	2.921	0.400	1	0
12204	2309609	STM2208, STM2209	2.630	0.484	1	0
19955	2309928	STM2208, STM2209	1.993	0.609	0	0
16674	2310140	STM2209, yeiP	1.717	0.751	0	0
21346	2310377	yeiP	1.037	0.736	0	0
1845	2310458	yeiP	0.796	0.726	0	0
8014	2310732	yeiP	0.957	0.847	0	0
14619	2310991	yeiP	1.065	0.939	0	0
21437	2311352	yeiR	1.009	0.743	0	0

3680	2311491	yeiR	1.193	0.840	0	0
7378	2311768	yeiR	0.860	0.645	0	0
18616	2312029	yeiR, yeiU	1.140	0.995	0	0
16971	2312211	yeiR, yeiU	0.825	0.995	0	0
10815	2312410	yeiR, yeiU	0.855	1.228	0	0
18365	2312606	yeiU	0.789	1.682	0	0
22037	2312974	yeiU	0.807	1.685	0	0
6525	2313215	spr	0.697	2.034	0	0
4956	2313360	spr	0.494	1.973	0	1
18725	2313627	spr	0.436	2.550	0	1
18381	2313720	spr	0.361	1.805	0	1
12682	2313793	spr	0.393	2.683	0	1
2677	2313962	spr	0.370	2.854	0	1
13216	2314453	rtn	0.619	2.014	0	1
5173	2314662	rtn	0.729	1.577	0	0
14435	2314798	rtn	0.795	1.444	0	0
6483	2315217	rtn	0.787	1.002	0	0
12438	2315347	rtn	0.806	1.278	0	0
15990	2315493	rtn	0.986	1.487	0	0
13929	2315812	rtn, yejA	0.998	1.169	0	0
18002	2316103	yejA	1.015	1.484	0	0
14812	2316308	yejA	0.911	1.366	0	0
16128	2316464	yejA	1.000	1.319	0	0
19885	2316761	yejA	0.951	1.022	0	0
19256	2316852	yejA	0.987	0.993	0	0
1932	2316931	yejA	1.076	1.133	0	0
4360	2317217	yejA	1.259	1.067	0	0
20801	2317580	yejA, yejB	1.262	1.364	0	0
19173	2317693	yejA, yejB	1.140	1.291	0	0
14175	2318061	yejB	1.162	1.412	0	0
22310	2318333	yejB	1.150	1.450	0	0
8520	2318661	yejB, yejE	1.043	1.044	0	0
964	2318907	yejB, yejE	1.023	1.041	0	0
18696	2319005	yejB, yejE	1.160	1.234	0	0
1504	2319195	yejE	1.046	1.262	0	0
16517	2319548	yejE	1.075	1.165	0	0

22256	2319681	yejE, yejF	0.609	0.820	0	0
12419	2319891	yejE, yejF	1.254	1.186	0	0
1598	2320144	yejF	1.039	1.118	0	0
16124	2320426	yejF	1.209	1.349	0	0
15535	2320523	yejF	0.766	0.947	0	0
14492	2320765	yejF	1.283	1.393	0	0
10581	2321017	yejF	0.794	0.954	0	0
13031	2321253	yejF	1.078	1.369	0	0
11935	2321558	yejF	0.838	1.987	0	1
11435	2321740	yejG	0.856	2.022	0	1
20450	2322063	yejG	0.925	2.149	0	1
12814	2322254	yejG	0.886	1.440	0	0
17166	2322338	yejG	1.054	1.197	0	0
4431	2322668	bcr	0.703	1.091	0	0
6912	2322849	bcr	1.066	1.057	0	0
13737	2323033	bcr	1.163	1.038	0	0
12762	2323250	bcr	0.933	0.907	0	0
11232	2323578	bcr	1.206	1.180	0	0
7414	2323880	bcr, rsuA	1.049	1.122	0	0
7654	2324128	rsuA, yejH	0.925	1.098	0	0
15145	2324244	rsuA, yejH	1.032	1.537	0	0
19468	2324503	rsuA, yejH	1.105	1.532	0	0
6509	2324788	rsuA, yejH	0.862	0.884	0	0
5773	2324917	yejH	0.773	0.817	0	0
1337	2325023	yejH	0.595	0.669	0	0
9008	2325453	yejH	0.668	0.863	0	0
18962	2325641	yejH	1.116	1.278	0	0
5318	2325822	yejH	1.170	1.191	0	0
16859	2325979	yejH, rplY	0.805	1.313	0	0
1499	2326369	rplY	0.929	1.948	0	0
21515	2326598	rplY	0.666	2.067	0	0
7588	2326770		0.615	1.445	0	0
10616	2327057	STM2225	0.543	0.834	0	0
20079	2327151	STM2225	0.667	0.843	0	0
529	2327301	STM2225	0.739	0.881	0	0
19787	2327737	STM2225, yejK	0.872	1.032	0	0

18851	2327969	yejK	0.861	1.170	0	0
19405	2328075	yejK	0.876	1.315	0	0
2412	2328442	yejK, yejL	0.643	1.063	0	0
608	2328626	yejK, yejL	0.704	1.992	0	0
2227	2328871	yejK, yejM	0.728	1.397	0	0
8882	2329057	yejM	0.681	1.667	0	0
12733	2329208	yejM	0.635	1.440	0	0
6868	2329330	yejM	0.814	1.438	0	0
9677	2329770	yejM	0.783	1.529	0	0
22269	2329906	yejM	0.761	1.525	0	0
9075	2330122	yejM	0.718	1.523	0	0
20334	2330445	yejM, tRNAPro2	0.453	1.806	0	0
18124	2330685	tRNAPro2	0.545	2.327	0	1
2980	2330911		0.645	2.149	0	1
3368	2331008	STM2230, STM2231	0.618	2.176	0	1
15759	2331352	STM2230, STM2231	0.891	1.124	0	0
17879	2331424	STM2230, STM2231	0.852	0.944	0	0
9731	2331836	STM2230, oafA	1.564	0.633	0	0
6339	2332003	oafA	1.712	0.506	0	0
20800	2332170	oafA	2.292	0.534	0	0
16881	2332495	oafA	2.059	0.393	0	0
9562	2332664	oafA	2.223	0.440	0	0
5514	2332882	oafA	2.439	0.685	0	0
11218	2333204	oafA	2.106	0.514	0	0
7208	2333387	oafA	2.197	0.491	0	0
4021	2333484	oafA	2.346	0.467	0	0
3554	2333908	oafA	1.928	0.573	0	0
13864	2334019	STM2233	2.135	0.628	0	0
18267	2334266	STM2233	2.864	0.647	1	0
6876	2334577	STM2233	2.720	0.625	1	0
9325	2334829	STM2233, STM2234	2.685	0.576	1	0
9817	2334940	STM2234	2.538	0.580	0	0
14115	2335095	STM2234	2.770	0.562	1	0
14608	2335427	STM2234, STM2235	2.242	0.577	1	0
14128	2335563	STM2234, STM2235	0.966	0.537	0	0
14430	2335716	STM2234, STM2235	1.367	0.598	0	0

3644	2335870	STM2235	0.648	0.478	0	0
12634	2336325	STM2235	1.138	0.573	0	0
21956	2336448	STM2235	0.891	0.640	0	0
3217	2336629	STM2235	1.030	0.515	0	0
7834	2337043	STM2235, STM2236	1.347	0.619	0	0
19844	2337166	STM2236	0.681	0.556	0	0
16395	2337470	STM2236, STM2237	1.054	0.665	0	0
7410	2337747	STM2237, STM2238	1.818	0.606	0	0
6494	2337921	STM2237, STM2238	1.896	0.495	0	0
4173	2338029	STM2238	2.147	0.525	0	0
7174	2338214	STM2238	2.701	0.548	0	0
16049	2338670	STM2238	2.335	0.427	0	0
19234	2338747	STM2238	2.421	0.430	0	0
18937	2339053	STM2238	2.988	0.506	1	0
5915	2339236	STM2239	2.814	0.548	1	0
7507	2339386	STM2239	2.985	0.625	1	0
7720	2339592	STM2239, STM2240	2.117	0.609	0	0
18232	2339939	STM2239, STM2240	1.375	0.756	0	0
16622	2340109	STM2240	1.249	0.804	0	0
11666	2340306	STM2240	0.587	0.670	0	0
9381	2340518	STM2240	1.232	0.661	0	0
13389	2340778	STM2240	1.144	0.722	0	0
14145	2341040	sspH2	0.976	0.540	0	0
8587	2341282	sspH2	0.979	0.621	0	0
20806	2341421	sspH2	1.623	0.870	0	0
5190	2341593	sspH2	1.198	0.704	0	0
12559	2341877	sspH2	1.380	0.744	0	0
12524	2342229	sspH2	0.775	0.658	0	0
18401	2342349	sspH2	1.026	0.758	0	0
8903	2342554	sspH2	1.345	1.214	0	0
10826	2342626	sspH2	0.589	0.749	0	0
16193	2342740	sspH2	0.721	0.785	0	0
15424	2342886	sspH2	1.487	1.169	0	0
18506	2343537	sspH2, STM2242	2.521	0.726	1	0
17407	2343865	STM2242	2.570	0.643	1	0
3898	2344037	STM2242, STM2243	2.119	0.650	0	0

3174	2344193	STM2243	1.645	0.519	0	0
10596	2344264	STM2243	1.965	0.674	0	0
14844	2344468	STM2243	1.582	0.642	0	0
16432	2344659	STM2243, STM2244	1.782	0.694	0	0
15889	2345057	STM2243, STM2244	2.460	0.449	1	0
6310	2345199		2.632	0.457	1	0
17192	2345521	STM2245	3.776	0.547	1	0
1645	2345878	STM2245	3.737	0.598	1	0
1151	2346118	STM2245	2.943	0.406	1	0
17680	2346377	STM2245, narP	3.657	0.548	1	0
7218	2346450	narP	3.477	0.547	1	0
14912	2346681	narP	2.365	0.695	1	0
3705	2346858	narP	1.060	0.553	0	0
20451	2347223	narP	1.627	1.229	0	0
15563	2347304	narP	1.435	0.914	0	0
5036	2347496		1.011	0.667	0	0
4099	2347921		0.486	0.829	0	0
7267	2348178		1.184	0.768	0	0
17481	2348447		1.378	0.793	0	0
2077	2348672		0.800	0.546	0	0
1855	2348761		1.350	0.747	0	0
20685	2348951		1.607	0.802	0	0
5142	2349050		1.094	0.804	0	0
20018	2349571		1.542	0.770	0	0
2646	2349712		1.432	0.715	0	0
5040	2349923		0.829	0.740	0	0
3909	2350166		0.777	0.745	0	0
9883	2350348		0.431	0.646	0	0
9151	2350684		2.792	1.054	0	0
18671	2350904		1.805	1.052	0	0
18335	2351097		1.244	0.905	0	0
4057	2351239		1.213	0.744	0	0
18549	2351568		2.361	1.095	0	0
20882	2351647		3.020	1.082	0	0
17576	2351798		1.497	0.995	0	0
15200	2352124		2.149	1.029	0	0

8128	2352313		1.214	0.806	0	0
9463	2352667		0.927	0.698	0	0
2107	2352934		1.436	0.700	0	0
16965	2353167		1.694	0.893	0	0
6930	2353401		1.100	0.668	0	0
16174	2353652		1.344	1.060	0	0
10644	2353903	napC	1.482	1.021	0	0
7670	2354094	napC	1.439	1.016	0	0
15612	2354242	napC	1.555	1.075	0	0
11528	2354555	napC, napB	1.189	1.142	0	0
18046	2354719	napC, napB	1.104	1.026	0	0
5839	2354872	napB, napH	1.118	0.856	0	0
5660	2355022	napB, napH	1.181	0.929	0	0
11167	2355204	napH	0.968	0.882	0	0
18426	2355504	napH	1.389	1.031	0	0
5568	2355713	napH, napG	0.728	0.783	0	0
18989	2355784	napH, napG	0.927	0.659	0	0
21786	2356378	napG	0.812	0.761	0	0
14392	2356482	napG, napA	0.983	0.870	0	0
13657	2356816	napG, napA	1.104	0.870	0	0
1849	2357005	napA	0.803	0.640	0	0
15232	2357155	napA	0.631	0.741	0	0
10015	2357447	napA	1.082	0.851	0	0
14886	2357559	napA	0.980	0.880	0	0
18393	2357870	napA	1.341	1.219	0	0
11401	2358039	napA	1.524	1.432	0	0
9558	2358443	napA	1.069	1.271	0	0
5595	2358611	napA	1.123	0.684	0	0
2198	2358869	napA	1.034	0.847	0	0
16656	2358961	napA, napD	0.859	0.767	0	0
15233	2359249	napA, napD	1.343	0.798	0	0
14032	2359422	napD, napF	0.888	0.842	0	0
4923	2359646	napF, eco	2.473	0.773	0	0
8033	2359717	napF, eco	2.153	0.582	0	0
16409	2360190	eco	1.597	0.736	0	0
6351	2360301	eco	0.764	0.549	0	0

2178	2360582		0.545	0.763	0	0
1723	2360887	yojI	1.390	1.603	0	0
22345	2361037	yojI	1.534	1.243	0	0
17645	2361174	yojI	1.585	1.150	0	0
2707	2361460	yojI	1.418	0.924	0	0
1282	2361727	yojI	1.393	0.948	0	0
17026	2361917	yojI	0.649	0.580	0	0
7406	2362189	yojI	1.439	1.100	0	0
9960	2362384	yojI	1.255	0.947	0	0
13669	2362478	yojI, alkB	1.095	0.691	0	0
15484	2362980	alkB	0.868	0.759	0	0
10201	2363109	alkB, ada	1.160	0.894	0	0
16015	2363357	alkB, ada	0.506	0.561	0	0
17140	2363614	ada	1.218	0.991	0	0
6038	2363806	ada	1.278	0.802	0	0
9582	2364029	ada	1.442	1.108	0	0
1560	2364190	ada	0.835	0.680	0	0
19709	2364299	ada, apbE	1.051	0.831	0	0
12769	2364772	apbE	1.132	0.903	0	0
6285	2364848	apbE	0.532	0.657	0	0
6124	2365099	apbE	0.898	0.885	0	0
22067	2365307	apbE	1.055	1.135	0	0
17047	2365469	apbE, ompC	0.647	1.040	0	0
1654	2365754	apbE, ompC	0.853	1.523	0	0
5465	2365885	ompC	0.664	1.495	0	0
11879	2366301	ompC	0.991	1.284	0	0
9722	2366543	ompC	1.406	1.201	0	0
20544	2366638	ompC	1.479	1.074	0	0
11298	2367027	micF, yojN	1.122	0.821	0	0
14810	2367293	micF, yojN	1.109	1.112	0	0
13838	2367436	micF, yojN	0.979	1.083	0	0
21178	2367731	yojN	0.798	1.048	0	0
2987	2367887	yojN	0.803	1.127	0	0
7213	2368197	yojN	0.839	0.967	0	0
13175	2368444	yojN	0.928	1.099	0	0
10209	2368596	yojN	1.079	1.127	0	0

11800	2368800	yojN	0.934	1.203	0	0
18423	2369080	yojN	0.719	0.935	0	0
604	2369152	yojN	1.168	1.441	0	0
18622	2369599	yojN, rcsB	0.870	1.288	0	0
19486	2369728	yojN, rcsB	0.765	1.186	0	0
6117	2369871	yojN, rcsB	0.847	1.267	0	0
19362	2370031	rcsB	0.589	1.178	0	0
19167	2370207	rcsB	0.650	1.423	0	0
14517	2370605	rcsB	0.717	1.665	0	0
19544	2370790	rcsC	0.570	1.235	0	0
5750	2371092	rcsC	0.855	1.301	0	0
16923	2371340	rcsC	0.995	1.242	0	0
2914	2371557	rcsC	0.986	1.059	0	0
7775	2371778	rcsC	1.099	1.015	0	0
3143	2372007	rcsC	1.093	0.994	0	0
14773	2372277	rcsC	1.127	1.069	0	0
3769	2372434	rcsC	0.916	0.927	0	0
2317	2372717	rcsC	1.194	1.066	0	0
14226	2372799	rcsC	1.104	1.006	0	0
12335	2373117	rcsC	0.918	0.941	0	0
277	2373349	rcsC	1.054	1.342	0	0
21426	2373473	rcsC	1.042	1.411	0	0
11753	2373617	rcsC	0.810	1.100	0	0
1396	2373935	rcsC, gyrA	0.859	1.516	0	0
22309	2374014	rcsC, gyrA	0.954	1.714	0	0
18818	2374403	gyrA	0.976	1.444	0	0
6412	2374711	gyrA	0.717	1.093	0	0
472	2374873	gyrA	0.783	1.504	0	0
11260	2375191	gyrA	1.051	1.635	0	0
20033	2375348	gyrA	0.410	0.607	0	0
21753	2375517	gyrA	1.064	1.371	0	0
14121	2375880	gyrA	0.790	1.224	0	0
9646	2376115	gyrA	0.611	1.629	0	1
4435	2376361	gyrA	1.001	2.000	0	1
8274	2376438	gyrA	1.044	1.800	0	0
12514	2376832	STM2273	2.465	0.698	1	0

9374	2377058	STM2273	2.579	0.593	1	0
14791	2377266	STM2273	3.008	0.642	1	0
18252	2377428	STM2273	3.147	0.607	1	0
15884	2377632	STM2273	3.115	0.592	1	0
3584	2377845	STM2273	2.810	0.445	1	0
5912	2378093	STM2273, STM2274	3.062	0.499	1	0
11371	2378390	STM2273, STM2274	2.513	0.467	1	0
11272	2378537	STM2274	2.475	0.528	1	0
450	2378706	STM2274	2.086	0.656	0	0
12119	2379127	STM2274, STM2275	2.611	0.451	0	0
5378	2379282	STM2274, STM2275	2.494	0.415	0	0
5388	2379599	STM2274, STM2275	2.427	0.504	0	0
3634	2379789	STM2275	2.502	0.538	0	0
1917	2379913	STM2275, ubiG	2.472	0.497	0	0
17314	2380093	STM2275, ubiG	2.290	0.592	0	0
21748	2380315	ubiG	1.900	0.847	0	0
19855	2380405	ubiG	1.515	0.983	0	0
7827	2380880	ubiG	0.950	1.001	0	0
8420	2381026	nrdA	0.876	0.996	0	0
16754	2381348	nrdA	0.664	1.093	0	0
883	2381518	nrdA	0.802	1.558	0	0
1986	2381710	nrdA	0.974	1.355	0	0
2558	2381960	nrdA	0.808	1.383	0	0
15527	2382052	nrdA	0.789	1.303	0	0
12426	2382488	nrdA	0.923	1.695	0	0
19346	2382620	nrdA	0.776	1.631	0	0
12627	2382779	nrdA	0.848	1.567	0	0
15590	2383001	nrdA	0.822	1.584	0	0
13131	2383446	nrdA, nrdB	0.841	1.516	0	0
4490	2383544	nrdA, nrdB	0.756	1.355	0	0
11200	2383787	nrdB	0.743	1.145	0	0
8424	2383875	nrdB	0.735	1.076	0	0
16091	2384327	nrdB	0.838	1.293	0	0
21371	2384442	nrdB, yfaE	0.976	1.350	0	0
13288	2384829	nrdB, yfaE	0.876	1.583	0	0
7512	2384901	yfaE	0.802	1.447	0	0

15926	2385160	STM2280	1.001	1.426	0	0
14629	2385277	STM2280	0.853	1.219	0	0
19907	2385574	STM2280	1.180	0.865	0	0
20128	2385794	STM2280	1.206	0.780	0	0
9376	2386124	STM2280, STM2281	1.034	0.655	0	0
517	2386337	STM2280, STM2281	1.086	0.819	0	0
2634	2386409	STM2280, STM2281	0.800	0.762	0	0
5872	2386515	STM2280, STM2281	0.668	0.826	0	0
21097	2386906	STM2281	0.852	1.634	0	0
14995	2387210	STM2281	0.802	2.005	0	1
18277	2387349	STM2281	0.628	1.952	0	1
4209	2387506	glpQ	0.480	2.149	0	1
9063	2387871	glpQ	0.513	2.307	0	1
11362	2388119	glpQ	0.538	2.260	0	1
10577	2388244	glpQ	0.567	2.363	0	1
18807	2388578	glpQ, glpT	0.393	2.552	0	1
9879	2388859	glpQ, glpT	0.393	3.125	0	1
8711	2389065	glpT	0.416	2.803	0	1
4074	2389244	glpT	0.376	2.718	0	1
15016	2389387	glpT	0.416	2.633	0	1
5262	2389693	glpT	0.447	3.207	0	1
6035	2389938	glpT, glpA	0.472	3.001	0	1
7251	2390216	glpT, glpA	0.582	1.932	0	1
10835	2390458	glpA	0.641	1.450	0	0
3703	2390604	glpA	0.740	1.265	0	0
15939	2390918	glpA	0.713	1.150	0	0
6977	2391048	glpA	0.776	1.130	0	0
13460	2391378	glpA, glpB	1.099	1.406	0	0
2836	2391560	glpA, glpB	0.884	1.373	0	0
10416	2391818	glpA, glpB	0.958	0.827	0	0
567	2391939	glpB	0.789	0.968	0	0
5673	2392212	glpB	0.833	0.929	0	0
22334	2392339	glpB	1.237	1.476	0	0
6822	2392681	glpB, glpC	1.022	1.165	0	0
1950	2392976	glpB, glpC	0.804	0.880	0	0
10031	2393096	glpC	0.586	0.592	0	0

15811	2393373	glpC	0.986	1.211	0	0
4552	2393576	glpC	1.086	0.936	0	0
9367	2393724	glpC	0.968	0.857	0	0
571	2394096	glpC	1.680	1.035	0	0
7909	2394196	glpC	2.104	0.864	0	0
4371	2394480	STM2287	3.667	0.589	1	0
2984	2394707	STM2287	2.714	0.526	1	0
5962	2394933	STM2287	3.333	0.513	1	0
10767	2395221	STM2287	3.306	0.533	1	0
344	2395339	STM2287	2.981	0.537	1	0
20810	2395592	STM2287	4.514	0.590	1	0
3258	2395776		3.197	0.728	1	0
20531	2396038	STM2288, STM2289	2.242	0.652	1	0
5583	2396314	STM2288, STM2289	1.382	0.641	0	0
4436	2396575	STM2289	1.082	0.608	0	0
4651	2396787	STM2289	1.391	0.666	0	0
16515	2396893	STM2289, yfaV	1.304	0.630	0	0
957	2397085	STM2289, yfaV	1.384	0.708	0	0
8887	2397377	yfaV	1.433	0.736	0	0
6224	2397718	yfaV	1.223	0.685	0	0
17117	2397925	yfaV	1.482	0.965	0	0
6206	2398017	yfaV	1.374	0.818	0	0
5614	2398380	yfaV, yfaW	1.269	0.672	0	0
19312	2398673	yfaW	1.383	0.963	0	0
7737	2398744	yfaW	0.869	0.772	0	0
2442	2399111	yfaW	1.098	0.660	0	0
1121	2399228	yfaW	1.452	0.771	0	0
16067	2399428	yfaW, yfaX	1.656	0.859	0	0
19111	2399829	yfaW, yfaX	1.312	0.696	0	0
10301	2399900	yfaX	0.697	0.614	0	0
20392	2400002	yfaX	1.581	0.799	0	0
16626	2400216	yfaX	1.052	0.689	0	0
20916	2400425	yfaX	1.385	0.757	0	0
11874	2400803	STM2293	1.068	0.847	0	0
17189	2401052	STM2293	1.561	1.078	0	0
4253	2401163	STM2293	0.771	0.493	0	0

14046	2401520	STM2293	1.018	1.135	0	0
18561	2401691	STM2293	0.812	0.884	0	0
18877	2401952	STM2293, yfaZ	0.961	1.246	0	0
14204	2402274	yfaZ, yfaO	0.916	1.109	0	0
1786	2402522	yfaZ, yfaO	0.982	0.972	0	0
9526	2402605	yfaZ, yfaO	1.017	1.055	0	0
19065	2402805	yfaZ, yfaO	1.494	1.050	0	0
5098	2403083	ais	2.128	1.058	0	0
384	2403340	ais	2.717	1.664	0	0
1648	2403676	ais, yfbE	1.230	0.721	0	0
3044	2403747	ais, yfbE	2.372	1.456	0	0
10184	2403933	ais, yfbE	2.139	1.486	0	0
5664	2404191	yfbE	1.476	1.984	0	1
15544	2404300	yfbE	1.111	1.946	0	1
18762	2404794	yfbE, pmrF	0.865	1.819	0	1
21332	2404871	yfbE, pmrF	1.070	1.791	0	1
16724	2405057	yfbE, pmrF	0.965	1.827	0	1
9918	2405311	pmrF	0.858	2.049	0	1
16702	2405496	pmrF	0.898	2.013	0	1
21791	2405734	pmrF, yfbG	0.911	2.043	0	1
2086	2406074	yfbG	0.847	1.565	0	0
16776	2406314	yfbG	1.027	2.019	0	0
12674	2406602	yfbG	0.698	1.515	0	0
15962	2406783	yfbG	0.880	2.147	0	1
18697	2407056	yfbG	0.927	1.666	0	1
6133	2407166	yfbG	1.215	1.678	0	0
17264	2407388	yfbG	1.012	1.845	0	0
19225	2407697	yfbG, STM2300	0.863	1.698	0	0
12194	2407908	yfbG, STM2300	0.700	1.380	0	0
19515	2408067	STM2300	0.922	1.963	0	1
16807	2408345	STM2300	0.793	1.873	0	1
14878	2408553	STM2300, pqaB	1.026	2.635	0	1
11841	2408691	STM2300, pqaB	0.840	1.811	0	1
1563	2409137	pqaB	0.894	1.512	0	0
10595	2409312	pqaB	0.847	1.897	0	0
3403	2409449	pqaB	0.758	1.609	0	0

13727	2409657	pqaB	0.913	1.940	0	1
17784	2409864	pqaB	0.712	1.876	0	1
16266	2410089	pqaB	0.654	1.255	0	0
19048	2410458	pqaB, STM2302	0.714	1.535	0	0
4500	2410559	pqaB, STM2302	0.803	2.140	0	1
21033	2410775	STM2302, STM2303	0.775	2.631	0	1
19656	2410919	STM2303	0.712	2.735	0	1
5906	2411354	pmrD	0.744	2.121	0	1
9303	2411598	pmrD	0.926	1.685	0	0
20415	2411833	pmrD, menE	0.817	1.506	0	0
16865	2412051	menE	0.870	1.285	0	0
12576	2412204	menE	0.722	1.041	0	0
7327	2412375	menE	0.458	0.813	0	0
10857	2412576	menE	1.053	1.179	0	0
13608	2412656	menE	1.525	1.481	0	0
2355	2413020	menE, menC	1.156	1.125	0	0
1029	2413426	menE, menC	1.610	1.159	0	0
3308	2413552	menC	0.759	0.609	0	0
7355	2413754	menC	0.591	0.641	0	0
22033	2413996	menC, menB	1.330	1.065	0	0
21483	2414294	menC, menB	0.937	0.885	0	0
15240	2414520	menB	0.598	0.566	0	0
880	2414819	menB	1.146	1.004	0	0
17283	2414907	menB, yfbB	0.983	0.871	0	0
13668	2415178	menB, yfbB	1.298	1.007	0	0
11319	2415365	yfbB	0.990	0.768	0	0
16045	2415634	yfbB, menD	1.293	1.101	0	0
6055	2415797	yfbB, menD	0.750	0.639	0	0
11015	2416087	menD	0.453	0.631	0	0
19547	2416357	menD	1.256	0.953	0	0
3961	2416535	menD	0.761	0.745	0	0
2037	2416742	menD	0.874	0.689	0	0
15787	2416984	menD	1.004	0.966	0	0
10384	2417289	menD	1.413	0.858	0	0
17421	2417370	menD, menF	1.473	1.058	0	0
4612	2417566	menD, menF	1.023	0.774	0	0

3375	2417812	menF	1.146	1.134	0	0
16704	2417994	menF	1.072	1.088	0	0
14998	2418104	menF	0.466	0.843	0	0
6909	2418650	menF	0.841	1.878	0	0
13769	2418721	menF	0.776	1.448	0	0
16247	2419034	menF, elaB	0.746	1.352	0	0
7847	2419368	elaB, elaA	0.915	1.163	0	0
10970	2419580	elaA, elaC	0.787	0.823	0	0
9000	2419795	elaA, elaC	0.994	1.199	0	0
17394	2419922	elaA, elaC	0.884	1.014	0	0
3834	2420003	elaA, elaC	1.351	1.404	0	0
9474	2420450	elaC, STM2314	1.265	1.380	0	0
825	2420559	STM2314	1.205	1.410	0	0
22046	2420717	STM2314	1.224	1.343	0	0
9164	2421032	STM2314	1.018	1.304	0	0
2284	2421379	STM2314	1.050	1.217	0	0
15270	2421498	STM2314	1.263	1.398	0	0
15323	2421671	yfbK	0.603	0.864	0	0
10026	2421979	yfbK	0.841	0.824	0	0
5801	2422296	yfbK	1.156	0.834	0	0
3129	2422394	yfbK	0.952	0.605	0	0
8568	2422734	yfbK	0.852	0.689	0	0
10147	2422864	yfbK	1.575	0.828	0	0
21603	2423078	yfbK	2.572	0.820	1	0
3707	2423329	yfbK	4.514	0.733	1	0
19691	2423665	yfbK	5.443	0.784	1	0
4343	2423884	yfbK	3.951	1.004	1	0
16047	2423985		3.418	1.007	1	0
9282	2424247		2.140	1.504	1	0
9114	2424509	nuoN	0.919	1.358	0	0
3164	2424620	nuoN	0.868	1.269	0	0
12838	2424878	nuoN	0.741	1.667	0	0
14570	2425134	nuoN	0.533	1.124	0	0
1410	2425328	nuoN	0.965	2.067	0	0
21889	2425473	nuoN	0.646	1.554	0	0
8877	2425783	nuoN	0.643	1.566	0	0

16390	2425873	nuoN, nuoM	0.613	1.339	0	0
1899	2426379	nuoM	0.855	1.825	0	0
13497	2426505	nuoM	0.699	1.405	0	0
13465	2426676	nuoM	0.783	1.985	0	1
15660	2426973	nuoM	0.747	2.271	0	1
14873	2427289	nuoM	0.707	1.581	0	0
9260	2427361	nuoM	0.665	2.032	0	1
16728	2427551	nuoM	0.715	2.181	0	1
12616	2427849	nuoL	0.632	1.863	0	1
14116	2428175	nuoL	0.825	2.284	0	1
13019	2428257	nuoL	0.659	1.985	0	1
4054	2428656	nuoL	0.708	2.265	0	1
7470	2428892	nuoL	0.741	2.264	0	1
3384	2429129	nuoL	0.670	1.929	0	1
7977	2429265	nuoL	0.521	1.779	0	1
12407	2429466	nuoL	0.643	2.141	0	1
18043	2429742	nuoL, nuoK	0.624	1.721	0	0
8581	2429915	nuoL, nuoK	0.623	2.124	0	0
18198	2430133	nuoK, nuoJ	0.344	0.875	0	0
10088	2430330	nuoJ	0.623	2.193	0	1
15280	2430485	nuoJ, nuoI	0.752	2.507	0	1
20885	2430859	nuoI	0.575	2.235	0	1
14281	2431003	nuoI, nuoH	0.690	2.154	0	1
9157	2431168	nuoI, nuoH	0.622	1.935	0	1
14218	2431531	nuoH	0.612	1.624	0	0
2923	2431657	nuoH	0.600	1.958	0	1
13487	2431842	nuoH	0.684	2.510	0	1
496	2432116	nuoH, nuoG	0.857	2.984	0	1
21907	2432384	nuoG	0.617	2.336	0	1
5084	2432675	nuoG	0.809	2.478	0	1
7043	2432986	nuoG	0.418	1.067	0	0
2969	2433149	nuoG	0.305	0.683	0	0
4491	2433291	nuoG	0.645	2.008	0	0
13047	2433466	nuoG	0.513	1.532	0	0
5900	2433834	nuoG	0.646	1.614	0	0
2709	2433948	nuoG	0.612	2.325	0	0

20926	2434339	nuoG	0.556	1.710	0	0
14607	2434596	nuoG	0.775	2.488	0	1
15720	2434676	nuoG, nuoF	0.739	2.332	0	1
17176	2434795	nuoG, nuoF	0.756	2.559	0	1
2155	2435037	nuoG, nuoF	0.632	3.117	0	1
6551	2435230	nuoF	0.513	1.485	0	0
12375	2435465	nuoF	0.631	2.318	0	0
3008	2435719	nuoF	0.415	1.524	0	0
642	2435959	nuoF, nuoE	0.823	2.826	0	1
5974	2436033	nuoF, nuoE	0.576	2.252	0	1
5373	2436489	nuoE, nuoC	0.640	2.676	0	1
17108	2436640	nuoE, nuoC	0.513	1.977	0	1
5060	2436928	nuoC	0.780	2.420	0	1
5007	2437298	nuoC	0.678	2.538	0	1
19655	2437435	nuoC	0.472	1.854	0	1
9714	2437690	nuoC	0.515	2.015	0	1
1870	2437946	nuoC	0.748	2.457	0	1
5423	2438057	nuoC	0.599	2.178	0	1
12385	2438325	nuoC	0.498	2.187	0	1
4069	2438573	nuoC, nuoB	0.437	2.097	0	1
4207	2438895	nuoB	0.407	1.869	0	1
18213	2439137	nuoB, nuoA	0.302	0.860	0	0
17818	2439240	nuoB, nuoA	0.484	1.991	0	1
10368	2439476	nuoB, nuoA	0.304	1.806	0	1
22292	2439688	nuoA	0.433	1.836	0	1
21535	2439930	nuoA, STM2329	0.407	1.824	0	0
19754	2440173	STM2329, lrhA	0.566	1.180	0	0
9845	2440244	STM2329, lrhA	0.619	1.252	0	0
2035	2440596	lrhA	0.742	0.735	0	0
9733	2440750	lrhA	1.170	0.751	0	0
2140	2441096	lrhA	2.007	0.579	0	0
4366	2441281	lrhA	2.163	0.471	0	0
9973	2441408	lrhA	1.647	0.515	0	0
1745	2441754	yfbQ	1.478	0.654	0	0
6799	2441961	yfbQ	1.677	0.713	0	0
4505	2442233	yfbQ	1.132	0.679	0	0

20726	2442479	yfbQ	1.093	0.904	0	0
8986	2442594	yfbQ	1.030	0.916	0	0
8666	2442986	yfbQ, STM2332	1.052	0.973	0	0
7489	2443171	yfbQ, STM2332	0.904	0.953	0	0
4426	2443290	STM2332	0.918	0.882	0	0
9962	2443554	STM2332	0.961	0.795	0	0
13335	2443845	STM2332	1.108	1.175	0	0
8637	2443974	yfbS	0.961	1.006	0	0
8570	2444060	yfbS	0.826	1.135	0	0
10029	2444405	yfbS	1.158	1.111	0	0
2772	2444657	yfbS	0.893	0.958	0	0
20157	2444785	yfbS	1.167	1.224	0	0
7107	2445215	yfbS	0.686	0.818	0	0
20736	2445290	yfbS	0.638	0.811	0	0
12802	2445677	yfbS	1.126	1.374	0	0
11399	2445782	yfbS	1.063	1.265	0	0
21199	2445892	yfbS, yfbT	0.653	1.020	0	0
15857	2446235	yfbS, yfbT	0.712	0.914	0	0
5709	2446496	yfbT	1.060	1.419	0	0
10974	2446696	yfbT, yfbU	0.903	1.096	0	0
7015	2447045	yfbU	0.724	1.017	0	0
13597	2447126	yfbU, STM2336	0.760	1.130	0	0
16200	2447242	yfbU, STM2336	0.562	0.822	0	0
15814	2447551	STM2336, ackA	0.703	1.075	0	0
13902	2447913	STM2336, ackA	0.514	0.933	0	0
20567	2447992	STM2336, ackA	0.518	0.845	0	0
2521	2448226	ackA	0.778	1.187	0	0
1033	2448340	ackA	0.779	1.225	0	0
9693	2448496	ackA	0.897	1.121	0	0
3619	2448687	ackA	0.744	1.202	0	0
5889	2449198	pta	0.809	1.002	0	0
3336	2449569	pta	0.992	0.967	0	0
20088	2449772	pta	0.984	1.224	0	0
16209	2449930	pta	0.513	0.715	0	0
13647	2450198	pta	0.324	0.619	0	0
9545	2450270	pta	1.263	1.265	0	0

19912	2450576	pta	0.773	0.933	0	0
5904	2450811	pta	0.670	0.763	0	0
15325	2451146	pta, yfcC	0.876	1.235	0	0
2773	2451226	pta, yfcC	0.903	1.181	0	0
11001	2451530	yfcC	0.670	0.909	0	0
3405	2451837	yfcC	0.678	0.637	0	0
15040	2452014	yfcC	0.887	0.774	0	0
6859	2452118	yfcC	0.400	0.451	0	0
8302	2452529	yfcC	1.249	0.919	0	0
2957	2452717	yfcC	1.226	1.019	0	0
15652	2452903	yfcC	0.935	1.091	0	0
2003	2453189	STM2340	0.812	1.284	0	0
11555	2453354	STM2340	1.123	1.464	0	0
6969	2453580	STM2340	1.115	1.368	0	0
1081	2453764	STM2340	1.216	1.591	0	0
20142	2454089	STM2340, STM2341	1.237	1.479	0	0
11933	2454313	STM2340, STM2341	1.117	1.398	0	0
3210	2454392	STM2340, STM2341	0.866	0.915	0	0
5147	2454813	STM2341	0.548	0.887	0	0
20227	2454915	STM2341, STM2342	1.002	1.516	0	0
15564	2455130	STM2341, STM2342	0.660	0.830	0	0
15693	2455437	STM2342	1.149	1.102	0	0
12226	2455589	STM2342	1.669	0.967	0	0
298	2455910	STM2342	3.975	1.243	1	0
9737	2456077	STM2342	3.929	1.068	1	0
11607	2456151	STM2342	4.131	1.155	1	0
10891	2456591	STM2342, STM2343	2.720	1.014	1	0
9464	2456666	STM2342, STM2343	3.434	1.039	1	0
15763	2456863	STM2343, STM2344	2.274	1.287	1	0
2890	2457138	STM2344, STM2345	1.115	1.058	0	0
4588	2457312	STM2344, STM2345	1.001	1.042	0	0
5316	2457493	STM2344, STM2345	0.872	0.913	0	0
11849	2457860	STM2345	1.078	1.365	0	0
21983	2457965	STM2345	0.898	1.414	0	0
19449	2458354	STM2345	0.695	1.904	0	1
16840	2458609	STM2346	0.879	1.765	0	1

1443	2458888	STM2346	0.788	1.794	0	1
6184	2459049	STM2346, yfcE	0.706	1.548	0	0
11991	2459120	STM2346, yfcE	0.756	1.658	0	0
9667	2459554	yfcE	0.860	1.441	0	0
11612	2459742	yfcE, yfcF	0.988	1.257	0	0
22057	2459877	yfcE, yfcF	1.089	1.228	0	0
15530	2460135	yfcF, yfcG	0.894	0.959	0	0
16773	2460245	yfcF, yfcG	1.128	1.080	0	0
21399	2460371	yfcF, yfcG	1.038	0.981	0	0
22324	2460523	yfcF, yfcG	1.163	1.318	0	0
3080	2461119	yfcH	1.031	1.648	0	0
8125	2461320	yfcH	0.680	0.689	0	0
8607	2461423	yfcH	1.114	1.155	0	0
11498	2461774	yfcH	0.985	1.073	0	0
11833	2461919	yfcH	1.169	1.226	0	0
743	2462197	hisP	1.380	1.452	0	0
6944	2462529	hisP	1.015	1.100	0	0
7418	2462647	hisP	0.940	0.844	0	0
16302	2462859	hisP	1.255	0.877	0	0
20506	2463151	hisP, hisM	1.559	1.091	0	0
17449	2463355	hisM	1.171	0.995	0	0
6366	2463660	hisM, hisQ	0.351	0.582	0	0
5387	2463731	hisM, hisQ	1.178	1.024	0	0
17975	2464070	hisQ	1.224	1.293	0	0
12708	2464312	hisQ	0.316	0.436	0	0
12136	2464508	hisQ, hisJ	0.934	1.000	0	0
8203	2464754	hisJ	0.468	0.616	0	0
20001	2464900	hisJ	0.522	0.589	0	0
9706	2465228	hisJ	0.469	0.536	0	0
20940	2465383	hisJ	0.742	0.823	0	0
14407	2465499	hisJ, argT	0.760	0.930	0	0
16424	2465776	argT	0.659	0.889	0	0
4924	2466082	argT	0.728	0.712	0	0
3738	2466276	argT	0.661	0.813	0	0
6069	2466347	argT	0.778	0.904	0	0
12875	2466613	argT, ubiX	0.659	0.942	0	0

15093	2466849	ubiX	0.695	1.003	0	0
2056	2467226	ubiX, STM2357	0.528	0.997	0	0
14978	2467474	ubiX, STM2357	0.877	1.638	0	0
2714	2467731	STM2357	1.102	1.465	0	0
15829	2467943	STM2357	0.911	0.838	0	0
19994	2468083	STM2357	1.359	0.878	0	0
16729	2468277	STM2357	1.306	0.781	0	0
7800	2468354	STM2357	1.318	0.829	0	0
13801	2468703	STM2357, STM2358	0.989	0.684	0	0
17742	2469027	STM2358	1.762	0.834	0	0
20513	2469304	STM2358	2.096	0.707	1	0
17054	2469532	STM2358	2.546	0.732	1	0
19758	2469729	STM2358, STM2359	3.035	0.794	1	0
12937	2469880	STM2358, STM2359	3.355	0.767	1	0
11412	2470004	STM2358, STM2359	2.434	0.684	1	0
15088	2470112	STM2358, STM2359	1.892	0.611	0	0
19562	2470603	STM2359	1.534	0.614	0	0
12999	2470911	STM2359	2.308	0.747	0	0
19323	2471099	STM2359	1.630	0.656	0	0
3416	2471249	STM2359, STM2360	0.978	0.610	0	0
2022	2471547	STM2359, STM2360	1.474	0.643	0	0
1959	2471642	STM2360	1.094	0.585	0	0
11138	2471723	STM2360	1.262	0.645	0	0
9091	2471820	STM2360	1.485	0.610	0	0
4988	2472474	STM2360, STM2361	0.754	0.630	0	0
8709	2472620	STM2360, STM2361	0.732	0.579	0	0
21719	2472838	STM2360, STM2361	0.809	0.673	0	0
5890	2473000	STM2360, STM2361	0.886	0.668	0	0
11000	2473072	STM2360, STM2361	1.002	0.739	0	0
4806	2473270	STM2361	0.674	0.626	0	0
21675	2473756	STM2361	1.049	0.893	0	0
15505	2474025	STM2361	0.592	0.700	0	0
18596	2474222	STM2361	0.863	1.203	0	0
10120	2474449	purF	0.858	1.143	0	0
6987	2474521	purF	0.912	1.176	0	0
17070	2474926	purF	0.843	1.371	0	0

15929	2475004	purF	0.815	1.110	0	0
15567	2475445	purF	0.519	0.951	0	0
6332	2475531	purF	0.638	1.121	0	0
9935	2475901	purF, cvpA	0.725	1.772	0	1
8352	2475975	purF, cvpA	0.710	1.905	0	1
6200	2476192	purF, cvpA	0.488	1.697	0	0
21953	2476324	cvpA	0.628	2.693	0	1
3425	2476418	cvpA	0.636	2.681	0	1
19037	2476721	cvpA, dedD	0.624	2.793	0	1
9616	2477226	dedD	0.699	2.493	0	1
1885	2477337	dedD, folC	0.574	1.649	0	0
4146	2477467	dedD, folC	0.730	2.298	0	0
8875	2477562	dedD, folC	0.544	1.580	0	0
18869	2478215	folC	0.666	1.174	0	0
14680	2478354	folC	0.691	1.551	0	0
13495	2478522	folC	0.899	2.389	0	0
5157	2478607	folC	1.072	1.974	0	0
2854	2478995	folC, accD	0.518	1.601	0	0
7184	2479361	accD	0.521	1.886	0	1
11809	2479573	accD	0.611	1.923	0	1
9143	2479811	accD, dedA	0.743	1.418	0	0
21423	2479925	accD, dedA	0.861	1.479	0	0
2613	2480085	dedA	0.777	1.470	0	0
20992	2480386	dedA	0.834	1.366	0	0
7533	2480648	dedA, truA	0.856	1.073	0	0
2165	2480871	truA	1.234	1.090	0	0
13473	2480988	truA	0.622	0.939	0	0
21541	2481353	truA, usg	0.804	1.066	0	0
11274	2481591	truA, usg	0.747	1.123	0	0
870	2481710	truA, usg	0.855	1.556	0	0
20221	2481906	usg	0.929	1.569	0	0
725	2482258	usg	0.952	1.889	0	1
20347	2482334	usg, pdxB	0.893	1.755	0	1
1746	2482460	usg, pdxB	0.951	2.249	0	1
15487	2482927	pdxB	1.082	1.206	0	0
21024	2483057	pdxB	0.839	1.256	0	0

9635	2483444	pdxB, flk	1.139	2.144	0	1
14197	2483550	pdxB, flk	1.358	2.165	0	1
15246	2483732	pdxB, flk	1.020	1.485	0	0
15867	2484067	flk	0.973	1.434	0	0
6507	2484314	flk	0.833	1.256	0	0
5642	2484467	flk	1.005	1.512	0	0
6252	2484557	flk	1.000	1.277	0	0
15964	2484877	STM2372	0.385	0.763	0	0
7770	2485032	STM2372	1.045	1.333	0	0
14563	2485387	STM2372	1.046	1.553	0	0
19764	2485610	STM2372	1.048	2.217	0	1
12394	2485871	STM2372	1.075	1.986	0	1
11124	2486149	STM2372, STM2373	2.318	1.046	1	0
6925	2486316	STM2373, STM2374	2.732	0.710	1	0
18387	2486473	STM2373, STM2374	3.490	0.533	1	0
8918	2486832	STM2375, STM2376	3.925	0.420	1	0
1638	2486919	STM2375, STM2376	3.921	0.509	1	0
16743	2487167	STM2375, STM2376	2.762	0.707	1	0
6945	2487476	STM2376	0.946	0.576	0	0
10874	2487598	STM2376	1.142	0.638	0	0
12206	2487932	STM2377	4.710	0.717	1	0
2402	2488035	STM2377	3.792	0.827	1	0
5985	2488439	STM2377	4.160	0.911	1	0
8055	2488580	STM2377	3.428	0.930	1	0
7108	2488666	STM2377, fabB	2.973	1.247	1	0
19806	2488897	STM2377, fabB	1.420	1.903	0	0
5832	2489278	fabB	0.503	1.048	0	0
13992	2489599	fabB, STM2379	0.881	2.327	0	1
2959	2489819	fabB, STM2379	0.813	1.743	0	1
9320	2489912	fabB, STM2379	0.906	1.758	0	1
3240	2490020	fabB, STM2379	0.820	1.537	0	0
4361	2490118	fabB, STM2379	1.120	1.410	0	0
2039	2490267	fabB, STM2379	1.209	1.531	0	0
9396	2490666	STM2379	1.383	1.198	0	0
18098	2490925	STM2379	1.033	1.208	0	0
7459	2491351	STM2379	0.655	0.781	0	0

8766	2491588	STM2379	0.949	1.130	0	0
7346	2491728	STM2379	0.801	1.045	0	0
3475	2491955	STM2379	1.232	1.727	0	1
19618	2492268	yfcL	1.078	1.964	0	1
5476	2492512	yfcL, yfcM	1.002	1.435	0	0
20360	2492583	yfcL, yfcM	0.988	1.521	0	0
20680	2492875	yfcM, yfcA	0.944	1.209	0	0
4847	2492972	yfcM, yfcA	1.054	1.299	0	0
2011	2493332	yfcM, yfcA	1.325	1.487	0	0
5931	2493450	yfcA	1.237	1.547	0	0
19728	2493680	yfcA	0.877	1.385	0	0
16527	2493977	yfcA, mepA	0.951	1.622	0	0
5927	2494163	mepA	0.685	1.010	0	0
5479	2494527	mepA, aroC	0.801	1.610	0	0
7358	2494662	mepA, aroC	0.935	1.902	0	1
17862	2494868	mepA, aroC	0.772	1.595	0	1
3073	2495080	aroC	1.002	1.470	0	0
20970	2495278	aroC	1.058	1.691	0	0
14745	2495614	aroC	0.716	1.150	0	0
7546	2495766	aroC, yfcB	0.814	1.165	0	0
21029	2495974	aroC, yfcB	1.112	1.468	0	0
20353	2496181	yfcB	1.167	1.325	0	0
18940	2496536	yfcB, yfcN	1.132	1.649	0	0
6637	2496723	yfcB, yfcN	1.057	1.331	0	0
19373	2496826	yfcB, yfcN	1.194	1.543	0	0
6417	2496962	yfcB, yfcN	1.057	1.563	0	0
21096	2497404	sixA	0.681	1.839	0	0
8242	2497732	sixA	0.472	1.406	0	0
9085	2497876	sixA	0.724	1.955	0	0
12044	2498054	sixA	0.787	1.580	0	0
13284	2498433	yfcX	0.817	0.919	0	0
11499	2498509	yfcX	0.962	0.974	0	0
20084	2498596	yfcX	0.886	1.056	0	0
1293	2499081	yfcX	0.908	1.216	0	0
15318	2499171	yfcX	0.964	0.992	0	0
15183	2499481	yfcX	1.028	0.935	0	0

6273	2499814	yfcX	1.313	0.852	0	0
8956	2499934	yfcX	0.907	0.860	0	0
16711	2500203	yfcX	1.193	0.893	0	0
10231	2500380	yfcX, yfcY	0.862	0.717	0	0
8966	2500611	yfcX, yfcY	1.110	0.726	0	0
12263	2500829	yfcY	0.894	0.632	0	0
11505	2501075	yfcY	1.194	0.779	0	0
12962	2501350	yfcY	1.117	0.870	0	0
15133	2501591	yfcY	1.101	0.907	0	0
16501	2501751	yfcY, yfcZ	1.247	1.149	0	0
13636	2502008	yfcZ, fadL	1.121	0.995	0	0
14322	2502096	yfcZ, fadL	1.065	0.983	0	0
12593	2502374	yfcZ, fadL	0.593	0.590	0	0
21863	2502745	fadL	0.741	0.930	0	0
2756	2502823	fadL	0.978	1.076	0	0
9083	2503110	fadL	0.595	0.649	0	0
12166	2503395	fadL	0.846	1.206	0	0
17583	2503664	fadL	0.816	1.272	0	0
9255	2503737	vacJ	0.808	1.210	0	0
5653	2503942	vacJ	0.988	1.649	0	0
20519	2504183	vacJ	0.903	1.559	0	0
18022	2504572	vacJ, yfdC	1.092	1.265	0	0
11207	2504765	vacJ, yfdC	1.272	0.906	0	0
18399	2504842	vacJ, yfdC	1.128	0.672	0	0
19428	2505080	yfdC	1.271	0.830	0	0
4846	2505324	yfdC, argW	1.277	0.821	0	0
12414	2505487	yfdC, argW	0.999	0.875	0	0
21579	2505908		1.371	2.063	0	0
1434	2506051	pgtE	1.917	1.557	0	0
20177	2506168	pgtE	2.165	1.066	0	0
17004	2506579	pgtE	2.895	0.794	0	0
2333	2506833	pgtE	2.037	0.560	0	0
19767	2506972	pgtE	2.855	0.524	0	0
20372	2507161	pgtE	3.690	0.580	1	0
6571	2507342	pgtE, pgtA	3.743	0.582	1	0
3827	2507586	pgtA	2.304	0.686	0	0

14457	2507909	pgtA	1.593	0.888	0	0
2482	2508150	pgtA	1.400	0.742	0	0
17243	2508248	pgtA	1.326	0.848	0	0
15992	2508478	pgtA, pgtB	1.112	0.865	0	0
22087	2508620	pgtA, pgtB	1.007	0.823	0	0
11623	2508990	pgtB	1.646	1.053	0	0
21456	2509328	pgtB	1.174	0.960	0	0
21074	2509512	pgtB	1.224	0.901	0	0
3200	2509746	pgtB	1.493	0.886	0	0
16231	2509817	pgtB	1.717	0.860	0	0
2603	2510190	pgtB	1.684	0.807	0	0
3807	2510310	pgtB	1.772	0.759	0	0
15194	2510534	pgtB, pgtC	1.476	0.744	0	0
4561	2510886	pgtB, pgtC	1.448	0.814	0	0
21129	2511034	pgtC	1.391	0.813	0	0
4694	2511106	pgtC	1.551	0.924	0	0
11076	2511495	pgtC	2.459	0.666	1	0
21482	2511720	pgtC, pgtP	3.750	0.581	1	0
12508	2512046	pgtC, pgtP	4.116	0.593	1	0
5111	2512269	pgtP	3.366	0.472	1	0
19558	2512340	pgtP	3.116	0.507	1	0
15094	2512462	pgtP	2.775	0.701	1	0
893	2512924	pgtP	1.360	0.622	0	0
15543	2513128	pgtP	1.415	0.562	0	0
1016	2513340	pgtP	0.805	0.502	0	0
20381	2513550		1.455	0.701	0	0
20100	2513808	STM2400	2.537	0.709	1	0
9836	2513928	STM2400	2.632	0.682	1	0
3524	2514340	STM2400, ddg	2.665	0.623	1	0
3039	2514414	STM2400, ddg	2.324	0.755	0	0
18819	2514542	ddg	1.653	0.694	0	0
13317	2514736	ddg	1.584	0.743	0	0
8409	2515142	ddg	0.771	0.497	0	0
6352	2515270	ddg	1.462	0.611	0	0
712	2515543		1.352	0.808	0	0
4862	2515648		1.354	0.905	0	0

14268	2515808		1.052	0.975	0	0
18569	2516142	yfdZ	0.815	1.280	0	0
4019	2516609	yfdZ	0.545	0.769	0	0
2728	2516690	yfdZ	0.707	0.883	0	0
346	2516937	yfdZ	0.861	1.320	0	0
7483	2517290	yfdZ	0.658	0.869	0	0
18593	2517362	yfdZ	0.662	0.839	0	0
9915	2517750		0.639	0.784	0	0
2332	2517831		0.794	0.746	0	0
11671	2518002	glk	0.771	0.991	0	0
20258	2518396	glk	1.070	1.420	0	0
1539	2518665	glk, STM2404	0.881	1.125	0	0
17135	2518894	glk, STM2404	1.072	1.108	0	0
20657	2519026	glk, STM2404	1.182	1.213	0	0
22103	2519098	glk, STM2404	1.177	1.216	0	0
3257	2519462	STM2404	0.861	0.794	0	0
4004	2519633	STM2404	1.199	0.901	0	0
21446	2519894	STM2404	1.332	1.062	0	0
820	2520195	STM2404	0.820	0.704	0	0
2896	2520425	STM2405	1.547	1.159	0	0
3639	2520562	STM2405	0.955	0.811	0	0
6826	2520900	STM2405	1.264	0.784	0	0
11966	2521142	STM2405	1.199	0.771	0	0
13105	2521255	STM2405	0.794	0.622	0	0
1509	2521568	STM2405	0.823	0.859	0	0
13188	2521647	STM2405	1.156	0.897	0	0
15122	2522018	STM2405, STM2406	1.171	0.881	0	0
10484	2522210	STM2405, STM2406	1.178	0.923	0	0
17523	2522443	STM2405, STM2406	1.024	0.892	0	0
22312	2522705	STM2406	1.204	1.158	0	0
12927	2522782	STM2406	0.884	0.698	0	0
18878	2523203	ypeC	1.087	0.880	0	0
14914	2523413	ypeC	1.024	1.384	0	0
17181	2523637		0.633	1.101	0	0
15440	2523708	mntH	0.749	1.012	0	0
6295	2523913	mntH	0.700	0.789	0	0

4793	2524224	mntH	0.943	0.914	0	0
12324	2524573	mntH	0.918	0.891	0	0
18578	2524649	mntH	0.932	1.051	0	0
11914	2524930	mntH, nupC	0.634	0.935	0	0
8483	2525050	mntH, nupC	0.558	0.752	0	0
19260	2525252	mntH, nupC	0.599	0.865	0	0
2419	2525524	nupC	0.586	0.980	0	0
14011	2525897	nupC	0.619	0.907	0	0
20002	2525998	nupC	0.659	0.832	0	0
2007	2526319	nupC	0.765	1.075	0	0
9352	2526398	nupC	0.681	0.826	0	0
4344	2526702	yfeA	0.987	1.078	0	0
11601	2526847	yfeA	0.894	0.897	0	0
17606	2527089	yfeA	1.013	1.085	0	0
12610	2527434	yfeA	1.056	0.870	0	0
2374	2527634	yfeA	1.127	1.098	0	0
5565	2527920	yfeA	1.113	0.847	0	0
17570	2528204	yfeA	0.829	0.973	0	0
15157	2528323	yfeA	0.906	1.167	0	0
10758	2528657	yfeA	0.635	2.032	0	1
16796	2528847	yfeA	0.424	2.417	0	1
11346	2529008	yfeA, alaX	0.524	3.249	0	1
17882	2529313	alaX, alaW	0.468	2.658	0	1
10602	2529491	alaW, yfeC	0.545	2.309	0	1
4230	2529661	yfeC, yfeD	0.723	1.820	0	0
3959	2529774	yfeD	0.689	1.665	0	1
12837	2530057	yfeD	0.943	2.046	0	1
19816	2530138	gltX	0.807	2.197	0	1
8631	2530641	gltX	0.530	0.964	0	0
6620	2530853	gltX	0.679	1.529	0	0
19394	2530955	gltX	0.677	1.407	0	0
18139	2531071	gltX	0.540	1.084	0	0
6897	2531609	gltX, valU	0.416	3.611	0	1
13340	2531776	gltX, valU	0.448	3.481	0	1
14170	2532027	valY, lysV	0.383	5.453	0	1
20675	2532138	lysV	0.348	2.673	0	1

13648	2532511	xapR	0.530	1.890	0	1
16842	2532627	xapR	0.791	1.894	0	1
5457	2532870	xapR	1.569	1.130	0	0
15692	2533121	xapR	3.328	0.764	1	0
4681	2533266	xapR	4.748	0.611	1	0
3895	2533461	xapR	4.055	0.700	1	0
9658	2533784	xapB	2.395	0.707	1	0
16641	2534070	xapB	1.617	0.836	0	0
17849	2534162	xapB	1.347	0.790	0	0
3179	2534441	xapB	1.126	0.793	0	0
18680	2534775	xapB	1.454	0.648	0	0
9492	2534850	xapB, xapA	1.293	0.633	0	0
12542	2535187	xapB, xapA	1.680	0.667	0	0
17284	2535343	xapA	0.981	0.516	0	0
16605	2535624	xapA, yfeN	5.410	0.674	1	0
7125	2535739	xapA, yfeN	5.196	0.517	1	0
4944	2536133	yfeN	4.063	0.662	1	0
22049	2536324	yfeN	2.706	0.570	1	0
20533	2536401	yfeN	2.324	0.370	1	0
21372	2536660	yfeN	1.455	0.726	0	0
7316	2536742	yfeR	0.479	0.543	0	0
15952	2537297	yfeR, yfeH	1.448	0.781	0	0
8361	2537420	yfeR, yfeH	0.877	0.689	0	0
14267	2537751	yfeR, yfeH	1.405	0.777	0	0
19226	2537983	yfeR, yfeH	0.881	0.981	0	0
6671	2538054	yfeR, yfeH	1.002	0.776	0	0
335	2538356	yfeH	1.080	1.049	0	0
2325	2538624	yfeH	0.814	0.932	0	0
11632	2538874	STM2426	0.786	1.067	0	0
1167	2538967	STM2426	0.903	1.433	0	0
2806	2539198	STM2426, lig	0.926	1.484	0	0
20216	2539303	STM2426, lig	1.072	1.375	0	0
6219	2539612	lig	0.743	1.170	0	0
11527	2539903	lig	0.936	1.413	0	0
13094	2540162	lig	1.211	1.504	0	0
22190	2540456	lig	1.102	1.328	0	0

7023	2540687	lig	0.869	1.688	0	0
9850	2540758	lig	0.707	1.601	0	0
16273	2540982	lig	0.730	2.174	0	1
1600	2541379	lig, zipA	0.693	2.962	0	1
15085	2541477	zipA	1.097	2.708	0	1
8885	2541839	zipA, cysZ	0.538	2.391	0	1
17445	2541969	zipA, cysZ	0.648	2.734	0	1
5480	2542117	zipA, cysZ	0.512	1.530	0	0
16397	2542280	zipA, cysZ	0.709	1.734	0	0
17577	2542669	cysZ	0.794	0.976	0	0
12059	2542839	cysZ, cysK	0.969	0.925	0	0
17949	2543216	cysK	0.631	0.706	0	0
542	2543287	cysK	0.857	0.944	0	0
2664	2543636	cysK	0.630	1.071	0	0
2624	2543768	cysK	0.935	1.504	0	0
20332	2544078	cysK	0.554	2.271	0	1
16488	2544176	ptsH	0.572	2.343	0	1
8376	2544562	ptsH, ptsI	0.550	1.910	0	0
16076	2544642	ptsH, ptsI	0.563	1.940	0	0
7088	2544865	ptsI	0.566	1.449	0	0
3339	2545054	ptsI	0.709	1.508	0	0
16960	2545128	ptsI	0.642	1.287	0	0
20590	2545537	ptsI	0.569	1.200	0	0
1691	2545645	ptsI	0.727	1.903	0	1
9507	2546111	ptsI	0.665	1.679	0	1
14160	2546290	ptsI, crr	0.537	1.487	0	0
2145	2546557	ptsI, crr	0.887	1.787	0	0
18239	2546641	crr	0.515	1.615	0	0
9139	2547052	crr	0.687	1.905	0	1
21725	2547128	crr	0.667	1.664	0	1
5956	2547271		0.787	1.627	0	1
19245	2547577	STM2434, pdxK	0.262	0.543	0	0
6578	2547989	STM2434, pdxK	1.097	0.778	0	0
2190	2548090	pdxK, ptsJ	1.060	0.859	0	0
4310	2548378	pdxK, ptsJ	0.651	0.697	0	0
11876	2548452	pdxK, ptsJ	1.218	0.804	0	0

11102	2548608	pdxK, ptsJ	1.285	0.926	0	0
14059	2549006	ptsJ	1.118	0.813	0	0
8356	2549230	ptsJ	0.856	0.643	0	0
8440	2549421	ptsJ, yfeJ	0.532	0.408	0	0
11178	2549789	yfeJ	1.308	0.839	0	0
16790	2549906	yfeJ	1.137	0.908	0	0
9607	2550119	yfeJ, yfeK	1.008	0.975	0	0
17121	2550294	yfeJ, yfeK	1.129	1.311	0	0
21773	2550554	yfeK, yfeL	0.961	1.065	0	0
13912	2550859	yfeK, yfeL	1.038	1.205	0	0
9206	2551031	yfeL	0.916	1.249	0	0
6937	2551335	yfeL	1.038	1.384	0	0
13089	2551475	yfeL	1.003	1.254	0	0
13375	2551617		1.161	1.247	0	0
10375	2551846	cysM	1.154	1.097	0	0
7206	2552118	cysM	1.186	0.980	0	0
16548	2552529	cysM	1.218	1.382	0	0
20928	2552603	cysM, cysA	1.273	1.480	0	0
20554	2552803	cysM, cysA	0.904	0.857	0	0
16640	2553069	cysA	0.446	0.736	0	0
9827	2553446	cysA	1.241	0.626	0	0
7383	2553679	cysA	1.550	0.713	0	0
20203	2553755	cysA, cysW	1.509	0.752	0	0
8964	2553843	cysA, cysW	1.046	0.631	0	0
16505	2554121	cysA, cysW	1.201	0.697	0	0
2297	2554588	cysW, cysU	1.145	0.708	0	0
757	2554720	cysW, cysU	1.628	1.023	0	0
15739	2554928	cysW, cysU	1.358	1.209	0	0
17978	2555015	cysW, cysU	1.569	1.665	0	0
14282	2555167	cysU	0.603	0.823	0	0
10724	2555541	cysU, cysP	1.123	0.782	0	0
255	2555853	cysU, cysP	1.000	0.770	0	0
21160	2555989	cysP	1.043	0.810	0	0
3219	2556127	cysP	1.002	0.687	0	0
1017	2556514	cysP	1.225	0.936	0	0
11329	2556736	cysP, ucpA	1.006	1.153	0	0

7194	2557052	ucpA	1.288	1.111	0	0
15279	2557243	ucpA	1.360	1.215	0	0
15865	2557459	ucpA	1.147	0.800	0	0
11268	2557710	ucpA, STM2446	1.054	1.243	0	0
22231	2557944	STM2446	1.004	1.244	0	0
19527	2558111	STM2446	0.952	1.316	0	0
17754	2558197	STM2446	0.939	1.174	0	0
5792	2558500	STM2446	1.218	1.226	0	0
20066	2558711	STM2446, STM2447	1.026	1.203	0	0
4367	2559068	STM2447	0.796	1.020	0	0
11682	2559184	STM2447	0.760	1.231	0	0
6559	2559519	STM2447, yfeZ	1.046	1.567	0	0
5144	2559677	yfeZ, STM2449	0.877	1.526	0	0
4859	2559763	yfeZ, STM2449	0.697	1.207	0	0
2524	2560100	yfeZ, STM2449	0.945	1.374	0	0
15644	2560431	STM2449, amiA	0.819	1.058	0	0
4574	2560609	STM2449, amiA	0.881	0.899	0	0
15465	2560884	amiA, hemF	1.010	0.871	0	0
1094	2561143	amiA, hemF	0.821	0.865	0	0
16173	2561217	hemF	1.002	0.918	0	0
3161	2561454	hemF	0.919	0.871	0	0
19399	2561669	hemF, STM2452	0.607	0.512	0	0
13944	2561753	hemF, STM2452	1.098	0.945	0	0
7852	2562115	STM2452, STM2453	1.871	0.772	0	0
2851	2562526	STM2453	1.530	0.786	0	0
18718	2562699	STM2453	1.683	0.916	0	0
20333	2562850	STM2453	1.297	0.776	0	0
9236	2562960	STM2453	1.467	0.826	0	0
15135	2563345	STM2453	0.436	0.544	0	0
12770	2563436	STM2453	1.103	0.913	0	0
1147	2563759	STM2453	1.545	0.871	0	0
11679	2563835		1.322	1.109	0	0
17018	2564101	eutR	1.604	0.957	0	0
5668	2564470	eutR	1.337	0.954	0	0
9851	2564629	eutR	1.321	0.831	0	0
14414	2564889	eutR	1.482	0.920	0	0

19377	2565052	eutR	1.185	0.871	0	0
9438	2565158	eutR, eutK	1.517	0.914	0	0
21636	2565391	eutR, eutK	0.899	0.619	0	0
11605	2565611	eutK	1.479	1.060	0	0
9842	2566140	eutL	1.261	0.756	0	0
1266	2566289	eutL	1.647	0.712	0	0
19269	2566570	eutL, eutC	1.145	0.693	0	0
7064	2566677	eutL, eutC	1.575	0.762	0	0
21519	2566820	eutC	1.390	0.960	0	0
8034	2567196	eutC	1.482	0.661	0	0
15017	2567436	eutC, eutB	2.963	0.692	0	0
17718	2567586	eutC, eutB	1.103	0.692	0	0
10508	2567957	eutB	1.545	0.693	0	0
16542	2568139	eutB	1.346	0.594	0	0
6692	2568315	eutB	1.352	0.595	0	0
2649	2568586	eutB	1.702	0.622	0	0
14034	2568673	eutB, eutA	0.821	0.709	0	0
293	2568775	eutB, eutA	0.644	0.572	0	0
13601	2569181	eutA	1.122	0.751	0	0
6678	2569400	eutA	0.884	0.598	0	0
18097	2569796	eutA	1.039	0.741	0	0
11256	2569939	eutA	0.516	0.471	0	0
871	2570219	eutA, eutH	1.115	0.743	0	0
8750	2570304	eutA, eutH	1.520	0.896	0	0
11954	2570552	eutH	1.473	0.885	0	0
18571	2570630	eutH	1.466	0.794	0	0
13764	2571091	eutH	0.692	0.638	0	0
13588	2571227	eutH	1.104	0.829	0	0
20514	2571547	eutH, eutG	2.127	0.825	0	0
22038	2571725	eutG	1.250	0.645	0	0
9253	2572017	eutG	0.695	0.606	0	0
2640	2572121	eutG	0.996	0.617	0	0
6188	2572503	eutG	1.728	0.656	0	0
12608	2572708	eutG, eutJ	1.899	0.720	0	0
14853	2572984	eutG, eutJ	1.560	0.830	0	0
12704	2573167	eutJ	1.015	0.582	0	0

1925	2573341	eutJ	0.835	0.393	0	0
14479	2573475	eutJ, eutE	0.728	0.551	0	0
8107	2573703	eutJ, eutE	1.470	0.667	0	0
16010	2573957	eutE	0.993	0.626	0	0
13305	2574260	eutE	0.681	0.646	0	0
18767	2574526	eutE	1.651	0.716	0	0
10624	2574775	eutE	1.877	0.706	0	0
10761	2574846	eutE, eutN	1.552	0.613	0	0
7339	2575057	eutE, eutN	1.284	0.625	0	0
21416	2575203	eutE, eutN	1.032	0.613	0	0
15718	2575475	eutN, eutM	1.244	0.468	0	0
2497	2575680	eutM, eutD	1.416	1.131	0	0
10896	2576088	eutD	1.791	0.711	0	0
14590	2576332	eutD	0.923	0.594	0	0
11457	2576601	eutD, eutT	1.318	0.735	0	0
20505	2576688	eutD, eutT	1.538	0.851	0	0
1051	2576936	eutD, eutT	0.758	0.452	0	0
18898	2577299	eutT	1.407	0.757	0	0
7582	2577416	eutT, eutQ	1.460	0.759	0	0
12702	2577517	eutT, eutQ	1.561	0.714	0	0
20678	2577748	eutT, eutQ	1.571	0.796	0	0
1021	2578013	eutQ, eutP	2.623	0.629	1	0
19935	2578305	eutQ, eutP	4.742	0.564	1	0
16151	2578503	eutP, eutS	4.438	0.543	1	0
497	2578817	eutP, eutS	3.906	0.687	1	0
16634	2578966	eutS	3.705	0.562	1	0
8154	2579109	eutS	3.522	0.616	1	0
19336	2579182	eutS	2.402	0.705	1	0
21779	2579450	tnpA_3	1.254	0.800	0	0
4170	2579921	tnpA_3	1.166	0.744	0	0
5512	2580020	tnpA_3, maeB	1.037	1.162	0	0
12696	2580449	maeB	0.948	0.916	0	0
12372	2580680	maeB	1.074	1.173	0	0
15907	2580784	maeB	1.074	1.262	0	0
5401	2580960	maeB	1.294	1.176	0	0
10661	2581271	maeB	0.687	0.809	0	0

7278	2581427	maeB	0.966	1.056	0	0
19590	2581679	maeB	0.955	0.963	0	0
9095	2581888	maeB	1.149	1.162	0	0
8438	2582238	maeB, talA	0.911	0.896	0	0
9532	2582392	maeB, talA	0.593	0.706	0	0
12724	2582488	maeB, talA	0.757	0.680	0	0
8215	2582801	talA	1.066	0.681	0	0
18320	2583140	talA, tktB	1.067	0.698	0	0
10124	2583244	talA, tktB	1.112	0.699	0	0
20613	2583624	tktB	0.868	0.760	0	0
2533	2583706	tktB	1.096	0.779	0	0
4468	2584127	tktB	1.452	0.899	0	0
20653	2584199	tktB	1.300	0.734	0	0
15172	2584502	tktB	1.044	0.783	0	0
19759	2584654	tktB	1.585	0.850	0	0
19133	2584764	tktB	1.014	0.791	0	0
4746	2585147	tktB	1.635	1.377	0	0
2793	2585410	tktB	1.132	1.560	0	0
22004	2585594	STM2475	1.330	1.559	0	0
9353	2585876	STM2475	1.454	1.419	0	0
851	2586090	STM2475, ypfG	1.199	1.104	0	0
11389	2586217	STM2475, ypfG	1.133	0.914	0	0
9804	2586547	ypfG	0.993	0.812	0	0
9428	2586825	ypfG	1.348	1.062	0	0
4382	2587013	ypfG	1.355	1.013	0	0
11364	2587299	ypfG, yffH	1.281	0.923	0	0
1393	2587462	yffH, STM2478	1.298	0.748	0	0
22056	2587745	yffH, STM2478	1.746	0.876	0	0
22232	2587846	yffH, STM2478	1.528	0.724	0	0
20756	2588062	yffH, STM2478	2.188	0.689	0	0
9479	2588442	STM2478	2.437	0.720	0	0
13130	2588524	STM2478	2.498	0.721	0	0
17308	2588666	STM2478	1.131	0.534	0	0
18040	2588992	STM2478	1.647	0.738	0	0
21195	2589063	STM2478	1.647	0.819	0	0
20697	2589502		1.520	0.746	0	0

14074	2589592	aegA	1.286	0.794	0	0
8004	2589779	aegA	1.275	0.828	0	0
7407	2590031	aegA	1.124	0.628	0	0
2573	2590352	aegA	1.460	0.715	0	0
12441	2590436	aegA	1.653	0.707	0	0
16564	2590823	aegA	1.435	0.632	0	0
13653	2590929	aegA	1.833	0.881	0	0
5662	2591256	aegA	1.343	0.671	0	0
6850	2591592	aegA, narQ	1.351	0.673	0	0
3511	2591665	aegA, narQ	1.455	0.661	0	0
10024	2591778	aegA, narQ	1.096	0.696	0	0
22128	2592115	narQ	1.232	0.795	0	0
4161	2592529	narQ	1.269	0.820	0	0
7618	2592683	narQ	1.128	0.719	0	0
19533	2592784	narQ	1.136	0.731	0	0
2088	2592891	narQ	1.497	0.861	0	0
3481	2593412	narQ, acrD	1.262	0.876	0	0
13994	2593586	acrD	1.532	0.876	0	0
15153	2593836	acrD	1.444	0.787	0	0
3811	2594046	acrD	1.334	0.804	0	0
3127	2594218	acrD	1.290	0.745	0	0
12389	2594589	acrD	1.237	0.755	0	0
16104	2594764	acrD	1.217	0.868	0	0
3547	2594878	acrD	0.972	0.681	0	0
16955	2595189	acrD	1.242	0.956	0	0
8733	2595398	acrD	1.207	0.986	0	0
7612	2595568	acrD	1.297	0.783	0	0
5881	2595872	acrD	0.645	0.591	0	0
2885	2595974	acrD	1.411	0.852	0	0
13318	2596145	acrD	0.936	0.653	0	0
7038	2596464	acrD	0.908	0.728	0	0
7126	2596665	acrD	1.346	1.004	0	0
21798	2596871	yffB	1.706	0.822	0	0
1714	2597156	yffB	1.744	0.830	0	0
4688	2597400	yffB, dapE	1.709	1.036	0	0
11446	2597641	yffB, dapE	0.996	1.231	0	0

12603	2597792	dapE	1.219	1.195	0	0
13214	2598158	dapE	0.878	1.062	0	0
11807	2598449	dapE, STM2484	0.939	0.999	0	0
7561	2598596	dapE, STM2484	1.078	1.094	0	0
13281	2598867	STM2484	0.742	0.926	0	0
11516	2598988	STM2484	0.992	1.637	0	0
16888	2599265	ypfI	0.548	1.110	0	0
2715	2599579	ypfI	1.516	1.113	0	0
12413	2599662	ypfI	0.425	0.699	0	0
8183	2599877	ypfI	1.061	1.099	0	0
18830	2600146	ypfI	1.651	1.233	0	0
11408	2600217	ypfI	0.601	0.540	0	0
18406	2600654	ypfI	0.515	0.680	0	0
6960	2600842	ypfI	0.496	0.751	0	0
12285	2601080	ypfI, STM2486	1.014	1.284	0	0
6760	2601154	ypfI, STM2486	0.922	0.949	0	0
5366	2601494	ypfI, STM2486	0.994	1.136	0	0
7436	2601712	STM2486	1.093	1.203	0	0
262	2601972	STM2486	1.161	1.291	0	0
7794	2602249	STM2486, purC	0.511	0.781	0	0
14063	2602373	STM2486, purC	0.925	1.188	0	0
3321	2602631	purC	0.901	1.243	0	0
18017	2602787	purC	0.920	1.464	0	0
10557	2602901	purC	0.846	1.426	0	0
1188	2603041	purC, nlpB	0.813	1.896	0	1
10641	2603537	nlpB	0.693	2.408	0	1
8857	2603621	nlpB	0.738	2.508	0	1
7266	2604005	nlpB	0.585	1.897	0	0
6289	2604170	nlpB, dapA	0.686	2.394	0	1
17787	2604407	nlpB, dapA	0.628	2.521	0	1
5818	2604823	dapA, gcvR	0.632	3.088	0	1
13905	2604925	dapA, gcvR	0.621	2.766	0	1
801	2605034	dapA, gcvR	0.654	2.125	0	1
19713	2605121	dapA, gcvR	0.785	2.031	0	1
2392	2605577	gcvR, bcp	0.819	1.434	0	0
8838	2605884	bcp	0.663	1.307	0	0

14722	2605959	bcp	0.688	1.527	0	0
13066	2606050	bcp	0.539	0.995	0	0
2326	2606492	STM2492	0.957	1.534	0	0
15251	2606773	STM2492, perM	0.535	0.800	0	0
4972	2606896	STM2492, perM	1.053	1.147	0	0
22006	2607103	perM	1.088	1.314	0	0
9621	2607423	perM	0.994	1.179	0	0
9546	2607735	perM, STM2494	0.811	1.213	0	0
19819	2607927	perM, STM2494	0.872	1.195	0	0
14861	2608215	STM2494	0.672	0.945	0	0
15823	2608327	STM2494	0.995	1.219	0	0
2998	2608546	STM2494	0.927	0.992	0	0
20563	2608817	STM2494	1.212	1.259	0	0
13506	2609015	STM2494, yfgD	1.031	1.248	0	0
20701	2609155	STM2494, yfgD	1.107	1.391	0	0
18799	2609395	STM2494, yfgD	0.870	1.512	0	0
18404	2609658	yfgD	0.785	1.849	0	1
16813	2609857	yfgE	0.696	2.355	0	1
3397	2610173	yfgE	0.713	2.263	0	1
17236	2610416	yfgE	0.707	2.066	0	1
16684	2610557	yfgE	0.655	2.063	0	1
21831	2610823	yfgE, uraA	0.960	1.533	0	0
17411	2611069	uraA	1.211	1.042	0	0
11689	2611164	uraA	0.984	0.989	0	0
18570	2611609	uraA	0.459	0.529	0	0
9610	2611745	uraA	1.294	0.997	0	0
1975	2611938	uraA	0.668	0.443	0	0
5602	2612296	uraA, upp	0.921	1.214	0	0
3221	2612445	upp	0.634	1.186	0	0
14678	2612617	upp, purM	0.507	1.349	0	0
2217	2612708	upp, purM	0.805	1.656	0	0
20782	2612813	upp, purM	0.921	1.707	0	0
9678	2613046	upp, purM	0.533	0.555	0	0
13444	2613297	purM	0.817	0.922	0	0
10503	2613747	purM, purN	1.163	1.161	0	0
7689	2614074	purM, purN	0.650	0.808	0	0

15235	2614369	purN	0.970	1.361	0	0
12618	2614593	purN, ppk	0.948	1.140	0	0
6993	2614817	ppk	1.160	0.928	0	0
20765	2614936	ppk	1.471	1.072	0	0
15676	2615039	ppk	1.413	1.279	0	0
1023	2615349	ppk	1.204	1.068	0	0
15490	2615548	ppk	0.972	1.051	0	0
2349	2615898	ppk	1.056	0.967	0	0
3826	2616034	ppk	1.033	0.985	0	0
13590	2616223	ppk	1.293	1.095	0	0
1652	2616505	ppk	1.229	1.116	0	0
14925	2616741	ppk, ppx	1.015	1.003	0	0
2901	2616971	ppk, ppx	0.828	0.845	0	0
12478	2617187	ppx	0.526	0.541	0	0
16795	2617305	ppx	1.471	1.198	0	0
3380	2617729	ppx	1.136	1.203	0	0
9921	2617943	ppx	1.223	1.116	0	0
2850	2618130	ppx	1.302	1.109	0	0
20269	2618280	ppx	1.421	1.033	0	0
9721	2618619	STM2503	1.521	1.085	0	0
19289	2618739	STM2503	1.753	0.904	0	0
20886	2619112	STM2503	1.610	0.826	0	0
9680	2619194	STM2503	1.603	0.794	0	0
11148	2619379	STM2503	1.665	0.645	0	0
10228	2619640	STM2503	2.967	0.633	1	0
1651	2619885	STM2503	4.304	0.604	1	0
8536	2620159	STM2503	3.948	0.540	1	0
18031	2620264	STM2503	4.391	0.652	1	0
13095	2620502	STM2503	2.556	0.767	1	0
10993	2620830	STM2503, STM2506	1.482	0.649	0	0
8081	2621055	STM2503, STM2504	1.835	0.920	0	0
18480	2621256	STM2504, STM2506	1.324	1.165	0	0
3687	2621549	STM2505, STM2507	1.058	1.203	0	0
15858	2621743	STM2505, STM2507	1.339	0.730	0	0
15106	2622030	STM2507, STM2508	1.278	0.664	0	0
21633	2622140	STM2507, STM2508	1.549	0.637	0	0

8468	2622215	STM2508	1.281	0.638	0	0
12377	2622745	STM2509	1.077	0.772	0	0
7920	2622938	STM2509, guaA	0.786	1.179	0	0
12451	2623037	STM2509, guaA	0.746	1.121	0	0
17270	2623399	guaA	0.895	1.690	0	0
7079	2623506	guaA	0.935	1.618	0	0
1240	2623843	guaA	0.917	1.310	0	0
4997	2624029	guaA	0.918	1.546	0	0
10538	2624324	guaA	1.014	1.919	0	1
14964	2624530	guaA, guaB	0.876	2.065	0	1
12721	2624615	guaA, guaB	0.894	2.054	0	1
12656	2624762	guaA, guaB	0.783	1.723	0	0
16687	2625096	guaB	0.719	1.380	0	0
20540	2625280	guaB	0.785	1.569	0	0
6208	2625487	guaB	0.581	1.551	0	0
362	2625889	guaB, xseA	0.714	3.866	0	1
2659	2626060	guaB, xseA	0.612	3.139	0	1
7326	2626141	guaB, xseA	0.685	2.032	0	0
12209	2626414	xseA	0.975	1.845	0	0
9681	2626540	xseA	0.662	1.162	0	0
4290	2627057	xseA	1.104	1.422	0	0
6446	2627164	xseA	1.183	1.519	0	0
9486	2627497		1.138	1.704	0	0
12163	2627578	shdA	1.049	1.532	0	0
4340	2627970	shdA	1.157	0.806	0	0
9929	2628050	shdA	0.920	0.694	0	0
4102	2628316	shdA	0.919	0.661	0	0
16766	2628424	shdA	0.852	0.636	0	0
12148	2628709	shdA	1.341	0.689	0	0
16433	2629029	shdA	1.085	0.661	0	0
8507	2629223	shdA	1.225	0.678	0	0
17360	2629469	shdA	1.791	0.758	0	0
8122	2629557	shdA	1.738	0.767	0	0
12847	2629688	shdA	1.256	0.669	0	0
1415	2630171	shdA	0.658	0.417	0	0
15750	2630300	shdA	0.890	0.594	0	0

1758	2630417	shdA	0.874	0.657	0	0
20146	2630851	shdA	2.017	0.800	0	0
4126	2631040	shdA	1.707	0.819	0	0
7230	2631275	shdA	1.354	0.697	0	0
4451	2631590	shdA	1.155	0.593	0	0
4833	2631752	shdA	0.742	0.647	0	0
14008	2632069	shdA	1.672	0.815	0	0
22255	2632156	shdA	1.487	0.887	0	0
15470	2632400	shdA	1.388	0.864	0	0
2023	2632662	shdA	1.615	0.786	0	0
14343	2632859	shdA	0.815	0.650	0	0
8288	2633137	shdA	1.801	0.697	0	0
20965	2633258	shdA	2.465	0.752	1	0
16103	2633589	shdA	4.127	0.540	1	0
1224	2633723	shdA	7.095	0.580	1	0
7518	2633843	shdA	6.278	0.505	1	0
12505	2634295		3.833	0.645	1	0
19654	2634550	ratB	2.545	0.721	1	0
20903	2634818	ratB	1.993	0.835	0	0
9781	2634890	ratB	1.669	0.779	0	0
3810	2635060	ratB	0.922	0.644	0	0
22111	2635403	ratB	1.381	1.044	0	0
21606	2635526	ratB	1.372	1.043	0	0
10412	2635790	ratB	1.294	1.015	0	0
8675	2636133	ratB	1.250	0.798	0	0
4727	2636264	ratB	1.227	0.824	0	0
11170	2636357	ratB	1.143	0.783	0	0
2510	2636653	ratB	0.896	0.626	0	0
18693	2636993	ratB	0.916	0.734	0	0
22164	2637258	ratB	1.601	1.403	0	0
15591	2637413	ratB	1.677	1.363	0	0
10385	2637752	ratB	1.260	1.034	0	0
12216	2637846	ratB	1.137	0.960	0	0
7053	2638094	ratB	1.182	1.176	0	0
13908	2638370	ratB	1.078	1.016	0	0
412	2638625	ratB	0.871	0.928	0	0

19525	2638796	ratB	0.779	0.603	0	0
5027	2639119	ratB	1.519	1.117	0	0
17599	2639366	ratB	1.620	1.196	0	0
12774	2639437	ratB	1.496	1.277	0	0
20743	2639753	ratB	1.564	1.088	0	0
15444	2639926	ratB	0.550	0.734	0	0
2590	2640080	ratB	0.995	0.998	0	0
669	2640389	ratB	1.155	0.895	0	0
10988	2640573	ratB	0.977	1.117	0	0
19910	2640860	ratB	0.361	0.636	0	0
6545	2641129	ratB	1.289	1.154	0	0
235	2641263	ratB	0.946	0.970	0	0
10187	2641524	ratB	1.063	1.512	0	0
8346	2641708	ratB	1.275	1.811	0	0
17467	2641955	ratB, ratA	1.235	1.561	0	0
2055	2642103	ratB, ratA	1.333	1.292	0	0
17424	2642363	ratA	1.528	0.945	0	0
15782	2642759	ratA	0.523	0.479	0	0
2488	2642966	ratA	1.484	0.799	0	0
19872	2643037	ratA	1.215	0.886	0	0
15218	2643376	ratA	0.918	0.842	0	0
14184	2643654	ratA	1.092	1.000	0	0
20305	2643908	ratA	1.053	0.847	0	0
2880	2644065	ratA	0.799	0.718	0	0
8841	2644235	ratA	0.726	0.683	0	0
6500	2644557	ratA	1.344	0.783	0	0
5737	2644628	ratA	0.752	0.526	0	0
10691	2644909	ratA	1.953	1.111	0	0
2597	2645179	ratA	1.317	0.905	0	0
18679	2645291	ratA	0.932	0.612	0	0
10951	2645531	ratA	1.106	0.752	0	0
6391	2645904	ratA	1.031	0.568	0	0
21159	2646147	ratA	0.602	0.376	0	0
19055	2646228	ratA	0.806	0.537	0	0
15502	2646405	ratA	1.212	0.566	0	0
20915	2646802	ratA	1.472	0.876	0	0

2263	2646876	ratA	1.602	0.857	0	0
4273	2647242	ratA	1.012	0.701	0	0
6457	2647418	ratA	1.639	0.731	0	0
20750	2647575	ratA, sinI	0.975	0.615	0	0
11372	2647869	ratA, sinI	0.854	0.608	0	0
6510	2648122	sinI	1.136	0.586	0	0
19212	2648399	sinI	0.651	0.527	0	0
20754	2648518	sinI	1.097	0.651	0	0
3400	2648871	sinI, sinH	1.250	0.723	0	0
18992	2648971	sinI, sinH	1.309	0.586	0	0
9942	2649200	sinH	1.464	0.705	0	0
383	2649589	sinH	1.362	0.628	0	0
2405	2649741	sinH	1.644	0.709	0	0
16921	2649889	sinH	1.761	0.639	0	0
8503	2650124	sinH	2.469	0.606	1	0
19904	2650459	sinH	4.476	0.645	1	0
10448	2650631	sinH	5.243	0.657	1	0
4958	2650784	sinH	5.583	0.584	1	0
9811	2651148	sinH, yfgJ	4.951	0.682	1	0
11175	2651346	yfgJ	2.532	0.733	1	0
8527	2651597	yfgJ, engA	1.838	0.889	0	0
9908	2651780	yfgJ, engA	1.088	0.757	0	0
16752	2651966	engA	1.533	1.105	0	0
20629	2652061	engA	1.425	1.099	0	0
2565	2652237	engA	0.834	0.771	0	0
21732	2652317	engA	1.315	1.219	0	0
10914	2652903	engA	1.049	1.353	0	0
4844	2653168	engA, yfgL	0.687	1.153	0	0
7130	2653349	engA, yfgL	0.685	1.449	0	0
14169	2653476	yfgL	0.857	1.782	0	0
14815	2653907	yfgL	0.531	1.214	0	0
7697	2654002	yfgL	0.866	1.896	0	1
8894	2654223	yfgL, yfgM	0.863	2.086	0	1
15340	2654512	yfgL, yfgM	0.579	1.156	0	0
18926	2654794	yfgM	0.789	1.511	0	0
14770	2654940	yfgM, hisS	0.890	1.552	0	0

11135	2655162	yfgM, hisS	0.932	1.661	0	0
1248	2655307	hisS	0.636	1.400	0	0
15419	2655536	hisS	0.800	1.722	0	0
13294	2655919	hisS	0.707	1.876	0	0
3921	2656125	hisS	0.759	1.600	0	0
9346	2656343	hisS, gcpE	0.598	1.520	0	0
8301	2656447	hisS, gcpE	0.717	1.663	0	0
779	2656797	gcpE	0.790	1.613	0	0
19206	2657011	gcpE	0.729	1.367	0	0
649	2657226	gcpE	0.784	1.679	0	0
19389	2657327	gcpE	0.729	1.713	0	0
10480	2657539	gcpE, yfgA	0.858	1.917	0	0
2006	2657926	yfgA	0.755	1.860	0	0
4106	2658029	yfgA	0.345	0.859	0	0
9211	2658382	yfgA	0.732	2.321	0	1
1618	2658546	yfgA	0.752	1.998	0	1
7574	2658830	yfgA, yfgB	0.836	1.467	0	0
21299	2658904	yfgB	0.803	1.367	0	0
11898	2659216	yfgB	0.802	1.163	0	0
17582	2659294	yfgB	0.765	1.303	0	0
11727	2659827	yfgB	0.750	1.941	0	0
10814	2659927	yfgB	0.707	1.719	0	0
5153	2660016	yfgB	1.150	2.207	0	0
21971	2660458	ndk	1.049	1.665	0	0
413	2660594	ndk, STM2527	1.086	1.276	0	0
945	2660678	ndk, STM2527	1.126	1.561	0	0
21370	2661146	STM2527	1.361	1.257	0	0
11044	2661257	STM2527	0.874	0.825	0	0
15399	2661463	STM2527, STM2528	1.375	0.811	0	0
1201	2661657	STM2527, STM2528	1.335	0.765	0	0
9441	2662036	STM2528	0.983	0.669	0	0
19800	2662299	STM2528, STM2529	1.109	0.616	0	0
17255	2662465	STM2528, STM2529	1.352	0.860	0	0
18475	2662659	STM2528, STM2529	1.139	0.654	0	0
13419	2662862	STM2529	1.598	1.032	0	0
16940	2663219	STM2529, STM2530	1.160	0.700	0	0

15982	2663340	STM2529, STM2530	1.503	0.797	0	0
9154	2663663	STM2530	1.182	0.610	0	0
1088	2663781	STM2530	1.198	0.697	0	0
19439	2663887	STM2530	1.332	0.769	0	0
20562	2664332	STM2530	1.628	0.725	0	0
1636	2664532	STM2530	2.630	0.706	1	0
15766	2664796	STM2530	4.331	0.688	1	0
21069	2664927	STM2530	4.299	0.612	1	0
8630	2665080	STM2530	5.556	0.626	1	0
21789	2665266	STM2530	7.186	0.637	1	0
9522	2665456	STM2530, pbpC	4.796	0.659	1	0
4897	2665851	pbpC	1.264	0.546	0	0
17476	2666039	pbpC	2.314	1.175	0	0
5920	2666181	pbpC	1.632	0.940	0	0
1267	2666522	pbpC	1.535	0.914	0	0
12759	2666758	pbpC	1.309	1.013	0	0
10427	2666935	pbpC	1.358	1.054	0	0
20556	2667273	pbpC	1.530	1.605	0	0
22210	2667498	pbpC	1.305	1.912	0	0
5923	2667730	pbpC	0.630	0.942	0	0
15127	2667948	pbpC, STM2532	0.909	1.143	0	0
9553	2668159	pbpC, STM2532	1.494	1.836	0	0
13044	2668440	STM2532	0.974	1.058	0	0
12099	2668524	STM2532	1.317	1.386	0	0
5959	2668808	STM2532	1.351	1.368	0	0
11383	2669133	STM2532	1.160	1.146	0	0
2209	2669358	STM2532	1.391	1.326	0	0
2233	2669576	STM2532	0.877	1.014	0	0
1980	2669670	STM2532	1.067	1.200	0	0
2000	2669910	STM2532	0.900	0.862	0	0
15038	2670229	STM2532	0.412	0.608	0	0
3356	2670389	STM2532	1.193	1.152	0	0
15921	2670637	STM2532	0.857	0.899	0	0
326	2670786	STM2532	1.002	1.036	0	0
12537	2671026	STM2532	0.980	1.016	0	0
11673	2671357	STM2532	1.291	1.313	0	0

8938	2671583	STM2532	1.126	0.992	0	0
14576	2671837	STM2532	1.323	1.195	0	0
10270	2672025	STM2532	1.122	1.083	0	0
16959	2672226	STM2532	1.104	1.208	0	0
6831	2672397	STM2532	1.133	1.129	0	0
7475	2672714	STM2532, sseA	1.239	1.226	0	0
10593	2672827	STM2532, sseA	1.465	1.144	0	0
3664	2673144	sseA	1.410	1.054	0	0
9108	2673363	sseA	1.632	0.852	0	0
11461	2673516	sseA, STM2534	2.355	0.911	1	0
12387	2673787	STM2534	4.436	0.815	1	0
9932	2674045	STM2534	4.492	0.683	1	0
1726	2674286	STM2534	3.228	0.790	1	0
16848	2674530	STM2534	3.113	1.013	1	0
19385	2674829		0.660	0.709	0	0
6766	2674900		1.323	1.630	0	0
20693	2675058		0.862	1.052	0	0
19139	2675136		0.816	0.948	0	0
15898	2675497	sseB	1.286	1.216	0	0
20405	2675792	sseB	1.170	1.415	0	0
7906	2675962	sseB	1.282	1.337	0	0
10556	2676207	sseB, pepB	0.586	0.849	0	0
21733	2676566	pepB	1.344	1.328	0	0
4967	2676739	pepB	1.302	1.295	0	0
16084	2676917	pepB	1.048	1.185	0	0
5102	2677264	pepB	1.181	1.371	0	0
20687	2677367	pepB	1.142	1.541	0	0
21887	2677746	pepB, yfhJ	0.876	1.839	0	0
21170	2677845	yfhJ, fdx	0.754	1.745	0	0
18785	2678150	yfhJ, fdx	0.647	1.030	0	0
7076	2678267	yfhJ, fdx	0.718	1.307	0	0
6978	2678464	fdx, hscA	0.962	1.308	0	0
13469	2678723	hscA	0.719	1.173	0	0
11429	2678819	hscA	0.880	1.407	0	0
3007	2679356	hscA	0.727	1.058	0	0
18234	2679534	hscA	1.121	1.817	0	0

18042	2679690	hscA	0.646	1.198	0	0
14821	2679864	hscA	0.769	1.466	0	0
18151	2680033	hscA, hscB	0.865	1.993	0	0
5333	2680168	hscA, hscB	0.558	1.115	0	0
11454	2680544	hscB	0.738	2.264	0	0
3793	2680792	hscB, yfhF	0.688	1.674	0	0
4216	2681074	yfhF	0.653	1.812	0	1
6863	2681343	yfhF, nifU	0.702	1.727	0	1
15033	2681512	yfhF, nifU	0.708	1.544	0	0
15523	2681648	nifU, nifS	0.728	1.505	0	0
4795	2681879	nifU, nifS	0.679	1.570	0	0
5831	2682122	nifS	0.605	1.325	0	0
16561	2682280	nifS	1.058	1.029	0	0
1371	2682722	nifS	0.882	1.906	0	0
4187	2682904	nifS, yfhP	0.787	1.612	0	0
12037	2683017	nifS, yfhP	0.906	1.831	0	0
1705	2683281	yfhP	1.250	1.908	0	0
19074	2683466	yfhP	0.756	1.487	0	0
3130	2683584	yfhP, STM2545	0.937	1.761	0	1
12415	2683897	STM2545	0.878	2.150	0	1
21028	2684277	STM2545, suhB	0.616	2.985	0	1
18425	2684582	STM2545, suhB	0.560	2.023	0	1
245	2684699	STM2545, suhB	0.549	2.538	0	1
6085	2684902	suhB, STM2547	0.790	1.561	0	0
2898	2685013	suhB, STM2547	0.443	0.647	0	0
10902	2685405	STM2547	0.696	0.651	0	0
4283	2685670	STM2547	0.958	0.615	0	0
8461	2685915	STM2547	1.987	0.731	0	0
5880	2686143	STM2547, asrA	4.102	0.691	1	0
8001	2686288	asrA	4.116	0.677	1	0
16153	2686428	asrA	4.021	0.519	1	0
16348	2686652	asrA	3.824	0.609	1	0
10997	2686844	asrA	4.692	0.593	1	0
19832	2687151	asrA, asrB	1.523	0.463	0	0
661	2687471	asrB	1.143	0.728	0	0
18905	2687696	asrB	1.101	0.679	0	0

6369	2687874	asrB, asrC	1.322	0.701	0	0
20666	2688118	asrB, asrC	1.527	0.797	0	0
8226	2688297	asrC	1.584	0.930	0	0
11104	2688561	asrC	1.508	0.956	0	0
6865	2688707	asrC	1.022	0.808	0	0
2057	2689071	asrC	1.172	0.868	0	0
7509	2689162	asrC	1.321	1.112	0	0
13134	2689563	STM2551	1.080	0.936	0	0
16890	2689819	STM2551	0.525	0.665	0	0
3020	2689940	STM2551	0.813	0.937	0	0
16623	2690149	STM2551	0.910	1.031	0	0
21260	2690294	STM2551, STM2552	1.429	1.184	0	0
21272	2690489	STM2551, STM2552	1.204	0.802	0	0
8331	2690894	STM2552, csiE	1.102	1.411	0	0
16644	2691017	STM2552, csiE	1.024	1.460	0	0
8508	2691187	STM2552, csiE	0.950	1.255	0	0
14385	2691493	csiE	1.007	1.182	0	0
8371	2691805	csiE	1.220	1.247	0	0
10113	2692054	csiE	0.986	0.907	0	0
9418	2692153	csiE	0.388	0.572	0	0
16668	2692424	hcaT	0.645	0.991	0	0
6333	2692555	hcaT	1.170	1.304	0	0
11055	2692767	hcaT	0.890	1.224	0	0
10453	2693005	hcaT	0.944	1.241	0	0
4078	2693395	hcaT	1.513	1.810	0	0
15027	2693470	hcaT	0.750	1.875	0	1
9566	2693855	hcaT, glyA	0.744	1.961	0	1
16308	2693933	glyA	0.819	2.090	0	1
19117	2694367	glyA	0.655	1.629	0	0
19850	2694459	glyA	0.814	1.889	0	0
1889	2694821	glyA, hmpA	0.740	1.589	0	0
3853	2694920	glyA, hmpA	0.668	1.152	0	0
11820	2695080	glyA, hmpA	0.761	0.989	0	0
14862	2695156	glyA, hmpA	0.807	0.821	0	0
8311	2695333	glyA, hmpA	0.850	0.820	0	0
1906	2695621	hmpA	1.317	0.848	0	0

20365	2695834	hmpA	1.174	0.864	0	0
10888	2696371	hmpA, cadC	0.776	0.650	0	0
20732	2696595	cadC	0.815	1.034	0	0
20149	2696811	cadC	0.733	0.791	0	0
14149	2696920	cadC	1.015	0.863	0	0
7615	2697207	cadC	1.265	0.674	0	0
22277	2697504	cadC	2.221	0.590	0	0
21648	2697734	cadC	2.959	0.493	1	0
14359	2697906	cadC	3.043	0.623	1	0
548	2698109	cadC, cadB	2.563	0.570	1	0
2118	2698415	cadB	3.020	0.518	1	0
3902	2698500	cadB	3.380	0.618	1	0
6347	2698813	cadB	3.436	0.624	1	0
6228	2699062	cadB	1.471	0.605	0	0
435	2699228	cadB	1.655	0.877	0	0
14054	2699452	cadB, cadA	1.447	0.760	0	0
14433	2699651	cadB, cadA	1.120	0.660	0	0
9728	2699919	cadA	0.855	0.596	0	0
18424	2700001	cadA	1.412	0.658	0	0
11112	2700109	cadA	1.639	0.704	0	0
12269	2700208	cadA	1.435	0.621	0	0
12002	2700867	cadA	1.104	0.620	0	0
6535	2701107	cadA	0.781	0.522	0	0
15392	2701214	cadA	1.201	0.665	0	0
9042	2701363	cadA	1.180	0.685	0	0
12433	2701726	cadA, yjdL	1.127	0.712	0	0
392	2702058	cadA, yjdL	2.131	1.814	0	0
10127	2702236	yjdL	1.028	0.720	0	0
16129	2702395	yjdL	0.974	0.518	0	0
21577	2702670	yjdL	1.352	0.879	0	0
13847	2702791	yjdL	1.248	0.950	0	0
13874	2703001	yjdL	1.362	1.122	0	0
8273	2703443	yjdL	0.921	2.315	0	1
13384	2703643	glnB	0.834	2.048	0	1
20752	2703760	glnB	0.828	1.662	0	1
11875	2703957	glnB	0.987	1.941	0	1

5778	2704046	glnB, yfhA	1.015	2.025	0	1
18685	2704162	glnB, yfhA	0.986	1.757	0	0
13962	2704372	glnB, yfhA	1.100	1.421	0	0
21546	2704487	yfhA	1.142	1.316	0	0
17078	2705174	yfhA	0.803	1.153	0	0
8792	2705245	yfhA	0.875	1.421	0	0
4708	2705532	yfhA, yfhG	1.019	1.474	0	0
12683	2705617	yfhA, yfhG	0.934	1.676	0	0
8996	2706139	yfhG	0.836	1.510	0	0
4005	2706326	yfhG, yfhK	0.881	1.443	0	0
21939	2706403	yfhG, yfhK	0.971	1.411	0	0
12878	2706640	yfhK	0.739	1.005	0	0
14326	2706908	yfhK	1.163	1.309	0	0
13729	2707008	yfhK	1.004	1.125	0	0
9975	2707336	yfhK	0.799	1.179	0	0
9168	2707593	yfhK	0.738	3.019	0	1
16748	2707819	yfhK	0.542	2.165	0	1
8357	2707909	yfhK	0.426	1.583	0	0
7628	2708013	yfhK	0.499	1.705	0	0
5721	2708233	purG	0.808	1.362	0	0
17973	2708433	purG	0.991	1.214	0	0
14327	2709053	purG	1.336	1.381	0	0
8546	2709192	purG	1.119	0.990	0	0
4195	2709363	purG	0.422	0.494	0	0
11864	2709794	purG	1.441	1.234	0	0
7602	2709924	purG	1.432	1.142	0	0
6383	2710174	purG	1.023	0.978	0	0
7059	2710347	purG	1.235	1.194	0	0
11436	2710635	purG	1.016	1.039	0	0
3256	2710728	purG	1.234	1.196	0	0
4235	2710927	purG	1.213	0.993	0	0
4203	2711363	purG	0.806	0.679	0	0
17730	2711446	purG	0.965	0.972	0	0
10193	2711682	purG, STM2566	1.252	1.221	0	0
1440	2712016	purG, STM2566	1.170	1.030	0	0
19268	2712296	purG, STM2566	1.287	1.123	0	0

7581	2712529	purG, yfhD	1.298	1.422	0	0
20825	2712740	yfhD	1.146	1.277	0	0
9800	2712889	yfhD	1.254	1.141	0	0
11216	2713175	yfhD	0.348	0.509	0	0
12951	2713248	yfhD	1.192	0.985	0	0
18736	2713623	yfhD	1.146	1.143	0	0
1470	2713740	yfhD	1.469	0.847	0	0
18537	2713902	yfhD	1.370	1.630	0	0
21431	2714107	yfhC	1.315	1.770	0	0
12541	2714475	yfhC	1.192	1.206	0	0
5769	2714802	yfhC, yfhB	1.284	1.410	0	0
4587	2714876	yfhC, yfhB	0.774	0.833	0	0
14117	2715127	yfhB	1.423	1.177	0	0
12766	2715264	yfhB, STM2570	1.492	0.967	0	0
13263	2715335	yfhB, STM2570	1.069	0.872	0	0
6796	2715782	STM2570	1.432	0.921	0	0
5623	2716106	STM2570	1.156	0.884	0	0
4314	2716373	STM2570	0.984	1.081	0	0
6991	2716556	STM2570, STM2571	1.246	0.971	0	0
19125	2716771	STM2570, STM2571	0.738	0.537	0	0
1738	2716930	STM2570, STM2571	1.493	0.919	0	0
20235	2717056	STM2571	1.241	1.058	0	0
6927	2717419	STM2571, yfhH	1.572	0.856	0	0
14647	2717500	STM2571, yfhH	1.821	0.961	0	0
11983	2717795	STM2571, yfhH	1.463	0.868	0	0
3551	2718028	yfhH	1.355	0.716	0	0
18796	2718444		0.889	0.685	0	0
8375	2718649	STM2573	1.788	0.811	0	0
16344	2718808	STM2573	2.285	0.792	0	0
19747	2719013	STM2573	2.316	0.799	0	0
8860	2719221	STM2573	4.146	0.689	1	0
2955	2719423	STM2573, STM2574	5.665	0.466	1	0
9629	2719581	STM2573, STM2574	4.479	0.515	1	0
9179	2719830	STM2573, STM2574	3.662	0.545	1	0
19494	2720051	STM2574	3.480	0.610	1	0
5804	2720350	STM2574, STM2575	2.632	0.644	1	0

8265	2720585	STM2574, STM2575	2.472	0.479	0	0
14950	2720794	STM2574, STM2575	2.882	0.516	1	0
15924	2721066	STM2575	3.139	0.679	1	0
4461	2721179	STM2575	2.204	0.631	0	0
9020	2721289	STM2575, yfhL	2.098	0.709	0	0
21707	2721501	STM2575, yfhL	1.372	0.815	0	0
8229	2721886	yfhL	1.010	1.176	0	0
8010	2722175	acpS	0.938	1.240	0	0
5391	2722515	acpS, pdxJ	1.229	1.167	0	0
940	2722663	acpS, pdxJ	1.185	1.048	0	0
1092	2722936	pdxJ	0.943	1.201	0	0
13399	2723150	pdxJ	0.888	1.055	0	0
565	2723242	pdxJ, recO	1.226	1.228	0	0
16682	2723604	pdxJ, recO	0.935	1.405	0	0
17611	2723737	recO	0.938	1.720	0	0
17234	2724017	recO, era	0.872	1.813	0	0
9630	2724333	recO, era	0.864	1.343	0	0
3115	2724425	era	0.630	1.201	0	0
5228	2724791	era	0.711	1.307	0	0
14879	2724899	era, rnc	0.606	0.884	0	0
12395	2725059	era, rnc	0.939	1.407	0	0
19218	2725438	rnc	0.899	2.393	0	0
6690	2725511	rnc	0.638	1.647	0	0
10481	2725687	rnc	0.770	1.360	0	0
7070	2726071	lepB	0.740	1.413	0	0
22091	2726379	lepB	0.524	0.844	0	0
555	2726477	lepB	1.379	1.427	0	0
589	2726813	lepB, lepA	0.695	1.401	0	0
14806	2726924	lepB, lepA	0.813	1.470	0	0
19210	2727290	lepA	0.900	1.124	0	0
20572	2727408	lepA	0.603	0.991	0	0
6934	2727650	lepA	0.589	0.974	0	0
14882	2727930	lepA	0.640	0.961	0	0
5685	2728190	lepA	0.893	1.258	0	0
5497	2728424	lepA	1.037	0.954	0	0
2493	2728505	lepA, gogB	1.141	0.893	0	0

3517	2728870	lepA, gogB	2.139	0.566	0	0
19047	2729041	gogB	2.624	0.613	0	0
16962	2729222	gogB	2.421	0.460	0	0
12549	2729566	gogB	1.862	0.473	0	0
19741	2729719	gogB	2.173	0.439	0	0
12907	2729842	gogB	2.359	0.496	0	0
6954	2730168	gogB	2.116	0.480	0	0
10834	2730289	gogB	2.230	0.440	0	0
16818	2730656		2.448	0.578	0	0
5751	2730815		2.341	0.525	0	0
5771	2730921	STM2585	2.517	0.600	0	0
6074	2731093	STM2585	2.519	0.511	0	0
13489	2731458	STM2585	2.209	0.450	0	0
19601	2731772	STM2585	1.822	0.686	0	0
21016	2731924		1.904	0.638	0	0
3818	2732222		1.400	0.544	0	0
6920	2732394		1.642	0.602	0	0
5471	2732521	STM2585A	1.794	0.633	0	0
14846	2732807	STM2585A	2.924	0.432	1	0
21711	2732998	STM2585A	3.304	0.426	1	0
21268	2733258		3.039	0.454	1	0
16384	2733578	STM2586	3.175	0.624	1	0
16745	2733909	STM2586, STM2587	5.997	0.569	1	0
12895	2734018	STM2586, STM2587	4.941	0.536	1	0
8576	2734168	STM2586, STM2587	3.518	0.608	1	0
1568	2734597	STM2587	3.361	0.603	1	0
6928	2734669	STM2587	3.456	0.645	1	0
5779	2734914	STM2587, STM2588	2.136	0.840	0	0
18074	2735150	STM2587, STM2588	1.833	0.946	0	0
7544	2735433	STM2588	1.837	0.872	0	0
11040	2735597	STM2588	1.680	0.798	0	0
10241	2735804	STM2588	1.581	0.669	0	0
1053	2735995	STM2588	1.723	0.686	0	0
7391	2736212	STM2588	1.205	0.680	0	0
21590	2736575	STM2588	0.657	0.635	0	0
12722	2736828	STM2588	2.002	0.694	0	0

19766	2737068	STM2588	2.375	0.683	0	0
10933	2737258	STM2588	2.024	0.697	0	0
11594	2737369	STM2588	2.113	0.707	0	0
6195	2737553	STM2589	1.929	0.798	0	0
3173	2737792	STM2589	1.731	0.714	0	0
3496	2738169	STM2589	1.479	0.735	0	0
11397	2738292	STM2589	1.886	0.712	0	0
16480	2738412	STM2589	1.992	0.729	0	0
1943	2738882	STM2589	0.708	0.658	0	0
943	2739139	STM2589	0.413	0.504	0	0
18072	2739319	STM2589	1.107	0.719	0	0
17830	2739491	STM2589	1.296	0.830	0	0
10808	2739654	STM2589	1.448	0.673	0	0
2586	2739938	STM2589	0.657	0.589	0	0
13504	2740173	STM2589	1.679	0.706	0	0
3222	2740287	STM2589	1.094	0.497	0	0
17161	2740467	STM2589	1.677	0.673	0	0
8315	2740863	STM2589, STM2590	0.315	0.501	0	0
19585	2740997	STM2589, STM2590	0.672	0.411	0	0
14211	2741266	STM2590	0.350	0.390	0	0
7957	2741501	STM2590, STM2591	1.543	0.550	0	0
3682	2741673	STM2590, STM2591	0.725	0.708	0	0
1046	2741996	STM2591	0.967	0.537	0	0
19813	2742146	STM2591, STM2592	1.343	0.824	0	0
22249	2742287	STM2591, STM2592	1.660	1.257	0	0
10603	2742707	STM2592	1.058	0.862	0	0
18047	2742852	STM2592, STM2593	1.144	1.000	0	0
11722	2743111	STM2592, STM2593	1.014	0.946	0	0
4652	2743218	STM2592, STM2593	0.729	0.699	0	0
16441	2743450	STM2593, STM2594	1.271	0.950	0	0
5533	2743588	STM2593, STM2594	1.199	0.910	0	0
17811	2744136	STM2594	1.367	1.031	0	0
8377	2744228	STM2594	1.336	0.784	0	0
6420	2744433	STM2594	1.234	0.727	0	0
3501	2744689	STM2594	1.730	0.756	0	0
17162	2744887	STM2594	0.995	0.783	0	0

16572	2745088	STM2594	1.852	1.158	0	0
12123	2745311	STM2594	1.776	1.045	0	0
2667	2745553	STM2594	1.306	1.007	0	0
20013	2745876	STM2594	1.169	1.197	0	0
9877	2746045	STM2594	1.144	0.889	0	0
2432	2746318	STM2594, STM2595	1.371	0.583	0	0
4467	2746427	STM2594, STM2595	1.082	0.600	0	0
13056	2746793	STM2595, STM2596	0.897	0.698	0	0
14393	2746945	STM2595, STM2596	1.513	0.760	0	0
8365	2747177	STM2596, STM2597	1.300	0.791	0	0
2514	2747347	STM2596, STM2597	0.822	0.560	0	0
5195	2747742	STM2597, STM2599	0.769	0.572	0	0
18360	2747922	STM2597, STM2599	1.415	1.080	0	0
1301	2748063	STM2597, STM2599	1.573	1.119	0	0
5349	2748280	STM2599, STM2598	1.066	1.889	0	1
6858	2748380	STM2599, STM2598	0.945	2.166	0	1
330	2748819	STM2599	0.849	1.540	0	0
21951	2749057	STM2599	1.060	1.755	0	1
21882	2749236	STM2599	1.116	1.938	0	1
18144	2749562	STM2600	1.050	1.589	0	0
2828	2749829	STM2600	1.014	1.284	0	0
9898	2749928	STM2600	1.040	0.930	0	0
19063	2750000	STM2600	1.051	0.850	0	0
4040	2750237	STM2600, STM2601	1.139	0.763	0	0
15217	2750473	STM2600, STM2601	1.413	0.778	0	0
15408	2750819	STM2601, STM2602	1.597	0.945	0	0
20431	2751139	STM2602, STM2603	1.469	0.819	0	0
10805	2751363	STM2603	1.038	0.585	0	0
4648	2751531	STM2603	1.188	0.754	0	0
6060	2751786	STM2603	1.820	0.692	0	0
333	2751895	STM2603	1.616	3.065	0	0
4225	2752283	STM2603, STM2604	1.242	0.694	0	0
12316	2752354	STM2603, STM2604	1.377	0.883	0	0
3612	2752425	STM2604, STM2605	1.240	0.782	0	0
18161	2752910	STM2605	1.389	0.791	0	0
20055	2753186	STM2605	1.170	0.471	0	0

17136	2753446	STM2605	0.775	0.585	0	0
11724	2753642	STM2605	1.381	0.570	0	0
5979	2753794	STM2605, STM2606	0.656	0.356	0	0
4606	2753949	STM2605, STM2606	1.783	0.724	0	0
3742	2754360	STM2606	1.553	0.881	0	0
1308	2754439	STM2606	1.923	0.952	0	0
10000	2754779	STM2606	0.670	0.804	0	0
13292	2754912	STM2606	1.151	0.708	0	0
7308	2755039	STM2606	1.254	0.944	0	0
21826	2755120	STM2606	1.779	0.971	0	0
4208	2755430	STM2606, STM2607	0.752	0.529	0	0
20085	2755756	STM2606, STM2607	1.284	0.800	0	0
7877	2756089	STM2608	1.630	0.734	0	0
3209	2756210	STM2608	1.181	0.604	0	0
15231	2756483	STM2608	1.009	0.551	0	0
18553	2756645	STM2608	1.349	0.714	0	0
19676	2756968	STM2608	0.460	0.541	0	0
9426	2757124	STM2608	1.097	0.808	0	0
3414	2757376	STM2608	0.988	0.607	0	0
19646	2757604	STM2608, STM2609	1.603	0.689	0	0
17109	2757820	STM2608, STM2609	1.658	0.541	0	0
12228	2758024	STM2609, STM2610	2.581	0.584	1	0
20669	2758152	STM2609, STM2610	2.781	0.585	1	0
3324	2758488	STM2610	2.565	0.461	1	0
11215	2758823		2.764	0.532	1	0
19874	2758945	STM2611	1.988	0.648	0	0
21343	2759292	STM2611	1.296	0.647	0	0
3605	2759391	STM2611	0.701	0.463	0	0
14456	2759625	STM2611, STM2612	1.425	0.671	0	0
12281	2759849	STM2611, STM2612	1.616	0.652	0	0
756	2760139	STM2612, STM2613	2.449	0.639	0	0
11093	2760213	STM2612, STM2613	1.661	0.531	0	0
10553	2760675	STM2614	2.493	0.638	0	0
11868	2760919	STM2614	1.460	0.737	0	0
22284	2761027	STM2614	1.316	0.785	0	0
20499	2761350		1.147	0.855	0	0

13876	2761517		0.562	0.831	0	0
21859	2761774	STM2615	0.731	1.759	0	0
12333	2761885	STM2615	0.579	1.793	0	1
17069	2762126	STM2615, STM2616	0.670	2.182	0	1
5468	2762479	STM2616	0.709	0.961	0	0
9955	2762568	STM2616	0.835	1.605	0	0
9652	2762875	STM2616, STM2617	0.833	2.035	0	0
21290	2763029	STM2616, STM2617	1.084	1.615	0	0
5670	2763240	STM2617	0.826	0.892	0	0
20791	2763524	STM2617	1.307	0.694	0	0
20728	2763595	STM2617, STM2618	1.099	0.930	0	0
4128	2763857	STM2617, STM2618	0.998	0.765	0	0
11463	2764366	STM2619	1.241	0.828	0	0
12658	2764581	STM2619, STM2620	1.119	0.840	0	0
18967	2764770	STM2620	1.240	1.107	0	0
17593	2764869	STM2620	1.102	1.021	0	0
4377	2765168	STM2620	0.975	0.853	0	0
16918	2765347	STM2620	1.040	0.869	0	0
21239	2765662	STM2621	1.127	0.972	0	0
14412	2765758	STM2621	1.064	0.832	0	0
17685	2766049	STM2621, STM2622	0.924	0.751	0	0
7331	2766355	STM2622	1.042	0.665	0	0
12996	2766480	STM2622, STM2623	1.051	0.892	0	0
17762	2766832	STM2623, STM2624	0.930	0.654	0	0
12164	2767028	STM2623, STM2624	1.130	0.619	0	0
20564	2767106	STM2623, STM2624	1.207	0.650	0	0
1996	2767329	STM2624, STM2625	1.078	0.653	0	0
12193	2767627	STM2625	1.108	0.638	0	0
17049	2767803	STM2625	1.154	0.692	0	0
6626	2768177	STM2625, STM2626	0.772	0.637	0	0
21858	2768381	STM2626	1.263	0.702	0	0
10917	2768496	STM2626	1.341	0.686	0	0
19718	2768701	STM2626	1.309	0.915	0	0
16709	2768807	STM2626	1.019	0.914	0	0
8051	2769220	STM2626, STM2627	0.758	0.661	0	0
18001	2769568	STM2627, STM2628	0.777	0.951	0	0

21778	2769815	STM2628, STM2629	0.742	1.037	0	0
18059	2769908	STM2628, STM2629	0.638	1.024	0	0
4535	2770006	STM2628, STM2629	0.714	0.831	0	0
1968	2770366	STM2630, STM2631	1.445	0.714	0	0
6423	2770640	STM2631, STM2632	2.027	0.672	0	0
8535	2770812	STM2631, STM2632	2.022	0.748	0	0
1217	2770987	STM2632	1.938	0.716	0	0
4567	2771133	STM2632	1.659	0.733	0	0
5504	2771606	STM2632	1.774	0.703	0	0
4174	2771830	STM2632	1.083	0.591	0	0
4121	2771941	STM2632	1.526	0.695	0	0
1937	2772082	STM2632	1.417	0.758	0	0
14603	2772326	STM2632	1.790	0.771	0	0
19421	2772481	STM2632	1.350	0.680	0	0
17293	2772988	STM2632	1.528	0.668	0	0
13326	2773161	STM2632	1.384	0.667	0	0
16697	2773433	STM2632, STM2633	1.513	0.679	0	0
9452	2773651	STM2632, STM2633	1.383	0.638	0	0
2331	2773877	STM2633	1.221	0.582	0	0
10021	2773948	STM2633	1.319	0.493	0	0
15427	2774245	STM2633	1.240	0.624	0	0
17822	2774429	STM2633	1.676	0.750	0	0
8965	2774735	STM2633, STM2634	1.404	0.641	0	0
18684	2774819	STM2633, STM2634	1.395	0.602	0	0
897	2775149	STM2634, STM2635	1.297	0.733	0	0
2561	2775453	STM2635	1.183	0.813	0	0
14455	2775666	STM2636	0.946	0.896	0	0
4943	2775798	STM2636	1.158	0.864	0	0
5277	2776106	STM2636	1.187	0.702	0	0
3409	2776222	STM2636	1.150	0.801	0	0
12819	2776466	STM2636	0.785	0.634	0	0
15412	2776803	STM2636	0.643	1.070	0	0
21995	2776895	STM2636	0.575	1.205	0	0
16516	2777113	STM2636	0.595	1.796	0	0
5630	2777522	rseC	0.750	1.233	0	0
8164	2777754	rseC	0.625	1.159	0	0

11776	2777961	rseC, rseB	0.669	1.230	0	0
16427	2778041	rseC, rseB	0.887	2.044	0	0
16713	2778389	rseB	0.756	1.527	0	0
7808	2778565	rseB	0.857	2.394	0	1
19883	2778720	rseB, rseA	0.752	2.539	0	1
2759	2778979	rseB, rseA	0.785	2.108	0	1
11034	2779360	rseA	0.851	1.600	0	0
18048	2779562	rseA, rpoE	0.824	1.291	0	0
16873	2779762	rseA, rpoE	0.686	1.132	0	0
17493	2779904	rpoE	0.839	1.327	0	0
16133	2780180	rpoE, nadB	1.006	0.857	0	0
6202	2780395	rpoE, nadB	1.199	0.819	0	0
20946	2780511	nadB	1.303	0.963	0	0
8606	2780869	nadB	1.220	0.820	0	0
18784	2780972	nadB	0.882	0.659	0	0
11361	2781314	nadB	1.148	1.075	0	0
4198	2781396	nadB	1.029	1.006	0	0
7511	2781502	nadB	1.211	1.460	0	0
5586	2781993	nadB	1.257	1.678	0	0
16109	2782246	yfiC	1.277	1.222	0	0
21955	2782376	yfiC	1.157	1.167	0	0
2474	2782738	yfiC, srmB	1.171	1.002	0	0
16624	2782886	yfiC, srmB	0.968	1.150	0	0
3320	2783175	yfiC, srmB	0.932	1.105	0	0
10555	2783318	srmB	1.217	1.279	0	0
18559	2783558	srmB	0.711	0.820	0	0
18215	2783629	srmB	0.886	0.957	0	0
1436	2783933	srmB	0.869	1.089	0	0
7816	2784266	yfiE	0.536	0.749	0	0
21905	2784462	yfiE	0.467	0.645	0	0
1413	2784571	yfiE	0.900	0.829	0	0
17359	2784743	yfiE	0.854	0.665	0	0
18859	2785010	yfiE, yfiK	1.402	0.741	0	0
2751	2785245	yfiE, yfiK	1.073	0.639	0	0
3175	2785561	yfiE, yfiK	1.189	0.678	0	0
19650	2785883	yfiD	1.240	0.730	0	0

2611	2786093	yfiD	0.936	0.728	0	0
7958	2786337	yfiD, ung	1.159	0.739	0	0
7118	2786418	yfiD, ung	0.490	0.330	0	0
2044	2786508	yfiD, ung	1.010	0.785	0	0
1832	2787030	ung	0.954	1.470	0	0
8949	2787203	ung	0.942	1.779	0	0
3128	2787460	yfiF	0.734	1.536	0	0
9659	2787544	yfiF	0.495	1.066	0	0
8137	2787877	yfiF	0.626	0.998	0	0
7950	2788158	yfiF, trxC	0.806	0.970	0	0
4066	2788404	yfiF, trxC	0.698	0.963	0	0
19729	2788484	yfiF, trxC	0.934	1.358	0	0
21886	2788628	yfiF, trxC	0.989	1.637	0	0
14107	2788970	trxC, yfiP	0.653	1.516	0	0
17909	2789296	yfiP	0.828	1.846	0	0
476	2789573	yfiP, yfiQ	0.900	1.587	0	0
20204	2789812	yfiQ	0.884	1.911	0	1
11147	2789889	yfiQ	0.867	1.993	0	1
14737	2790152	yfiQ	0.900	1.548	0	0
21695	2790354	yfiQ	1.187	1.670	0	0
17056	2790523	yfiQ	1.147	1.393	0	0
15641	2790878	yfiQ	0.833	1.094	0	0
8650	2791144	yfiQ	1.095	1.206	0	0
12778	2791317	yfiQ	1.047	1.261	0	0
5738	2791469	yfiQ	0.488	0.737	0	0
9117	2791819	yfiQ	0.924	1.307	0	0
16583	2792015	yfiQ	0.719	1.280	0	0
21177	2792132	yfiQ	0.820	1.715	0	1
2654	2792504	yfiQ, pssA	0.587	3.127	0	1
4562	2792607	pssA	0.516	2.873	0	1
1548	2792715	pssA	0.463	2.644	0	1
7993	2793117	pssA	0.760	1.417	0	0
12399	2793285	pssA	0.783	1.233	0	0
9454	2793671	pssA, yfiM	0.742	0.974	0	0
11313	2793857	pssA, yfiM	0.429	0.693	0	0
20872	2794032	yfiM	0.419	0.563	0	0

19193	2794366	kgtP	0.944	1.064	0	0
19513	2794576	kgtP	1.065	1.078	0	0
18281	2794828	kgtP	0.946	0.809	0	0
5680	2794931	kgtP	0.924	0.979	0	0
2327	2795176	kgtP	0.837	1.066	0	0
14573	2795448	kgtP	1.098	0.996	0	0
3879	2795534	kgtP	0.929	1.012	0	0
15808	2795670	kgtP	1.007	0.959	0	0
4028	2796073	kgtP, STM2655	0.824	1.620	0	1
10144	2796192	STM2655	0.636	2.197	0	1
17926	2796276	STM2655	0.449	1.870	0	1
14833	2796689	rrfG	0.451	3.948	0	1
13447	2796823	rrfG, rrlG	0.392	4.476	0	1
10315	2797178	rrlG	0.355	5.741	0	1
10314	2797529	rrlG	0.366	5.557	0	1
10061	2797690	rrlG	0.359	5.038	0	1
681	2797829	rrlG	0.306	5.825	0	1
812	2798023	rrlG	0.367	4.657	0	1
513	2798386	rrlG	0.360	5.154	0	1
3424	2798548	rrlG	0.350	5.482	0	1
12251	2798906	rrlG	0.364	5.833	0	1
4342	2799086	rrlG	0.312	3.727	0	1
7608	2799279	rrlG	0.370	4.985	0	1
22065	2799567	rrlG	0.398	4.775	0	1
12492	2799739	rrlG	0.331	4.239	0	1
18607	2799811	rrlG	0.339	3.538	0	1
10756	2800054	rrlG, gltW	0.373	4.344	0	1
17297	2800174	rrlG, gltW	0.294	5.025	0	1
256	2800646	rrsG	0.296	7.133	0	1
12317	2800822	rrsG	0.333	6.515	0	1
14523	2800950	rrsG	0.338	6.128	0	1
13054	2801219	rrsG	0.348	5.926	0	1
815	2801609	rrsG	0.386	4.653	0	1
14545	2801839	rrsG	0.373	3.850	0	1
2862	2802005	rrsG	0.410	2.939	0	1
3087	2802101	clpB	0.420	2.408	0	1

15617	2802355	clpB	0.522	1.894	0	1
4277	2802508	clpB	0.675	1.964	0	1
13449	2802617	clpB	0.659	1.929	0	1
963	2803068	clpB	0.704	2.020	0	1
6475	2803346	clpB	0.806	1.965	0	1
1001	2803466	clpB	0.801	1.923	0	0
8552	2803641	clpB	0.780	1.734	0	1
11631	2804091	clpB	0.992	2.110	0	1
12798	2804196	clpB	0.759	1.982	0	1
2708	2804538	clpB	0.830	2.215	0	1
9876	2804683	clpB	0.725	2.050	0	1
13160	2804809	clpB, yfiH	0.722	2.182	0	1
4834	2805090	clpB, yfiH	0.812	1.515	0	0
16116	2805292	yfiH	0.759	1.390	0	0
11685	2805528	yfiH, rluD	0.722	1.353	0	0
922	2805632	yfiH, rluD	0.767	1.467	0	0
20114	2805915	yfiH, rluD	0.538	1.164	0	0
17208	2806363	rluD, yfiO	0.770	1.342	0	0
22349	2806606	rluD, yfiO	0.667	1.431	0	0
9105	2806817	rluD, yfiO	0.706	1.479	0	0
18467	2806934	rluD, yfiO	0.696	1.319	0	0
12660	2807141	yfiO	0.749	1.667	0	0
4951	2807357	yfiO, yfiA	0.664	1.757	0	0
5869	2807599	yfiA	0.708	1.704	0	0
3617	2807740	yfiA, STM2666	0.556	1.796	0	1
17091	2808135	pheA	0.826	1.852	0	1
13226	2808214	pheA	0.964	1.516	0	0
8853	2808639	pheA	1.097	0.995	0	0
4682	2808751	pheA	0.944	0.870	0	0
22159	2808901	pheA	1.160	0.979	0	0
12785	2809108	pheA	1.282	1.018	0	0
1631	2809502	STM2668	0.975	0.824	0	0
10140	2809729	STM2668	1.106	0.905	0	0
12365	2809846	STM2668	0.999	0.789	0	0
19103	2810212	STM2668	0.617	0.577	0	0
16416	2810419	STM2668, tyrA	1.005	0.952	0	0

4375	2810491	STM2668, tyrA	1.231	1.126	0	0
19620	2810700	STM2668, tyrA	1.107	1.131	0	0
17019	2811070	tyrA	0.790	0.855	0	0
19192	2811172	tyrA	1.154	1.148	0	0
9187	2811506	tyrA, aroF	1.028	0.873	0	0
18096	2811633	tyrA, aroF	1.311	0.890	0	0
20037	2811713	tyrA, aroF	1.261	0.985	0	0
5495	2812135	aroF	1.425	0.703	0	0
21838	2812304	aroF	1.964	0.789	0	0
22035	2812548	aroF, yfiR	2.733	0.744	0	0
19555	2812922	aroF, yfiR	1.734	0.706	0	0
17634	2812996	yfiR, yfiN	3.063	0.777	1	0
10070	2813387	yfiR, yfiN	2.487	0.699	1	0
19704	2813479	yfiN	3.196	0.639	1	0
17492	2813555	yfiN	2.763	0.686	1	0
18266	2814051	yfiN	1.627	0.790	0	0
14716	2814134	yfiN	1.779	0.841	0	0
5250	2814475	yfiN	1.406	0.957	0	0
4664	2814550	yfiN	1.194	0.896	0	0
7649	2814886	rplS	0.837	1.079	0	0
20400	2815157	rplS	0.683	1.791	0	0
2457	2815325	rplS, trmD	0.483	1.121	0	0
20127	2815616	rplS, trmD	0.722	1.999	0	1
14865	2815895	trmD	0.751	2.134	0	1
7807	2816078	trmD, rimM	0.650	1.703	0	0
10047	2816149	trmD, rimM	0.641	1.685	0	0
11367	2816605	rimM	0.473	2.039	0	1
6771	2816838	rimM, rpsP	0.645	2.963	0	1
17129	2816957	rimM, rpsP	0.725	2.787	0	1
15228	2817267	rpsP, ffh	0.714	1.805	0	1
15111	2817521	ffh	0.727	1.299	0	0
6769	2817626	ffh	0.875	1.377	0	0
19771	2817903	ffh	0.920	1.516	0	0
15189	2818154	ffh	0.402	0.821	0	0
15635	2818358	ffh, corE	0.804	2.286	0	1
17061	2818673	ffh, corE	0.692	3.033	0	1

13627	2818751	ffh, corE	0.745	2.994	0	1
18244	2819026	corE	0.705	1.515	0	0
6884	2819286	corE, yfjD	0.733	1.144	0	0
6931	2819493	yfjD	0.988	1.436	0	0
20819	2819574	yfjD	0.701	1.037	0	0
2994	2819679	yfjD	0.819	1.259	0	0
6082	2820155	yfjD	0.991	1.297	0	0
22239	2820408	yfjD	1.077	1.265	0	0
10185	2820481	yfjD, STM2680	0.925	1.143	0	0
12570	2820842	STM2680	0.900	0.945	0	0
21421	2821031	STM2680	0.873	1.089	0	0
7370	2821293	STM2680	0.746	1.531	0	0
8531	2821428	STM2680	0.561	1.694	0	1
10554	2821500	STM2680	0.674	1.697	0	1
15600	2821895	grpE, yfjB	0.559	1.913	0	1
14306	2822239	grpE, yfjB	0.739	1.605	0	0
13135	2822399	grpE, yfjB	0.764	1.299	0	0
4809	2822630	yfjB, STM2682	0.774	0.910	0	0
16769	2822777	yfjB, STM2682	0.936	1.109	0	0
18073	2823086	yfjB, recN	0.788	0.804	0	0
14486	2823288	recN	0.988	1.082	0	0
17382	2823597	recN	1.129	1.196	0	0
15311	2823833	recN	1.309	1.074	0	0
2929	2824132	recN	1.049	0.829	0	0
13100	2824366	recN	0.690	0.748	0	0
14454	2824495	recN	0.952	1.053	0	0
10294	2824772	recN, smpA	0.849	1.464	0	0
14434	2824922	smpA	0.856	2.024	0	1
21514	2825135	smpA	0.615	1.923	0	1
21823	2825376	smpA	0.741	2.736	0	1
2478	2825671	yfjF	0.652	2.069	0	1
19409	2825904	yfjF, yfjG	0.799	1.212	0	0
619	2826041	yfjF, yfjG	0.564	0.881	0	0
20930	2826202	yfjF, yfjG	0.936	1.234	0	0
13713	2826450	yfjG, smpB	1.191	1.292	0	0
2486	2826781	smpB	1.701	0.846	0	0

16165	2826899	smpB	2.024	0.749	0	0
10630	2827275		2.664	0.483	1	0
4087	2827441		3.149	0.732	1	0
15897	2827532		3.786	0.651	1	0
12512	2827667		2.906	0.714	1	0
6701	2827830		1.952	0.572	0	0
21211	2828259		1.341	0.738	0	0
13126	2828523		1.524	0.748	0	0
20727	2828807		1.158	0.851	0	0
21851	2828922		1.674	0.827	0	0
7039	2829086		1.496	0.742	0	0
6903	2829386		0.693	0.343	0	0
14189	2829655		1.026	0.560	0	0
6071	2829736		2.378	0.862	0	0
7068	2830095		1.374	0.861	0	0
5310	2830348		1.329	0.900	0	0
20251	2830564		1.593	1.037	0	0
19768	2830788		1.424	0.968	0	0
2638	2830924		0.407	0.565	0	0
6485	2831003		1.789	0.802	0	0
9870	2831461		0.961	0.615	0	0
10727	2831721		1.828	0.899	0	0
4489	2831916		1.722	0.972	0	0
4352	2832030		0.860	0.663	0	0
11439	2832242		1.027	0.765	0	0
14019	2832423		1.563	0.829	0	0
10582	2832966		1.388	0.674	0	0
5902	2833218		2.045	0.744	0	0
16719	2833408		1.850	0.829	0	0
4642	2833495		1.683	0.679	0	0
19900	2833839		2.091	0.857	0	0
16043	2834116		1.038	0.771	0	0
16164	2834316		1.245	0.814	0	0
21009	2834455		1.715	0.900	0	0
11398	2834631		1.414	0.720	0	0
2399	2834779		1.013	0.728	0	0

6170	2835014		1.094	0.678	0	0
14042	2835295		1.725	0.825	0	0
22267	2835647		0.858	0.559	0	0
16785	2835851		1.100	0.492	0	0
8106	2835934		1.218	0.654	0	0
15735	2836285		0.884	0.510	0	0
21151	2836516		1.834	0.987	0	0
5544	2836783		1.205	0.656	0	0
19083	2837053		1.291	0.899	0	0
5225	2837299		1.834	0.762	0	0
858	2837413		1.557	0.825	0	0
11550	2837752		0.791	0.479	0	0
4532	2837828		1.873	0.710	0	0
8364	2838096		1.357	0.653	0	0
3518	2838339		1.720	0.710	0	0
8042	2838598		1.574	0.615	0	0
8751	2838669	STM2690	1.256	0.568	0	0
10041	2838963	STM2690	1.124	0.580	0	0
16475	2839352	STM2690	1.228	1.051	0	0
10092	2839466	STM2690	1.379	1.213	0	0
17494	2839818	STM2690	0.566	0.722	0	0
19887	2839889	STM2690	1.168	1.546	0	0
9723	2840007	STM2690	1.735	1.456	0	0
4437	2840266	STM2690, STM2691	1.220	0.797	0	0
8163	2840467	STM2690, STM2691	1.485	0.776	0	0
10303	2840801	STM2691	1.877	0.860	0	0
19051	2841077	STM2691	1.410	0.666	0	0
15555	2841374	STM2691	1.317	0.842	0	0
12259	2841582	STM2691	1.888	0.702	0	0
7103	2841683	STM2691	1.011	0.572	0	0
15790	2841966	STM2691	1.399	0.772	0	0
3043	2842125	STM2691	1.469	0.871	0	0
17130	2842485	STM2691, STM2692	1.337	0.773	0	0
9327	2842673	STM2691, STM2692	1.251	0.790	0	0
7691	2842757	STM2692	0.843	0.712	0	0
20250	2843050	STM2692	1.001	1.106	0	0

12936	2843261	STM2692	1.166	1.104	0	0
21243	2843665	STM2692	0.779	2.195	0	1
17651	2843906	10Sa	0.570	3.313	0	1
16318	2844020	10Sa	0.581	3.597	0	1
7917	2844217	10Sa	0.468	3.315	0	1
8169	2844548	STM2694	0.664	1.445	0	0
19600	2844624	STM2694	0.671	1.142	0	0
19733	2844721	STM2694, STM2695	0.740	0.926	0	0
7951	2845052	STM2694, STM2695	0.932	0.692	0	0
2935	2845268	STM2695	1.001	0.673	0	0
17584	2845530	STM2695	1.109	0.715	0	0
1130	2845780	STM2695	0.929	0.618	0	0
14954	2845979	STM2695, STM2696	1.506	0.633	0	0
1182	2846227	STM2695, STM2696	1.326	0.471	0	0
9779	2846421	STM2696, STM2697	1.833	0.721	0	0
8842	2846811	STM2697	0.500	0.482	0	0
18420	2846925	STM2697	1.831	0.664	0	0
6636	2847294	STM2697	1.594	0.758	0	0
16866	2847455	STM2697	1.028	0.679	0	0
18779	2847570	STM2697	0.839	0.508	0	0
22344	2847829	STM2697	1.662	0.674	0	0
7831	2848034	STM2697	1.665	0.661	0	0
15171	2848350	STM2697	1.163	0.512	0	0
1098	2848425	STM2697	1.341	0.649	0	0
10445	2848743	STM2697	1.356	0.622	0	0
2686	2849045	STM2697	1.547	0.650	0	0
7869	2849236	STM2697, STM2698	1.195	0.718	0	0
10440	2849485	STM2697, STM2698	1.615	0.632	0	0
5071	2849634	STM2698, STM2699	1.330	0.571	0	0
9094	2849900	STM2699, STM2700	1.082	0.640	0	0
7114	2850053	STM2700	1.587	0.659	0	0
18015	2850194	STM2700, STM2701	1.197	0.630	0	0
7644	2850689	STM2701	0.979	0.635	0	0
15772	2850762	STM2701	1.300	0.886	0	0
4337	2850928	STM2701	1.276	1.159	0	0
19751	2851379	STM2701, STM2702	1.425	1.204	0	0

3259	2851608	STM2701, STM2703	1.385	1.110	0	0
6229	2851752	STM2703, STM2702	1.383	0.837	0	0
18196	2851974	STM2703, STM2702	1.812	0.702	0	0
20560	2852180	STM2703, STM2702	3.268	0.733	1	0
13221	2852383	STM2703	3.336	0.663	1	0
14156	2852599	STM2703, STM2704	1.617	0.631	0	0
15112	2852896	STM2703, STM2704	3.062	0.598	1	0
12532	2853060	STM2704	2.630	0.589	1	0
1557	2853131	STM2704	2.647	0.573	1	0
839	2853618	STM2705	2.729	0.612	1	0
665	2853794	STM2705	2.646	0.623	1	0
16967	2853981	STM2705, STM2706	3.736	0.648	1	0
14880	2854188	STM2705, STM2706	2.880	0.613	1	0
13792	2854558	STM2706	2.031	0.613	0	0
10394	2854735	STM2706	2.558	0.884	0	0
19030	2854902	STM2706	1.638	0.786	0	0
16979	2855134	STM2706	1.124	0.663	0	0
14872	2855296	STM2706	1.014	0.702	0	0
2316	2855548	STM2706, STM2707	1.674	0.626	0	0
17143	2855626	STM2706, STM2707	1.836	0.723	0	0
2541	2855778	STM2706, STM2707	1.578	0.707	0	0
8764	2856370	STM2707, STM2708	1.415	0.684	0	0
21640	2856477	STM2707, STM2708	1.286	0.745	0	0
2983	2856767	STM2708	1.530	0.624	0	0
2928	2857063	STM2708, STM2709	1.778	0.558	0	0
16526	2857134	STM2708, STM2709	1.225	0.587	0	0
6575	2857441	STM2708, STM2709	1.407	0.583	0	0
7522	2857543	STM2709, STM2710	0.574	0.504	0	0
19417	2857965	STM2710	1.706	0.553	0	0
2768	2858149	STM2710, STM2711	1.526	0.582	0	0
17579	2858261	STM2710, STM2711	1.207	0.649	0	0
15672	2858615	STM2711, STM2712	1.959	0.641	0	0
5868	2858760	STM2711, STM2712	1.731	0.582	0	0
16150	2858910	STM2711, STM2712	1.262	0.562	0	0
9565	2859286	STM2712, STM2713	1.258	0.514	0	0
1708	2859465	STM2713, STM2714	0.640	0.560	0	0

8045	2859542	STM2713, STM2714	1.415	0.551	0	0
1351	2859799	STM2714	1.508	0.610	0	0
631	2860215	STM2715	1.562	0.707	0	0
831	2860376	STM2715, STM2716	1.026	0.703	0	0
389	2860496	STM2715, STM2716	1.889	0.768	0	0
10379	2860708	STM2715, STM2716	1.302	0.660	0	0
13034	2861107	STM2717, STM2718	1.196	0.525	0	0
13279	2861196	STM2718	1.128	0.619	0	0
16399	2861468	STM2718, STM2719	1.484	0.623	0	0
20113	2861659	STM2719	0.923	0.705	0	0
6434	2862047	STM2719, STM2720	1.403	0.603	0	0
3118	2862139	STM2719, STM2720	1.460	0.561	0	0
628	2862325	STM2719, STM2720	0.704	0.451	0	0
18605	2862703	STM2720	1.281	0.604	0	0
8345	2862928	STM2720	1.039	0.631	0	0
997	2863007	STM2720	1.702	0.787	0	0
19184	2863277	STM2720, STM2721	1.294	0.796	0	0
9477	2863391	STM2720, STM2721	1.504	0.773	0	0
5037	2863900	STM2721, STM2722	0.850	0.504	0	0
6302	2864133	STM2721, STM2722	1.379	0.640	0	0
18264	2864222	STM2721, STM2722	0.687	0.551	0	0
8393	2864394	STM2722	1.982	0.700	0	0
7261	2864632	STM2722	1.524	0.699	0	0
8009	2864757	STM2722	1.319	0.556	0	0
8526	2865123	STM2722	1.541	0.594	0	0
10793	2865401	STM2722, STM2723	0.958	0.444	0	0
6232	2865516	STM2722, STM2723	1.479	0.614	0	0
1997	2865769	STM2722, STM2723	1.413	0.522	0	0
12761	2866106	STM2723	1.547	0.696	0	0
1164	2866271	STM2723	1.407	0.564	0	0
18394	2866518	STM2723	2.005	0.495	0	0
14869	2866760	STM2723	3.025	0.492	0	0
1927	2866975	STM2724	2.414	0.469	0	0
21543	2867087	STM2724	3.046	0.415	0	0
17005	2867490	STM2724	1.867	0.560	0	0
8476	2867662	STM2724	1.718	0.422	0	0

1364	2867816	STM2724	2.053	0.552	0	0
13391	2867977	STM2724	2.339	0.496	0	0
7147	2868115	STM2724	2.140	0.537	0	0
222	2868586	STM2724	1.035	0.693	0	0
16346	2868846	STM2724	0.806	0.498	0	0
2367	2869122	STM2726	0.839	0.609	0	0
17628	2869361	STM2726	1.206	0.596	0	0
17674	2869509	STM2726	1.085	0.524	0	0
11884	2869692	STM2726, STM2727	1.014	0.656	0	0
20986	2869926	STM2726, STM2727	0.873	0.582	0	0
4335	2870081	STM2727, STM2728	0.881	0.692	0	0
10100	2870425	STM2727, STM2728	1.016	0.599	0	0
19495	2870600	STM2729	1.004	0.538	0	0
7253	2870738	STM2729	1.201	0.587	0	0
6068	2871123	STM2729	0.822	0.615	0	0
2605	2871302	STM2729	1.412	0.663	0	0
4959	2871407	STM2729	1.383	0.507	0	0
9697	2871478	STM2729	1.191	0.574	0	0
16592	2872025	STM2729	1.260	0.659	0	0
5447	2872260	STM2729	1.744	0.646	0	0
7567	2872408	STM2729	1.828	0.606	0	0
12861	2872710	STM2729, STM2730	0.819	0.596	0	0
20628	2872825	STM2729, STM2730	2.419	0.591	1	0
13571	2872936	STM2729, STM2730	2.942	0.552	1	0
2164	2873254	STM2730	2.589	0.478	0	0
15110	2873388	STM2730	2.320	0.589	0	0
9818	2873742	STM2730, STM2731	1.858	0.517	0	0
7948	2873965	STM2730, STM2731	3.058	0.601	0	0
1729	2874210	STM2732, STM2733	2.081	0.572	0	0
16582	2874396	STM2732, STM2733	2.256	0.726	0	0
3432	2874510	STM2733, STM2734	1.901	0.611	0	0
13415	2874664	STM2733, STM2734	1.966	0.468	0	0
8665	2875061	STM2735	0.861	0.502	0	0
14567	2875229	STM2735	1.341	0.730	0	0
18283	2875361	STM2735, STM2736	0.641	0.395	0	0
9326	2875779	STM2736, STM2738	0.802	0.608	0	0

15367	2876066	STM2736, STM2737	0.615	0.640	0	0
12755	2876321	STM2737, STM2738	0.817	0.640	0	0
3636	2876528	STM2738, STM2739	0.728	0.569	0	0
20854	2876798	STM2739	0.786	0.594	0	0
15344	2876919	STM2739	0.585	0.594	0	0
7569	2877204	STM2739	1.000	0.761	0	0
14794	2877469	STM2739	0.960	0.724	0	0
8883	2877739	STM2739, STM2740	0.952	0.788	0	0
16611	2877860	STM2740	0.855	0.786	0	0
11096	2878055	STM2740	0.398	0.406	0	0
6958	2878422	STM2740	0.868	0.567	0	0
16970	2878493	STM2740	0.839	0.585	0	0
912	2878705	STM2740	1.278	0.658	0	0
2761	2878950	STM2740	1.249	0.641	0	0
14982	2879220	STM2741	1.166	0.578	0	0
9584	2879390	STM2741	1.037	0.504	0	0
11281	2879693	STM2741	1.591	0.587	0	0
830	2879929	STM2741	2.060	0.753	0	0
10400	2880000	STM2741, STM2742	1.502	0.649	0	0
22315	2880337	STM2742	2.521	0.643	1	0
7782	2880464	STM2742	2.754	0.597	1	0
10452	2880806	STM2742	2.517	0.556	0	0
22180	2880918	STM2742	2.703	0.566	1	0
9961	2881365	STM2742	2.353	0.639	1	0
18085	2881436	STM2742	2.052	0.570	0	0
18839	2881767		1.110	0.481	0	0
9853	2882034	STM2743	1.954	0.420	0	0
15961	2882203	STM2743	2.437	0.511	0	0
8530	2882319	STM2743	2.126	0.320	0	0
18741	2882615	STM2743	2.649	0.585	1	0
4744	2882924	STM2743, STM2744	2.776	0.456	1	0
9024	2883020	STM2743, STM2744	2.939	0.680	1	0
12906	2883222	STM2743, STM2744	1.466	0.514	0	0
2785	2883316	STM2744, STM2745	1.871	0.557	0	0
8329	2883449	STM2745	1.479	0.564	0	0
9079	2883647	STM2745	1.184	0.634	0	0

1483	2883725	STM2745	1.540	0.552	0	0
3486	2884229	STM2745	0.804	0.585	0	0
11266	2884471	STM2745	1.702	0.690	0	0
14038	2884809	STM2745	1.972	0.862	0	0
236	2885014	STM2745	0.964	0.658	0	0
3832	2885341	STM2745	0.703	0.912	0	0
11669	2885562		1.032	0.604	0	0
8180	2885771	STM2746	2.606	0.634	1	0
13488	2885892	STM2746	3.051	0.558	1	0
7188	2886054	STM2746	2.830	0.435	1	0
1366	2886594	STM2746	1.945	0.485	0	0
8447	2886679	STM2746	1.889	0.453	0	0
18668	2886842	STM2746	1.725	0.446	0	0
16938	2887166	STM2746, STM2747	1.662	0.408	0	0
12864	2887320	STM2746, STM2747	1.819	0.498	0	0
15665	2887519	STM2747	1.874	0.482	0	0
11492	2887923	STM2747	1.747	0.511	0	0
13741	2888036	STM2747	2.218	0.558	0	0
10392	2888425	STM2748, STM2749	3.172	0.742	1	0
17480	2888504	STM2748, STM2749	3.240	0.919	1	0
8479	2888609	STM2748, STM2749	2.117	0.741	0	0
21777	2888695	STM2748, STM2749	1.191	0.686	0	0
17546	2888797	STM2748, STM2749	1.639	0.717	0	0
3099	2889130	STM2748, STM2750	0.861	0.584	0	0
14797	2889792	STM2751, STM2752	0.943	0.572	0	0
2043	2889928	STM2751, STM2752	1.275	0.493	0	0
20193	2890068	STM2751, STM2752	0.814	0.499	0	0
8484	2890254	STM2752	1.246	0.530	0	0
19610	2890410	STM2752	1.762	0.688	0	0
5346	2890645	STM2752, STM2753	1.472	0.744	0	0
4027	2891052	STM2752, STM2753	1.216	0.563	0	0
5624	2891250	STM2753	0.599	0.627	0	0
12758	2891458	STM2753	1.427	0.590	0	0
20112	2891832	STM2753	1.595	0.864	0	0
14917	2891966	STM2753, STM2754	2.066	0.893	0	0
19845	2892239	STM2754	3.237	0.623	1	0

1095	2892488	STM2754	2.928	0.662	1	0
16407	2892626	STM2754	3.577	0.586	1	0
11169	2892855	STM2754	3.290	0.515	1	0
711	2893163	STM2754	3.169	0.578	1	0
4739	2893401	STM2754	3.281	0.561	1	0
14028	2893532	STM2754	2.822	0.628	1	0
12536	2893604		2.916	0.626	1	0
19412	2893678	STM2755	2.351	0.661	1	0
2900	2894238	STM2755	1.690	0.592	0	0
21107	2894311	STM2755	1.338	0.548	0	0
9499	2894565	STM2755, STM2756	0.596	0.455	0	0
3203	2894929	STM2756, STM2757	1.341	0.522	0	0
7002	2895176	STM2756, STM2757	1.598	0.595	0	0
8023	2895437	STM2757	1.542	0.693	0	0
17646	2895545	STM2757	1.699	0.723	0	0
4628	2895860	STM2757, STM2758	1.534	0.616	0	0
20616	2896068	STM2757, STM2758	1.310	0.596	0	0
9716	2896268	STM2758	1.780	0.705	0	0
6284	2896467	STM2758	1.445	0.691	0	0
17690	2896681	STM2758	1.301	0.692	0	0
3109	2896916	STM2758	1.658	0.788	0	0
15770	2897270	STM2758, STM2759	1.398	0.656	0	0
1752	2897474	STM2758, STM2759	1.245	0.662	0	0
17714	2897559	STM2758, STM2759	1.493	0.655	0	0
10702	2897732	STM2758, STM2759	1.594	0.691	0	0
13728	2898141	STM2759	1.195	0.612	0	0
8799	2898292	STM2759	1.299	0.755	0	0
22052	2898519	STM2759	1.296	0.593	0	0
20767	2898684	STM2759	1.683	0.745	0	0
5394	2899003	STM2759	1.110	0.552	0	0
3393	2899204	STM2759	1.625	0.692	0	0
15583	2899492		1.413	0.748	0	0
1882	2899713		1.335	0.664	0	0
15664	2899803		1.331	0.705	0	0
6639	2900156	STM2760	1.223	0.912	0	0
7921	2900365	STM2760	0.863	0.723	0	0

2671	2900631	STM2760	1.601	0.733	0	0
8832	2900885	STM2760	1.445	0.464	0	0
8059	2901060	STM2760, STM2761	2.794	0.571	1	0
15029	2901259	STM2760, STM2761	3.013	0.554	1	0
20964	2901431	STM2761	3.021	0.408	1	0
13162	2901672	STM2761	2.295	0.440	0	0
16786	2901817	STM2761	2.555	0.445	0	0
15100	2902265	STM2761	2.330	0.470	0	0
1808	2902408	STM2761	2.078	0.537	0	0
17158	2902642	STM2761	2.239	0.484	0	0
11006	2902989	STM2761	2.321	0.417	0	0
1572	2903096	STM2761	1.861	0.422	0	0
5350	2903300	STM2761	1.541	0.303	0	0
385	2903634	STM2762	1.747	0.437	0	0
9118	2903893	STM2762	1.722	0.427	0	0
2337	2903996	STM2762	1.491	0.411	0	0
4578	2904350	STM2762	2.151	0.483	0	0
12357	2904461	STM2762	2.056	0.476	0	0
17254	2904728	STM2762	2.029	0.436	0	0
19371	2904928	STM2762	1.826	0.276	0	0
16800	2905085	STM2762, STM2763	1.701	0.433	0	0
22241	2905455	STM2763	1.267	0.643	0	0
9112	2905613	STM2763	1.224	0.605	0	0
13956	2905715	STM2763	1.253	0.632	0	0
14056	2905946	STM2763	1.400	0.767	0	0
7473	2906136		1.726	0.747	0	0
14877	2906278		1.283	0.658	0	0
18660	2906420		1.674	0.646	0	0
18023	2907051	STM2764, STM2765	1.852	0.615	0	0
12881	2907137	STM2764, STM2765	1.799	0.583	0	0
8817	2907405	STM2765	2.648	0.547	0	0
4303	2907559	STM2765	2.177	0.569	0	0
12459	2907907	STM2766	2.302	0.488	0	0
21542	2908046	STM2766	2.017	0.366	0	0
7981	2908321	STM2766	2.401	0.495	0	0
7888	2908454	STM2766, STM2767	2.240	0.562	1	0

10472	2908763	STM2766, STM2767	2.304	0.508	1	0
9911	2908884	STM2767	2.218	0.566	0	0
11467	2909234	STM2767	2.020	0.516	0	0
16885	2909328	STM2767	1.926	0.446	0	0
15488	2909627	STM2767	2.260	0.496	0	0
14704	2909811	STM2767	1.891	0.562	0	0
6315	2910137	STM2767	2.229	0.654	0	0
4953	2910346	STM2767, STM2768	1.727	0.650	0	0
21240	2910559	STM2767, STM2768	1.364	0.680	0	0
10885	2910803	STM2767, STM2768	0.963	0.572	0	0
9794	2910960	STM2769	1.174	0.634	0	0
20596	2911035	STM2769	1.114	0.710	0	0
7662	2911523	STM2769	1.202	1.015	0	0
15792	2911839		1.721	1.030	0	0
324	2912001		1.402	0.827	0	0
19411	2912096		1.649	0.807	0	0
17068	2912381		1.456	0.969	0	0
19116	2912710	fljA	2.049	0.778	0	0
5525	2912966	fljA	3.030	0.776	0	0
4624	2913104	fljA	3.233	0.813	0	0
12103	2913199	fljA	2.428	0.833	0	0
15732	2913647	fljB	0.799	0.838	0	0
10493	2913861	fljB	0.834	1.055	0	0
19988	2914130	fljB	0.712	1.028	0	0
21270	2914201	fljB	0.778	1.104	0	0
9763	2914401	fljB, hin	0.724	1.629	0	0
4933	2914744	fljB, hin	1.587	1.021	0	0
17013	2914907	fljB, hin	1.848	0.607	0	0
17503	2915058	fljB, hin	1.975	0.742	0	0
15869	2915425		2.536	0.672	0	0
14627	2915582		2.176	0.803	0	0
10518	2915875		1.326	0.854	0	0
20943	2916071	iroB	0.987	0.780	0	0
14152	2916218	iroB	1.009	0.696	0	0
16261	2916337	iroB	0.793	0.660	0	0
8948	2916574	iroB	1.001	0.683	0	0

1387	2916888	iroB	1.196	0.707	0	0
12421	2917011	iroB	0.601	0.396	0	0
10574	2917329	iroB, iroC	1.325	0.626	0	0
7388	2917637	iroC	0.692	0.519	0	0
8079	2917842	iroC	1.826	0.689	0	0
11951	2918172	iroC	1.247	0.637	0	0
18333	2918431	iroC	0.964	0.602	0	0
6568	2918608	iroC	1.493	0.625	0	0
5763	2918838	iroC	1.816	0.971	0	0
22150	2919114	iroC	2.027	0.856	0	0
2995	2919194	iroC	1.673	0.668	0	0
7277	2919586	iroC	1.528	0.788	0	0
21699	2919813	iroC	1.668	0.970	0	0
2029	2919920	iroC	0.879	0.592	0	0
351	2920038	iroC	2.005	0.989	0	0
2849	2920389	iroC	2.301	0.688	0	0
20457	2920485	iroC	1.719	0.871	0	0
8772	2920702	iroC	0.893	0.500	0	0
11441	2921081	iroC, iroD	1.716	0.762	0	0
19563	2921200	iroD	1.620	0.721	0	0
12293	2921283	iroD	2.428	0.810	0	0
7451	2921564	iroD	1.652	0.764	0	0
11204	2921919	iroD	1.348	0.784	0	0
2652	2922054	iroD	1.840	0.692	0	0
9130	2922415	iroD, iroE	1.817	0.890	0	0
14724	2922722	iroE	2.107	0.747	0	0
9520	2922941	iroE	1.639	0.841	0	0
16792	2923169	iroE	1.081	0.909	0	0
13498	2923294	iroE	1.523	1.048	0	0
18240	2923657	iroN	2.167	0.864	0	0
4182	2923874	iroN	2.070	0.577	0	0
19491	2924087	iroN	2.748	0.585	1	0
15972	2924271	iroN	3.379	0.732	1	0
8959	2924347	iroN	3.056	0.624	1	0
5987	2924461	iroN	2.704	0.659	1	0
19400	2924889	iroN	1.872	0.719	0	0

11221	2925056	iroN	1.690	0.768	0	0
8624	2925212	iroN	0.870	0.464	0	0
15225	2925673	iroN	1.100	0.513	0	0
10936	2925909	iroN	0.981	0.568	0	0
16400	2926056	iroN	0.958	0.536	0	0
12458	2926289	STM2779	1.309	0.529	0	0
17632	2926447	STM2779	1.970	0.548	0	0
10926	2926598	STM2779	2.201	0.489	0	0
11990	2926905	STM2779, STM2780	2.304	0.547	0	0
6567	2927100	STM2780	2.827	0.503	1	0
13837	2927517	STM2780	2.548	0.594	1	0
9510	2927627	STM2780	2.152	0.538	0	0
21489	2927807	STM2780	1.780	0.551	0	0
6842	2928185	STM2780, virK	2.737	0.572	1	0
6180	2928332	virK	2.814	0.551	1	0
10180	2928670	virK	2.215	0.615	1	0
15764	2928767	virK	1.663	0.552	0	0
9901	2929023	virK	1.573	0.519	0	0
13524	2929232	virK, mig-14	1.743	0.641	0	0
7739	2929343	mig-14	1.773	0.638	0	0
3597	2929421	mig-14	1.682	0.588	0	0
5034	2929924	mig-14	2.138	0.572	0	0
10189	2930182	mig-14	2.358	0.539	0	0
11994	2930398	mig-14	2.276	0.588	0	0
7028	2930632		2.317	0.560	0	0
11540	2930794		2.234	0.619	0	0
7452	2930964		2.149	0.522	0	0
11992	2931123		2.558	0.655	0	0
21488	2931272	nxiA	2.028	0.871	0	0
9233	2931655	nxiA	1.236	0.807	0	0
3695	2931866	nxiA	1.008	0.933	0	0
14015	2932259	nxiA	0.853	0.819	0	0
5243	2932340	nxiA, tctE	1.169	1.163	0	0
15757	2932655	nxiA, tctE	0.943	1.430	0	0
2311	2932838	tctE	1.077	0.956	0	0
10372	2933060	tctE	1.071	1.228	0	0

7962	2933152	tctE	1.385	1.545	0	0
19295	2933505	tctE	0.865	1.294	0	0
4858	2933848	tctE, tctD	1.081	1.402	0	0
4827	2934002	tctE, tctD	1.142	1.343	0	0
11404	2934367	tctD, STM2786	0.868	1.073	0	0
17030	2934456	tctD, STM2786	1.268	1.623	0	0
11739	2934592	tctD, STM2786	1.050	1.260	0	0
17965	2934729	tctD, STM2786	0.939	0.898	0	0
21848	2935176	STM2786, STM2787	1.368	1.537	0	0
15812	2935387	STM2786, STM2787	1.354	1.593	0	0
12612	2935488	STM2786, STM2787	1.376	1.368	0	0
12793	2935785	STM2787, STM2788	1.177	1.140	0	0
10584	2935999	STM2788	1.427	1.452	0	0
8343	2936361	STM2788	1.193	1.240	0	0
12657	2936590	STM2788	1.263	1.324	0	0
22341	2936665	STM2788	1.277	1.252	0	0
17853	2936859	STM2788	1.085	0.947	0	0
19960	2937134	STM2788	0.964	1.306	0	0
12693	2937459	STM2788, STM2789	1.025	0.932	0	0
3946	2937741	STM2789	1.413	0.848	0	0
12318	2937822	STM2789	1.374	0.844	0	0
12569	2938007	STM2789	1.315	0.827	0	0
21552	2938331	STM2789	1.264	0.613	0	0
11080	2938673	STM2789, ygaF	1.099	0.616	0	0
545	2938804	ygaF	1.354	0.681	0	0
13602	2939043	ygaF	0.753	0.571	0	0
13765	2939224	ygaF	1.114	0.605	0	0
13334	2939412	ygaF	1.421	0.731	0	0
15801	2939805	ygaF, gabD	0.632	0.630	0	0
5610	2939903	ygaF, gabD	1.383	0.747	0	0
13078	2940098	gabD	1.472	0.750	0	0
21168	2940454	gabD	1.630	0.745	0	0
21059	2940525	gabD	0.695	0.444	0	0
3786	2940840	gabD	2.322	0.791	0	0
12079	2941007	gabD	2.007	0.718	0	0
5684	2941105	gabD, gabT	1.929	0.616	0	0

16698	2941556	gabT	2.182	0.686	0	0
21238	2941762	gabT	1.919	1.020	0	0
6621	2942022	gabT	0.899	0.697	0	0
4477	2942154	gabT	1.510	1.110	0	0
6465	2942525	gabT, gabP	2.081	1.387	0	0
3490	2942749	gabT, gabP	0.857	0.782	0	0
15159	2942971	gabP	1.642	0.918	0	0
3006	2943153	gabP	2.078	0.850	0	0
13429	2943399	gabP	2.582	0.742	1	0
21490	2943526	gabP	2.268	0.856	1	0
17717	2943863	gabP	1.664	0.765	0	0
5834	2944051	gabP, ygaE	2.037	0.705	0	0
2134	2944326	gabP, ygaE	1.987	0.705	0	0
8281	2944502	ygaE	2.027	0.671	0	0
6646	2944639	ygaE	1.674	0.696	0	0
17703	2944940	ygaE	2.532	0.694	1	0
13441	2945136	ygaU	3.702	0.639	1	0
19735	2945228	ygaU	4.337	0.658	1	0
15295	2945550	ygaU, STM2797	3.851	0.683	1	0
12246	2945736	ygaU, yqaE	3.394	0.650	1	0
14613	2945818	ygaU, yqaE	3.491	0.675	1	0
13562	2946289	yqaE, STM2797	1.978	0.701	0	0
12860	2946519	ygaP	0.925	0.599	0	0
4582	2946834	ygaP	1.209	0.993	0	0
21950	2947001		1.430	1.257	0	0
8897	2947133		1.528	1.120	0	0
9602	2947414	stpA	2.085	0.830	0	0
16083	2947758	stpA	1.744	0.693	0	0
7033	2947929	stpA, STM2800	1.248	0.473	0	0
17443	2948027	stpA, STM2800	1.291	0.705	0	0
3242	2948386	STM2800	0.825	0.992	0	0
20154	2948574	STM2800	0.927	1.156	0	0
10963	2948838	ygaC, ygaM	1.036	1.110	0	0
22254	2948911	ygaC, ygaM	1.081	1.183	0	0
17957	2949228	ygaC, ygaM	1.060	0.947	0	0
9919	2949302	ygaC, ygaM	1.086	0.909	0	0

17840	2949374	ygaC, ygaM	1.184	1.041	0	0
14691	2949913	STM2803	1.268	1.183	0	0
15818	2950002	STM2803	1.310	1.011	0	0
8759	2950244	STM2803	1.386	0.937	0	0
12590	2950543	STM2803	1.614	0.991	0	0
937	2950775	STM2803, STM2804	4.784	0.894	0	0
1184	2951127	STM2803, STM2804	1.161	0.652	0	0
4206	2951255	STM2803, STM2804	0.949	0.607	0	0
10812	2951534	STM2804, nrdH	1.096	0.900	0	0
12653	2951839	nrdH, nrdI	1.050	1.011	0	0
8369	2951914	nrdH, nrdI	1.012	0.973	0	0
17652	2952185	nrdI, nrdE	1.112	0.860	0	0
9882	2952437	nrdI, nrdE	1.255	0.690	0	0
13519	2952535	nrdE	1.321	0.650	0	0
10964	2952803	nrdE	1.353	0.827	0	0
12628	2952920	nrdE	0.949	0.657	0	0
17324	2953284	nrdE	1.525	0.779	0	0
14252	2953643	nrdE	1.468	0.855	0	0
15460	2953739	nrdE	1.509	0.850	0	0
14781	2953949	nrdE	1.249	0.764	0	0
18842	2954248	nrdE, nrdF	1.484	0.774	0	0
13748	2954354	nrdE, nrdF	0.711	0.572	0	0
20447	2954502	nrdE, nrdF	1.443	0.878	0	0
5795	2954597	nrdF	1.325	0.831	0	0
302	2955173	nrdF	1.433	0.724	0	0
21020	2955302	nrdF	2.148	0.873	0	0
5354	2955545	nrdF, proV	2.577	0.819	1	0
13586	2955822	proV	3.562	0.893	1	0
21996	2956019	proV	2.651	0.842	1	0
2672	2956216	proV	2.815	1.040	1	0
13309	2956535	proV	2.559	0.893	1	0
6062	2956607	proV	2.702	0.866	1	0
21829	2957063	proV, proW	2.077	1.154	0	0
15569	2957138	proW	1.746	1.099	0	0
22174	2957281	proW	1.370	1.066	0	0
6953	2957595	proW	0.791	0.788	0	0

13383	2957780	proW, proX	1.265	1.253	0	0
6248	2958160	proW, proX	1.316	1.038	0	0
4341	2958248	proX	0.980	0.880	0	0
2724	2958363	proX	1.307	0.851	0	0
7862	2958768	proX	1.741	1.058	0	0
12406	2958845	proX	1.459	1.040	0	0
11327	2959356	STM2812	1.389	0.855	0	0
5374	2959429	STM2812	1.557	1.069	0	0
14743	2959723	STM2812	0.707	0.459	0	0
5559	2959901	STM2812	1.122	0.783	0	0
3337	2960016	STM2812	1.399	0.878	0	0
12920	2960360	STM2812	0.451	0.547	0	0
19215	2960608		1.399	1.342	0	0
5179	2960721	emrR	1.153	1.270	0	0
4864	2961073	emrR	1.189	1.060	0	0
19496	2961181	emrR	1.366	1.173	0	0
12104	2961516	emrR, emrA	1.168	1.296	0	0
1472	2961742	emrA	1.452	1.164	0	0
11889	2961898	emrA	1.289	1.183	0	0
6144	2962299	emrA	1.203	1.096	0	0
477	2962522	emrA, emrB	1.865	1.115	0	0
17815	2962593	emrA, emrB	1.542	1.149	0	0
2078	2962908	emrA, emrB	0.846	0.708	0	0
3132	2963112	emrB	0.774	0.717	0	0
18350	2963441	emrB	1.389	0.875	0	0
8316	2963614	emrB	1.055	0.779	0	0
367	2963730	emrB	1.432	0.988	0	0
17660	2963952	emrB	0.855	0.654	0	0
6950	2964279	emrB	1.560	0.829	0	0
15891	2964520		1.541	0.772	0	0
12159	2964634	STM2816	1.630	0.637	0	0
11351	2964750	STM2816	1.581	0.620	0	0
16140	2965131	STM2816	2.944	0.429	1	0
3970	2965360	STM2816	3.423	0.558	1	0
6376	2965599	STM2816	3.201	0.551	1	0
1488	2965865	STM2816	3.827	0.556	1	0

20160	2965962	STM2816	5.090	0.543	1	0
10261	2966246	STM2816, luxS	3.772	0.601	1	0
9626	2966609	luxS	2.196	0.818	1	0
16558	2966743	luxS	1.927	0.947	0	0
9493	2966994	luxS, gshA	1.441	0.974	0	0
21319	2967148	luxS, gshA	1.069	1.011	0	0
1567	2967455	gshA	1.323	1.016	0	0
4811	2967699	gshA	1.204	0.975	0	0
5594	2967895	gshA	1.186	1.033	0	0
18274	2968030	gshA	1.119	1.097	0	0
13010	2968277	gshA	0.944	1.140	0	0
13390	2968502	gshA	0.947	1.769	0	1
5897	2968815	gshA, yqaA	0.934	2.409	0	1
8349	2968943	yqaA	0.687	1.512	0	0
16221	2969344	yqaA, yqaB	0.701	1.838	0	1
7082	2969500	yqaB	0.636	2.111	0	1
2825	2969620	yqaB	0.665	2.000	0	1
16008	2969867	yqaB	0.448	3.217	0	1
11045	2970124	STM2821	0.406	3.099	0	1
494	2970276	STM2821, STM2822	0.382	4.986	0	1
7380	2970686	STM2822, STM2823	0.377	4.497	0	1
8723	2970757	STM2823, STM2824	0.334	2.221	0	0
18187	2971098	csrA	0.495	2.174	0	1
21385	2971199	csrA	0.600	2.052	0	1
15022	2971275	csrA	0.520	1.549	0	0
21970	2971573	csrA, alaS	0.774	1.764	0	1
19311	2972004	alaS	0.868	1.694	0	1
5003	2972141	alaS	0.819	1.695	0	1
20273	2972398	alaS	0.738	1.201	0	0
3572	2972589	alaS	0.915	1.520	0	0
19776	2972775	alaS	1.015	1.721	0	0
15975	2973205	alaS	0.873	1.704	0	0
3591	2973338	alaS	0.828	1.533	0	0
4478	2973561	alaS	0.828	1.585	0	0
10566	2973724	alaS	0.680	1.546	0	0
8563	2973971	alaS	0.810	1.970	0	0

4970	2974234	alaS, oraA	0.973	1.482	0	0
17623	2974390	alaS, oraA	0.959	1.412	0	0
12877	2974720	oraA	0.931	1.569	0	0
15328	2974910	oraA, recA	0.722	1.632	0	0
11958	2975066	oraA, recA	0.764	1.529	0	0
904	2975313	recA	1.030	1.706	0	1
19648	2975603	recA	0.882	1.789	0	1
3156	2975910	recA	0.815	1.368	0	0
17770	2975992	recA, ygaD	0.648	1.136	0	0
20670	2976323	recA, ygaD	0.991	1.356	0	0
21539	2976601	ygaD	1.305	1.045	0	0
4026	2976679	ygaD	1.116	1.006	0	0
11356	2976752	ygaD	0.957	0.840	0	0
22266	2976888	ygaD	1.237	1.135	0	0
3743	2977314	mltB	1.097	1.197	0	0
11363	2977759	mltB	1.104	1.024	0	0
17905	2977980	mltB, srlA	1.471	1.024	0	0
19941	2978122	mltB, srlA	1.347	0.810	0	0
10652	2978266	mltB, srlA	1.523	0.683	0	0
4554	2978340	mltB, srlA	1.594	0.741	0	0
8387	2978815	srlA, srlE	1.454	0.657	0	0
1864	2978911	srlE	1.508	0.729	0	0
12874	2979255	srlE	1.177	0.813	0	0
3683	2979360	srlE, slrB	1.467	0.788	0	0
11921	2979829	slrB, srlD	1.041	0.646	0	0
12000	2979925	slrB, srlD	1.440	0.699	0	0
18438	2980183	slrB, srlD	1.644	0.691	0	0
3851	2980315	srlD	1.540	0.802	0	0
20878	2980629	srlD, gutM	1.314	1.022	0	0
12677	2980850	srlD, gutM	1.144	0.780	0	0
4548	2981052	gutM	0.774	0.637	0	0
11470	2981330	gutM, srlR	1.172	0.878	0	0
19623	2981409	gutM, srlR	1.244	0.806	0	0
11614	2981712	srlR	0.631	0.693	0	0
19019	2981807	srlR	1.201	1.356	0	0
5827	2982120	srlR, gutQ	1.073	0.823	0	0

18374	2982524	gutQ	1.381	1.037	0	0
9981	2982638	gutQ	1.275	1.058	0	0
15902	2982733	gutQ	1.447	1.195	0	0
6426	2982811	gutQ	1.219	1.152	0	0
12477	2983329	gutQ	1.384	1.538	0	0
994	2983560	ygaA	1.070	1.155	0	0
6591	2983716	ygaA	0.709	0.773	0	0
3527	2984038	ygaA	1.419	0.830	0	0
13494	2984358	ygaA	1.373	0.706	0	0
7411	2984555	ygaA	1.648	0.752	0	0
11959	2984794	ygaA, STM2840	1.328	0.661	0	0
1335	2984866	ygaA, STM2840	1.692	0.765	0	0
20968	2985009	ygaA, STM2840	1.550	0.591	0	0
17226	2985166	ygaA, STM2840	1.421	0.841	0	0
18564	2985262	ygaA, STM2840	1.401	0.753	0	0
17320	2985815	STM2840	1.150	0.717	0	0
13351	2986002	STM2840	0.915	0.567	0	0
12928	2986146	STM2840, ygbD	1.222	0.794	0	0
8976	2986546	ygbD	1.297	0.728	0	0
7994	2986701	ygbD	1.375	0.740	0	0
12334	2986858	ygbD	1.396	0.759	0	0
16508	2987267	ygbD	0.968	0.873	0	0
12337	2987511	ygbD	1.835	1.305	0	0
9840	2987589	ygbD	1.689	1.205	0	0
1532	2987799	hypF	2.197	0.977	0	0
449	2988040	hypF	2.124	1.371	0	0
16085	2988384	hypF	1.373	1.193	0	0
4696	2988621	hypF	1.596	1.537	0	0
11126	2988906	hypF	1.487	1.439	0	0
10860	2989094	hypF	1.294	1.198	0	0
1433	2989294	hypF	0.641	0.707	0	0
2436	2989545	hypF	1.318	0.855	0	0
14959	2989662	hypF	1.296	2.203	0	0
4963	2989926	hypF	1.106	0.979	0	0
19594	2990097	hypF, hydN	1.309	0.936	0	0
21717	2990379	hypF, hydN	1.331	0.824	0	0

2413	2990661	hydN	1.527	0.751	0	0
16108	2990780	hydN	1.344	0.711	0	0
16628	2991066	hydN, STM2844	1.545	0.704	0	0
15893	2991273	STM2844	1.285	0.618	0	0
16264	2991606	STM2844	0.805	0.475	0	0
1036	2991835	STM2844, hycI	1.044	0.623	0	0
13988	2991913	STM2844, hycI	1.660	0.825	0	0
18780	2992140	hycI, hycH	0.975	0.650	0	0
19625	2992524	hycI, hycH	1.184	0.742	0	0
21067	2992595	hycI, hycH	1.415	0.800	0	0
17613	2992679	hycH, hycG	1.485	0.974	0	0
6542	2993208	hycG	1.784	0.685	0	0
14902	2993324	hycG, hycF	1.715	0.706	0	0
6592	2993558	hycG, hycF	1.269	0.668	0	0
12170	2993867	hycF, hycE	1.970	0.722	0	0
16599	2993938	hycF, hycE	1.364	0.706	0	0
4065	2994256	hycF, hycE	1.812	0.668	0	0
9543	2994475	hycE	1.419	0.700	0	0
11578	2994646	hycE	1.098	0.617	0	0
1612	2994934	hycE	1.327	0.529	0	0
3926	2995049	hycE	0.581	0.533	0	0
11565	2995458	hycE	1.368	0.634	0	0
20937	2995566	hycE	1.908	0.750	0	0
4333	2995836	hycE, hycD	0.676	0.620	0	0
15850	2996024	hycE, hycD	1.979	0.725	0	0
19812	2996226	hycD	1.639	0.628	0	0
16394	2996517	hycD, hycC	1.541	0.738	0	0
6832	2996645	hycD, hycC	3.756	0.729	0	0
13250	2996789	hycD, hycC	1.652	0.693	0	0
6607	2997263	hycC	1.646	0.721	0	0
17657	2997470	hycC	1.914	0.782	0	0
7678	2997620	hycC	1.061	0.637	0	0
6702	2997873	hycC	0.584	0.389	0	0
4357	2998034	hycC	1.251	0.621	0	0
7040	2998347	hycC, hycB	1.366	0.685	0	0
18361	2998679	hycC, hycB	1.092	0.644	0	0

19925	2998813	hycC, hycB	1.170	0.673	0	0
17246	2998987	hycB	1.089	0.745	0	0
10913	2999303	hycB, hycA	0.851	1.171	0	0
3263	2999555	hycA, hypA	0.661	2.442	0	1
14823	2999742	hycA, hypA	0.818	2.576	0	1
15059	2999836	hycA, hypA	0.816	2.407	0	1
3929	3000182	hypB	0.967	1.016	0	0
6914	3000448	hypB	1.086	0.779	0	0
18437	3000567	hypB	1.017	0.794	0	0
16380	3000837	hypB, hypC	1.510	0.881	0	0
970	3000924	hypB, hypC	1.059	0.745	0	0
12295	3001214	hypC, hypD	0.423	0.457	0	0
7830	3001493	hypD	1.554	0.800	0	0
17003	3001831	hypD	1.589	0.982	0	0
6552	3002027	hypD, hypE	1.831	0.768	0	0
12303	3002167	hypD, hypE	0.632	0.592	0	0
14683	3002436	hypD, hypE	1.293	0.841	0	0
11913	3002747	hypE	1.038	0.746	0	0
6776	3002891	hypE	1.448	1.074	0	0
8973	3003074	hypE	1.470	1.063	0	0
6975	3003441	hypE, fhlA	0.815	0.954	0	0
18224	3003673	fhlA	1.334	0.977	0	0
10675	3003876	fhlA	1.617	0.875	0	0
1200	3004121	fhlA	1.460	0.728	0	0
13073	3004212	fhlA	1.392	0.717	0	0
11368	3004387	fhlA	0.844	0.718	0	0
12117	3004492	fhlA	0.985	0.551	0	0
16560	3004623	fhlA	0.861	0.495	0	0
14094	3005186	fhlA	1.444	0.740	0	0
17298	3005297	fhlA	1.142	0.699	0	0
13714	3005597	fhlA	1.613	0.795	0	0
18936	3005739	fhlA	1.381	0.806	0	0
14895	3006117	ygbA, sitA	1.273	0.746	0	0
6241	3006345	ygbA, sitA	1.208	0.604	0	0
14585	3006431	ygbA, sitA	1.170	0.637	0	0
13884	3006808	sitA, sitB	1.469	0.731	0	0

14201	3007052	sitA, sitB	1.042	0.595	0	0
20109	3007254	sitA, sitB	1.292	0.685	0	0
13863	3007462	sitB	1.046	0.625	0	0
1712	3007536	sitB	0.778	0.514	0	0
19407	3007867	sitB, sitC	1.408	0.704	0	0
6829	3008081	sitC	1.380	0.690	0	0
15459	3008414	sitC	1.782	0.798	0	0
2997	3008534	sitC, sitD	1.469	0.645	0	0
16489	3008832	sitC, sitD	1.411	0.786	0	0
19395	3008932	sitD	1.547	0.808	0	0
797	3009366	sitD	2.307	0.871	0	0
3436	3009461	sitD	2.025	0.704	0	0
10216	3009697	sitD	3.843	0.788	1	0
19605	3009856		3.689	0.632	1	0
11645	3010120	avrA	2.837	0.617	1	0
16675	3010375	avrA	2.932	0.600	1	0
10137	3010574	avrA	2.898	0.560	1	0
10039	3010745	avrA	3.407	0.607	1	0
2048	3011080	avrA, sprB	3.108	0.612	1	0
22230	3011377	sprB	2.579	0.600	1	0
9138	3011588	sprB	2.075	0.529	0	0
19434	3011719	sprB	1.966	0.501	0	0
10436	3011965	sprB	1.811	0.404	0	0
6136	3012204	hilC	2.299	0.558	1	0
15776	3012400	hilC	2.283	0.644	1	0
9419	3012571	hilC	1.853	0.551	0	0
8614	3012642	hilC	2.197	0.599	0	0
6432	3012928	hilC	2.054	0.543	0	0
21250	3013378	hilC, STM2868	2.156	0.682	0	0
11719	3013508	STM2868	1.777	0.561	0	0
6210	3013806	STM2868, orgA	1.780	0.708	0	0
4607	3013877	STM2868, orgA	1.753	0.629	0	0
13405	3014169	STM2868, orgA	1.799	0.860	0	0
10670	3014476	orgA, STM2870	1.558	1.070	0	0
21377	3014553	orgA, STM2870	1.626	1.195	0	0
3998	3014930	STM2870	1.637	0.870	0	0

17045	3015095	STM2870, prgK	1.872	0.718	0	0
6533	3015201	STM2870, prgK	1.889	0.690	0	0
7100	3015647	prgK	1.880	0.557	0	0
11385	3015857	prgK, prgJ	2.052	0.623	0	0
21869	3016028	prgK, prgJ	2.435	0.484	0	0
2353	3016264	prgJ, prgI	2.105	0.562	0	0
6612	3016470	prgJ, prgI	2.492	0.623	1	0
8031	3016801	prgH	2.327	0.615	1	0
3482	3017024	prgH	2.426	0.777	0	0
15324	3017216	prgH	2.892	0.772	1	0
6095	3017496	prgH, hilD	2.354	0.580	1	0
22213	3017739	prgH, hilD	2.269	0.506	0	0
7991	3017905	prgH, hilD	2.083	0.526	0	0
12902	3018004	hilD	2.079	0.600	0	0
19853	3018083	hilD	1.920	0.602	0	0
1155	3018588	hilD	2.087	0.612	0	0
16000	3018833		1.804	0.514	0	0
3553	3018992		2.068	0.547	0	0
12308	3019366	hilA	2.963	0.447	1	0
12277	3019476	hilA	2.954	0.481	1	0
7199	3019602	hilA	3.177	0.506	1	0
21727	3019899	hilA	2.268	0.488	1	0
2116	3020112	hilA	2.128	0.548	0	0
18582	3020250	hilA	1.729	0.518	0	0
14522	3020586	hilA	2.051	0.481	0	0
14009	3020900	hilA	3.067	0.569	1	0
6917	3021000	hilA	2.808	0.479	1	0
21321	3021308	hilA, iagB	3.440	0.439	1	0
2244	3021512	iagB	3.031	0.527	1	0
19584	3021688	iagB	3.149	0.621	1	0
16039	3021913	iagB	2.410	0.735	1	0
622	3022102	sptP	1.688	0.775	0	0
5238	3022436	sptP	1.170	0.791	0	0
7288	3022514	sptP	1.093	0.719	0	0
3255	3022984	sptP	1.595	0.656	0	0
9789	3023188	sptP	1.899	0.599	0	0

10828	3023333	sptP	2.307	0.588	1	0
21768	3023415	sptP	2.542	0.505	1	0
4188	3023729	sptP, sicP	2.159	0.443	0	0
15023	3024033	sptP, sicP	2.004	0.562	0	0
8187	3024284	sicP, STM2880	1.925	0.620	0	0
4307	3024463	sicP, STM2880	1.389	0.532	0	0
11545	3024548	STM2880, iacP	1.942	0.651	0	0
21186	3024908	iacP, sipA	1.645	0.693	0	0
7777	3025115	sipA	1.540	0.636	0	0
18532	3025441	sipA	1.611	0.727	0	0
12090	3025621	sipA	2.049	0.683	0	0
1233	3025851	sipA	2.497	0.654	0	0
6788	3025924	sipA	2.523	0.709	0	0
9866	3026130	sipA	2.783	0.900	1	0
6911	3026493	sipA	2.755	0.793	1	0
15347	3026802	sipA, sipD	3.462	0.627	1	0
18700	3026873	sipA, sipD	3.476	0.625	1	0
1292	3027259	sipD	3.365	0.702	1	0
21165	3027356	sipD	3.325	0.629	1	0
21000	3027735	sipD	2.460	0.792	0	0
5299	3027911	sipD, sipC	1.958	0.720	0	0
19902	3028156	sipD, sipC	2.252	0.652	0	0
7497	3028282	sipC	2.067	0.747	0	0
11172	3028600	sipC	2.696	0.616	1	0
10309	3028825	sipC	3.488	0.516	1	0
14231	3028922	sipC	3.325	0.475	1	0
4610	3029205	sipC, sipB	4.537	0.545	1	0
13333	3029356	sipC, sipB	3.308	0.607	1	0
4800	3029440	sipC, sipB	2.661	0.500	1	0
9203	3029727	sipB	1.610	0.615	0	0
3520	3030058	sipB	1.714	0.579	0	0
5483	3030417	sipB	1.620	0.535	0	0
13227	3030502	sipB	1.666	0.581	0	0
10981	3030865	sipB	1.730	0.495	0	0
8095	3030975	sipB, sicA	1.854	0.529	0	0
3045	3031263	sipB, sicA	1.813	0.595	0	0

13146	3031588	sicA, spaS	1.837	0.551	0	0
3459	3031828	sicA, spaS	1.884	0.547	0	0
8710	3031929	spaS	2.127	0.493	0	0
14362	3032001	spaS	2.746	0.560	1	0
21560	3032364	spaS	2.883	0.586	1	0
10399	3032540	spaS	2.673	0.626	1	0
20859	3032896	spaS, spaR	2.384	0.551	1	0
14993	3032996	spaS, spaR	2.050	0.577	0	0
1837	3033428	spaR, spaQ	1.290	0.491	0	0
12991	3033648	spaR, spaQ	1.932	0.491	0	0
2893	3033908	spaQ, spaP	1.942	0.503	0	0
4177	3034091	spaP	2.002	0.489	0	0
9906	3034316	spaP, spaO	2.745	0.492	1	0
17866	3034399	spaP, spaO	3.188	0.542	1	0
12840	3034626	spaP, spaO	2.081	0.501	0	0
21843	3034836	spaO	0.965	0.590	0	0
11740	3035077	spaO	1.239	0.643	0	0
12242	3035348	spaO, invJ	1.634	0.547	0	0
5940	3035581	spaO, invJ	1.702	0.530	0	0
8449	3035917	invJ	2.572	0.470	0	0
1610	3036108	invJ	2.852	0.576	1	0
5794	3036344	invJ, invI	3.590	0.614	1	0
16619	3036603	invJ, invI	3.597	0.643	1	0
2857	3036810	invI, invC	2.570	0.558	1	0
20735	3036974	invI, invC	1.844	0.584	0	0
19380	3037217	invC	1.523	0.648	0	0
18738	3037373	invC	1.399	0.676	0	0
15901	3037612	invC	1.865	0.651	0	0
18018	3037940	invC, invB	2.676	0.580	0	0
4514	3038012	invC, invB	2.194	0.591	0	0
6147	3038429	invB, invA	2.608	0.485	0	0
3316	3038502	invB, invA	2.243	0.518	0	0
3872	3038885	invA	1.990	0.492	0	0
8397	3039067	invA	2.053	0.541	0	0
12332	3039280	invA	1.954	0.592	0	0
437	3039382	invA	1.680	0.699	0	0

17555	3039582	invA	2.002	0.611	0	0
21269	3039806	invA	1.806	0.724	0	0
3260	3040179	invA	1.740	0.602	0	0
592	3040398	invA	1.975	0.521	0	0
9469	3040636	invA, invE	1.917	0.409	0	0
21975	3040765	invA, invE	2.201	0.534	0	0
14980	3040953	invE	1.770	0.545	0	0
13310	3041174	invE	2.029	0.590	0	0
21945	3041593	invE, invG	1.890	0.529	0	0
11791	3041800	invE, invG	2.198	0.548	0	0
5534	3041981	invE, invG	2.162	0.638	0	0
6150	3042247	invG	2.134	0.572	0	0
9359	3042328	invG	2.297	0.600	1	0
22168	3042433	invG	2.308	0.629	1	0
19927	3042896	invG	2.055	0.509	0	0
11249	3043165	invG	2.326	0.555	0	0
1692	3043372	invG, invF	1.826	0.619	0	0
10170	3043624	invG, invF	2.317	0.555	1	0
3988	3043708	invG, invF	2.333	0.479	1	0
13462	3043987	invF, invH	3.398	0.553	1	0
10559	3044360	invF, invH	3.078	0.635	1	0
13397	3044517	invH	3.023	0.553	1	0
11751	3044677	invH	2.650	0.507	1	0
15316	3044807	STM2901	2.734	0.527	1	0
17432	3045047	STM2901	2.014	0.771	0	0
1807	3045444	STM2901, STM2902	1.448	0.640	0	0
17057	3045572	STM2901, STM2902	1.467	0.555	0	0
984	3045699	STM2902	1.873	0.576	0	0
9999	3045973	STM2902	2.298	0.554	1	0
2699	3046261		2.926	0.524	1	0
19920	3046594	STM2903	2.207	0.478	0	0
9344	3046714	STM2903	2.267	0.645	0	0
10842	3046979	STM2903, STM2904	1.258	0.646	0	0
7368	3047213	STM2904, STM2905	0.948	0.626	0	0
8597	3047343	STM2904, STM2905	1.066	0.615	0	0
10676	3047752	STM2905	0.871	0.784	0	0

12912	3047860	STM2905	0.974	0.795	0	0
8000	3047997	STM2905	1.056	0.776	0	0
15074	3048343	STM2906, pphB	2.038	0.728	0	0
9690	3048463	STM2906, pphB	2.833	0.644	1	0
10607	3048813	STM2906, pphB	3.091	0.476	1	0
16351	3049016	pphB	2.686	0.420	1	0
19483	3049345	pphB	2.143	0.551	0	0
8423	3049598	STM2908	2.216	0.659	0	0
20200	3049730	STM2908	1.728	0.706	0	0
15997	3049882	STM2908, mutS	1.146	0.785	0	0
17218	3050082	STM2908, mutS	0.795	0.889	0	0
2339	3050354	STM2908, mutS	0.841	0.816	0	0
4023	3050512	STM2908, mutS	1.079	0.808	0	0
3307	3050619	mutS	0.944	0.866	0	0
4210	3051004	mutS	0.740	0.732	0	0
7258	3051164	mutS	0.853	0.690	0	0
5128	3051383	mutS	1.354	1.005	0	0
10146	3051780	mutS	1.755	1.032	0	0
14480	3051853	mutS	1.499	1.008	0	0
21724	3052299	mutS	1.495	1.048	0	0
650	3052431	mutS	1.351	1.352	0	0
442	3052741	mutS	1.040	1.157	0	0
10046	3052906	STM2910	1.198	1.040	0	0
19991	3053213	STM2910	1.420	0.805	0	0
466	3053286	STM2910, STM2911	1.494	0.799	0	0
864	3053620	STM2910, STM2911	1.545	0.774	0	0
13584	3053799	STM2911	0.676	0.614	0	0
19474	3054105	STM2911, STM2912	1.009	0.656	0	0
20826	3054293	STM2911, STM2912	1.653	0.857	0	0
19969	3054422	STM2911, STM2912	1.451	0.891	0	0
4753	3054677	STM2911, STM2912	2.024	0.916	0	0
15260	3054785	STM2911, STM2912	2.007	1.284	0	0
989	3055153	STM2912	1.566	1.634	0	0
8636	3055422	STM2912	1.327	1.469	0	0
12853	3055526	STM2913	1.027	1.258	0	0
6792	3055724	STM2913	0.591	0.827	0	0

17456	3056076	STM2913	1.002	1.609	0	0
10967	3056313	STM2913	1.026	1.454	0	0
16285	3056419	STM2913	1.162	1.667	0	0
9195	3056658	STM2913	1.370	1.403	0	0
20913	3057029	STM2913, STM2914	1.143	1.308	0	0
908	3057227	STM2913, STM2914	0.792	0.939	0	0
5921	3057385	STM2913, STM2914	1.174	1.148	0	0
9511	3057749	STM2914	1.233	1.203	0	0
15769	3057977	STM2914	0.533	0.844	0	0
635	3058107	STM2914, ygbM	0.979	1.317	0	0
15991	3058236	STM2914, ygbM	1.268	1.595	0	0
20108	3058340	STM2914, ygbM	1.299	1.516	0	0
8394	3058803	ygbM, ygbL	1.565	1.696	0	0
3079	3058874	ygbM, ygbL	1.443	1.645	0	0
3734	3059173	ygbM, ygbL	1.288	1.766	0	0
3289	3059407	ygbL	1.264	1.792	0	1
16154	3059556	ygbL, ygbK	1.290	1.806	0	1
19357	3059887	ygbL, ygbK	1.086	1.419	0	0
12479	3060083	ygbK	1.249	1.531	0	0
1789	3060438	ygbK	1.298	1.741	0	0
10523	3060598	ygbK	0.838	1.454	0	0
17799	3060735	ygbK, ygbJ	1.250	2.139	0	1
19237	3060911	ygbK, ygbJ	1.180	1.965	0	1
17269	3061342	ygbJ, ygbI	0.643	1.129	0	0
20720	3061605	ygbJ, ygbI	0.688	1.220	0	0
14972	3061839	ygbJ, ygbI	0.696	1.204	0	0
12858	3061913	ygbJ, ygbI	0.728	1.167	0	0
16857	3062188	ygbI	0.975	1.610	0	0
18917	3062330	ygbI	1.082	1.652	0	0
6611	3062729	STM2920, STM2921	0.907	1.525	0	0
17749	3062979	STM2920, STM2921	0.902	1.049	0	0
1931	3063154	STM2920, STM2921	0.899	0.777	0	0
8417	3063254	STM2920, STM2921	1.130	0.768	0	0
15131	3063556	STM2921, STM2922	0.607	0.585	0	0
6787	3063729	STM2921, STM2922	0.952	0.546	0	0
16303	3064014	STM2922	1.078	0.664	0	0

9388	3064236	STM2922	0.979	0.600	0	0
18852	3064522	STM2922	1.204	0.704	0	0
3185	3064637	STM2922	0.993	0.672	0	0
14769	3064894	STM2922, STM2923	0.886	0.916	0	0
9688	3065194	STM2923	0.930	1.469	0	0
16470	3065265	STM2923	0.806	1.729	0	1
14311	3065686	rpoS	0.502	2.668	0	1
356	3065839	rpoS	0.530	3.315	0	1
7748	3065953	rpoS	0.466	2.579	0	1
14859	3066379	rpoS	0.518	2.853	0	1
7645	3066450	rpoS	0.486	2.325	0	1
7115	3066571	rpoS, nlpD	0.501	2.566	0	1
8037	3067026	nlpD	0.490	2.428	0	1
20529	3067269	nlpD	0.697	1.792	0	1
14171	3067340	nlpD	0.646	1.620	0	0
14940	3067676	nlpD	0.890	1.451	0	0
6681	3067787	nlpD	0.769	1.305	0	0
10511	3068163	pcm	0.914	1.603	0	0
19050	3068330	pcm	0.996	1.756	0	0
8659	3068471	pcm, surE	0.761	1.371	0	0
3606	3068841	pcm, surE	1.149	1.748	0	1
8134	3069054	surE	1.100	1.961	0	1
13916	3069211	surE, ygbO	1.000	1.650	0	0
15550	3069309	surE, ygbO	1.010	1.569	0	0
17563	3069609	surE, ygbO	1.242	1.513	0	0
19696	3069926	ygbO	1.023	1.263	0	0
15549	3070212	ygbO	0.718	0.846	0	0
11613	3070347	ygbO, ispF	0.985	1.018	0	0
9498	3070687	ygbO, ispF	1.133	1.209	0	0
21375	3070813	ispF, ispD	1.243	1.568	0	0
1841	3071075	ispF, ispD	0.680	1.164	0	0
9484	3071322	ispD	0.888	2.197	0	1
8089	3071428	ispD	0.791	2.174	0	1
9559	3071696	ispD, ygbQ	0.783	2.031	0	1
20256	3071888	ispD, ygbQ	0.920	1.700	0	0
19737	3072261	ygbE	1.264	1.015	0	0

1538	3072381	ygbE, cysC	1.212	1.020	0	0
6114	3072722	ygbE, cysC	0.489	0.589	0	0
3630	3072916	cysC, cysN	0.368	0.434	0	0
16092	3073201	cysC, cysN	0.902	0.622	0	0
1249	3073426	cysN	1.113	0.844	0	0
4783	3073600	cysN	1.344	0.810	0	0
9208	3073871	cysN	0.654	0.730	0	0
12703	3074051	cysN	1.070	0.893	0	0
5582	3074171	cysN	1.520	1.045	0	0
6337	3074576	cysN, cysD	1.160	0.752	0	0
5932	3074684	cysN, cysD	1.105	0.724	0	0
18715	3074809	cysN, cysD	1.259	0.738	0	0
3977	3075197	cysD, iap	0.840	0.606	0	0
17307	3075272	cysD, iap	1.130	0.810	0	0
14496	3075350	cysD, iap	0.967	0.592	0	0
944	3075440	cysD, iap	1.472	0.754	0	0
4504	3076115	iap	1.146	0.672	0	0
11802	3076356	iap	1.385	0.856	0	0
600	3076559	iap	1.411	0.839	0	0
19956	3076810		0.721	0.513	0	0
1987	3076999		1.482	0.646	0	0
21517	3077219		1.119	0.572	0	0
14609	3077463		1.006	0.495	0	0
9261	3077581		0.521	0.661	0	0
15310	3077764		0.968	0.757	0	0
9188	3078163		1.497	0.642	0	0
9686	3078257	ygbF	1.509	0.616	0	0
6481	3078390	ygbF	1.443	0.634	0	0
9442	3078641	ygbF, STM2938	1.431	0.771	0	0
13716	3078795	ygbF, STM2938	1.429	0.738	0	0
22209	3079368	STM2938	1.169	0.587	0	0
7420	3079553	STM2938, ygcH	0.846	0.632	0	0
10697	3079718	STM2938, ygcH	0.582	0.509	0	0
2200	3079915	ygcH	1.504	0.797	0	0
12781	3080193	ygcH, STM2940	1.302	0.873	0	0
6473	3080264	ygcH, STM2940	1.627	0.934	0	0

9221	3080632	STM2940	0.981	0.765	0	0
18152	3080919	STM2940, yghJ	1.420	0.680	0	0
20642	3081146	STM2940, yghJ	1.247	0.716	0	0
1214	3081369	yghJ	1.324	0.707	0	0
5692	3081550	yghJ	1.167	0.567	0	0
19023	3081699	yghJ	1.289	0.598	0	0
13193	3082047	yghJ, STM2942	1.117	0.668	0	0
12026	3082266	yghJ, STM2942	1.049	0.655	0	0
22126	3082401	STM2942	1.031	0.751	0	0
21502	3082602	STM2942, STM2943	1.185	0.693	0	0
16294	3082701	STM2942, STM2943	1.324	0.717	0	0
981	3082846	STM2942, STM2943	0.970	0.567	0	0
2965	3083154	STM2943	1.736	0.739	0	0
10135	3083673	STM2943	1.564	0.697	0	0
15727	3083785	STM2943	0.954	0.561	0	0
21720	3084017	STM2943, ygcB	1.283	0.749	0	0
9495	3084168	STM2943, ygcB	1.448	0.742	0	0
9308	3084419	STM2943, ygcB	1.475	0.671	0	0
7360	3084772	ygcB	1.445	0.671	0	0
20389	3084908	ygcB	1.431	0.651	0	0
15620	3085005	ygcB	1.048	0.545	0	0
18900	3085371	ygcB	1.223	0.614	0	0
10841	3085559	ygcB	1.501	0.633	0	0
5764	3085869	ygcB	1.521	0.558	0	0
3448	3086102	ygcB	1.983	0.618	0	0
20009	3086297	ygcB	3.024	0.546	1	0
1213	3086434	ygcB	4.442	0.616	1	0
7364	3086654	ygcB, sopD	4.716	0.615	1	0
19865	3087007	ygcB, sopD	3.397	0.432	1	0
16935	3087219	sopD	3.105	0.536	1	0
9917	3087338	sopD	2.810	0.515	1	0
20255	3087640	sopD	3.486	0.560	1	0
20053	3087826	sopD	3.426	0.502	1	0
975	3088141		3.484	0.581	1	0
20237	3088337	cysH	3.053	0.600	1	0
20662	3088607	cysH	1.974	0.728	0	0

13475	3088880	cysH	1.618	0.616	0	0
16692	3089104	cysH, cysI	1.529	0.719	0	0
19591	3089295	cysH, cysI	1.786	0.691	0	0
18631	3089599	cysI	1.956	0.634	0	0
20797	3089751	cysI	0.722	0.607	0	0
3965	3089880	cysI	1.541	0.643	0	0
16925	3090022	cysI	1.635	0.719	0	0
16387	3090193	cysI	1.482	0.698	0	0
5928	3090524	cysI	0.915	0.468	0	0
12460	3090707	cysI, cysJ	1.556	0.655	0	0
1378	3090914	cysI, cysJ	1.767	0.717	0	0
20040	3091013	cysI, cysJ	1.374	0.633	0	0
17369	3091270	cysJ	0.945	0.731	0	0
6803	3091718	cysJ	1.354	0.686	0	0
9252	3092001	cysJ	1.544	0.553	0	0
15121	3092153	cysJ	1.783	0.780	0	0
10016	3092520	cysJ, ptpS	2.907	0.671	0	0
644	3092669	cysJ, ptpS	2.542	0.742	0	0
21090	3092843	cysJ, ptpS	3.116	0.774	0	0
14147	3093119	ptpS	2.020	0.946	0	0
16662	3093314		1.288	1.063	0	0
748	3093629	STM2950	1.453	1.009	0	0
4843	3093862	STM2950	1.446	0.905	0	0
4719	3094139	STM2950	1.326	0.944	0	0
3050	3094235	STM2950	0.945	0.777	0	0
8901	3094357	STM2950	0.715	0.590	0	0
6390	3094699		1.508	0.813	0	0
15021	3094940		0.904	0.580	0	0
6470	3095146		0.851	0.565	0	0
5790	3095389		0.660	0.651	0	0
12834	3095521		0.624	0.548	0	0
1131	3095904		1.578	0.813	0	0
15341	3096126		1.615	0.931	0	0
706	3096247		1.805	0.760	0	0
19458	3096473		1.773	0.761	0	0
1026	3096573	ygcF	1.798	1.142	0	0

14578	3097054	ygcF	0.892	1.905	0	0
20932	3097189	ygcF	0.814	1.618	0	0
7679	3097334	ygcF, eno	0.753	1.541	0	0
14796	3097419	ygcF, eno	0.839	1.327	0	0
9855	3097613	ygcF, eno	0.671	1.180	0	0
1850	3098067	eno	1.448	1.326	0	0
21174	3098329	eno	0.790	1.488	0	0
13604	3098614	eno	0.750	1.810	0	0
9775	3098843	eno, pyrG	0.476	0.994	0	0
2117	3098930	eno, pyrG	0.822	1.574	0	0
11810	3099215	pyrG	0.626	1.278	0	0
10867	3099540	pyrG	0.975	1.204	0	0
14017	3099666	pyrG	1.005	1.332	0	0
14551	3099770	pyrG	0.892	1.260	0	0
20546	3100224	pyrG	0.771	2.196	0	1
16601	3100386	pyrG	0.866	2.512	0	1
12929	3100462	pyrG	0.806	2.112	0	1
9774	3100540	pyrG	0.852	1.949	0	1
19599	3100697	pyrG, mazG	0.990	1.315	0	0
6193	3101367	mazG	2.736	0.649	0	0
7839	3101438	mazG	3.119	0.446	1	0
8673	3101672	mazG	2.851	0.586	1	0
2979	3101958		3.573	0.646	1	0
16893	3102099		2.879	0.726	0	0
21166	3102459	STM2955	3.239	1.067	0	0
14123	3102611	STM2955	2.035	1.179	0	0
12659	3102694	STM2955	1.451	1.038	0	0
14466	3102952	STM2955, relA	1.032	1.506	0	0
5631	3103410	relA	1.189	1.290	0	0
9529	3103569	relA	0.937	1.236	0	0
9837	3103698	relA	0.899	1.033	0	0
4903	3104065	relA	0.904	1.602	0	0
8477	3104309	relA	0.890	1.297	0	0
7338	3104499	relA	0.780	1.272	0	0
4455	3104689	relA	0.970	1.412	0	0
5667	3104789	relA	0.895	1.571	0	0

13261	3105049	relA	0.912	1.800	0	0
10364	3105443	relA, ygcA	1.045	1.683	0	0
17046	3105660	ygcA	1.136	1.699	0	0
5791	3105766	ygcA	0.926	1.350	0	0
5555	3105984	ygcA	1.064	1.542	0	0
19252	3106288	ygcA, barA	1.040	1.145	0	0
17955	3106474	ygcA, barA	1.016	1.110	0	0
13632	3106788	ygcA, barA	1.152	1.109	0	0
21191	3106998	barA	1.008	0.761	0	0
8330	3107284	barA	1.228	0.785	0	0
14050	3107517	barA	1.243	0.863	0	0
17482	3107744	barA	1.516	0.846	0	0
2557	3107815	barA	1.235	0.827	0	0
15791	3108042	barA	1.307	0.961	0	0
11312	3108449	barA	1.113	0.874	0	0
7695	3108573	barA	1.152	0.902	0	0
409	3108805	barA	1.442	1.175	0	0
12522	3108999	barA	1.269	1.574	0	0
20914	3109227	barA	1.136	1.449	0	0
9515	3109417	STM2959	0.929	1.514	0	1
12018	3109496	STM2959	1.072	1.657	0	1
13968	3109597	STM2959	1.051	1.441	0	0
8444	3110190	STM2959	1.212	1.486	0	0
11289	3110454	STM2959	0.903	1.273	0	0
6271	3110621	STM2959, gudD	1.021	1.219	0	0
21293	3110723	STM2959, gudD	1.073	1.288	0	0
13537	3111095	gudD	1.063	1.380	0	0
9923	3111257	gudD	1.198	1.509	0	0
14718	3111520	gudD	0.874	1.049	0	0
21386	3111825	gudD	1.077	1.288	0	0
17404	3111987	gudD, ygcY	1.388	1.635	0	0
6529	3112184	gudD, ygcY	1.270	1.410	0	0
788	3112459	ygcY	0.852	1.154	0	0
3948	3112743	ygcY	1.251	1.482	0	0
1175	3112922	ygcY	0.971	1.355	0	0
5360	3113212	ygcY, gudT	1.032	1.347	0	0

9769	3113376	ygcY, gudT	1.164	1.516	0	0
15758	3113530	ygcY, gudT	0.999	1.510	0	0
19720	3113801	gudT	0.899	1.209	0	0
19690	3113970	gudT	0.975	1.258	0	0
20959	3114294	gudT	0.900	1.398	0	0
11063	3114580	gudT	0.723	1.401	0	0
2289	3114651	gudT	0.680	1.274	0	0
19804	3114873	gudT	0.762	1.408	0	0
18757	3115000	gudT	0.781	1.732	0	0
22000	3115200	STM2963	0.825	1.558	0	0
1871	3115509	STM2963	0.905	1.599	0	0
15927	3115679	STM2963, yqcB	0.801	1.149	0	0
5488	3115767	STM2963, yqcB	1.057	1.288	0	0
14148	3116277	yqcB	0.516	0.871	0	0
18263	3116454	yqcB, yqcC	0.808	2.083	0	1
11459	3116744	yqcB, yqcC	0.462	2.374	0	1
17891	3117045	yqcC, csrB	0.530	2.487	0	1
1429	3117212	csrB	0.535	2.542	0	1
12662	3117343	csrB, syd	0.467	2.413	0	1
17390	3117678	syd, yqcD	0.581	2.748	0	1
7932	3117848	syd, yqcD	0.657	1.777	0	0
6215	3118129	syd, yqcD	0.950	1.540	0	0
13967	3118330	yqcD	0.846	1.238	0	0
19672	3118545	yqcD, ygdH	0.814	1.258	0	0
13357	3118753	ygdH	0.834	1.362	0	0
13693	3118915	ygdH	0.682	1.406	0	0
6327	3119195	ygdH	0.623	1.018	0	0
16916	3119526	ygdH	0.745	1.286	0	0
11917	3119628	ygdH	0.891	1.372	0	0
15708	3119934	ygdH	0.742	1.498	0	0
721	3120061	ygdH	0.830	1.519	0	0
4001	3120377	sdaC	0.500	1.620	0	0
21572	3120555	sdaC	0.488	2.073	0	1
8109	3120739	sdaC	0.387	2.543	0	1
3314	3121059	sdaC	0.356	3.440	0	1
3435	3121219	sdaC	0.467	3.985	0	1

5224	3121501	sdaC	0.354	2.325	0	1
4064	3121744	sdaC, sdaB	0.494	3.161	0	1
12865	3121965	sdaC, sdaB	0.338	2.509	0	1
13079	3122082	sdaB	0.358	2.593	0	1
4699	3122402	sdaB	0.490	2.936	0	1
1287	3122575	sdaB	0.543	3.840	0	1
3643	3122859	sdaB	0.337	1.267	0	0
5810	3123006	sdaB	0.551	3.712	0	1
7054	3123356	sdaB, exo	0.543	3.181	0	1
20380	3123455	sdaB, exo	0.605	3.384	0	1
929	3123752	exo	0.430	1.127	0	0
6079	3123988	exo	0.544	0.981	0	0
17220	3124225	exo	1.210	1.457	0	0
9449	3124343	exo	0.987	1.054	0	0
18855	3124755	fucO	1.135	0.935	0	0
2946	3124857	fucO	0.354	0.508	0	0
11331	3125161	fucO	1.142	0.808	0	0
3146	3125395	fucO	1.382	0.708	0	0
12692	3125651	fucO, fucA	0.865	0.455	0	0
21973	3125807	fucO, fucA	1.552	0.655	0	0
20110	3126118	fucA	3.210	0.653	1	0
14474	3126298	fucA	5.192	0.551	1	0
10298	3126377	fucA	5.241	0.541	1	0
20957	3126839		5.084	0.626	1	0
7148	3126998		6.536	0.574	1	0
1011	3127123		5.829	0.518	1	0
1811	3127507		3.652	0.602	1	0
20977	3127578		3.465	0.807	1	0
18408	3127972	fucI	1.065	0.511	0	0
15160	3128175	fucI	1.408	0.635	0	0
20947	3128259	fucI	1.416	0.665	0	0
12746	3128615	fucI	1.286	0.830	0	0
8689	3128788	fucI	1.070	0.648	0	0
11357	3129025	fucI	1.346	0.676	0	0
8072	3129137	fucI	1.410	0.600	0	0
16058	3129584	fucI	0.818	0.528	0	0

12172	3129671	fucI, fucK	0.798	0.704	0	0
19024	3129853	fucI, fucK	1.449	0.649	0	0
11624	3130079	fucK	1.707	0.704	0	0
11084	3130280	fucK	0.526	0.561	0	0
21915	3130569	fucK	1.642	0.875	0	0
4660	3130910	fucK	1.295	0.673	0	0
2484	3131013	fucK	1.536	0.754	0	0
19864	3131307	fucK, fucU	2.205	0.958	0	0
20606	3131507	fucU, fucR	1.544	1.001	0	0
13906	3131653	fucU, fucR	1.437	1.161	0	0
2076	3131867	fucU, fucR	1.554	1.719	0	0
20698	3132126	fucR	1.201	1.540	0	0
10780	3132334	fucR	1.180	1.481	0	0
18910	3132467	fucR	1.114	1.498	0	0
20303	3132910	ygdE	0.914	1.785	0	0
20208	3133022	ygdE	0.874	1.360	0	0
5439	3133341	ygdE	1.321	1.067	0	0
10621	3133494	ygdE	1.047	0.976	0	0
300	3133754	ygdE	1.229	0.944	0	0
7158	3134114	ygdE, ygdD	0.897	0.852	0	0
7214	3134248	ygdE, ygdD	1.040	0.869	0	0
17633	3134374	ygdD, gcvA	1.112	1.072	0	0
14918	3134526	ygdD, gcvA	1.099	1.286	0	0
17471	3134937	gcvA	0.757	1.949	0	1
10753	3135153	gcvA	0.607	2.423	0	1
6547	3135425	gcvA	0.394	2.866	0	1
13299	3135549	gcvA, ygdI	0.417	2.930	0	1
12730	3135748	ygdI, csdA	0.669	2.157	0	1
6247	3135854	ygdI, csdA	0.858	1.790	0	1
5428	3135925	ygdI, csdA	0.875	1.740	0	1
16198	3136572	csdA	1.095	0.996	0	0
3996	3136701	csdA, ygdK	1.205	1.011	0	0
20584	3137064	csdA, ygdK	1.124	0.833	0	0
16992	3137227	ygdK	2.013	0.953	0	0
284	3137364	ygdK	2.139	0.825	1	0
2903	3137717	STM2986	3.925	0.599	1	0

6807	3137820	STM2986	4.500	0.605	1	0
8497	3138120	STM2986	4.707	0.577	1	0
4460	3138385	STM2986	3.914	0.545	1	0
9062	3138664	STM2986	3.586	0.597	1	0
16829	3138859	ygdL	2.576	0.915	1	0
4284	3139033	ygdL	1.191	0.727	0	0
9966	3139243	ygdL	1.349	1.070	0	0
10605	3139423	ygdL	1.079	1.273	0	0
612	3139623	ygdL	1.101	1.168	0	0
11765	3139908	ygdL, mltA	1.072	1.279	0	0
14419	3140140	mltA	0.787	1.009	0	0
5980	3140319	mltA	0.861	1.227	0	0
10895	3140582	mltA, metZ	0.802	1.540	0	1
6326	3140886	mltA, metZ	0.606	3.114	0	1
22333	3140998	mltA, metZ	0.514	4.126	0	1
4928	3141240	mltA	0.475	2.749	0	1
9805	3141571	amiC	0.671	1.936	0	1
19788	3141642	amiC	0.656	1.747	0	1
10151	3141879	amiC	0.791	1.673	0	1
14783	3142244	amiC	0.761	1.266	0	0
12882	3142366	amiC, argA	0.735	1.348	0	0
19824	3142746	amiC, argA	0.720	1.129	0	0
5835	3142877	amiC, argA	0.715	0.976	0	0
8836	3142952	amiC, argA	0.721	0.893	0	0
19197	3143434	argA	1.038	0.749	0	0
1340	3143637	argA	1.104	0.939	0	0
6146	3143813	argA	1.229	1.007	0	0
12863	3144068	argA	0.779	0.995	0	0
16430	3144257	recD	1.023	1.477	0	0
17490	3144468	recD	1.137	1.631	0	0
12691	3144784	recD	1.646	1.321	0	0
8019	3144898	recD	1.414	1.428	0	0
18094	3145015	recD	1.248	1.180	0	0
22013	3145253	recD	1.521	1.417	0	0
3154	3145657	recD	1.516	1.364	0	0
19186	3145854	recD	1.417	1.226	0	0

7390	3146106	recD, recB	1.178	1.205	0	0
14552	3146257	recD, recB	1.374	1.323	0	0
11944	3146395	recD, recB	1.181	1.190	0	0
13041	3146740	recB	1.022	1.042	0	0
20021	3146881	recB	1.139	1.198	0	0
5257	3147029	recB	1.032	1.064	0	0
14303	3147408	recB	2.307	1.332	0	0
14946	3147715	recB	0.750	0.955	0	0
7641	3147883	recB	1.762	1.154	0	0
5878	3148213	recB	1.499	1.395	0	0
10743	3148443	recB	0.880	1.057	0	0
2684	3148666	recB	1.206	1.205	0	0
11931	3148763	recB	1.121	1.337	0	0
11090	3149050	recB	0.792	0.986	0	0
19826	3149180	recB	1.218	1.356	0	0
5944	3149326	recB	1.119	1.335	0	0
14275	3149817	recB, ptr	1.153	1.426	0	0
6245	3149940	recB, ptr	1.156	1.322	0	0
13947	3150144	ptr	1.043	1.014	0	0
14265	3150286	ptr	0.864	0.973	0	0
6538	3150408	ptr	1.034	0.863	0	0
4593	3150701	ptr	0.689	0.808	0	0
10422	3151050	ptr	0.894	0.758	0	0
21394	3151365	ptr	0.985	0.839	0	0
18285	3151492	ptr	0.851	0.822	0	0
11644	3151725	ptr	1.055	0.866	0	0
2543	3151845	ptr	1.028	0.828	0	0
10492	3152229	ptr	0.905	0.836	0	0
21388	3152529	ptr	0.920	1.003	0	0
16011	3152620	ptr	0.584	0.660	0	0
4986	3152703	ptr, recC	0.922	0.913	0	0
20035	3152853	ptr, recC	0.989	1.073	0	0
9854	3153424	recC	1.197	0.993	0	0
8811	3153666	recC	1.253	0.925	0	0
9615	3153811	recC	1.166	0.787	0	0
18079	3154049	recC	0.722	0.705	0	0

533	3154274	recC	1.193	0.984	0	0
8664	3154577	recC	0.923	0.751	0	0
9813	3154803	recC	1.450	0.961	0	0
19403	3154940	recC	1.583	0.955	0	0
21492	3155101	recC	1.407	0.821	0	0
12181	3155336	recC	1.470	0.795	0	0
20326	3155623	recC	1.261	0.920	0	0
3302	3155789	recC	1.252	0.814	0	0
14341	3156064	recC	1.297	1.031	0	0
13666	3156269	recC, ppdC	1.072	0.840	0	0
8237	3156533	ppdC, ygdB	0.811	0.784	0	0
17106	3156724	ppdC, ygdB	1.047	0.576	0	0
5231	3156976	ygdB, ppdB	1.187	0.719	0	0
6653	3157151	ygdB, ppdB	0.964	0.639	0	0
11675	3157249	ygdB, ppdB	1.256	0.756	0	0
17118	3157545	ppdB, ppdA	0.897	0.623	0	0
914	3157769	ppdB, ppdA	1.110	0.895	0	0
17142	3158052	ppdA, thyA	1.137	1.414	0	0
8244	3158316	thyA	1.095	1.474	0	0
19230	3158674	thyA	0.856	1.338	0	0
3725	3158777	thyA	0.891	1.285	0	0
8233	3159044	thyA, lgt	0.659	1.023	0	0
539	3159206	thyA, lgt	0.885	1.562	0	0
6064	3159411	lgt	1.135	1.463	0	0
19344	3159783	lgt	0.752	2.071	0	1
9076	3159883	lgt, ptsP	0.596	1.908	0	1
18159	3160005	lgt, ptsP	0.832	2.224	0	0
2815	3160308	ptsP	0.896	1.819	0	1
8682	3160651	ptsP	0.834	1.775	0	1
13938	3160895	ptsP	0.981	1.595	0	0
6737	3160970	ptsP	0.587	1.042	0	0
20030	3161276	ptsP	0.935	1.781	0	0
11785	3161516	ptsP	0.842	1.872	0	1
20245	3161831	ptsP	0.778	2.086	0	1
21478	3162031	ptsP	0.715	2.133	0	1
16122	3162200	ptsP, ygdP	0.668	1.951	0	1

313	3162454	ptsP, ygdP	0.743	1.715	0	0
4197	3162724	ygdP	0.712	0.893	0	0
21003	3162876	ygdP	0.843	0.809	0	0
18853	3163182	mutH	0.808	0.758	0	0
20705	3163351	mutH	0.797	0.927	0	0
848	3163606	mutH	0.660	0.913	0	0
9859	3163770	mutH, ygdQ	0.863	1.395	0	0
13084	3163978	mutH, ygdQ	0.751	1.326	0	0
15496	3164224	ygdQ	0.998	2.014	0	1
22211	3164316	ygdQ	0.948	1.854	0	1
20252	3164825	ygdQ, ygdR	0.907	1.018	0	0
15785	3164905	ygdQ, ygdR	1.012	1.017	0	0
18638	3165094	ygdR, tas	1.083	0.880	0	0
5185	3165224	ygdR, tas	1.045	1.029	0	0
4483	3165518	tas	0.903	0.959	0	0
21545	3165888	tas	1.170	1.001	0	0
3178	3166038	tas	1.388	1.084	0	0
10952	3166127	tas	1.037	1.062	0	0
18523	3166221	tas	0.992	1.051	0	0
8419	3166491	tas	1.097	1.491	0	0
19851	3166791	ygeD	1.060	1.543	0	0
14266	3167214	ygeD	1.237	1.394	0	0
10564	3167474	ygeD	1.168	1.431	0	0
9984	3167545	ygeD	1.027	1.372	0	0
3331	3167829	ygeD	1.010	1.459	0	0
16880	3168006	ygeD, aas	1.083	1.271	0	0
5953	3168199	ygeD, aas	1.036	1.059	0	0
16872	3168507	aas	0.987	1.213	0	0
444	3168624	aas	1.331	1.276	0	0
6537	3168819	aas	1.060	1.117	0	0
6526	3169276	aas	1.138	1.077	0	0
3466	3169434	aas	0.560	0.910	0	0
13477	3169820	aas	1.058	1.257	0	0
4502	3169899	aas	0.902	1.020	0	0
6101	3170064	aas	0.948	1.038	0	0
14589	3170373	aas, galR	0.739	1.057	0	0

7717	3170467	aas, galR	0.726	1.187	0	0
1430	3170898	galR	1.283	1.579	0	0
16933	3171136	galR	1.043	1.580	0	0
17601	3171379	galR, STM3012	0.548	0.946	0	0
20131	3171542	galR, STM3012	1.025	1.884	0	0
19508	3171631	STM3012	0.945	1.599	0	0
7528	3172015	STM3012	1.457	1.685	0	0
2067	3172172	STM3012	1.319	1.921	0	0
9251	3172521	STM3012	1.255	1.621	0	0
9640	3172664		1.064	1.458	0	0
7429	3172880	lysA	1.103	1.132	0	0
11773	3173039	lysA	0.978	0.970	0	0
9595	3173351	lysA	1.351	0.871	0	0
15842	3173639	lysA, lysR	1.073	0.801	0	0
2897	3173884	lysA, lysR	1.376	0.754	0	0
19681	3174023	lysA, lysR	1.318	0.635	0	0
2065	3174260	lysA, lysR	1.156	0.964	0	0
12053	3174543	lysR	0.921	1.020	0	0
20129	3174647	lysR	0.860	1.057	0	0
10544	3174928	lysR	1.138	1.008	0	0
5215	3175174	ygeA	1.470	1.159	0	0
7058	3175377	ygeA	0.641	0.557	0	0
2450	3175689	ygeA	1.133	0.828	0	0
13275	3175861	ygeA, araE	1.313	0.796	0	0
16528	3175972	ygeA, araE	1.168	0.715	0	0
5255	3176182	araE	0.552	0.578	0	0
16797	3176470	araE	1.186	0.805	0	0
3202	3176708	araE	1.107	0.698	0	0
489	3176784	araE	1.812	0.740	0	0
21926	3177226	araE	3.023	0.558	0	0
8144	3177471	araE	2.492	0.690	0	0
1957	3177744	kduD	1.864	0.773	0	0
21035	3177978	kduD	1.900	0.823	0	0
11161	3178187	kduD	1.078	0.681	0	0
18166	3178284	kduD	1.158	0.642	0	0
2526	3178490	kduD, kduI	1.219	0.618	0	0

16519	3178696	kduD, kduI	1.287	0.620	0	0
21592	3179085	kduI	1.822	0.782	0	0
11782	3179211	kduI	1.632	0.722	0	0
21979	3179475	kduI	1.173	0.967	0	0
21311	3179599	kduI	1.051	1.270	0	0
8430	3179813	yqeF	1.076	1.854	0	0
5146	3180193	yqeF	0.845	1.599	0	0
395	3180367	yqeF	0.782	1.567	0	0
17735	3180549	yqeF	1.091	1.903	0	0
10915	3180907	yqeF	1.249	1.364	0	0
22094	3181112	yqeF, STM3020	1.044	1.119	0	0
1471	3181375	STM3020	0.983	0.797	0	0
226	3181485	STM3020, STM3021	1.430	0.789	0	0
14352	3181770	STM3020, STM3021	1.318	0.749	0	0
4418	3181898	STM3020, STM3021	0.971	0.667	0	0
8466	3182235	STM3020, STM3021	1.043	0.840	0	0
9004	3182515	STM3022	1.655	0.874	0	0
9084	3182667	STM3022	2.070	0.804	0	0
12147	3182981	STM3022	2.264	0.754	0	0
4589	3183197	STM3022	2.056	0.853	0	0
12841	3183379	STM3022	2.128	0.866	0	0
9159	3183656	STM3022	2.063	0.833	0	0
21318	3183811	yohL, yohM	2.012	0.844	0	0
13510	3183975	yohL, yohM	1.196	0.738	0	0
13701	3184155	yohL, yohM	1.560	0.822	0	0
1161	3184414	yohL, yohM	1.182	0.768	0	0
1289	3184693	yohM	0.897	0.768	0	0
17985	3184768	yohM	0.912	0.765	0	0
22325	3185005	yohM	1.613	1.060	0	0
7643	3185079		1.521	0.865	0	0
19593	3185491	STM3025	1.307	1.079	0	0
1438	3185776	STM3025	1.360	0.841	0	0
10165	3185996	STM3025	1.374	0.755	0	0
17169	3186159	STM3025	2.537	0.632	1	0
8249	3186440	STM3025	4.255	0.477	1	0
381	3186656	STM3026	3.728	0.600	1	0

13366	3187069	STM3026	3.037	0.515	1	0
8029	3187251	STM3026	3.278	0.477	1	0
6809	3187359	STM3026	3.297	0.465	1	0
21989	3187471	STM3026	4.478	0.574	1	0
1071	3187600	STM3026	3.996	0.714	1	0
11551	3187679	STM3026	3.861	0.630	1	0
16669	3188433	stdC	1.275	0.657	0	0
4409	3188504	stdC, stdB	1.283	0.507	0	0
11517	3188732	stdC, stdB	1.225	0.628	0	0
12455	3188954	stdB	0.650	0.637	0	0
11151	3189192	stdB	1.091	0.584	0	0
20491	3189410	stdB	1.260	0.640	0	0
11139	3189636	stdB	1.725	0.555	0	0
16998	3189831	stdB	1.946	0.765	0	0
7858	3190104	stdB	0.832	0.404	0	0
12503	3190238	stdB	1.842	0.712	0	0
10561	3190668	stdB	1.757	0.595	0	0
6870	3190788	stdB	1.440	0.552	0	0
6595	3191101	stdB, stdA	1.178	0.504	0	0
18820	3191343	stdB, stdA	2.182	0.590	0	0
11422	3191554	stdA	3.740	0.592	1	0
21076	3191810	stdA	3.999	0.495	1	0
17133	3191983	stdA	3.076	0.501	1	0
7462	3192290		2.709	0.540	1	0
280	3192462	STM3030	2.826	0.617	1	0
10360	3192558	STM3030	2.820	0.542	1	0
12824	3192792	STM3030	2.694	0.576	1	0
2393	3193115	STM3030, STM3031	2.514	0.613	0	0
9110	3193233	STM3030, STM3031	2.976	0.647	1	0
4639	3193326	STM3030, STM3031	3.223	0.581	1	0
18695	3193617	STM3031	4.307	0.583	1	0
697	3194064	STM3031	2.954	0.715	1	0
7514	3194156		1.790	0.743	0	0
17386	3194437	tnpA_4	1.354	0.928	0	0
3142	3194638	tnpA_4	1.328	0.803	0	0
13991	3194915	tnpA_4	1.547	1.027	0	0

10457	3195044	tnpA_4	1.396	0.884	0	0
6067	3195433	STM3033	0.861	0.856	0	0
10666	3195682	STM3033, STM3034	0.997	0.887	0	0
17814	3195753	STM3033, STM3034	0.685	0.670	0	0
14106	3196042	STM3034, STM3035	0.945	0.709	0	0
6721	3196359	STM3036	0.927	0.757	0	0
9572	3196466	STM3036	0.998	0.735	0	0
2894	3196604	STM3036	1.007	0.799	0	0
3530	3197026	STM3036	0.700	1.871	0	1
15045	3197134	STM3036	0.654	2.018	0	1
18944	3197494	glyU	0.573	3.714	0	1
15061	3197749	glyU, STM3038	0.789	2.318	0	1
17865	3197904	glyU, STM3038	0.820	2.072	0	1
13849	3198015	STM3038	0.604	1.386	0	0
14915	3198322	STM3038, idi	0.948	1.908	0	0
16450	3198571	STM3038, idi	0.878	1.629	0	0
7195	3198685	STM3038, idi	0.921	1.490	0	0
538	3198982	idi	1.010	1.994	0	0
11635	3199095	idi	0.617	1.781	0	0
6705	3199556	lysS	0.732	1.159	0	0
3346	3199686	lysS	0.726	1.384	0	0
22097	3199927	lysS	0.880	1.645	0	0
13409	3200163	lysS	0.818	1.482	0	0
11706	3200459	lysS	0.927	1.882	0	0
16389	3200676	lysS	0.752	1.627	0	0
2503	3200902	lysS, prfB	0.716	1.458	0	0
14047	3201139	lysS, prfB	0.944	1.923	0	0
3911	3201259	prfB	0.565	1.565	0	0
7141	3201499	prfB	0.804	1.917	0	1
16835	3201732	prfB	1.070	2.715	0	1
15263	3201844	prfB	1.192	2.355	0	1
1592	3201936	prfB, recJ	1.068	2.384	0	1
9316	3202389	recJ	1.051	1.381	0	0
12817	3202738	recJ	0.610	0.851	0	0
12383	3202903	recJ	1.042	1.343	0	0
12266	3203121	recJ	1.057	1.336	0	0

8849	3203354	recJ	0.754	1.107	0	0
6489	3203655	recJ	0.762	1.051	0	0
13091	3203884	recJ, dsbC	0.834	1.150	0	0
8878	3203973	recJ, dsbC	0.819	1.137	0	0
22224	3204366	dsbC	0.921	1.350	0	0
6524	3204476	dsbC, xerD	0.954	1.382	0	0
12700	3204595	dsbC, xerD	0.885	1.339	0	0
4825	3204693	dsbC, xerD	0.944	1.427	0	0
19666	3205210	xerD, fldB	0.812	1.521	0	0
20286	3205401	xerD, fldB	0.706	1.179	0	0
15283	3205601	xerD, fldB	0.780	1.341	0	0
21215	3205793	fldB	0.844	2.222	0	1
4557	3205865	fldB	0.811	2.146	0	1
19070	3206254	ygfX	0.656	1.076	0	0
6364	3206514	ygfX, ygfY	1.115	1.065	0	0
20757	3206823	ygfX, ygfY	1.443	1.017	0	0
6266	3206901	ygfY, ygfZ	1.210	1.075	0	0
18887	3207185	ygfZ	1.185	1.210	0	0
15410	3207359	ygfZ	0.876	1.073	0	0
16861	3207759	ygfZ	0.946	1.675	0	0
18236	3207852	ygfZ	0.584	1.082	0	0
2569	3208203	yqfA	0.640	0.975	0	0
16275	3208379	yqfA	0.934	1.235	0	0
7517	3208644	yqfA	1.026	0.972	0	0
13672	3208875	yqfA, yqfB	0.995	0.768	0	0
9097	3209109	yqfA, yqfB	0.836	0.719	0	0
9380	3209224	yqfB, bglA	0.974	0.752	0	0
7276	3209514	yqfB, bglA	0.973	0.813	0	0
2719	3209745	bglA	0.945	0.603	0	0
1961	3209902	bglA	0.861	0.645	0	0
10992	3210052	bglA	0.862	0.629	0	0
12495	3210443	bglA	1.338	0.702	0	0
20101	3210682	bglA	2.438	0.669	1	0
875	3210918	STM3052	3.142	0.652	1	0
1663	3210997	STM3052	4.284	0.637	1	0
16928	3211329	STM3052	3.477	0.595	1	0

17396	3211417	STM3052	4.680	0.609	1	0
5939	3211567	STM3052	3.489	0.390	1	0
14764	3211968	STM3052	3.502	0.812	1	0
1170	3212107	STM3052, gcvP	2.988	1.149	1	0
12050	3212512	gcvP	1.287	1.669	0	1
14991	3212597	gcvP	1.125	1.683	0	1
4534	3212880	gcvP	0.946	2.215	0	1
13476	3213083	gcvP	0.658	1.809	0	1
4894	3213324	gcvP	0.754	1.829	0	0
21324	3213610	gcvP	0.822	2.171	0	1
17752	3213690	gcvP	0.710	2.000	0	1
760	3213917	gcvP	0.787	2.156	0	1
15630	3214279	gcvP	0.781	2.512	0	1
7226	3214456	gcvP	0.752	2.562	0	1
7052	3214662	gcvP	0.736	2.401	0	1
17557	3214846	gcvP	0.735	3.024	0	1
19789	3215002	gcvP	0.523	1.927	0	1
6833	3215219	gcvP, gcvH	0.661	1.678	0	0
19383	3215608	gcvH, gcvT	0.666	1.866	0	0
19104	3215800	gcvH, gcvT	0.453	1.249	0	0
10642	3215941	gcvH, gcvT	0.709	1.980	0	1
4462	3216335	gcvT	0.914	2.561	0	1
19727	3216484	gcvT	0.680	2.006	0	1
17740	3216838	gcvT	1.181	1.495	0	0
7035	3217011	gcvT	1.635	1.286	0	0
22017	3217297	visC	1.141	1.242	0	0
11523	3217524	visC	1.022	1.260	0	0
664	3217664	visC	0.961	1.366	0	0
2362	3217980	visC	1.104	1.310	0	0
9695	3218052	visC	1.134	1.300	0	0
7711	3218308	visC	0.952	1.517	0	0
1519	3218567	visC, ubiH	0.794	1.450	0	0
837	3218761	visC, ubiH	1.050	1.589	0	0
12258	3219061	ubiH	1.260	1.533	0	0
4816	3219172	ubiH	0.760	1.278	0	0
15284	3219535	ubiH	1.211	1.888	0	0

20218	3219702	ubiH, pepP	1.140	1.486	0	0
10108	3219916	ubiH, pepP	1.450	1.575	0	0
20659	3220146	pepP	1.122	1.335	0	0
18990	3220295	pepP	0.979	1.007	0	0
4131	3220577	pepP	0.425	0.669	0	0
3030	3220791	pepP	0.977	1.396	0	0
12366	3220934	pepP	0.696	1.106	0	0
10759	3221057	pepP, ygfB	0.757	1.184	0	0
3843	3221524	ygfB, ygfE	0.706	2.936	0	1
12452	3221837	ygfB, ygfE	0.503	3.085	0	1
10921	3222003	ygfB, ygfE	0.467	2.938	0	1
10655	3222196	ygfA	0.520	3.986	0	1
17185	3222296	ygfA	0.501	3.950	0	1
18373	3222589	ygfA	0.509	2.135	0	1
6604	3222880		0.585	1.959	0	1
15913	3223186		0.801	1.820	0	1
4656	3223352	serA	0.818	1.597	0	0
3443	3223551	serA	0.896	1.401	0	0
1172	3223642	serA	0.993	1.147	0	0
5826	3223943	serA	1.095	0.923	0	0
6283	3224297	serA	1.210	1.007	0	0
11164	3224479	serA	0.900	1.110	0	0
714	3224642	serA	1.395	1.329	0	0
21017	3224896	serA, rpiA	1.260	1.391	0	0
19447	3225211	rpiA, iciA	1.040	1.223	0	0
3694	3225454	rpiA, iciA	1.119	1.274	0	0
4749	3225560	rpiA, iciA	1.152	0.973	0	0
19270	3225702	rpiA, iciA	1.030	1.023	0	0
18209	3225934	iciA	0.922	0.793	0	0
1087	3226302	iciA	1.325	1.123	0	0
18915	3226526		1.155	1.218	0	0
10526	3226632		1.169	1.158	0	0
7299	3226913	yggE	1.164	1.063	0	0
10653	3227153	yggE	0.957	0.882	0	0
19393	3227475	yggE	0.944	0.941	0	0
11209	3227629	yggE, yggA	0.641	0.747	0	0

17233	3227813	yggE, yggA	0.906	0.699	0	0
7867	3228208	yggA	1.930	0.733	0	0
13517	3228434	yggA, yggB	2.446	0.883	0	0
6827	3228637	yggA, yggB	2.300	0.758	0	0
10498	3228734	yggB	2.319	0.819	0	0
9356	3229117	yggB	1.574	0.836	0	0
5051	3229260	yggB	1.473	0.888	0	0
20995	3229360	yggB	1.439	1.021	0	0
4547	3229646	yggB, fba	0.982	1.397	0	0
18521	3229825	fba	0.707	1.140	0	0
16804	3230114	fba	0.742	1.505	0	0
474	3230256	fba	0.560	1.218	0	0
3406	3230499	fba	0.604	1.337	0	0
1994	3230573	fba	0.694	1.366	0	0
18708	3231110	pgk	0.707	1.626	0	0
10680	3231219	pgk	0.678	1.470	0	0
6748	3231567	pgk	0.763	1.719	0	1
21895	3231819	pgk	0.626	1.722	0	1
20136	3232064	pgk, epd	0.650	1.857	0	1
3211	3232153	pgk, epd	0.687	1.851	0	1
2789	3232409	epd	0.809	1.741	0	0
20472	3232513	epd	0.853	2.172	0	1
13172	3232980	epd, STM3071	0.885	1.656	0	1
13176	3233141	epd, STM3071	1.011	1.387	0	0
1578	3233251	epd, STM3071	1.009	0.971	0	0
6642	3233358	epd, STM3071	1.101	0.981	0	0
9997	3233825	STM3072	1.231	0.661	0	0
1458	3233908	STM3072, STM3073	0.703	0.496	0	0
17934	3234296	STM3072, STM3073	1.133	0.779	0	0
20462	3234487	STM3073	0.652	0.509	0	0
8300	3234609	STM3073, STM3074	0.954	0.665	0	0
13167	3234710	STM3073, STM3074	0.541	0.480	0	0
14165	3235004	STM3073, STM3074	1.410	0.942	0	0
7959	3235282	STM3074, STM3075	1.383	0.743	0	0
1534	3235588	STM3074, STM3075	1.218	0.845	0	0
8097	3235659	STM3074, STM3075	1.463	0.840	0	0

13663	3235950	STM3075	1.371	0.855	0	0
15841	3236293	STM3075	0.776	0.865	0	0
9181	3236416	tktA	0.830	1.060	0	0
13259	3236746	tktA	0.860	1.895	0	1
10358	3236999	tktA	0.787	1.653	0	1
3514	3237140	tktA	0.792	1.572	0	0
4873	3237386	tktA	0.992	2.121	0	1
4317	3237604	tktA	0.770	1.799	0	1
840	3237797	tktA	0.780	1.922	0	1
13297	3237925	tktA	0.806	2.088	0	1
9808	3238363	tktA, yggG	0.991	1.968	0	1
1599	3238636	tktA, yggG	0.986	1.219	0	0
8783	3238786	tktA, yggG	1.135	1.170	0	0
7031	3238901	yggG	1.196	1.052	0	0
21459	3239189	yggG	1.045	1.569	0	0
17351	3239597	speB	0.848	1.050	0	0
15290	3239825	speB	0.710	0.653	0	0
8458	3239940	speB	1.137	0.926	0	0
17186	3240121	speB	0.940	0.978	0	0
11305	3240372	speB	1.145	1.142	0	0
1465	3240537	speB	1.122	1.229	0	0
10423	3240667	speB, STM3079	1.364	1.115	0	0
249	3240994	STM3079	1.392	0.878	0	0
9137	3241235	STM3079	2.817	0.631	1	0
5907	3241393	STM3079	2.955	0.582	1	0
14760	3241748	STM3079	2.982	0.519	1	0
11546	3241994	STM3079, STM3080	2.940	0.446	1	0
1686	3242114	STM3080	3.073	0.597	1	0
14698	3242443	STM3080, STM3081	3.023	0.582	1	0
21691	3242541	STM3080, STM3081	2.772	0.674	1	0
8437	3242662	STM3080, STM3081	1.176	0.463	0	0
10831	3243070	STM3081	1.554	0.659	0	0
1057	3243405	STM3081, STM3082	1.608	0.678	0	0
6102	3243512	STM3081, STM3082	1.567	0.701	0	0
9046	3243678	STM3081, STM3082	1.476	0.641	0	0
16016	3243955	STM3082	1.610	0.693	0	0

21465	3244339	STM3082, STM3083	1.979	0.617	0	0
1728	3244506	STM3082, STM3083	1.541	0.694	0	0
15743	3244729	STM3082, STM3083	1.337	0.638	0	0
7405	3244923	STM3083	1.105	0.597	0	0
3369	3245219	STM3083	0.836	0.541	0	0
2444	3245439	STM3083	0.668	0.575	0	0
10862	3245684	STM3083	0.557	0.519	0	0
15335	3245845	STM3083	0.482	0.473	0	0
17999	3246136	STM3083	0.471	0.570	0	0
14943	3246291		0.480	0.593	0	0
20164	3246428		0.524	0.682	0	0
21881	3246579		0.567	0.765	0	0
17904	3246864		0.433	0.836	0	0
16448	3247141	STM3084	0.713	0.981	0	0
16875	3247220	STM3084	0.668	0.859	0	0
13704	3247647	STM3084	0.672	0.863	0	0
7400	3247843	STM3084	0.995	0.918	0	0
14477	3248109	STM3084, STM3085	1.848	0.801	0	0
5819	3248419	STM3085	3.135	0.553	1	0
6482	3248571	STM3085	3.656	0.548	1	0
9568	3248904	STM3085, speA	3.484	0.689	1	0
19348	3249033	STM3085, speA	2.981	0.783	1	0
9725	3249274	speA	1.987	0.943	0	0
21027	3249592	speA	0.735	0.715	0	0
523	3249743	speA	1.204	1.276	0	0
968	3249881	speA	1.295	1.386	0	0
16258	3250054	speA	1.241	1.192	0	0
9574	3250286	speA	1.044	1.264	0	0
3063	3250362	speA	1.142	1.190	0	0
21708	3250872	speA, yqgC	0.651	1.975	0	1
21457	3251022	speA, yqgB	0.718	2.013	0	1
19076	3251238	speA, yqgB	0.696	2.505	0	1
6751	3251569	yqgD, metK	0.701	1.166	0	0
19166	3251690	yqgD, metK	0.772	1.011	0	0
11302	3251881	yqgD, metK	0.902	1.030	0	0
2507	3252265	metK	1.037	0.936	0	0

20434	3252396	metK	0.915	0.908	0	0
15291	3252621	metK	0.902	0.944	0	0
1750	3252939	galP	1.069	0.866	0	0
2974	3253010	galP	1.134	0.733	0	0
5273	3253343	galP	1.217	0.772	0	0
5717	3253451	galP	1.014	0.786	0	0
2467	3253757	galP	0.891	0.899	0	0
6596	3253994	galP	1.233	1.047	0	0
338	3254096	galP	1.164	1.235	0	0
5640	3254545	galP, sprT	0.903	1.235	0	0
15142	3254649	galP, sprT	1.092	1.438	0	0
13949	3254880	sprT	1.152	1.771	0	0
11182	3255182	sprT, endA	0.656	1.067	0	0
601	3255363	endA	1.024	1.133	0	0
15575	3255569	endA	1.083	0.905	0	0
17038	3255709	endA, yggJ	1.424	1.144	0	0
8304	3255948	endA, yggJ	1.174	1.070	0	0
17808	3256333	yggJ	1.272	1.446	0	0
20681	3256466	yggJ, gshB	0.995	1.270	0	0
288	3256810	yggJ, gshB	1.204	1.513	0	0
15677	3257020	gshB	1.112	1.819	0	1
20075	3257205	gshB	1.291	2.577	0	1
18169	3257373	gshB	1.106	2.218	0	1
10945	3257729	gshB, yqgE	1.366	1.684	0	1
17051	3257804	gshB, yqgE	1.475	1.935	0	1
11071	3258198	yqgE, yqgF	1.542	1.489	0	0
6740	3258388	yqgE, yqgF	1.147	1.236	0	0
11918	3258487	yqgE, yqgF	1.267	1.165	0	0
4892	3258784	yqgF	1.177	1.322	0	0
11725	3259040	yqgF	1.352	1.527	0	0
10361	3259321	STM3098	1.316	1.846	0	0
18312	3259442	STM3098	0.760	0.890	0	0
17894	3259786	STM3098	1.314	1.218	0	0
20709	3259872	STM3098	0.957	1.030	0	0
21305	3260038	STM3098, yggR	1.036	0.783	0	0
17524	3260434	yggR	1.252	0.747	0	0

10813	3260539	yggR, yggS	1.013	0.766	0	0
2122	3260857	yggR, yggS	1.145	0.940	0	0
3772	3260928	yggR, yggS	1.068	0.883	0	0
6158	3261307	yggR, yggS	1.169	1.009	0	0
21148	3261609	yggS, yggT	1.350	1.246	0	0
16242	3261753	yggT, yggU	1.297	1.403	0	0
18751	3261870	yggT, yggU	1.360	1.420	0	0
314	3262006	yggT, yggU	1.081	1.244	0	0
19324	3262379	yggU, yggV	1.141	1.557	0	0
21214	3262496	yggV	1.014	1.356	0	0
10939	3262876	yggV, yggW	1.044	1.528	0	0
9389	3263144	yggW	0.977	1.038	0	0
9550	3263243	yggW	1.158	1.303	0	0
6534	3263556	yggW	0.826	1.163	0	0
7889	3263739	yggW	0.813	0.900	0	0
21058	3264012	yggW	1.207	1.189	0	0
8640	3264330	yggM	1.104	0.926	0	0
1104	3264401	yggM	1.030	0.907	0	0
4883	3264639	yggM	1.012	0.690	0	0
12264	3264783	yggM	1.009	0.738	0	0
11593	3265227	yggM	0.793	0.857	0	0
9153	3265355	yggM	0.717	1.034	0	0
10979	3265470	yggM, ansB	0.720	1.204	0	0
582	3265862	ansB	0.802	1.183	0	0
7933	3266138	ansB	0.943	1.372	0	0
4912	3266349	ansB	1.003	1.137	0	0
21622	3266481	ansB	1.267	1.003	0	0
8660	3266797	ansB, yggN	1.290	1.064	0	0
7552	3266996	yggN	0.952	0.920	0	0
15643	3267069	yggN	0.935	1.080	0	0
8293	3267475	yggN, yggL	0.963	1.065	0	0
20463	3267672	yggN, yggL	1.034	1.113	0	0
15466	3267800	yggN, yggL	0.801	0.899	0	0
11597	3268107	yggL, yggH	0.929	1.058	0	0
16177	3268206	yggL, yggH	0.657	0.938	0	0
9860	3268665	yggH, mutY	0.998	1.184	0	0

22329	3268792	yggH, mutY	1.082	1.151	0	0
12892	3269057	mutY	1.083	1.015	0	0
16924	3269359	mutY, yggX	1.101	1.161	0	0
2276	3269530	mutY, yggX	0.755	0.884	0	0
20048	3269740	yggX, mltC	1.105	1.274	0	0
6012	3269816	yggX, mltC	0.952	1.010	0	0
2498	3270221	mltC	1.151	1.398	0	0
8818	3270341	mltC	1.025	1.173	0	0
13743	3270523	mltC	1.071	1.087	0	0
18721	3270768	mltC	1.184	1.161	0	0
15403	3271072	mltC, nupG	1.126	1.197	0	0
20060	3271379	nupG	0.773	0.930	0	0
5535	3271457	nupG	0.556	0.648	0	0
12499	3271786	nupG	1.109	1.174	0	0
5148	3272055	nupG	1.008	0.953	0	0
20470	3272127	nupG	0.918	0.943	0	0
10585	3272270	nupG	0.924	0.912	0	0
5332	3272539	nupG	1.154	1.176	0	0
4914	3272775	speC	1.091	1.000	0	0
21873	3273089	speC	1.339	0.984	0	0
4584	3273408	speC	1.173	0.920	0	0
2770	3273579	speC	1.219	0.862	0	0
15315	3273861	speC	1.163	0.997	0	0
359	3274124	speC	1.314	1.062	0	0
9937	3274368	speC	1.308	0.864	0	0
6176	3274442	speC	1.237	0.857	0	0
19782	3274548	speC	1.064	0.826	0	0
8803	3274893	speC, yqgA	1.226	1.188	0	0
13614	3275000	speC, yqgA	0.720	0.775	0	0
9136	3275287	speC, yqgA	1.414	0.804	0	0
19266	3275427	yqgA	1.039	0.684	0	0
2031	3275733	yqgA, pheV	0.900	1.172	0	0
332	3276076	pheV	0.610	2.925	0	1
807	3276235		0.696	2.387	0	1
7328	3276397	STM3117	0.872	1.985	0	1
5734	3276472	STM3117	1.018	1.279	0	0

2817	3277051	STM3117, STM3118	1.515	0.679	0	0
11832	3277156	STM3117, STM3118	1.604	0.515	0	0
21746	3277495	STM3118	2.596	0.572	0	0
4495	3277566	STM3118	2.356	0.510	0	0
7285	3277674	STM3118	2.972	0.603	0	0
832	3278217	STM3118, STM3119	1.471	0.643	0	0
19079	3278436	STM3118, STM3119	1.312	0.622	0	0
10859	3278593	STM3119	1.495	0.689	0	0
12233	3278820	STM3119, STM3120	1.391	0.684	0	0
2359	3278955	STM3119, STM3120	1.844	0.780	0	0
14125	3279168	STM3120, STM3121	1.749	0.709	0	0
1664	3279585	STM3120, STM3121	1.968	0.653	0	0
10748	3279821	STM3120, STM3121	1.639	0.840	0	0
6244	3279916	STM3120, STM3121	1.008	0.697	0	0
7005	3280206	STM3121	1.078	0.727	0	0
16082	3280372	STM3121	0.836	0.731	0	0
19503	3280506	STM3121	0.954	0.644	0	0
6424	3280639	STM3122	1.006	0.690	0	0
6654	3281100	STM3122	1.327	0.764	0	0
2013	3281261	STM3122	1.402	0.624	0	0
8915	3281578	STM3122	1.040	0.669	0	0
7783	3281764	STM3122	1.171	0.898	0	0
4334	3282007	STM3122	0.879	1.048	0	0
12633	3282188	STM3122	1.495	0.861	0	0
9924	3282292	STM3122	1.423	0.824	0	0
2158	3282658	STM3122, STM3123	1.333	0.760	0	0
12784	3282780	STM3123	1.215	0.667	0	0
10772	3283010	STM3123	1.483	0.545	0	0
8139	3283118	STM3123	1.966	0.679	0	0
2546	3283565	STM3123	1.936	0.543	0	0
8340	3283707	STM3123, STM3124	1.893	0.529	0	0
16201	3283993	STM3123, STM3124	1.597	0.618	0	0
2506	3284235	STM3124	1.020	0.557	0	0
17688	3284341	STM3124	1.385	0.756	0	0
17993	3284804		1.693	0.673	0	0
15073	3284898	STM3125	1.748	0.716	0	0

4518	3285067	STM3125	1.547	0.732	0	0
16690	3285241	STM3125	1.513	0.767	0	0
10715	3285561	STM3125, STM3126	1.538	0.781	0	0
3762	3285633	STM3125, STM3126	1.575	0.714	0	0
13720	3285800	STM3125, STM3126	1.707	0.756	0	0
6107	3286375	STM3126	1.132	0.674	0	0
8571	3286494	STM3126	1.094	0.668	0	0
1353	3286734	STM3126	1.663	0.755	0	0
10541	3286940	STM3126	1.902	0.603	0	0
10567	3287090	STM3126, STM3127	1.489	0.622	0	0
11598	3287446	STM3127	1.375	0.701	0	0
11002	3287705	STM3127, STM3128	1.494	0.746	0	0
11095	3287792	STM3127, STM3128	1.762	0.896	0	0
15851	3288142	STM3128	1.009	0.704	0	0
11069	3288417	STM3128	1.764	0.690	0	0
12546	3288516	STM3128, STM3129	1.637	0.795	0	0
11222	3288854	STM3128, STM3129	1.363	0.623	0	0
16051	3288981	STM3128, STM3129	1.151	0.636	0	0
546	3289243	STM3128, STM3129	1.202	0.693	0	0
14181	3289454	STM3129	1.254	0.902	0	0
6075	3289815	STM3129	1.378	0.827	0	0
4634	3289888	STM3129	1.370	0.786	0	0
15282	3290152	STM3129	0.861	0.575	0	0
13736	3290245	STM3129	1.149	0.685	0	0
8115	3290448		1.013	0.634	0	0
15304	3290677	STM3130	1.092	0.653	0	0
4507	3290869	STM3130	0.992	0.570	0	0
15933	3291321	STM3130, STM3131	2.183	0.630	0	0
9093	3291435	STM3131	1.838	0.556	0	0
710	3291675	STM3131	3.247	0.546	1	0
10667	3292055		3.078	0.521	1	0
8046	3292244	STM3132	1.864	0.609	0	0
12765	3292370	STM3132	1.898	0.612	0	0
14952	3292667	STM3132	2.292	0.588	1	0
2286	3292862	STM3132	2.674	0.566	1	0
8224	3293025	STM3132	3.096	0.615	1	0

15229	3293219	STM3132, STM3133	3.212	0.543	1	0
16793	3293459	STM3132, STM3133	3.279	0.686	1	0
7362	3293789	STM3133	3.383	0.510	1	0
1022	3294066	STM3133	4.172	0.525	1	0
19974	3294196	STM3133	3.193	0.537	1	0
2064	3294506	STM3134	2.738	0.687	1	0
1634	3294726	STM3134	1.672	0.672	0	0
22093	3294919	STM3134	1.455	0.709	0	0
7823	3295103	STM3134	1.394	0.677	0	0
9639	3295459	STM3134	1.169	0.751	0	0
10456	3295671	STM3134, STM3135	0.684	0.540	0	0
10947	3295754	STM3134, STM3135	1.391	0.671	0	0
5856	3296080	STM3134, STM3135	1.530	0.777	0	0
12350	3296226	STM3135	1.135	0.770	0	0
7162	3296466	STM3135	1.142	0.826	0	0
12768	3296734	STM3135	1.133	0.901	0	0
4879	3296864	STM3135	1.578	0.898	0	0
6262	3297082	STM3135, STM3136	1.256	0.635	0	0
9868	3297181	STM3135, STM3136	1.301	0.934	0	0
20788	3297693	STM3136	1.574	0.948	0	0
5522	3297863	STM3136	0.536	0.612	0	0
14541	3298120	STM3136	1.265	0.923	0	0
10701	3298324	STM3136	1.209	0.856	0	0
647	3298670	STM3136, STM3137	1.160	0.996	0	0
2582	3298895	STM3137	1.557	1.060	0	0
17801	3299136	STM3137	1.657	0.949	0	0
17317	3299248	STM3137	1.549	0.979	0	0
20637	3299388	STM3137	1.146	1.030	0	0
15375	3299467	STM3137	1.686	1.256	0	0
1621	3299907	STM3137	1.604	1.073	0	0
15489	3300159	STM3137	2.201	1.027	0	0
15377	3300251	STM3137	2.196	0.866	0	0
10852	3300563		2.455	0.836	0	0
17454	3300835	STM3138	2.381	0.700	0	0
7495	3301132	STM3138	2.749	0.669	1	0
15222	3301361	STM3138	2.694	0.651	1	0

9648	3301505	STM3138	2.874	0.709	1	0
15143	3301777	STM3138	3.335	0.643	1	0
8658	3302067	STM3138	3.435	0.614	1	0
19702	3302240		2.656	0.691	1	0
15611	3302350	gsp	2.444	0.792	1	0
14178	3302536	gsp	1.928	0.939	0	0
21440	3302641	gsp	1.699	0.913	0	0
15219	3303009	gsp	1.540	0.916	0	0
3345	3303126	gsp	0.815	0.629	0	0
4868	3303610	gsp	0.840	0.845	0	0
17839	3303752	gsp	1.135	0.989	0	0
2596	3303952	gsp, yghU	1.029	0.891	0	0
18813	3304289	gsp, yghU	1.063	0.792	0	0
4294	3304418	gsp, yghU	1.294	0.973	0	0
21755	3304551	gsp, yghU	1.581	1.070	0	0
14329	3304936	yghU	1.335	1.012	0	0
18461	3305089	yghU	1.150	0.936	0	0
6122	3305251	yghU	1.502	1.088	0	0
5825	3305559	STM3141	1.240	0.692	0	0
882	3305708	STM3141	1.573	1.086	0	0
15880	3306113	STM3141, STM3142	1.526	0.863	0	0
10905	3306301	STM3141, STM3142	1.468	0.760	0	0
4987	3306574	STM3142	1.465	0.849	0	0
3457	3306741	STM3142	1.290	0.786	0	0
19248	3306922	STM3142	1.561	0.711	0	0
5605	3307264	STM3142, hybG	1.690	0.943	0	0
10615	3307456	STM3142, hybG	1.651	1.247	0	0
13046	3307759	hybG, hybF	0.539	0.594	0	0
20464	3307968	hybF, hybE	1.206	0.896	0	0
1109	3308142	hybF, hybE	1.144	0.839	0	0
16072	3308403	hybE, hybD	0.818	0.692	0	0
6964	3308605	hybE, hybD	1.169	1.042	0	0
9993	3308754	hybD	1.037	0.884	0	0
10619	3308994	hybD, hybC	1.122	0.911	0	0
21762	3309122	hybD, hybC	1.289	1.006	0	0
2630	3309508	hybC	1.177	1.402	0	0

15513	3309700	hybC	0.977	1.990	0	1
6846	3309774	hybC	0.918	2.123	0	1
2300	3310165	hybC	1.047	0.959	0	0
8963	3310433	hybC	1.022	0.934	0	0
18786	3310563	hybC, hybB	1.176	0.947	0	0
8241	3310762	hybC, hybB	1.211	0.844	0	0
19937	3311143	hybB	0.989	0.724	0	0
8699	3311269	hybB	1.236	0.692	0	0
1877	3311556	hybB	1.032	0.611	0	0
12972	3311645	hybB, hybA	0.952	0.769	0	0
8176	3311876	hybB, hybA	1.046	0.607	0	0
7650	3312297	hybA	1.114	0.710	0	0
16449	3312371	hybA	1.139	0.708	0	0
7792	3312620	hybA, hypO	1.274	0.908	0	0
16546	3312821	hybA, hypO	1.409	0.876	0	0
9165	3313181	hypO	1.153	1.085	0	0
6723	3313421	hypO	0.952	1.169	0	0
13596	3313504	hypO	0.723	0.808	0	0
18253	3313770	hypO	1.251	1.047	0	0
18849	3313996	hypO, yghW	1.457	0.975	0	0
5796	3314096	hypO, yghW	1.652	1.074	0	0
6371	3314200	hypO, yghW	1.132	0.785	0	0
4486	3314558	yghW, STM3152	1.251	0.826	0	0
5323	3314916	STM3152	1.280	0.877	0	0
14789	3315101	STM3152	1.444	1.052	0	0
19482	3315319	STM3152	1.270	0.930	0	0
11358	3315390	STM3152	1.138	0.993	0	0
11352	3315889	STM3152	1.856	0.868	0	0
12121	3316077	STM3152	1.930	0.969	0	0
4941	3316321	STM3152, yqhA	1.457	1.065	0	0
19889	3316503	yqhA	1.250	1.349	0	0
14097	3316787	yqhA	0.984	1.197	0	0
11035	3317024	yqhA, STM3154	1.650	1.076	0	0
22252	3317277	STM3154, STM3155	2.430	0.972	1	0
10173	3317445	STM3154, STM3155	3.048	0.972	1	0
811	3317710	STM3154, STM3155	2.940	1.032	1	0

16403	3317970	STM3154, STM3155	2.431	0.842	1	0
19026	3318107	STM3156, yghA	2.053	0.745	0	0
13778	3318440	yghA	1.401	0.949	0	0
5398	3318519	yghA	1.596	0.934	0	0
6609	3318672	yghA	0.651	0.636	0	0
4414	3318989	yghA	1.186	1.065	0	0
5715	3319116	yghA	0.949	0.787	0	0
20656	3319564	exbD	0.967	0.979	0	0
3745	3319638	exbD	1.009	1.056	0	0
6697	3319763	exbD	1.046	1.069	0	0
3916	3320114	exbD, exbB	1.183	1.086	0	0
18444	3320410	exbD, exbB	0.989	0.946	0	0
4884	3320597	exbB, metC	0.994	0.860	0	0
13763	3320720	exbB, STM3160	1.299	0.949	0	0
10048	3320976	exbB, STM3160	1.490	0.749	0	0
16257	3321300	STM3160, metC	0.720	0.682	0	0
11780	3321449	metC	1.318	0.759	0	0
2891	3321594	metC	1.088	0.775	0	0
21011	3321958	metC, yghB	1.256	1.062	0	0
10783	3322095	metC, yghB	0.765	0.836	0	0
17936	3322267	yghB	1.047	1.542	0	0
6948	3322381	yghB	1.075	1.656	0	0
19603	3322850	yghB	0.659	1.128	0	0
3688	3322945	yghB	0.982	1.751	0	0
2344	3323304	yqhC	1.115	1.242	0	0
13582	3323678	yqhC, yqhD	1.135	0.800	0	0
8391	3323755	yqhC, yqhD	2.175	1.070	0	0
19244	3324019	yqhC, yqhD	1.177	0.673	0	0
15019	3324099	yqhC, yqhD	1.107	0.597	0	0
9801	3324294	yqhC, yqhD	1.324	0.784	0	0
20600	3324765	yqhD	1.200	0.762	0	0
7487	3324838	yqhD	1.022	0.779	0	0
14290	3325014	yqhD, yqhE	1.071	0.702	0	0
624	3325383	yqhE	0.970	0.806	0	0
15474	3325678	yqhE	0.852	0.836	0	0
3654	3325799	yqhE	0.764	0.840	0	0

12149	3326136	yqhE, STM3166	0.956	0.695	0	0
7135	3326280	STM3166	0.815	0.664	0	0
14883	3326405	STM3166	0.659	0.705	0	0
7361	3326778	STM3166	0.797	0.624	0	0
14596	3326950	STM3166	1.035	0.595	0	0
14423	3327137	STM3166	0.466	0.334	0	0
3265	3327448	STM3166, STM3167	1.274	0.569	0	0
21404	3327528	STM3166, STM3167	1.402	0.582	0	0
14350	3327905	STM3167	2.279	0.710	1	0
4358	3328001	STM3167	2.246	0.665	1	0
12604	3328291	STM3167	3.170	0.726	1	0
9969	3328611		5.754	0.599	1	0
6469	3328888		4.956	0.642	1	0
16088	3329075	ygiR	0.437	0.475	0	0
13101	3329208	ygiR	0.589	0.631	0	0
18386	3329438	ygiR	1.765	0.825	0	0
19671	3329673	ygiR	1.418	0.735	0	0
11799	3329861	ygiR	1.101	0.891	0	0
17242	3330188	ygiR	0.777	0.678	0	0
15765	3330419	ygiR	0.896	0.901	0	0
8961	3330531	ygiR	0.727	0.728	0	0
11544	3330803	ygiR	1.006	1.005	0	0
12788	3331091	ygiR	1.110	0.902	0	0
11315	3331378	ygiR, STM3169	0.793	0.498	0	0
18273	3331563	STM3169	0.901	0.613	0	0
4935	3331683	STM3169	0.913	0.566	0	0
6657	3332046	STM3169	1.158	0.682	0	0
8501	3332299	STM3169, STM3170	1.501	0.571	0	0
6113	3332527	STM3169, STM3170	2.197	0.602	0	0
12523	3332737	STM3170, ygiK	1.414	0.596	0	0
20945	3332975	STM3170, ygiK	4.381	0.876	1	0
16208	3333090	ygiK	4.508	0.895	1	0
22306	3333183	ygiK	3.517	0.923	1	0
3764	3333497	ygiK	1.855	0.703	0	0
11743	3333896	ygiK	2.936	0.857	1	0
22170	3334057	ygiK	3.348	0.852	1	0

20185	3334297	ygiK	2.767	1.066	1	0
13224	3334572	sufI	1.329	1.143	0	0
17497	3334738	sufI	1.426	1.467	0	0
14195	3334919	sufI	1.545	1.506	0	0
11941	3335063	sufI	1.403	1.458	0	0
2691	3335297	sufI	1.403	1.201	0	0
18362	3335369	sufI	0.873	1.258	0	0
775	3335837	sufI	1.125	1.212	0	0
10011	3336096	sufI, plsC	1.159	1.174	0	0
4848	3336246	sufI, plsC	1.233	1.130	0	0
5955	3336330	plsC	1.226	1.147	0	0
14131	3336716	plsC	1.024	1.088	0	0
9250	3336790	plsC	1.302	1.215	0	0
10983	3337177	parC	0.860	1.179	0	0
22197	3337337	parC	1.085	1.279	0	0
6174	3337561	parC	0.878	1.187	0	0
20838	3337807	parC	1.041	1.073	0	0
1604	3338209	parC	0.557	0.673	0	0
5917	3338327	parC	1.103	1.223	0	0
4531	3338505	parC	1.146	1.290	0	0
7851	3338796	parC	0.964	1.197	0	0
17086	3339123	parC	0.986	2.790	0	1
6336	3339194	parC	0.909	3.394	0	1
2875	3339279	parC	0.978	2.674	0	1
1525	3339757	STM3175	0.507	0.819	0	0
451	3339863	STM3175	1.332	1.421	0	0
11497	3340224	STM3175, ygiW	1.041	1.249	0	0
15418	3340375	STM3175, ygiW	1.194	1.258	0	0
8880	3340655	ygiW, qseB	1.368	1.035	0	0
585	3340783	ygiW, qseB	1.227	0.914	0	0
467	3340854	ygiW, qseB	1.205	0.982	0	0
11150	3341328	qseB, qseC	1.242	0.785	0	0
16689	3341547	qseC	1.066	0.844	0	0
12506	3341800	qseC	1.220	0.868	0	0
17956	3341967	qseC	1.082	0.812	0	0
11502	3342095	qseC	1.027	0.747	0	0

19725	3342317	qseC	1.324	0.889	0	0
18395	3342498	qseC, mdaB	0.938	0.628	0	0
15731	3342877	mdaB	1.039	0.825	0	0
19441	3343130	mdaB, ygiN	1.126	1.082	0	0
9596	3343445	mdaB, ygiN	1.079	1.320	0	0
5774	3343571	ygiN	0.882	1.398	0	0
18911	3343762	ygiN	0.671	1.372	0	0
10795	3344033	parE	0.781	1.824	0	1
6479	3344220	parE	0.902	1.710	0	1
21281	3344477	parE	0.555	0.975	0	0
12101	3344801	parE	0.493	0.905	0	0
2971	3344882	parE	0.974	1.518	0	0
4400	3345229	parE	0.301	0.577	0	0
2963	3345398	parE	0.909	1.258	0	0
20260	3345537	parE	0.875	1.351	0	0
15320	3345878	parE, yqiA	0.935	2.152	0	1
8814	3346060	parE, yqiA	1.152	2.476	0	1
8456	3346211	parE, yqiA	0.744	1.556	0	0
4771	3346419	yqiA	0.911	1.752	0	0
16930	3346647	yqiA, icc	0.901	2.051	0	0
4856	3346886	yqiA, icc	0.483	1.069	0	0
13023	3347263	icc	0.733	1.706	0	1
11068	3347339	icc, yqiB	0.751	1.824	0	1
20438	3347698	icc, yqiB	0.685	1.040	0	0
1580	3347850	yqiB, yqiE	0.743	0.985	0	0
10731	3348217	yqiE, tolC	0.982	1.206	0	0
8007	3348314	yqiE, tolC	0.881	1.020	0	0
9219	3348540	yqiE, tolC	0.784	1.135	0	0
15934	3348775	yqiE, tolC	0.835	1.178	0	0
21928	3349022	tolC	0.839	1.271	0	0
18590	3349223	tolC	0.881	1.443	0	0
3841	3349401	tolC	0.853	1.392	0	0
22293	3349499	tolC	0.919	1.399	0	0
7536	3349794	tolC, ygiB	0.931	1.300	0	0
18737	3350177	ygiB	0.852	1.929	0	0
15537	3350357	ygiB	0.603	1.023	0	0

316	3350440	ygiB, ygiC	1.041	1.588	0	0
2141	3350896	ygiB, ygiC	1.320	1.069	0	0
6328	3350979	ygiC	1.108	1.201	0	0
7404	3351137	ygiC	0.894	1.195	0	0
16213	3351573	ygiC	1.019	1.383	0	0
19059	3351788	ygiC	1.102	1.512	0	0
3116	3352041	ygiC	0.882	1.455	0	0
7606	3352172	ygiD	0.831	1.334	0	0
13230	3352394	ygiD	1.147	1.218	0	0
465	3352535	ygiD	0.938	1.121	0	0
19936	3352909	ygiD, ygiE	1.092	0.653	0	0
9037	3353003	ygiD, ygiE	1.205	0.697	0	0
3535	3353396	ygiD, ygiE	1.331	0.936	0	0
12995	3353641	ygiE	0.914	0.690	0	0
13072	3353804	ygiE	2.488	0.757	1	0
13445	3354013	STM3191	4.040	0.510	1	0
321	3354225	STM3191	1.777	0.451	0	0
17475	3354474	STM3191	3.549	0.472	1	0
1450	3354750	STM3191	5.144	0.617	1	0
21578	3354885	STM3191	4.608	0.567	1	0
13180	3355152	STM3191, STM3192	2.578	0.557	1	0
6033	3355386	STM3191, STM3192	1.661	0.565	0	0
3692	3355663	STM3192	1.300	0.634	0	0
17764	3355831	STM3192, STM3193	2.032	0.551	0	0
18850	3356149	STM3192, STM3193	4.035	0.608	1	0
8566	3356245	STM3192, STM3193	3.826	0.605	1	0
6835	3356522	STM3193, STM3194	3.932	0.568	1	0
10817	3356635	STM3193, STM3194	3.337	0.624	1	0
16627	3356908	STM3193, STM3194	2.011	0.637	0	0
15476	3357296	STM3194	1.432	0.863	0	0
12154	3357500	STM3194	0.848	0.944	0	0
3437	3357657		0.988	1.183	0	0
20344	3357810		0.726	1.208	0	0
18487	3357886	ribB	0.698	1.316	0	0
11406	3358449	ribB, yqiC	0.491	2.476	0	1
17486	3358523	ribB, yqiC	0.519	2.823	0	1

16637	3358819	ribB, yqiC	0.613	2.398	0	1
13891	3358890	ribB, yqiC	0.651	2.028	0	1
5253	3359280		0.745	0.961	0	0
17076	3359533	glgS, STM3198	1.352	0.913	0	0
5799	3359671	glgS, STM3198	1.781	0.972	0	0
6861	3359759	glgS, STM3198	1.230	0.750	0	0
11763	3360089	STM3198, yqiK	1.212	0.675	0	0
18131	3360367	STM3198, yqiK	1.262	0.538	0	0
2696	3360600	yqiK	1.272	0.736	0	0
19642	3360857	yqiK	1.076	0.703	0	0
21969	3360955	yqiK	1.145	0.763	0	0
22005	3361145	yqiK	1.049	0.783	0	0
9588	3361358	yqiK	0.985	0.740	0	0
7119	3361738	yqiK	0.909	0.834	0	0
11798	3362041	yqiK	1.295	1.909	0	1
10778	3362130		0.863	1.927	0	1
11064	3362415		0.833	2.375	0	1
11762	3362554	rfaE	0.579	1.211	0	0
14045	3362717	rfaE	1.003	1.942	0	0
2438	3362913	rfaE	0.835	1.073	0	0
12016	3363321	rfaE	1.113	1.620	0	0
21799	3363608	rfaE	1.280	1.494	0	0
1326	3363884	rfaE	1.228	1.918	0	0
13684	3363960	rfaE	1.101	1.431	0	0
21856	3364322	rfaE, glnE	1.421	1.288	0	0
6722	3364512	glnE	1.084	1.079	0	0
17729	3364730	glnE	1.145	1.035	0	0
13826	3364931	glnE	1.014	0.983	0	0
9305	3365002	glnE	0.901	0.910	0	0
17826	3365388	glnE	1.447	1.337	0	0
21131	3365532	glnE	1.491	1.251	0	0
1810	3365709	glnE	1.565	1.119	0	0
13346	3365893	glnE	1.235	1.036	0	0
9751	3365965	glnE	1.581	1.196	0	0
4433	3366325	glnE	1.428	1.120	0	0
2644	3366768	glnE	1.026	0.903	0	0

9392	3366848	glnE	1.115	0.880	0	0
3300	3367218	glnE, ygiF	1.371	1.003	0	0
10242	3367493	ygiF	0.852	0.937	0	0
20174	3367570	ygiF	1.183	1.138	0	0
14628	3367882	ygiF	0.425	0.554	0	0
19106	3368091	ygiF, ygiM	0.929	0.923	0	0
5777	3368283	ygiF, ygiM	0.952	0.864	0	0
10959	3368474	ygiF, ygiM	0.859	0.889	0	0
9633	3368727	ygiF, ygiM	0.851	0.966	0	0
7074	3368943	ygiM, cca	1.111	1.352	0	0
5038	3369187	cca	0.802	1.208	0	0
14583	3369333	cca	0.834	1.231	0	0
2085	3369650	cca	1.169	1.423	0	0
5478	3369891	cca	0.918	1.040	0	0
20328	3370105	cca	1.010	1.226	0	0
10504	3370360	cca	1.092	1.633	0	0
10875	3370597	bacA	1.155	1.424	0	0
17527	3370708	bacA	1.208	1.305	0	0
17216	3371013	bacA	1.495	1.190	0	0
13642	3371379	bacA	1.969	1.105	0	0
7030	3371546	bacA, folB	1.727	1.066	0	0
19739	3371761	bacA, folB	1.304	0.972	0	0
3504	3371905	folB, ygiH	1.311	1.009	0	0
17156	3372220	folB, ygiH	0.950	1.281	0	0
6913	3372413	ygiH	0.834	1.693	0	1
15821	3372507	ygiH	1.032	1.905	0	1
5055	3372723		0.513	1.102	0	0
13033	3373150	gcp	0.963	1.043	0	0
17981	3373443	gcp	0.788	1.665	0	1
11541	3373647	gcp, rpsU	0.614	2.296	0	1
8639	3373789	gcp, rpsU	0.588	2.971	0	1
12923	3373878	gcp, rpsU	0.487	2.754	0	1
21373	3374030	gcp, rpsU	0.529	3.573	0	1
20376	3374586	dnaG	0.600	1.942	0	1
21960	3374717	dnaG	0.681	1.602	0	0
2387	3374838	dnaG	0.670	1.482	0	0

14360	3375070	dnaG	0.422	0.713	0	0
18555	3375285	dnaG	0.685	0.900	0	0
18442	3375470	dnaG	0.880	1.280	0	0
11855	3375746	dnaG	0.799	1.413	0	0
7133	3375957	dnaG, rpoD	0.765	1.582	0	0
3451	3376354	rpoD	0.657	1.520	0	0
14498	3376512	rpoD	0.760	1.370	0	0
18602	3376832	rpoD	0.658	1.740	0	1
10082	3376946	rpoD	0.865	1.867	0	1
2033	3377164	rpoD	0.705	1.617	0	0
7967	3377255	rpoD	0.791	1.699	0	0
7363	3377756	rpoD	0.790	1.463	0	0
10080	3377875	rpoD	0.887	1.376	0	0
9748	3378125	rpoD	0.865	1.420	0	0
19667	3378383	mug	0.830	1.377	0	0
8716	3378588	mug, ileX	0.723	1.304	0	0
1137	3378858	mug, ileX	0.599	2.408	0	1
12155	3378936	mug, ileX	0.547	2.176	0	1
21978	3379186	mug, yqjH	0.547	2.157	0	1
5938	3379332	yqjH	0.637	1.653	0	1
14136	3379739	yqjH, yqjI	1.007	0.806	0	0
12651	3379895	yqjH, yqjI	0.871	0.633	0	0
5910	3380001	yqjH, yqjI	0.916	0.563	0	0
10203	3380140	yqjH, yqjI	1.020	0.659	0	0
10781	3380469	yqjI	0.795	0.917	0	0
18269	3380699	yqjI	0.753	0.977	0	0
5754	3381079	STM3216	0.888	0.931	0	0
4387	3381251	STM3216	0.949	0.877	0	0
2315	3381596	STM3216	1.163	0.766	0	0
8591	3381839	STM3216	1.510	0.805	0	0
20016	3381942	STM3216	1.681	0.771	0	0
13564	3382284	STM3216	2.970	0.611	1	0
2909	3382356	STM3216	2.694	0.593	1	0
18816	3382724	STM3216	2.181	0.755	1	0
9373	3382919	aer	1.506	0.905	0	0
14367	3383212	aer	1.149	1.106	0	0

19213	3383391	aer	0.914	1.025	0	0
9927	3383558	aer	1.041	1.146	0	0
13803	3383841	aer	1.114	1.123	0	0
13580	3384130	aer	1.445	1.159	0	0
306	3384255	aer	1.445	1.141	0	0
8677	3384514	aer, oat	1.585	0.781	0	0
4013	3384769	oat	1.452	0.719	0	0
7333	3384929	oat	1.142	0.686	0	0
21380	3385020	oat	0.816	0.778	0	0
9998	3385458	oat	1.279	0.754	0	0
11960	3385645	oat	0.910	0.719	0	0
3568	3385867	oat, fadH	1.262	0.833	0	0
972	3386136	fadH	0.563	0.622	0	0
6955	3386208	fadH	1.051	0.807	0	0
19662	3386608	fadH	1.266	0.907	0	0
8104	3386714	fadH	0.748	0.580	0	0
15326	3386868	fadH	1.253	0.920	0	0
264	3387264	fadH	1.534	0.987	0	0
9357	3387514	fadH	1.228	0.878	0	0
14611	3387668	fadH	1.323	0.937	0	0
16472	3387808	fadH	0.881	0.747	0	0
12417	3387922	fadH	1.033	0.856	0	0
21352	3388398	ygjO	1.133	1.073	0	0
4129	3388624	ygjO	1.462	1.312	0	0
4172	3388862	ygjO	0.944	0.925	0	0
8314	3388996	ygjO	0.923	0.780	0	0
13459	3389237	ygjO, ygjP	0.945	0.990	0	0
20041	3389547	ygjO, ygjP	1.062	1.406	0	0
11888	3389694	ygjO, ygjP	0.935	1.118	0	0
9237	3389766	ygjO, ygjP	1.160	1.096	0	0
10284	3390066	ygjQ	1.019	1.061	0	0
11988	3390321	ygjQ	0.955	0.836	0	0
3538	3390639	ygjQ, ygjR	1.239	0.996	0	0
16298	3390743	ygjQ, ygjR	0.723	0.727	0	0
7712	3390963	ygjR	1.097	1.184	0	0
22090	3391106	ygjR	1.192	1.343	0	0

5353	3391480	ygjR	0.989	1.184	0	0
15237	3391651	ygjR	0.957	1.642	0	0
2660	3391983	ygjT	0.572	3.169	0	1
10249	3392259	ygjT	0.567	1.872	0	1
12272	3392403	ygjT	0.582	2.256	0	1
21865	3392540	ygjT	0.615	1.825	0	1
7236	3392813	ygjT	0.775	1.199	0	0
14154	3393083	ygjT, ygjU	0.858	1.077	0	0
5797	3393360	ygjU	0.870	1.510	0	0
4712	3393533	ygjU	0.721	1.573	0	0
2464	3393724	ygjU	0.660	2.062	0	1
10833	3393991	ygjU	0.792	2.079	0	1
8774	3394305	ygjU	0.839	1.519	0	0
9055	3394567	ygjU	0.708	1.745	0	0
1369	3394778	yqjA	0.713	1.588	0	0
15745	3394940	yqjA	0.724	2.032	0	0
14371	3395163	yqjA	0.729	1.551	0	0
5618	3395278	yqjA	0.556	1.140	0	0
6121	3395518	yqjA, yqjB	0.580	1.217	0	0
1919	3395809	yqjA, yqjB	0.856	1.436	0	0
2682	3396050	yqjB, yqjC	0.627	1.360	0	0
18556	3396237	yqjC	0.764	1.077	0	0
15092	3396432	yqjC, yqjD	0.917	1.056	0	0
17673	3396769	yqjD, yqjE	0.692	1.003	0	0
10922	3396858	yqjD, yqjE	0.983	1.085	0	0
15211	3397217	yqjE, yqjK	0.766	1.347	0	0
8705	3397327	yqjE, yqjK	0.714	1.293	0	0
5021	3397755	yqjK, yqjF	0.757	1.021	0	0
19534	3397860	yqjF	1.046	1.092	0	0
4211	3398058	yqjF, yqjG	1.202	0.810	0	0
11564	3398292	yqjF, yqjG	1.337	0.764	0	0
18156	3398407	yqjG	1.208	0.697	0	0
20413	3398706	yqjG	1.032	0.767	0	0
17794	3398886	yqjG	1.416	0.958	0	0
2931	3399257	yqjG, yhaH	1.771	0.681	0	0
18183	3399355	yqjG, yhaH	2.133	0.738	0	0

11940	3399664	yhaH	1.907	1.009	0	0
1444	3399852	yhaH	1.859	1.341	0	0
17077	3400210	yhaJ	1.347	1.131	0	0
21862	3400380	yhaJ	1.372	1.051	0	0
1210	3400557	yhaJ, yhaK	1.356	1.253	0	0
12195	3400907	yhaJ, yhaK	1.342	1.165	0	0
9059	3400986	yhaJ, yhaK	1.227	1.156	0	0
21728	3401311	yhaJ, yhaK	1.426	0.961	0	0
12915	3401482	yhaK, yhaL	1.267	1.017	0	0
2429	3401689	yhaL	1.409	1.176	0	0
21204	3402006	yhaN	1.113	1.523	0	0
7944	3402250	yhaN	0.861	0.844	0	0
11942	3402362	yhaN	1.527	0.912	0	0
17939	3402755	yhaN	1.367	0.942	0	0
8017	3402834	yhaN	0.978	0.713	0	0
4308	3403095	yhaN	1.253	0.700	0	0
4165	3403326	yhaN, yhaO	2.414	0.614	1	0
6514	3403422	yhaN, yhaO	2.693	0.711	1	0
19685	3403836	yhaO	1.903	0.693	0	0
19175	3403950	yhaO	1.894	0.663	0	0
13243	3404106	yhaO	1.063	0.563	0	0
2462	3404289	yhaO	1.296	0.796	0	0
22322	3404657	yhaO	1.241	0.711	0	0
13887	3404734	yhaO	1.370	0.728	0	0
4792	3405145	tdcG	1.471	0.780	0	0
19170	3405409	tdcG	0.459	0.407	0	0
15452	3405679	tdcG	1.110	0.870	0	0
17116	3405845	tdcG	1.485	0.886	0	0
6808	3405925	tdcG	1.510	0.767	0	0
1737	3406328	tdcG	1.158	0.726	0	0
16277	3406412	tdcG, tdcE	1.264	0.698	0	0
18493	3406697	tdcG, tdcE	1.499	0.720	0	0
20224	3406917	tdcE	1.379	0.741	0	0
18823	3407259	tdcE	1.220	0.641	0	0
4658	3407475	tdcE	1.583	0.700	0	0
21182	3407757	tdcE	1.067	0.625	0	0

12454	3407834	tdcE	1.517	0.795	0	0
14168	3408069	tdcE	0.667	0.594	0	0
7765	3408400	tdcE	1.838	0.617	0	0
17473	3408579	tdcE	3.309	0.662	1	0
20416	3408796	tdcE, tdcD	3.516	0.520	1	0
16860	3408940	tdcE, tdcD	2.952	0.581	1	0
5470	3409168	tdcD	1.944	0.622	0	0
14955	3409350	tdcD	2.301	0.557	1	0
9738	3409742	tdcD	3.869	0.571	1	0
339	3409895	tdcD	4.044	0.712	1	0
13800	3410219	tdcD, tdcC	1.417	0.551	0	0
2398	3410428	tdcC	1.631	0.848	0	0
9343	3410660	tdcC	0.910	0.701	0	0
13136	3410853	tdcC	1.141	0.597	0	0
18889	3410967	tdcC	1.518	0.677	0	0
19029	3411319	tdcC	2.873	0.553	1	0
15592	3411567	tdcC, tdcB	3.322	0.484	1	0
17095	3411822	tdcB	2.808	0.551	1	0
19714	3411925	tdcB	3.109	0.559	1	0
11750	3412207	tdcB	2.917	0.558	1	0
18355	3412310	tdcB	3.509	0.606	1	0
11176	3412752	tdcB, tdcA	2.051	0.666	0	0
1588	3412913	tdcA	1.830	0.644	0	0
7702	3413058	tdcA	1.904	0.706	0	0
8567	3413335	tdcA	2.122	0.553	0	0
4264	3413656	tdcA	2.173	0.495	0	0
19054	3413885		1.812	0.570	0	0
20906	3413967		2.113	0.650	0	0
13890	3414210		1.265	0.906	0	0
534	3414577	rnpB	1.093	1.156	0	0
19581	3414794	rnpB	0.905	2.426	0	1
20949	3414906	rnpB	0.683	3.287	0	1
3198	3415223	garK	0.919	2.532	0	1
15348	3415320	garK	0.981	2.179	0	1
6644	3415632	garK	0.600	0.681	0	0
1649	3415797	garK	1.244	1.465	0	0

15794	3416085	garK	0.834	1.010	0	0
10723	3416316	garK	0.734	0.934	0	0
13424	3416609	garK, garR	1.199	1.624	0	0
20343	3416794	garK, garR	0.835	1.065	0	0
11626	3417049	garR	1.042	1.371	0	0
9043	3417299	garR	0.821	1.005	0	0
9965	3417529	garR, garL	1.177	0.908	0	0
4034	3417655	garR, garL	1.231	0.761	0	0
20515	3417822	garL	2.572	1.092	1	0
5489	3418184	garL, garD	4.132	0.653	1	0
8005	3418320	garL, garD	4.238	0.634	1	0
5701	3418630	garD	4.696	0.608	1	0
3086	3418721	garD	4.993	0.689	1	0
14840	3419114	garD	2.314	1.059	1	0
14658	3419369	garD	1.301	0.814	0	0
14225	3419541	garD	1.014	0.909	0	0
3548	3419693	garD	1.206	1.006	0	0
2161	3419807	garD	1.245	1.104	0	0
2199	3420164	garD	1.387	0.996	0	0
17544	3420333		1.173	0.791	0	0
20156	3420661	STM3251	1.236	0.860	0	0
2656	3420895	STM3251	1.169	0.595	0	0
9909	3420977	STM3251	1.158	0.460	0	0
11179	3421366	STM3251	1.026	0.891	0	0
2794	3421446	STM3251, agaR	0.858	0.834	0	0
13816	3421833	STM3251, agaR	0.729	0.757	0	0
17888	3421968	agaR	0.700	0.759	0	0
18656	3422105	agaR, STM3253	0.657	0.588	0	0
12315	3422319	agaR, STM3253	0.540	0.564	0	0
22238	3422591	agaR, STM3253	0.552	0.484	0	0
7501	3422856	STM3253	0.673	0.470	0	0
20182	3423018	STM3253, STM3254	0.938	0.579	0	0
21920	3423331	STM3253, STM3254	1.849	0.643	0	0
17458	3423524	STM3254	2.638	0.513	1	0
6605	3423723	STM3254	4.244	0.526	1	0
12081	3424065	STM3254, STM3255	3.109	0.421	1	0

12386	3424232	STM3254, STM3255	2.568	0.483	0	0
1085	3424349	STM3254, STM3255	2.566	0.507	1	0
14819	3424661	STM3255	3.921	0.549	1	0
791	3424920	STM3255	4.131	0.578	1	0
19692	3425094	STM3255	3.204	0.740	1	0
5744	3425271	STM3255	1.029	0.469	0	0
3681	3425589	STM3255, STM3256	1.232	0.735	0	0
19964	3425794	STM3255, STM3256	1.092	0.643	0	0
4538	3425959	STM3256	1.225	0.671	0	0
9241	3426346	STM3256, STM3257	0.832	0.606	0	0
18409	3426595	STM3256, STM3257	0.672	0.640	0	0
22076	3426673	STM3257	0.640	0.619	0	0
19670	3426897	STM3257	0.857	0.646	0	0
10139	3427000	STM3257	0.934	0.795	0	0
12990	3427269	STM3257	0.900	0.924	0	0
13617	3427734	STM3257, STM3258	1.134	0.833	0	0
21972	3427892	STM3257, STM3258	1.190	0.787	0	0
12376	3428100	STM3258	1.037	0.638	0	0
12299	3428372	STM3258, STM3259	1.093	0.721	0	0
12638	3428548	STM3258, STM3259	1.159	0.640	0	0
18088	3428877	STM3260	1.330	0.680	0	0
16843	3428951	STM3260	1.196	0.634	0	0
16494	3429022	STM3260	0.824	0.662	0	0
9029	3429469	STM3260	1.258	0.740	0	0
10931	3429761	STM3260	1.285	0.808	0	0
4759	3429937	STM3260, STM3261	0.958	0.596	0	0
918	3430088	STM3260, STM3261	1.311	0.725	0	0
15609	3430280	STM3261	0.906	0.645	0	0
19189	3430461	STM3261	1.533	0.818	0	0
4728	3430668	STM3261	0.990	0.596	0	0
9790	3431097	STM3261, STM3262	0.865	0.963	0	0
9222	3431379	STM3262	0.893	0.670	0	0
6573	3431528	STM3262	1.165	0.873	0	0
17948	3431744	STM3262	1.173	1.072	0	0
19987	3431999	STM3262	1.068	1.075	0	0
13843	3432127	STM3262	1.107	1.168	0	0

21567	3432318		1.191	1.358	0	0
2753	3432713	yraL	0.851	0.969	0	0
13451	3432810	yraL	1.121	1.384	0	0
16042	3433216	yraL, yraM	0.973	2.065	0	1
20171	3433297	yraL, yraM	1.200	2.462	0	1
7521	3433501	yraL, yraM	1.151	2.189	0	1
10994	3433597	yraL, yraM	1.243	1.813	0	1
1596	3433892	yraM	0.967	1.158	0	0
16280	3433974	yraM	1.168	1.311	0	0
21467	3434562	yraM	1.663	1.527	0	0
5049	3434746	yraM	1.431	1.162	0	0
19046	3434934	yraM, yraN	1.860	1.563	0	0
20924	3435065	yraM, yraN	1.393	1.272	0	0
19305	3435297	yraM, yraN	0.863	1.017	0	0
16317	3435629	yraN, yraO	0.938	1.366	0	0
18966	3435740	yraO	1.132	1.598	0	0
8950	3435960	yraO, yraP	0.973	1.499	0	0
6307	3436291	yraO, yraP	1.107	1.479	0	0
16788	3436529	yraP	1.058	1.833	0	0
2841	3436711	yraP	0.929	1.529	0	0
13481	3436968	yraR	1.062	1.784	0	0
9102	3437209	yraR	1.248	1.574	0	0
14207	3437411	yraR, yhbO	1.068	0.952	0	0
6747	3437601	yraR, yhbO	1.000	0.899	0	0
17552	3437738	yraR, yhbO	1.265	0.845	0	0
21759	3437838	yraR, yhbO	1.076	0.744	0	0
13609	3438276	yhbP, yhbQ	0.930	1.022	0	0
18613	3438678	yhbP, yhbQ	1.152	1.675	0	0
15409	3438755	yhbP, yhbQ	1.111	1.827	0	0
17102	3438826	yhbP, yhbQ	0.813	1.343	0	0
4385	3439136	yhbS	0.897	1.549	0	0
11915	3439510	yhbS, yhbT	0.869	1.421	0	0
18186	3439656	yhbS, yhbT	0.938	1.146	0	0
18220	3439848	yhbS, yhbT	1.298	1.141	0	0
7170	3440227	yhbT, yhbU	1.604	0.858	0	0
7235	3440376	yhbT, yhbU	1.571	0.735	0	0

17357	3440668	yhbU	1.366	0.648	0	0
17235	3440755	yhbU	0.860	0.491	0	0
13817	3440905	yhbU, yhbV	1.214	0.762	0	0
17479	3441263	yhbV	1.910	0.660	0	0
14553	3441496	yhbV	1.368	0.532	0	0
18711	3441716	yhbV	1.541	0.679	0	0
15425	3441901	yhbV, yhbW	1.027	0.525	0	0
21297	3442299	yhbW	1.101	0.720	0	0
6127	3442506	yhbW	1.007	0.597	0	0
1208	3442710	yhbW	1.333	0.683	0	0
4076	3442953	yhbW	1.145	0.542	0	0
11681	3443035	yhbW	0.760	0.466	0	0
17342	3443286	yhbW	1.340	0.682	0	0
20373	3443640	STM3277	2.881	0.595	1	0
18492	3443774	STM3277, STM3278	3.614	0.576	1	0
4762	3443934	STM3277, STM3278	4.222	0.521	1	0
18914	3444276	STM3277, STM3278	4.652	0.436	1	0
19763	3444436	STM3277, STM3278	4.800	0.504	1	0
19626	3444528	STM3278	4.461	0.543	1	0
7313	3444624	STM3278	5.275	0.568	1	0
10530	3445058	mtr	2.361	0.696	1	0
6503	3445367	mtr	1.534	0.636	0	0
2809	3445677	mtr	1.090	0.671	0	0
17206	3445794	mtr	1.130	0.631	0	0
15049	3446026	mtr	0.978	0.729	0	0
11328	3446106	mtr	1.133	0.600	0	0
8914	3446478	deaD	0.904	0.631	0	0
21741	3446584	deaD	0.773	0.663	0	0
21152	3446913	deaD	1.373	0.945	0	0
5524	3447091	deaD	0.625	0.575	0	0
10205	3447526	deaD	0.900	0.913	0	0
20552	3447603	deaD	0.331	0.594	0	0
21875	3447978	deaD	0.552	2.028	0	1
18185	3448104	deaD	0.544	2.586	0	1
15619	3448390	deaD, nlpI	0.469	2.907	0	1
22300	3448523	deaD, nlpI	0.430	3.488	0	1

17608	3448744	nlpI	0.408	3.178	0	1
16594	3448874	nlpI	0.408	3.352	0	1
20609	3449004	nlpI	0.435	3.106	0	1
13145	3449378	nlpI, pnp	0.559	2.063	0	1
22002	3449661	pnp	0.575	1.630	0	1
13404	3449937	pnp	0.636	1.837	0	1
3858	3450067	pnp	0.655	1.822	0	1
8378	3450235	pnp	0.477	1.369	0	0
9745	3450516	pnp	0.721	2.026	0	1
1325	3450864	pnp	0.571	1.838	0	1
21674	3451019	pnp	0.901	2.466	0	1
15670	3451361	pnp	0.634	2.486	0	1
8392	3451505	pnp	0.542	2.191	0	1
7008	3451833	rpsO	0.544	2.478	0	1
12791	3451904	rpsO	0.594	2.574	0	1
5600	3452195	rpsO, truB	0.874	1.717	0	1
16544	3452408	truB	0.606	0.945	0	0
18516	3452743	truB	0.712	0.872	0	0
9446	3452868	truB	0.787	1.016	0	0
10006	3453187	truB, rbfA	0.986	1.186	0	0
510	3453357	truB, rbfA	1.446	1.220	0	0
4038	3453589	rbfA, infB	1.009	1.413	0	0
2238	3453679	rbfA, infB	0.623	1.143	0	0
8662	3454119	infB	0.849	1.298	0	0
632	3454284	infB	0.600	1.018	0	0
5578	3454590	infB	0.898	1.616	0	0
7653	3454732	infB	0.883	1.478	0	0
8629	3454874	infB	0.459	0.745	0	0
12250	3455147	infB	1.207	1.581	0	0
15179	3455257	infB	0.609	1.260	0	0
12797	3455592	infB	0.639	1.113	0	0
492	3455780	infB	0.827	1.364	0	0
19463	3456020	infB	0.905	1.350	0	0
8506	3456264	infB	0.837	1.359	0	0
1163	3456462	infB, nusA	0.822	1.515	0	0
5066	3456716	infB, nusA	0.827	1.316	0	0

21559	3456858	nusA	0.884	1.388	0	0
18827	3457127	nusA	0.791	1.313	0	0
5672	3457497	nusA	0.660	1.289	0	0
2302	3457665	nusA	0.501	1.081	0	0
4205	3457788	nusA	0.558	1.284	0	0
3794	3458129	nusA, yhbC	0.457	1.873	0	0
17897	3458270	yhbC	0.419	2.006	0	0
10285	3458665	yhbC, metY	0.371	3.605	0	1
21610	3458900	metY, argG	0.449	2.192	0	1
21084	3459021	argG	0.392	0.940	0	0
11487	3459322	argG	0.646	0.979	0	0
3131	3459424	argG	0.705	0.875	0	0
15289	3459829	argG	0.760	0.836	0	0
4020	3459900	argG	0.661	0.748	0	0
21100	3460105	argG	0.775	0.853	0	0
11166	3460376	STM3291	0.681	0.878	0	0
10669	3460515	STM3291	0.675	0.862	0	0
1739	3460733	STM3291	0.609	0.866	0	0
749	3461098	STM3291	0.578	0.922	0	0
14213	3461371	STM3291	0.472	1.343	0	0
7785	3461516	STM3291	0.431	1.876	0	1
19470	3461590	STM3291	0.397	2.344	0	1
4048	3461672	STM3291	0.379	2.739	0	1
1843	3462117	leuU, secG	0.350	4.156	0	1
14901	3462231	leuU, secG	0.422	3.792	0	1
485	3462302	secG	0.521	3.386	0	1
13503	3462386	secG, mrsA	0.477	3.080	0	1
7293	3463214	mrsA	0.950	1.622	0	0
16514	3463356	mrsA	0.826	1.564	0	0
3093	3463679	mrsA	0.733	1.764	0	1
10774	3463875	mrsA, folP	0.706	1.914	0	1
10074	3463980	mrsA, folP	0.770	1.933	0	1
3313	3464066	mrsA, folP	0.740	1.869	0	1
4773	3464572	folP	0.857	1.508	0	0
12234	3464644	folP	0.698	1.772	0	0
15407	3464909	folP, hflB	0.580	1.806	0	0

13798	3465003	folP, hflB	0.649	2.274	0	1
20051	3465441	hflB	0.547	2.460	0	1
14490	3465588	hflB	0.591	2.423	0	1
14445	3465742	hflB	0.530	2.126	0	1
11752	3465945	hflB	0.513	2.410	0	1
7752	3466378	hflB	0.435	1.894	0	1
11837	3466589	hflB	0.374	1.555	0	0
5693	3466792	hflB, ftsJ	0.510	2.086	0	1
7647	3467062	hflB, ftsJ	0.538	1.912	0	1
5213	3467202	ftsJ, yhbY	0.508	1.735	0	0
18189	3467477	ftsJ, yhbY	0.480	1.974	0	1
17012	3467585	ftsJ, yhbY	0.546	2.064	0	1
9146	3467766	ftsJ	0.588	1.476	0	0
13522	3467909	greA	0.531	1.569	0	0
18174	3468385	greA, dacB	0.474	2.031	0	0
7203	3468530	greA, dacB	0.520	2.666	0	1
20565	3468651	greA, dacB	0.716	3.038	0	1
11639	3468887	dacB	0.735	1.683	0	1
8997	3469195	dacB	0.747	1.302	0	0
15076	3469532	dacB	0.821	1.194	0	0
16401	3469768	dacB	0.786	1.272	0	0
2240	3469937	dacB	0.953	1.315	0	0
22153	3470034	dacB	0.994	1.131	0	0
16115	3470245	yhbZ	0.604	0.994	0	0
7481	3470494	yhbZ	0.787	1.021	0	0
9066	3470823	yhbZ	0.919	1.041	0	0
12709	3470958	yhbZ	1.107	1.253	0	0
8080	3471356	yhbZ	0.803	0.938	0	0
12346	3471561	yhbZ	0.801	0.966	0	0
7766	3471764	yhbZ	0.837	1.091	0	0
16529	3471894		0.618	0.882	0	0
8656	3472075		0.768	1.528	0	0
7880	3472529	rpmA	0.455	2.600	0	1
9314	3472741	rpmA	0.469	2.981	0	1
8561	3472939	rpmA, rplU	0.415	2.690	0	1
7143	3473167	rpmA, rplU	0.509	2.241	0	1

19576	3473394	rplU, ispB	0.558	2.052	0	1
3423	3473519	rplU, ispB	0.523	1.897	0	1
1577	3473789	ispB	0.630	1.624	0	1
9585	3474010	ispB	0.487	1.568	0	1
12857	3474364	nlp	0.523	1.214	0	0
12173	3474515	nlp	0.672	0.985	0	0
10156	3474798	nlp	0.752	1.159	0	0
11562	3474937	murA	0.800	1.198	0	0
6589	3475132	murA	0.928	1.424	0	0
15956	3475283	murA	0.822	1.506	0	0
21474	3475671	murA	0.771	1.401	0	0
10918	3475912	murA	0.400	0.761	0	0
5604	3476128	murA	0.825	1.653	0	0
4975	3476245	murA, yrbA	0.746	1.522	0	0
18701	3476420	murA, yrbA	0.350	0.677	0	0
21605	3476491	murA, yrbA	0.839	1.700	0	0
11028	3476939	yrbA, yrbB	0.854	1.495	0	0
3790	3477032	yrbB, yrbC	0.761	1.294	0	0
11133	3477416	yrbC	0.900	1.281	0	0
20169	3477754	yrbC, yrbD	0.938	1.229	0	0
5123	3477842	yrbC, yrbD	0.960	1.244	0	0
7890	3478116	yrbD	0.951	1.067	0	0
6717	3478254	yrbD, yrbE	1.138	1.405	0	0
22262	3478665	yrbE	1.110	1.407	0	0
16987	3478741	yrbE	1.287	1.546	0	0
16763	3478921	yrbE, yrbF	1.385	1.544	0	0
21629	3479185	yrbE, yrbF	0.949	1.288	0	0
5603	3479410	yrbF	0.690	0.865	0	0
16342	3479809	yrbF, yrbG	1.046	1.573	0	0
1914	3479882	yrbF, yrbG	0.868	1.611	0	0
20404	3480050	yrbF, yrbG	0.939	1.957	0	0
16680	3480351	yrbG	0.671	1.431	0	0
15914	3480679	yrbG, yrbH	0.980	2.044	0	1
3982	3480914	yrbG, yrbH	0.856	1.663	0	1
8035	3481080	yrbH	0.515	1.028	0	0
2456	3481377	yrbH	0.667	1.338	0	0

6111	3481576	yrbH, yrbI	0.841	1.666	0	1
2193	3481652	yrbH, yrbI	0.945	1.879	0	1
19934	3481918	yrbH, yrbI	0.923	1.952	0	1
17773	3482168	yrbI, yrbK	0.857	1.985	0	1
13006	3482505	yrbI, yrbK	0.696	1.650	0	1
12493	3482752	yrbK, yhbN	0.671	1.462	0	0
14928	3482823	yrbK, yhbN	0.667	1.354	0	0
813	3483178	yhbN, yhbG	0.864	1.523	0	0
9034	3483449	yhbN, yhbG	0.729	1.325	0	0
17244	3483610	yhbG	0.759	1.473	0	0
3467	3483877	yhbG	0.719	1.634	0	0
20534	3483957	yhbG, rpoN	0.682	1.290	0	0
1013	3484322	yhbG, rpoN	0.705	1.884	0	1
3950	3484394	rpoN	0.728	2.348	0	1
10163	3484554	rpoN	0.610	1.753	0	1
6100	3484761	rpoN	0.604	1.690	0	1
4185	3485221	rpoN	0.709	1.781	0	1
6957	3485359	rpoN	0.810	1.764	0	0
2805	3485450	rpoN, yhbH	0.552	1.427	0	0
6795	3485840	yhbH, ptsN	0.639	1.464	0	0
3103	3485949	yhbH, ptsN	0.628	1.288	0	0
15018	3486273	ptsN	0.527	1.407	0	0
16658	3486418	ptsN, yhbJ	0.467	1.188	0	0
19201	3486744	yhbJ	0.671	1.492	0	0
21236	3486970	yhbJ	0.807	1.801	0	0
15885	3487126	yhbJ	0.724	1.659	0	0
13915	3487423	yhbJ, ptsO	0.617	1.470	0	0
15401	3487638	ptsO	0.724	1.472	0	0
18749	3487893	ptsO, yrbL	0.671	1.487	0	0
16577	3488151	yrbL	0.702	1.143	0	0
19397	3488382	yrbL	0.887	1.148	0	0
2519	3488514	yrbL	0.883	1.300	0	0
6986	3488598	yrbL	0.820	1.191	0	0
18440	3488872	mtgA	0.884	1.384	0	0
5639	3489099	mtgA	1.023	1.568	0	0
10941	3489208	mtgA	0.955	1.460	0	0

15140	3489778	mtgA, yhbL	0.942	1.803	0	0
7832	3489911	mtgA, yhbL	0.589	1.204	0	0
21912	3490221	yhbL	0.614	2.612	0	1
8460	3490323	yhbL	0.701	3.305	0	1
17444	3490534	yhbL, arcB	0.443	1.658	0	0
11370	3490867	arcB	0.815	1.624	0	0
10278	3491067	arcB	0.784	1.204	0	0
419	3491186	arcB	0.562	0.867	0	0
8344	3491447	arcB	0.800	1.162	0	0
553	3491751	arcB	0.836	1.107	0	0
3034	3492009	arcB	0.747	1.098	0	0
2081	3492141	arcB	0.785	0.968	0	0
12938	3492356	arcB	0.983	1.042	0	0
2470	3492639	arcB	0.911	1.064	0	0
1185	3492819	arcB	1.003	1.103	0	0
11038	3493170	arcB, yhcC	0.942	1.054	0	0
5651	3493408	yhcC	0.618	1.216	0	0
17029	3493563	yhcC	0.465	1.150	0	0
8954	3493775	yhcC	0.507	2.877	0	0
21977	3494073	yhcC, gltB	0.606	1.706	0	0
1688	3494241	yhcC, gltB	0.517	1.026	0	0
7966	3494508	gltB	0.926	1.335	0	0
20773	3494747	gltB	1.134	1.125	0	0
980	3494885	gltB	0.837	0.971	0	0
21495	3495039	gltB	0.910	0.873	0	0
18647	3495451	gltB	0.834	0.701	0	0
16618	3495602	gltB	0.764	0.705	0	0
21378	3495752	gltB	1.090	0.872	0	0
12518	3495862	gltB	1.023	0.787	0	0
17147	3496174	gltB	0.978	0.810	0	0
10656	3496609	gltB	0.922	0.641	0	0
5952	3496699	gltB	0.873	0.704	0	0
8112	3497057	gltB	1.125	0.689	0	0
6549	3497258	gltB	1.193	0.701	0	0
7229	3497435	gltB	1.228	0.694	0	0
2881	3497621	gltB	1.415	0.743	0	0

17727	3497892	gltB	1.036	0.709	0	0
1274	3498076	gltB	1.365	0.814	0	0
15892	3498436	gltB	0.981	0.780	0	0
701	3498573	gltB, gltD	1.634	0.804	0	0
10823	3498851	gltB, gltD	1.279	0.751	0	0
19304	3499039	gltD	1.419	0.780	0	0
19109	3499168	gltD	1.068	0.738	0	0
6743	3499599	gltD	1.034	0.685	0	0
16152	3499790	gltD	0.731	0.517	0	0
8850	3499932	gltD	0.985	0.718	0	0
18030	3500000	gltD	0.791	0.634	0	0
22151	3500457	yhcG	0.991	0.749	0	0
21179	3500637	yhcG	1.121	0.649	0	0
9360	3500811	yhcG	1.079	0.506	0	0
12851	3501007	yhcG	1.104	0.674	0	0
17177	3501207	yhcG	1.155	0.723	0	0
17155	3501605	yhcG, STM3333	1.330	1.104	0	0
12738	3501695	STM3333	1.256	1.052	0	0
9700	3502000	STM3333	1.059	1.091	0	0
15439	3502211	STM3333	1.062	0.947	0	0
6627	3502287	STM3333	0.943	0.962	0	0
21549	3502757	STM3333, STM3334	1.132	1.118	0	0
11872	3502884	STM3333, STM3334	1.097	1.336	0	0
915	3503048	STM3333, STM3334	0.911	1.040	0	0
1895	3503407	STM3334	0.543	0.612	0	0
1839	3503516	STM3334	1.016	1.224	0	0
20310	3503692	STM3334	0.919	1.342	0	0
9949	3504080	STM3334	0.678	1.466	0	0
4249	3504189	STM3334	0.896	2.384	0	1
9497	3504564	yhcH	0.967	2.281	0	1
19292	3504678	yhcH	0.948	1.971	0	1
13626	3504873	yhcH	1.004	1.875	0	0
16810	3505062	yhcH, nanK	0.374	0.617	0	0
1824	3505385	nanK	1.268	2.133	0	0
1254	3505497	nanK	0.578	0.965	0	0
2133	3505787	nanK, nanE	1.028	1.773	0	0

8348	3506062	nanK, nanE	0.704	1.432	0	0
10395	3506202	nanK, nanE	0.902	1.882	0	1
7749	3506422	nanE	0.818	1.809	0	1
15657	3506505	nanE, nanT	0.791	1.914	0	1
10141	3506924	nanT	0.604	1.370	0	0
261	3507093	nanT	1.297	2.336	0	1
5857	3507317	nanT	0.920	2.025	0	1
13507	3507415	nanT	0.610	1.392	0	0
11895	3507866	nanT	0.748	2.005	0	0
6013	3507984	nanT	0.678	1.681	0	0
2834	3508257	nanT, nanA	0.762	2.178	0	1
17457	3508625	nanA	0.653	1.919	0	1
15680	3508805	nanA	0.564	1.811	0	0
20897	3508982	nanA	0.601	1.645	0	0
2941	3509270	nanA, yhcK	0.609	1.428	0	0
2798	3509581	yhcK	0.754	1.674	0	0
9272	3509792	yhcK	0.597	1.672	0	0
21998	3509888	yhcK	0.779	2.260	0	1
549	3510181	yhcK, sspB	0.707	2.052	0	1
21477	3510328	yhcK, sspB	0.555	1.758	0	1
20466	3510590	sspB, sspA	0.694	2.218	0	1
6392	3510708	sspB, sspA	0.664	1.864	0	1
17855	3511056	sspA	0.569	2.426	0	1
9302	3511186	sspA	0.568	2.998	0	1
7176	3511366	sspA	0.526	2.575	0	1
436	3511783	STM3343	0.718	1.258	0	0
20362	3511976	STM3343	0.542	0.929	0	0
7668	3512225	STM3343	0.615	1.306	0	0
218	3512296	STM3343	0.651	1.271	0	0
11818	3512667	rpsI	0.451	2.512	0	1
14081	3512766	rpsI	0.462	2.640	0	1
6161	3513201	rpsI, rplM	0.535	3.058	0	1
18771	3513277	rplM	0.529	2.801	0	1
18959	3513363	rplM	0.525	2.845	0	1
8130	3513598	rplM, yhcM	0.519	1.824	0	1
16081	3513750	yhcM	0.726	1.483	0	0

5510	3514213	yhcM	0.869	1.309	0	0
3709	3514558	yhcM, yhcB	0.798	1.570	0	0
14103	3514829	yhcM, yhcB	0.787	1.881	0	1
1192	3514930	yhcM, yhcB	0.805	2.029	0	1
9711	3515224	yhcB, degQ	0.822	1.858	0	1
8381	3515373	degQ	0.706	1.404	0	0
17309	3515689	degQ	0.914	1.211	0	0
10588	3515818	degQ	1.050	1.272	0	0
6898	3515985	degQ	1.089	1.182	0	0
5014	3516339	degQ	1.091	1.382	0	0
14662	3516564	degQ, degS	0.956	1.638	0	0
1983	3516717	degQ, degS	0.669	1.211	0	0
20857	3516857	degS	0.870	1.758	0	0
4123	3517119	degS	0.917	1.623	0	0
20082	3517492	degS	1.048	1.475	0	0
9226	3517759	degS	1.202	1.407	0	0
1402	3517975	STM3350	1.051	1.149	0	0
5727	3518046	STM3350	0.838	1.085	0	0
15679	3518314	STM3350	1.100	0.890	0	0
8193	3518591	STM3350	1.077	0.562	0	0
8398	3518669	STM3350	1.089	0.610	0	0
8432	3518900	STM3350, oadB	1.610	0.693	0	0
1181	3519062	STM3350, oadB	1.934	0.733	0	0
19574	3519229	oadB	2.168	0.853	0	0
18464	3519699	oadB	1.350	0.654	0	0
18392	3519886	oadB	1.148	0.436	0	0
7055	3520006	oadB	1.672	0.597	0	0
5292	3520337	oadB, oadA	1.942	0.663	0	0
9865	3520468	oadB, oadA	2.487	0.781	0	0
20917	3520696	oadA	1.247	0.481	0	0
17940	3520991	oadA	2.126	0.745	0	0
21802	3521293	oadA	1.912	0.679	0	0
10281	3521446	oadA	1.971	0.731	0	0
15855	3521807	oadA, oadG	1.017	0.771	0	0
21892	3522063	oadA, oadG	4.973	0.663	1	0
4819	3522172	oadA, oadG	4.374	0.574	1	0

3894	3522527	oadG, STM3354	4.171	0.738	1	0
424	3522737	STM3354	3.503	0.651	1	0
18769	3522838	STM3354, STM3355	4.504	0.567	1	0
15848	3523053	STM3354, STM3355	2.207	0.657	1	0
5023	3523384	STM3355	1.173	0.619	0	0
13844	3523628	STM3355	1.164	0.596	0	0
14346	3523772	STM3355, STM3356	1.175	0.620	0	0
3718	3524058	STM3355, STM3356	1.001	0.568	0	0
11475	3524229	STM3356	0.918	0.559	0	0
15623	3524491	STM3356	0.884	0.553	0	0
11194	3524752	STM3356	0.484	0.658	0	0
5909	3524885	STM3356	0.719	0.651	0	0
1883	3525136	STM3356	0.606	0.582	0	0
19287	3525353	STM3356, STM3357	0.705	0.725	0	0
11995	3525494	STM3356, STM3357	0.717	0.791	0	0
20046	3525918	STM3357, STM3358	0.728	0.895	0	0
9185	3526137	STM3357, STM3358	0.697	0.886	0	0
13557	3526370	STM3357, STM3358	0.878	1.279	0	0
7246	3526572	STM3358	0.723	1.329	0	0
19381	3526664	STM3358, mdh	0.753	1.740	0	1
12695	3526742	STM3358, mdh	0.812	1.741	0	1
6129	3527119	mdh	0.709	1.829	0	0
22119	3527504	mdh	0.648	1.967	0	0
22191	3527662	mdh, argR	0.742	1.567	0	0
5133	3527773	mdh, argR	0.567	1.498	0	0
20236	3528014	mdh, argR	0.598	1.295	0	0
5064	3528445	argR, yhcN	0.777	0.838	0	0
3756	3528554	yhcN	0.776	0.802	0	0
7707	3528805	yhcN, STM3362	0.913	0.747	0	0
19677	3529103	STM3362	0.702	0.649	0	0
2907	3529202	STM3362	0.764	0.739	0	0
17184	3529413	STM3362	0.921	0.759	0	0
6712	3529710	yhcO	0.851	0.696	0	0
9433	3529857	yhcO	0.766	0.739	0	0
19168	3530079	yhcO, yhcP	0.956	0.743	0	0
12827	3530169	yhcO, yhcP	0.953	0.699	0	0

475	3530512	yhcP	1.394	0.795	0	0
2440	3530660	yhcP	1.218	0.615	0	0
3837	3530822	yhcP	1.086	0.833	0	0
16827	3531183	yhcP	1.098	0.830	0	0
20070	3531596	yhcP	1.224	0.941	0	0
11413	3531709	yhcP	1.118	0.770	0	0
15843	3531917	yhcP	1.279	0.784	0	0
8182	3532294	yhcP, yhcQ	1.099	0.729	0	0
13228	3532473	yhcQ	1.154	0.766	0	0
7776	3532677	yhcQ	1.041	0.637	0	0
18512	3532865	yhcQ, qseA	1.505	0.832	0	0
9299	3533080	yhcQ, yhcR	0.928	0.649	0	0
17154	3533283	yhcQ, yhcR	1.481	0.752	0	0
530	3533517	yhcR, qseA	1.485	0.845	0	0
20799	3533884	qseA	1.500	1.188	0	0
9637	3534130	qseA	1.004	1.242	0	0
5999	3534208		1.194	1.295	0	0
14223	3534550	tldD	1.235	1.557	0	0
3980	3534778	tldD	1.056	1.266	0	0
20864	3534928	tldD	0.880	1.126	0	0
7710	3535205	tldD	0.625	0.950	0	0
1219	3535381	tldD	0.675	0.738	0	0
21647	3535643	tldD	1.305	1.388	0	0
16867	3535762	tldD	0.950	1.233	0	0
5008	3536149	tldD, yhdP	0.904	1.206	0	0
12254	3536267	yhdP	1.051	1.518	0	0
14382	3536541	yhdP	0.931	1.300	0	0
7539	3536701	yhdP	0.603	0.862	0	0
9207	3536931	yhdP	0.884	1.211	0	0
6030	3537182	yhdP	1.272	1.180	0	0
18084	3537319	yhdP	1.147	1.279	0	0
4524	3537657	yhdP	1.319	0.970	0	0
10335	3537844	yhdP	1.157	1.071	0	0
12085	3538060	yhdP	1.111	1.214	0	0
13966	3538419	yhdP	1.081	1.373	0	0
14519	3538543	yhdP	0.931	1.085	0	0

21563	3538794	yhdP	1.074	1.344	0	0
5097	3539089	yhdP	0.928	1.286	0	0
5691	3539260	yhdP	1.102	1.699	0	0
4705	3539516	yhdP	1.075	2.007	0	1
13613	3539822	yhdP, cafA	0.809	1.786	0	1
2631	3539894	yhdP, cafA	0.730	1.575	0	1
13271	3540000	yhdP, cafA	0.878	1.531	0	0
18449	3540323	cafA	0.772	1.206	0	0
7900	3540485	cafA	0.933	1.352	0	0
1575	3540678	cafA	0.747	1.515	0	0
18329	3541134	cafA	0.763	1.641	0	1
11757	3541286	cafA, yhdE	0.779	1.647	0	1
5100	3541407	cafA, yhdE	0.464	0.954	0	0
251	3541799	yhdE	0.678	1.950	0	1
15618	3541969	yhdE, mreD	0.751	2.127	0	1
17933	3542104	yhdE, mreD	0.839	2.786	0	1
4818	3542252	yhdE, mreD	0.986	2.775	0	1
3803	3542650	mreD, mreC	0.871	2.027	0	1
5949	3542985	mreC	0.776	1.997	0	1
19644	3543056	mreC	0.821	2.078	0	1
18103	3543288	mreC	0.859	2.394	0	1
14408	3543434	mreC	0.764	2.159	0	1
17998	3543903	mreB	0.755	1.969	0	1
2874	3543977	mreB	0.646	1.547	0	0
5110	3544270	mreB	0.739	2.216	0	1
21310	3544502	mreB	0.618	2.263	0	1
2736	3544829	mreB	0.836	2.448	0	1
14646	3544906	mreB, yhdA	0.608	1.426	0	0
15191	3545194	yhdA	0.783	1.421	0	0
18051	3545402	yhdA	0.852	1.380	0	0
673	3545474	yhdA	0.723	1.389	0	0
16934	3545915	yhdA	0.870	1.149	0	0
1294	3546072	yhdA	0.940	1.153	0	0
15146	3546167	yhdA	0.975	1.026	0	0
9625	3546401	yhdA	0.977	1.130	0	0
2972	3546701	yhdA, yhdH	0.948	1.371	0	0

6834	3546954	yhdA, yhdH	1.064	1.201	0	0
18974	3547113	yhdA, yhdH	0.848	0.867	0	0
8881	3547499	yhdH	1.012	1.116	0	0
22044	3547756	yhdH, STM3377	1.042	1.362	0	0
16975	3547935	yhdH, STM3377	1.293	1.239	0	0
5169	3548098	STM3377	1.033	0.928	0	0
8040	3548327	STM3377	1.038	0.897	0	0
21937	3548471	STM3377	1.124	0.936	0	0
9410	3548801	STM3377, STM3378	1.017	0.798	0	0
1389	3549085	STM3378	0.862	0.967	0	0
17883	3549336	STM3378	0.742	1.111	0	0
1545	3549561	STM3378	0.660	1.321	0	0
22250	3549790	accB	0.789	1.482	0	0
13291	3549863	accB	0.808	1.534	0	0
20096	3550112	accB, accC	0.732	1.520	0	0
9699	3550436	accB, accC	0.797	1.508	0	0
4750	3550608	accC	0.793	1.576	0	0
12118	3550718	accC	0.677	1.268	0	0
19390	3551135	accC	0.982	1.538	0	0
14967	3551379	accC	0.994	1.424	0	0
18774	3551535	accC, yhdT	0.611	0.899	0	0
3586	3551802	accC, yhdT	1.017	1.359	0	0
17962	3551874	accC, yhdT	0.927	1.132	0	0
11893	3552007	yhdT, panF	1.164	1.224	0	0
22223	3552271	panF	1.012	1.035	0	0
20853	3552638	panF	1.236	0.970	0	0
21855	3552959	panF	0.663	0.883	0	0
20610	3553044	panF	0.950	0.887	0	0
1962	3553298	panF, prmA	0.931	0.937	0	0
6770	3553675	panF, prmA	1.170	0.744	0	0
10991	3553884	prmA	0.856	0.745	0	0
19859	3553971	prmA	1.034	0.803	0	0
22303	3554279	prmA	0.912	0.836	0	0
8474	3554561	prmA	0.924	0.727	0	0
13304	3554676		0.939	0.698	0	0
10573	3554841	yhdG	1.111	0.803	0	0

14126	3555166	yhdG	1.064	1.027	0	0
4752	3555373	yhdG	0.924	1.131	0	0
1904	3555624	yhdG	0.759	0.887	0	0
21201	3555796	yhdG, fis	0.924	0.942	0	0
4913	3556027	yhdG, fis	0.791	0.771	0	0
22317	3556274	fis, yhdJ	0.827	0.796	0	0
7041	3556346	fis, yhdJ	0.762	0.691	0	0
3848	3556630	yhdJ	0.833	0.688	0	0
17520	3557031	yhdJ	0.678	0.623	0	0
20485	3557249	yhdJ, yhdU	0.879	0.753	0	0
9591	3557420	yhdJ, yhdU	0.791	0.636	0	0
19524	3557648	yhdU, STM3388	0.796	0.741	0	0
3960	3557871	STM3388	0.753	0.760	0	0
6261	3557948	STM3388	0.957	0.833	0	0
18822	3558309	STM3388	0.959	0.828	0	0
9070	3558592	STM3388	1.007	0.904	0	0
20350	3558788	STM3388	1.180	0.989	0	0
20166	3559139	STM3388	1.003	0.851	0	0
12001	3559309	STM3388	0.928	0.631	0	0
4143	3559553	STM3388	0.992	0.552	0	0
15557	3559637	STM3388	1.714	0.602	0	0
17819	3559846	STM3388	5.140	0.686	1	0
7010	3560206	envR	4.926	0.499	1	0
1689	3560455	envR	3.933	0.499	1	0
21085	3560669	envR, acrE	4.659	0.405	1	0
21534	3560918	envR, acrE	4.695	0.422	1	0
8181	3561094	envR, acrE	4.236	0.607	1	0
12635	3561377	acrE	2.525	0.556	1	0
18793	3561476	acrE	1.878	0.605	0	0
6389	3561751	acrE	1.282	0.592	0	0
11465	3561828	acrE, acrF	1.118	0.561	0	0
2255	3562252	acrF	1.300	0.762	0	0
5666	3562496	acrF	0.720	0.641	0	0
290	3562729	acrF	1.107	0.667	0	0
2257	3562851	acrF	1.101	0.686	0	0
10976	3563215	acrF	1.282	0.761	0	0

19239	3563332	acrF	0.883	0.793	0	0
16491	3563499	acrF	0.914	0.891	0	0
6862	3563761	acrF	0.986	0.856	0	0
9470	3564037	acrF	1.145	0.720	0	0
9127	3564301	acrF	1.010	0.776	0	0
4407	3564580	acrF	0.908	0.704	0	0
4459	3564790	acrF	0.752	0.531	0	0
3583	3564901	acrF	0.706	0.579	0	0
1143	3565094	acrF	1.134	0.707	0	0
19177	3565428	yhdV	0.882	0.988	0	0
9199	3565590	yhdV	0.922	0.612	0	0
8251	3565907		0.904	0.905	0	0
10467	3565996		1.091	0.940	0	0
20102	3566281		0.961	1.372	0	0
13269	3566369		0.830	1.965	0	1
456	3566751	rrfF	0.490	5.330	0	1
17922	3566832	rrfF, thrV	0.285	1.728	0	0
2572	3567025	rrfF, thrV	0.360	4.578	0	1
18985	3567147	rrfF, thrV	0.422	4.449	0	1
1976	3567759	rrlD	0.338	5.546	0	1
19649	3567867	rrlD	0.338	4.838	0	1
5629	3568153	rrlD	0.343	5.103	0	1
20393	3568347	rrlD	0.370	4.942	0	1
2403	3568461	rrlD	0.337	5.396	0	1
9169	3568698	rrlD	0.349	5.062	0	1
3059	3568956	rrlD	0.369	5.370	0	1
993	3569314	rrlD	0.349	5.123	0	1
3579	3569444	rrlD	0.366	5.045	0	1
4715	3569587	rrlD	0.393	5.654	0	1
18305	3569875	rrlD	0.370	4.644	0	1
2451	3570209	rrlD	0.391	4.005	0	1
15354	3570380	rrlD, STM3397	0.334	5.190	0	1
15876	3570521	STM3397, rrsD	0.329	5.531	0	1
17380	3570643	STM3397, rrsD	0.351	6.018	0	1
14254	3570993	rrsD	0.343	6.203	0	1
12225	3571169	rrsD	0.332	6.171	0	1

14631	3571519	rrsD	0.402	5.803	0	1
10084	3571833	rrsD	0.352	4.830	0	1
20891	3571965	rrsD	0.372	4.341	0	1
10973	3572292	rrsD, yrdA	0.431	3.498	0	1
17886	3572416	rrsD, yrdA	0.431	2.726	0	1
14396	3572487	yrdA	0.474	2.685	0	1
4151	3572958	yrdA	0.573	1.559	0	0
21255	3573137	yrdB	0.678	1.781	0	0
13186	3573404	yrdB, aroE	0.581	1.209	0	0
15035	3573515	yrdB, aroE	0.777	1.593	0	0
10500	3573742	aroE	0.863	1.517	0	0
734	3574028	aroE	1.007	1.429	0	0
17495	3574126	aroE, yrdC	0.809	1.383	0	0
4590	3574566	yrdC	0.721	1.499	0	0
3207	3574651	yrdC, yrdD	0.659	1.604	0	0
3310	3574910	yrdC, yrdD	0.610	2.256	0	1
18811	3575181	yrdD	0.557	2.196	0	1
12328	3575388	yrdD, smg	0.700	2.192	0	1
11077	3575668	smg, smf	0.685	2.100	0	1
2924	3575906	smg, smf	0.857	1.579	0	0
9552	3575999	smg, smf	0.876	1.350	0	0
4055	3576275	smf	0.617	0.859	0	0
9771	3576528	smf, def	0.847	1.176	0	0
8592	3576838	smf, def	0.783	1.247	0	0
11271	3576918	smf, def	0.794	1.558	0	0
21815	3576999	smf, def	0.857	1.686	0	0
12935	3577321	def, fmt	1.017	1.907	0	1
16414	3577448	fmt	0.746	1.714	0	1
18654	3577930	fmt	0.953	1.651	0	1
18517	3578060	fmt, sun	0.938	1.901	0	1
8697	3578263	fmt, sun	0.547	0.792	0	0
3748	3578454	sun	0.379	0.513	0	0
1089	3578906	sun	0.925	1.108	0	0
21173	3579014	sun	0.636	0.609	0	0
16554	3579317	sun, trkA	1.014	0.896	0	0
5649	3579579	sun, trkA	0.752	0.676	0	0

20768	3579703	sun, trkA	0.919	0.860	0	0
3536	3579868	trkA	0.815	0.910	0	0
10910	3580144	trkA	0.914	0.865	0	0
19518	3580217	trkA	0.874	0.778	0	0
12466	3580643	trkA	1.111	0.961	0	0
17782	3580748	trkA	0.932	0.845	0	0
483	3581015	trkA, mscL	1.671	0.850	0	0
5802	3581221	mscL	1.779	0.979	0	0
21698	3581558	mscL	1.377	1.094	0	0
16874	3581752	STM3411	0.873	1.039	0	0
8770	3581858	STM3411	0.823	1.180	0	0
12694	3582285	STM3411, zntR	0.631	1.127	0	0
9443	3582365	zntR	0.518	1.072	0	0
13697	3582505	zntR, yhdN	0.632	1.474	0	0
11058	3582773	zntR, yhdN	0.481	1.598	0	0
22124	3582865	yhdN, rplQ	0.517	2.053	0	1
11916	3583381	rplQ, rpoA	0.440	2.812	0	1
10347	3583547	rplQ, rpoA	0.433	2.416	0	1
21448	3583638	rplQ, rpoA	0.442	2.760	0	1
19011	3583966	rpoA	0.392	2.925	0	1
14766	3584266	rpoA	0.381	2.253	0	1
1967	3584540	rpoA, rpsD	0.304	2.240	0	0
16178	3584824	rpsD	0.446	2.883	0	1
4224	3584905	rpsD	0.456	2.535	0	1
8747	3585082	rpsD, rpsK	0.299	1.739	0	0
21358	3585236	rpsD, rpsK	0.429	2.473	0	1
15373	3585552	rpsK, rpsM	0.369	2.368	0	1
6871	3585716	rpsK, rpsM	0.360	2.099	0	0
13042	3586052	rpsM, rpmJ	0.356	1.972	0	1
7047	3586343	rpmJ, secY	0.502	2.331	0	1
11473	3586605	secY	0.441	2.608	0	1
2270	3586839	secY	0.388	2.511	0	1
12065	3587008	secY	0.382	1.975	0	1
18331	3587122	secY	0.302	0.743	0	0
21336	3587323	secY	0.427	2.625	0	1
20938	3587759	secY, rplO	0.516	2.787	0	1

9528	3587967	rplO, rpmD	0.480	2.607	0	1
16894	3588041	rplO, rpmD	0.448	2.474	0	1
17145	3588369	rpmD, rpsE	0.482	2.245	0	1
18930	3588649	rpsE, rplR	0.453	2.032	0	1
4393	3588829	rpsE, rplR	0.357	2.509	0	1
8426	3589050	rplR, rplF	0.434	2.620	0	1
4566	3589250	rplR, rplF	0.436	2.567	0	1
14366	3589376	rplF	0.396	2.253	0	1
591	3589786	rplF, rpsH	0.452	2.799	0	1
19807	3589892	rpsH, rpsN	0.419	2.448	0	1
18586	3590125	rpsH, rpsN	0.394	2.159	0	1
22287	3590262	rpsN, rplE	0.393	2.299	0	1
10405	3590682	rplE	0.332	1.769	0	1
2633	3590798	rplE, rplX	0.336	1.930	0	1
15859	3591020	rplE, rplX	0.392	2.051	0	1
22127	3591107	rplE, rplX	0.381	2.321	0	1
12408	3591388	rplX, rplN	0.370	2.740	0	1
4602	3591736	rplN, rpsQ	0.450	1.958	0	1
2725	3591980	rpsQ, rpmC	0.485	1.354	0	0
17141	3592115	rpsQ, rpmC	0.539	1.292	0	0
5716	3592510	rplP, rpsC	0.580	1.157	0	0
20798	3592682	rplP, rpsC	0.656	1.433	0	0
10710	3592983	rpsC	0.405	0.908	0	0
19105	3593112	rpsC	0.561	1.215	0	0
21788	3593244	rpsC, rplV	0.665	1.385	0	0
20755	3593461	rpsC, rplV	0.545	1.488	0	0
12134	3593721	rplV, rpsS	0.699	1.445	0	0
942	3593816	rplV, rpsS	0.605	1.310	0	0
22160	3594172	rpsS, rplB	0.685	1.636	0	0
19784	3594593	rplB	0.604	1.712	0	0
15588	3594751	rplB, rplW	0.614	1.568	0	0
8651	3594917	rplB, rplW	0.417	1.208	0	0
8255	3595150	rplW, rplD	0.582	1.702	0	0
16143	3595371	rplW, rplD	0.612	2.150	0	1
12501	3595529	rplD, rplC	0.563	2.008	0	1
8220	3595827	rplD, rplC	0.545	1.917	0	1

11504	3596071	rplC	0.494	2.082	0	1
16147	3596171	rplC	0.469	2.525	0	1
12926	3596563	rplC, rpsJ	0.406	2.719	0	1
21522	3596732	rpsJ, hopD	0.442	3.571	0	1
12363	3596864	rpsJ, hopD	0.440	2.952	0	1
8305	3597102	hopD	0.550	1.189	0	0
22083	3597476	bfr	0.741	1.086	0	0
14356	3597682	bfr	0.809	0.966	0	0
14531	3597884	bfr	0.662	0.775	0	0
1836	3598083	bfr, bfd	0.814	1.034	0	0
3660	3598193	bfr, bfd	0.866	1.170	0	0
8453	3598266	bfr, bfd	0.757	1.168	0	0
6557	3598726	tufA	0.444	2.929	0	1
14070	3598997	tufA	0.406	3.152	0	1
2818	3599366	tufA	0.391	3.154	0	1
7814	3599487	tufA	0.514	2.016	0	0
1947	3599788	tufA, fusA	0.443	1.857	0	0
10690	3599909	tufA, fusA	0.436	1.392	0	0
8925	3600208	fusA	0.503	1.610	0	0
8257	3600297	fusA	0.440	1.426	0	0
17306	3600369	fusA	0.360	1.025	0	0
4135	3600847	fusA	0.558	1.656	0	0
17991	3600936	fusA	0.446	1.561	0	0
6313	3601334	fusA	0.404	1.282	0	0
5743	3601542	fusA	0.555	1.983	0	1
411	3601650	fusA	0.520	2.340	0	1
2335	3601798	fusA, rpsG	0.408	1.821	0	1
12243	3602227	rpsG	0.470	1.908	0	0
3376	3602298	rpsG	0.475	1.753	0	0
14931	3602549	rpsG, rpsL	0.473	1.864	0	0
16672	3602700	rpsL	0.459	2.923	0	1
4204	3603105	rpsL, yheL	0.526	1.850	0	1
1065	3603254	yheL, yheM	0.965	1.873	0	0
10881	3603325	yheL, yheM	0.741	1.524	0	0
15317	3603907	yheM, yheN	0.907	1.363	0	0
7595	3603983	yheN, yheO	0.897	1.163	0	0

16845	3604167	yheN, yheO	0.747	1.071	0	0
2409	3604502	yheO	1.013	1.297	0	0
16311	3604672	yheO	0.941	1.097	0	0
3276	3604782	yheO	1.165	1.153	0	0
14640	3605225	fkpA	0.642	0.782	0	0
3165	3605454	fkpA, slyX	0.622	1.006	0	0
17535	3605534	fkpA, slyX	0.657	1.257	0	0
14814	3605801	fkpA, slyX	0.721	1.665	0	0
15242	3605963	fkpA, slyX	0.616	1.582	0	0
7402	3606146		0.712	1.641	0	0
10777	3606468	slyD	0.570	2.091	0	1
18140	3606617	slyD	0.573	1.881	0	1
15663	3606884	slyD	0.635	2.128	0	1
936	3607179	slyD, yheV	0.999	1.386	0	0
15149	3607306	slyD, yheV	0.815	1.209	0	0
14242	3607648	kefB	0.828	1.385	0	0
9926	3607913	kefB	0.959	1.155	0	0
4769	3608181	kefB	0.841	0.867	0	0
12631	3608428	kefB	1.140	1.080	0	0
8156	3608540	kefB	0.960	1.024	0	0
18477	3608787	kefB	0.860	1.032	0	0
888	3608967	kefB	0.666	0.999	0	0
3550	3609324	kefB, yheR	0.892	1.258	0	0
2090	3609414	kefB, yheR	0.945	1.544	0	0
18136	3609635	yheR, yheS	0.921	1.509	0	0
2536	3609806	yheR, yheS	0.986	1.434	0	0
20969	3610026	yheS	0.901	1.309	0	0
8269	3610446	yheS	1.151	1.194	0	0
6304	3610572	yheS	0.655	0.792	0	0
588	3610768	yheS	1.222	1.425	0	0
641	3610912	yheS	1.284	1.369	0	0
17149	3611320	yheS	1.045	1.417	0	0
10326	3611499	yheS	0.771	1.543	0	0
4784	3611709	yheS	0.846	3.246	0	0
6828	3611898		0.597	1.640	0	0
6003	3612186	STM3461, yheT	0.900	1.374	0	0

4621	3612396	STM3461, yheT	1.008	1.222	0	0
8927	3612652	STM3461, yheT	0.975	1.205	0	0
6399	3612723	STM3461, yheT	0.916	1.357	0	0
4242	3613208	yheT	0.789	0.787	0	0
10599	3613437	yheT, yheU	0.902	1.171	0	0
3343	3613508	yheT, yheU	0.821	1.213	0	0
1533	3613851	yheU, prkB	0.853	1.399	0	0
19078	3613939	prkB	0.880	1.239	0	0
9724	3614180	prkB	0.562	0.899	0	0
7925	3614521	prkB	0.724	1.313	0	0
14300	3614635	prkB	0.725	1.284	0	0
10104	3614923	yhfA	0.774	2.094	0	1
6572	3615131	yhfA	0.630	2.169	0	1
4969	3615307	yhfA, crp	0.500	2.192	0	1
14014	3615656	yhfA, crp	0.459	1.707	0	0
11131	3615912	crp, yhfK	0.658	2.000	0	1
4168	3616001	crp, yhfK	0.577	1.788	0	1
5414	3616313	yhfK	0.616	2.279	0	1
19231	3616460	yhfK	0.631	2.407	0	1
6563	3616811	yhfK	0.697	1.480	0	0
479	3616982	yhfK	1.003	1.826	0	0
6070	3617238	yhfK	0.778	1.536	0	0
13638	3617477	yhfK	0.697	1.311	0	0
13020	3617729	yhfK	0.836	1.580	0	0
1191	3617985	yhfK	0.779	1.532	0	0
16123	3618056	yhfK	0.602	1.118	0	0
1642	3618272	yhfK	0.552	1.504	0	0
10282	3618667	argD	0.914	1.210	0	0
20561	3618813	argD	1.029	1.141	0	0
1061	3619114	argD	0.971	0.923	0	0
3477	3619226	argD	0.852	0.800	0	0
1259	3619417	argD	0.809	0.904	0	0
7947	3619646	argD	0.747	0.868	0	0
13925	3619728	argD	0.861	0.909	0	0
10036	3620148	pabA	0.617	0.673	0	0
12456	3620265	pabA	1.005	0.897	0	0

3667	3620337	pabA, fic	0.741	0.973	0	0
12111	3620632	pabA, fic	0.890	0.792	0	0
4544	3621071	fic, yhfG	0.810	1.264	0	0
14684	3621219	fic, yhfG	0.899	1.582	0	0
21344	3621587	ppiA, yhfC	0.893	1.363	0	0
3152	3621774	ppiA, yhfC	0.926	1.315	0	0
19698	3622046	ppiA, yhfC	0.687	0.759	0	0
2743	3622173	ppiA, yhfC	0.779	0.721	0	0
20843	3622393	yhfC	0.927	0.780	0	0
8475	3622714	yhfC	1.064	0.786	0	0
3395	3622805	yhfC	0.557	0.632	0	0
14604	3623009	yhfC	1.118	0.895	0	0
12644	3623449	nirB	1.126	0.692	0	0
20467	3623565	nirB	1.020	0.776	0	0
2491	3623862	nirB	1.243	0.793	0	0
17006	3624132	nirB	1.251	0.903	0	0
10659	3624361	nirB	1.345	0.878	0	0
6715	3624541	nirB	1.472	0.956	0	0
4979	3624657	nirB	1.351	0.954	0	0
11788	3624939	nirB	1.131	0.746	0	0
12993	3625160	nirB	1.364	0.787	0	0
5453	3625276	nirB	1.336	0.803	0	0
19361	3625681	nirB, nirD	1.090	0.712	0	0
15327	3625890	nirB, nirD	1.012	0.728	0	0
14102	3626032	nirD	1.371	0.737	0	0
21397	3626140	nirD	1.473	0.796	0	0
18016	3626294	nirD, nirC	1.587	0.665	0	0
20239	3626628	nirC	1.495	0.698	0	0
3303	3626750	nirC	1.501	0.758	0	0
15114	3627179	nirC, cysG	0.424	0.421	0	0
1501	3627339	nirC, cysG	1.153	0.778	0	0
13120	3627442	cysG	1.637	0.901	0	0
1251	3627592	cysG	1.307	1.106	0	0
3151	3628152	cysG	1.504	1.041	0	0
3673	3628400	cysG	1.696	0.786	0	0
12442	3628586	cysG	4.496	0.749	1	0

8297	3628794	cysG, bigA	3.945	0.546	1	0
13530	3629026	bigA	8.526	0.503	1	0
20818	3629267	bigA	4.187	0.571	1	0
4254	3629406	bigA	4.587	0.597	1	0
18372	3629827	bigA	1.830	0.686	0	0
1054	3629912	bigA	1.590	0.585	0	0
4289	3630141	bigA	1.595	0.620	0	0
15277	3630249	bigA	1.544	0.560	0	0
16498	3630490	bigA	0.990	0.674	0	0
14113	3630713	bigA	1.754	0.777	0	0
19990	3631115	bigA	0.894	0.706	0	0
3788	3631372	bigA	1.263	0.891	0	0
15628	3631600	bigA	1.465	1.144	0	0
15507	3631697	bigA	1.455	1.077	0	0
10474	3631944	bigA	1.027	1.102	0	0
16222	3632087	bigA	0.764	0.868	0	0
3801	3632456	bigA	1.299	0.793	0	0
22108	3632527	bigA	0.988	0.777	0	0
416	3632847	bigA	1.254	0.713	0	0
11330	3632927	bigA	1.608	0.677	0	0
21816	3633177	bigA	1.367	0.640	0	0
6624	3633495	bigA	1.167	0.734	0	0
21849	3633716	bigA	0.905	0.557	0	0
19388	3634034	bigA	0.652	0.440	0	0
7323	3634311	bigA	1.302	0.720	0	0
4878	3634504	bigA	1.299	0.815	0	0
11871	3634677	bigA	1.338	0.953	0	0
16511	3634940	bigA	1.594	0.840	0	0
21910	3635005		1.348	0.764	0	0
19918	3635279	tnpA_5	1.295	0.899	0	0
9417	3635480	tnpA_5, yhfL	1.278	0.793	0	0
15863	3635917	tnpA_5, yhfL	3.647	0.662	1	0
18126	3636023	tnpA_5, yhfL	3.372	0.719	1	0
17592	3636136		3.604	0.882	1	0
21505	3636432	trpS	1.617	1.207	0	0
17387	3636705	trpS	1.295	1.192	0	0

21641	3636996	trpS	1.003	1.212	0	0
11206	3637115	trpS	0.850	1.307	0	0
8298	3637223	trpS, gph	0.913	1.428	0	0
11654	3637696	gph	0.891	1.296	0	0
15741	3637983	gph, rpe	1.091	1.499	0	0
11311	3638219	gph, rpe	0.841	1.072	0	0
4925	3638352	gph, rpe	0.995	1.241	0	0
10689	3638528	rpe	0.928	1.411	0	0
11157	3638764	rpe, dam	0.773	1.373	0	0
5248	3639074	rpe, dam	0.935	1.443	0	0
10023	3639311	dam	0.852	1.365	0	0
9398	3639506	dam	0.817	1.522	0	0
5267	3639825	dam, damX	0.694	1.563	0	0
5683	3639903	dam, damX	0.569	1.132	0	0
12540	3640189	damX	0.931	2.131	0	1
8858	3640332	damX	0.906	2.078	0	1
13829	3640683	damX	0.606	1.554	0	0
21947	3640764	damX	0.709	1.784	0	0
13097	3641012	damX	0.646	1.541	0	0
3344	3641371	damX, aroB	0.673	1.714	0	1
21313	3641555	aroB	0.804	2.137	0	1
17767	3641643	aroB	0.740	2.221	0	1
2508	3642051	aroB	0.539	1.597	0	0
9057	3642217	aroB, aroK	0.567	2.042	0	0
15462	3642508	aroB, aroK	0.549	2.064	0	0
22263	3642605	aroB, aroK	0.536	2.408	0	1
15971	3642715	aroK	0.463	2.545	0	1
7513	3643084	aroK	0.594	1.955	0	1
2194	3643156	aroK	0.713	1.724	0	1
3135	3643388	hofQ	0.740	1.046	0	0
18216	3643588	hofQ	0.707	0.752	0	0
6303	3643836	hofQ	0.902	0.925	0	0
1010	3644148	hofQ	1.145	0.784	0	0
10195	3644410	hofQ, yrfA	0.678	0.657	0	0
18488	3644562	hofQ, yrfA	0.769	0.797	0	0
1117	3644710	hofQ, yrfA	0.763	0.638	0	0

4193	3645074	yrfA, yrfB	1.449	0.796	0	0
14999	3645403	yrfB, yrfC	0.495	0.569	0	0
15520	3645572	yrfB, yrfC	1.058	0.775	0	0
10027	3645749	yrfC, yrfD	0.955	0.686	0	0
896	3646142	yrfC, yrfD	1.175	0.911	0	0
927	3646267	yrfD, mrcA	1.351	0.822	0	0
925	3646604	yrfD, mrcA	1.054	0.835	0	0
20987	3646816	yrfD, mrcA	1.125	1.068	0	0
17974	3647067	mrcA	0.885	0.903	0	0
15363	3647190	mrcA	0.964	0.975	0	0
12047	3647484	mrcA	1.089	0.993	0	0
9065	3647611	mrcA	1.050	0.928	0	0
10479	3647839	mrcA	0.915	1.020	0	0
12076	3648073	mrcA	0.914	1.167	0	0
4079	3648387	mrcA	1.108	1.442	0	0
18778	3648491	mrcA	1.133	1.079	0	0
2895	3648811	mrcA	0.790	1.405	0	0
20278	3649139	mrcA	0.817	1.161	0	0
12100	3649227		0.776	1.322	0	0
10425	3649493	yrfE	0.998	1.511	0	0
5203	3649819	yrfE, yrfF	0.967	1.547	0	0
4010	3649940	yrfE, yrfF	1.416	1.111	0	0
18091	3650156	yrfE, yrfF	0.946	2.458	0	1
14289	3650413	yrfF	0.772	2.244	0	1
6490	3650484	yrfF	0.715	2.113	0	1
12290	3650712	yrfF	0.872	1.645	0	1
3332	3651064	yrfF	0.772	1.345	0	0
7187	3651161	yrfF	0.828	1.426	0	0
21172	3651232	yrfF	0.713	1.279	0	0
9198	3651623	yrfF	0.849	1.882	0	0
5004	3651694	yrfF	0.612	1.170	0	0
11344	3652259	yrfF, yrfG	0.778	1.763	0	1
14871	3652357	yrfG	0.838	1.661	0	1
13322	3652758	yrfG, yrfH	0.690	1.449	0	0
7633	3652852	yrfG, yrfH	1.027	1.924	0	0
4620	3653043	yrfH, yrfI	0.944	1.584	0	0

19577	3653431	yrfH, yrfI	0.860	1.444	0	0
4030	3653545	yrfI	0.857	1.538	0	0
15566	3653776	yrfI	0.953	1.636	0	0
1945	3654122	yrfI	0.873	1.864	0	1
9904	3654259	yrfI	0.872	1.886	0	1
21521	3654466	yhgE	0.752	1.493	0	0
22079	3654739	yhgE	1.138	1.449	0	0
7683	3654867	yhgE	0.934	0.937	0	0
8006	3655000	yhgE	1.127	0.860	0	0
7482	3655165	yhgE	0.681	0.758	0	0
5997	3655606	yhgE	1.174	0.910	0	0
7870	3655729	yhgE	0.993	0.889	0	0
15415	3656149	yhgE, pckA	0.805	1.199	0	0
18658	3656350	yhgE, pckA	0.757	1.382	0	0
9930	3656435	yhgE, pckA	0.622	1.270	0	0
17083	3656629	pckA	0.638	1.328	0	0
3305	3656817	pckA	0.590	1.167	0	0
19604	3657006	pckA	0.799	1.619	0	0
16365	3657108	pckA	0.628	1.300	0	0
602	3657561	pckA	0.898	1.987	0	0
17328	3657669	pckA	0.668	1.365	0	0
9740	3657863	pckA	0.716	1.493	0	0
16497	3658239	envZ	0.716	1.698	0	0
6348	3658596	envZ	0.503	0.765	0	0
17485	3658750	envZ	1.109	1.479	0	0
18067	3659020	envZ	1.274	1.588	0	0
15007	3659117	envZ	1.191	1.715	0	1
9787	3659423	envZ	1.055	1.647	0	1
2458	3659573	envZ	1.034	1.988	0	1
10764	3659848	envZ, ompR	1.062	1.510	0	0
7828	3660147	ompR, greB	1.277	1.448	0	0
5012	3660288	ompR, greB	1.352	1.298	0	0
2594	3660391	ompR, greB	1.502	1.372	0	0
12623	3660718	ompR, greB	1.549	1.256	0	0
2051	3661089	yhgF	0.988	1.016	0	0
21964	3661195	yhgF	1.031	1.015	0	0

21627	3661392	yhgF	0.888	1.065	0	0
6160	3661729	yhgF	1.037	1.156	0	0
2059	3661867	yhgF	1.251	1.380	0	0
17505	3661946	yhgF	0.946	1.034	0	0
7332	3662290	yhgF	1.312	1.091	0	0
5400	3662452	yhgF	1.395	1.211	0	0
8769	3662932	yhgF	1.771	1.298	0	0
7719	3663154	yhgF	1.464	1.146	0	0
8271	3663427	feoA	3.562	1.111	1	0
12482	3663632	feoA	3.270	0.901	1	0
3715	3663866	feoA, feoB	3.041	0.964	1	0
17531	3664055	feoA, feoB	2.733	0.884	1	0
21128	3664148	feoB	3.532	1.002	1	0
15576	3664314	feoB	2.368	0.949	1	0
17966	3664610	feoB	1.336	0.922	0	0
20975	3664940	feoB	1.311	1.197	0	0
8678	3665082	feoB	0.910	0.877	0	0
22185	3665195	feoB	1.153	1.075	0	0
1126	3665654	feoB	1.533	1.241	0	0
17281	3665897	feoB	0.763	0.882	0	0
20579	3666127	feoB, yhgG	1.173	0.997	0	0
15080	3666301	feoB, yhgG	0.787	0.732	0	0
7725	3666440	yhgG, STM3508	1.213	1.006	0	0
7840	3666559	yhgG, STM3508	1.037	0.805	0	0
12042	3667020	STM3508	1.271	0.980	0	0
5469	3667133	STM3508	1.295	0.968	0	0
1101	3667529	STM3508	0.902	1.422	0	0
7601	3667651	STM3508	0.963	1.335	0	0
14415	3667963	bioH	0.733	0.902	0	0
3859	3668065	bioH	1.001	1.386	0	0
7321	3668281	bioH, yhgH	0.876	1.139	0	0
9819	3668640	bioH, yhgH	0.923	1.003	0	0
8712	3668888	bioH, yhgH	0.977	1.150	0	0
2911	3669068	bioH, yhgH	0.864	1.335	0	0
15998	3669358	yhgI	1.005	1.902	0	0
4939	3669582	yhgI	0.623	1.125	0	0

9379	3669653	yhgI	0.895	1.716	0	0
16223	3669914	yhgI, gntT	0.834	1.812	0	0
14892	3670131	gntT	0.733	1.436	0	0
8802	3670319	gntT	0.765	1.154	0	0
1701	3670390	gntT	0.678	0.989	0	0
18730	3670643	gntT	0.735	1.113	0	0
4929	3670985	gntT	1.207	1.041	0	0
15574	3671348	gntT	1.048	1.059	0	0
2940	3671600	gntT	1.362	1.116	0	0
10174	3671778		1.263	1.033	0	0
18496	3671852	malQ	1.170	1.223	0	0
8326	3672181	malQ	1.073	1.299	0	0
3947	3672314	malQ	1.094	1.325	0	0
21813	3672463	malQ	1.154	1.580	0	0
15944	3672784	malQ	1.320	1.723	0	0
13934	3672899	malQ	1.119	1.518	0	0
12483	3673400	malQ	1.144	1.141	0	0
17459	3673534	malQ	0.615	0.786	0	0
11469	3673792	malQ	1.252	1.322	0	0
8459	3674125	malQ, malP	1.112	1.377	0	0
20648	3674254	malQ, malP	1.128	1.205	0	0
18572	3674508	malP	1.361	1.144	0	0
14192	3674828	malP	1.232	1.080	0	0
17532	3674940	malP	1.142	0.921	0	0
9338	3675144	malP	1.260	1.065	0	0
9702	3675349	malP	1.045	0.911	0	0
13786	3675515	malP	0.944	0.786	0	0
19589	3675834	malP	0.985	0.949	0	0
19760	3676026	malP	1.327	1.006	0	0
9268	3676292	malP	1.643	0.922	0	0
7857	3676456	malP, malT	2.123	1.124	0	0
4810	3676764	malT	1.805	1.468	0	0
1834	3676866	malT	1.622	1.483	0	0
14530	3677265	malT	1.054	1.591	0	0
20624	3677452	malT	1.065	1.889	0	0
4286	3677653	malT	0.803	1.235	0	0

3471	3677815	malT	0.567	1.052	0	0
16013	3678009	malT	0.675	1.031	0	0
15366	3678371	malT	1.001	1.630	0	0
4857	3678565	malT	0.935	1.461	0	0
12909	3678773	malT	1.091	1.440	0	0
1503	3679063	malT	1.047	1.641	0	0
16671	3679296	malT	0.743	1.320	0	0
19392	3679591		0.765	1.130	0	0
7347	3679817	STM3516	0.981	1.286	0	0
8939	3679936	STM3516	0.922	1.116	0	0
18115	3680209	STM3516, STM3517	0.915	1.074	0	0
12562	3680329	STM3516, STM3517	1.061	1.240	0	0
20661	3680400	STM3516, STM3517	0.661	1.012	0	0
13138	3680877	rtcA	0.915	0.899	0	0
13552	3681012	rtcA	0.883	0.879	0	0
11970	3681333	rtcA, rtcB	0.554	0.756	0	0
7090	3681429	rtcA, rtcB	1.099	1.145	0	0
11736	3681704	rtcA, rtcB	0.841	0.798	0	0
12914	3681851	rtcB	0.992	0.921	0	0
15057	3682054	rtcB	1.305	0.905	0	0
9913	3682406	rtcB	1.264	0.919	0	0
4663	3682521	rtcB	0.989	0.665	0	0
16607	3682592	rtcB	1.195	0.833	0	0
5163	3682680	rtcB	1.357	0.751	0	0
10684	3683348	STM3521	1.214	0.830	0	0
15288	3683622	STM3521	1.107	0.849	0	0
4774	3683775	STM3521	1.025	1.076	0	0
3374	3683918	STM3521	1.130	0.784	0	0
1672	3684303	STM3521	1.276	0.873	0	0
5259	3684453	STM3521, rtcR	1.459	0.946	0	0
15694	3684656	STM3521, rtcR	1.392	1.019	0	0
6281	3684847	STM3521, rtcR	1.448	1.082	0	0
16573	3685161	rtcR	0.729	1.040	0	0
8993	3685295	rtcR	0.821	0.973	0	0
9224	3685410	rtcR	1.123	1.278	0	0
12812	3685755	rtcR	1.068	1.918	0	0

2749	3685898	rtcR	1.168	2.815	0	1
5491	3686155	rtcR	1.214	3.141	0	1
18450	3686434	glpR	0.895	2.289	0	1
6733	3686788	glpR	0.921	1.715	0	1
9311	3687054	glpR	0.812	1.642	0	0
20931	3687175	glpR	0.837	1.601	0	0
9459	3687381	glpR, glpG	0.564	1.300	0	0
6869	3687698	glpG	0.851	1.748	0	0
685	3687988	glpG	0.585	2.209	0	1
1482	3688137	glpG	0.602	2.077	0	1
11839	3688357	glpG, glpE	0.516	1.728	0	0
1888	3688480	glpG, glpE	0.500	2.176	0	0
19044	3688689	glpE, glpD	0.456	2.069	0	0
8368	3689022	glpD	0.418	3.500	0	1
13921	3689260	glpD	0.457	2.895	0	1
18080	3689585	glpD	0.417	3.016	0	1
5206	3689798	glpD	0.411	2.817	0	1
2313	3689912	glpD	0.440	3.202	0	1
8093	3690201	glpD	0.436	3.360	0	1
9985	3690397	STM3527	0.489	2.818	0	1
9768	3690497	STM3527	0.526	2.654	0	1
3931	3690694	STM3527	0.696	2.050	0	1
13972	3691044	STM3527, STM3528	0.885	0.910	0	0
19545	3691316	STM3528	0.855	0.806	0	0
13914	3691435	STM3528	0.738	0.685	0	0
18201	3691704	STM3528	1.094	1.012	0	0
12956	3691987	STM3528	1.026	1.023	0	0
11815	3692215	STM3528	1.041	0.880	0	0
7590	3692332	STM3528	1.324	0.840	0	0
2272	3692675	STM3528, STM3529	1.065	0.689	0	0
10865	3692881	STM3529	1.182	0.736	0	0
11267	3693164	STM3529	0.953	0.641	0	0
17971	3693381	STM3529	0.824	0.587	0	0
17945	3693664	STM3529	0.693	0.483	0	0
19452	3693753	STM3529	0.777	0.573	0	0
3993	3693889	STM3529	0.967	0.531	0	0

7934	3694095		0.887	0.570	0	0
10563	3694455		1.060	0.740	0	0
6860	3694633		0.993	0.675	0	0
17541	3694781	STM3531	1.358	0.759	0	0
15709	3695169	STM3531	0.990	0.700	0	0
4096	3695345	STM3531	0.886	0.627	0	0
5113	3695574	STM3531	1.233	0.682	0	0
19582	3695716	STM3531	1.304	0.663	0	0
17172	3696113	STM3531	1.384	0.682	0	0
3038	3696347	STM3531	1.258	0.834	0	0
16927	3696508	STM3531	0.830	0.605	0	0
14695	3696769	STM3531, STM3532	1.224	0.655	0	0
21547	3696926	STM3531, STM3532	1.773	0.736	0	0
16777	3697081	STM3532	1.737	0.791	0	0
19833	3697430	STM3532	2.103	0.957	0	0
498	3697533	STM3532	2.358	1.041	0	0
13426	3697787	STM3532	1.707	0.984	0	0
18938	3698205	STM3533	1.269	1.139	0	0
1707	3698350	STM3533	1.347	1.033	0	0
18311	3698595	STM3533	1.502	1.054	0	0
17980	3698757	STM3533	1.376	0.958	0	0
20649	3698948	STM3533, glgP	1.174	0.793	0	0
4315	3699185	glgP	0.904	0.847	0	0
4130	3699271	glgP	1.011	0.924	0	0
20848	3699702	glgP	1.047	0.918	0	0
3708	3699859	glgP	1.285	0.936	0	0
15001	3700174	glgP	1.278	1.257	0	0
15973	3700290	glgP	0.563	0.763	0	0
4017	3700530	glgP	1.166	1.105	0	0
17581	3700834	glgP	1.265	1.146	0	0
5646	3701087	glgP	1.217	1.167	0	0
1495	3701331	glgP	1.434	1.395	0	0
13881	3701419	glgP, glgA	1.078	1.273	0	0
20179	3701646	glgP, glgA	1.280	1.368	0	0
20402	3701945	glgA	1.048	1.265	0	0
19467	3702261	glgA	1.166	1.035	0	0

10257	3702471	glgA	1.145	1.141	0	0
9228	3702667	glgA	0.651	0.805	0	0
15461	3702980	glgA, glgC	1.122	0.994	0	0
9571	3703161	glgA, glgC	1.282	1.015	0	0
9217	3703362	glgC	1.027	0.965	0	0
8669	3703629	glgC	1.237	1.030	0	0
11286	3703909	glgC	1.338	1.279	0	0
13372	3704069	glgC	1.260	1.014	0	0
2559	3704231	glgC, glgX	1.274	1.014	0	0
4399	3704350	glgC, glgX	0.896	0.842	0	0
16040	3704701	glgX	1.220	1.023	0	0
578	3704902	glgX	1.702	1.264	0	0
3816	3705201	glgX	1.546	1.031	0	0
10198	3705274	glgX	1.557	1.084	0	0
18243	3705597	glgX	1.252	0.939	0	0
15666	3705732	glgX	1.209	0.993	0	0
18155	3705941	glgX	1.148	0.794	0	0
4757	3706354	glgX, glgB	1.169	0.930	0	0
16727	3706565	glgB	1.066	0.836	0	0
5109	3706717	glgB	1.417	1.009	0	0
10117	3706937	glgB	1.387	0.907	0	0
17219	3707118	glgB	1.384	1.101	0	0
8573	3707524	glgB	1.150	1.103	0	0
2262	3707676	glgB	1.218	0.951	0	0
13917	3707953	glgB	1.232	0.878	0	0
312	3708213	glgB	1.133	1.009	0	0
6939	3708388	glgB	1.239	1.019	0	0
4669	3708487	glgB	1.112	0.981	0	0
21850	3708782	asd	0.975	0.958	0	0
6926	3708931	asd	0.590	0.778	0	0
10580	3709303	asd	0.771	0.960	0	0
20248	3709396	asd, yhgN	1.175	1.263	0	0
6478	3709710	asd, yhgN	0.730	0.993	0	0
9082	3709788	asd, yhgN	0.963	1.321	0	0
13867	3710023	asd, yhgN	1.161	1.185	0	0
13364	3710295	gntU	1.020	0.835	0	0

1303	3710670	gntU	1.039	0.802	0	0
9938	3710844	gntU	1.235	0.857	0	0
1059	3710947	gntU	0.883	0.689	0	0
8320	3711183	gntU	1.360	0.974	0	0
19064	3711469	gntU, gntK	1.153	0.756	0	0
19098	3711661	gntU, gntK	1.199	0.822	0	0
21890	3712034	gntK	1.055	1.372	0	0
20275	3712107	gntK	0.981	1.336	0	0
7289	3712473	gntR	1.079	1.216	0	0
10466	3712664	gntR	0.928	0.994	0	0
6623	3712770	gntR	0.918	0.888	0	0
7774	3713127	gntR	1.099	1.202	0	0
11542	3713270	gntR, yhhW	0.706	0.845	0	0
14645	3713382	gntR, yhhW	0.469	0.420	0	0
6076	3713877	yhhW	1.164	0.775	0	0
8596	3713977	yhhW	0.928	0.720	0	0
11900	3714369	yhhW, yhhX	0.872	0.535	0	0
6401	3714471	yhhX	0.909	0.528	0	0
1985	3714726	yhhX	1.009	0.643	0	0
15573	3714897	yhhX	0.854	0.677	0	0
13919	3715163	yhhX, yhhY	0.833	0.661	0	0
21658	3715363	yhhX, yhhY	0.837	0.684	0	0
1231	3715551	yhhX, yhhY	1.062	0.750	0	0
5467	3715767	yhhY	0.936	0.654	0	0
1320	3716130	STM3547	1.510	0.610	0	0
19521	3716280	STM3547	2.218	0.544	0	0
21623	3716447	STM3547	2.316	0.434	0	0
18744	3716651	STM3547	2.154	0.485	0	0
8679	3716978	STM3547	2.066	0.465	0	0
8800	3717296	STM3547, STM3548	2.092	0.568	0	0
9056	3717524	STM3547, STM3548	2.364	0.446	0	0
3247	3717754	STM3548	2.253	0.503	0	0
10305	3717861	STM3548	2.736	0.510	0	0
9107	3717966	STM3548, STM3549	2.671	0.469	0	0
15788	3718409	STM3549	2.970	0.562	1	0
21491	3718616	STM3549	3.436	0.592	1	0

4550	3718869	STM3549	1.835	0.593	0	0
4734	3719064	STM3549, STM3550	0.928	0.515	0	0
3956	3719224	STM3549, STM3550	1.046	0.498	0	0
4081	3719452	STM3550	0.957	0.550	0	0
10233	3719759	STM3550	1.145	0.594	0	0
8063	3719922	STM3550	0.777	0.657	0	0
20281	3720122	STM3550	1.338	0.727	0	0
16932	3720351	STM3550	1.246	0.785	0	0
17355	3720687	ggt	1.099	0.632	0	0
6163	3720868	ggt	1.326	0.686	0	0
4073	3721065	ggt	0.769	0.554	0	0
17014	3721257	ggt	1.369	0.696	0	0
4716	3721577	ggt	1.302	0.708	0	0
8896	3721782	ggt	1.324	0.760	0	0
8153	3722053	ggt, yhhA	1.351	0.973	0	0
17612	3722183	ggt, yhhA	0.770	0.715	0	0
16249	3722319	ggt, yhhA	1.067	0.958	0	0
6683	3722390	ggt, yhhA	1.024	0.951	0	0
18672	3722884	ugpQ	1.178	1.134	0	0
12083	3723125	ugpQ	1.006	1.003	0	0
16069	3723329	ugpQ	1.600	1.249	0	0
9604	3723480	ugpQ, ugpC	1.263	1.023	0	0
8500	3723891	ugpQ, ugpC	1.054	0.754	0	0
5309	3723978	ugpC	1.594	0.900	0	0
10866	3724270	ugpC	1.129	0.661	0	0
10212	3724526	ugpC	1.418	0.849	0	0
4910	3724747	ugpC, ugpE	1.251	0.748	0	0
9284	3724887	ugpC, ugpE	1.297	0.727	0	0
15847	3725131	ugpE	1.240	0.760	0	0
4200	3725406	ugpE, ugpA	0.796	0.731	0	0
17669	3725510	ugpE, ugpA	1.244	0.961	0	0
2277	3725771	ugpE, ugpA	1.188	0.843	0	0
19801	3726060	ugpA	1.677	1.181	0	0
5581	3726333	ugpA, ugpB	1.145	0.950	0	0
12767	3726542	ugpA, ugpB	0.872	0.697	0	0
20285	3726784	ugpB	1.042	1.143	0	0

11907	3726907	ugpB	1.101	1.256	0	0
5947	3727282	ugpB	1.002	1.016	0	0
19267	3727466	ugpB	1.095	0.999	0	0
20440	3727548	ugpB	1.102	1.056	0	0
10990	3727768	ugpB	0.953	0.669	0	0
5402	3728073	ugpB, STM3558	1.326	1.014	0	0
13343	3728253	STM3558	1.310	1.051	0	0
2319	3728496	STM3558, yhhV	0.995	0.876	0	0
16270	3728567	STM3558, yhhV	0.293	0.571	0	0
19090	3729126	livF	1.074	0.780	0	0
4227	3729329	livF	1.254	0.691	0	0
1991	3729487	livF, livG	0.604	0.524	0	0
21548	3729829	livG	1.350	0.683	0	0
18147	3729938	livG	1.422	0.746	0	0
5077	3730117	livG, livM	0.895	0.578	0	0
2731	3730210	livG, livM	0.751	0.606	0	0
13490	3730613	livM	1.344	0.765	0	0
10904	3730910	livM	1.162	0.609	0	0
2481	3731127	livM	1.366	0.678	0	0
18294	3731209	livM	1.351	0.756	0	0
3650	3731478	livM, livH	1.655	0.782	0	0
5404	3731799	livM, livH	1.355	0.682	0	0
8039	3731960	livH	1.531	0.820	0	0
7927	3732296	livH	1.082	0.689	0	0
22061	3732439	livH, livK	1.265	0.727	0	0
10845	3732602	livH, livK	1.167	0.771	0	0
10424	3732836	livK	1.182	0.655	0	0
15839	3733194	livK	0.898	0.630	0	0
6240	3733449	livK, yhhK	0.981	0.580	0	0
20478	3733675	livK, yhhK	0.833	0.479	0	0
21213	3733896	livK, yhhK	0.779	0.588	0	0
8691	3733983	yhhK, STM3566	0.892	0.587	0	0
13266	3734338	STM3566	0.751	0.630	0	0
6738	3734409		1.044	0.729	0	0
18848	3734485	livJ	0.860	0.762	0	0
20010	3734939	livJ	1.079	0.793	0	0

2988	3735094	livJ	0.961	0.762	0	0
2650	3735293	livJ	0.892	0.719	0	0
15397	3735601	livJ	0.472	0.635	0	0
14065	3735672	livJ	0.804	1.002	0	0
2197	3735869	livJ	0.873	1.411	0	0
16003	3736314	rpoH	0.524	1.898	0	0
16595	3736404	rpoH	0.545	2.425	0	1
1275	3736718	rpoH	0.535	2.760	0	1
15722	3736983	rpoH	0.484	2.400	0	1
16048	3737175	rpoH, ftsX	0.659	2.628	0	1
7547	3737450	ftsX	0.760	1.721	0	1
14053	3737720	ftsX	0.845	1.445	0	0
14884	3737794	ftsX	0.901	1.663	0	0
19841	3738098	ftsX, ftsE	0.926	1.552	0	0
15873	3738445	ftsX, ftsE	0.908	1.682	0	0
11411	3738674	ftsE	0.883	1.817	0	0
18781	3738887	ftsE, ftsY	0.931	1.903	0	0
6338	3739022	ftsE, ftsY	0.963	1.832	0	0
2643	3739339	ftsY	1.010	1.626	0	0
9384	3739585	ftsY	0.884	1.357	0	0
1411	3739768	ftsY	0.813	1.301	0	0
21954	3739840	ftsY	0.467	0.822	0	0
17148	3740164	ftsY, yhhF	0.791	1.245	0	0
11125	3740242	ftsY, yhhF	1.233	1.607	0	0
15599	3740466	ftsY, yhhF	0.684	1.036	0	0
19940	3740913	yhhF, yhhL	0.982	1.160	0	0
13143	3741148	yhhL	0.817	0.980	0	0
21553	3741323	yhhM, yhhN	1.154	1.355	0	0
15006	3741595	yhhM, yhhN	1.052	1.292	0	0
15959	3741691	yhhM, yhhN	1.219	1.421	0	0
21400	3741980	yhhM, yhhN	1.003	1.272	0	0
19949	3742084	yhhN, zntA	1.164	1.244	0	0
14390	3742409	zntA	1.316	1.194	0	0
15632	3742560	zntA	0.533	0.616	0	0
18998	3742798	zntA	1.197	0.875	0	0
10464	3743172	zntA	1.394	0.818	0	0

597	3743245	zntA	1.625	0.883	0	0
12879	3743466	zntA	1.553	1.141	0	0
12322	3743718	zntA	1.151	0.651	0	0
3068	3743935	zntA	0.801	0.697	0	0
16726	3744145	zntA	1.512	1.022	0	0
19454	3744516	zntA, tcp	2.136	1.053	0	0
14960	3744792	tcp	1.944	0.613	0	0
16823	3744869	tcp	1.733	0.752	0	0
18866	3745216	tcp	1.578	0.792	0	0
16181	3745314	tcp	1.319	0.748	0	0
7716	3745387	tcp	1.415	0.821	0	0
19981	3745870	tcp	1.259	1.006	0	0
7613	3746080	tcp	1.220	1.440	0	0
21259	3746379	tcp	1.065	1.619	0	1
12552	3746467	yhhQ	1.096	1.595	0	1
4105	3746729	yhhP, yhhQ	0.913	1.444	0	0
5059	3746889	yhhP, yhhQ	0.937	1.118	0	0
7177	3746963	yhhP, yhhQ	0.936	1.117	0	0
1676	3747459	yhhQ, STM3580	1.062	0.985	0	0
5245	3747565	STM3580	1.163	1.118	0	0
16580	3747637	STM3580	1.244	1.298	0	0
19303	3748180	STM3580	0.850	1.236	0	0
6569	3748374	yhhS	0.968	1.251	0	0
14440	3748541	yhhS	0.902	1.141	0	0
2054	3748686	yhhS	1.228	1.340	0	0
15980	3749135	yhhS, yhhT	0.967	1.044	0	0
7607	3749339	yhhS, yhhT	0.971	1.053	0	0
19288	3749535	yhhS, yhhT	1.140	0.951	0	0
6908	3749828	yhhS, yhhT	1.249	0.821	0	0
13253	3749934	yhhT	1.174	0.881	0	0
263	3750060	yhhT	1.221	0.958	0	0
8053	3750227	yhhT, acpT	1.217	0.853	0	0
15330	3750617	acpT	0.963	1.534	0	0
17539	3750876	acpT, nikR	1.013	1.475	0	0
19928	3751038	acpT, nikR	1.013	1.558	0	0
21108	3751320	nikR	0.826	1.355	0	0

17322	3751474	nikR	0.293	0.444	0	0
18219	3751825	yhhJ	1.112	1.604	0	0
17675	3751949	yhhJ	1.140	1.620	0	0
18881	3752175	yhhJ	1.106	1.240	0	0
17500	3752513	yhhJ	1.146	0.903	0	0
12031	3752732	yhhJ	1.069	0.933	0	0
3566	3752934	yhhJ, yhiH	1.245	0.887	0	0
2535	3753013	yhhJ, yhiH	1.544	0.941	0	0
21697	3753359	yhhJ, yhiH	1.360	1.020	0	0
15881	3753538	yhiH	1.273	0.924	0	0
13092	3753906	yhiH	1.300	0.976	0	0
7105	3754049	yhiH	0.816	0.706	0	0
21025	3754275	yhiH	1.717	1.116	0	0
14401	3754583	yhiH	1.507	1.016	0	0
10515	3754802	yhiH	1.407	1.060	0	0
9586	3754873	yhiH	1.180	0.938	0	0
3594	3755189	yhiH	0.411	0.450	0	0
5530	3755337	yhiH	1.632	1.087	0	0
11676	3755523	yhiH	0.572	0.649	0	0
1767	3755740	yhiH, yhiI	0.927	0.773	0	0
3026	3755971	yhiH, yhiI	0.659	0.804	0	0
17759	3756340	yhiI	1.280	1.419	0	0
6990	3756550	yhiI	0.817	1.015	0	0
9666	3756738	yhiI	1.010	1.503	0	0
7910	3756906	yhiI	1.030	1.401	0	0
6334	3756989	yhiI	1.038	1.427	0	0
6422	3757455	yhiN	1.098	1.299	0	0
14935	3757629	yhiN	0.814	0.915	0	0
4241	3757971	yhiN	0.989	1.255	0	0
19120	3758057	yhiN, pitA	0.588	0.808	0	0
18727	3758375	yhiN, pitA	1.039	1.780	0	1
12396	3758534	yhiN, pitA	0.848	1.697	0	1
10296	3758902	pitA	0.871	1.454	0	0
15545	3759037	pitA	0.687	1.182	0	0
1223	3759245	pitA	0.843	1.422	0	0
3421	3759594	pitA	0.715	1.457	0	0

11165	3759794	pitA	0.647	1.741	0	1
20813	3759940	pitA	0.763	1.815	0	1
15281	3760122	uspB	0.614	1.380	0	0
18891	3760416	uspB, uspA	0.683	1.429	0	0
7399	3760659	uspB, uspA	0.609	1.341	0	0
21219	3760809	uspB, uspA	0.732	1.875	0	0
9792	3760949	uspA	0.569	1.571	0	0
21984	3761130	uspA, yhiP	0.590	1.716	0	0
5261	3761400	yhiP	0.622	1.272	0	0
11711	3761635	yhiP	0.805	1.338	0	0
10529	3761724	yhiP	0.731	1.322	0	0
4822	3762197	yhiP	0.878	1.091	0	0
1586	3762318	yhiP	0.842	1.111	0	0
9579	3762499	yhiP	0.902	1.102	0	0
18415	3762743	yhiP	0.661	1.038	0	0
21428	3763038	yhiP	1.024	2.405	0	1
20497	3763167	yhiQ	0.918	2.416	0	1
9483	3763447	yhiQ	1.036	2.460	0	1
15547	3763904	yhiQ	1.257	2.008	0	1
8073	3764002	yhiQ, prlC	1.217	1.530	0	0
5015	3764256	yhiQ, prlC	1.514	1.619	0	0
16632	3764568	prlC	0.989	1.156	0	0
7887	3764824	prlC	1.064	1.214	0	0
9216	3764918	prlC	1.065	1.312	0	0
19564	3765111	prlC	1.133	1.539	0	0
8210	3765429	prlC	1.120	1.476	0	0
7931	3765646	prlC	1.011	1.545	0	0
13287	3765729	prlC	1.259	1.716	0	0
10062	3765955	prlC	1.161	1.558	0	0
1455	3766035	prlC	1.052	1.064	0	0
7635	3766523	STM3595	1.257	1.240	0	0
11643	3766763	STM3595	1.175	1.227	0	0
12502	3767069	STM3595	1.418	1.179	0	0
3914	3767247	STM3595, yhiR	1.125	1.019	0	0
16276	3767431	STM3595, yhiR	1.104	1.148	0	0
2136	3767569	STM3595, yhiR	0.974	1.119	0	0

17958	3767704	STM3595, yhiR	1.033	1.087	0	0
6059	3767852	STM3595, yhiR	1.028	1.126	0	0
14361	3768141	yhiR, gor	0.635	0.881	0	0
16299	3768557	gor	1.125	1.450	0	0
8695	3768875	gor	1.117	1.171	0	0
15037	3769024	gor	1.276	1.392	0	0
12551	3769345	gor	1.198	1.456	0	0
3777	3769419	gor	1.329	1.410	0	0
1650	3769566	gor	0.674	0.787	0	0
19309	3769687	gor	1.197	1.279	0	0
1327	3770002	STM3598	1.409	1.522	0	0
11768	3770290	STM3598	2.102	0.901	0	0
5612	3770652	STM3598	2.660	0.617	0	0
11088	3770729	STM3598	3.331	0.763	1	0
2681	3771125	STM3598, STM3599	4.037	0.593	1	0
18432	3771368	STM3598, STM3599	2.426	0.592	1	0
11929	3771568	STM3599	1.870	0.621	0	0
12321	3771815	STM3599	2.642	0.703	1	0
6394	3772035	STM3599	2.751	0.687	1	0
15802	3772217	STM3599	2.229	0.716	0	0
7914	3772425	STM3599	2.103	0.626	0	0
18872	3772704	STM3599, STM3600	3.368	0.608	1	0
3056	3772952	STM3600	3.363	0.533	1	0
750	3773087	STM3600	2.604	0.590	1	0
6234	3773275	STM3600	2.167	0.575	1	0
1544	3773489	STM3600, STM3601	2.406	0.619	0	0
17332	3773684	STM3600, STM3601	2.072	0.666	0	0
4118	3773945	STM3601	1.098	0.640	0	0
11674	3774324	STM3601	0.832	0.591	0	0
18694	3774553	STM3601, STM3602	0.808	0.539	0	0
10419	3774661	STM3601, STM3602	0.857	0.607	0	0
782	3774879	STM3602	0.842	0.585	0	0
18790	3775219	STM3602, treF	0.925	0.612	0	0
21158	3775440	STM3602, treF	0.820	0.620	0	0
20674	3775589	STM3602, treF	0.958	0.722	0	0
12804	3775749	treF	1.217	0.772	0	0

13474	3775940	treF	1.287	0.760	0	0
967	3776056	treF	1.561	0.783	0	0
8205	3776583	treF	0.942	0.839	0	0
12597	3776735	treF	1.274	1.284	0	0
15831	3777058	treF	1.109	1.569	0	0
14324	3777232	STM3604	0.952	1.737	0	0
11087	3777419	STM3604	0.898	1.431	0	0
21792	3777605	STM3604	1.007	1.681	0	0
10132	3777863	STM3604	0.582	0.947	0	0
18999	3778168	STM3604	1.029	1.421	0	0
4125	3778282	STM3604	0.887	1.495	0	0
3460	3778675	STM3604, STM3605	0.963	1.147	0	0
19835	3778908	STM3604, STM3605	1.400	0.876	0	0
21224	3779074	STM3604, STM3605	1.542	0.876	0	0
3562	3779285	STM3605	2.413	0.673	0	0
702	3779417	STM3605	2.754	0.620	1	0
4693	3779649	yhjB	3.301	0.805	1	0
21616	3779938	yhjB	2.653	0.706	1	0
10829	3780143	yhjB	3.069	0.553	1	0
4763	3780374	yhjB, yhjC	2.589	0.632	1	0
16459	3780586	yhjC	1.694	0.706	0	0
16587	3780866	yhjC	1.175	0.781	0	0
21432	3781066	yhjC	1.212	0.858	0	0
8827	3781221	yhjC, yhjD	1.024	0.842	0	0
17992	3781599	yhjD	1.159	1.131	0	0
8946	3781680	yhjD	1.084	1.092	0	0
2028	3781960	yhjD	0.967	0.913	0	0
672	3782246	yhjD	0.815	0.839	0	0
20880	3782378	yhjD	0.743	0.834	0	0
13118	3782462	yhjD	0.976	0.904	0	0
20967	3782862	yhjE	0.828	0.775	0	0
17700	3783067	yhjE	0.887	0.923	0	0
19145	3783153	yhjE	0.909	0.949	0	0
12218	3783360	yhjE	0.853	0.801	0	0
19863	3783809	yhjE	1.206	0.895	0	0
3981	3784000	yhjE	0.939	0.899	0	0

7426	3784320	yhjE	1.426	1.142	0	0
3990	3784406	yhjG	1.146	1.134	0	0
17287	3784613	yhjG	1.453	1.318	0	0
4949	3784839	yhjG	1.481	1.115	0	0
19973	3785130	yhjG	1.338	0.999	0	0
4029	3785226	yhjG	1.240	0.862	0	0
19366	3785589	yhjG	1.362	1.130	0	0
2416	3785900	yhjG	1.161	1.081	0	0
19243	3786027	yhjG	1.175	1.372	0	0
12680	3786377	yhjG	1.087	2.038	0	0
22131	3786467	yhjG	0.900	1.824	0	0
13446	3786549	yhjG	0.783	1.352	0	0
12799	3787059	yhjH	0.928	1.971	0	0
18391	3787294	yhjH, kdgK	1.028	1.587	0	0
11905	3787448	yhjH, kdgK	1.138	1.465	0	0
19180	3787560	yhjH, kdgK	1.132	1.149	0	0
15294	3787837	kdgK	1.089	1.042	0	0
19682	3788124	kdgK	1.154	1.508	0	0
3297	3788305	kdgK	1.092	1.466	0	0
20062	3788624	yhjJ	0.910	1.336	0	0
6305	3788727	yhjJ	1.182	1.317	0	0
13272	3789072	yhjJ	0.883	0.907	0	0
6387	3789369	yhjJ	0.961	1.139	0	0
8334	3789556	yhjJ	0.827	1.144	0	0
5620	3789637	yhjJ	0.927	1.105	0	0
1802	3789827	yhjJ	0.849	1.691	0	1
19343	3790138	yhjJ	0.688	2.345	0	1
5846	3790306	yhjJ, dctA	0.609	2.126	0	1
20361	3790673	dctA	0.512	1.256	0	0
15554	3790762	dctA	0.742	2.008	0	1
9190	3790990	dctA	0.597	1.693	0	1
3828	3791378	dctA	0.621	1.886	0	1
21677	3791489	dctA	0.581	1.813	0	0
5888	3791672	dctA	0.604	1.512	0	0
9968	3791905	dctA, yhjK	0.682	1.043	0	0
3150	3792107	yhjK	0.957	1.221	0	0

10704	3792433	yhjK	1.017	1.008	0	0
7325	3792651	yhjK	0.962	0.925	0	0
19542	3792929	yhjK	0.870	0.899	0	0
8211	3793219	yhjK	1.152	1.121	0	0
7212	3793323	yhjK	1.117	1.239	0	0
2196	3793605	yhjK	1.156	1.174	0	0
9007	3793773	yhjK	1.044	1.106	0	0
17210	3794110	yhjK, yhjL	1.153	0.937	0	0
270	3794290	yhjL	0.752	0.770	0	0
6162	3794364	yhjL	1.146	0.872	0	0
5995	3794801	yhjL	1.440	1.062	0	0
8431	3794875	yhjL	1.244	0.995	0	0
14762	3795063	yhjL	1.295	1.078	0	0
7742	3795225	yhjL	1.515	1.186	0	0
16248	3795624	yhjL	1.908	1.376	0	0
12987	3795749	yhjL	1.867	1.451	0	0
10711	3796095	yhjL	1.962	1.202	0	0
13051	3796290	yhjL	0.980	0.789	0	0
14511	3796609	yhjL	0.917	0.833	0	0
18670	3796811	yhjL	1.408	1.120	0	0
5176	3796969	yhjL	1.488	1.044	0	0
12592	3797172	yhjL	1.306	1.073	0	0
5129	3797410	yhjL, bcsC	1.124	1.016	0	0
12698	3797500	yhjL, bcsC	1.308	0.973	0	0
9140	3797774	yhjL, bcsC	1.387	1.189	0	0
20837	3797975	bcsC	1.235	1.048	0	0
14308	3798388	bcsC	1.047	0.979	0	0
16639	3798515	bcsC, yhjN	1.213	1.001	0	0
10821	3798650	bcsC, yhjN	0.778	0.736	0	0
9366	3798980	yhjN	1.292	1.013	0	0
16444	3799286	yhjN	1.386	1.004	0	0
20069	3799539	yhjN	1.360	1.032	0	0
10297	3799613	yhjN	1.374	1.120	0	0
17096	3799890	yhjN	0.663	0.775	0	0
605	3800223	yhjN	1.326	1.251	0	0
5990	3800366	yhjN	1.618	1.402	0	0

18669	3800444	yhjN	1.549	1.268	0	0
2314	3800813	yhjN, yhjO	1.677	1.040	0	0
20708	3800905	yhjN, yhjO	1.824	1.179	0	0
6134	3801015	yhjN, yhjO	1.718	1.135	0	0
13114	3801528	yhjO	1.245	1.195	0	0
15273	3801686	yhjO	1.412	1.057	0	0
16889	3801911	yhjO	1.269	0.980	0	0
8644	3802171	yhjO	1.013	0.801	0	0
13745	3802320	yhjO	0.734	0.673	0	0
17290	3802573	yhjO	1.239	0.870	0	0
5487	3802962	yhjO	1.559	1.016	0	0
758	3803165	yhjO	1.306	1.001	0	0
17215	3803291	yhjO	1.340	1.161	0	0
3721	3803655	yhjO, yhjQ	1.367	0.973	0	0
11963	3803888	yhjO, yhjQ	1.171	0.992	0	0
15501	3804108	yhjQ	2.246	1.193	0	0
18410	3804341	yhjQ, yhjR	2.362	1.198	0	0
6396	3804564	yhjQ, yhjR	2.426	1.254	1	0
18618	3804771	yhjR, yhjS	2.677	1.209	1	0
21782	3804935	yhjS	2.833	1.124	1	0
19131	3805043	yhjS	2.599	0.883	1	0
21418	3805181	yhjS	2.652	0.839	1	0
21447	3805456	yhjS	1.492	1.004	0	0
910	3805755	yhjS, yhjT	1.569	1.002	0	0
5542	3806007	yhjS, yhjT	1.440	0.972	0	0
15204	3806211	yhjT, yhjU	1.416	1.017	0	0
17927	3806397	yhjU	1.339	1.072	0	0
8904	3806520	yhjU	0.616	0.574	0	0
10784	3806716	yhjU	1.325	1.000	0	0
22077	3807289	yhjU	1.122	0.999	0	0
13244	3807368	yhjU	1.243	0.968	0	0
21555	3807643	yhjU	1.319	1.363	0	0
13159	3807938	yhjU	0.933	1.539	0	0
5416	3808165		0.760	2.373	0	1
14759	3808323	STM3624A	0.642	2.674	0	1
828	3808528	STM3624A, yhjV	1.103	2.316	0	1

10651	3808905	yhjV	1.800	0.897	0	0
18099	3809072	yhjV	3.051	0.726	0	0
10279	3809280	yhjV	1.983	0.580	0	0
18964	3809569	yhjV	3.219	0.628	1	0
11425	3809734	yhjV	3.436	0.608	1	0
13549	3809831	yhjV	3.650	0.576	1	0
10660	3810000	yhjV	4.309	0.582	1	0
2730	3810108	yhjV	4.576	0.559	1	0
16996	3810687	dppF	1.461	0.720	0	0
17179	3810855	dppF	1.386	0.861	0	0
19144	3810989	dppF	1.684	0.757	0	0
16461	3811366	dppF, dppD	1.242	0.766	0	0
19569	3811528	dppF, dppD	0.688	0.379	0	0
15064	3811777	dppF, dppD	1.781	0.885	0	0
21015	3811961	dppD	1.608	0.848	0	0
9288	3812278	dppD	1.611	1.009	0	0
4755	3812357	dppD, dppC	1.327	0.832	0	0
18846	3812508	dppD, dppC	1.416	0.772	0	0
3144	3812880	dppC	0.547	0.583	0	0
9274	3813209	dppC	1.502	1.023	0	0
5335	3813281	dppC, dppB	1.103	0.759	0	0
10316	3813550	dppC, dppB	1.044	0.906	0	0
7591	3813700	dppB	1.213	0.870	0	0
13526	3814031	dppB	0.664	0.732	0	0
2471	3814207	dppB	1.335	1.220	0	0
10005	3814426	dppB, dppA	1.070	0.962	0	0
1302	3814609	dppB, dppA	0.954	0.879	0	0
19016	3814937	dppA	1.075	0.911	0	0
5440	3815101	dppA	1.185	0.920	0	0
17084	3815444	dppA	1.009	0.973	0	0
20170	3815624	dppA	0.901	1.089	0	0
11733	3815734	dppA	0.918	1.138	0	0
9830	3816144	dppA	1.090	1.035	0	0
10378	3816282	dppA	1.044	0.790	0	0
19550	3816482		1.126	0.814	0	0
10801	3816791	STM3631	1.273	1.034	0	0

20158	3816960	STM3631	1.315	0.792	0	0
7551	3817142	STM3631	1.095	0.639	0	0
906	3817360	STM3631	1.587	0.739	0	0
21092	3817437	STM3631	1.504	0.746	0	0
6635	3817887	STM3631	1.474	0.783	0	0
15442	3818016	STM3631, STM3632	1.419	0.662	0	0
8715	3818368	STM3632	0.987	0.721	0	0
5526	3818510	STM3632	1.487	0.870	0	0
217	3818722	STM3632	1.317	0.866	0	0
15346	3819116	STM3632, STM3633	1.540	0.988	0	0
15736	3819217	STM3632, STM3633	1.447	0.961	0	0
1576	3819402	STM3632, STM3633	1.268	1.013	0	0
14557	3819788	STM3633	1.260	1.630	0	0
12896	3819875	STM3633	0.845	1.569	0	0
3212	3820172	STM3633	0.578	3.625	0	1
14222	3820347		0.622	4.479	0	1
10407	3820521	proK	0.544	4.866	0	1
5074	3820907	proK, yhjW	0.668	1.337	0	0
5355	3821051	yhjW	0.793	1.282	0	0
6349	3821271	yhjW	0.938	0.943	0	0
13978	3821544	yhjW	1.308	1.029	0	0
12150	3821676	yhjW	0.855	0.727	0	0
5643	3821916	yhjW	1.190	0.919	0	0
18994	3822243	yhjW	1.001	0.738	0	0
3928	3822385	yhjW	1.205	0.816	0	0
9897	3822565	yhjW, lpfE	0.857	0.768	0	0
18802	3822770	lpfE	0.942	0.859	0	0
4915	3822989	lpfE, lpfD	1.186	0.822	0	0
15723	3823354	lpfE, lpfD	1.765	0.786	0	0
13603	3823493	lpfD	1.868	0.989	0	0
20993	3823853	lpfD	1.362	0.675	0	0
6360	3823924	lpfD	1.754	0.827	0	0
14956	3824064	lpfD	1.405	0.786	0	0
14842	3824597	lpfC	1.081	0.635	0	0
9862	3824775	lpfC	0.695	0.536	0	0
12642	3824922	lpfC	1.397	0.712	0	0

8415	3825228	lpfC	1.361	0.567	0	0
13935	3825325	lpfC	1.515	0.581	0	0
22179	3825683	lpfC	1.538	0.839	0	0
13436	3825794	lpfC	1.264	0.907	0	0
19686	3826010	lpfC	0.656	0.767	0	0
16493	3826373	lpfC	1.326	0.634	0	0
8768	3826453	lpfC	1.851	0.722	0	0
3113	3826812	lpfC, lpfB	3.011	0.568	1	0
7069	3826985	lpfC, lpfB	3.905	0.595	1	0
21328	3827152	lpfB	4.972	0.500	1	0
10363	3827485	lpfB, lpfA	2.203	0.500	0	0
7929	3827561	lpfB, lpfA	2.044	0.541	0	0
7292	3827989	lpfA	3.541	0.487	1	0
21387	3828219	lpfA	3.080	0.572	1	0
7086	3828425	lpfA	3.742	0.772	1	0
15353	3828647	yhjY	1.868	0.764	0	0
1660	3828782	yhjY	1.799	0.860	0	0
716	3828980	yhjY, tag	1.335	0.980	0	0
2585	3829079	yhjY, tag	1.227	0.880	0	0
9641	3829417	yhjY, tag	1.128	1.231	0	0
7044	3829794	tag, yiaC	1.031	1.118	0	0
15729	3829894	tag, yiaC	1.049	1.054	0	0
14595	3830179	yiaC	1.090	1.056	0	0
13231	3830418	bisC	1.082	1.234	0	0
12828	3830672	bisC	1.215	1.178	0	0
3645	3830839	bisC	1.214	0.911	0	0
7128	3831078	bisC	1.188	0.939	0	0
21643	3831181	bisC	1.433	1.056	0	0
16169	3831304	bisC	1.588	1.221	0	0
11500	3831682	bisC	1.282	1.053	0	0
531	3832026	bisC	1.266	1.004	0	0
6904	3832252	bisC	1.204	0.854	0	0
7012	3832323	bisC, yiaD	1.155	0.869	0	0
18238	3832704	bisC, yiaD	2.117	0.928	0	0
4484	3832786	bisC, yiaD	1.455	0.751	0	0
13268	3833031	bisC, yiaD	1.601	0.924	0	0

12006	3833439	yiaE	1.688	0.980	0	0
21847	3833516	yiaE	1.625	0.824	0	0
7169	3833659	yiaE	1.787	0.915	0	0
1816	3834100	yiaE	1.201	0.966	0	0
1783	3834329	yiaE	1.310	1.038	0	0
14514	3834463	yiaE	1.293	1.330	0	0
7968	3834583	yiaE	1.207	1.426	0	0
9304	3834929	yiaF	0.977	1.336	0	0
303	3835190	yiaF	1.089	1.402	0	0
8403	3835284	yiaF	1.317	1.215	0	0
20862	3835503	yiaF, yiaG	1.733	0.868	0	0
8706	3835798	yiaF, yiaG	1.258	0.934	0	0
12416	3835951	yiaG, cspA	1.167	1.139	0	0
6425	3836104	yiaG, cspA	0.628	1.229	0	0
12087	3836271	cspA	0.667	1.743	0	0
5765	3836797	STM3650	0.777	1.014	0	0
230	3837001	STM3650	0.869	0.812	0	0
18667	3837190	STM3650	1.011	0.620	0	0
13654	3837402		1.137	0.616	0	0
22304	3837746	STM3651	1.146	0.655	0	0
18298	3837890	STM3651	0.919	0.618	0	0
9278	3838035	STM3651	0.878	0.847	0	0
21804	3838428	STM3651, STM3652	0.755	0.647	0	0
20492	3838665	STM3652, STM3653	0.826	0.598	0	0
3581	3838834	STM3652, STM3653	0.883	0.498	0	0
911	3839113	STM3653	1.602	0.614	0	0
16089	3839241	STM3653	1.617	0.661	0	0
4796	3839504	STM3653	2.319	0.571	0	0
9664	3839768	glyS	2.296	0.741	1	0
22205	3839919	glyS	2.412	0.956	1	0
13615	3840107	glyS	1.466	1.119	0	0
21139	3840243	glyS	1.106	1.124	0	0
19484	3840610	glyS	1.012	1.054	0	0
4124	3840803	glyS	1.201	1.382	0	0
8618	3840883	glyS	1.433	1.221	0	0
4365	3841210	glyS	1.327	1.261	0	0

3631	3841459	glyS	0.618	0.721	0	0
1423	3841779	glyS	1.132	1.147	0	0
20612	3842067	glyS, glyQ	1.223	1.130	0	0
20325	3842218	glyS, glyQ	0.674	0.855	0	0
12617	3842488	glyQ	0.974	1.466	0	0
707	3842712	glyQ	1.042	2.199	0	1
21599	3842943	glyQ, STM3657	1.074	1.683	0	1
341	3843213	STM3657, yiaH	1.112	1.059	0	0
20428	3843332	STM3657, yiaH	1.051	0.714	0	0
20279	3843672	yiaH	1.375	0.640	0	0
691	3843789	yiaH	1.368	0.606	0	0
5171	3843873	yiaH	1.679	0.729	0	0
19757	3844317	yiaH	1.778	0.710	0	0
15385	3844589	yiaB	2.334	0.761	0	0
5183	3844737	yiaB	2.286	0.723	0	0
369	3844890	yiaB, xylB	2.015	0.902	0	0
16942	3845111	yiaB, xylB	1.635	0.785	0	0
14229	3845292	xylB	1.018	0.532	0	0
8995	3845586	xylB	1.434	0.781	0	0
7450	3845896	xylB	1.228	0.700	0	0
9038	3846046	xylB	1.312	0.696	0	0
2372	3846306	xylB	0.972	0.754	0	0
7037	3846474	xylB, xylA	1.025	0.738	0	0
9225	3846692	xylB, xylA	0.709	0.569	0	0
894	3847017	xylA	1.047	0.676	0	0
1733	3847096	xylA	1.011	0.603	0	0
687	3847475	xylA	1.546	0.795	0	0
18831	3847748	xylA, xylR	2.569	0.542	0	0
595	3847871	xylA, xylR	2.763	0.668	0	0
17446	3848066	xylA, xylR	2.324	0.672	0	0
12963	3848161	xylA, xylR	2.914	0.751	1	0
12706	3848242	xylR	2.756	0.699	1	0
1211	3848761	xylR	1.557	0.850	0	0
2443	3849087	xylR	1.119	1.190	0	0
8145	3849306		0.777	1.438	0	0
8418	3849435	bax	0.905	1.482	0	0

20841	3849663	bax	0.801	1.705	0	0
21406	3849938	bax	0.661	1.389	0	0
1949	3850222	bax, malS	0.773	1.512	0	0
5390	3850312	bax, malS	0.988	1.358	0	0
2816	3850453	bax, malS	1.037	1.421	0	0
7791	3850795	malS	1.071	0.869	0	0
10847	3851134	malS	0.977	0.748	0	0
8707	3851312	malS	1.297	0.722	0	0
20173	3851606	malS	0.600	0.579	0	0
1234	3851773	malS	1.382	0.740	0	0
14460	3852059	malS	1.391	0.886	0	0
12487	3852161	malS	1.159	0.783	0	0
5031	3852483	malS, avtA	0.792	0.717	0	0
20172	3852674	avtA	0.874	0.842	0	0
2589	3852850	avtA	0.689	0.741	0	0
7660	3852984	avtA	1.127	0.794	0	0
804	3853333	avtA	0.901	0.712	0	0
16507	3853604	avtA	1.202	0.842	0	0
19908	3853747	avtA	0.565	0.485	0	0
16158	3853901	avtA	1.251	1.143	0	0
9215	3854204	ysaA	0.967	1.588	0	0
6120	3854475	ysaA	1.247	1.243	0	0
7566	3854668	ysaA, yiaJ	1.276	1.051	0	0
18510	3854899	ysaA, yiaJ	1.169	0.906	0	0
1561	3855220	yiaJ, yiaK	1.046	0.794	0	0
8902	3855436	yiaJ, yiaK	0.837	0.631	0	0
2674	3855611	yiaJ, yiaK	0.981	0.583	0	0
22025	3855776	yiaJ, yiaK	1.104	0.731	0	0
14777	3856137	yiaK	1.166	0.692	0	0
6685	3856288	yiaK, yiaL	0.874	0.509	0	0
21193	3856369	yiaK, yiaL	1.484	0.577	0	0
7084	3856757	yiaL, STM3670	4.282	0.733	1	0
5122	3856871	yiaL, STM3670	4.382	0.773	1	0
17875	3857040	yiaL, STM3670	4.442	0.758	1	0
4927	3857429	STM3670	2.425	1.219	1	0
17011	3857653	STM3670	2.905	0.730	1	0

5341	3857880	STM3670, yiaM	4.544	0.618	1	0
18007	3858178	yiaM	5.193	0.572	1	0
12284	3858366	yiaM, yiaN	4.213	0.541	1	0
13542	3858620	yiaM, yiaN	2.457	0.626	1	0
19424	3858771	yiaN	1.908	0.558	0	0
22240	3859126	yiaN	2.008	0.716	0	0
21750	3859266	yiaN	2.244	0.651	0	0
3139	3859417	yiaN	3.177	0.540	1	0
21222	3859684	yiaN, yiaO	7.140	0.622	1	0
8002	3859820	yiaN, yiaO	6.831	0.618	1	0
10382	3860048	yiaO	5.869	0.600	1	0
1858	3860142	yiaO	5.188	0.616	1	0
18447	3860622	yiaO, lyxK	1.786	0.696	0	0
8490	3860794	yiaO, lyxK	1.959	0.582	0	0
13501	3860951	lyxK	0.967	0.714	0	0
6750	3861207	lyxK	1.034	0.491	0	0
19823	3861459	lyxK	1.527	0.700	0	0
8635	3861682	lyxK	1.253	0.579	0	0
11850	3862062	lyxK, sgbH	1.908	0.761	0	0
3893	3862174	lyxK, sgbH	2.332	0.881	0	0
12806	3862372	lyxK, sgbH	2.047	0.681	0	0
10925	3862522	sgbH	2.238	0.714	0	0
22122	3862900	sgbH, sgbU	1.307	0.480	0	0
21273	3863090	sgbU	1.779	0.588	0	0
11205	3863446	sgbU	1.197	0.588	0	0
1019	3863566	sgbU, sgbE	1.623	0.569	0	0
4049	3863835	sgbU, sgbE	1.486	0.810	0	0
9741	3863928	sgbU, sgbE	1.670	0.653	0	0
11579	3864365	sgbE	1.658	0.869	0	0
13204	3864570	sgbE	1.439	0.951	0	0
18013	3864789		1.523	1.178	0	0
3611	3864863	STM3678	1.586	1.135	0	0
1944	3865020	STM3678	1.629	0.909	0	0
18533	3865445	STM3678, STM3679	3.297	0.770	1	0
20290	3865557	STM3678, STM3679	4.793	0.737	1	0
6053	3865868	STM3678, STM3679	3.985	0.651	1	0

752	3865977	STM3678, STM3679	3.281	0.673	1	0
19586	3866221	STM3679	2.062	0.686	0	0
9324	3866558	STM3679	1.521	0.722	0	0
5515	3866665	STM3679	1.067	0.623	0	0
20592	3867053	STM3679	1.705	0.897	0	0
7990	3867203	STM3679	1.198	0.539	0	0
3277	3867396	STM3679	1.236	0.641	0	0
7345	3867609	STM3679	1.543	0.925	0	0
2268	3867959	aldB	1.020	0.924	0	0
1007	3868207	aldB	1.519	1.466	0	0
20213	3868280	aldB	1.858	1.375	0	0
18628	3868636	aldB	1.263	0.752	0	0
9405	3868714	aldB	1.401	0.861	0	0
14127	3869022	aldB	1.390	0.740	0	0
6402	3869213	aldB, STM3681	1.470	0.754	0	0
2236	3869576	aldB, STM3681	1.247	0.670	0	0
17150	3869654	aldB, STM3681	1.525	0.746	0	0
7460	3869749	aldB, STM3681	1.467	0.715	0	0
365	3870158	STM3681	1.781	0.815	0	0
884	3870263	STM3681	1.782	0.758	0	0
11975	3870677		3.754	0.652	1	0
13568	3870868	selB	5.293	0.787	1	0
16205	3871019	selB	3.626	0.899	1	0
5241	3871224	selB	2.434	0.981	1	0
10045	3871457	selB	1.835	0.961	0	0
6995	3871790	selB	0.993	0.835	0	0
4147	3871943	selB	1.011	0.868	0	0
1336	3872193	selB	1.365	0.976	0	0
3095	3872492	selB	1.013	0.876	0	0
17398	3872678	selB, selA	1.667	1.103	0	0
2147	3872885	selB, selA	1.360	0.966	0	0
21476	3873025	selB, selA	1.547	1.199	0	0
10188	3873363	selA	1.657	0.957	0	0
16029	3873434	selA	1.073	0.919	0	0
21609	3873874	selA	1.161	1.135	0	0
19446	3874028	selA	0.846	0.786	0	0

12336	3874119	selA	0.769	0.776	0	0
21645	3874566	yibF	1.088	1.106	0	0
8416	3874829	yibF, mtlA	0.921	0.751	0	0
6372	3874901	yibF, mtlA	0.892	0.782	0	0
7806	3875102	yibF, mtlA	0.777	0.599	0	0
19921	3875272	mtlA	1.001	0.706	0	0
16243	3875599	mtlA	1.103	0.839	0	0
1747	3875866	mtlA	1.046	0.889	0	0
22175	3876005	mtlA	1.384	1.062	0	0
2720	3876251	mtlA	1.654	0.893	0	0
4282	3876462	mtlA	0.877	0.695	0	0
9293	3876675	mtlA	1.178	0.752	0	0
2220	3876966	mtlA, mtlD	1.310	0.872	0	0
15163	3877147	mtlA, mtlD	1.296	0.839	0	0
7444	3877364	mtlD	1.380	0.878	0	0
7972	3877520	mtlD	1.041	0.694	0	0
9450	3877916	mtlD	1.272	1.399	0	0
10161	3878011	mtlD	0.990	1.060	0	0
13308	3878215	mtlD, mtlR	1.118	1.173	0	0
14478	3878587	mtlR	1.034	1.186	0	0
19404	3878722	mtlR	1.214	1.264	0	0
15413	3879120	STM3688	1.730	0.989	0	0
9880	3879320	STM3688, yibL	1.785	0.793	0	0
16391	3879434	STM3688, yibL	1.688	0.794	0	0
5726	3879623	STM3688, yibL	1.865	0.743	0	0
3750	3879741	STM3688, yibL	1.731	0.674	0	0
4962	3880049	STM3690	3.195	0.565	1	0
16323	3880120	STM3690	3.887	0.599	1	0
2781	3880684	STM3690	2.620	0.465	0	0
18223	3880905	STM3690, STM3691	3.355	0.491	1	0
19980	3881115	STM3690, STM3691	3.542	0.472	1	0
21654	3881225	STM3691	4.260	0.618	1	0
6916	3881444	STM3691	3.263	0.556	1	0
1171	3881727	STM3691	1.625	0.736	0	0
2245	3882067	STM3691	0.764	0.571	0	0
1500	3882290	STM3691	1.511	0.930	0	0

12571	3882369	STM3691	0.987	0.859	0	0
12705	3882642	STM3691	0.906	0.736	0	0
5395	3882968	STM3691	0.927	0.683	0	0
14622	3883198	STM3691	1.186	0.813	0	0
1374	3883299	STM3691	1.088	0.756	0	0
7703	3883674	STM3691	1.081	0.779	0	0
14533	3883901	STM3691	0.994	0.583	0	0
12187	3884004	STM3691	1.058	0.684	0	0
3239	3884354	STM3691	0.785	0.763	0	0
11559	3884484	STM3691	0.704	0.661	0	0
2739	3884793	STM3691	0.884	0.723	0	0
15658	3884888	STM3691	0.859	0.709	0	0
6400	3885021	STM3691	0.731	0.794	0	0
11937	3885313	STM3691	0.965	1.080	0	0
13768	3885688	lldP	0.550	2.119	0	1
12257	3885830	lldP	0.505	2.685	0	1
9900	3885972	lldP	0.613	3.194	0	1
21233	3886178	lldP	0.568	3.226	0	1
2951	3886496	lldP	0.539	3.048	0	1
1437	3886658	lldP	0.405	2.229	0	1
3269	3886901	lldP	0.591	3.265	0	1
16105	3887269	lldP, lldR	0.471	2.219	0	1
3357	3887521	lldP, lldR	0.519	3.198	0	1
9795	3887759	lldR	0.577	3.579	0	1
7003	3887930	lldR, lldD	0.606	3.310	0	1
21803	3888207	lldR, lldD	0.666	3.499	0	1
18841	3888302	lldR, lldD	0.493	1.744	0	0
18893	3888485	lldD	0.758	2.817	0	1
18181	3888637	lldD	0.584	2.125	0	1
6521	3889010	lldD	0.802	3.498	0	1
3648	3889240	lldD, yibK	0.562	2.546	0	1
8661	3889425	lldD, yibK	0.682	2.661	0	1
10589	3889657	yibK	0.692	2.969	0	1
17913	3889934	yibK	0.881	2.256	0	1
1617	3890147	STM3696	0.906	1.760	0	1
21744	3890380	STM3696	0.920	1.379	0	0

6976	3890583	STM3696	0.727	0.893	0	0
9609	3890875	STM3696	0.911	0.972	0	0
6026	3891140	STM3696, STM3697	0.874	0.767	0	0
18709	3891333	STM3696, STM3697	0.763	0.648	0	0
6719	3891436	STM3696, STM3697	0.974	0.741	0	0
14216	3891743	STM3697	0.973	0.750	0	0
11697	3891986	STM3697	0.997	0.716	0	0
18899	3892179	STM3697	1.230	0.682	0	0
2102	3892480	STM3697, STM3698	1.744	0.690	0	0
4072	3892673	STM3698	2.039	0.666	0	0
19598	3892968	STM3698	2.665	0.604	0	0
14515	3893126	STM3698	2.603	0.798	0	0
15123	3893265	STM3698	2.861	0.646	0	0
11967	3893673	STM3698	1.286	1.296	0	0
10923	3893888	STM3698	0.922	1.522	0	0
16360	3894126	cysE	1.081	1.848	0	0
13628	3894369	cysE	1.079	1.576	0	0
1755	3894497	cysE	1.061	1.771	0	1
9328	3894731	cysE	0.864	2.241	0	1
21902	3894890	cysE	0.853	2.389	0	1
13076	3895086	cysE, gpsA	0.964	1.979	0	1
2842	3895159	cysE, gpsA	0.807	1.737	0	0
9931	3895478	gpsA	1.067	1.926	0	1
13219	3895823	gpsA	0.838	2.273	0	1
15416	3896006	gpsA, secB	0.702	2.044	0	1
14618	3896329	gpsA, secB	0.773	1.628	0	1
10343	3896428	gpsA, secB	0.746	1.435	0	0
5627	3896499	secB	0.779	1.421	0	0
16651	3896996	grxC, yibN	0.617	1.237	0	0
3829	3897289	yibN, pmgI	0.824	1.474	0	0
3503	3897469	yibN, pmgI	0.923	1.120	0	0
20923	3897569	yibN, pmgI	0.877	1.002	0	0
3389	3897818	pmgI	1.130	0.903	0	0
12241	3898123	pmgI	0.920	0.727	0	0
14697	3898385	pmgI	1.355	0.909	0	0
5233	3898515	pmgI	1.343	0.912	0	0

16186	3898827	pmgI, yibP	0.936	0.850	0	0
9391	3899131	yibP	0.723	1.069	0	0
20679	3899213	yibP	0.812	1.362	0	0
17952	3899412	yibP	1.195	1.899	0	0
15668	3899663	yibP	0.528	0.719	0	0
6048	3899754	yibP	1.454	1.816	0	0
252	3900187	yibP, yigQ	1.551	1.407	0	0
8262	3900372	yigQ	1.153	1.837	0	0
532	3900450	yigQ	1.060	1.550	0	0
4707	3900827	yigQ	1.227	1.317	0	0
21722	3900905	yigQ	1.239	1.271	0	0
17743	3901271	yigQ	1.969	0.942	0	0
8195	3901466	yibD	4.604	0.878	1	0
20734	3901779	yibD	6.897	0.753	1	0
3693	3902040	yibD	3.179	0.693	1	0
14464	3902211	yibD	3.616	0.762	1	0
1152	3902480	yibD	3.520	0.822	1	0
2466	3902563	yibD	2.515	0.792	1	0
11549	3902922	tdh	1.517	0.995	0	0
18929	3903011	tdh	1.306	1.063	0	0
17302	3903445	tdh	1.058	1.413	0	0
5622	3903629	tdh	0.960	1.582	0	0
8278	3903765	tdh	0.927	1.407	0	0
401	3903860	tdh	0.807	1.448	0	0
8727	3904023	tdh, kbl	0.904	1.357	0	0
8714	3904479	kbl	0.733	1.183	0	0
1725	3904777	kbl	0.882	1.340	0	0
6684	3904925	kbl, rfaD	0.976	1.031	0	0
3995	3905200	kbl, rfaD	0.846	1.236	0	0
8188	3905424	kbl, rfaD	0.881	1.173	0	0
7555	3905556	rfaD	0.985	1.196	0	0
18200	3905709	rfaD	0.931	1.143	0	0
2791	3906064	rfaD, rfaF	0.989	1.392	0	0
17271	3906289	rfaF	0.908	1.627	0	0
12727	3906506	rfaF	1.059	1.968	0	0
16386	3906832	rfaF, rfaC	0.906	1.264	0	0

9684	3907015	rfaF, rfaC	0.876	1.223	0	0
2641	3907285	rfaC	1.149	1.746	0	0
13572	3907376	rfaC	0.998	1.448	0	0
13234	3907667	rfaC	1.253	1.375	0	0
22092	3907792	rfaC, rfaL	1.585	1.408	0	0
16402	3908208	rfaC, rfaL	3.458	0.746	1	0
5340	3908348	rfaL	3.561	0.705	1	0
9899	3908677	rfaL	3.623	0.497	1	0
9144	3908753	rfaL	3.209	0.506	1	0
4803	3909062	rfaL	2.812	0.609	1	0
6356	3909237	rfaL	2.629	0.537	1	0
9990	3909540	rfaK	2.101	0.539	0	0
16667	3909714	rfaK	2.033	0.594	0	0
12248	3909879	rfaK	2.042	0.657	0	0
8075	3910167	rfaK	2.417	0.575	1	0
17676	3910448	rfaK	2.509	0.487	1	0
2345	3910555	rfaK	2.160	0.484	0	0
12102	3910785	rfaK, rfaZ	1.893	0.495	0	0
2676	3911063	rfaK, rfaZ	2.188	0.402	0	0
8725	3911160	rfaZ	2.174	0.537	0	0
20004	3911426	rfaZ	2.172	0.567	0	0
554	3911793	rfaZ, rfaY	1.913	0.542	0	0
21382	3912004	rfaZ, rfaY	1.942	0.617	0	0
17513	3912251	rfaY	1.596	0.551	0	0
8687	3912527	rfaY, rfaJ	2.341	0.551	0	0
2698	3912743	rfaY, rfaJ	2.028	0.555	0	0
18677	3912834	rfaY, rfaJ	1.979	0.505	0	0
17653	3913007	rfaJ	2.062	0.594	0	0
6839	3913211	rfaJ	1.790	0.547	0	0
20122	3913599	rfaJ, rfaI	2.409	0.417	1	0
19616	3913887	rfaJ, rfaI	2.534	0.454	1	0
2408	3914050	rfaI	1.835	0.609	0	0
19873	3914289	rfaI	2.098	0.515	0	0
20805	3914378	rfaI	1.966	0.587	0	0
12232	3914490	rfaI	1.814	0.510	0	0
8796	3914814	rfaI, rfaB	2.537	0.625	0	0

14293	3915136	rfaB	2.961	0.561	1	0
15540	3915351	rfaB	2.776	0.520	1	0
1148	3915484	rfaB	2.794	0.468	1	0
10417	3915788	rfaB, yibR	2.503	0.493	1	0
4596	3916045	rfaB, yibR	3.816	0.659	1	0
16545	3916176	yibR, rfaP	4.260	0.765	1	0
18120	3916527	rfaP	1.820	0.673	0	0
18195	3916644	rfaP	1.238	0.568	0	0
9749	3916753	rfaP, rfaG	1.543	0.736	0	0
15999	3917290	rfaG	1.507	0.767	0	0
18511	3917390	rfaG	1.766	0.844	0	0
8652	3917705	rfaG	2.229	0.869	0	0
2187	3917787	rfaG	1.999	0.925	0	0
8054	3917919	rfaG, rfaQ	1.810	0.783	0	0
11360	3918371	rfaQ	0.910	0.696	0	0
17034	3918633	rfaQ	0.944	0.867	0	0
2902	3918768	rfaQ	1.008	0.810	0	0
2871	3918899	rfaQ, kdtA	1.094	0.894	0	0
11074	3919336	kdtA	1.087	1.149	0	0
2807	3919528	kdtA	0.746	1.517	0	0
1490	3919681	kdtA	0.815	1.444	0	0
20688	3919919	kdtA	0.845	1.398	0	0
17427	3920166	kdtA, kdtB	0.773	1.195	0	0
3106	3920458	kdtA, kdtB	0.785	1.774	0	1
12203	3920634	kdtB	0.871	2.094	0	1
22112	3920906	kdtB	0.745	2.157	0	1
19080	3921055	kdtB	0.737	2.299	0	1
14069	3921278	mutM	0.881	2.137	0	1
9468	3921483	mutM	0.942	1.544	0	0
3280	3921763	mutM	0.913	1.438	0	0
14337	3922044	mutM, rpmG	0.623	1.738	0	0
438	3922146	mutM, rpmG	0.496	2.115	0	0
8067	3922436	rpmG, rpmB	0.558	2.982	0	1
7340	3922721	rpmB, radC	0.578	2.555	0	1
14913	3922855	rpmB, radC	0.719	2.422	0	1
14800	3923178	radC, dfp	0.781	1.293	0	0

13841	3923449	radC, dfp	0.677	1.188	0	0
11018	3923655	radC, dfp	0.513	0.907	0	0
5397	3923796	dfp	0.709	1.064	0	0
8909	3924121	dfp	0.992	1.241	0	0
322	3924249	dfp, dut	1.094	1.443	0	0
17041	3924535	dfp, dut	0.801	1.243	0	0
1764	3924676	dfp, dut	0.837	1.366	0	0
13121	3924940	dut, ttk	0.755	1.489	0	0
4564	3925152	ttk	0.798	1.920	0	1
14096	3925451	ttk	0.750	1.780	0	1
14186	3925623	ttk	0.702	1.736	0	1
20359	3925695	ttk	0.832	2.189	0	1
5269	3926001	pyrE	0.788	2.012	0	1
13173	3926278	pyrE	0.776	1.401	0	0
9032	3926584	pyrE	1.082	1.125	0	0
7838	3926816	pyrE, rph	1.004	1.339	0	0
246	3927034	pyrE, rph	0.831	1.582	0	0
3930	3927278	rph, yicC	0.884	1.765	0	0
2448	3927409	rph, yicC	0.890	1.544	0	0
13854	3927481	rph, yicC	0.902	1.489	0	0
6553	3927598	rph, yicC	1.007	1.473	0	0
6924	3928165	yicC	1.188	1.264	0	0
577	3928289	yicC	1.389	1.342	0	0
12771	3928360		1.154	1.105	0	0
4647	3928710	STM3736	1.000	0.879	0	0
6811	3929050	STM3736, STM3737	1.060	0.738	0	0
11010	3929285	STM3736, STM3737	1.182	0.577	0	0
19537	3929553	STM3736, STM3737	1.234	0.677	0	0
11423	3929627	STM3736, STM3737	1.528	0.682	0	0
14044	3929887	STM3737	1.350	0.769	0	0
16822	3930054	STM3737	1.687	0.785	0	0
2888	3930323		1.636	0.800	0	0
8263	3930519		1.615	0.795	0	0
17402	3930790		0.921	0.989	0	0
19939	3931040		1.513	1.323	0	0
5759	3931130	yicF	1.332	1.193	0	0

5710	3931460	yicF	1.429	1.293	0	0
1461	3931772	yicF	1.301	0.947	0	0
18691	3932068	yicF	1.638	0.953	0	0
4274	3932253	yicF	1.170	0.825	0	0
16761	3932390	yicF	0.793	0.529	0	0
21057	3932656	yicF, gmk	0.993	0.798	0	0
17709	3932793	yicF, gmk	0.829	0.905	0	0
16740	3933053	yicF, gmk	1.100	1.027	0	0
4226	3933158	yicF, gmk	0.997	1.038	0	0
5321	3933244	yicF, gmk	0.548	0.587	0	0
21037	3933684	gmk, rpoZ	0.657	1.825	0	0
9003	3934043	spoT	0.605	2.166	0	1
9162	3934241	spoT	0.630	2.060	0	1
19639	3934583	spoT	0.708	1.696	0	1
5357	3934689	spoT	0.840	1.901	0	1
10840	3934940	spoT	0.737	1.664	0	0
21206	3935162	spoT	0.902	2.060	0	1
9773	3935459	spoT	1.002	2.095	0	1
247	3935572	spoT	0.886	2.140	0	1
20106	3935919	spoT, spoU	0.853	1.731	0	1
1216	3936020	spoT, spoU	0.938	1.790	0	0
2878	3936138	spoT, spoU	0.663	1.323	0	0
7709	3936522	spoU, recG	0.908	1.921	0	0
11853	3936744	spoU, recG	0.828	1.739	0	0
15434	3937068	recG	0.926	1.701	0	0
18003	3937142	recG	1.274	2.113	0	0
275	3937290	recG	0.723	1.226	0	0
9291	3937630	recG	0.793	1.190	0	0
5580	3937868	recG	0.881	1.358	0	0
17547	3938105	recG	0.555	0.909	0	0
11716	3938243	recG	1.057	1.607	0	0
2688	3938526	recG	1.074	1.229	0	0
18892	3938706	recG	1.204	1.494	0	0
4743	3939108	STM3745	1.346	1.356	0	0
16581	3939211	STM3745	1.319	1.306	0	0
6582	3939583	STM3745	1.175	1.239	0	0

11660	3939827	STM3745, gltS	0.812	0.845	0	0
17567	3939905	STM3745, gltS	1.002	1.155	0	0
1777	3940157	STM3745, gltS	0.786	0.995	0	0
8797	3940335	gltS	1.012	1.154	0	0
11137	3940685	gltS	0.837	0.927	0	0
7263	3940913	gltS, yicE	0.677	0.890	0	0
3677	3940993	gltS, yicE	0.657	1.109	0	0
1817	3941175	gltS, yicE	0.678	1.176	0	0
9698	3941311	gltS, yicE	0.795	1.446	0	0
12697	3941498	yicE	0.797	1.061	0	0
3942	3942045	yicE	1.122	0.960	0	0
10810	3942207	yicE	1.307	0.991	0	0
9176	3942324	yicE, yicH	1.094	0.814	0	0
1417	3942735	yicH	0.956	1.101	0	0
3292	3942873	yicH	1.091	1.209	0	0
18535	3942947	yicH	0.824	0.862	0	0
14754	3943409	yicH	0.916	0.787	0	0
10438	3943595	yicH	1.259	1.106	0	0
12463	3943666	yicH	1.040	1.115	0	0
16095	3944121	yicH	0.952	1.002	0	0
18814	3944363	yicH	1.075	1.241	0	0
14314	3944566	yicI	1.308	1.556	0	0
19387	3944638	yicI	1.387	1.647	0	0
16149	3944887	yicI	1.068	0.804	0	0
21056	3945185	yicI	1.258	0.941	0	0
3886	3945342	yicI	1.079	0.784	0	0
6010	3945526	yicI	1.323	0.653	0	0
6790	3945755	yicI	0.858	0.586	0	0
1847	3945931	yicI	1.697	0.740	0	0
16481	3946168	yicI	1.360	0.770	0	0
20167	3946458	yicI	1.206	0.676	0	0
17517	3946611	yicI	1.284	0.772	0	0
1237	3946889	yicI, yicJ	1.263	0.660	0	0
11901	3946963	yicI, yicJ	0.845	0.581	0	0
11732	3947380	yicJ	1.239	0.602	0	0
3458	3947753	yicJ	1.189	0.780	0	0

4223	3947908	yicJ	1.046	0.584	0	0
18795	3948137	yicJ, selC	1.237	0.736	0	0
7232	3948267	yicJ, selC	0.977	0.622	0	0
21118	3948352	yicJ, selC	1.578	0.889	0	0
22063	3948905		2.629	0.770	1	0
13461	3949070	STM3752	2.521	0.633	1	0
2228	3949344	STM3752	2.672	0.407	1	0
8221	3949433	STM3752	2.579	0.355	0	0
14219	3949761	STM3752, sugR	1.463	0.500	0	0
11648	3949940	sugR	1.305	0.620	0	0
11092	3950000	sugR	1.176	0.630	0	0
865	3950143	sugR	1.122	0.751	0	0
6849	3950649	sugR	0.982	0.701	0	0
10858	3950788	sugR, STM3754	0.787	0.725	0	0
13911	3951074	sugR, STM3754	1.183	0.842	0	0
14447	3951211	STM3754	1.249	0.936	0	0
16187	3951551	rhuM	1.123	0.980	0	0
16474	3951635	rhuM	1.290	1.078	0	0
3075	3951864	rhuM	1.106	0.858	0	0
12344	3952285	rhuM	1.516	0.708	0	0
1218	3952421	rhuM	2.274	0.884	1	0
13082	3952748		2.948	0.699	1	0
20017	3952878		3.373	0.664	1	0
15879	3953165		3.016	0.510	1	0
14505	3953311		2.676	0.456	1	0
13511	3953448		3.004	0.390	1	0
3462	3953699	rmbA	2.214	0.439	0	0
6468	3954041	rmbA	2.982	0.395	1	0
4098	3954260	rmbA	3.310	0.490	1	0
13050	3954489	rmbA, misL	4.442	0.500	1	0
22178	3954768	misL	5.397	0.557	1	0
20240	3954926	misL	4.619	0.628	1	0
11480	3955186	misL	2.142	0.615	1	0
7646	3955389	misL	1.880	0.609	0	0
17478	3955649	misL	1.604	0.658	0	0
9991	3955833	misL	1.401	0.767	0	0

15060	3956012	misL	1.440	0.627	0	0
2787	3956375	misL	1.349	0.663	0	0
13450	3956579	misL	1.022	0.588	0	0
8574	3956706	misL	1.286	0.750	0	0
9756	3957066	misL	1.174	0.659	0	0
8172	3957143	misL	1.477	0.798	0	0
18609	3957471	misL	1.399	0.615	0	0
22078	3957677		1.877	0.676	0	0
4095	3957817	fidL	1.593	0.615	0	0
4077	3958112	fidL	1.509	0.637	0	0
14761	3958297	fidL, marT	2.124	0.717	0	0
4920	3958450	fidL, marT	2.244	0.741	0	0
15642	3958736	marT	1.345	0.682	0	0
21451	3959067	marT	3.407	0.627	1	0
12620	3959313	marT, STM3760	3.546	0.384	1	0
921	3959546	STM3760, slsA	3.124	0.467	1	0
16828	3959810	slsA	2.516	0.585	0	0
17580	3959881	slsA	2.283	0.567	0	0
18884	3960106	slsA	1.745	0.595	0	0
1928	3960253	slsA	1.749	0.783	0	0
10822	3960469	slsA	1.616	0.846	0	0
14736	3960812	cigR	1.912	0.794	0	0
11629	3961066	cigR	2.162	0.740	0	0
14485	3961316	cigR	2.378	0.611	0	0
8589	3961495	cigR	3.324	0.656	1	0
22289	3961747	mgtB	3.092	0.691	1	0
7780	3961886	mgtB	3.954	0.610	1	0
11240	3962175	mgtB	3.394	0.699	1	0
6306	3962310	mgtB	2.754	0.705	1	0
9010	3962605	mgtB	1.510	0.735	0	0
5815	3962688	mgtB	1.608	0.779	0	0
9126	3963061	mgtB	1.233	0.940	0	0
3849	3963290	mgtB	0.944	0.830	0	0
18202	3963576	mgtB	1.455	0.776	0	0
15168	3963869	mgtB	2.307	0.688	1	0
7099	3963983	mgtB	2.911	0.574	1	0

21458	3964253	mgtB	4.838	0.647	1	0
11847	3964533	mgtB, mgtC	5.590	0.557	1	0
20974	3964636	mgtB, mgtC	6.134	0.633	1	0
14556	3964726	mgtC	5.221	0.607	1	0
6325	3965195	mgtC, yicL	3.740	0.650	1	0
17020	3965399	mgtC, yicL	4.507	0.706	1	0
9204	3965668	yicL	3.901	0.748	1	0
8900	3965875	yicL	2.006	0.714	0	0
8353	3966064	yicL	1.613	0.921	0	0
17843	3966264	yicL	1.207	0.824	0	0
21117	3966602		1.085	0.799	0	0
4530	3966814	STM3766	1.180	0.732	0	0
9907	3966885	STM3766	0.508	0.436	0	0
7997	3966956	STM3766	1.186	0.617	0	0
6680	3967319	STM3766	1.407	0.719	0	0
2383	3967598	STM3766	1.258	0.893	0	0
8084	3967797	STM3766, STM3767	1.105	0.709	0	0
16963	3967921	STM3766, STM3767	1.864	0.963	0	0
3322	3968448	STM3767	1.644	0.855	0	0
14395	3968532	STM3767, STM3768	1.864	0.857	0	0
8290	3968870	STM3767, STM3768	0.716	0.545	0	0
11253	3969121	STM3768	1.129	0.639	0	0
20999	3969303	STM3768	1.130	0.842	0	0
22248	3969599	STM3768	1.368	0.675	0	0
13831	3969792	STM3768, STM3769	1.067	0.671	0	0
11295	3969890	STM3768, STM3769	1.291	0.799	0	0
12300	3970227	STM3769	1.221	0.622	0	0
14733	3970298	STM3769	1.115	0.626	0	0
20364	3970478	STM3769	0.676	0.615	0	0
3967	3970708	STM3769, STM3770	1.256	0.636	0	0
21583	3971105	STM3770	1.561	0.964	0	0
7437	3971378	STM3770	1.386	0.720	0	0
9409	3971481	STM3770, STM3771	1.650	0.893	0	0
18049	3971744	STM3771	1.541	0.827	0	0
10446	3971873	STM3771	1.367	0.661	0	0
11513	3972297	STM3771, STM3772	0.869	0.645	0	0

12676	3972457	STM3772	1.325	1.098	0	0
21422	3972671	STM3772, STM3773	1.543	1.154	0	0
4954	3972812	STM3773	1.544	1.158	0	0
22196	3973094	STM3773	1.655	1.384	0	0
8150	3973383	STM3773	1.308	0.900	0	0
6341	3973475	STM3773	1.315	0.869	0	0
13980	3973761	STM3773	1.776	1.038	0	0
9692	3973980	STM3773	1.511	0.910	0	0
13681	3974320	STM3773	1.508	0.807	0	0
14963	3974419	STM3773	1.374	0.888	0	0
2195	3974623	STM3773	1.776	1.191	0	0
15938	3974694	STM3773	0.653	0.677	0	0
5361	3975163	STM3773, STM3774	1.242	1.153	0	0
10192	3975309	STM3773, STM3774	0.866	1.016	0	0
6887	3975523	STM3773, STM3774	1.151	1.078	0	0
10148	3975904	STM3775	1.215	0.900	0	0
2153	3976112	STM3775	1.218	0.966	0	0
3329	3976277	STM3775	1.244	0.933	0	0
3064	3976389	STM3775	1.289	0.858	0	0
3001	3976659	STM3775	1.660	0.884	0	0
2406	3977063	STM3775	1.400	0.886	0	0
8990	3977172	STM3775	1.456	0.758	0	0
7796	3977350		1.269	0.746	0	0
1005	3977734	yicM	1.585	0.803	0	0
3752	3977859	yicM	1.316	0.717	0	0
11173	3978100	yicM	1.182	0.768	0	0
5141	3978338	yicM, STM3777	1.491	0.801	0	0
19060	3978590	yicM, STM3777	1.105	0.617	0	0
5002	3978759	yicM, STM3777	1.088	0.681	0	0
6036	3978974	yicM, STM3777	1.077	0.642	0	0
21870	3979264	STM3778	1.541	0.678	0	0
6505	3979451		2.265	0.635	0	0
2426	3979603	STM3779	2.422	0.565	0	0
5121	3979771	STM3779	2.515	0.573	0	0
4767	3980106	STM3779, STM3780	2.941	0.502	1	0
17892	3980292	STM3780	3.264	0.587	1	0

1628	3980661	STM3780	4.015	0.474	1	0
21690	3980887	STM3780, STM3781	3.549	0.627	1	0
20412	3981088	STM3780, STM3781	2.456	0.663	1	0
4938	3981230	STM3781	1.888	0.669	0	0
19337	3981338	STM3781	1.846	0.708	0	0
19265	3981732	STM3781	1.179	0.483	0	0
2583	3981865	STM3781	1.364	0.739	0	0
4896	3982140	STM3781	1.528	0.538	0	0
2487	3982369	STM3781, STM3782	1.681	0.577	0	0
13982	3982576	STM3781, STM3782	2.242	0.644	0	0
9322	3982905	STM3782	1.592	0.581	0	0
6816	3982992	STM3782	1.513	0.592	0	0
13943	3983404	STM3782	2.914	0.577	1	0
11120	3983666	STM3782, STM3783	2.618	0.540	1	0
15774	3983748	STM3782, STM3783	2.627	0.573	1	0
15903	3983956	STM3782, STM3783	2.389	0.509	0	0
17796	3984286	STM3783, STM3784	2.757	0.570	1	0
17704	3984493	STM3784, STM3785	2.881	0.468	1	0
19014	3984772	STM3784, STM3785	2.254	0.542	0	0
15521	3984939	STM3785	1.752	0.670	0	0
8763	3985227	STM3785	0.887	0.591	0	0
17138	3985319	STM3785	0.900	0.791	0	0
5289	3985503	STM3785	0.944	0.808	0	0
14688	3985838	yicN	1.108	0.940	0	0
6649	3985990	yicN	0.984	0.703	0	0
22171	3986156	yicN, uhpT	1.074	0.805	0	0
6855	3986350	yicN, uhpT	1.288	0.818	0	0
17640	3986813	uhpT	1.119	0.634	0	0
10604	3986963	uhpT	1.254	0.694	0	0
15082	3987264	uhpT	1.311	0.681	0	0
15083	3987423	uhpT	1.204	0.612	0	0
20330	3987546	uhpT	1.265	0.705	0	0
12667	3987657	uhpT	1.283	0.843	0	0
20257	3988135	uhpC	1.312	0.855	0	0
17033	3988279	uhpC	1.826	1.047	0	0
12970	3988669	uhpC	0.976	0.705	0	0

13347	3988893	uhpC	2.005	1.119	0	0
1379	3989023	uhpC, uhpB	1.719	1.039	0	0
3957	3989259	uhpC, uhpB	1.495	1.029	0	0
8015	3989342	uhpC, uhpB	1.530	1.025	0	0
21366	3989773	uhpB	1.685	1.160	0	0
14491	3989931	uhpB	1.072	0.950	0	0
7682	3990104	uhpB	0.820	0.810	0	0
20652	3990268	uhpB	1.256	1.031	0	0
22118	3990495	uhpB	1.428	1.112	0	0
10968	3990580	uhpB, uhpA	1.795	1.221	0	0
22085	3991130	uhpA	1.170	0.916	0	0
21885	3991229	uhpA, STM3791	1.462	1.217	0	0
19628	3991438	uhpA, STM3791	1.611	1.012	0	0
21441	3991712	STM3791	1.434	1.006	0	0
521	3992067	STM3791	0.964	0.792	0	0
21771	3992190	STM3791	0.888	0.807	0	0
762	3992291	STM3791, STM3792	1.218	0.830	0	0
21801	3992581	STM3791, STM3792	1.440	0.772	0	0
3731	3992887	STM3792	0.878	0.596	0	0
21351	3993017	STM3792	1.845	0.689	0	0
3699	3993372	STM3792	2.667	0.600	0	0
16337	3993531	STM3792	2.116	0.614	0	0
13107	3993906	STM3792, STM3793	1.721	0.640	0	0
12625	3994043	STM3793	1.684	0.600	0	0
1998	3994255	STM3793	1.101	0.561	0	0
2017	3994483	STM3793, STM3794	0.692	0.559	0	0
18903	3994789	STM3793, STM3794	0.655	0.650	0	0
7057	3994860	STM3793, STM3794	0.682	0.755	0	0
18143	3995107	STM3794	0.798	0.645	0	0
20816	3995184	STM3794	0.907	0.675	0	0
6456	3995688	ilvN	0.933	0.726	0	0
10839	3995877	ilvN	0.919	0.904	0	0
2588	3996147	ilvN, ilvB	0.990	0.746	0	0
7219	3996238	ilvN, ilvB	1.106	0.711	0	0
17642	3996492	ilvB	1.412	0.801	0	0
21538	3996588	ilvB	1.265	0.722	0	0

20536	3996742	ilvB	1.278	0.768	0	0
20407	3997134	ilvB	0.851	0.998	0	0
4756	3997436	ilvB	0.904	1.970	0	1
6407	3997565	ilvB	0.549	2.107	0	1
13148	3997798	ilvB, ivbL	0.578	2.407	0	1
2295	3998133	ivbL, ysdB	0.729	1.427	0	0
18931	3998440	ysdB, STM3796A	0.861	0.946	0	0
2539	3998571	STM3796A	0.761	0.809	0	0
9184	3998865	STM3796A	0.962	0.961	0	0
4671	3999059	STM3796A	0.438	0.381	0	0
1464	3999163	STM3796A	1.098	0.948	0	0
18897	3999573	emrD	0.399	0.572	0	0
3845	3999662	emrD	1.385	1.074	0	0
21879	3999928	emrD	1.335	1.982	0	0
18731	4000054	emrD	1.002	1.428	0	0
13896	4000380	emrD	0.691	0.979	0	0
4260	4000477	emrD	0.907	1.301	0	0
8703	4000759	emrD	0.847	1.270	0	0
10017	4000964	STM3799	1.228	1.847	0	0
19480	4001374	STM3799	1.374	1.466	0	0
7433	4001567	STM3799	1.215	1.311	0	0
13203	4001832	STM3799	1.150	1.247	0	0
9653	4002016	STM3799, dsdC	1.009	0.986	0	0
10825	4002258	STM3799, dsdC	0.969	0.864	0	0
3603	4002429	dsdC	1.243	0.893	0	0
13122	4002666	dsdC, dsdX	1.148	0.881	0	0
8564	4002865	dsdC, dsdX	1.165	1.018	0	0
17321	4003033	dsdC, dsdX	0.740	0.739	0	0
6226	4003130	dsdC, dsdX	0.956	1.132	0	0
21123	4003466	dsdX	0.833	1.352	0	0
446	4003882	dsdX	0.838	1.669	0	0
5052	4003973	dsdX, dsdA	0.708	1.147	0	0
17786	4004342	dsdX, dsdA	0.705	1.512	0	0
2685	4004438	dsdA	0.704	1.488	0	0
21584	4004715	dsdA	0.945	1.802	0	0
9158	4004873	dsdA	0.982	2.047	0	0

7763	4005129	dsdA	0.870	1.920	0	0
18949	4005392	dsdA	0.734	2.029	0	0
1363	4005508	dsdA	0.877	2.361	0	0
5875	4005726	dsdA	0.802	2.378	0	0
5911	4006130	yidF	1.051	1.561	0	0
14958	4006374	yidF, yidG	0.991	1.234	0	0
13784	4006488	yidF, yidG	1.106	1.255	0	0
13652	4006779	yidF, yidG	0.922	1.113	0	0
4378	4007068	yidG, yidH	1.065	0.919	0	0
11416	4007168	yidG, yidH	1.110	0.910	0	0
21731	4007390	yidH	1.354	0.813	0	0
13589	4007745	yidE	1.340	0.999	0	0
16799	4007910	yidE	0.892	0.732	0	0
5848	4008219	yidE	1.101	0.802	0	0
12807	4008342	yidE	1.075	0.821	0	0
269	4008515	yidE	1.264	0.855	0	0
14064	4008836	yidE	1.075	1.015	0	0
10116	4008966	yidE	1.157	0.884	0	0
12484	4009155	yidE	0.805	0.948	0	0
15065	4009227	yidE	0.959	1.106	0	0
20988	4009812	ibpB	0.704	2.036	0	0
2285	4009883	ibpB	0.594	1.743	0	0
11100	4010229	ibpB, ibpA	0.758	2.268	0	1
587	4010326	ibpA, yidQ	0.858	2.243	0	1
3922	4010549	ibpA, yidQ	0.836	1.388	0	0
13677	4010648	ibpA, yidQ	0.909	1.438	0	0
15154	4010875	yidQ	1.066	1.541	0	0
18328	4011340	yidR	1.475	1.286	0	0
4233	4011458	yidR	1.294	0.992	0	0
17829	4011722	yidR	1.622	1.097	0	0
11663	4011923	yidR	1.428	0.985	0	0
15205	4012183	yidR	1.934	1.176	0	0
21424	4012424	yidR, ccmH	1.285	0.931	0	0
16774	4012597	yidR, ccmH	2.514	1.191	0	0
8087	4012829	ccmH	0.582	0.690	0	0
20207	4013106	ccmH	1.144	0.812	0	0

8720	4013375	ccmH, ccmG	1.413	0.737	0	0
14299	4013600	ccmH, ccmG	0.783	0.626	0	0
12171	4013879	ccmG	1.658	0.984	0	0
5492	4013978	ccmG, ccmF	1.292	0.854	0	0
253	4014301	ccmG, ccmF	1.037	0.756	0	0
21167	4014499	ccmF	1.735	0.904	0	0
7735	4014640	ccmF	1.290	0.753	0	0
18279	4014851	ccmF	0.675	0.453	0	0
5451	4015094	ccmF	0.881	0.712	0	0
10426	4015276	ccmF	0.412	0.583	0	0
913	4015628	ccmF	2.985	1.087	0	0
22285	4015880	ccmF, ccmE	1.547	0.938	0	0
19227	4016025	ccmF, ccmE	1.174	0.944	0	0
1969	4016167	ccmF, ccmE	1.306	0.772	0	0
18957	4016575	ccmE, ccmD	2.771	1.043	0	0
2361	4016726	ccmE, ccmD	1.307	0.852	0	0
281	4017052	ccmC	2.267	0.973	0	0
9812	4017241	ccmC	1.131	0.664	0	0
2877	4017383	ccmC, ccmB	1.006	0.761	0	0
20550	4017595	ccmC, ccmB	0.766	0.685	0	0
13012	4017862	ccmB	1.579	0.761	0	0
7989	4018095	ccmB, ccmA	1.458	0.709	0	0
3567	4018329	ccmB, ccmA	1.103	0.682	0	0
8523	4018580	ccmA, STM3820	5.886	0.685	1	0
21470	4018792	ccmA, STM3820	8.078	0.750	1	0
14757	4018997	ccmA, STM3820	6.410	0.617	1	0
1226	4019159	STM3820	6.332	0.590	1	0
16919	4019404	STM3820	5.107	0.650	1	0
15314	4019788	STM3820	4.734	0.577	1	0
6539	4019916	STM3820	4.225	0.473	1	0
774	4020170	STM3820, torD	4.595	0.547	1	0
10736	4020260	STM3820, torD	5.260	0.520	1	0
22010	4020346	STM3820, torD	5.676	0.570	1	0
17760	4020709	torD	2.200	0.741	0	0
17887	4020956	torD, torA	1.648	0.641	0	0
6989	4021363	torA	1.418	0.621	0	0

5699	4021486	torA	1.345	0.614	0	0
13125	4021665	torA	1.233	0.656	0	0
4255	4021983	torA	1.247	0.594	0	0
12020	4022202	torA	0.689	0.601	0	0
16250	4022372	torA	1.238	0.634	0	0
12526	4022663	torA	1.140	0.705	0	0
20511	4022897	torA	1.529	0.877	0	0
18651	4023102	torA	1.425	0.738	0	0
9155	4023391	torA, torC	1.138	0.518	0	0
13528	4023579	torA, torC	1.366	0.695	0	0
17123	4023790	torA, torC	0.689	0.569	0	0
808	4024091	torC	1.364	0.697	0	0
9747	4024187	torC, torR	1.248	0.718	0	0
10318	4024503	torC, torR	1.206	1.024	0	0
20408	4024661	torC, torR	1.210	1.226	0	0
19815	4024785	torC, torR	1.010	1.151	0	0
15077	4025047	torR	1.207	1.671	0	0
3021	4025454	torT	0.927	1.653	0	0
6027	4025570	torT	1.612	1.408	0	0
20663	4025685	torT	1.102	1.302	0	0
3952	4026091	torT, torS	1.250	0.887	0	0
20764	4026178	torT, torS	1.467	0.960	0	0
14285	4026307	torT, torS	1.646	0.902	0	0
2567	4026693	torT, torS	1.246	0.821	0	0
8514	4027024	torS	1.141	0.729	0	0
10087	4027128	torS	1.038	0.771	0	0
12548	4027309	torS	1.140	0.804	0	0
6264	4027442	torS	1.049	0.796	0	0
15062	4027865	torS	1.421	0.833	0	0
12048	4027938	torS	1.249	0.748	0	0
19313	4028447	torS	1.228	0.853	0	0
11103	4028679	torS	1.273	1.069	0	0
13037	4028781	torS	1.318	1.109	0	0
1911	4029073	torS	1.420	0.922	0	0
17617	4029319	dgoT	1.361	0.902	0	0
5513	4029495	dgoT	1.462	0.875	0	0

13378	4029660	dgoT	1.609	0.796	0	0
20098	4029929	dgoT	1.580	0.780	0	0
2886	4030226	dgoT	1.169	0.628	0	0
14949	4030338	dgoT	1.678	0.654	0	0
10149	4030545	dgoT	1.250	0.744	0	0
19402	4030831	dgoT, dgoA	1.771	0.646	0	0
20430	4031145	dgoA	0.995	0.552	0	0
14245	4031313	dgoA	1.431	0.716	0	0
7652	4031578	dgoA	1.177	0.554	0	0
21682	4031746	dgoA	1.325	0.778	0	0
5669	4031964	dgoA	1.364	0.728	0	0
17528	4032243		1.959	0.688	0	0
4782	4032357	dgoK	1.656	0.758	0	0
17371	4032759	dgoK	1.391	0.701	0	0
22244	4032910	dgoK	1.140	0.755	0	0
19946	4033213	dgoK	1.179	0.684	0	0
10625	4033448	dgoK, dgoR	0.987	0.632	0	0
10253	4033672	dgoK, dgoR	1.137	0.562	0	0
16989	4033908	dgoR	0.975	0.581	0	0
10308	4034061	dgoR	0.843	0.604	0	0
19708	4034270	dgoR, yidA	0.941	0.860	0	0
8745	4034579	yidA	0.993	0.670	0	0
14483	4034701	yidA	1.089	0.676	0	0
21119	4034841	yidA	1.311	0.624	0	0
21367	4035014	yidA, STM3832	2.448	0.675	0	0
3691	4035089	yidA, STM3832	2.054	0.686	0	0
17228	4035402	yidA, STM3832	2.639	0.570	0	0
16576	4035915	STM3832	2.915	0.584	1	0
13986	4036078	STM3832	3.215	0.552	1	0
22068	4036331	STM3832, STM3833	4.487	0.618	1	0
11451	4036472	STM3832, STM3833	5.166	0.664	1	0
19010	4036828	STM3833	3.239	0.677	1	0
4011	4036976	STM3833	2.571	0.746	1	0
9285	4037170	STM3833	1.308	0.667	0	0
13555	4037529	STM3833, STM3834	1.517	0.696	0	0
16375	4037695	STM3833, STM3834	1.195	0.761	0	0

3328	4037781	STM3833, STM3834	0.844	0.715	0	0
10726	4037897	STM3834	1.073	0.897	0	0
10336	4038032	STM3834	1.228	1.070	0	0
20296	4038293	STM3834	0.901	1.004	0	0
13795	4038841	gyrB	1.089	1.752	0	0
4157	4039032	gyrB	0.753	1.267	0	0
15518	4039293	gyrB	0.789	1.207	0	0
4898	4039595	gyrB	0.827	1.210	0	0
560	4039682	gyrB	1.193	1.840	0	0
17285	4039903	gyrB	1.072	1.736	0	0
18577	4040229	gyrB	0.941	1.404	0	0
16877	4040444	gyrB	0.814	1.112	0	0
2201	4040525	gyrB	1.147	1.117	0	0
21941	4040835	gyrB	1.023	1.633	0	0
14294	4041092	gyrB	0.816	1.898	0	1
3126	4041221	gyrB	0.849	2.006	0	1
340	4041313	gyrB, recF	1.131	2.340	0	1
10598	4041708	gyrB, recF	0.969	1.903	0	1
13417	4041926	recF	0.998	2.403	0	1
8584	4042094	recF	0.714	1.629	0	1
10836	4042485	recF	0.900	1.782	0	1
5315	4042715	recF, dnaN	0.934	1.444	0	0
2026	4042875	dnaN	0.988	1.483	0	0
6237	4043104	dnaN	1.328	1.539	0	0
12292	4043211	dnaN	0.795	1.115	0	0
9434	4043571	dnaN	0.748	1.197	0	0
6181	4043657	dnaN, dnaA	0.738	1.207	0	0
16994	4043815	dnaN, dnaA	0.924	1.264	0	0
5766	4044307	dnaA	0.726	1.096	0	0
18006	4044578	dnaA	0.638	1.369	0	0
4265	4044823	dnaA	0.469	1.406	0	0
14143	4044938	dnaA	0.557	2.176	0	1
5216	4045027	dnaA	0.496	2.323	0	1
10194	4045240	dnaA, rpmH	0.444	2.474	0	1
5435	4045632	rpmH, rnpA	0.462	3.435	0	1
21846	4045820	rnpA, STM3841	0.536	3.371	0	1

15877	4046107	rnpA, STM3841	0.523	2.770	0	1
14087	4046274	STM3841, yidC	0.548	2.326	0	1
16597	4046393	yidC	0.544	2.806	0	1
17361	4046598	yidC	0.571	1.987	0	1
8765	4046976	yidC	0.660	1.762	0	1
6910	4047113	yidC	0.790	1.883	0	1
3148	4047487	yidC	0.718	1.802	0	0
13930	4047699	yidC	0.734	1.801	0	1
12757	4047807	yidC, trmE	0.717	1.819	0	1
1154	4048178	trmE	0.738	0.996	0	0
6151	4048448	trmE	0.738	0.866	0	0
20918	4048678	trmE	0.999	0.971	0	0
19257	4048755	trmE	0.921	0.897	0	0
7009	4048968	trmE	0.867	0.834	0	0
1304	4049303	trmE	0.940	1.019	0	0
18557	4049543	trmE	0.713	0.788	0	0
4976	4049689		0.564	0.700	0	0
1150	4049813		0.825	0.661	0	0
12210	4050077		0.766	0.694	0	0
18979	4050395		0.854	0.633	0	0
20835	4050543		1.043	0.827	0	0
19042	4050783		1.144	0.761	0	0
20220	4050981	STM3845	1.467	0.770	0	0
4640	4051189	STM3845	1.190	0.607	0	0
9505	4051571	STM3845	2.031	0.576	0	0
15216	4051741	STM3845	2.118	0.591	0	0
20520	4051919	STM3845, STM3846	1.936	0.507	0	0
9992	4052232	STM3845, STM3846	1.835	0.509	0	0
6213	4052528	STM3846	1.915	0.563	0	0
6564	4052608	STM3846	1.915	0.492	0	0
18086	4052979	STM3846	2.058	0.468	0	0
13860	4053108	STM3846	2.288	0.408	0	0
3083	4053292		1.992	0.414	0	0
5244	4053477		1.302	0.501	0	0
14369	4053813	yidY	1.166	0.595	0	0
16355	4054072	yidY	1.023	0.722	0	0

10265	4054348	yidY	0.887	0.843	0	0
7733	4054546	yidY	0.762	0.771	0	0
14448	4054712	yidY	0.661	0.609	0	0
20417	4054929	yidY, yidZ	0.932	0.721	0	0
13828	4055147	yidY, yidZ	1.058	0.859	0	0
17447	4055239	yidZ	1.213	0.914	0	0
4162	4055560	yidZ	1.212	1.067	0	0
19353	4055738	yidZ	0.796	0.850	0	0
17353	4056116	yidZ, yieE	1.115	0.982	0	0
20031	4056198	yieE	1.275	0.962	0	0
7386	4056372	yieE	1.296	0.838	0	0
2294	4056558	yieE	1.423	0.882	0	0
17942	4056707	yieE, yieF	1.164	0.869	0	0
11265	4057121	yieF	1.241	0.945	0	0
13855	4057503	yieF	1.079	1.120	0	0
4257	4057716	yieG	1.064	1.100	0	0
11276	4057887	yieG	1.179	1.302	0	0
19386	4058120	yieG	1.056	1.003	0	0
12811	4058250	yieG	1.265	1.107	0	0
1481	4058643	yieG	1.109	1.099	0	0
17693	4058909	yieG, yieH	1.107	1.325	0	0
17434	4059136	yieG, yieH	0.821	1.121	0	0
3199	4059218	yieG, yieH	0.764	0.926	0	0
16078	4059344	yieG, yieH	0.930	1.122	0	0
21884	4059596	yieG, yieH	0.911	1.402	0	0
16325	4059773	yieH	0.865	1.966	0	1
17666	4060072	phoU	0.721	2.426	0	1
8405	4060203	phoU	0.689	2.570	0	1
13878	4060679	phoU	0.662	2.248	0	1
21948	4060796	phoU, pstB	0.773	2.571	0	1
15819	4061049	phoU, pstB	0.614	2.386	0	1
9100	4061378	pstB	0.870	2.150	0	1
2775	4061472	pstB	0.682	1.779	0	1
21893	4061588	pstB, pstA	0.855	2.202	0	1
1730	4061848	pstB, pstA	0.753	2.417	0	1
12423	4062102	pstA	0.825	2.419	0	1

19992	4062514	pstA, pstC	0.821	2.404	0	1
3802	4062679	pstA, pstC	0.708	2.273	0	1
3638	4062805	pstA, pstC	0.652	2.194	0	0
18078	4062978	pstC	0.880	2.744	0	1
1221	4063296	pstC	0.905	2.553	0	1
8509	4063426	pstC	0.644	1.580	0	0
14304	4063700	pstC, pstS	0.621	2.691	0	1
14101	4064071	pstS	0.892	2.755	0	1
17809	4064201	pstS	0.896	2.952	0	1
1329	4064433	pstS	0.705	2.343	0	1
12613	4064601	pstS	0.805	1.995	0	1
1903	4064672	pstS	1.071	2.100	0	1
16534	4065189	STM3858	0.925	1.017	0	0
9928	4065280	STM3858	1.262	1.190	0	0
2246	4065686	STM3858	1.285	0.875	0	0
4553	4065914	STM3858	1.288	0.831	0	0
11292	4066143	STM3858	1.607	0.716	0	0
3657	4066260	STM3858, STM3859	1.255	0.838	0	0
14291	4066385	STM3858, STM3859	1.250	0.666	0	0
4452	4066715	STM3859, STM3860	1.289	0.646	0	0
18347	4067022	STM3859, STM3860	1.318	0.534	0	0
17972	4067143	STM3859, STM3860	1.213	0.757	0	0
21438	4067240	STM3859, STM3860	1.463	0.805	0	0
6492	4067343	STM3859, STM3860	1.326	0.713	0	0
6098	4067935	STM3860	1.384	0.797	0	0
4536	4068162	STM3860	1.335	0.936	0	0
17655	4068437	STM3860	1.269	1.027	0	0
5325	4068508	STM3860	1.397	1.107	0	0
10056	4068829	STM3860	1.086	1.641	0	1
17741	4069007	glmS	0.791	1.770	0	1
19356	4069147	glmS	0.719	1.615	0	0
20660	4069550	glmS	0.861	2.549	0	1
17196	4069621	glmS	0.810	2.359	0	1
14048	4069900	glmS	0.742	1.828	0	1
19101	4070142	glmS	0.865	1.908	0	1
19013	4070450	glmS	0.709	2.287	0	1

12520	4070546	glmS	0.637	2.193	0	1
1235	4070771	glmS	0.875	2.356	0	1
21008	4070935	glmS	0.769	1.612	0	1
14569	4071217	glmS, glmU	0.742	1.756	0	1
2522	4071561	glmU	0.760	1.811	0	1
7603	4071786	glmU	0.667	1.589	0	0
19448	4071908	glmU	0.638	1.610	0	0
3805	4072183	glmU	0.709	1.623	0	0
12030	4072458	glmU	0.997	1.574	0	0
15298	4072757	glmU, STM3863	0.890	0.881	0	0
1647	4072843	glmU, STM3863	1.016	1.034	0	0
18870	4073122	STM3863	0.927	0.882	0	0
19546	4073448	STM3863	0.756	1.172	0	0
11237	4073573	STM3863	0.852	1.492	0	0
4354	4073893	atpC	0.386	1.236	0	0
7020	4074076	atpC	0.478	1.848	0	1
10259	4074186	atpC, atpD	0.586	2.107	0	1
12133	4074319	atpC, atpD	0.584	2.362	0	1
20219	4074749	atpD	0.590	2.623	0	1
13707	4074874	atpD	0.580	2.863	0	1
20195	4075088	atpD	0.500	2.446	0	1
15649	4075459	atpD	0.559	2.673	0	1
11359	4075538	atpD, atpG	0.369	1.254	0	0
1897	4075836	atpD, atpG	0.522	2.314	0	1
12239	4075913	atpD, atpG	0.506	2.414	0	1
9503	4076173	atpG	0.462	2.019	0	0
4154	4076442	atpG, atpA	0.466	2.326	0	1
10341	4076574	atpG, atpA	0.498	2.292	0	1
19505	4076889	atpA	0.583	2.424	0	1
12646	4076999	atpA	0.490	2.378	0	1
5382	4077147	atpA	0.412	1.805	0	0
4155	4077683	atpA	0.466	2.624	0	1
8628	4077871	atpA	0.537	2.156	0	1
6785	4078075	atpA, atpH	0.465	2.241	0	1
448	4078268	atpA, atpH	0.365	2.176	0	1
15797	4078499	atpH	0.407	2.350	0	1

14941	4078699	atpH, atpF	0.419	2.841	0	1
6375	4078979	atpF	0.375	2.530	0	1
7026	4079269	atpF, atpE	0.397	2.494	0	1
15002	4079546	atpE, atpB	0.466	2.378	0	1
6179	4079792	atpB	0.495	2.969	0	1
21354	4079874	atpB	0.463	2.653	0	1
16408	4080083	atpB	0.447	2.876	0	1
16482	4080457	atpB, atpI	0.589	2.253	0	1
6463	4080528	atpB, atpI	0.580	1.792	0	1
10044	4080849	atpI	0.788	1.007	0	0
15274	4080964	atpI	0.851	0.821	0	0
19891	4081229	gidB	0.690	0.960	0	0
12179	4081431	gidB	0.783	1.099	0	0
8830	4081760	gidB	0.634	0.973	0	0
16838	4082005	gidB, gidA	0.868	1.223	0	0
14676	4082131	gidB, gidA	0.834	1.379	0	0
8569	4082465	gidA	0.798	1.361	0	0
452	4082664	gidA	0.845	1.235	0	0
16335	4082988	gidA	0.674	0.974	0	0
8028	4083067	gidA	0.719	1.047	0	0
13328	4083392	gidA	0.753	1.379	0	0
5933	4083647	gidA	0.645	1.387	0	0
13960	4083763	gidA	0.741	1.644	0	0
15647	4083970	gidA	0.519	1.805	0	0
11557	4084208	gidA, mioC	0.484	1.930	0	1
13471	4084586	mioC	0.531	1.976	0	1
11524	4084724	mioC, asnC	0.525	1.652	0	0
14521	4084821	mioC, asnC	0.484	1.115	0	0
17388	4085214	asnC, asnA	0.712	0.554	0	0
16228	4085312	asnC, asnA	0.813	0.696	0	0
7062	4085439	asnC, asnA	1.011	0.813	0	0
12932	4085713	asnA	0.912	0.736	0	0
12998	4086009	asnA	0.749	0.987	0	0
1272	4086332	yieM	1.102	1.360	0	0
3434	4086517	yieM	1.114	1.362	0	0
3744	4086622	yieM	1.188	1.459	0	0

11442	4086868	yieM	0.902	0.952	0	0
16915	4087150	yieM	1.086	1.084	0	0
15890	4087489	yieM	0.865	1.012	0	0
4032	4087638	yieM	0.643	0.627	0	0
4266	4087979	yieM, yieN	0.743	0.946	0	0
2186	4088109	yieM, yieN	1.009	1.358	0	0
19919	4088322	yieN	0.865	1.232	0	0
13132	4088604	yieN	1.057	1.174	0	0
14067	4088696	yieN	1.070	1.113	0	0
1442	4088982	yieN	1.100	1.007	0	0
12801	4089350	yieN, kup	0.876	1.006	0	0
6052	4089421	yieN, kup	0.787	0.904	0	0
543	4089753	kup	0.888	0.963	0	0
18952	4089892	kup	0.638	0.632	0	0
16953	4090007	kup	0.934	0.847	0	0
19647	4090169	kup	1.138	0.792	0	0
6312	4090606	kup	0.790	0.815	0	0
19509	4090721	kup	1.175	0.918	0	0
15164	4091041	kup	0.869	0.752	0	0
15208	4091264	kup, rbsD	0.751	0.855	0	0
6320	4091565	rbsD	0.842	0.961	0	0
11661	4091636	rbsD, rbsA	0.879	1.036	0	0
17403	4091927	rbsD, rbsA	0.443	0.562	0	0
11197	4092212	rbsA	1.022	0.623	0	0
17713	4092527	rbsA	1.106	0.700	0	0
14776	4092661	rbsA	1.167	0.776	0	0
14363	4092764	rbsA	1.152	0.746	0	0
12859	4093075	rbsA	1.245	0.862	0	0
17452	4093374	rbsA, rbsC	1.214	0.943	0	0
16655	4093676	rbsC	1.150	0.779	0	0
7227	4093780	rbsC	1.088	0.813	0	0
3160	4093981	rbsC	0.585	0.463	0	0
13393	4094224	rbsC, rbsB	0.482	0.507	0	0
18271	4094437	rbsC, rbsB	1.123	0.962	0	0
6762	4094564	rbsB	1.086	0.956	0	0
10330	4094661	rbsB	1.154	1.064	0	0

4326	4095114	rbsB, rbsK	0.346	0.778	0	0
11132	4095426	rbsB, rbsK	1.064	1.590	0	0
15243	4095619	rbsK	0.992	1.636	0	0
12208	4095799	rbsK	1.102	1.483	0	0
9612	4095904	rbsK	1.023	1.286	0	0
19976	4096239	rbsK, rbsR	1.080	1.464	0	0
8161	4096577	rbsR	0.959	1.332	0	0
22135	4096707	rbsR	0.655	1.103	0	0
16443	4097042	rbsR	0.925	1.282	0	0
14315	4097155	rbsR	0.538	0.876	0	0
13875	4097527	yieO	0.760	1.303	0	0
3868	4097635	yieO	0.896	1.404	0	0
16009	4097789	yieO	0.960	1.231	0	0
15032	4098131	yieO	1.026	0.920	0	0
9720	4098299	yieO	1.112	0.989	0	0
4269	4098391	yieO	0.523	0.641	0	0
11486	4098737	yieO	0.923	1.017	0	0
5384	4098910	yieO	0.878	1.004	0	0
8862	4099307	yieO, yieP	0.551	1.359	0	0
14000	4099595	yieP	0.467	2.667	0	1
4623	4099693	yieP, rrsC	0.451	3.130	0	1
2869	4099764	yieP, rrsC	0.417	3.227	0	1
1285	4100014	yieP, rrsC	0.500	4.395	0	1
20217	4100113	rrsC	0.388	3.946	0	1
19783	4100245	rrsC	0.400	4.250	0	1
8795	4100909	rrsC	0.343	5.944	0	1
7257	4101085	rrsC	0.343	6.306	0	1
17854	4101244	rrsC	0.334	5.453	0	1
4159	4101557	rrsC, gltU	0.322	5.202	0	1
6105	4101655	gltU, rrlC	0.341	4.357	0	1
21395	4101946	rrlC	0.373	4.175	0	1
1554	4102164	rrlC	0.431	4.505	0	1
2932	4102452	rrlC	0.438	5.639	0	1
15648	4102595	rrlC	0.371	5.098	0	1
5035	4102725	rrlC	0.383	5.470	0	1
13387	4103083	rrlC	0.379	6.034	0	1

14984	4103341	rrlC	0.368	5.710	0	1
10428	4103577	rrlC	0.337	4.039	0	1
2668	4103886	rrlC	0.329	4.822	0	1
3847	4104025	rrlC	0.335	5.164	0	1
12553	4104170	rrlC	0.364	4.672	0	1
2844	4104268	rrlC	0.355	4.292	0	1
10230	4104824	rrlC, 9S	0.376	4.896	0	1
5529	4104895	rrlC, 9S	0.390	4.620	0	1
19122	4105204	aspT, trpT	0.418	4.618	0	1
8560	4105420		0.359	2.868	0	1
18682	4105571	yifA	0.480	2.944	0	1
18345	4105869	yifA	0.573	1.627	0	0
21747	4106138	yifA, yifE	0.673	1.121	0	0
7937	4106338	yifA, yifE	0.877	1.313	0	0
15606	4106541	yifA, yifE	0.826	1.322	0	0
20025	4106622	yifA, yifE	0.808	1.302	0	0
5695	4106874	yifB	0.722	1.224	0	0
7819	4107275	yifB	0.864	1.056	0	0
4169	4107375	yifB	0.957	1.078	0	0
7494	4107461	yifB	0.589	0.747	0	0
13137	4107892	yifB	0.904	0.882	0	0
2066	4108137	yifB	0.795	0.677	0	0
651	4108264	yifB, ilvL	0.724	0.820	0	0
8580	4108523	yifB, ilvL	0.813	0.947	0	0
1084	4108889	ilvG	0.814	1.463	0	0
22288	4109048	ilvG	0.586	0.966	0	0
5894	4109290	ilvG	0.427	0.503	0	0
2947	4109476	ilvG	1.100	0.805	0	0
764	4109648	ilvG	0.835	0.681	0	0
3542	4109719	ilvG	1.091	0.686	0	0
14727	4110225	ilvG, ilvM	1.029	0.807	0	0
2381	4110304	ilvG, ilvM	0.967	0.805	0	0
13687	4110588	ilvG, ilvM	1.209	0.848	0	0
19360	4110887	ilvE	1.170	0.886	0	0
12867	4111012	ilvE	1.337	0.914	0	0
19657	4111084	ilvE	1.353	1.067	0	0

6477	4111451	ilvE	1.555	1.055	0	0
3642	4111741	ilvE, ilvD	1.693	1.160	0	0
21060	4111938	ilvD	1.582	1.002	0	0
1687	4112063	ilvD	1.470	1.113	0	0
13217	4112440	ilvD	1.318	0.959	0	0
17257	4112691	ilvD	1.334	0.954	0	0
14340	4112912	ilvD	1.385	0.899	0	0
18508	4113066	ilvD	1.478	0.961	0	0
8942	4113301	ilvD	1.155	0.755	0	0
16585	4113595	ilvD, ilvA	1.391	1.005	0	0
13350	4113809	ilvA	0.928	0.623	0	0
6227	4113938	ilvA	1.310	0.965	0	0
17304	4114236	ilvA	1.226	1.181	0	0
9281	4114583	ilvA	0.863	0.868	0	0
10274	4114723	ilvA	1.194	1.120	0	0
11145	4114922	ilvA	1.151	1.029	0	0
1323	4115045	ilvA	1.192	1.270	0	0
9618	4115413	STM3906	0.896	0.766	0	0
751	4115635	STM3906, STM3907	0.999	0.846	0	0
21999	4115860	STM3906, STM3907	1.034	0.712	0	0
12494	4116122	STM3907	1.112	0.710	0	0
14173	4116297	ilvY	1.108	0.711	0	0
20152	4116389	ilvY	1.210	0.875	0	0
3986	4116627	ilvY	0.818	0.623	0	0
9133	4116985	ilvY, ilvC	1.044	0.875	0	0
15870	4117287	ilvY, ilvC	1.249	0.789	0	0
12319	4117358	ilvY, ilvC	0.989	0.767	0	0
3476	4117633	ilvY, ilvC	1.125	0.897	0	0
7919	4117988	ilvC	1.209	1.013	0	0
21392	4118118	ilvC	1.219	0.946	0	0
8556	4118306	ilvC	0.589	0.497	0	0
4153	4118596	ilvC	1.027	1.111	0	0
13625	4118810	ilvC	0.931	1.395	0	0
5813	4118937	ppiC	1.104	1.210	0	0
2352	4119257	ppiC, STM3911	1.116	1.040	0	0
20439	4119412	ppiC, STM3911	1.194	0.993	0	0

12955	4119715	rep	0.991	1.108	0	0
3844	4119837	rep	1.015	1.059	0	0
3711	4120144	rep	1.191	0.954	0	0
6613	4120270	rep	1.017	0.898	0	0
13809	4120363	rep	0.723	0.572	0	0
16757	4120749	rep	1.247	0.920	0	0
987	4121034	rep	1.363	1.093	0	0
20982	4121253	rep	1.075	1.179	0	0
21115	4121415	rep	1.020	1.209	0	0
11619	4121544	rep	0.993	1.408	0	0
9172	4121924	gppA	0.804	1.639	0	0
21574	4122246	gppA	0.789	1.825	0	1
8696	4122463	gppA	0.672	2.338	0	1
2933	4122758	gppA	0.735	2.721	0	1
10876	4122979	gppA	0.334	0.583	0	0
12985	4123089	gppA	0.337	0.547	0	0
12390	4123393	gppA	0.533	0.606	0	0
8955	4123660	gppA, rhlB	0.487	0.957	0	0
19614	4123864	rhlB	0.253	0.318	0	0
4885	4123961	rhlB	0.992	0.538	0	0
5456	4124155	rhlB	0.684	2.262	0	1
11037	4124581	rhlB, trxA	0.521	2.320	0	1
18429	4124652	rhlB, trxA	0.488	1.819	0	1
16383	4124835	rhlB, trxA	0.536	2.299	0	1
6354	4125228	rho	0.399	1.990	0	1
21275	4125438	rho	0.392	2.386	0	1
5284	4125522	rho	0.480	2.429	0	1
959	4125899	rho	0.788	1.834	0	0
9348	4125997	rho	0.614	1.496	0	0
1134	4126331	rho	0.557	1.209	0	0
20342	4126537	rho	0.772	1.434	0	0
18576	4126793	rho, rfe	0.655	0.980	0	0
10431	4126918	rfe	0.615	0.748	0	0
14237	4127041	rfe	0.666	0.859	0	0
12275	4127324	rfe	0.707	0.868	0	0
17173	4127400	rfe	0.782	0.966	0	0

21529	4127987	rfe, wzzE	0.749	1.613	0	0
22346	4128144	rfe, wzzE	0.710	1.515	0	0
13439	4128449	wzzE	0.833	1.546	0	0
3628	4128532	wzzE	0.845	1.537	0	0
14151	4128850	wzzE, wecB	0.768	1.386	0	0
12905	4128985	wzzE, wecB	0.749	1.388	0	0
9500	4129262	wecB	0.715	1.534	0	0
20306	4129413	wecB	0.718	1.725	0	0
15509	4129750	wecB	0.810	1.490	0	0
17774	4129863	wecB	0.810	1.588	0	0
1408	4130073	wecB, wecC	0.758	1.408	0	0
16525	4130412	wecC	0.814	1.396	0	0
10052	4130508	wecC	0.999	1.680	0	0
8874	4130721	wecC	1.021	1.628	0	0
12786	4131042	wecC	1.130	1.600	0	0
1933	4131374	wecC, rffG	0.970	1.610	0	0
3532	4131607	wecC, rffG	0.913	1.746	0	1
20929	4131839	rffG	0.913	1.548	0	1
2889	4132069	rffG	0.821	1.241	0	0
10012	4132205	rffG	0.762	1.217	0	0
13780	4132436	rffG, rffH	0.802	1.215	0	0
6378	4132734	rffH, wecD	1.082	1.955	0	1
6343	4132930	rffH, wecD	0.983	2.225	0	1
11628	4133001	wecD	0.887	1.852	0	1
15203	4133237	wecD, wecE	0.917	1.755	0	1
20647	4133620	wecD, wecE	1.001	1.517	0	0
1398	4133874	wecE	0.805	1.356	0	0
4577	4133945	wecE	1.019	1.228	0	0
21326	4134154	wecE	1.015	1.312	0	0
17408	4134501	wecE, wzxE	1.329	1.552	0	0
11856	4134745	wecE, wzxE	1.132	1.684	0	0
11036	4134830	wzxE	0.847	1.388	0	0
9180	4135206	wzxE	1.068	1.857	0	0
9427	4135447	wzxE	0.910	1.160	0	0
2368	4135567	wzxE, STM3927	0.878	1.471	0	0
9674	4135870	wzxE, STM3927	1.044	1.355	0	0

1558	4135961	wzxE, STM3927	0.680	0.887	0	0
1258	4136251	STM3927	0.962	1.167	0	0
21138	4136374	STM3927	0.984	1.320	0	0
16304	4136763	STM3927, wecF	0.971	1.102	0	0
11503	4137030	STM3927, wecF	0.981	1.351	0	0
7898	4137222	wecF	0.838	1.069	0	0
14082	4137504	wecF	0.932	1.196	0	0
1479	4137729	wecF	0.799	0.981	0	0
13400	4137852	wecF	1.035	1.283	0	0
11620	4138045	wecF, wecG	1.030	1.300	0	0
17367	4138422	wecF, wecG	1.081	1.333	0	0
2364	4138535	wecG	0.853	1.082	0	0
21685	4138845	wecG	1.134	1.374	0	0
5138	4139088	wecG, yifK	0.777	1.014	0	0
14439	4139309	yifK	0.878	0.980	0	0
2495	4139576	yifK	0.736	1.093	0	0
17072	4139664	yifK	0.679	1.233	0	0
19247	4139864	yifK	0.734	1.196	0	0
11538	4140151	yifK	0.727	1.298	0	0
13813	4140430	yifK, argX	0.550	2.239	0	1
10897	4140648	yifK, argX	0.457	3.250	0	1
20577	4140830	argX, hisR	0.457	3.664	0	1
17178	4141072	hisR, leuT	0.413	5.571	0	1
5175	4141362		0.372	3.572	0	1
1385	4141609		0.436	2.684	0	1
1757	4141753		0.345	1.853	0	0
19202	4141828		0.437	1.455	0	0
13325	4142245	hemY	0.823	2.171	0	1
20494	4142472	hemY	0.965	1.967	0	1
10217	4142749	hemY	0.832	1.720	0	1
16345	4142920	hemY	0.922	1.671	0	0
1626	4143064	hemY	0.508	0.920	0	0
301	4143261	hemY, hemX	1.204	1.635	0	0
12898	4143647	hemX	1.103	1.675	0	0
16543	4143895	hemX	1.100	1.608	0	0
1844	4143998	hemX	0.783	1.143	0	0

2391	4144266	hemX	1.049	1.822	0	0
20495	4144550	hemX, hemD	0.984	1.513	0	0
3665	4144693	hemX, hemD	0.986	1.626	0	1
12405	4144845	hemD	1.081	1.783	0	1
12167	4145211	hemD, hemC	0.992	1.415	0	0
22110	4145453	hemD, hemC	0.500	0.981	0	0
13723	4145610	hemC	0.847	1.306	0	0
19319	4145786	hemC	0.634	1.319	0	0
18071	4146016	hemC, cyaA	0.731	1.471	0	0
11957	4146362	hemC, cyaA	0.636	1.892	0	1
18428	4146545	cyaA	0.696	2.078	0	1
13249	4146811	cyaA	0.882	2.077	0	1
13321	4146955	cyaA	0.707	1.453	0	0
17441	4147210	cyaA	0.985	2.078	0	0
4709	4147506	cyaA	0.888	1.496	0	0
3469	4147668	cyaA	0.921	1.689	0	0
5822	4147775	cyaA	0.741	1.118	0	0
6282	4148120	cyaA	1.222	1.323	0	0
18290	4148427	cyaA	1.089	1.530	0	0
2978	4148609	cyaA, STM3940	1.067	1.422	0	0
15866	4148721	cyaA, STM3940	1.047	1.313	0	0
5648	4148993	STM3940	1.396	0.780	0	0
11229	4149202	STM3940, STM3941	2.557	0.669	1	0
19415	4149447	STM3941	3.107	0.533	1	0
1953	4149777	STM3941, STM3942	3.516	0.636	1	0
20504	4149880	STM3941, STM3942	4.061	0.482	1	0
14320	4150224	STM3942	2.830	0.556	1	0
18970	4150365	STM3942	1.968	0.632	0	0
11934	4150440	STM3942	2.066	0.756	0	0
14534	4150833	cyaY	1.022	0.785	0	0
7248	4150936	cyaY, STM3944	1.087	0.752	0	0
15948	4151348	STM3944	0.932	0.621	0	0
1439	4151493	STM3944	1.032	0.738	0	0
5816	4151839	yifL, dapF	1.056	0.887	0	0
18172	4151987	yifL, dapF	1.081	1.028	0	0
20247	4152246	dapF	1.139	1.388	0	0

18742	4152505	dapF	1.037	1.377	0	0
12032	4152576	dapF	0.800	1.142	0	0
17838	4152826	dapF, yigA	1.198	2.058	0	1
8861	4153052	dapF, yigA	1.257	2.477	0	1
21631	4153267	yigA	1.161	1.717	0	1
21692	4153485	yigA, xerC	1.161	1.809	0	1
8098	4153888	xerC	1.064	1.578	0	0
20242	4154019	xerC	1.053	1.472	0	0
3791	4154249	xerC, yigB	1.024	1.421	0	0
5294	4154599	xerC, yigB	1.014	1.315	0	0
12596	4154727	yigB	1.067	1.315	0	0
16217	4154799	yigB	1.158	1.572	0	0
6724	4155014	yigB	1.102	1.549	0	0
10085	4155388	uvrD	0.984	1.778	0	0
5755	4155654	uvrD	1.044	1.467	0	0
19916	4155888	uvrD	1.350	1.332	0	0
20324	4155966	uvrD	1.053	1.278	0	0
2899	4156173	uvrD	1.070	1.324	0	0
14002	4156508	uvrD	1.408	1.220	0	0
2934	4156694	uvrD	1.178	1.122	0	0
20309	4157015	uvrD	0.866	0.959	0	0
2126	4157091	uvrD	1.064	1.034	0	0
19632	4157362	uvrD	0.818	1.000	0	0
9175	4157657	uvrD	0.900	1.646	0	0
17225	4157981	corA	0.806	2.675	0	1
4089	4158098	corA	0.632	2.709	0	1
18639	4158369	corA	0.860	1.423	0	0
2717	4158547	corA	1.249	1.093	0	0
2509	4158905	corA	2.155	0.736	0	0
14469	4158991	corA	2.805	0.709	1	0
17556	4159222	yigF	4.840	0.484	1	0
13407	4159451	yigF	4.283	0.460	1	0
2593	4159669	yigF, yigG	4.215	0.590	1	0
5821	4159905	yigF, yigG	3.838	0.475	1	0
4120	4160011	yigG	2.313	0.610	0	0
12126	4160295	yigG, rarD	2.783	0.670	0	0

1798	4160620	rarD	1.436	0.786	0	0
6660	4160798	rarD	1.379	0.948	0	0
19744	4160942	rarD	1.170	0.931	0	0
21109	4161379	rarD, yigI	0.993	0.775	0	0
20582	4161565	yigI, pldA	0.986	0.856	0	0
5043	4161734	yigI, pldA	0.867	0.927	0	0
8443	4161841	yigI, pldA	0.851	0.870	0	0
14339	4162198	pldA, recQ	1.208	1.064	0	0
10264	4162469	recQ	1.040	1.189	0	0
7016	4162547	recQ	0.734	0.874	0	0
11027	4162773	recQ	1.158	1.481	0	0
22218	4163144	recQ	1.257	1.553	0	0
7788	4163217	recQ	1.280	1.555	0	0
3428	4163454	recQ	1.030	1.219	0	0
9752	4163795	recQ	1.110	1.318	0	0
10770	4163919	recQ	1.130	1.114	0	0
19459	4164173	recQ, rhtC	1.215	1.291	0	0
6673	4164461	rhtC	1.192	1.389	0	0
8086	4164821	rhtC	0.776	0.900	0	0
21845	4164920	rhtC	1.267	1.112	0	0
14558	4165058	rhtC	1.383	1.147	0	0
3975	4165365	rthB, pldB	1.095	1.053	0	0
258	4165688	rthB, pldB	0.981	1.812	0	0
15498	4165819	rthB, pldB	1.026	1.570	0	0
4292	4166070	rthB, pldB	0.804	1.482	0	0
14174	4166150	rthB, pldB	1.060	1.622	0	1
18112	4166348	pldB	0.803	1.805	0	1
12367	4166817	pldB, yigL	0.910	1.247	0	0
11338	4167028	yigL	1.008	1.248	0	0
13171	4167241	yigL	0.979	1.320	0	0
16659	4167405	yigL, yigM	0.689	0.986	0	0
8428	4167671	yigM	0.767	1.115	0	0
13074	4167982	yigM	0.881	0.991	0	0
19658	4168198	yigM	0.858	0.731	0	0
9058	4168428	yigM	1.138	0.701	0	0
20887	4168505	yigM	1.034	0.800	0	0

1876	4168828	metR	1.380	0.748	0	0
18110	4169024	metR	1.668	0.781	0	0
5131	4169194	metR	0.607	0.511	0	0
5571	4169479	metR, metE	1.513	0.744	0	0
14076	4169723	metR, metE	1.984	0.665	0	0
2628	4169912	metR, metE	1.493	0.905	0	0
6250	4170102	metE	1.330	0.782	0	0
14333	4170331	metE	0.866	0.620	0	0
5945	4170518	metE	1.319	0.783	0	0
20614	4170759	metE	1.690	0.991	0	0
11827	4171147	metE	0.510	0.287	0	0
12021	4171245	metE	1.484	0.864	0	0
1574	4171431	metE	1.337	0.697	0	0
3799	4171663	metE	0.894	0.629	0	0
4630	4171737	metE	1.279	0.726	0	0
8967	4172157	STM3966	1.688	0.712	0	0
12304	4172460	STM3966	1.586	0.876	0	0
16477	4172626	STM3966	1.593	0.980	0	0
12156	4172891	STM3966	1.301	0.793	0	0
20683	4172996	STM3966	1.328	0.873	0	0
8132	4173321	STM3966	0.637	0.663	0	0
5452	4173677	STM3966	1.084	0.889	0	0
1669	4173792	dlhH	1.094	0.914	0	0
13622	4173880	dlhH	1.278	1.108	0	0
12975	4174369	dlhH, udp	1.125	0.967	0	0
12893	4174597	dlhH, udp	0.910	0.955	0	0
818	4174801	dlhH, udp	0.853	0.918	0	0
11779	4174872	dlhH, udp	0.872	0.911	0	0
9581	4175031	dlhH, udp	0.767	0.806	0	0
7798	4175324	udp, yigN	0.992	0.994	0	0
4492	4175683	yigN	1.103	0.997	0	0
4684	4175863	yigN	1.242	0.978	0	0
19473	4175952	yigN	1.142	0.859	0	0
1247	4176095	yigN	1.283	0.956	0	0
3887	4176409	yigN	1.103	1.005	0	0
4895	4176529	yigN	1.150	0.985	0	0

9886	4176904	yigN, ubiE	1.015	1.001	0	0
16635	4177259	ubiE	0.738	0.994	0	0
3084	4177407	ubiE	0.785	1.107	0	0
14826	4177660	ubiE, yigP	0.786	1.165	0	0
22204	4177896	ubiE, yigP	0.905	1.293	0	0
4770	4178144	yigP	0.981	1.341	0	0
14701	4178256	yigP, aarF	0.915	1.450	0	0
4374	4178679	aarF	0.930	1.455	0	0
3943	4178798	aarF	0.919	1.337	0	0
21765	4179080	aarF	0.987	1.170	0	0
4243	4179295	aarF	0.857	0.999	0	0
10650	4179593	aarF	0.860	1.048	0	0
10102	4179714	aarF	0.838	1.136	0	0
8401	4179871	aarF	0.798	1.097	0	0
9619	4180115	aarF, tatA	0.733	1.157	0	0
2203	4180294	tatA, tatB	0.619	1.149	0	0
10081	4180637	tatA, tatB	0.535	1.423	0	0
5208	4180889	tatB, tatC	0.645	1.790	0	1
20770	4181053	tatB, tatC	0.652	1.883	0	1
2741	4181291	tatC	0.675	1.714	0	1
3719	4181415	tatC	0.746	1.600	0	1
12094	4181642	tatC, yigW	0.690	1.772	0	1
16903	4181739	tatC, yigW	0.618	1.530	0	0
14988	4182132	yigW	0.796	1.459	0	0
20744	4182526	yigW	0.758	1.589	0	0
18748	4182682	yigW	0.824	1.994	0	0
21776	4182899	rfaH	0.677	1.785	0	0
20823	4183218	rfaH, yigC	0.757	1.911	0	1
22024	4183378	rfaH, yigC	0.687	2.009	0	1
5338	4183508	rfaH, yigC	0.656	1.976	0	1
12188	4183657	rfaH, yigC	0.714	2.011	0	1
12826	4184114	yigC	0.809	1.665	0	1
20994	4184273	yigC	0.777	1.749	0	0
20639	4184599	yigC	0.714	1.599	0	0
6205	4184747	yigC, ubiB	0.742	1.569	0	0
6207	4184856	yigC, ubiB	0.683	1.403	0	0

6192	4185165	ubiB	0.846	1.470	0	0
19038	4185326	ubiB	0.895	1.420	0	0
9580	4185595	ubiB	1.851	1.136	0	0
21891	4185885	STM3980	2.835	0.678	1	0
17880	4186130	STM3980	2.776	0.626	1	0
19008	4186320	STM3980	3.728	0.635	1	0
11011	4186539	STM3980, STM3981	3.716	0.512	1	0
3439	4186694	STM3980, STM3981	3.139	0.640	1	0
12934	4187059	STM3981	1.668	0.688	0	0
19246	4187262	STM3981	1.241	0.744	0	0
9922	4187472	STM3981	1.028	0.683	0	0
9478	4187712	STM3981	1.117	0.727	0	0
9290	4187803	STM3981	1.342	0.841	0	0
13740	4188100		1.227	0.861	0	0
21678	4188294	fadA	1.316	1.058	0	0
18087	4188367	fadA	1.190	0.918	0	0
11961	4188676	fadA	1.061	0.772	0	0
15941	4188981	fadA	1.094	0.835	0	0
19762	4189268	fadA	1.171	0.854	0	0
368	4189550	fadA, fadB	1.452	1.170	0	0
11784	4189683	fadA, fadB	1.113	0.821	0	0
6418	4189887	fadB	1.034	0.803	0	0
7132	4190139	fadB	1.244	0.891	0	0
17788	4190274	fadB	1.330	1.046	0	0
18574	4190561	fadB	1.382	0.937	0	0
11224	4190794	fadB	1.272	0.976	0	0
1283	4190947	fadB	0.944	0.749	0	0
21961	4191235	fadB, pepQ	1.270	0.824	0	0
6386	4191500	fadB, pepQ	1.137	1.128	0	0
21295	4191796	fadB, pepQ	1.167	1.071	0	0
10461	4191909	fadB, pepQ	1.069	1.051	0	0
20143	4192116	pepQ	0.854	0.936	0	0
3937	4192366	pepQ	0.716	0.844	0	0
13717	4192614	pepQ, yigZ	1.027	1.139	0	0
21688	4192810	pepQ, yigZ	1.005	1.162	0	0
21125	4192902	pepQ, yigZ	1.214	1.206	0	0

17375	4193390	yigZ, trkH	0.923	1.122	0	0
12944	4193560	yigZ, trkH	1.183	1.475	0	0
709	4193666	trkH	1.092	1.279	0	0
5194	4193934	trkH	0.790	1.126	0	0
22099	4194358	trkH	1.002	1.283	0	0
5941	4194530	trkH	0.951	1.219	0	0
15559	4194731	trkH, hemG	0.941	1.214	0	0
17385	4194890	trkH, hemG	0.929	1.291	0	0
1675	4195045	trkH, hemG	0.724	1.137	0	0
11956	4195118	hemG	0.777	1.256	0	0
16864	4195512	hemG	0.531	2.829	0	1
1382	4195647	hemG, rrsA	0.475	4.230	0	1
13038	4195909	rrsA	0.348	3.617	0	1
1907	4196040	rrsA	0.361	4.106	0	1
14546	4196485	rrsA	0.367	5.370	0	1
9788	4196836	rrsA	0.356	5.882	0	1
8328	4197012	rrsA	0.337	6.440	0	1
15456	4197171	rrsA, ileT	0.343	5.668	0	1
9989	4197484	rrsA, ileT	0.344	5.857	0	1
11534	4197721	alaT, rrlA	0.339	4.464	0	1
20181	4197910	rrlA	0.358	4.094	0	1
6848	4198077	rrlA	0.342	4.320	0	1
10678	4198252	rrlA	0.366	3.932	0	1
5961	4198663	rrlA	0.331	4.066	0	1
21713	4198813	rrlA	0.355	5.332	0	1
877	4198904	rrlA	0.341	5.872	0	1
15215	4199171	rrlA	0.388	5.859	0	1
6115	4199429	rrlA	0.413	5.464	0	1
19765	4199780	rrlA	0.355	4.722	0	1
7762	4199934	rrlA	0.332	4.234	0	1
3402	4200113	rrlA	0.341	4.882	0	1
8222	4200258	rrlA	0.363	5.103	0	1
18260	4200356	rrlA	0.374	4.850	0	1
14140	4200912	rrlA, rrfA	0.414	5.129	0	1
3985	4201135	rrfA	0.358	4.039	0	1
19275	4201218	rrfA	0.399	5.293	0	1

6793	4201533		0.517	3.363	0	1
3983	4201800	mobB	0.610	1.724	0	0
13774	4202045	mobB	0.852	1.566	0	0
8626	4202252	mobB, mobA	0.837	1.439	0	0
5005	4202440	mobB, mobA	0.881	1.229	0	0
15116	4202754	mobA, yihD	0.867	1.520	0	0
14527	4202904	mobA, yihD	0.879	1.490	0	0
3519	4203169	mobA, yihE	0.752	1.905	0	1
9129	4203284	yihE	0.757	1.810	0	1
4250	4203381	yihE	0.814	1.724	0	0
14193	4203901	yihE, dsbA	0.915	1.080	0	0
11434	4204104	yihE, dsbA	0.715	0.857	0	0
2757	4204311	dsbA	0.778	1.152	0	0
8518	4204502	dsbA	0.920	1.314	0	0
15768	4204717	dsbA	0.838	0.828	0	0
5619	4204883	yihG	0.822	0.742	0	0
12116	4205185	yihG	0.850	0.708	0	0
1165	4205372	yihG	0.816	0.943	0	0
14830	4205663	yihG, polA	0.730	1.261	0	0
6014	4205900	yihG, polA	0.962	1.369	0	0
10989	4206120	yihG, polA	0.743	0.982	0	0
21721	4206313	polA	0.896	1.063	0	0
4323	4206543	polA	0.995	1.182	0	0
292	4206818	polA	1.275	1.161	0	0
5788	4206932	polA	1.024	0.985	0	0
21493	4207161	polA	1.034	1.162	0	0
18407	4207495	polA	0.946	1.016	0	0
4499	4207684	polA	0.790	0.835	0	0
13254	4207861	polA	1.142	1.206	0	0
19308	4208204	polA	1.190	1.058	0	0
11903	4208444	polA	1.294	1.006	0	0
11277	4208558	polA	0.999	0.906	0	0
9310	4208756	polA, spf	1.021	1.199	0	0
736	4209061	spf	0.786	1.398	0	0
18809	4209248	yihA	0.807	1.362	0	0
9361	4209461	yihA	0.695	0.937	0	0

2202	4209826	yihA, STM4002	0.816	1.092	0	0
4558	4209897	yihA, STM4002	0.747	1.098	0	0
6781	4210104	yihA, STM4002	0.779	1.135	0	0
13594	4210380	yihI	0.702	1.225	0	0
3596	4210689	yihI, hemN	0.907	1.348	0	0
16694	4210868	yihI, hemN	0.736	1.291	0	0
15942	4211017	hemN	0.541	0.911	0	0
8260	4211170	hemN	0.713	1.461	0	0
21455	4211563	hemN	1.114	1.434	0	0
17028	4211776	hemN	0.986	1.046	0	0
18153	4211963	hemN	1.096	1.199	0	0
15162	4212249	hemN	0.991	1.478	0	0
481	4212418	hemN	1.212	1.928	0	0
19331	4212698		0.822	1.565	0	0
10112	4212812		1.100	1.689	0	0
13008	4212925	glnG	0.892	1.231	0	0
21533	4213219	glnG	1.468	1.165	0	0
12578	4213634	glnG	1.550	1.007	0	0
2833	4213765	glnG	1.292	1.025	0	0
10134	4213917	glnG	1.486	0.927	0	0
5448	4214229	glnG	1.317	0.992	0	0
18836	4214586	glnG, glnL	1.249	1.028	0	0
11795	4214769	glnL	1.126	1.067	0	0
5223	4214937	glnL	1.013	1.182	0	0
10275	4215115	glnL	1.148	1.418	0	0
8185	4215318	glnL	0.570	0.901	0	0
21624	4215578	glnL, glnA	1.148	1.447	0	0
1515	4215804	glnA	0.839	1.119	0	0
1569	4216145	glnA	0.863	0.971	0	0
15396	4216282	glnA	0.774	1.064	0	0
21652	4216381	glnA	0.629	1.021	0	0
19290	4216839	glnA	0.759	1.604	0	0
19478	4216957	glnA, typA	0.708	1.929	0	0
7685	4217169	glnA, typA	0.777	1.584	0	0
10545	4217332	glnA, typA	0.694	1.858	0	0
7630	4217552	typA, STM4008	0.741	1.530	0	0

7379	4217713	typA, STM4008	0.889	1.441	0	0
18343	4218095	typA	0.897	1.097	0	0
14334	4218340	typA	0.940	1.246	0	0
2812	4218489	typA	0.853	1.142	0	0
18351	4218724	typA	0.969	1.065	0	0
21518	4219090	typA	1.201	1.063	0	0
7570	4219186		1.357	0.996	0	0
7493	4219585	STM4010	1.500	0.733	0	0
20830	4219760	STM4010	1.568	0.748	0	0
6974	4219915	STM4010	1.613	0.672	0	0
20907	4220093	STM4010, STM4011	1.505	0.576	0	0
16364	4220168	STM4010, STM4011	1.562	0.675	0	0
11278	4220528	STM4011	1.523	0.666	0	0
20833	4220659	STM4011	1.476	0.654	0	0
3789	4221098	STM4011, STM4012	1.793	0.576	0	0
3991	4221365	STM4012	2.419	0.562	1	0
1114	4221482	STM4012	2.228	0.661	1	0
21738	4221684	STM4012	3.329	0.578	1	0
1590	4222020	STM4012	2.934	0.576	1	0
1068	4222111	STM4012, STM4013	2.272	0.571	0	0
1629	4222445	STM4012, STM4013	2.295	0.532	0	0
12579	4222652	STM4013	2.110	0.454	0	0
13312	4222836	STM4013	2.674	0.602	0	0
4068	4223183	STM4013, STM4014	1.977	0.552	0	0
988	4223339	STM4013, STM4014	1.666	0.561	0	0
20871	4223429	STM4014	1.374	0.522	0	0
19058	4223745	STM4014	1.210	0.575	0	0
696	4223980	STM4014, STM4015	1.298	0.618	0	0
5690	4224051	STM4014, STM4015	1.132	0.564	0	0
13257	4224599	STM4015	2.731	0.527	1	0
1581	4224736	STM4015	3.710	0.507	1	0
9267	4224811	STM4015	3.249	0.457	1	0
20322	4225105	STM4015, yshA	3.668	0.383	1	0
17389	4225458	yshA	3.834	0.517	1	0
18184	4225641	yshA	3.529	0.515	1	0
21812	4225805	yshA, yihO	4.371	0.508	1	0

16712	4225949	yshA, yihO	4.245	0.488	1	0
3647	4226172	yihO	2.991	0.583	0	0
7505	4226500	yihO	1.832	0.620	0	0
5306	4226837	yihO	1.903	0.597	0	0
2534	4227065	yihO	1.848	0.611	0	0
3446	4227212	yihO, yihP	1.510	0.650	0	0
3573	4227303	yihO, yihP	1.683	0.678	0	0
234	4227735	yihP	1.293	0.752	0	0
810	4227916	yihP	1.617	0.768	0	0
1020	4228201	yihP	1.441	0.910	0	0
17548	4228392	yihP	1.791	0.980	0	0
21681	4228526	yihP	1.507	0.932	0	0
11710	4228874	yihP, yihQ	1.375	0.636	0	0
17686	4228985	yihP, yihQ	1.076	0.714	0	0
20971	4229257	yihQ	1.304	0.663	0	0
17224	4229515	yihQ	1.840	0.768	0	0
13895	4229688	yihQ	1.402	0.778	0	0
6511	4229845	yihQ	1.629	0.792	0	0
7694	4230077	yihQ	1.578	0.775	0	0
9861	4230311	yihQ	1.185	0.743	0	0
22313	4230645	yihQ	1.224	0.945	0	0
10131	4230716	yihQ, yihR	1.361	0.950	0	0
11932	4231064	yihQ, yihR	1.375	0.858	0	0
1299	4231194	yihR	1.207	0.913	0	0
3987	4231553	yihR	1.191	0.815	0	0
19118	4231764	yihR, yihS	1.391	0.739	0	0
7377	4231911	yihR, yihS	1.474	0.708	0	0
5270	4232139	yihS	1.457	0.666	0	0
21943	4232384	yihS	1.374	0.677	0	0
17850	4232501	yihS	1.437	0.730	0	0
10819	4232840	yihS, yihT	1.364	0.651	0	0
18211	4233033	yihS, yihT	1.066	0.592	0	0
6694	4233250	yihS, yihT	1.300	0.610	0	0
7171	4233390	yihT	1.155	0.528	0	0
16094	4233710	yihT	0.747	0.568	0	0
10338	4234112	yihT, yihU	1.074	0.654	0	0

3299	4234333	yihU, yihV	1.109	0.564	0	0
6877	4234594	yihU, yihV	0.949	0.669	0	0
9946	4234805	yihU, yihV	0.873	0.567	0	0
11445	4234876	yihU, yihV	0.966	0.665	0	0
16425	4235101	yihV	1.322	0.733	0	0
19930	4235399	yihV, yihW	1.313	0.790	0	0
17864	4235688	yihV, yihW	1.252	0.914	0	0
10792	4235821	yihW	1.340	1.102	0	0
8757	4236024	yihW	1.394	1.093	0	0
18229	4236318	yihW, yihX	1.314	1.207	0	0
12500	4236476	yihW, yihX	0.928	1.083	0	0
14312	4236727	yihX	0.976	1.191	0	0
7439	4236915	yihX, rbn	0.980	1.227	0	0
21686	4237298	yihX, rbn	0.979	1.181	0	0
18946	4237524	rbn	1.019	1.185	0	0
14985	4237663	rbn	0.970	1.039	0	0
9856	4237902	rbn, yihZ	1.010	1.241	0	0
4794	4238070	rbn, yihZ	0.966	1.249	0	0
14818	4238282	yihZ, yiiD	0.976	1.280	0	0
5575	4238676	yiiD	1.396	1.272	0	0
1886	4238875	yiiD	1.346	1.172	0	0
16993	4239021	yiiD	1.018	1.078	0	0
852	4239196	yiiD	1.249	1.286	0	0
11812	4239384	yiiD	1.128	1.425	0	0
7142	4239748	STM4030	0.813	1.237	0	0
7879	4239884	STM4030	0.777	1.140	0	0
12213	4240039	STM4030	0.793	0.983	0	0
5330	4240300	STM4030, STM4031	0.779	0.761	0	0
352	4240389	STM4030, STM4031	0.769	0.832	0	0
1156	4240467	STM4030, STM4031	0.824	0.744	0	0
12432	4240774	STM4031, STM4032	0.883	0.864	0	0
16758	4241097	STM4032	1.004	0.744	0	0
19532	4241469	STM4032	0.892	0.656	0	0
10369	4241701	STM4032, STM4033	0.842	0.729	0	0
6270	4241964	STM4033	0.868	0.945	0	0
6448	4242209		0.965	1.612	0	0

12794	4242387	fdhE	0.922	1.539	0	0
11128	4242476	fdhE	0.783	1.292	0	0
6267	4242858	fdhE	0.990	1.701	0	1
11696	4243195	fdhE, fdoI	0.912	1.940	0	1
21817	4243352	fdhE, fdoI	0.978	2.103	0	1
18560	4243591	fdhE, fdoI	0.814	1.307	0	0
10762	4243832	fdoI, fdoH	0.866	1.764	0	1
1778	4243983	fdoI, fdoH	0.925	1.706	0	1
3431	4244344	fdoH	0.773	1.943	0	1
4410	4244542	fdoH	0.582	1.221	0	0
10686	4244734	fdoH, fdoG	0.923	1.552	0	0
20386	4244867	fdoH, fdoG	0.875	1.646	0	0
484	4245102	fdoH, fdoG	1.129	1.880	0	1
19492	4245367	fdoG	0.880	1.727	0	1
11568	4245683	fdoG	0.794	1.691	0	0
15931	4245917	fdoG	0.724	1.324	0	0
2172	4246148	fdoG	0.799	1.752	0	1
1306	4246253	fdoG	0.867	1.855	0	1
11834	4246585	fdoG	0.782	1.652	0	0
9755	4246754	fdoG	0.699	1.933	0	1
1859	4246887	fdoG	0.816	2.402	0	1
13883	4247265	fdoG	0.875	1.964	0	1
16215	4247394	fdoG	0.780	2.285	0	1
11846	4247555	fdoG, fdhD	0.938	2.445	0	1
21909	4247823	fdoG, fdhD	0.731	1.654	0	1
17763	4248017	fdoG, fdhD	0.785	1.336	0	0
6004	4248243	fdoG, fdhD	0.964	1.205	0	0
3549	4248419	fdhD	1.053	0.967	0	0
2346	4248896		1.486	0.809	0	0
2275	4249096	STM4039	1.384	0.613	0	0
18309	4249342	STM4039	1.907	0.563	0	0
9577	4249437	STM4039	2.291	0.577	1	0
5577	4249576	STM4039	2.542	0.584	1	0
2435	4249868	STM4039, yiiG	3.279	0.584	1	0
6046	4250011	STM4039, yiiG	3.505	0.651	1	0
18436	4250311	STM4039, yiiG	3.840	0.524	1	0

9732	4250666	yiiG	3.417	0.517	1	0
254	4250914	yiiG	3.049	0.586	1	0
12607	4251046	yiiG	4.403	0.388	1	0
2160	4251271	yiiG	2.830	0.625	1	0
7022	4251567		2.144	0.417	0	0
10524	4251657		2.220	0.601	0	0
3372	4251734		2.085	0.610	0	0
17378	4252033	STM4041	1.840	0.725	0	0
8785	4252396	STM4041, STM4042	0.724	0.693	0	0
19096	4252693	STM4042, STM4042A	0.852	0.579	0	0
12671	4252838	STM4042, STM4042A	1.253	0.833	0	0
16814	4252955	STM4042, STM4042A	0.923	0.827	0	0
19040	4253348	STM4042A	1.469	1.283	0	0
7270	4253540	yiiL	1.315	1.377	0	0
16310	4253889	yiiL, STM4044	0.624	0.638	0	0
18963	4253961	yiiL, STM4044	1.493	0.873	0	0
17765	4254243	yiiL, STM4044	1.427	0.691	0	0
1803	4254443	STM4044	1.516	0.633	0	0
15733	4254757	STM4044	1.575	0.670	0	0
17395	4254933	STM4044	1.337	0.741	0	0
12224	4255174	STM4044, rhaD	1.439	0.733	0	0
8493	4255437	rhaD	1.366	0.728	0	0
14185	4255652	rhaD	1.774	0.870	0	0
21036	4255881	rhaD	1.329	0.741	0	0
12581	4256029	rhaD, rhaA	1.194	0.525	0	0
6763	4256342	rhaD, rhaA	0.878	0.455	0	0
18065	4256574	rhaA	1.725	0.751	0	0
12245	4256821	rhaA	1.483	0.706	0	0
18175	4256927	rhaA	0.992	0.645	0	0
3238	4257289	rhaA, rhaB	0.966	0.566	0	0
17827	4257431	rhaA, rhaB	1.757	0.686	0	0
18525	4257731	rhaA, rhaB	1.736	0.724	0	0
17094	4257971	rhaB	1.586	0.693	0	0
17810	4258164	rhaB	1.301	0.832	0	0
11749	4258243	rhaB	1.457	0.653	0	0
9673	4258561	rhaB	1.238	0.700	0	0

13678	4258802	rhaB, rhaS	1.143	0.684	0	0
4931	4259019	rhaB, rhaS	1.117	0.683	0	0
14066	4259140	rhaB, rhaS	1.154	0.681	0	0
4687	4259243	rhaS	1.138	0.774	0	0
4351	4259798	rhaS, rhaR	1.318	1.063	0	0
13735	4259885	rhaR	1.424	1.032	0	0
19099	4260122	rhaR	1.351	1.076	0	0
220	4260240	rhaR	1.106	1.170	0	0
11560	4260564	rhaR	1.325	0.887	0	0
10075	4260785	rhaR	1.161	0.785	0	0
9709	4260869	rhaR, rhaT	1.411	0.809	0	0
6177	4261196	rhaT	1.441	0.780	0	0
12629	4261406	rhaT	1.327	0.700	0	0
4094	4261696	rhaT	1.395	0.712	0	0
6593	4261887	rhaT	2.211	0.564	0	0
8979	4262205	rhaT, STM4051	3.323	0.520	1	0
9089	4262483	STM4051	2.948	0.422	1	0
15546	4262680	STM4051	2.661	0.548	1	0
21557	4262948	STM4051	3.529	0.595	1	0
1893	4263147	STM4051	3.446	0.576	1	0
8612	4263404	STM4052	3.810	0.558	1	0
19241	4263672	STM4052	3.216	0.594	1	0
294	4263883	STM4052	2.341	0.739	1	0
15250	4264039	STM4052	2.078	0.718	0	0
17851	4264349	STM4052	1.689	0.693	0	0
9280	4264556	STM4052	1.256	0.619	0	0
6815	4264671	STM4052, STM4053	1.258	0.643	0	0
9611	4264890	STM4052, STM4053	1.739	0.640	0	0
718	4265181	STM4053	1.524	0.539	0	0
20711	4265300	STM4053, STM4054	1.456	0.591	0	0
3153	4265480	STM4053, STM4054	1.337	0.585	0	0
19519	4265847	STM4054	1.069	0.531	0	0
15994	4266147	STM4054, sodA	1.509	0.722	0	0
2377	4266235	STM4054, sodA	1.195	0.732	0	0
12723	4266591	STM4054, sodA	0.695	0.825	0	0
18062	4266835	sodA, yiiM	1.178	1.593	0	0

20598	4266948	sodA, yiiM	0.885	1.348	0	0
7768	4267215	yiiM	0.522	0.498	0	0
1242	4267482	yiiM, STM4057	1.307	1.105	0	0
16410	4267584	yiiM, STM4057	1.217	0.942	0	0
12986	4267918	STM4057	1.304	0.945	0	0
12716	4268127	STM4057	1.393	1.212	0	0
11005	4268225	STM4057	1.368	1.222	0	0
6522	4268599	cpxA	1.327	1.445	0	0
21229	4268845	cpxA	1.218	1.499	0	0
3602	4268995	cpxA	1.220	1.430	0	0
11758	4269278	cpxA	1.076	1.220	0	0
16523	4269552	cpxA	1.166	1.604	0	0
2292	4269733	cpxA, cpxR	1.161	1.615	0	0
14317	4269877	cpxA, cpxR	1.002	1.333	0	0
19809	4270217	cpxR, cpxP	0.370	0.460	0	0
5925	4270395	cpxR, cpxP	0.856	1.445	0	0
10091	4270533	cpxR, cpxP	0.825	1.797	0	1
15810	4270808	cpxR, cpxP	0.767	1.706	0	1
19602	4271032	cpxP, yiiP	0.873	1.692	0	1
20264	4271304	yiiP	0.643	0.981	0	0
3028	4271523	yiiP	0.909	1.185	0	0
14238	4271784	yiiP	0.829	1.339	0	0
10266	4272053	yiiP, pfkA	0.871	1.344	0	0
5001	4272224	pfkA	0.872	1.272	0	0
12107	4272302	pfkA	0.675	0.906	0	0
19214	4272461	pfkA	0.893	1.197	0	0
20894	4272956	pfkA	0.731	0.937	0	0
1630	4273184	pfkA, sbp	0.672	0.872	0	0
15268	4273429	sbp	0.472	0.684	0	0
17777	4273532	sbp	0.579	0.849	0	0
12029	4273855	sbp	0.770	0.695	0	0
18943	4274139	sbp, ushB	0.912	0.717	0	0
12782	4274273	sbp, ushB	0.724	0.632	0	0
14095	4274551	ushB	0.969	0.904	0	0
663	4274766	ushB	1.020	1.168	0	0
2280	4274876	ushB	1.346	1.067	0	0

11909	4275002	ushB	1.512	1.016	0	0
5704	4275319	STM4065	1.759	0.817	0	0
3347	4275655	STM4065	1.904	0.624	0	0
13339	4275843	STM4065	1.891	0.594	0	0
4291	4275926	STM4065	1.884	0.565	0	0
17658	4276236	STM4065	3.452	0.755	1	0
21142	4276433	STM4065	3.746	0.628	1	0
18689	4276566	STM4065, STM4066	3.211	0.710	1	0
7756	4276828	STM4065, STM4066	1.740	0.519	0	0
7335	4276924	STM4066	1.988	0.584	0	0
11691	4277413	STM4066	1.203	0.681	0	0
7312	4277628	STM4066, STM4067	1.267	0.708	0	0
21294	4277888	STM4067	1.379	0.562	0	0
731	4278077	STM4067	1.881	0.808	0	0
22040	4278278	STM4067	1.909	0.864	0	0
13673	4278397	STM4067	1.855	0.957	0	0
18278	4278883	STM4067, STM4068	1.367	1.584	0	0
18625	4279063	STM4068	1.180	1.434	0	0
930	4279161	STM4068	1.349	1.650	0	0
21830	4279533	STM4068, STM4069	1.423	1.353	0	0
17484	4279725	STM4069, STM4070	1.255	1.242	0	0
5536	4279807	STM4069, STM4070	0.780	0.769	0	0
3610	4280094	STM4070	1.608	1.109	0	0
9945	4280361	STM4071	1.247	0.825	0	0
5408	4280556	STM4071	1.469	0.868	0	0
1908	4280713	STM4071, ydeV	1.132	0.677	0	0
18319	4281014	ydeV	1.589	0.652	0	0
14855	4281230	ydeV	1.208	0.647	0	0
4316	4281566	ydeV	1.236	0.783	0	0
13352	4281789	ydeV	1.908	0.795	0	0
16127	4282069	ydeV	2.751	0.650	1	0
16901	4282151	ydeV	2.950	0.609	1	0
4244	4282404	ydeV, ydeW	3.581	0.534	1	0
21644	4282571	ydeV, ydeW	3.869	0.735	1	0
21767	4282987	ydeW	1.285	0.658	0	0
6892	4283199	ydeW, ego	0.866	0.731	0	0

10565	4283369	ydeW, ego	1.025	0.806	0	0
14400	4283494	ydeW, ego	0.930	0.929	0	0
11443	4283783	ego	1.132	0.890	0	0
6080	4284023	ego	1.367	0.691	0	0
1593	4284152	ego	1.402	0.704	0	0
13307	4284565	ego, ydeY	1.343	0.746	0	0
8999	4284727	ego, ydeY	1.485	0.737	0	0
18619	4284847	ego, ydeY	1.604	0.711	0	0
20068	4285032	ydeY	1.375	0.613	0	0
6672	4285448	ydeY	1.802	0.742	0	0
15981	4285620	ydeY, ydeZ	1.738	0.643	0	0
1955	4285812	ydeY, ydeZ	1.843	0.646	0	0
18975	4286017	ydeY, ydeZ	1.932	0.569	0	0
19242	4286235	ydeZ	2.499	0.515	0	0
5793	4286510	ydeZ	3.084	0.576	0	0
11897	4286581	ydeZ	2.043	0.553	0	0
10692	4287068	yneA	1.258	0.625	0	0
7778	4287293	yneA	1.204	0.625	0	0
21485	4287400	yneA	1.091	0.549	0	0
5753	4287587	yneA	1.412	0.635	0	0
21218	4287936	yneA, yneB	2.754	0.598	1	0
8975	4288177	yneB	2.947	0.486	1	0
18026	4288274	yneB	3.004	0.599	1	0
1641	4288565	yneB	2.322	0.667	0	0
20346	4288908	yneB, yneC	2.224	0.544	0	0
2604	4289055	yneC, STM4080	1.677	0.700	0	0
11566	4289133	yneC, STM4080	1.601	0.615	0	0
16633	4289364	yneC, STM4080	1.312	1.129	0	0
18117	4289711	STM4080	1.038	1.385	0	0
21450	4289840	STM4080	1.190	1.252	0	0
19027	4290049	STM4080	0.952	1.156	0	0
9254	4290412	tpiA	1.097	1.100	0	0
19110	4290672	tpiA	1.127	1.183	0	0
22183	4290774	tpiA	1.111	1.122	0	0
14554	4290995	tpiA	1.106	1.136	0	0
3214	4291093	tpiA	1.037	1.073	0	0

4309	4291568	yiiQ, yiiR	1.083	0.898	0	0
1946	4291691	yiiQ, yiiR	0.968	0.915	0	0
9987	4291787	yiiQ, yiiR	1.384	0.924	0	0
4015	4292206	yiiR	1.170	1.079	0	0
10742	4292481	fpr	0.672	0.966	0	0
6373	4292707	fpr	1.063	1.694	0	0
11239	4292913	fpr	1.038	1.715	0	1
21002	4293096	fpr	1.030	1.653	0	1
10468	4293218	fpr, glpX	1.031	1.627	0	0
12823	4293346	fpr, glpX	1.012	1.503	0	0
18339	4293850	glpX	0.872	1.884	0	1
9051	4294048	glpX	0.668	2.588	0	1
6949	4294194	glpX	0.714	4.000	0	1
2620	4294437	glpX, glpK	0.474	3.926	0	1
15258	4294815	glpK	0.546	4.366	0	1
7455	4294886	glpK	0.414	3.482	0	1
16004	4295135	glpK	0.389	3.964	0	1
11857	4295395	glpK	0.421	3.538	0	1
3023	4295689	glpK	0.367	3.376	0	1
3585	4295838	glpK	0.427	2.570	0	1
1881	4296116	glpK, glpF	0.478	3.298	0	1
19857	4296355	glpF	0.400	2.556	0	1
1484	4296446	glpF	0.442	2.469	0	1
6106	4296674	glpF, yiiU	0.389	2.246	0	1
4742	4296951	glpF, yiiU	0.526	1.613	0	0
4496	4297098	glpF, yiiU	0.557	1.660	0	0
17906	4297177	yiiU	0.499	1.676	0	0
5732	4297317	yiiU	0.466	1.699	0	1
2616	4297896	menG	0.663	2.021	0	1
21010	4298022	menG	0.746	1.708	0	0
14778	4298412	menG, menA	0.715	1.041	0	0
1716	4298574	menA	0.673	0.868	0	0
2306	4298746	menA	0.792	1.068	0	0
20443	4299130	menA, hslU	0.738	1.703	0	0
2299	4299351	menA, hslU	0.743	2.036	0	1
1050	4299558	hslU	0.611	2.004	0	1

3003	4299777	hslU	0.610	2.252	0	1
19961	4299848	hslU	0.604	1.970	0	0
2104	4300185	hslU	0.714	1.713	0	1
6034	4300427	hslU	0.722	1.675	0	1
13822	4300502	hslU, hslV	0.613	1.472	0	0
1028	4300800	hslU, hslV	0.817	2.718	0	1
16999	4300992	hslV	0.775	3.375	0	1
18589	4301206	hslV, ftsN	0.856	3.230	0	1
22181	4301299	hslV, ftsN	0.768	2.456	0	1
14417	4301700	ftsN	0.678	1.603	0	0
6749	4301873	ftsN	0.944	2.475	0	1
21810	4302124	ftsN	0.841	2.035	0	1
18632	4302206	ftsN, cytR	0.766	1.734	0	1
9194	4302555	cytR	0.857	1.178	0	0
8011	4302830	cytR	0.804	1.101	0	0
738	4302967	cytR	0.938	1.219	0	0
16194	4303243	cytR	0.941	1.146	0	0
7121	4303314	cytR	0.889	1.157	0	0
17391	4303539	cytR, priA	0.654	0.919	0	0
787	4303714	priA	0.715	0.744	0	0
3690	4304281	priA	1.042	1.211	0	0
4999	4304571	priA	1.435	1.439	0	0
1405	4304678	priA	1.228	1.207	0	0
6707	4304819	priA	1.111	1.054	0	0
13868	4305159	priA	0.764	1.040	0	0
9839	4305331	priA, rpmE	0.744	1.440	0	0
22142	4305558	priA, rpmE	0.803	3.349	0	1
524	4305747	priA, rpmE	0.926	2.742	0	1
8845	4306020	STM4097	0.891	1.556	0	0
18141	4306279	STM4097	0.541	0.391	0	0
20761	4306601	STM4097	1.300	0.733	0	0
3880	4306795	STM4097	1.126	0.729	0	0
17114	4306945	STM4097, STM4098	1.168	0.712	0	0
4678	4307132	STM4098	1.365	0.607	0	0
20863	4307401	STM4098	1.657	0.675	0	0
21291	4307743	STM4098	2.854	0.492	1	0

16837	4307957	STM4098	3.585	0.539	1	0
21520	4308138	STM4098	2.802	0.554	1	0
20571	4308270	STM4098	3.579	0.554	1	0
14635	4308441	STM4098	4.298	0.606	1	0
12353	4308773	STM4098	3.320	0.544	1	0
12347	4309029	STM4098	3.718	0.643	1	0
1616	4309350	metJ, metB	1.898	0.925	0	0
21646	4309438	metJ, metB	1.993	1.051	0	0
19871	4309636	metJ, metB	1.308	0.769	0	0
3177	4309772	metJ, metB	0.959	0.561	0	0
6896	4310134	metB	1.360	0.875	0	0
9156	4310343	metB	1.130	1.067	0	0
22009	4310489	metB, metL	1.362	1.194	0	0
13900	4310786	metB, metL	1.408	1.245	0	0
14022	4311054	metL	1.007	0.932	0	0
6675	4311157	metL	1.422	1.068	0	0
558	4311358	metL	1.215	1.018	0	0
1034	4311807	metL	0.572	0.513	0	0
20581	4311949	metL	1.746	1.103	0	0
2627	4312240	metL	0.666	0.529	0	0
17022	4312420	metL	1.447	1.043	0	0
16648	4312492	metL	1.024	0.777	0	0
10010	4312979	metL	1.640	1.035	0	0
21888	4313176	metL	1.707	1.086	0	0
1713	4313336		1.829	0.874	0	0
5372	4313485	STM4102	2.384	0.810	1	0
17979	4313788	STM4102	2.965	0.712	1	0
9490	4314106	STM4102	2.772	0.587	1	0
10626	4314256	STM4102	2.998	0.633	1	0
17090	4314585	STM4102	4.467	0.552	1	0
15613	4314792		3.676	0.505	1	0
3813	4314867		3.524	0.519	1	0
19221	4315161		3.643	0.538	1	0
17073	4315240		3.094	0.610	1	0
15301	4315494	STM4103	3.321	0.552	1	0
20528	4315653	STM4103	2.960	0.629	1	0

16283	4316016	STM4103	1.158	0.678	0	0
2293	4316205	STM4103	1.338	0.846	0	0
1609	4316386	STM4103, STM4104	1.146	0.955	0	0
6584	4316758	STM4103, STM4104	1.161	0.775	0	0
20557	4317026	STM4103, STM4104	1.579	0.636	0	0
7367	4317165	STM4104	1.446	0.575	0	0
7021	4317458	STM4104	1.145	0.615	0	0
5229	4317622	STM4104	0.771	0.600	0	0
8945	4317930	STM4104	1.171	0.729	0	0
5421	4318033	STM4104, metF	1.048	0.630	0	0
16708	4318388	metF	1.182	0.709	0	0
12033	4318483	metF	1.033	0.701	0	0
18714	4318559	metF	1.234	0.757	0	0
15951	4318638	metF	1.271	0.916	0	0
8806	4319293	metF, katG	1.784	0.858	0	0
16419	4319555	katG	1.075	0.611	0	0
12665	4319737	katG	1.755	1.031	0	0
16490	4319867	katG	1.245	0.887	0	0
16381	4320013	katG	1.617	0.853	0	0
4192	4320137	katG	1.195	0.705	0	0
8066	4320551	katG	1.567	0.720	0	0
17699	4320710	katG	1.232	0.723	0	0
6016	4321112	katG	1.674	0.828	0	0
4802	4321269	katG	1.435	0.833	0	0
12887	4321489	katG	1.488	1.000	0	0
18666	4321720	katG	1.388	0.816	0	0
8828	4321831	yijF	1.684	1.050	0	0
10352	4321990	yijF	1.641	0.943	0	0
17873	4322487	yijF	1.309	0.820	0	0
17406	4322626	yijF	1.522	0.921	0	0
16484	4322981	gldA	1.957	1.215	0	0
10307	4323137	gldA	1.433	1.058	0	0
5381	4323344	gldA	1.524	0.700	0	0
16686	4323679	gldA	1.210	0.784	0	0
13927	4323815	gldA, talC	1.762	0.897	0	0
19062	4323891	gldA, talC	1.762	0.791	0	0

3362	4324177	gldA, talC	1.699	0.870	0	0
3398	4324457	talC	1.387	0.775	0	0
4666	4324707	talC, ptsA	1.007	0.678	0	0
1375	4324850	talC, ptsA	1.381	0.897	0	0
2767	4325210	ptsA	1.076	0.788	0	0
16114	4325437	ptsA	1.525	0.844	0	0
10444	4325521	ptsA	1.228	0.605	0	0
10267	4325615	ptsA	1.989	0.879	0	0
14689	4326109	ptsA	1.346	0.794	0	0
13923	4326217	ptsA	0.758	0.627	0	0
21144	4326458	ptsA	1.290	0.712	0	0
19944	4326744	ptsA	1.137	0.707	0	0
21982	4327024	ptsA, frwC	1.216	0.601	0	0
4503	4327167	ptsA, frwC	1.025	0.604	0	0
17052	4327274	ptsA, frwC	1.247	0.651	0	0
9536	4327652	frwC	1.074	0.675	0	0
9440	4327885	frwC	1.156	0.638	0	0
1770	4328188	frwC, frwB	1.218	0.610	0	0
6558	4328334	frwC, frwB	1.903	0.771	0	0
19368	4328568	frwB, pflD	1.263	0.712	0	0
1774	4328819	pflD	1.208	0.676	0	0
13886	4329137	pflD	1.535	0.832	0	0
1731	4329246	pflD	1.226	0.751	0	0
21472	4329540	pflD	1.330	0.774	0	0
8450	4329683	pflD	1.139	0.795	0	0
5887	4329938	pflD	1.028	0.985	0	0
21469	4330132	pflD	0.644	0.607	0	0
3487	4330216	pflD	1.185	0.913	0	0
12140	4330521	pflD	1.536	0.992	0	0
14623	4330792	pflD, pflC	1.431	1.298	0	0
16824	4330919	pflD, pflC	0.797	0.894	0	0
6340	4331365	pflC	1.119	0.951	0	0
22354	4331557	pflC, frwD	0.773	0.870	0	0
6962	4331743	pflC, frwD	1.331	0.912	0	0
11890	4332069	frwD	1.212	1.288	0	0
16237	4332283	yijO	0.891	1.247	0	0

2130	4332449	yijO	1.136	1.332	0	0
15789	4332523	yijO	1.216	1.381	0	0
10700	4332836	yijO	1.073	1.505	0	0
10390	4333160	yijO	0.894	1.824	0	0
2068	4333276	yijO	0.704	1.579	0	0
10403	4333467	yijO, yijP	0.809	1.565	0	0
19216	4333727	yijP	0.965	1.356	0	0
11621	4333976	yijP	0.946	1.105	0	0
13183	4334052	yijP	0.960	1.338	0	0
20636	4334472	yijP	0.658	0.833	0	0
17750	4334685	yijP	1.070	1.370	0	0
745	4334919	yijP	1.116	1.911	0	0
12404	4335004	yijP	0.896	1.708	0	1
12205	4335377	yijP, ppc	1.191	1.639	0	1
3867	4335481	yijP, ppc	0.971	1.369	0	0
3216	4335899	ppc	1.261	1.370	0	0
18230	4335973	ppc	1.160	1.317	0	0
14829	4336281	ppc	1.027	1.105	0	0
18116	4336593	ppc	1.124	1.170	0	0
5803	4336812	ppc	1.078	1.284	0	0
8740	4337029	ppc	1.103	1.211	0	0
5010	4337195	ppc	1.207	1.213	0	0
10853	4337391	ppc	1.183	1.318	0	0
4497	4337700	ppc	1.050	1.372	0	0
20308	4337893	ppc	0.879	1.260	0	0
7904	4338025	ppc	1.373	1.222	0	0
10469	4338431	argE	1.231	0.887	0	0
14663	4338587	argE	1.050	0.752	0	0
6988	4338687	argE	1.010	0.712	0	0
18297	4339061	argE, argC	0.704	0.697	0	0
7976	4339265	argE, argC	0.778	0.752	0	0
18794	4339385	argE, argC	0.776	0.903	0	0
6588	4339609	argE, argC	0.880	0.875	0	0
16428	4339839	argE, argC	0.969	0.936	0	0
13479	4340094	argC, argB	1.121	0.706	0	0
11223	4340379	argC, argB	1.381	0.745	0	0

17000	4340471	argC, argB	1.642	0.799	0	0
19326	4340656	argB	0.895	0.658	0	0
11684	4341133	argB, argH	1.396	1.125	0	0
355	4341379	argH	1.160	0.939	0	0
5608	4341538	argH	1.208	0.888	0	0
1445	4341662	argH	1.115	0.865	0	0
13198	4341742	argH	1.108	0.842	0	0
5455	4342170	argH	1.349	0.953	0	0
2642	4342339	argH	1.453	0.992	0	0
15220	4342642	argH, oxyR	1.223	1.017	0	0
17067	4342979	oxyR	1.268	1.014	0	0
12055	4343053	oxyR	1.034	1.094	0	0
14681	4343360	oxyR	0.994	1.443	0	0
1875	4343540	oxyR	0.859	1.415	0	0
13418	4343646	oxyR	0.726	1.615	0	0
7928	4344135	udhA	0.902	1.931	0	1
3019	4344274	udhA	0.732	1.708	0	1
12453	4344483	udhA	0.741	1.541	0	0
12294	4344690	udhA	0.962	1.474	0	0
21635	4344905	udhA	0.826	1.244	0	0
19072	4345136	udhA, yijC	0.928	1.307	0	0
1768	4345412	udhA, yijC	0.811	1.215	0	0
3515	4345547	udhA, yijC	0.801	1.224	0	0
14600	4345718	udhA, yijC	0.892	1.531	0	0
8373	4345958	yijC, yijD	0.915	1.794	0	1
5573	4346176	yijC, yijD	1.091	1.994	0	1
2832	4346361	yijD	0.763	1.562	0	0
3881	4346732	trmA	0.738	1.431	0	0
10300	4346940	trmA	0.922	1.538	0	0
15978	4347215	trmA	0.659	0.918	0	0
7856	4347388	trmA	0.959	0.985	0	0
6806	4347698	trmA, btuB	0.700	1.227	0	0
19477	4347771	trmA, btuB	0.766	1.776	0	0
8748	4348037	trmA, btuB	0.773	1.994	0	0
19413	4348350	btuB	0.753	1.140	0	0
18817	4348580	btuB	1.007	1.016	0	0

19384	4348783	btuB	0.839	1.038	0	0
7371	4348950	btuB	0.932	1.147	0	0
13087	4349363	btuB	0.966	1.072	0	0
9249	4349461	btuB, murI	1.120	1.116	0	0
8794	4349683	btuB, murI	0.674	1.094	0	0
13624	4349878	btuB, murI	0.626	1.252	0	0
20189	4350045	murI	0.729	1.585	0	0
17010	4350319	murI	0.707	1.878	0	1
13001	4350678	murI, rrsB	0.535	3.396	0	1
19736	4350751	rrsB	0.486	3.819	0	1
9269	4350980	rrsB	0.349	3.720	0	1
5009	4351111	rrsB	0.365	4.101	0	1
18971	4351556	rrsB	0.381	5.220	0	1
3125	4351779	rrsB	0.352	5.306	0	1
19219	4351907	rrsB	0.350	5.861	0	1
6638	4352083	rrsB	0.341	6.664	0	1
14858	4352433	rrsB, ileU	0.351	5.359	0	1
13270	4352654	ileU, STM4134	0.348	4.555	0	1
4349	4352981	rrlB	0.385	4.052	0	1
9785	4353148	rrlB	0.355	4.455	0	1
14802	4353323	rrlB	0.375	3.958	0	1
18765	4353611	rrlB	0.404	4.703	0	1
5093	4353774	rrlB	0.390	5.346	0	1
21136	4353884	rrlB	0.340	4.581	0	1
11379	4354242	rrlB	0.376	5.849	0	1
20336	4354500	rrlB	0.332	4.913	0	1
3182	4354736	rrlB	0.340	3.386	0	1
2618	4354851	rrlB	0.349	5.591	0	1
6695	4355045	rrlB	0.342	4.350	0	1
14565	4355329	rrlB	0.371	5.259	0	1
21045	4355427	rrlB	0.391	5.248	0	1
19398	4355696	rrlB	0.381	5.331	0	1
8008	4356051	rrlB, rrfB	0.399	4.480	0	1
2562	4356203	rrfB, murB	0.440	3.684	0	1
6905	4356596	murB	0.474	2.913	0	1
11558	4356695	murB	0.535	2.993	0	1

8021	4356784	murB	0.557	2.313	0	1
1508	4357133	murB	0.977	1.638	0	0
5757	4357359	murB, birA	0.834	1.116	0	0
1951	4357604	murB, birA	0.644	0.991	0	0
12556	4357924	birA	1.102	1.102	0	0
19208	4358056	birA	1.114	0.875	0	0
20874	4358443	birA	3.251	1.078	1	0
6852	4358659		2.937	1.059	1	0
1346	4358844	coaA	2.984	0.979	1	0
15155	4358973	coaA	2.977	1.018	1	0
16598	4359165	coaA	2.057	1.170	0	0
18122	4359493	coaA	0.791	1.720	0	0
15883	4359732	coaA, thrU	0.544	1.602	0	0
16372	4359817	coaA, thrU	0.462	2.512	0	1
19748	4360037	coaA, STM4141	0.358	3.677	0	1
13658	4360272	STM4141, glyT	0.413	3.873	0	1
20858	4360549	tufB	0.387	4.227	0	1
11813	4360763	tufB	0.423	3.307	0	1
16557	4361024	tufB	0.396	3.564	0	1
7480	4361302	tufB	0.515	3.117	0	1
3155	4361517	tufB, secE	0.472	3.058	0	1
3253	4361770	secE	0.528	3.311	0	1
20444	4361985	secE, nusG	0.476	2.879	0	1
17987	4362064	secE, nusG	0.437	3.282	0	1
12624	4362487	nusG	0.493	1.914	0	0
6025	4362573	nusG	0.601	2.211	0	1
21284	4362842	nusG, rplK	0.390	3.006	0	1
5329	4363081	rplK, rplA	0.517	2.622	0	1
13386	4363202	rplK, rplA	0.500	2.814	0	1
7537	4363524	rplA	0.469	1.703	0	0
21739	4363819	rplA	0.531	2.068	0	1
5574	4364058	rplA	0.581	2.561	0	1
18138	4364281	rplJ	0.433	2.417	0	1
10222	4364554	rplJ	0.397	2.210	0	1
10820	4364648	rplJ	0.408	2.479	0	1
11852	4364857	rplJ, rplL	0.424	3.013	0	1

12429	4365226	rplL	0.355	1.790	0	0
10164	4365370	rplL	0.436	2.259	0	0
8810	4365689	rpoB	0.421	2.034	0	0
11198	4365894	rpoB	0.515	1.753	0	0
16036	4365987	rpoB	0.521	1.670	0	0
6211	4366368	rpoB	0.672	1.493	0	0
17050	4366545	rpoB	0.528	1.073	0	0
16426	4366752	rpoB	0.739	1.435	0	0
20476	4367046	rpoB	0.710	1.444	0	0
21005	4367140	rpoB	0.844	1.519	0	0
14241	4367442	rpoB	0.459	1.084	0	0
8848	4367697	rpoB	0.750	1.547	0	0
8780	4367782	rpoB	0.543	1.218	0	0
6590	4368148	rpoB	0.576	1.113	0	0
19321	4368333	rpoB	0.820	1.627	0	0
16060	4368599	rpoB	0.803	1.450	0	0
2239	4368722	rpoB	0.757	1.291	0	0
6006	4369114	rpoB	0.968	1.815	0	0
12516	4369236	rpoB	0.625	1.084	0	0
8744	4369579	rpoB, rpoC	0.779	1.355	0	0
12444	4369777	rpoB, rpoC	0.864	1.399	0	0
19791	4369912	rpoC	0.615	0.931	0	0
14187	4370000	rpoC	0.844	1.384	0	0
10899	4370448	rpoC	0.768	1.137	0	0
12007	4370642	rpoC	0.778	1.485	0	0
8540	4370798	rpoC	0.776	1.354	0	0
19112	4370965	rpoC	0.850	1.385	0	0
6276	4371201	rpoC	0.840	1.201	0	0
9556	4371493	rpoC	0.530	0.659	0	0
17376	4371812	rpoC	0.831	1.155	0	0
15086	4371927	rpoC	1.007	1.296	0	0
10213	4372283	rpoC	0.824	1.215	0	0
2032	4372393	rpoC	0.709	1.100	0	0
10984	4372638	rpoC	0.782	1.405	0	0
8275	4372858	rpoC	0.837	1.209	0	0
13233	4373091	rpoC	0.882	1.330	0	0

19416	4373315	rpoC	0.917	1.350	0	0
12436	4373484	rpoC	1.009	1.148	0	0
14827	4373714	rpoC	1.317	1.133	0	0
11020	4373914	rpoC, STM4155	1.307	0.841	0	0
4383	4374337	STM4155	2.112	0.507	0	0
16268	4374599	STM4156	3.560	0.549	1	0
8729	4374775	STM4156	3.379	0.576	1	0
10459	4374929	STM4156, STM4157	2.712	0.564	1	0
22064	4375118	STM4156, STM4157	2.245	0.399	0	0
8793	4375452	STM4157	2.109	0.489	0	0
21868	4375626	STM4157	2.445	0.600	0	0
11928	4375776	STM4157	2.297	0.472	0	0
10168	4376040	STM4157	3.069	0.619	1	0
327	4376284	STM4157, STM4158	3.071	0.521	1	0
8173	4376474	STM4158	3.724	0.544	1	0
7139	4376594	STM4158	3.791	0.628	1	0
5518	4376947		2.109	0.752	1	0
20832	4377282	thiH	1.496	0.879	0	0
3796	4377371	thiH	0.989	0.598	0	0
4526	4377585	thiH	1.114	0.695	0	0
14890	4377861	thiH	1.213	0.604	0	0
5599	4378106	thiH	1.622	0.733	0	0
6299	4378406	thiH, thiG	1.354	0.736	0	0
20839	4378580	thiH, thiG	1.267	0.684	0	0
847	4378901	thiG, STM4161	1.341	0.634	0	0
9783	4379089	thiG, STM4161	1.425	0.700	0	0
17460	4379188	thiG, STM4161	1.478	0.720	0	0
18739	4379514	STM4161, thiF	1.685	0.712	0	0
11646	4379755	thiF	1.665	0.737	0	0
7726	4379833	thiF, thiE	1.430	0.738	0	0
2737	4380193	thiF, thiE	1.405	0.608	0	0
17060	4380331	thiE	1.118	0.804	0	0
19430	4380459	thiE	1.032	0.622	0	0
5971	4380719	thiE, thiC	1.642	0.816	0	0
16512	4380991	thiC	1.416	0.837	0	0
7502	4381247	thiC	1.146	0.781	0	0

14572	4381526	thiC	1.477	0.827	0	0
11300	4381630	thiC	1.100	0.629	0	0
9358	4382019	thiC	1.012	1.032	0	0
6231	4382155	thiC	0.811	1.239	0	0
21589	4382462	thiC	0.728	2.205	0	0
19410	4382646	thiC	0.806	1.712	0	0
9273	4382977	rsd, yjaD	1.055	1.207	0	0
20812	4383082	rsd, yjaD	1.097	1.127	0	0
17639	4383303	rsd, yjaD	1.064	0.860	0	0
8992	4383495	rsd, yjaD	0.881	0.835	0	0
15966	4383846	yjaD, hemE	0.735	0.774	0	0
18118	4384119	hemE	1.260	1.357	0	0
22316	4384273	hemE	1.152	1.294	0	0
13142	4384463	hemE	1.108	1.127	0	0
19899	4384796	hemE, nfi	1.097	1.410	0	0
11982	4384869	hemE, nfi	0.519	0.765	0	0
8280	4385062	hemE, nfi	0.642	1.371	0	0
17870	4385163	hemE, nfi	0.810	1.387	0	0
7409	4385360	nfi	0.876	1.103	0	0
12668	4385901	yjaG	0.860	1.429	0	0
15753	4386110	yjaG	0.854	1.578	0	0
514	4386276	yjaG, hupA	1.061	1.553	0	0
8734	4386487	yjaG, hupA	0.563	1.645	0	0
19284	4386709	hupA, yjaH	0.601	2.266	0	1
16991	4386963	yjaH	0.700	2.148	0	1
6238	4387233	yjaH	1.101	1.593	0	0
11334	4387499	yjaH	0.947	1.925	0	1
16332	4387638	yjaH	0.947	1.765	0	1
9337	4387864	zraP	0.784	1.249	0	0
3429	4388170	zraP, hydH	0.727	1.149	0	0
8116	4388432	zraP, hydH	1.026	1.316	0	0
10475	4388503	zraP, hydH	0.951	1.233	0	0
7995	4388736	hydH	0.788	0.805	0	0
6519	4389125	hydH	0.985	1.036	0	0
18993	4389364	hydH, hydG	1.034	1.043	0	0
3924	4389437	hydH, hydG	1.255	1.170	0	0

3698	4389826	hydG	0.920	1.021	0	0
8638	4389940	hydG	1.130	1.242	0	0
16112	4390136	hydG	1.440	1.235	0	0
20047	4390282	hydG	1.046	1.122	0	0
14648	4390541	hydG	1.386	1.273	0	0
5719	4390612	hydG	1.298	1.265	0	0
19796	4391008	hydG	1.326	1.410	0	0
15674	4391114	hydG	1.112	1.472	0	0
2024	4391608	purD	0.948	1.472	0	0
17672	4391838	purD	1.189	1.515	0	0
7173	4392066	purD	0.983	0.887	0	0
5065	4392243	purD	0.972	0.803	0	0
19822	4392438	purD, purH	1.431	1.139	0	0
15977	4392719	purD, purH	1.209	0.992	0	0
1136	4392833	purD, purH	0.772	0.731	0	0
6821	4393214	purH	0.659	0.877	0	0
20883	4393304	purH	0.793	0.973	0	0
6652	4393526	purH	1.100	1.187	0	0
11702	4393727	purH	0.623	1.026	0	0
17299	4394039	purH	0.662	2.121	0	1
11343	4394318	purH, rrsE	0.430	3.121	0	1
3864	4394564	rrsE	0.520	4.165	0	1
11041	4394656	rrsE	0.362	4.153	0	1
7193	4394788	rrsE	0.395	4.256	0	1
21022	4395101	rrsE	0.397	5.803	0	1
6961	4395452	rrsE	0.353	5.722	0	1
6223	4395628	rrsE	0.326	6.186	0	1
3778	4395787	rrsE	0.311	5.618	0	1
14137	4396136	rrsE, gltV	0.388	5.031	0	1
18935	4396242	gltV, rrlE	0.362	5.358	0	1
12229	4396420	rrlE	0.333	4.051	0	1
17508	4396747	rrlE	0.336	3.783	0	1
8194	4397035	rrlE	0.390	4.819	0	1
15336	4397181	rrlE	0.364	5.002	0	1
16600	4397308	rrlE	0.352	5.253	0	1
14962	4397666	rrlE	0.358	5.761	0	1

3671	4397924	rrlE	0.454	5.851	0	1
15269	4398160	rrlE	0.365	3.978	0	1
13003	4398427	rrlE	0.335	4.935	0	1
12472	4398608	rrlE	0.397	5.632	0	1
18504	4398753	rrlE	0.365	4.932	0	1
15700	4398851	rrlE	0.399	4.720	0	1
21921	4399120	rrlE	0.351	5.377	0	1
12888	4399599	rrfE	0.379	4.704	0	1
9778	4399670	rrfE	0.592	3.748	0	1
2864	4399912		0.597	3.208	0	1
16966	4400213		0.628	1.429	0	0
6802	4400296	yjaB	0.575	1.315	0	0
10494	4400645	yjaB, metA	0.654	1.000	0	0
5087	4400732	yjaB, metA	0.681	0.767	0	0
12449	4401099	yjaB, metA	0.826	0.789	0	0
8586	4401338	metA	0.875	0.747	0	0
18254	4401440	metA	1.220	0.636	0	0
7097	4401744	aceB	1.787	0.616	0	0
10843	4402018	aceB	1.808	0.569	0	0
8184	4402247	aceB	1.951	0.573	0	0
5210	4402417	aceB	2.346	0.647	0	0
6798	4402512	aceB	1.851	0.601	0	0
4950	4402830	aceB	1.313	0.622	0	0
7975	4403170	aceB	1.172	0.555	0	0
4346	4403415	aceB, aceA	1.322	0.622	0	0
16883	4403665	aceA	1.456	0.685	0	0
19367	4403893	aceA	1.098	0.657	0	0
19516	4404034	aceA	1.200	0.660	0	0
22246	4404174	aceA	1.281	0.746	0	0
4869	4404592	aceA, aceK	1.399	0.712	0	0
3855	4404663	aceA, aceK	1.202	0.625	0	0
9244	4404841	aceA, aceK	1.195	0.825	0	0
7094	4405092	aceK	1.444	0.831	0	0
17262	4405234	aceK	0.502	0.358	0	0
13922	4405667	aceK	1.621	0.689	0	0
21683	4405739	aceK	1.464	0.839	0	0

6734	4405944	aceK	1.624	0.831	0	0
8633	4406267	aceK	1.322	0.873	0	0
5551	4406486	aceK	1.291	1.072	0	0
15044	4406717	aceK	1.234	1.247	0	0
13645	4407040	STM4186	0.855	1.129	0	0
21143	4407168	STM4186	1.282	1.506	0	0
16593	4407485	STM4186, iclR	0.926	1.023	0	0
13904	4407624	STM4186, iclR	0.742	1.004	0	0
6756	4407696	iclR	1.192	1.520	0	0
7485	4408069	iclR, metH	1.320	1.870	0	0
22350	4408299	iclR, metH	1.045	2.075	0	0
8680	4408566	metH	1.182	1.375	0	0
14155	4408861	metH	1.022	0.801	0	0
7846	4409088	metH	1.632	1.056	0	0
4918	4409201	metH	1.219	0.850	0	0
12829	4409418	metH	2.010	1.085	0	0
18821	4409651	metH	1.234	0.823	0	0
21968	4409853	metH	1.721	1.089	0	0
9192	4410191	metH	1.166	0.816	0	0
10013	4410322	metH	1.773	1.105	0	0
15449	4410619	metH	0.948	0.731	0	0
17791	4410866	metH	0.735	0.784	0	0
20089	4411134	metH	1.178	1.099	0	0
826	4411349	metH	1.340	1.181	0	0
4488	4411562	metH	0.822	0.738	0	0
20300	4411656	metH	1.397	1.152	0	0
2953	4412000	yjbB	1.142	0.893	0	0
18806	4412257	yjbB	1.073	0.928	0	0
2404	4412459	yjbB	0.886	0.838	0	0
12305	4412551	yjbB	1.004	0.818	0	0
18261	4412795	yjbB	1.177	0.750	0	0
18258	4413195	yjbB	1.520	0.923	0	0
724	4413323	yjbB	1.798	0.793	0	0
11314	4413594	yjbB	1.294	0.922	0	0
11744	4413829	yjbB	1.267	1.023	0	0
13807	4413942		1.473	1.240	0	0

12244	4414184	pepE	1.424	0.867	0	0
11393	4414396	pepE, STM4191	1.631	0.963	0	0
8179	4414680	pepE, STM4191	1.933	0.828	0	0
20186	4414871	pepE, STM4192	1.962	0.827	0	0
18257	4415037	pepE, STM4192	2.133	0.719	0	0
13675	4415441	yjbC	1.616	0.799	0	0
7149	4415670	yjbC	1.615	0.930	0	0
19338	4415744	yjbC	1.332	0.877	0	0
13595	4416020	yjbC	0.996	1.056	0	0
14822	4416156	yjbC	1.073	1.461	0	0
14961	4416465	yjbD	0.963	1.486	0	0
14262	4416739	yjbD, STM4195	0.658	0.802	0	0
14746	4417061	STM4195	1.000	0.819	0	0
12918	4417153	STM4195	1.001	0.753	0	0
19134	4417487	STM4195	1.528	0.809	0	0
8690	4417686	STM4195	2.448	0.722	1	0
18403	4417943	STM4195, STM4196	2.953	0.572	1	0
19264	4418041	STM4195, STM4196	3.571	0.540	1	0
14660	4418260	STM4196	3.374	0.547	1	0
9803	4418424	STM4196	2.945	0.457	1	0
12598	4418894	STM4196, STM4197	3.548	0.548	1	0
3272	4419129	STM4197	2.623	0.438	0	0
11024	4419241	STM4197	3.171	0.573	0	0
20137	4419588	STM4198	2.282	0.563	0	0
13696	4419827	STM4198, STM4199	1.769	0.597	0	0
6498	4419936	STM4198, STM4199	2.524	0.598	1	0
6804	4420176	STM4198, STM4199	3.071	0.640	1	0
17988	4420353	STM4199	3.680	0.619	1	0
6436	4420585	STM4199, STM4200	3.172	0.739	1	0
17223	4420913	STM4200	2.079	0.759	0	0
11187	4421018	STM4200	2.590	0.690	0	0
7886	4421133	STM4200	0.754	0.427	0	0
3444	4421294	STM4200	1.022	0.511	0	0
1228	4421649	STM4200	2.048	0.640	0	0
15983	4421955	STM4200	4.326	0.686	1	0
18069	4422145	STM4200	5.502	0.545	1	0

1468	4422233	STM4200, STM4201	5.489	0.585	1	0
19369	4422458	STM4200, STM4201	6.378	0.576	1	0
18265	4422912	STM4201, STM4202	2.706	0.709	1	0
5367	4423116	STM4201, STM4202	1.858	0.723	0	0
11778	4423392	STM4202	1.314	0.581	0	0
10590	4423603	STM4202	1.863	0.689	0	0
12600	4423895	STM4202, STM4203	1.546	0.618	0	0
19335	4424093	STM4202, STM4203	1.826	0.617	0	0
9665	4424308	STM4202, STM4203	1.081	0.568	0	0
16737	4424503	STM4203, STM4204	1.960	0.664	0	0
22206	4424773	STM4204	1.811	0.633	0	0
677	4424899	STM4204	1.561	0.746	0	0
19435	4425000	STM4204	1.568	0.670	0	0
22096	4425395	STM4204	1.922	0.572	0	0
17944	4425535	STM4204	2.161	0.504	0	0
13416	4425693	STM4204	2.418	0.541	0	0
10068	4426131	STM4204, STM4205	1.803	0.614	0	0
4111	4426204	STM4204, STM4205	1.431	0.636	0	0
21933	4426425	STM4204, STM4205	1.467	0.620	0	0
6585	4426765	STM4205	1.918	0.541	0	0
7629	4426967	STM4205, STM4206	2.288	0.522	1	0
4213	4427292	STM4205, STM4206	2.783	0.567	1	0
8374	4427418	STM4206	3.268	0.635	1	0
20420	4427588	STM4206, STM4207	1.551	0.724	0	0
10658	4427924	STM4207	1.515	0.573	0	0
9025	4428149	STM4207	1.089	0.648	0	0
7680	4428283	STM4207	1.408	0.621	0	0
21296	4428648	STM4207, STM4208	1.401	0.644	0	0
10276	4428868	STM4208	1.608	0.697	0	0
7841	4429091	STM4208	1.381	0.639	0	0
3350	4429279	STM4208	1.594	0.660	0	0
18326	4429543	STM4208, STM4209	1.633	0.709	0	0
20822	4429683	STM4208, STM4209	1.441	0.656	0	0
16705	4429872	STM4209, STM4210	1.486	0.715	0	0
11526	4430055	STM4210	1.467	0.662	0	0
10671	4430363	STM4210	1.630	0.634	0	0

10169	4430484	STM4210	2.083	0.799	0	0
12574	4430569	STM4210, STM4211	1.527	0.773	0	0
15303	4431000	STM4211	2.463	0.785	0	0
678	4431186	STM4211	2.073	0.883	0	0
995	4431501	STM4211	1.850	0.715	0	0
4302	4431680	STM4211	1.607	0.803	0	0
19693	4431928	STM4211	1.184	0.674	0	0
5589	4432291	STM4211	1.567	0.649	0	0
22109	4432483	STM4211	1.720	0.781	0	0
4852	4432589	STM4211	1.332	0.619	0	0
8355	4432858	STM4211	1.746	0.706	0	0
20074	4433129	STM4211	2.474	0.666	0	0
14606	4433349		3.501	0.627	1	0
735	4433481	STM4212	5.078	0.716	1	0
19552	4433742	STM4212	4.790	0.622	1	0
15068	4433966	STM4212, STM4213	4.983	0.607	1	0
16212	4434207	STM4212, STM4213	2.778	0.696	1	0
8177	4434451	STM4213	1.576	0.674	0	0
4501	4434782	STM4213	0.993	0.547	0	0
4263	4434933	STM4213	1.236	0.691	0	0
17647	4435066	STM4213	1.441	0.771	0	0
500	4435388	STM4213, STM4214	1.689	0.622	0	0
2943	4435574	STM4213, STM4214	1.880	0.576	0	0
7343	4435881	STM4214, STM4215	4.085	0.460	1	0
20192	4436049	STM4215	4.903	0.520	1	0
19095	4436200	STM4215, STM4216	4.454	0.569	1	0
6132	4436476	STM4216	3.221	0.529	1	0
14323	4436744	STM4216, STM4217	1.942	0.620	0	0
21516	4437069	STM4217	1.497	0.448	0	0
12077	4437281	STM4217, STM4218	3.547	0.566	1	0
13213	4437418	STM4217, STM4218	3.882	0.588	1	0
12359	4437588	STM4218	2.083	0.440	0	0
1799	4437947	STM4219	3.284	0.539	1	0
19609	4438149	STM4219	3.761	0.781	1	0
13623	4438239	STM4219	3.099	0.863	1	0
8958	4438463	lysC	1.676	1.229	0	0

8985	4438557	lysC	1.463	0.973	0	0
493	4438789	lysC	1.389	1.110	0	0
16224	4439042	lysC	1.281	1.096	0	0
19962	4439554	lysC	1.103	0.837	0	0
14336	4439787	lysC, pgi	1.659	1.085	0	0
2027	4439923	lysC, pgi	1.497	0.926	0	0
15180	4440122	lysC, pgi	1.466	0.902	0	0
6362	4440204	lysC, pgi	1.357	0.811	0	0
11449	4440667	pgi	1.167	0.968	0	0
5772	4440914	pgi	0.927	0.904	0	0
21842	4441126	pgi	1.200	0.920	0	0
12792	4441314	pgi	1.554	0.905	0	0
8114	4441389	pgi	1.695	0.879	0	0
19894	4441729	pgi	3.791	0.763	1	0
19732	4441964	yjbE	6.065	0.713	1	0
19665	4442215	yjbE, yjbF	5.060	0.596	1	0
22152	4442529	yjbF	2.183	0.641	1	0
14105	4442669	yjbF	1.805	0.612	0	0
12609	4442981	yjbF, yjbG	1.816	0.693	0	0
9213	4443153	yjbF, yjbG	1.873	0.665	0	0
16412	4443258	yjbG	1.731	0.713	0	0
19141	4443521	yjbG, yjbH	1.235	0.446	0	0
8854	4443883	yjbG, yjbH	1.578	0.608	0	0
12352	4444078	yjbH	1.454	0.671	0	0
18194	4444233	yjbH	1.323	0.655	0	0
6488	4444447	yjbH	1.270	0.634	0	0
5597	4444598	yjbH	1.669	0.920	0	0
8003	4444828	yjbH	1.393	0.992	0	0
2718	4445157	yjbH	1.207	0.773	0	0
21993	4445246	yjbH	0.876	0.724	0	0
12142	4445460	yjbH	0.740	0.632	0	0
9815	4445632	yjbH	1.158	0.754	0	0
5550	4445973	yjbH, yjbA	1.341	0.923	0	0
19710	4446335	yjbA	0.797	0.579	0	0
7366	4446521	yjbA	1.157	0.908	0	0
3048	4446657		0.852	0.938	0	0

16819	4447067	malG	1.191	1.716	0	0
19798	4447244	malG	1.111	1.579	0	0
2191	4447500	malG	0.995	1.631	0	0
11003	4447590	malG	1.117	1.513	0	0
15691	4447848	malG, malF	1.444	1.887	0	0
7093	4448162	malF	1.687	1.636	0	0
1334	4448403	malF	1.412	1.576	0	0
18650	4448477	malF	1.225	1.575	0	0
13156	4448822	malF	1.215	1.523	0	0
18417	4449006	malF	0.862	1.137	0	0
5770	4449170	malF	0.605	0.860	0	0
18713	4449568	malF, malE	0.719	0.734	0	0
4016	4449810	malE	1.190	1.266	0	0
10861	4449886	malE	1.111	1.261	0	0
10608	4450140	malE	0.904	1.079	0	0
18800	4450380	malE	1.051	1.301	0	0
7443	4450543	malE, malK	0.907	0.998	0	0
670	4450889	malE, malK	1.133	1.297	0	0
16292	4451114	malK	1.171	1.787	0	0
18857	4451189	malK	1.070	1.510	0	0
4646	4451399	malK	1.210	2.049	0	0
2182	4451774	malK, lamB	1.144	2.380	0	0
20542	4451981	lamB	1.213	1.746	0	0
14079	4452231	lamB	1.139	1.742	0	0
1516	4452470	lamB	0.837	1.483	0	0
6901	4452618	lamB	1.097	1.572	0	0
12681	4452804	lamB	1.089	1.416	0	0
8090	4453044	lamB	1.152	1.237	0	0
19376	4453287	lamB, malM	1.142	1.550	0	0
2746	4453565	malM	1.224	1.646	0	0
17017	4453733	malM	0.976	1.696	0	1
17248	4454060	malM	1.053	1.835	0	1
13090	4454352	malM, ubiC	1.118	1.716	0	0
11043	4454565	ubiC	0.933	1.561	0	0
10908	4454649	ubiC	1.022	1.485	0	0
2824	4454758	ubiC, ubiA	0.759	1.430	0	0

11072	4455039	ubiC, ubiA	0.581	0.974	0	0
6185	4455398	ubiA	1.135	1.754	0	0
15596	4455688	ubiA	0.736	1.312	0	0
14389	4455804	ubiA	1.042	1.859	0	1
955	4455968	ubiA	1.048	2.107	0	1
1158	4456210	plsB	0.861	1.668	0	1
14232	4456286	plsB	0.901	1.733	0	1
14599	4456734	plsB	1.149	1.474	0	0
9576	4456896	plsB	1.105	1.206	0	0
2038	4457295	plsB	0.404	0.722	0	0
8732	4457496	plsB	1.324	1.463	0	0
14805	4457649	plsB	1.011	1.122	0	0
9754	4457765	plsB	0.962	1.252	0	0
21225	4458138	plsB	1.074	1.642	0	0
5199	4458254	plsB	0.975	1.431	0	0
11627	4458629	plsB, dgkA	0.977	1.270	0	0
11543	4458909	plsB, dgkA	0.602	0.876	0	0
14582	4459096	dgkA, lexA	0.625	1.012	0	0
9161	4459180	lexA	0.851	1.481	0	0
21775	4459596	lexA, dinF	0.806	1.972	0	1
3627	4459823	lexA, dinF	0.744	1.883	0	1
8846	4459928	dinF	0.819	1.988	0	1
14481	4460008	dinF	0.820	2.054	0	1
10254	4460191	dinF	0.550	0.871	0	0
9128	4460616	dinF	1.251	1.156	0	0
14905	4460821	dinF	1.353	1.313	0	0
4512	4461084	dinF, STM4239	0.961	1.005	0	0
9457	4461331	STM4239, yjbJ	1.553	1.321	0	0
5975	4461609	yjbJ	1.078	1.195	0	0
16716	4461697		0.912	1.299	0	0
18446	4462050	zur	1.072	1.521	0	0
4416	4462296	zur, STM4242	1.078	1.524	0	0
9088	4462458	zur, STM4242	1.251	1.303	0	0
7621	4462540	zur, STM4242	1.246	1.067	0	0
9239	4462784	STM4242	1.536	1.021	0	0
7863	4463070	STM4242	1.173	0.814	0	0

9078	4463311	STM4242	0.809	0.709	0	0
19176	4463501	STM4242, yjbN	0.912	0.792	0	0
13088	4463909	yjbN	0.893	1.676	0	0
10610	4464110	yjbN	1.016	1.239	0	0
19164	4464369	yjbN	0.915	0.932	0	0
3323	4464460	yjbN	1.052	1.015	0	0
17844	4464771	yjbN, yjbO	0.876	1.050	0	0
19615	4464929	yjbO	0.880	1.227	0	0
7882	4465099	yjbO	0.731	1.598	0	0
4713	4465376		0.687	2.817	0	1
16897	4465449		0.707	2.807	0	1
13781	4465725	qor	0.860	2.282	0	1
18499	4466109	qor, dnaB	1.268	1.162	0	0
19886	4466368	qor, dnaB	1.081	1.253	0	0
6464	4466522	qor, dnaB	1.062	1.371	0	0
4973	4466674	qor, dnaB	0.855	1.041	0	0
20202	4466787	qor, dnaB	0.610	0.837	0	0
1108	4467217	dnaB	0.946	1.440	0	0
18745	4467486	dnaB	0.559	0.871	0	0
8018	4467597	dnaB, alr	0.891	1.105	0	0
9413	4467884	dnaB, alr	1.054	1.197	0	0
1673	4467985	alr	1.213	1.529	0	0
21221	4468292	alr	0.832	1.108	0	0
2629	4468550	alr	1.053	1.245	0	0
12465	4468719	alr	0.986	1.236	0	0
20488	4469014	alr, tyrB	0.926	1.224	0	0
4541	4469226	tyrB	0.909	1.436	0	0
3158	4469385	tyrB	1.237	1.438	0	0
17437	4469494	tyrB	0.694	0.779	0	0
14442	4469896	tyrB	1.120	0.855	0	0
22098	4470003	tyrB	1.245	1.120	0	0
10207	4470313	tyrB, aphA	1.114	1.145	0	0
12378	4470529	aphA	0.950	0.943	0	0
8441	4470711	aphA	0.975	1.134	0	0
1372	4470980	aphA	1.008	0.655	0	0
2430	4471188	aphA, yjbQ	1.138	0.889	0	0

8545	4471456	yjbQ, yjbR	0.923	1.076	0	0
6398	4471563	yjbQ, yjbR	0.953	1.219	0	0
4259	4471905	yjbQ, yjbR	1.043	1.138	0	0
11116	4472019	yjbR	1.294	1.248	0	0
396	4472206	yjbR	1.060	1.236	0	0
17063	4472675	STM4252, STM4253	1.228	1.008	0	0
15039	4472776	STM4252, STM4253	1.620	1.038	0	0
8487	4473120	STM4253	1.329	0.806	0	0
12742	4473332	STM4253, uvrA	1.085	0.909	0	0
21192	4473651	uvrA	1.454	1.038	0	0
20958	4473750	uvrA	1.712	1.138	0	0
19081	4473965	uvrA	1.468	0.844	0	0
17900	4474121	uvrA	1.139	0.876	0	0
2551	4474392	uvrA	1.368	0.905	0	0
8270	4474688	uvrA	1.736	0.880	0	0
12420	4474934	uvrA	1.291	0.757	0	0
20196	4475037	uvrA	1.442	1.028	0	0
10103	4475396	uvrA	1.098	1.109	0	0
14244	4475517	STM4255, uvrA	0.863	1.222	0	0
18922	4475733	STM4255, uvrA	0.979	1.315	0	0
5274	4476064	STM4255, uvrA	0.824	2.740	0	1
5926	4476198	uvrA, ssb	0.749	3.492	0	1
11426	4476490	ssb	1.354	2.609	0	1
18505	4476832		2.814	0.903	1	0
20377	4476903		3.150	0.813	1	0
6090	4477130		2.880	0.481	1	0
5687	4477509	STM4257	2.843	0.525	1	0
2089	4477705	STM4257	3.088	0.587	1	0
11971	4477879	STM4257	2.441	0.490	0	0
19731	4478079	STM4257, STM4258	2.657	0.553	1	0
7809	4478312	STM4257, STM4258	2.378	0.467	1	0
6342	4478506	STM4258	2.205	0.398	0	0
9718	4478766	STM4258	2.390	0.542	0	0
2049	4479051	STM4258	2.018	0.442	0	0
9530	4479297	STM4258	2.559	0.510	1	0
19067	4479448	STM4258, STM4259	2.480	0.443	1	0

13945	4479733	STM4258, STM4259	2.356	0.398	0	0
20449	4479926	STM4259	2.444	0.553	1	0
5546	4480087	STM4259	2.459	0.484	1	0
18132	4480332	STM4259	1.628	0.278	0	0
1230	4480670	STM4259	2.221	0.450	0	0
5326	4480821	STM4259, STM4260	2.349	0.506	0	0
15020	4480947	STM4259, STM4260	2.307	0.381	0	0
12491	4481335	STM4260	2.137	0.440	0	0
518	4481421	STM4260	1.534	0.606	0	0
15886	4481616	STM4260	2.337	0.467	0	0
17659	4481914	STM4260	2.147	0.444	0	0
5339	4482114	STM4260, STM4261	2.809	0.435	1	0
9744	4482349	STM4260, STM4261	2.470	0.413	1	0
15448	4482731	STM4261	2.992	0.480	1	0
7706	4482925	STM4261	2.407	0.509	0	0
1948	4483137	STM4261	1.742	0.548	0	0
14305	4483442	STM4261	2.121	0.600	0	0
19352	4483622	STM4261	2.257	0.685	0	0
14449	4483893	STM4261	2.669	0.760	0	0
19036	4484136	STM4261	2.425	0.557	0	0
12666	4484209	STM4261	2.647	0.649	1	0
9113	4484473	STM4261	2.717	0.557	1	0
15506	4484630	STM4261	2.975	0.522	1	0
2570	4484871	STM4261	2.494	0.465	0	0
8829	4485072	STM4261	2.575	0.550	0	0
13452	4485352	STM4261	3.170	0.503	1	0
16997	4485627	STM4261	2.645	0.443	1	0
8812	4485732	STM4261	2.211	0.500	0	0
12789	4485985	STM4261	1.806	0.659	0	0
18901	4486056	STM4261	1.567	0.630	0	0
5076	4486338	STM4261	1.469	0.741	0	0
6601	4486756	STM4261	1.459	0.996	0	0
18606	4486982	STM4261	1.118	0.798	0	0
18868	4487132	STM4261	1.526	0.712	0	0
15173	4487348	STM4261	1.441	0.793	0	0
1204	4487595	STM4261	1.213	0.784	0	0

6501	4487942	STM4261	1.472	1.148	0	0
20424	4488117	STM4261	1.271	1.307	0	0
10162	4488199	STM4261	1.120	1.273	0	0
482	4488550	STM4261	1.718	0.972	0	0
18960	4488787	STM4261	1.357	0.856	0	0
8779	4489110	STM4261	1.307	0.806	0	0
12236	4489368	STM4261	1.192	0.775	0	0
3036	4489485	STM4261	0.629	0.663	0	0
1915	4489734	STM4261	1.544	0.989	0	0
9713	4489913	STM4261	0.614	0.429	0	0
6031	4490002	STM4261	1.547	0.850	0	0
15227	4490318	STM4261	1.518	0.855	0	0
7810	4490679	STM4261	1.565	0.827	0	0
15654	4490915	STM4261	0.937	0.632	0	0
20446	4490990	STM4261	1.485	1.014	0	0
3732	4491319	STM4261	1.465	0.739	0	0
18255	4491542	STM4261	1.210	0.712	0	0
19637	4491791	STM4261	1.283	0.763	0	0
15166	4491919	STM4261	1.151	0.778	0	0
623	4492228	STM4261	1.732	0.910	0	0
11658	4492463	STM4261	2.337	0.679	1	0
8700	4492759	STM4261	3.157	0.589	1	0
4780	4492848	STM4261	3.054	0.591	1	0
12480	4493050	STM4261	3.111	0.593	1	0
11485	4493329	STM4261	1.616	0.642	0	0
21345	4493543	STM4261	1.607	0.700	0	0
7855	4493623	STM4261	1.541	0.641	0	0
7476	4493965	STM4261	2.151	0.610	0	0
14937	4494251	STM4261	2.264	0.544	0	0
5681	4494545	STM4261	2.166	0.553	0	0
3205	4494792	STM4261	1.270	0.654	0	0
8157	4494876	STM4261	1.425	0.817	0	0
16331	4495064	STM4261	1.392	0.803	0	0
2084	4495360	STM4261	1.902	0.832	0	0
13734	4495628	STM4261	1.722	0.726	0	0
286	4495851	STM4261	1.843	0.765	0	0

6628	4496066	STM4261	1.828	0.642	0	0
7000	4496345	STM4261	1.684	0.598	0	0
20960	4496539	STM4261	1.752	0.557	0	0
272	4496778	STM4261	2.165	0.566	1	0
17024	4496910	STM4261	2.622	0.536	1	0
15852	4497057	STM4261	2.888	0.483	0	0
17438	4497360	STM4261	4.720	0.570	1	0
20351	4497580	STM4261	4.936	0.635	1	0
6260	4497875	STM4261	3.321	0.471	1	0
3419	4498016	STM4261	2.802	0.418	1	0
8741	4498125	STM4261	2.391	0.535	1	0
11521	4498623	STM4261	2.270	0.586	0	0
9276	4498851	STM4261, STM4262	2.963	0.487	1	0
10050	4499110	STM4261, STM4262	2.736	0.515	1	0
9317	4499324	STM4262	2.358	0.404	0	0
7593	4499404	STM4262	2.616	0.448	0	0
13894	4499703	STM4262	1.872	0.512	0	0
21220	4499918	STM4262	2.476	0.504	0	0
14911	4500134	STM4262	2.132	0.396	0	0
12485	4500377	STM4262	1.842	0.415	0	0
2445	4500600	STM4262	2.427	0.521	1	0
17561	4500840	STM4262	2.448	0.576	1	0
17430	4500927	STM4262	2.678	0.592	1	0
20036	4501098	STM4262	2.207	0.542	0	0
15707	4501316		1.952	0.563	0	0
16176	4501702	yjcB	1.548	0.601	0	0
900	4501875	yjcB	1.391	0.633	0	0
2370	4502213	yjcB, yjcC	1.944	0.517	0	0
5485	4502341	yjcC	2.678	0.554	0	0
20725	4502468	yjcC	2.697	0.552	0	0
13611	4502985	yjcC	1.939	0.578	0	0
4415	4503058	yjcC	1.501	0.538	0	0
14864	4503373	yjcC	1.485	0.582	0	0
13909	4503472	yjcC	1.779	0.625	0	0
15486	4503647	yjcC	1.794	0.579	0	0
16803	4504131	soxS, soxR	1.386	0.738	0	0

8245	4504253	soxS, soxR	1.238	0.676	0	0
10288	4504459	soxS, soxR	1.347	0.717	0	0
21958	4504606	soxS, soxR	1.345	0.807	0	0
15822	4504893	STM4267	0.859	0.804	0	0
19530	4505077	STM4267	0.889	0.722	0	0
16080	4505428	STM4267	0.949	0.850	0	0
7610	4505541	STM4267	1.020	1.017	0	0
16783	4505907	yjcD	1.030	1.499	0	0
3168	4506079	yjcD	1.059	1.658	0	0
7954	4506357	yjcD	0.999	1.225	0	0
674	4506533	yjcD	1.273	1.246	0	0
19966	4506768	yjcD	1.278	0.876	0	0
19121	4506969	yjcD	1.008	0.692	0	0
11911	4507240	yjcD, yjcE	0.932	0.728	0	0
11653	4507330	yjcD, yjcE	1.416	1.073	0	0
7180	4507604	yjcE	1.065	0.726	0	0
3057	4507689	yjcE	1.222	0.820	0	0
19426	4508193	yjcE	0.889	0.923	0	0
16117	4508302	yjcE	0.577	0.644	0	0
2119	4508478	yjcE	0.886	0.604	0	0
4427	4508558	yjcE	0.701	0.639	0	0
20626	4509017	yjcE	1.404	1.152	0	0
16647	4509365	STM4270	1.266	1.299	0	0
12036	4509442	STM4270	0.696	0.935	0	0
11047	4509643	STM4270	0.616	0.725	0	0
603	4509892	STM4270, STM4271	1.630	1.153	0	0
266	4510123	STM4270, STM4271	1.763	1.111	0	0
6663	4510430	STM4270, STM4271	1.651	0.915	0	0
8399	4510655	STM4270, STM4271	1.188	0.676	0	0
1122	4510738	STM4271, STM4272	1.891	0.823	0	0
2883	4510905	STM4272	1.558	0.727	0	0
3780	4511114	STM4272	1.398	0.711	0	0
8981	4511502	yjcG	1.432	0.920	0	0
20135	4511661	yjcG	1.617	1.002	0	0
3417	4511859	yjcG	1.538	0.932	0	0
22192	4512190	yjcG	1.777	0.814	0	0

15390	4512435	yjcG	1.795	0.791	0	0
22045	4512708	yjcG	1.484	0.851	0	0
17323	4512989	yjcG	1.586	0.764	0	0
15721	4513139	yjcG, yjcH	1.611	0.806	0	0
793	4513431	yjcG, yjcH	1.470	0.848	0	0
3962	4513614	yjcH	0.484	0.660	0	0
20892	4513749	yjcH, acs	1.204	1.010	0	0
1531	4513974	acs	1.278	1.073	0	0
12137	4514177	acs	1.252	0.730	0	0
21052	4514431	acs	1.273	1.198	0	0
10519	4514758	acs	1.098	0.958	0	0
3123	4514899	acs	0.870	0.773	0	0
10128	4515018	acs	1.162	1.057	0	0
20459	4515330	acs	1.066	0.870	0	0
16458	4515649	acs, STM4276	1.036	0.657	0	0
7085	4515881	acs, STM4276	0.999	0.659	0	0
9141	4515980	acs, nrfA	0.992	0.545	0	0
4457	4516125	nrfA	0.869	0.576	0	0
8865	4516244	nrfA	0.964	0.689	0	0
4871	4516806	nrfA	0.897	0.641	0	0
16608	4516926	nrfA	1.049	0.766	0	0
2115	4517108	nrfA, nrfB	1.347	0.711	0	0
20368	4517500	nrfA, nrfB	1.405	0.693	0	0
14782	4517632	nrfB	0.954	0.722	0	0
14180	4517932	nrfB, nrfC	1.106	0.820	0	0
21254	4518187	nrfB, nrfC	0.875	0.841	0	0
11522	4518290	nrfC	1.350	0.893	0	0
22138	4518418	nrfC, nrfD	1.417	1.201	0	0
394	4518858	nrfD	1.276	1.121	0	0
21396	4519118	nrfD	0.630	0.754	0	0
13845	4519365	nrfD, nrfE	1.777	0.836	0	0
8143	4519531	nrfD, nrfE	1.255	0.756	0	0
3747	4519802	nrfD, nrfE	1.647	0.899	0	0
7663	4519916	nrfE	0.790	0.841	0	0
11952	4520041	nrfE	1.231	0.782	0	0
21511	4520209	nrfE	1.038	0.567	0	0

4638	4520665	nrfE	0.906	0.588	0	0
12941	4520919	nrfE	1.339	0.753	0	0
7503	4521052	nrfE	1.185	0.701	0	0
11840	4521232	nrfE	1.673	0.787	0	0
5324	4521369	nrfE	1.291	0.609	0	0
7416	4521648	nrfE, nrfG	1.083	0.641	0	0
2371	4521829	nrfE, nrfG	1.824	0.900	0	0
4525	4522045	nrfG	0.704	0.585	0	0
7164	4522314	nrfG	1.314	1.050	0	0
7419	4522675	gltP	0.886	0.639	0	0
20387	4522815	gltP	1.050	0.969	0	0
19700	4522981	gltP	0.910	0.931	0	0
19860	4523260	gltP	1.004	1.037	0	0
4091	4523571	gltP	0.929	0.991	0	0
18081	4523787	gltP	1.088	1.439	0	0
18880	4523986	gltP	0.858	1.121	0	0
22011	4524154	gltP	1.057	1.379	0	0
4183	4524474	yjcO	0.920	1.021	0	0
3167	4524564	yjcO	1.314	1.198	0	0
18588	4524889	yjcO	1.257	1.148	0	0
14142	4525145	yjcO	1.547	1.085	0	0
2407	4525455	yjcO, fdhF	0.937	0.864	0	0
14431	4525586	yjcO, fdhF	0.929	0.816	0	0
6433	4525842	fdhF	1.006	0.686	0	0
22177	4526042	fdhF	1.388	0.870	0	0
17174	4526199	fdhF	1.194	0.732	0	0
5112	4526393	fdhF	1.294	0.848	0	0
4957	4526683	fdhF	1.151	0.703	0	0
14500	4526974	fdhF	1.089	0.783	0	0
8726	4527128	fdhF	1.060	0.725	0	0
3526	4527334	fdhF	0.829	0.834	0	0
13179	4527539	fdhF	1.074	1.343	0	0
3795	4527847	fdhF, lpxO	1.400	1.013	0	0
4058	4528208	lpxO	1.108	0.779	0	0
1974	4528339	lpxO	0.861	0.814	0	0
19358	4528495	lpxO	1.090	0.918	0	0

3730	4528734	lpxO	0.962	0.895	0	0
16500	4529011	lpxO, phnO	1.157	0.887	0	0
20370	4529339	phnO	1.190	0.889	0	0
19328	4529466	phnO	0.893	0.789	0	0
15561	4529600	phnO, phnB	0.756	0.652	0	0
7259	4529935	phnB	1.050	0.836	0	0
14856	4530042	phnB	0.916	0.650	0	0
19504	4530153	phnB, phnA	1.080	0.788	0	0
9231	4530279	phnB, phnA	0.873	0.806	0	0
16139	4530451	phnB, phnA	1.010	0.898	0	0
7825	4531073	proP	0.665	0.788	0	0
18726	4531269	proP	1.140	1.299	0	0
17542	4531428	proP	1.049	1.185	0	0
1956	4531830	proP	0.717	1.481	0	0
432	4531993	proP	0.907	2.032	0	1
5135	4532228	proP	0.892	1.902	0	1
5369	4532360	proP	0.904	2.232	0	1
21111	4532586		0.673	2.332	0	1
6922	4532668	basS	0.298	0.742	0	0
9920	4533047	basS	0.790	1.418	0	0
16376	4533308	basS	0.970	1.827	0	1
3897	4533582	basS	0.922	1.913	0	1
1793	4533794	basS, basR	0.628	0.946	0	0
2241	4533973	basS, basR	0.887	1.715	0	0
3906	4534363	basR, yjdB	1.005	1.785	0	0
19161	4534528	basR, yjdB	1.271	1.503	0	0
5837	4534764	basR, yjdB	1.126	1.231	0	0
9071	4534907	yjdB	1.219	1.275	0	0
1771	4535120	yjdB	1.301	1.210	0	0
4922	4535400	yjdB	1.237	1.147	0	0
18089	4535689	yjdB	1.407	1.578	0	0
4995	4535892	yjdB	1.343	1.788	0	0
744	4535985	yjdB	1.092	1.385	0	0
5389	4536113	yjdB	0.963	1.027	0	0
15014	4536398	yjdB, yjdE	0.720	0.713	0	0
16809	4536523	yjdE	1.310	0.922	0	0

13996	4536862	yjdE	1.368	0.832	0	0
18190	4537111	yjdE	1.623	0.758	0	0
4031	4537507	yjdE	2.098	0.721	0	0
19772	4537578	yjdE	2.617	0.780	0	0
4137	4537737	yjdE, adiY	2.527	0.647	0	0
12566	4537825	yjdE, adiY	2.716	0.707	1	0
7024	4538385	adiY	2.525	0.571	1	0
4212	4538668	adiY, adi	1.891	0.594	0	0
15384	4538902	adi	1.645	0.724	0	0
5205	4539046	adi	1.427	0.720	0	0
8099	4539179	adi	1.209	0.756	0	0
3367	4539511	adi	1.387	0.776	0	0
17824	4539688	adi	0.846	0.598	0	0
20951	4539761	adi	0.814	0.763	0	0
3390	4540212	adi	1.446	0.801	0	0
14177	4540405	adi	1.715	0.814	0	0
12783	4540595	adi	1.089	0.605	0	0
14655	4540905	adi	1.334	0.684	0	0
18473	4541133	adi	0.940	0.666	0	0
15056	4541319	adi, melR	1.398	0.806	0	0
10247	4541396	adi, melR	1.219	0.788	0	0
428	4541486	melR	1.234	1.032	0	0
9668	4541886	melR	1.150	1.083	0	0
2846	4542105	melR, melA	1.359	0.957	0	0
4181	4542227	melR, melA	1.243	0.817	0	0
8227	4542405	melR, melA	1.410	0.787	0	0
3037	4542666	melA	0.617	0.634	0	0
6520	4543149	melA	1.680	0.683	0	0
4258	4543277	melA	1.371	0.757	0	0
10035	4543651	melA, melB	1.150	0.631	0	0
7550	4543835	melB	1.082	0.616	0	0
15176	4543906	melB	1.000	0.634	0	0
12787	4544153	melB	1.274	0.638	0	0
5569	4544495	melB	1.335	0.700	0	0
5481	4544566	melB	1.555	0.695	0	0
9753	4544888	melB	1.731	0.764	0	0

3533	4545091	melB	1.573	0.845	0	0
11962	4545447	fumB	1.490	0.846	0	0
15536	4545661	fumB	1.155	0.877	0	0
7297	4545749	fumB	1.518	1.104	0	0
10928	4545973	fumB	1.577	0.890	0	0
18295	4546378	fumB	1.564	0.876	0	0
2944	4546579	fumB	1.462	0.671	0	0
15043	4546777	fumB	1.132	0.645	0	0
14863	4546910	fumB	0.937	0.478	0	0
20936	4547002	fumB	1.613	0.713	0	0
8070	4547525	dcuB	1.572	0.579	0	0
7341	4547617	dcuB	0.598	0.544	0	0
18519	4547833	dcuB	1.699	0.690	0	0
11376	4548064	dcuB	2.186	0.886	0	0
20395	4548449	dcuB	3.747	0.892	1	0
1262	4548651	dcuB, STM4302	2.778	0.758	1	0
16536	4548732	dcuB, STM4302	3.404	0.686	1	0
17265	4548833	dcuB, STM4302	3.583	0.716	1	0
17538	4549282	STM4302, dcuR	2.475	0.772	0	0
5943	4549455	dcuR	1.928	0.888	0	0
434	4549729	dcuR	1.259	0.909	0	0
14257	4549894	dcuR, dcuS	1.376	0.856	0	0
12595	4550194	dcuR, dcuS	1.284	0.849	0	0
3720	4550407	dcuS	1.673	0.807	0	0
16665	4550514	dcuS	1.393	0.759	0	0
17920	4550854	dcuS	1.274	0.678	0	0
12779	4550925	dcuS	1.432	0.762	0	0
777	4551195	dcuS	2.128	0.811	0	0
5553	4551540	dcuS, STM4305	4.727	0.687	1	0
2437	4551744	dcuS, STM4305	4.813	0.490	1	0
10418	4551886	dcuS, STM4305	3.966	0.534	1	0
14016	4552092	STM4305	5.469	0.628	1	0
22161	4552398	STM4305	6.928	0.651	1	0
1056	4552634	STM4305	4.348	0.891	1	0
2822	4552751	STM4305	2.661	0.630	1	0
4167	4553144	STM4305	1.522	0.557	0	0

8565	4553347	STM4305	1.853	0.651	0	0
16579	4553487	STM4305	1.967	0.673	0	0
16284	4553661	STM4305	1.115	0.683	0	0
15104	4553914	STM4305, STM4306	1.814	0.717	0	0
2179	4554272	STM4305, STM4306	1.700	0.743	0	0
14051	4554489	STM4306, STM4307	1.415	0.711	0	0
9256	4554693	STM4306, STM4307	1.655	0.748	0	0
9701	4554846	STM4306, STM4307	1.621	0.700	0	0
19556	4555134	STM4307	1.521	0.798	0	0
13700	4555284	STM4307, STM4308	1.970	0.910	0	0
1818	4555685	STM4307, STM4308	1.167	0.634	0	0
13865	4555760	STM4308	1.537	0.798	0	0
1823	4556037	STM4308	1.543	0.760	0	0
569	4556330	STM4308	1.047	0.598	0	0
9601	4556608	STM4309	1.516	0.671	0	0
21706	4556698	STM4309	1.513	0.736	0	0
15783	4556954	STM4309	1.545	0.690	0	0
13551	4557211	STM4309	1.758	0.735	0	0
1940	4557510	STM4309	1.754	0.812	0	0
3758	4557688	STM4309, STM4310	2.434	0.674	1	0
1311	4557879	STM4309, STM4310	2.733	0.606	1	0
20427	4557951	STM4309, STM4310	3.940	0.590	1	0
7622	4558338	STM4310	2.784	0.632	1	0
10521	4558626	STM4310	2.041	0.757	0	0
9903	4558750	STM4310	1.953	0.607	0	0
291	4558959	STM4310, tnpA_6	2.489	0.594	0	0
15351	4559188	tnpA_6	2.057	0.486	0	0
20145	4559493	tnpA_6	1.291	0.678	0	0
3544	4559701	tnpA_6	1.221	0.831	0	0
6024	4559940		1.380	0.749	0	0
13598	4560010		1.570	0.717	0	0
10516	4560339	STM4312, STM4313	1.611	0.572	0	0
13163	4560533	STM4312, STM4313	1.772	0.489	0	0
345	4560863	STM4313, rtsB	1.948	0.504	0	0
455	4561067	rtsB, rtsA	2.428	0.606	0	0
862	4561221	rtsB, rtsA	1.591	0.610	0	0

18489	4561577	rtsA	2.784	0.539	1	0
3252	4561658	rtsA	2.861	0.666	1	0
21499	4562068	rtsA, STM4316	2.364	0.461	0	0
5501	4562268	STM4316, STM4317	2.485	0.581	0	0
1105	4562494	STM4316, STM4317	1.626	0.756	0	0
20904	4562740	STM4317, STM4318	1.239	0.834	0	0
21899	4562879	STM4318	1.044	0.912	0	0
18142	4563046	STM4318	1.044	0.741	0	0
11790	4563285	STM4318	0.656	0.732	0	0
1916	4563623	phoN	1.947	0.815	0	0
287	4563893	phoN	3.277	0.736	1	0
16417	4564077	phoN	3.291	0.671	1	0
14747	4564219	phoN	2.943	0.495	1	0
17568	4564513	phoN	2.939	0.436	1	0
20922	4564825		2.793	0.531	0	0
15773	4564916		2.520	0.564	0	0
16055	4565001		1.594	0.521	0	0
20205	4565395		0.993	0.610	0	0
16849	4565517	STM4320	0.813	0.717	0	0
18735	4565792	STM4320	0.835	0.805	0	0
2479	4565896	STM4320	0.644	0.755	0	0
692	4566331		0.601	2.651	0	1
15437	4566488		0.516	3.561	0	1
19633	4566706	pheR	0.495	2.102	0	0
14510	4566978	pheR, yjdC	0.650	1.274	0	0
4384	4567277	yjdC	0.985	1.361	0	0
8190	4567464	yjdC, dsbD	0.994	1.068	0	0
14637	4567607	yjdC, dsbD	1.043	1.083	0	0
9342	4567934	dsbD	1.487	1.207	0	0
1187	4568032	dsbD	1.271	1.107	0	0
6361	4568230	dsbD	0.806	0.835	0	0
14706	4568469	dsbD	1.275	1.223	0	0
2703	4568877	dsbD	1.158	0.994	0	0
22182	4569075	dsbD, cutA	1.230	1.130	0	0
2579	4569201	dsbD, cutA	0.919	1.076	0	0
11680	4569386	dsbD, cutA	1.153	1.215	0	0

683	4569581	cutA, dcuA	1.747	1.124	0	0
16206	4569929	dcuA	0.985	0.911	0	0
5425	4570137	dcuA	0.581	0.623	0	0
20072	4570313	dcuA	1.297	1.106	0	0
18541	4570522	dcuA	0.996	0.938	0	0
19683	4570907	dcuA	0.602	1.061	0	0
21445	4571136	dcuA, aspA	0.695	2.084	0	0
5942	4571329	aspA	0.501	1.143	0	0
10138	4571494	aspA	0.691	1.785	0	0
1873	4571816	aspA	0.677	1.582	0	0
11119	4571942	aspA	0.598	1.464	0	0
11049	4572275	aspA, fxsA	0.600	1.423	0	0
1060	4572489	aspA, fxsA	0.654	1.881	0	0
7477	4572699	aspA, fxsA	0.566	1.969	0	0
13315	4572793	aspA, fxsA	0.596	1.929	0	0
6543	4573163	fxsA	0.685	1.142	0	0
18627	4573416	yjeH	0.743	1.430	0	0
643	4573627	yjeH	1.035	1.060	0	0
2004	4573867	yjeH	0.902	1.124	0	0
3453	4574085	yjeH	0.617	0.993	0	0
13563	4574180	yjeH	0.898	1.331	0	0
17175	4574525	yjeH, mopB	0.545	1.555	0	0
21526	4574748	yjeH, mopB	0.516	2.431	0	1
5567	4574870	yjeH, mopB	0.525	2.339	0	1
9300	4575004	mopB, mopA	0.452	2.275	0	1
10609	4575250	mopA	0.487	2.419	0	1
17455	4575537	mopA	0.549	2.943	0	1
6212	4575884	mopA	0.534	2.703	0	1
8383	4576149	mopA	0.615	3.262	0	1
13197	4576242	mopA	0.608	2.882	0	1
6856	4576531	mopA, yjeI	0.535	2.372	0	1
8347	4576622	mopA, yjeI	0.416	2.130	0	1
6346	4576972	yjeI	0.758	1.962	0	1
16833	4577246	yjeI	0.928	1.641	0	1
8787	4577328		1.113	1.457	0	0
13907	4577563	yjeJ	1.998	1.039	0	0

10576	4577829	yjeJ	2.398	0.685	1	0
7506	4578131	yjeJ	3.895	0.738	1	0
17253	4578327	yjeJ	4.087	0.687	1	0
7524	4578404	yjeJ	3.100	0.755	1	0
4389	4578867	yjeK	1.340	0.950	0	0
11972	4579138	yjeK, efp	1.082	1.210	0	0
3047	4579337	yjeK, efp	1.020	1.877	0	1
15254	4579449	yjeK, efp	1.009	2.474	0	1
8542	4579815	yjeK, efp	0.361	0.786	0	0
9589	4579928	yjeK, efp	0.613	1.933	0	1
22162	4580149	ecnA, ecnB	0.786	1.747	0	1
21049	4580432	ecnB	0.753	1.196	0	0
2073	4580645	ecnR	1.036	1.227	0	0
4042	4580861	ecnR	0.935	1.265	0	0
6508	4581053	ecnR, sugE	0.980	1.299	0	0
13255	4581196	ecnR, sugE	0.841	1.369	0	0
11708	4581382	ecnR, sugE	0.904	2.007	0	1
14484	4581494	ecnR, sugE	0.788	2.009	0	1
4808	4581829	blc	1.077	1.454	0	0
11236	4582080	blc	1.123	1.035	0	0
9333	4582345	blc	0.891	0.923	0	0
16882	4582556	blc, frdD	0.803	0.810	0	0
16025	4582817	frdD	0.946	1.061	0	0
12839	4582899	frdD, frdC	0.694	0.732	0	0
7296	4583239	frdD, frdC	0.959	1.034	0	0
11989	4583327	frdC, frdB	1.071	1.056	0	0
12180	4583847	frdB	0.981	1.159	0	0
21196	4583931	frdB	0.992	1.377	0	0
4231	4584245	frdB, frdA	0.412	0.537	0	0
809	4584381	frdB, frdA	0.949	0.999	0	0
20254	4584786	frdA	1.300	1.312	0	0
4439	4584877	frdA	1.103	1.205	0	0
5070	4585074	frdA	1.065	1.058	0	0
10437	4585323	frdA	0.932	0.914	0	0
271	4585491	frdA	0.949	1.267	0	0
12146	4585857	frdA, yjeA	1.270	1.064	0	0

4110	4586048	frdA, yjeA	1.690	1.242	0	0
12903	4586285	yjeA	1.324	0.926	0	0
20468	4586392	yjeA	1.184	0.870	0	0
11703	4586678	yjeA	1.126	0.801	0	0
11437	4587041	yjeA, yjeM	2.083	0.753	0	0
4394	4587278	yjeM	2.470	0.626	1	0
8113	4587435	yjeM	3.260	0.631	1	0
13577	4587545	yjeM	3.287	0.559	1	0
13379	4587976	yjeM	3.018	0.618	1	0
11659	4588142	yjeM	2.925	0.602	1	0
7013	4588278	yjeM	2.289	0.648	0	0
8454	4588529	yjeM, yjeO	1.515	0.670	0	0
19792	4588879	yjeO	1.317	0.899	0	0
21307	4588950	yjeO	1.245	0.894	0	0
10354	4589144		1.144	0.962	0	0
13251	4589421	yjeP	1.225	1.140	0	0
3836	4589623	yjeP	1.057	1.081	0	0
16134	4589759	yjeP	1.350	1.148	0	0
11211	4590125	yjeP	1.435	1.155	0	0
2845	4590403	yjeP	0.966	0.834	0	0
2015	4590671	yjeP	1.318	1.186	0	0
2389	4590812	yjeP	1.050	1.166	0	0
10277	4591130	yjeP	1.262	1.144	0	0
16683	4591289	yjeP	0.839	0.940	0	0
20731	4591377	yjeP	1.289	1.228	0	0
22039	4591734	yjeP	1.179	1.410	0	0
14451	4591986	yjeP	1.244	1.601	0	0
7004	4592199	yjeP	1.060	1.072	0	0
16863	4592526	yjeP	1.007	1.172	0	0
6647	4592741	yjeP, psd	1.104	1.307	0	0
8978	4592817	yjeP, psd	0.762	1.001	0	0
19119	4593208	psd	0.907	1.386	0	0
14471	4593411	psd	0.850	1.436	0	0
8223	4593483	psd	1.038	1.297	0	0
22208	4593583	psd	0.969	1.238	0	0
12074	4594134	yjeQ	0.934	1.419	0	0

11945	4594228	yjeQ	0.638	0.949	0	0
3387	4594582	yjeQ, orn	0.847	1.871	0	1
21021	4594783	yjeQ, orn	0.906	2.091	0	1
5017	4594925	yjeQ, orn	0.871	1.740	0	0
6947	4595125	yjeQ, orn	0.973	1.433	0	0
11294	4595384	STM4351	0.616	1.129	0	0
20357	4595628	STM4351	0.841	1.164	0	0
453	4595919	STM4351, glyV	0.513	1.987	0	1
13438	4596141	STM4351, glyV	0.454	2.339	0	1
20715	4596286	STM4351, glyV	0.454	2.954	0	1
5708	4596488	STM4351, glyX	0.422	3.669	0	1
3069	4596726	glyY	0.379	3.287	0	1
14144	4597035		0.624	3.143	0	1
11668	4597138		0.651	2.906	0	1
13496	4597488	yjeS	0.993	1.336	0	0
11279	4597565	yjeS	0.923	1.224	0	0
13410	4597914	yjeF, yjeS	1.177	1.489	0	0
21093	4598119	yjeF, yjeS	0.985	1.890	0	1
11882	4598330	yjeF, yjeS	1.027	2.244	0	1
6744	4598511	yjeF, yjeS	0.521	1.174	0	0
20134	4598716	yjeF, yjeS	0.519	0.722	0	0
6651	4598951	yjeF	0.659	0.805	0	0
13166	4599365	yjeF	1.082	1.356	0	0
8313	4599516	yjeF, yjeE	0.973	1.437	0	0
19157	4599829	yjeF, yjeE	1.055	1.449	0	0
5748	4599939	yjeE, amiB	0.915	1.127	0	0
8987	4600085	yjeE, amiB	1.008	1.268	0	0
7530	4600346	amiB	1.491	1.224	0	0
20895	4600625	amiB	1.338	1.381	0	0
18439	4600817	amiB	0.701	0.739	0	0
13757	4601028	amiB	1.386	1.672	0	0
3270	4601216	amiB, mutL	1.017	1.308	0	0
12808	4601334	amiB, mutL	0.892	1.391	0	0
20941	4601742	mutL	0.783	1.081	0	0
21661	4601843	mutL	1.278	1.734	0	0
19185	4602189	mutL	1.308	1.743	0	0

18287	4602413	mutL	0.698	1.039	0	0
7998	4602579	mutL	1.233	1.772	0	0
3233	4602977	mutL	1.062	1.714	0	0
6778	4603198	mutL, miaA	0.923	1.330	0	0
9101	4603279	mutL, miaA	1.052	1.796	0	0
17132	4603638	miaA	0.760	2.416	0	1
2318	4603840	miaA	0.688	2.632	0	1
21594	4604051	miaA	0.677	2.537	0	1
6550	4604301	miaA, hfq	0.549	2.145	0	1
20633	4604556	hfq, hflX	0.548	1.993	0	1
14525	4604649	hfq, hflX	0.473	1.993	0	1
5626	4604926	hflX	0.546	2.297	0	1
13656	4605003	hflX	0.498	2.796	0	1
13104	4605266	hflX	0.585	3.105	0	1
21672	4605649	hflX	0.695	2.325	0	1
1313	4605853	hflX	0.594	2.020	0	0
18954	4606090	hflX, hflK	0.646	1.276	0	0
8951	4606363	hflK	0.651	2.551	0	0
20262	4606567	hflK	0.575	1.471	0	0
12115	4606779	hflK	0.500	1.476	0	0
12504	4607031	hflK	0.764	2.132	0	1
13035	4607218	hflK, hflC	0.844	1.939	0	1
14740	4607431	hflK, hflC	0.711	1.560	0	0
5358	4607724	hflC	0.631	1.680	0	0
12964	4607948	hflC	0.685	1.817	0	1
18280	4608042	hflC	0.701	1.909	0	1
478	4608331	hflC, yjeT	0.730	2.048	0	1
15786	4608666	hflC, yjeT	0.541	1.581	0	0
4280	4608781	yjeT, purA	0.531	1.711	0	0
21335	4608952	yjeT, purA	0.565	2.128	0	1
21019	4609236	purA	0.529	3.487	0	1
12013	4609538	purA	0.628	2.632	0	1
6204	4609638	purA	0.474	2.248	0	1
1527	4609771	purA	0.518	2.602	0	1
17852	4610040	purA	0.431	1.944	0	1
488	4610458	yjeB	0.615	2.796	0	1

12054	4610563	yjeB	0.703	2.572	0	1
2149	4610685	yjeB, vacB	0.792	2.604	0	1
18996	4611029	vacB	0.935	1.136	0	0
12260	4611373	vacB	1.061	1.300	0	0
16896	4611481	vacB	0.980	1.348	0	0
18646	4611642	vacB	0.830	0.916	0	0
16995	4611867	vacB	0.874	0.992	0	0
16232	4612203	vacB	0.997	1.123	0	0
20473	4612479	vacB	1.196	1.190	0	0
7155	4612659	vacB	1.197	1.211	0	0
13732	4612947	vacB	1.080	1.171	0	0
11533	4613046	vacB	0.968	0.975	0	0
8310	4613295	vacB, yjfH	0.991	0.932	0	0
14005	4613568	yjfH	1.537	1.014	0	0
5334	4613644	yjfH	1.776	1.178	0	0
14161	4613829	yjfH	1.401	0.945	0	0
19151	4614354	yjfI	4.210	0.750	1	0
11881	4614594	yjfI, yjfJ	6.116	0.536	1	0
6187	4614785	yjfI, yjfJ	6.000	0.627	1	0
7815	4614867	yjfJ	5.528	0.542	1	0
18443	4615040	yjfJ	4.451	0.530	1	0
7242	4615156	yjfJ, STM4372	3.023	0.529	1	0
7498	4615545	STM4372	1.576	0.581	0	0
6580	4615714	STM4372	1.220	0.629	0	0
2180	4615851	STM4372	1.558	0.691	0	0
7861	4616323	STM4372, yjfK	1.296	0.674	0	0
4627	4616499	STM4372, yjfK	1.343	0.836	0	0
10510	4616595	STM4372, yjfK	1.188	0.875	0	0
16806	4616918	yjfK, yjfL	1.777	0.655	0	0
6155	4617272	yjfL, yjfM	2.952	0.631	1	0
17984	4617417	yjfL, yjfM	3.892	0.651	1	0
16654	4617697	yjfM	2.630	0.746	1	0
17878	4617984	yjfM, yjfC	1.509	0.535	0	0
13302	4618072	yjfM, yjfC	1.514	0.707	0	0
16725	4618411	yjfC	1.566	0.789	0	0
12039	4618494	yjfC	1.280	0.816	0	0

12632	4618856	yjfC	1.391	0.760	0	0
6615	4619099	yjfC	1.515	0.872	0	0
13373	4619274	yjfC, aidB	1.251	0.741	0	0
6002	4619461	yjfC, aidB	1.359	0.820	0	0
375	4619628	aidB	1.090	0.754	0	0
4139	4619806	aidB	1.082	0.822	0	0
18055	4620224	aidB	1.005	0.856	0	0
6073	4620354	aidB	1.291	1.565	0	0
18770	4620584	aidB	1.188	1.186	0	0
13395	4620898	aidB	0.568	1.674	0	0
22283	4621026	aidB	0.749	2.890	0	1
3033	4621318	yjfN	0.502	2.664	0	1
13805	4621515	yjfN	0.521	2.484	0	1
4985	4621663	yjfN, yjfO	0.574	2.412	0	1
3480	4622015	yjfN, yjfO	0.522	1.552	0	0
11079	4622086	yjfO, yjfP	0.694	1.549	0	0
4008	4622169	yjfO, yjfP	0.828	1.462	0	0
9983	4622422	yjfO, yjfP	0.980	1.547	0	0
19178	4622951	yjfQ	0.907	1.566	0	0
1469	4623211	yjfQ	0.965	1.210	0	0
11920	4623293	yjfQ	1.012	1.045	0	0
8482	4623670	yjfQ	0.918	0.623	0	0
8480	4623795	yjfQ, yjfR	0.959	0.657	0	0
5509	4623923	yjfQ, yjfR	0.988	0.742	0	0
1106	4624003	yjfQ, yjfR	0.931	0.772	0	0
14060	4624138	yjfQ, yjfR	0.847	0.662	0	0
7490	4624824	yjfR, sgaT	0.892	0.593	0	0
17334	4624938	yjfR, sgaT	0.969	0.498	0	0
18526	4625156	yjfR, sgaT	0.953	0.596	0	0
16429	4625234	yjfR, sgaT	0.945	0.621	0	0
20012	4625658	sgaT	1.006	0.696	0	0
8425	4625907	sgaT	1.080	0.750	0	0
814	4625979	sgaT	1.324	0.749	0	0
15920	4626275	sgaT, sgaB	1.258	0.775	0	0
2734	4626583	sgaT, sgaB	1.408	0.742	0	0
11683	4626685	sgaB, ptxA	1.515	0.737	0	0

6767	4626908	sgaB, ptxA	1.497	0.742	0	0
3388	4627295	ptxA, sgaH	1.360	0.661	0	0
2948	4627474	sgaH	1.495	1.192	0	0
2490	4627644	sgaH, sgaU	1.206	0.622	0	0
7117	4627741	sgaH, sgaU	1.582	0.771	0	0
16496	4628077	sgaU	0.959	0.591	0	0
20689	4628386	sgaU	1.801	1.017	0	0
11373	4628607	sgaU, sgaE	1.271	0.675	0	0
2336	4628814	sgaU, sgaE	1.384	0.788	0	0
15359	4628948	sgaE	1.270	0.886	0	0
13560	4629300	sgaE	1.498	1.071	0	0
13789	4629558	sgaE	1.287	0.816	0	0
21796	4629793	yjfY, STM4390	1.263	0.878	0	0
8165	4629921	yjfY, STM4390	0.899	0.956	0	0
7224	4630218	yjfY, STM4390	0.647	1.780	0	1
15639	4630289	yjfY, STM4390	0.728	2.628	0	1
11255	4630493	rpsF, priB	0.597	2.974	0	1
6131	4630852	rpsF, priB	0.706	2.241	0	1
3188	4630960	priB, rpsR	0.514	1.818	0	1
11714	4631196	priB, rpsR	0.624	2.498	0	1
12966	4631582	rplI	0.593	1.610	0	0
18128	4631725	rplI, yifZ	0.697	1.630	0	0
6992	4631881	rplI, yifZ	0.929	1.442	0	0
5592	4632248	yifZ	0.707	0.695	0	0
19291	4632493	yifZ	1.079	0.794	0	0
21752	4632686	yifZ	1.005	0.805	0	0
20676	4632918	yifZ	1.392	1.276	0	0
15878	4633159	ytfB	1.020	1.424	0	0
21408	4633298	ytfB	0.980	1.809	0	1
4051	4633499	ytfB, fklB	0.935	1.824	0	1
11712	4633747	ytfB, fklB	0.997	1.275	0	0
7252	4633906	ytfB, fklB	0.884	1.166	0	0
16941	4634184	fklB	1.122	1.304	0	0
19876	4634596	cycA	0.937	1.317	0	0
10647	4634754	cycA	1.067	1.319	0	0
11946	4634825	cycA	0.773	1.001	0	0

5678	4635065	cycA	0.671	0.896	0	0
17530	4635300	cycA	0.910	1.143	0	0
14651	4635594	cycA	1.035	1.291	0	0
18336	4635918	cycA	0.797	0.976	0	0
2188	4635991	cycA	0.974	1.404	0	0
4619	4636087	cycA	0.652	0.944	0	0
10909	4636420	ytfE	0.963	1.334	0	0
15825	4636606	ytfE	1.054	1.281	0	0
14041	4636997	ytfE	1.266	0.939	0	0
13062	4637091	ytfE	1.330	0.908	0	0
18882	4637284	ytfE, ytfF	1.388	1.094	0	0
13480	4637733	ytfF	1.349	0.945	0	0
3620	4637857	ytfF	1.028	0.928	0	0
9109	4638087	ytfF	1.293	0.877	0	0
22281	4638351	ytfF, ytfG	1.164	0.616	0	0
17468	4638624	ytfG, ytfH	1.323	0.764	0	0
21080	4638785	ytfG, ytfH	1.235	0.646	0	0
14138	4639010	ytfG, ytfH	0.896	0.908	0	0
4732	4639085	ytfG, ytfH	1.487	1.094	0	0
7964	4639547	cpdB	1.133	0.878	0	0
11386	4639633	cpdB	1.360	1.032	0	0
12587	4639886	cpdB	1.268	0.871	0	0
14462	4640039	cpdB	1.156	0.925	0	0
21209	4640360	cpdB	1.092	0.813	0	0
12286	4640688	cpdB	1.318	0.858	0	0
3311	4640897	cpdB	1.158	0.837	0	0
3473	4640985	cpdB	1.100	0.942	0	0
11531	4641366	cpdB, cysQ	0.925	0.903	0	0
4880	4641508	cpdB, cysQ	1.068	1.226	0	0
14994	4641621	cpdB, cysQ	0.932	1.152	0	0
17766	4641729	cpdB, cysQ	0.920	1.249	0	0
19570	4642248	cysQ	0.915	1.242	0	0
14945	4642454	cysQ	0.978	1.499	0	0
611	4642659	ytfJ	1.022	1.502	0	0
11468	4642976	ytfJ, ytfK	0.910	0.940	0	0
4603	4643169	ytfJ, ytfK	0.946	1.083	0	0

11447	4643261	ytfJ, ytfK	0.870	1.112	0	0
18503	4643472	ytfJ, ytfK	0.795	1.111	0	0
2100	4643786	ytfL	0.711	1.146	0	0
21532	4644041	ytfL	0.741	1.033	0	0
6089	4644213	ytfL	0.720	0.832	0	0
18076	4644375	ytfL	0.716	0.952	0	0
15247	4644698	ytfL	0.909	1.152	0	0
16871	4644883	ytfL	0.715	1.229	0	0
9535	4645047	ytfL	0.804	1.457	0	0
1345	4645122	ytfL	0.958	1.746	0	0
15584	4645241	ytfL, msrA	0.654	1.162	0	0
7837	4645829	msrA, ytfM	0.799	1.035	0	0
13573	4646026	msrA, ytfM	0.845	1.182	0	0
12545	4646250	msrA, ytfM	0.864	1.603	0	0
4851	4646510	ytfM	0.918	1.055	0	0
3726	4646615	ytfM	0.954	0.983	0	0
5776	4647024	ytfM	1.203	1.350	0	0
6297	4647154	ytfM	0.869	1.106	0	0
371	4647524	ytfM, ytfN	1.322	1.425	0	0
20150	4647730	ytfM, ytfN	1.292	1.324	0	0
13177	4647811	ytfN	0.726	0.978	0	0
12369	4648195	ytfN	1.140	1.230	0	0
5977	4648438	ytfN	1.101	1.184	0	0
13859	4648673	ytfN	1.344	1.190	0	0
18149	4648899	ytfN	1.367	1.483	0	0
11456	4648991	ytfN	1.364	1.439	0	0
3934	4649258	ytfN	1.101	0.979	0	0
10869	4649575	ytfN	0.987	1.127	0	0
13777	4649824	ytfN	1.457	1.565	0	0
2827	4649930	ytfN	1.097	1.517	0	0
11453	4650221	ytfN	1.332	1.559	0	0
14614	4650453	ytfN	0.913	1.127	0	0
11322	4650546	ytfN	0.950	1.146	0	0
16902	4650810	ytfN	0.648	0.972	0	0
15458	4650935	ytfN	0.964	1.197	0	0
17413	4651296	ytfN, ytfP	0.684	1.331	0	0

8544	4651557	ytfP	0.707	1.510	0	0
21992	4651829	ytfP	0.975	1.590	0	0
8550	4652023	STM4412	1.399	1.472	0	0
5212	4652156	STM4412	1.734	1.360	0	0
19770	4652438	STM4412	2.027	0.808	0	0
9385	4652628	STM4412	2.263	0.704	0	0
12539	4652843	STM4412	3.477	0.691	1	0
1990	4653042	STM4412	4.106	0.602	1	0
10178	4653205	STM4412, STM4413	3.393	0.573	1	0
7463	4653292	STM4412, STM4413	3.169	0.569	1	0
9649	4653819	STM4413	2.126	0.531	0	0
9229	4653901	STM4413	2.035	0.599	0	0
6664	4654179	STM4413	3.276	0.684	0	0
1496	4654386	STM4413	3.236	0.530	0	0
15912	4654799	ppa	2.273	0.894	0	0
2689	4654890	ppa	0.919	0.640	0	0
17127	4655079	ppa	1.240	1.582	0	0
8292	4655249	ppa	1.060	1.425	0	0
2369	4655575	ppa, fbp	0.746	1.184	0	0
1594	4655848	fbp	0.831	1.219	0	0
9557	4656100	fbp	1.100	1.392	0	0
16981	4656177	fbp, mpl	0.944	1.349	0	0
16717	4656437	fbp, mpl	1.111	1.161	0	0
10732	4656653	fbp, mpl	0.932	1.055	0	0
10718	4656771	fbp, mpl	1.157	1.247	0	0
5760	4657210	mpl	1.088	1.161	0	0
16779	4657416	mpl	0.861	0.910	0	0
1014	4657728	mpl	1.325	1.177	0	0
20352	4657989	mpl	0.806	0.902	0	0
3456	4658164	STM4417	1.218	1.089	0	0
21410	4658256	STM4417	0.887	1.098	0	0
22212	4658591	STM4417	0.845	0.687	0	0
17296	4658696	STM4417	0.968	0.664	0	0
9598	4659061	STM4417	1.292	0.497	0	0
17848	4659257	STM4417	1.390	0.689	0	0
584	4659594	STM4418	1.967	0.712	0	0

6269	4659679	STM4418	1.628	0.593	0	0
11444	4659887	STM4418	1.318	0.582	0	0
5767	4660122	STM4418	0.999	0.671	0	0
3088	4660356	STM4418	1.155	0.685	0	0
3447	4660598	STM4418	0.856	0.699	0	0
16816	4660733	STM4418	0.796	0.637	0	0
2740	4661104	STM4418, STM4419	0.470	0.413	0	0
20032	4661279	STM4418, STM4419	0.359	0.447	0	0
454	4661387	STM4419	0.649	0.629	0	0
7699	4661749	STM4419	0.688	0.422	0	0
4248	4661972	STM4419	1.028	0.728	0	0
20482	4662188	STM4419	0.886	0.594	0	0
22227	4662505	STM4419	1.482	0.797	0	0
17429	4662600	STM4419	1.665	0.861	0	0
18058	4662984		1.783	0.716	0	0
7298	4663114	STM4420	1.946	0.702	0	0
22106	4663318	STM4420	1.565	0.852	0	0
22059	4663661	STM4420	1.373	0.967	0	0
7988	4663882	STM4420	1.334	0.853	0	0
14923	4664086	STM4420, STM4421	1.411	1.020	0	0
11650	4664321	STM4420, STM4421	1.315	0.869	0	0
1409	4664407	STM4421	1.298	0.773	0	0
22202	4664635	STM4421	1.016	0.614	0	0
7234	4664926	STM4421	0.902	0.689	0	0
14357	4665192	STM4421	0.926	0.617	0	0
18154	4665429	STM4421, STM4423	0.584	0.523	0	0
20354	4665673	STM4421, STM4422	0.540	0.417	0	0
1333	4665811	STM4421, STM4422	0.756	0.670	0	0
12113	4666089	STM4422, STM4423	1.610	0.532	0	0
1868	4666286	STM4423	2.501	0.530	1	0
22257	4666563	STM4423, STM4424	2.725	0.529	1	0
13570	4666804	STM4424	2.895	0.498	1	0
3273	4666937	STM4424	2.944	0.493	1	0
10287	4667236	STM4424	2.540	0.462	1	0
2830	4667353	STM4424	2.665	0.510	0	0
16756	4667646	STM4424, STM4425	3.474	0.619	1	0

5560	4667793	STM4424, STM4425	3.358	0.598	1	0
7249	4667890	STM4425	2.688	0.534	1	0
8155	4668344	STM4425	1.720	0.683	0	0
17025	4668563	STM4425, srfJ	1.413	0.594	0	0
15382	4668730	STM4425, srfJ	1.500	0.623	0	0
14470	4668848	STM4425, srfJ	1.615	0.581	0	0
8117	4668978	srfJ	1.429	0.558	0	0
17278	4669220	srfJ	1.819	0.647	0	0
10806	4669699	srfJ	1.431	0.862	0	0
22043	4669770	srfJ	1.627	0.917	0	0
21429	4670155	srfJ, STM4427	0.565	0.655	0	0
20355	4670438	STM4427	1.401	0.868	0	0
17677	4670676	STM4427	1.260	0.933	0	0
1263	4670917	STM4427	1.090	0.884	0	0
881	4671054	STM4427	1.294	1.107	0	0
1384	4671162	STM4427, STM4428	1.277	0.855	0	0
2967	4671564	STM4428	1.963	0.801	0	0
8825	4671636	STM4428	2.125	0.749	0	0
13521	4671947	STM4428	0.684	0.564	0	0
6000	4672240	STM4428	1.640	0.823	0	0
4772	4672313	STM4428	0.941	0.560	0	0
16538	4672739	STM4428, STM4429	1.609	0.776	0	0
13750	4672878	STM4429	1.654	0.750	0	0
2843	4673193	STM4429	1.629	0.677	0	0
14616	4673433	STM4429, STM4430	1.366	0.725	0	0
16296	4673678	STM4430	1.407	0.691	0	0
8319	4673890	STM4430	0.855	0.553	0	0
8451	4673988	STM4430	0.916	0.487	0	0
13857	4674315	STM4430, STM4431	0.704	0.556	0	0
3070	4674574	STM4430, STM4431	0.575	0.484	0	0
19640	4674662	STM4430, STM4431	0.564	0.565	0	0
2473	4674847	STM4431	0.795	0.566	0	0
1960	4675147	STM4431	1.032	0.610	0	0
18385	4675319	STM4431	1.060	0.635	0	0
19742	4675671	STM4431, STM4432	1.269	0.735	0	0
6608	4675916	STM4432	1.256	0.558	0	0

18724	4676136	STM4432	1.554	0.751	0	0
14564	4676343	STM4432	1.614	0.601	0	0
21836	4676555	STM4432, STM4433	2.522	0.728	1	0
15629	4676775	STM4433	4.231	0.600	1	0
1722	4676898	STM4433	2.496	0.627	0	0
6442	4677079	STM4433	5.221	0.637	0	0
17953	4677399	STM4433	2.098	0.585	0	0
7392	4677676	STM4433, STM4434	1.751	0.546	0	0
4271	4677941	STM4434	4.337	0.615	1	0
13655	4678036	STM4434	3.647	0.485	1	0
4748	4678420	STM4434	1.909	0.646	0	0
2410	4678671	STM4434	1.394	0.536	0	0
20671	4678757	STM4434	1.492	0.598	0	0
5545	4678893	STM4434, STM4435	1.401	0.520	0	0
13593	4679200	STM4435	3.820	0.675	1	0
7829	4679440	STM4435	4.172	0.525	1	0
22026	4679595	STM4435	3.798	0.610	1	0
17668	4679845	STM4435, STM4436	3.672	0.589	1	0
5279	4680184	STM4436	4.930	0.541	1	0
3248	4680255	STM4436	4.444	0.615	1	0
15752	4680498	STM4436	3.841	0.743	1	0
5570	4680697	STM4436	2.076	0.754	0	0
1229	4681026	STM4436	1.092	0.790	0	0
20190	4681242	yjgA	1.254	1.460	0	0
16972	4681603	yjgA, pmbA	1.394	2.203	0	1
13942	4681718	yjgA, pmbA	1.343	2.089	0	1
18832	4681805	yjgA, pmbA	1.259	1.801	0	1
8668	4682279	pmbA	1.292	1.051	0	0
3262	4682356	pmbA	1.123	1.083	0	0
17823	4682669	pmbA	1.045	0.813	0	0
8191	4682866	pmbA, cybC	1.688	0.850	0	0
3907	4683111	pmbA, cybC	1.688	0.915	0	0
5429	4683375	cybC	2.294	1.116	0	0
13846	4683502	cybC, STM4440	2.124	1.202	0	0
6735	4683722	STM4440	1.337	0.839	0	0
1613	4683885	STM4440, STM4441	1.555	0.782	0	0

19035	4684254	STM4441, STM4442	1.461	0.732	0	0
11998	4684545	STM4441, STM4442	1.111	0.604	0	0
3246	4684632	STM4442, STM4443	1.340	0.809	0	0
13493	4684914	STM4442, STM4443	1.384	0.600	0	0
5236	4685130	STM4443	1.158	0.737	0	0
4475	4685429	STM4443, STM4444	1.301	0.759	0	0
11097	4685675	STM4443, STM4444	1.677	0.795	0	0
6382	4685760	STM4444	1.460	0.755	0	0
3624	4686127	STM4444, STM4445	1.541	0.715	0	0
18641	4686359	STM4444, STM4445	1.511	0.721	0	0
18245	4686504	STM4445	1.358	0.680	0	0
13579	4686800	STM4445	1.773	0.853	0	0
22139	4686973	STM4445	0.797	0.688	0	0
9739	4687226	STM4445, STM4446	1.652	0.776	0	0
9301	4687396	STM4445, STM4446	0.992	0.610	0	0
1256	4687717	STM4446	1.941	0.678	0	0
7116	4687854	STM4446	1.736	0.678	0	0
10592	4688041	STM4446	1.313	0.768	0	0
17031	4688245	STM4446, STM4447	1.145	0.678	0	0
2221	4688678	STM4447	1.491	0.730	0	0
17274	4688812	STM4447	1.662	0.832	0	0
4608	4689127	STM4447, STM4448	1.301	0.757	0	0
1814	4689308	STM4448	1.428	0.775	0	0
13516	4689437	STM4448	1.026	0.646	0	0
17212	4689755	STM4448	1.722	1.055	0	0
8472	4689855	STM4448	1.476	0.905	0	0
5970	4690052	STM4448	1.049	0.676	0	0
8336	4690459	STM4448	1.352	0.915	0	0
21828	4690581	STM4448	1.117	0.871	0	0
7983	4690832	STM4448	1.078	0.766	0	0
7385	4691049	STM4448, STM4449	1.288	0.932	0	0
6186	4691352	STM4449, STM4450	1.107	1.088	0	0
4585	4691581	STM4449, STM4450	1.032	1.165	0	0
1751	4691781	STM4450	0.898	1.512	0	0
17340	4691935		0.998	1.728	0	0
4196	4692234	nrdG	0.893	1.202	0	0

3613	4692395	nrdG	0.731	1.076	0	0
5151	4692529	nrdG, nrdD	1.073	1.016	0	0
727	4692786	nrdG, nrdD	1.358	0.901	0	0
11930	4693206	nrdD	1.166	0.641	0	0
16075	4693387	nrdD	1.271	0.774	0	0
10324	4693553	nrdD	1.378	0.639	0	0
13639	4693634	nrdD	1.218	0.610	0	0
823	4694137	nrdD	1.186	0.642	0	0
9508	4694316	nrdD	1.017	0.642	0	0
11483	4694599	nrdD	1.045	0.653	0	0
16920	4694671	nrdD	1.110	0.699	0	0
12467	4694883	nrdD	0.999	0.604	0	0
2274	4695000	nrdD	0.835	0.548	0	0
13119	4695354	treC	1.231	0.721	0	0
22136	4695590	treC	0.573	0.604	0	0
17511	4695886	treC	1.445	0.729	0	0
18422	4696108	treC	1.438	0.689	0	0
1954	4696231	treC	1.368	0.735	0	0
9104	4696414	treC	1.435	0.654	0	0
19464	4696788	treC	1.197	0.769	0	0
1399	4697038	treC	1.468	0.907	0	0
19143	4697215		1.245	0.766	0	0
21962	4697440		1.372	0.902	0	0
14772	4697702		1.507	0.826	0	0
10083	4697865		1.194	0.790	0	0
7554	4698199		0.928	0.701	0	0
20875	4698312		1.217	0.890	0	0
6708	4698444	treR	1.014	0.838	0	0
6199	4698686	treR	0.780	0.820	0	0
2212	4699066	treR	0.855	1.529	0	0
15349	4699359	treR, mgtA	0.660	3.245	0	0
1441	4699566	treR, mgtA	0.560	1.870	0	0
17291	4699711	treR, mgtA	0.690	2.440	0	0
18342	4699838	mgtA	0.503	0.748	0	0
17681	4700181	mgtA	1.370	1.008	0	0
7865	4700382	mgtA	1.203	0.760	0	0

18364	4700601	mgtA	1.255	0.879	0	0
21376	4700826	mgtA	1.430	0.841	0	0
6165	4700921	mgtA	1.516	0.725	0	0
19947	4701276	mgtA	1.450	0.843	0	0
22130	4701438	mgtA	0.528	0.564	0	0
17131	4701785	mgtA	1.095	0.988	0	0
2601	4702016	mgtA	0.567	0.830	0	0
19785	4702296	mgtA	1.085	1.114	0	0
6984	4702505	STM4457	1.332	1.282	0	0
10499	4702712	STM4457	1.045	1.695	0	0
9005	4702899	STM4457	0.975	1.528	0	0
20103	4703163	STM4457, yjgF	0.948	1.503	0	0
14007	4703377	STM4457, yjgF	0.920	1.042	0	0
19903	4703508	yjgF, pyrI	1.184	1.144	0	0
1812	4703746	yjgF, pyrI	0.930	0.854	0	0
10359	4703929	pyrI	1.020	0.831	0	0
19953	4704254	pyrI, pyrB	0.738	0.625	0	0
3903	4704596	pyrB, yjgG	0.976	1.264	0	0
9412	4704829	pyrB, yjgG	1.049	1.645	0	0
20742	4704924	pyrB, yjgG	1.024	1.650	0	0
1794	4705072	pyrB, yjgG	0.978	1.625	0	0
14715	4705310	pyrB, pyrL	1.238	1.176	0	0
6541	4705389	pyrL, STM4463	1.156	1.000	0	0
15130	4705639	STM4463	1.233	0.767	0	0
16714	4706080	STM4464	1.190	0.899	0	0
10827	4706260	STM4464	1.213	0.840	0	0
7140	4706551	STM4464	0.719	0.504	0	0
10153	4706829	STM4464	1.597	0.789	0	0
2826	4706908	STM4464	1.140	0.727	0	0
16537	4707139	STM4464	1.407	0.606	0	0
14885	4707449	STM4464, STM4465	1.520	0.695	0	0
8062	4707576	STM4464, STM4465	1.342	0.594	0	0
2363	4707907	STM4465	1.246	0.611	0	0
14276	4708094	STM4465	1.539	0.783	0	0
19578	4708215	STM4465	1.343	0.556	0	0
7434	4708370	STM4465, STM4466	1.659	0.704	0	0

17183	4708909	STM4466	1.552	0.717	0	0
21793	4709067	STM4466	1.616	0.805	0	0
10725	4709301	STM4466, STM4467	1.422	0.621	0	0
18217	4709403	STM4466, STM4467	1.509	0.705	0	0
7243	4709685	STM4466, STM4467	1.983	0.563	0	0
14668	4709899	STM4467	2.378	0.460	0	0
14234	4710057	STM4467	3.581	0.478	1	0
1910	4710426	STM4467	2.969	0.474	1	0
19196	4710501	STM4467	3.603	0.506	1	0
2060	4710616	STM4467	3.553	0.494	1	0
21530	4711130	yjgK	3.548	0.490	1	0
8970	4711201	yjgK	2.562	0.565	1	0
17545	4711402	yjgK	2.287	0.663	1	0
8598	4711508	yjgK	1.841	0.722	0	0
7938	4711833	argI	0.959	0.833	0	0
5775	4712268	argI	1.067	0.653	0	0
17638	4712339	argI	1.176	0.693	0	0
1526	4712611	argI, yjgD	0.748	0.681	0	0
10507	4712866	argI, yjgD	0.845	0.679	0	0
9218	4713219	yjgD, miaE	0.991	0.879	0	0
343	4713380	miaE	0.917	0.902	0	0
17912	4713472	miaE	1.169	0.900	0	0
1024	4713692	miaE	1.290	0.732	0	0
17426	4713962	miaE	1.720	0.882	0	0
16781	4714354		2.940	0.617	1	0
13898	4714546	ytgA	4.227	0.520	1	0
17812	4714630	ytgA	3.931	0.584	1	0
9191	4714708	ytgA	3.614	0.548	1	0
2192	4715194	ytgA, yjgM	3.018	0.735	1	0
17757	4715320	yjgM, yjgN	3.338	0.667	1	0
17842	4715526	yjgM, yjgN	3.954	0.730	1	0
13979	4715753	yjgM, yjgN	3.847	0.680	1	0
3989	4716037	yjgN	3.298	0.586	1	0
5689	4716307	yjgN	2.426	0.605	1	0
6669	4716589	yjgN	1.688	0.843	0	0
11152	4716819	yjgN	1.319	1.163	0	0

1186	4717040	valS	1.088	1.362	0	0
4036	4717129	valS	1.105	1.448	0	0
18645	4717514	valS	1.226	1.764	0	0
4022	4717696	valS	1.034	1.344	0	0
20383	4717928	valS	1.053	1.976	0	0
19938	4718175	valS	1.052	1.613	0	0
18164	4718277	valS	1.288	1.804	0	0
20879	4718591	valS	0.736	1.436	0	0
15345	4718866	valS	0.914	1.664	0	1
17578	4719112	valS	1.032	2.094	0	1
3600	4719364	valS	1.057	1.781	0	1
5187	4719553	valS	0.882	2.110	0	1
8823	4719822	valS	0.797	2.391	0	1
11183	4719931	valS, holC	1.127	2.957	0	1
19856	4720227	valS, holC	0.830	2.094	0	1
12379	4720303	valS, holC	0.736	1.482	0	0
16358	4720428	holC, pepA	0.911	2.018	0	0
12439	4720629	holC, pepA	0.816	1.348	0	0
18529	4721070	pepA	1.142	1.779	0	0
5254	4721176	pepA	0.933	1.416	0	0
10583	4721512	pepA	0.902	1.480	0	0
2174	4721631	pepA	0.789	1.664	0	1
11570	4721931	pepA, yjgP	0.748	2.153	0	1
2338	4722252	pepA, STM4478	1.135	2.300	0	1
19451	4722400	STM4478, yjgP	0.849	1.944	0	1
6932	4722566	yjgP	0.770	1.734	0	0
445	4722923	yjgP	0.848	1.960	0	0
18610	4723004	yjgP, yjgQ	0.871	1.514	0	0
20148	4723447	yjgQ	1.075	1.462	0	0
3588	4723606	yjgQ	0.769	1.157	0	0
5675	4723896	yjgQ	1.115	1.323	0	0
15195	4724053	yjgQ	1.014	1.223	0	0
20634	4724216	yjgQ	1.074	1.514	0	0
4321	4724489	yjgQ	0.993	1.258	0	0
14679	4724762	idnR	1.524	1.111	0	0
9569	4724939	idnR	1.074	0.772	0	0

18111	4725124	idnR	1.495	0.791	0	0
8048	4725299	idnR	1.427	0.743	0	0
19878	4725405	idnR	1.397	0.823	0	0
10251	4725897	idnR, idnT	1.526	0.780	0	0
13455	4726086	idnR, idnT	1.609	0.764	0	0
19250	4726297	idnT	1.347	0.657	0	0
22117	4726563	idnT	1.439	0.723	0	0
15198	4726720	idnT	1.494	0.688	0	0
17867	4727024	idnT	2.088	0.744	0	0
21317	4727195	idnT, idnO	2.122	0.617	0	0
13999	4727298	idnT, idnO	1.832	0.674	0	0
8558	4727666	idnO	2.161	0.658	0	0
4353	4727889	idnO	2.604	0.693	1	0
11431	4728115	idnO, idnD	2.273	0.660	1	0
18070	4728262	idnO, idnD	2.100	0.577	0	0
19462	4728651	idnD	3.974	0.448	1	0
9294	4728737	idnD, idnK	4.042	0.452	1	0
2502	4729071	idnD, idnK	3.174	0.561	1	0
10009	4729366	idnD, idnK	3.718	0.565	1	0
4761	4729467	idnK	3.625	0.638	1	0
15126	4729633	idnK	2.425	0.688	1	0
19862	4729872	yjgB	1.534	0.829	0	0
2243	4730037	yjgB	1.260	0.811	0	0
12289	4730279	yjgB	1.269	0.974	0	0
5698	4730611	yjgB, leuX	0.929	1.940	0	0
509	4730780	yjgB, leuX	1.266	2.492	0	1
16898	4731121	yjgB, STM4488	0.533	2.652	0	1
19152	4731208	yjgB, STM4488	0.571	2.557	0	1
19636	4731434	STM4488	0.781	1.193	0	0
17857	4731819	STM4489	0.914	0.628	0	0
1146	4731931	STM4489	0.925	0.609	0	0
19183	4732298	STM4489	1.151	0.726	0	0
6841	4732371	STM4489	0.986	0.699	0	0
19510	4732607	STM4489	1.055	0.619	0	0
10755	4732924	STM4489	1.832	0.705	0	0
19149	4733191	STM4489	1.890	0.562	0	0

7254	4733397	STM4489	2.149	0.504	0	0
21339	4733555	STM4489	2.336	0.599	0	0
19653	4733849	STM4489	1.567	0.721	0	0
933	4734011	STM4489	1.583	0.628	0	0
7017	4734365	STM4489	1.698	0.709	0	0
9354	4734436	STM4489	1.897	0.742	0	0
9166	4734581	STM4489	1.468	0.698	0	0
12672	4734900	STM4489	1.273	0.700	0	0
11107	4735166	STM4489, STM4490	1.207	0.658	0	0
10944	4735265	STM4489, STM4490	1.005	0.545	0	0
14920	4735497	STM4490	0.881	0.722	0	0
14657	4735808	STM4490	0.984	0.754	0	0
334	4736123	STM4490	1.020	0.879	0	0
746	4736297	STM4490	1.198	1.022	0	0
5251	4736556		1.479	0.930	0	0
14642	4736653	STM4491	1.397	0.711	0	0
14977	4736968	STM4491	1.162	1.087	0	0
5286	4737280	STM4491	1.141	1.063	0	0
19450	4737413	STM4491	1.028	1.027	0	0
20163	4737739	STM4491	0.940	1.023	0	0
2249	4737967	STM4491	0.926	1.021	0	0
21723	4738043	STM4491	1.025	1.048	0	0
9687	4738394	STM4491	0.897	1.041	0	0
20008	4738596	STM4491	0.747	0.895	0	0
6599	4738812	STM4491, STM4492	0.813	0.915	0	0
2870	4738927	STM4491, STM4492	0.823	0.820	0	0
5606	4739345	STM4492	1.040	0.970	0	0
1394	4739585	STM4492	1.052	0.947	0	0
9173	4739679	STM4492	1.173	0.947	0	0
1355	4739930	STM4492	1.141	1.046	0	0
17665	4740074	STM4492	1.257	1.144	0	0
4823	4740343	STM4492	1.136	1.023	0	0
16061	4740433	STM4492	0.995	1.022	0	0
5548	4740832	STM4492	0.487	0.556	0	0
16688	4741077	STM4492	1.091	1.136	0	0
14721	4741367	STM4492, STM4493	0.916	0.935	0	0

21665	4741482	STM4492, STM4493	1.070	1.175	0	0
11508	4741625	STM4492, STM4493	0.897	0.976	0	0
2208	4741869	STM4493	0.877	0.961	0	0
20573	4742191	STM4493, STM4494	0.943	1.084	0	0
1518	4742511	STM4493, STM4494	0.751	1.093	0	0
18614	4742588	STM4493, STM4494	0.795	1.141	0	0
2837	4742787	STM4494	0.897	1.315	0	0
8338	4742895	STM4494	0.942	1.189	0	0
9934	4743152	STM4494	1.254	1.323	0	0
12447	4743605	STM4494, STM4495	1.105	0.736	0	0
17087	4743738	STM4495	1.464	0.683	0	0
794	4744096	STM4495	2.134	0.482	0	0
9905	4744268	STM4495	2.601	0.488	1	0
15683	4744531	STM4495	2.567	0.591	1	0
2269	4744698	STM4495	2.689	0.424	1	0
11471	4744828	STM4495	2.478	0.516	1	0
1347	4745200	STM4495	2.879	0.522	1	0
6290	4745323	STM4495	3.082	0.586	1	0
1759	4745652	STM4495	2.501	0.794	0	0
1255	4745868	STM4495	3.542	0.771	1	0
14971	4745942	STM4495	3.217	0.781	1	0
1044	4746216	STM4495	1.925	0.783	0	0
13336	4746599	STM4495	1.271	0.675	0	0
3452	4746818	STM4495	1.168	0.852	0	0
12402	4746891	STM4495	0.771	0.612	0	0
6587	4747125	STM4495, STM4496	1.239	0.844	0	0
9679	4747500	STM4496	0.631	0.417	0	0
17802	4747692	STM4496	1.498	1.032	0	0
13539	4747868	STM4496	1.476	0.917	0	0
6739	4748206	STM4496	1.480	1.086	0	0
786	4748355	STM4496	1.415	1.081	0	0
11894	4748588	STM4496	0.909	0.740	0	0
9430	4748721	STM4496	1.088	0.900	0	0
9956	4749136	STM4496	0.948	0.780	0	0
11317	4749231	STM4496	1.031	0.929	0	0
2137	4749408	STM4496	0.916	0.792	0	0

19012	4749788	STM4496	1.001	0.863	0	0
19379	4749875	STM4496	0.868	0.775	0	0
11985	4750120	STM4496	0.771	0.697	0	0
761	4750264	STM4496	0.924	0.833	0	0
16909	4750355	STM4496	0.951	0.891	0	0
20349	4750558	STM4496	1.087	0.884	0	0
7896	4750836	STM4496, STM4497	0.908	0.966	0	0
7891	4751379	STM4497, STM4498	0.875	0.973	0	0
1601	4751482	STM4497, STM4498	0.845	0.935	0	0
5523	4751810	STM4498	1.059	1.167	0	0
759	4751919	STM4498	1.307	1.090	0	0
20038	4752023	STM4498	1.137	0.930	0	0
1690	4752503		1.766	0.546	0	0
3521	4752725	yeeN	2.077	0.501	0	0
4798	4752902	yeeN	2.269	0.526	0	0
7849	4753059	yeeN	2.605	0.589	0	0
19453	4753215	yeeN	2.529	0.626	0	0
2540	4753329	yeeN	3.040	0.626	1	0
14164	4753589	yeeN	2.537	0.612	1	0
8283	4753858		2.475	0.796	1	0
1656	4754365	yjhP	1.586	0.796	0	0
9786	4754459	yjhP	1.136	0.615	0	0
15625	4754638	yjhP	1.263	0.690	0	0
1253	4754914	yjhP	1.275	0.697	0	0
13434	4755038	yjhP	1.227	0.710	0	0
20323	4755127	yjhP	1.182	0.698	0	0
7430	4755624	yjhP, STM4501	0.955	1.385	0	0
1861	4755830	yjhP, STM4501	0.843	1.604	0	0
16912	4756029	STM4501, STM4502	1.306	1.592	0	0
21132	4756293	STM4502	1.436	0.835	0	0
8422	4756456	STM4502	1.679	0.657	0	0
3108	4756579	STM4502	2.086	0.649	0	0
20118	4756990	STM4503	1.901	0.496	0	0
22157	4757195	STM4503	2.333	0.564	1	0
16952	4757358	STM4503	2.591	0.574	1	0
2839	4757651	STM4503	2.494	0.416	0	0

17080	4757788	STM4503	2.524	0.500	1	0
8905	4757969	STM4503, STM4504	2.572	0.502	1	0
5287	4758206	STM4504	2.134	0.503	0	0
19018	4758447	STM4504	2.379	0.481	0	0
14375	4758751	STM4504	2.518	0.471	0	0
21805	4759023	STM4504	2.794	0.476	0	0
19621	4759244	STM4504, STM4505	2.537	0.547	0	0
18933	4759529	STM4505	2.860	0.448	1	0
5437	4759610	STM4505	2.686	0.679	1	0
5901	4759923	STM4505	1.437	0.706	0	0
2441	4760007	STM4506	1.396	0.765	0	0
19332	4760370	STM4506	1.069	0.857	0	0
21072	4760677	STM4506, uxuR	1.118	0.860	0	0
11704	4760853	STM4506, uxuR	1.131	0.966	0	0
686	4760969	STM4506, uxuR	1.120	1.173	0	0
16452	4761047	STM4506, uxuR	1.053	1.008	0	0
20059	4761529	uxuR	1.299	1.339	0	0
817	4761739	uxuR	1.125	1.154	0	0
10765	4761899	trpS2	1.210	1.121	0	0
5392	4762093	trpS2	0.980	0.764	0	0
2945	4762315	trpS2	1.317	0.708	0	0
6286	4762464	trpS2	1.643	0.843	0	0
21922	4762810	trpS2	1.847	0.964	0	0
12112	4762978	trpS2	3.400	0.957	1	0
7952	4763071	trpS2, STM4509	3.992	0.903	1	0
8717	4763534	STM4509	3.396	0.520	1	0
1627	4763682	STM4509	3.253	0.758	1	0
2852	4763762	STM4509	2.897	0.719	1	0
20712	4764256	STM4510	1.414	0.610	0	0
9833	4764404	STM4510	1.059	0.640	0	0
14708	4764554	STM4510	1.414	0.753	0	0
18808	4764778	STM4510	1.390	0.800	0	0
8214	4765116	yjiE	1.509	1.092	0	0
657	4765276	yjiE	1.563	0.966	0	0
3592	4765511	yjiE	1.077	0.822	0	0
7250	4765905	yjiE	1.478	1.068	0	0

12630	4766077	yjiE, iadA	1.648	1.079	0	0
13190	4766167	yjiE, iadA	1.546	0.946	0	0
16664	4766609	iadA	1.373	0.737	0	0
5994	4766777	iadA	1.464	0.763	0	0
16110	4767049	iadA	1.690	0.961	0	0
8022	4767247	iadA	1.896	1.005	0	0
17354	4767326	iadA, yjiG	1.601	0.917	0	0
12615	4767691	iadA, yjiG	1.405	0.790	0	0
18321	4767806	yjiG, yjiH	1.032	0.627	0	0
7395	4768214	yjiH	1.735	0.652	0	0
537	4768417	yjiH	1.838	0.632	0	0
18833	4768643	yjiH	2.689	0.600	0	0
12158	4768751	yjiH	1.918	0.507	0	0
16922	4769135		2.432	0.618	1	0
13685	4769260	yjiJ	2.289	0.739	1	0
17273	4769422	yjiJ	2.197	0.861	1	0
4252	4769542	yjiJ	1.895	0.926	0	0
16721	4769765	yjiJ	1.689	1.082	0	0
4932	4770043	yjiJ	1.421	1.012	0	0
8988	4770446	yjiJ	1.553	1.079	0	0
3382	4770672	yjiJ, yjiN	1.664	0.955	0	0
16812	4770759	yjiJ, yjiN	1.600	0.989	0	0
18681	4771045	yjiN	1.348	0.833	0	0
10093	4771130	yjiN	1.507	0.854	0	0
3724	4771385	yjiN	1.374	0.705	0	0
1552	4771659	yjiN	1.927	0.842	0	0
11245	4771950	yjiN	0.951	0.493	0	0
3072	4772211	yjiN, yjiO	1.114	0.630	0	0
18550	4772422	yjiO	1.259	0.717	0	0
9712	4772729	yjiO	0.589	0.647	0	0
19327	4772872	yjiO	1.239	0.723	0	0
2960	4773127	yjiO	1.588	0.850	0	0
13158	4773258	yjiO	1.764	0.926	0	0
17885	4773551	yjiO, STM4518	1.085	0.719	0	0
20448	4773686	yjiO, STM4518	1.629	0.865	0	0
1706	4773948	STM4518	0.922	0.653	0	0

20558	4774318	STM4519	1.471	0.903	0	0
21340	4774423	STM4519	1.453	0.847	0	0
12431	4774735	STM4519	1.275	0.695	0	0
1420	4774819	STM4519	1.422	0.751	0	0
15089	4775152	STM4519	1.385	0.671	0	0
3824	4775260	STM4519	1.327	0.649	0	0
21363	4775641	STM4519, yjiS	1.908	0.637	0	0
4092	4775881	yjiS	2.337	0.693	0	0
15108	4776143	yjiS, STM4520	3.112	0.691	1	0
8972	4776319	STM4520, STM4522	2.616	0.508	1	0
12641	4776455	STM4520, STM4522	2.847	0.462	0	0
13420	4776767	STM4522	2.788	0.508	1	0
5557	4776909	STM4522	2.994	0.542	1	0
15710	4777285	STM4522, yjiW	2.494	0.561	1	0
13679	4777362	STM4522, yjiW	2.232	0.558	0	0
3290	4777498	yjiW	2.309	0.550	0	0
14513	4777855	hsdS	2.189	0.582	0	0
10894	4778085	hsdS	3.108	0.332	1	0
11754	4778283	hsdS	3.723	0.455	1	0
18546	4778421	hsdS	3.561	0.488	1	0
8671	4778535	hsdS	3.410	0.491	1	0
10932	4778839	hsdS	3.577	0.441	1	0
19493	4778931	hsdS	3.494	0.588	1	0
15935	4779492	hsdM	1.373	0.766	0	0
15105	4779705	hsdM	0.962	0.673	0	0
14675	4779875	hsdM	1.069	0.743	0	0
8146	4780004	hsdM	1.382	0.821	0	0
4043	4780320	hsdM	0.856	0.720	0	0
19349	4780562	hsdM	1.166	1.339	0	0
4539	4780737	hsdM, hsdR	1.303	1.448	0	0
905	4780818	hsdM, hsdR	1.418	1.687	0	0
3497	4781344	hsdR	1.870	1.013	0	0
11902	4781606	hsdR	1.219	0.845	0	0
17868	4781801	hsdR	1.525	0.943	0	0
1400	4781918	hsdR	1.622	1.127	0	0
9432	4782288	hsdR	0.905	0.796	0	0

12689	4782511	hsdR	1.515	0.968	0	0
19354	4782632	hsdR	1.419	0.898	0	0
1478	4782785	hsdR	1.770	0.861	0	0
14957	4783137	hsdR	1.059	0.842	0	0
19429	4783273	hsdR	1.290	0.921	0	0
8863	4783585	hsdR	1.132	1.052	0	0
22132	4783713	hsdR	1.032	1.046	0	0
7754	4784137	hsdR, mrr	1.219	0.918	0	0
14006	4784366	hsdR, mrr	1.201	0.858	0	0
11004	4784450	hsdR, mrr	1.051	0.783	0	0
11509	4784762	mrr	1.228	0.938	0	0
22215	4784906	mrr	1.141	0.922	0	0
14785	4785207	mrr, STM4528	1.212	1.152	0	0
12899	4785330	mrr, STM4528	1.075	1.204	0	0
12391	4785622	STM4528, STM4529	0.918	0.954	0	0
10638	4785824	STM4528, STM4529	0.966	1.099	0	0
9821	4785898	STM4528, STM4529	0.934	0.917	0	0
18623	4786281	yjiA	1.059	1.375	0	0
11061	4786542	yjiA	1.114	1.076	0	0
10344	4786632	yjiA	1.345	0.997	0	0
5120	4786883	yjiA	1.472	1.462	0	0
1607	4787213	yjiA	1.186	1.028	0	0
11238	4787325	yjiA, yjiX	0.810	0.841	0	0
14750	4787665	yjiA, yjiX	0.489	0.708	0	0
14373	4787792	yjiX, yjiY	1.143	0.769	0	0
10782	4788116	yjiY	1.653	0.885	0	0
15365	4788333	yjiY	1.243	0.620	0	0
14712	4788501	yjiY	0.632	0.714	0	0
3230	4788759	yjiY	1.293	0.739	0	0
2655	4789000	yjiY	0.666	0.588	0	0
6353	4789157	yjiY	0.972	0.744	0	0
7352	4789520	yjiY	1.315	0.836	0	0
19880	4789760	yjiY, tsr	0.818	0.990	0	0
12400	4789910	yjiY, tsr	0.987	1.536	0	0
6823	4790032	yjiY, tsr	1.062	1.895	0	1
6097	4790420	tsr	1.090	1.587	0	1

21904	4790595	tsr	0.898	1.392	0	0
2950	4790718	tsr	0.887	1.285	0	0
14410	4790818	tsr	0.851	1.092	0	0
4830	4791326	tsr	0.948	1.334	0	0
8724	4791481	tsr	0.869	1.091	0	0
16856	4791722	STM4534	0.850	1.301	0	0
19423	4791941	STM4534	0.908	1.277	0	0
11264	4792083	STM4534	1.280	1.328	0	0
4304	4792251	STM4534	1.180	1.152	0	0
17558	4792370	STM4534	1.630	1.139	0	0
14413	4792816	STM4534	1.575	1.061	0	0
13376	4792979	STM4534	1.621	1.071	0	0
3978	4793337	STM4534	1.456	0.837	0	0
18544	4793483	STM4534	1.698	0.892	0	0
13164	4793787	STM4534	1.443	0.786	0	0
13425	4794031	STM4534	1.255	0.772	0	0
9086	4794270	STM4534	1.601	0.892	0	0
18982	4794577	STM4534, STM4535	1.793	0.737	0	0
10509	4794808	STM4535	2.275	0.850	0	0
8152	4794887	STM4535, STM4536	2.839	0.814	1	0
2166	4795217	STM4535, STM4536	3.488	0.708	1	0
7939	4795358	STM4536, STM4537	4.275	0.642	1	0
4513	4795688	STM4536, STM4537	4.016	0.694	1	0
14707	4795806	STM4537	3.755	0.631	1	0
1742	4795902	STM4537	3.376	0.673	1	0
3992	4796357	STM4537, STM4538	5.348	0.614	1	0
5137	4796476	STM4537, STM4538	4.651	0.631	1	0
12958	4796769	STM4538	2.786	0.566	1	0
3697	4796896	STM4538	2.131	0.693	0	0
2733	4797272	STM4538, STM4539	2.543	0.729	0	0
7564	4797461	STM4539	2.123	0.604	0	0
11493	4797662	STM4539	2.424	0.664	1	0
14398	4797866	STM4539	2.309	0.759	1	0
7675	4798152	STM4539, STM4540	1.018	0.497	0	0
17719	4798390	STM4539, STM4540	2.177	0.725	0	0
19592	4798547	STM4540	2.574	0.781	0	0

2025	4798792	STM4540	3.376	0.743	1	0
15124	4798938	STM4540	3.258	0.920	1	0
1869	4799356	STM4540	1.315	1.303	0	0
19020	4799523	STM4540	1.018	1.284	0	0
15041	4799692	mdoB	0.978	1.558	0	0
5472	4799806	mdoB	0.745	1.062	0	0
20064	4800132	mdoB	1.016	1.477	0	0
15426	4800459	mdoB	0.956	1.390	0	0
12997	4800543	mdoB	0.992	1.390	0	0
2148	4800748	mdoB	0.887	1.579	0	0
4037	4801038	mdoB	0.882	1.370	0	0
11748	4801142	mdoB	0.992	1.778	0	0
999	4801506	mdoB	1.017	1.510	0	0
5930	4801828	mdoB	1.231	1.883	0	0
10798	4801959	mdoB	0.849	1.299	0	0
8219	4802146	mdoB	1.309	1.433	0	0
3564	4802437	yjjA	0.935	0.956	0	0
15917	4802705	yjjA	1.088	1.050	0	0
15223	4802939	yjjA, dnaC	1.061	1.045	0	0
889	4803083	yjjA, dnaC	1.024	1.143	0	0
2082	4803273	dnaC	1.210	1.073	0	0
18193	4803516	dnaC, dnaT	1.185	0.927	0	0
5427	4803843	dnaC, dnaT	1.041	1.258	0	0
14966	4804047	dnaT	1.218	1.320	0	0
11291	4804264	dnaT, STM4545	1.306	1.190	0	0
21736	4804585	STM4545	1.376	1.083	0	0
3710	4804721	STM4545, yjjP	1.549	0.871	0	0
16750	4804855	STM4545, yjjP	1.508	0.934	0	0
14634	4805176	yjjP	1.504	0.675	0	0
18798	4805349	yjjP	1.609	0.678	0	0
19460	4805468	yjjP	2.060	0.590	0	0
21780	4805778	yjjP, yjjQ	3.774	0.423	1	0
8445	4806122	yjjQ	3.976	0.577	1	0
2401	4806340	yjjQ, bglJ	3.005	0.601	0	0
3197	4806508	yjjQ, bglJ	3.698	0.568	1	0
12445	4806760	yjjQ, bglJ	4.242	0.545	1	0

7078	4806973	bglJ	3.020	0.763	1	0
21621	4807237	bglJ	2.096	1.227	0	0
21175	4807325	bglJ	1.627	1.158	0	0
13057	4807728	STM4549	1.009	1.079	0	0
11318	4807972	STM4549	1.144	1.159	0	0
15182	4808066	STM4549	0.591	0.751	0	0
3304	4808338	STM4549, fhuF	1.061	0.896	0	0
16001	4808414	STM4549, fhuF	0.861	0.792	0	0
13332	4808898	fhuF	0.897	0.747	0	0
6746	4809073	fhuF, STM4551	0.706	0.689	0	0
3759	4809205	fhuF, STM4551	0.745	0.782	0	0
19651	4809407	STM4551	1.032	0.804	0	0
5407	4809776	STM4551	1.019	0.885	0	0
21127	4809911	STM4551, STM4552	0.940	0.784	0	0
5006	4810092	STM4551, STM4552	0.714	1.218	0	0
16731	4810452	STM4551, STM4552	0.517	2.455	0	1
8513	4810558	STM4552	0.470	3.211	0	1
12199	4810691	leuV	0.441	3.515	0	1
505	4811010	leuV, leuP	0.517	4.205	0	1
7614	4811197	leuP, leuQ	0.595	2.479	0	1
12446	4811362	leuQ, rsmC	0.798	2.047	0	1
4528	4811650	rsmC	0.779	1.226	0	0
10078	4811870	rsmC, holD	1.058	1.573	0	0
13583	4812067	rsmC, holD	1.087	1.720	0	0
8296	4812154	rsmC, holD	0.927	1.659	0	0
9864	4812626	holD, rimI	0.720	1.388	0	0
9259	4812722	rimI, yjjG	0.712	0.929	0	0
3334	4813106	yjjG	0.827	1.051	0	0
10097	4813443	yjjG, prfC	1.018	1.325	0	0
18826	4813628	yjjG, prfC	0.974	1.433	0	0
16119	4813863	prfC	1.100	1.605	0	0
5158	4814046	prfC	0.968	1.450	0	0
14741	4814258	prfC	0.956	1.482	0	0
18210	4814442	prfC	0.833	0.949	0	0
3015	4814758	prfC	1.000	1.406	0	0
21959	4814855	prfC	1.059	1.381	0	0

7435	4815071	prfC	1.054	1.166	0	0
12374	4815404	prfC, osmY	0.673	0.775	0	0
9871	4815683	osmY	1.026	0.917	0	0
9415	4815824	osmY	0.948	0.841	0	0
22348	4816101	osmY	0.957	0.779	0	0
14209	4816227	osmY, STM4562	1.053	0.714	0	0
21040	4816601	STM4562, yjjU	1.176	1.065	0	0
16272	4816758	yjjU	1.464	1.130	0	0
2018	4817035	yjjU	1.148	1.575	0	0
12220	4817176	yjjU	1.119	1.622	0	0
3319	4817331	yjjU	1.281	1.572	0	0
2222	4817614	yjjU, yjjV	1.390	1.362	0	0
3351	4817760	yjjU, yjjV	1.264	1.267	0	0
5140	4818169	yjjV	1.424	1.360	0	0
5370	4818300	yjjV	1.436	1.561	0	0
10887	4818678	yjjV	1.296	1.262	0	0
1416	4818897	yjjW	0.712	0.807	0	0
17649	4819055	yjjW	1.061	0.849	0	0
13853	4819292	yjjW	1.562	0.777	0	0
15799	4819598	yjjW, yjjI	1.447	0.665	0	0
873	4819671	yjjW, yjjI	1.234	0.795	0	0
11794	4819873	yjjW, yjjI	1.450	0.765	0	0
9852	4820229	yjjI	1.154	0.623	0	0
11862	4820443	yjjI	1.143	0.613	0	0
16832	4820517	yjjI	0.958	0.635	0	0
14093	4820838	yjjI	0.854	0.555	0	0
18773	4821149	yjjI, deoC	0.711	0.503	0	0
13558	4821302	yjjI, deoC	1.146	0.648	0	0
10534	4821461	yjjI, deoC	0.906	0.660	0	0
5982	4821830	deoC	1.231	0.711	0	0
21867	4821903	deoC, deoA	1.315	0.812	0	0
18598	4822297	deoA	1.132	0.835	0	0
15069	4822368	deoA	1.262	0.782	0	0
11263	4822532	deoA	1.285	0.958	0	0
21081	4822743	deoA	0.454	0.404	0	0
4729	4822960	deoA	1.337	1.266	0	0

14748	4823341	deoA, deoB	1.054	2.645	0	1
13029	4823613	deoA, deoB	0.612	4.576	0	1
5343	4823690	deoB	0.618	5.695	0	1
16219	4823866	deoB	0.677	3.521	0	1
12952	4824235	deoB	0.770	1.093	0	0
1032	4824562	deoB	1.221	1.210	0	0
13220	4824807	deoB, deoD	1.066	1.096	0	0
6687	4824885	deoB, deoD	0.409	0.391	0	0
18090	4825121	deoD	0.908	0.943	0	0
17596	4825342	deoD	0.545	0.726	0	0
7131	4825481	deoD	0.987	1.015	0	0
3464	4825897	STM4571	0.932	1.177	0	0
15278	4826116	STM4571	0.767	0.623	0	0
18330	4826272	STM4571	1.278	1.052	0	0
16031	4826455	STM4571, stjB	1.170	1.102	0	0
15562	4826776	STM4571, stjB	1.305	0.984	0	0
16145	4826936	stjB	1.446	0.946	0	0
2966	4827094	stjB	1.412	0.757	0	0
13229	4827249	stjB	1.322	0.716	0	0
551	4827441	stjB	1.505	0.850	0	0
5170	4827768	stjB	1.306	0.728	0	0
15400	4828203	stjB	1.442	0.815	0	0
16645	4828350	stjB	1.485	0.820	0	0
16476	4828491	stjB	1.527	0.773	0	0
5264	4828820	stjB	3.051	0.677	1	0
17698	4829048	stjB, stjC	3.485	0.634	1	0
7197	4829366	stjC	3.056	0.591	1	0
15188	4829533	stjC	2.727	0.572	1	0
5385	4829640	stjC	2.555	0.526	0	0
9519	4829826	stjC, STM4574	2.328	0.671	0	0
10683	4830125	STM4574	1.418	0.724	0	0
304	4830377	STM4574	1.782	0.720	0	0
20778	4830600	STM4574, STM4575	1.657	0.678	0	0
19066	4830865	STM4575	3.213	0.620	1	0
21573	4830971	STM4575	3.856	0.588	1	0
16334	4831378	STM4575	4.164	0.676	1	0

2614	4831575	lplA	3.246	0.952	1	0
10745	4831811	lplA	1.836	0.841	0	0
11450	4831885	lplA	1.930	0.966	0	0
9719	4832139	lplA	2.054	1.023	0	0
22282	4832374	lplA	1.633	1.147	0	0
7110	4832445	lplA, smp	1.446	1.201	0	0
21630	4832697	lplA, smp	1.786	1.470	0	0
11981	4833202	smp, serB	1.634	2.245	0	1
20545	4833361	smp, serB	1.445	1.765	0	1
16333	4833559	serB	1.507	1.374	0	0
9606	4833908	serB, radA	1.441	1.196	0	0
11848	4834073	serB, radA	1.498	1.327	0	0
20793	4834256	radA	1.386	1.631	0	0
17280	4834467	radA	1.154	1.359	0	0
4860	4834766	radA	1.311	1.433	0	0
2607	4834940	radA	1.257	1.469	0	0
5506	4835130	radA	0.934	1.181	0	0
6758	4835328	radA, nadR	1.247	1.547	0	0
21656	4835603	radA, nadR	0.893	1.423	0	0
18168	4835774	nadR	0.885	1.478	0	0
3301	4835878	nadR	1.142	1.675	0	0
14512	4836195	nadR	1.002	1.099	0	0
19660	4836446	nadR	1.183	1.280	0	0
10389	4836692	nadR	1.242	1.204	0	0
5328	4836921	nadR	1.013	1.736	0	0
18665	4837217	yjjK	0.729	1.993	0	1
8847	4837529	yjjK	0.894	1.821	0	1
17710	4837684	yjjK	0.778	1.454	0	0
1296	4837878	yjjK	1.179	1.549	0	0
265	4838086	yjjK	1.428	1.514	0	0
5443	4838400	yjjK	1.000	2.072	0	1
4889	4838481	yjjK	1.007	1.989	0	1
14597	4838836	yjjK, slt	0.920	2.718	0	1
1529	4838997	yjjK, slt	0.910	2.431	0	1
20006	4839215	yjjK, slt	1.148	1.602	0	0
15429	4839413	slt	1.173	1.129	0	0

12192	4839663	slt	1.394	1.003	0	0
11484	4839814	slt	1.277	0.969	0	0
13791	4840121	slt	1.471	1.157	0	0
19233	4840315	slt	1.296	1.142	0	0
10255	4840683	slt, trpR	0.777	0.768	0	0
7665	4840893	slt, trpR	0.901	1.032	0	0
10949	4840991	trpR	0.864	1.379	0	0
640	4841307	trpR	1.070	1.665	0	0
9205	4841431	yjjX	1.054	1.580	0	0
3945	4841562	yjjX, gpmB	0.844	1.149	0	0
10804	4841929	yjjX, gpmB	1.097	1.419	0	0
17696	4842000	yjjX, gpmB	1.385	1.636	0	0
10600	4842454	gpmB	0.557	1.229	0	0
5319	4842719	rob	0.753	2.123	0	1
8064	4842983	rob	0.899	1.987	0	1
10799	4843055	rob	0.953	1.927	0	1
19954	4843267	rob, creA	1.040	1.737	0	0
13280	4843513	rob, creA	1.294	1.157	0	0
17098	4843818	rob, creA	1.081	0.864	0	0
2354	4844043	creA, creB	0.868	0.827	0	0
1141	4844216	creB	1.109	1.193	0	0
17871	4844462	creB, creC	0.492	0.718	0	0
20638	4844773	creB, creC	0.923	1.100	0	0
14896	4844855	creB, creC	1.162	1.218	0	0
2527	4845190	creC	1.032	0.760	0	0
19580	4845379	creC	1.585	1.103	0	0
14422	4845668	creC	0.985	0.689	0	0
2648	4845873	creC	1.340	1.038	0	0
596	4846040	creC, creD	1.781	1.273	0	0
15362	4846319	creD	0.874	0.786	0	0
14742	4846604	creD	1.272	0.753	0	0
13211	4846677	creD	1.240	0.699	0	0
8359	4846779	creD	1.173	0.827	0	0
3531	4847255	creD	1.018	0.595	0	0
8623	4847392	creD	1.072	0.610	0	0
13990	4847680	creD	1.552	0.620	0	0

8069	4847949	sthE	1.145	0.473	0	0
21411	4848207	sthE	1.444	0.848	0	0
3166	4848283	sthE	1.462	0.787	0	0
4675	4848662	sthE	1.234	0.770	0	0
12643	4848885	sthE	1.586	0.665	0	0
15558	4849020	sthE, sthD	1.418	0.673	0	0
14205	4849153	sthE, sthD	1.535	0.658	0	0
8776	4849462	sthD	1.212	0.638	0	0
5812	4849718	sthD, sthB	1.707	0.590	0	0
19608	4849871	sthD, sthB	1.559	0.599	0	0
4320	4850162	sthB	1.505	0.645	0	0
10409	4850433	sthB	1.202	0.476	0	0
17874	4850564	sthB	1.377	0.591	0	0
8684	4850729	sthB	1.150	0.576	0	0
15678	4850894	sthB	1.264	0.567	0	0
5617	4851336	sthB	1.991	0.598	0	0
16370	4851500	sthB	2.416	0.624	1	0
8801	4851742	sthB	2.890	0.616	1	0
21330	4852027	sthB	3.385	0.547	1	0
11390	4852216	sthB, sthA	3.387	0.592	1	0
3162	4852415	sthB, sthA	3.387	0.603	1	0
18699	4852611	sthA	2.414	0.536	1	0
15640	4852835	sthA, STM4595	1.626	0.511	0	0
15319	4853217	sthA, STM4595	2.974	0.645	1	0
19986	4853306	STM4595	3.135	0.571	1	0
21735	4853521	STM4595	3.084	0.567	1	0
17007	4853849	STM4596	3.403	0.651	1	0
3674	4853959	STM4596	2.517	0.570	0	0
17924	4854120	STM4596	2.994	0.653	1	0
17288	4854264	STM4596	2.945	0.617	1	0
19224	4854585	STM4596	1.963	0.613	0	0
17099	4854662	STM4596	1.882	0.623	0	0
